# Supplementary material for: Diverse synthesis of α-tertiary amines and tertiary alcohols via desymmetric reduction of malonic esters
Source: Nat Commun. 2022 Aug 13;13:4759. doi: 10.1038/s41467-022-32560-1 (PMC9376102; doi:10.1038/s41467-022-32560-1)
Supplement: Supplementary file 1 — Supplementary Information [file 41467_2022_32560_MOESM1_ESM.pdf]

Supplementary Information

Diverse Synthesis of α-Tertiary Amines and Tertiary Alcohols via Desymmetric Reduction of Malonic Esters

Haichao Liu[1], Vincent Ho Man Lau[1], Pan Xu[1], Tsz Hin Chan[1], Zhongxing Huang*[1]

[1]State Key Laboratory of Synthetic Chemistry, Department of Chemistry, University of Hong Kong, Hong Kong, China

Correspondence to: huangzx@hku.hk

Table of Contents

**1**	**Supplementary methods**	**S3**
	1.1 General Information	S3
	1.2 Preparation and Characterization of Ligands	S4
	1.3 Preparation and Characterization of Substrates	S9
	1.4 Procedures for the Desymmetrization of Amino/Oxymalonic esters and Characterization of Chiral Products	S39
	1.5 Procedures for the Application of the Desymmetrization Products	S125
	1.6 Copies of NMR Spectra	S139
**2**	**Supplementary references**	**S276**

1. Supplementary methods

1.1 General Information.

Unless stated otherwise, all reactions were run under nitrogen or argon atmosphere. Toluene and THF were prepared by distillation over sodium-benzophenone ketyl prior to use. $ZnEt_2$ (1.0 M in hexane), $(MeO)_3SiH$, and $Et_3N\cdot3HF$ were purchased from J&K Chemical Ltd., Energy Chemical, and Shanghai Macklin Biochemical Co., Ltd, respectively. These three chemicals were used as received. Thin layer chromatography (TLC) was run on silica gel plates purchased from Yantai Jiangyou Silica gel Development Co., Ltd.

^{1}H NMR and ^{13}C NMR spectra were obtained on a Bruker 400 or 500 spectrometer (400/500 MHz for ^{1}H, 100/125 MHz for ^{13}C, 376 MHz for ^{19}F). Chemical shifts are reported as parts per million (ppm) with residual solvent signals as internal standard ($CHCl_3$, δ = 7.26 ppm for ^{1}H NMR, δ = 77.00 ppm for ^{13}C NMR; CH_3OH, δ = 3.31 ppm for ^{1}H NMR, δ = 49.00 ppm for ^{13}C NMR). Data for ^{1}H NMR were presented as following: chemical shifts (δ, ppm), multiplicity (br = broad, s = singlet, d = doublet, t = triplet, q = quartet, dd = doublet of doublets, tt = triplet of triplets, td = triplet of doublets, m = multiplet), coupling constant (Hz), and integration. Chiral HPLC spectra were measured on an Agilent 1260 Infinity II. The $[\alpha]_D$ were recorded using INESA SGW-531 Automatic Polarimeter. Infrared absorption spectra were recorded in chloroform on a PerkinElmer UATR two FT-IR and the data presented as per centimeter (cm^{-1}). High-resolution EI mass spectra were recorded on a Thermo Scientific DFS high resolution magnetic sector MS. High-resolution ESI-MS measurements were performed on a Bruker impact II high-resolution LC-QTOF mass spectrometer. Accurate masses from high-resolution mass spectra were reported for the molecular ion $[M+Na]^+$, or $[M+H]^+$.

1.2 Preparation and Characterization of Ligands

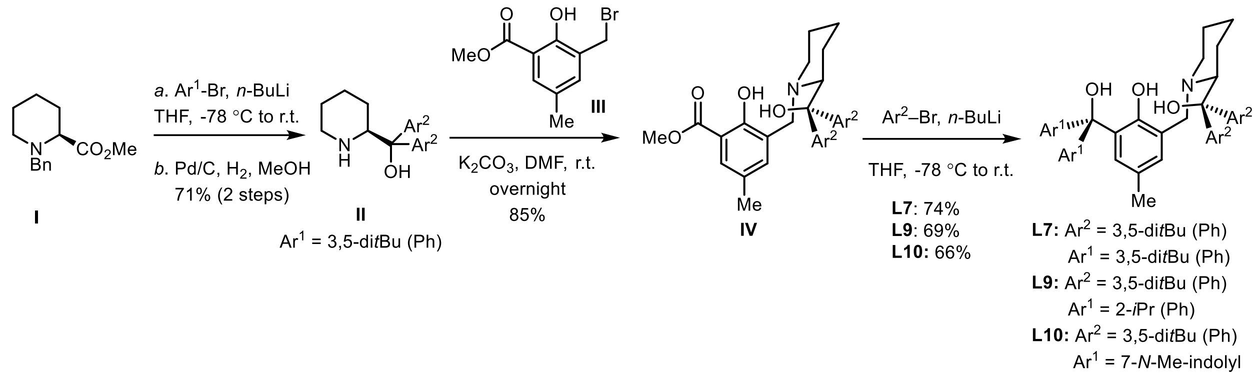

To a stirred solution of 1-bromo-3,5-di-^{t}butylbenzene (14.00 g, 52.5 mmol) in THF (100 ml) at -78 °C was added *n*-BuLi (22 ml, 2.4 M in hexane, 52.5 mmol) dropwise. The resulting suspension was stirred at -78 °C for 1 h before a solution of ester **I** (3.5 g, 15 mmol) in THF (0.3 M, based on ester) was added dropwise and then warmed to room temperature. After stirring at room temperature for 2 hours, the reaction was quenched with saturated NH_4Cl solution, extracted with EtOAc, washed with brine, dried over Na_2SO_4, and filtered. The filtrate was concentrated under vacuum and the crude product was used in the next step without purification.

To a stirred solution of the above crude product in MeOH (60 ml) was added Pd/C (1.5 g, 10% Pd/C with 55% H_2O). The mixture was stirred at room temperature under 1 atm hydrogen for 36 h. Then the mixture was filtered through Celite. The filtrate was concentrated under vacuum and submitted to flash column chromatography (hexane/EtOAc = 10:1 to 5:1 to 1:1) to yield **II** in 71% yield (5.25 g) as a white solid.

To a stirred solution of **II** (2.46 g, 5 mmol) in THF/DMF (6ml/12 mL) was added benzylbromide **III**[1](1.90 g, 7.5 mmol) and stirred for overnight. The resulting mixture was quenched with saturated NH_4Cl solution, extracted with EtOAc, washed with brine, dried over Na_2SO_4, and filtered. After concentrated, the residue was purified through flash chromatography on silica gel eluted with hexane/EtOAc (10:1) to afford intermediate **IV** (2.85 g, 85% yield) as white solid.

To a solution of aryl bromide (8 mmol) in THF (8 mL) at -78 °C was added *n*-BuLi (3.3 mL, 8 mmol) dropwise. The resulting suspension was stirred at -78 °C for 1 h before a solution of ester **IV** (669 mg, 1.0 mmol) in THF (4 mL) was added dropwise and then warmed to room temperature. After stirring at room temperature for 2 hours, the reaction was quenched with saturated NH_4Cl solution, extracted with EtOAc, washed with brine, dried over Na_2SO_4, and filtered. The filtrate was concentrated under vacuum and submitted to flash column chromatography (hexane/EtOAc = 10:1) to yield **L7** (753.1 mg, 74%), **L9** (612.2 mg, 69%), and **L10** (594.2 mg, 66%) as a white solid, respectively.

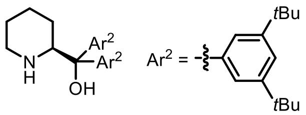

II

^{1}H NMR (400 MHz, $CDCl_3$) δ 7.55 (dd, *J* = 2.0 Hz, 1.6 Hz, 2H), 7.39 (dd, *J* = 2.0 Hz, 1.6 Hz, 2H), 7.26 (q, *J* = 2.0 Hz, 1H), 7.21 (q, *J* = 2.0 Hz, 1H), 4.13 (s, 1H), 3.49 (d, *J* = 10.0 Hz, 1H), 3.02 (d, *J* = 10.4 Hz, 1H), 2.70 (t, *J* = 11.2 Hz, 1H), 1.88-1.55 (m, 4H), 1.34 (s, 9H), 1.33 (s, 9H), 1.31 (s, 9H), 1.31 (s, 9H).

^{13}C NMR (100 MHz, $CDCl_3$) δ 150.32, 149.63, 145.54, 143.28, 120.43, 120.11, 119.90, 119.80, 79.23, 63.06, 46.80, 34.95, 34.86, 31.58, 31.56, 25.76, 25.51, 24.63.

IR (neat, cm^{-1}) 2959, 1360, 1181, 736, 710.

HRMS (ESI) calcd $C_{34}H_{54}NO^+$ $[M+H]^+$: 492.4200. Found: 492.4196.

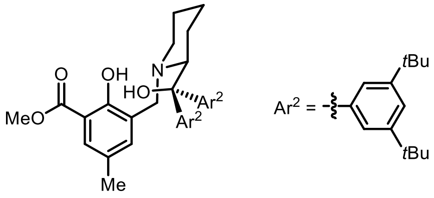

IV

^{1}H NMR (400 MHz, $CDCl_3$) δ 10.83 (br s, 1H), 7.61 (d, *J* = 1.2 Hz, 2H), 7.48 (d, *J* = 1.2 Hz, 1H), 7.45 (d, *J* = 1.2 Hz, 2H), 7.17 (s, 1H), 7.14 (s, 1H), 7.00 (d, *J* = 1.6 Hz, 1H), 5.48 (br s, 1H), 3.90 (s, 3H), 3.68 (d, *J* = 13.2 Hz, 1H), 3.59 (dd, *J* = 8.4, 4.8 Hz, 1H), 3.06 (d, *J* = 13.2 Hz, 1H), 2.78 (d, *J* = 12.4 Hz, 1H), 2.31-2.24 (m, 1H), 2.22 (s, 3H), 1.61-1.43 (m, 6H), 1.35 (s, 9H), 1.34 (s, 9H), 1.30 (s, 9H), 1.28 (s, 9H).

^{13}C NMR (100 MHz, $CDCl_3$) δ 170.73, 157.77, 149.68, 149.40, 148.31, 145.66, 137.62, 128.20, 127.74, 127.40, 120.55, 120.14, 119.57, 119.21, 111.47, 79.00, 67.99, 52.85, 52.13, 50.18, 34.87, 34.85, 31.55, 31.54, 31.48, 31.45, 25.83, 23.20, 22.37, 20.40.

IR (neat, cm^{-1}) 2943, 1673, 1440, 759, 697.

HRMS (ESI) calcd $C_{44}H_{64}NO_4^+$ $[M+H]^+$: 670.4830. Found: 670.4829.

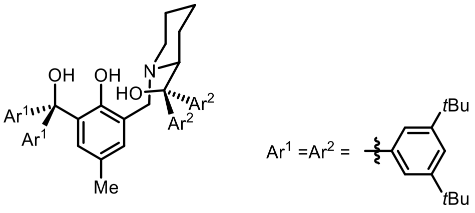

L7

^{1}H NMR (400 MHz, $CDCl_3$) δ 7.11 (d, *J* = 1.6 Hz, 2H), 7.38 (s, 2H), 7.28 (d, *J* = 2.0 Hz, 2H), 7.26 (s,

1H), 7.21 (d, J = 1.6 Hz, 2H), 6.90 (d, J = 1.6 Hz, 1H), 6.83 (s, 2H), 6.63 (d, J = 1.6 Hz, 1H), 5.96 (d, J = 1.6 Hz, 1H), 5.42 (br s, 1H), 4.26 (br s, 1H), 3.97 (d, J = 12.4 Hz, 1H), 3.82 (d, J = 10.0 Hz, 1H), 2.80 (d, J = 13.6 Hz, 1H), 2.46 (t, J = 11.2 Hz, 1H), 2.04 (s, 3H), 1.90-1.78 (m, 2H), 1.53-1.40 (m, 4H), 1.30 (s, 18H), 1.23 (s, 18H), 1.21 (s, 18H), 1.20 (s, 18H).

^{13}C NMR (100 MHz, $CDCl_3$) δ 153.22, 150.59, 150.43, 150.15, 148.80, 147.08, 146.42, 146.26, 133.09, 129.88, 127.80, 125.66, 123.15, 122.37, 121.29, 120.69, 120.61, 120.35, 120.33, 119.93, 119.67, 119.57, 83.27, 81.86, 68.28, 49.65, 34.94, 34.93, 34.91, 34.70, 34.68, 31.51, 31.48, 31.42, 24.90, 20.64.

IR (neat, cm^{-1}) 2953, 1597, 1476, 1361, 1247, 877, 716.

HRMS (ESI) calcd. for $C_{71}H_{104}NO_3^+$ $[M+H]^+$: 1018.8011, Found: 1018.7998.

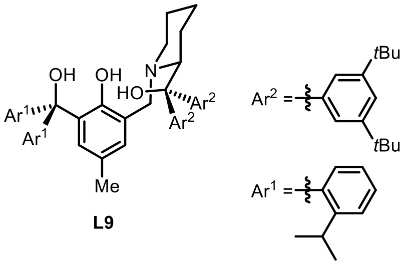

L9

^{1}H NMR (400 MHz, $CDCl_3$) δ 7.42-7.16 (m, 10H), 6.91-6.00 (m, 6H), 5.59-5.29 (m, 1H), 4.41-3.93 (m, 3H), 3.61-3.58 (m, 1H), 3.22 (m, 1H), 2.92-2.89 (m, 1H), 2.64-2.58 (m, 1H), 2.04-1.88 (m, 5H), 1.72-1.45 (m, 4H), 1.30-1.15 (m, 42H), 0.96 (m, 2H), 0.86 (d, J = 6.0 Hz, 6H).

^{13}C NMR (100 MHz, $CDCl_3$) δ 153.00, 150.54, 150.50, 150.34, 149.72, 145.82, 144.53, 143.72, 143.15, 142.83, 132.52, 129.53, 129.29, 128.50, 128.40, 127.68, 127.38, 127.06, 126.88, 126.48, 126.25, 123.62, 123.53, 122.25, 120.67, 120.38, 120.25, 119.61, 119.48, 119.27, 84.80, 81.97, 81.82, 68.11, 49.47, 34.95, 34.93, 34.83, 34.80, 31.51, 31.41, 31.28, 30.03, 29.61, 25.26, 23.22, 20.57.

IR (neat, cm^{-1}) 2953, 1597, 1472, 1246, 874, 757

HRMS (ESI) calcd. for $C_{61}H_{84}NO_3^+$ $[M+H]^+$: 878.6446, Found: 878.6435.

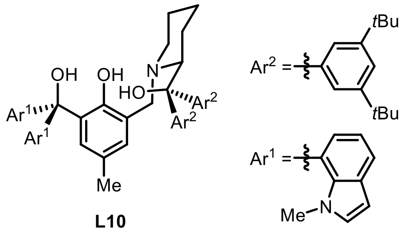

L10

^{1}H NMR (400 MHz, $CDCl_3$) δ 7.56 (t, J = 8.0 Hz, 2H), 7.41-7.32 (m, 6H), 7.23 (s, 1H), 7.13-6.69 (m, 3H), 6.52-6.50 (m, 3H), 6.31-6.20 (m, 2H), 4.10-3.72 (m, 4H), 3.57-3.54 (m, 3H), 3.24-3.22 (m, 3H),

3.01-2.88 (m, 1H), 2.55-2.44 (m, 1H), 2.14-1.84 (m, 5H), 1.57-1.44 (m, 4H), 1.28 (m, 18H), 1.16 (s, 12H), 1.03 (s, 6H).

^{13}C NMR (100 MHz, $CDCl_3$) δ 152.70, 150.59, 150.49, 150.28, 135.00, 134.93, 134.42, 134.35, 133.90, 131.93, 131.90, 131.76, 131.60, 131.56, 131.49, 131.44, 131.34, 131.20, 130.47, 130.16, 129.71, 128.87, 127.16, 126.85, 125.43, 125.27, 122.93, 122.84, 122.33, 121.07, 120.80, 120.60, 120.21, 119.99, 119.62, 119.42, 119.15, 117.90, 117.48, 117.22, 100.60, 84.35, 84.23, 82.35, 81.86, 67.79, 67.68, 60.37, 53.38, 47.10, 39.03, 38.90, 38.84, 34.93, 34.89, 34.76, 34.59, 31.57 31.47, 31.26, 31.08, 22.64, 20.65, 20.64, 19.92, 14.12.

IR (neat, cm^{-1}) 2954, 1475, 1215, 1090, 753.

HRMS (ESI) calcd. for $C_{61}H_{78}N_3O_3^+$ $[M+H]^+$: 900.6038, Found: 900.6019.

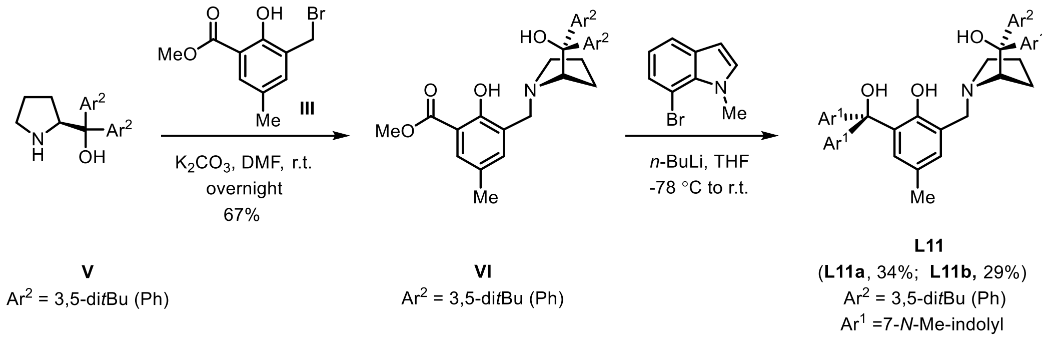

L11a and **L11b** were prepared from **V**[2] following the same procedure as described for the synthesis of **L10**.

VI was synthesized in 67% yield (1.21 g) with 2.77 mmol scale as a white solid and purified by flash column chromatography (hexane/EtOAc = 10:1). **L11a** and **b** were synthesized in a total 63% yield (**L11a**, 300 mg, 34%; **L11a**, 290 mg, 29%) from **VI** with a 1.0 mmol scale as brown solid. Purification by flash column chromatography (CH_2Cl_2/hexane = 4:1, then hexane/EtOAc = 20:1).

Note: L11a and **L11b** are a pair of atropisomers due to the rotation barrier of the triarylmethanol motif.

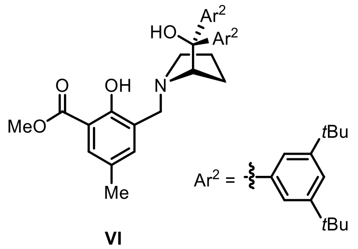

VI

^{1}H NMR (400 MHz, $CDCl_3$) δ 10.73 (br s, 1H), 7.64 (d, J = 1.6 Hz, 2H), 7.48 (d, J = 2.0 Hz, 1H), 7.46

(d, J = 2.0 Hz, 2H), 7.19 (dd, J = 2.0, 1.6 Hz, 1H), 7.13-7.12 (m, 2H), 5.19 (br s, 1H), 3.98 (dd, J = 9.2, 5.6 Hz, 1H), 3.90 (s, 3H), 3.29 (d, J = 13.6 Hz, 1H), 3.04 (d, J = 13.6 Hz, 1H), 2.91 (m, 1H), 2.40 (dt, J = 6.8, 9.6 Hz, 1H), 2.24 (s, 3H), 1.91-1.86 (m, 1H), 1.68-1.55 (m, 3H), 1.32 (s, 18H), 1.26 (s, 18H).

^{13}C NMR (100 MHz, $CDCl_3$) δ 170.80, 157.39, 149.76, 149.52, 147.19, 145.90, 136.68, 128.21, 127.96, 127.46, 120.17, 119.91, 119.73, 119.60, 111.32, 78.21, 71.98, 55.33, 53.45, 52.13, 34.86, 34.83, 31.57, 31.47, 29.66, 23.79, 20.42.

IR (neat, cm^{-1}) 2953, 1675, 1437, 1232, 879, 700.

HRMS (ESI) calcd $C_{43}H_{62}NO_4^+$ [M+H]$^+$: 656.4673. Found: 656.4668.

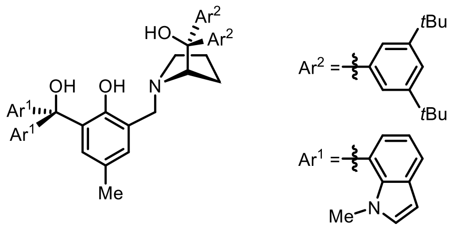

L11a (R_f = 0.55, hexane:EtOAc 10:1)

^{1}H NMR (400 MHz, $CDCl_3$) δ 7.59 (t, J = 6.8 Hz, 2H), 7.45 (s, 2H), 7.36 (s, 2H), 7.23 (m, 2H), 6.99 (d, J = 3.2 Hz, 1H), 6.93 (d, J = 2.8 Hz, 1H), 6.78 (q, J = 6.8 Hz, 2H), 6.69 (s, 1H), 6.54-6.53 (m, 3H), 6.31 (d, J = 7.2 Hz, 1H), 6.26 (s, 1H), 6.18 (br s, 1H), 3.96 (m, 1H), 3.82 (d, J = 13.2 Hz, 1H), 3.60 (s, 3H), 3.31 (d, J = 13.2 Hz, 1H), 2.85 (m, 1H), 2.44 (q, J = 8.4 Hz, 1H), 2.00 (m, 3H), 2.00-1.94 (m, 1H), 1.79-1.70 (m, 2H), 1.53-1.52 (m, 1H), 1.27-1.25 (m, 36H).

^{13}C NMR (100 MHz, $CDCl_3$) δ 152.52, 150.37, 150.09, 145.17, 144.61, 134.97, 134.35, 134.14, 132.16, 131.66, 131.62, 131.54, 130.21, 130.18, 128.50, 127.28, 125.34, 123.58, 122.22, 121.15, 120.97, 120.36, 120.32, 120.18, 119.97, 117.91, 117.34, 100.68, 100.61, 84.38, 80.42, 73.39, 60.53, 54.44, 39.11, 38.98, 34.85, 34.82, 31.42, 31.39, 29.01, 23.56, 20.63.

IR (neat, cm^{-1}) 2955, 1470, 1247, 1090, 754.

HRMS (ESI) calcd. for $C_{60}H_{76}N_3O_3^+$ [M+H]$^+$: 886.5881, Found: 886.5872.

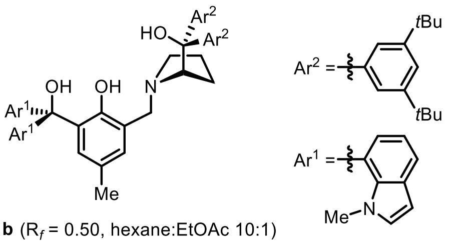

L11b (R_f = 0.50, hexane:EtOAc 10:1)

^{1}H NMR (400 MHz, $CDCl_3$) δ 7.58 (m, 2H), 7.40 (s, 2H), 7.35 (s, 2H), 7.21 (s, 1H), 7.08 (s, 1H), 6.95 (d,

J = 2.8 Hz, 1H), 6.90 (d, J = 3.2 Hz, 1H), 6.81-6.75 (m, 2H), 6.52-6.50 (m, 3H), 6.70 (s, 1H), 6.37 (d, J = 6.8 Hz, 1H), 6.31 (s, 1H), 4.03 (m, 1H), 3.96 (d, J = 12.8 Hz, 1H), 3.59 (s, 3H), 3.41 (d, J = 12.8 Hz, 1H), 3.22 (s, 3H), 2.74 (m, 1H), 2.46 (m, 1H), 2.00 (s, 3H), 2.00 (m, 1H), 1.84-1.71 (m, 2H), 1.56 (m, 1H), 1.26 (s, 9H), 1.25 (s, 9H), 1.15 (s, 9H), 1.14 (s, 9H).

^{13}C NMR (100 MHz, $CDCl_3$) δ 152.04, 150.44, 150.01, 144.91, 144.29, 134.82, 134.32, 133.59, 131.62, 131.56, 131.31, 130.89, 130.76, 128.69, 127.49, 125.23, 124.10, 122.90, 121.13, 120.98, 120.53, 120.44, 120.13, 119.97, 117.96, 117.93, 100.68, 84.58, 80.63, 80.42, 73.12, 60.68, 54.04, 38.93, 38.35, 34.82, 34.77, 31.42, 31.28, 28.76, 23.74, 20.61.

IR (neat, cm^{-1}) 2955, 1473, 1215, 1090, 754.

HRMS (ESI) calcd. for $C_{60}H_{76}N_3O_3^+$ $[M+H]^+$: 886.5881, Found: 886.5872.

1.3 Preparation and Characterization of Substrates

Method A

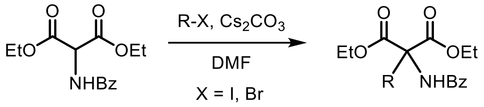

To a stirred solution of diethyl benzoylaminomalonate (100 mol%) in DMF (0.25 M) was added Cs_2CO_3 (300 mol%) and corresponding alkyl bromide or alkyl iodide (200 mol%). The reaction mixture was stirred at room temperature for 4-24h. The reaction mixture was extracted with EtOAc, washed with brine, dried over Na_2SO_4, and filtered. The filtrate was concentrated under vacuum and submitted to flash column chromatography (hexane/EtOAc) to yield the desired disubstituted aminomalonic esters.

Method B

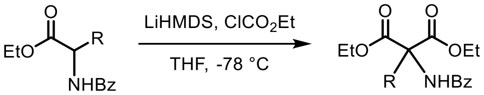

To a stirred solution of ethyl ester (100 mol%) in dry THF (0.25 M) was added LiHMDS (250 mol%, 1 M solution in THF) at -78 °C. The resulting mixture was stirred at -78 °C for 30 min and ethyl chloroformate (200 mol%) was added dropwise. Then the reaction was allowed to warm to room temperature. After stirring at room temperature for 1 h, the reaction mixture was quenched with saturated NH_4Cl solution, extracted with EtOAc, washed with brine, dried over Na_2SO_4, and filtered. The filtrate was concentrated under vacuum and submitted to flash column chromatography (hexane/EtOAc) to yield the desired disubstituted aminomalonic esters.

Method C

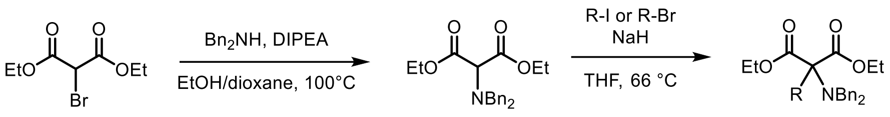

To a stirred solution of diethyl bromomalonate (100 mol%) and dibenzylamine (100 mol%) in EtOH/dioxnae (0.25 M, 1:1) was added diisopropylethylamine (200 ml%). The reaction mixture was stirred at 100℃ for 24 h. The mixture was then diluted with water, extracted with EtOAc, washed with brine, dried over Na_2SO_4, and filtered. The filtrate was concentrated under vacuum and submitted to flash column chromatography (hexane/EtOAc = 10:1) to yield dibenzylaminomalonic ester[1].

To a stirred solution of above product (100 mol%) in dry THF (0.25 M) was added NaH (200 mol%) at 0 ℃. After stirring for 10 min, the corresponding alkyl iodide or bromide (200 mol%) was added dropwise and the reaction mixture was stirred at 66 ℃ for 4 h. The mixture was then quenched with saturated NH_4Cl solution, extracted with EtOAc, washed with brine, dried over Na_2SO_4, and filtered. The filtrate was concentrated under vacuum and submitted to flash column chromatography (hexane/EtOAc) to yield the desired disubstituted aminomalonic esters.

<u>Method D</u>

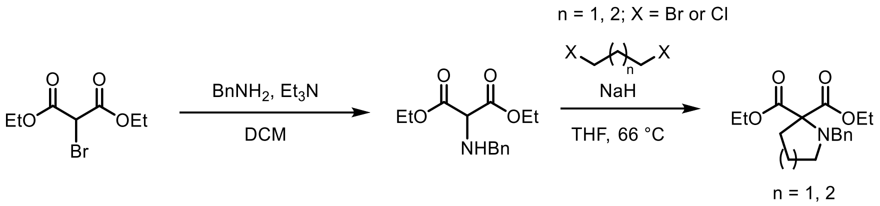

To a stirred solution of diethyl bromomalonate (100 mol%) and benzylamine (100 mol%) in DCM (0.25 M) was added Et_3N (200 ml%). The reaction mixture was stirred at room temperature for 24h. The mixture was then diluted with water, extracted with EtOAc, washed with brine, dried over Na_2SO_4, and filtered. The filtrate was concentrated under vacuum and submitted to flash column chromatography (hexane/EtOAc = 3:1) to yield diethyl 2-(benzylamino)malonate.

To a stirred solution of diethyl 2-(benzylamino)malonate (100 mol%) in THF (0.25 M) was added NaH (300 mol%) at 0 ℃. After stirring for 10 min, the corresponding dichloride or dibromide (200 mol%) was added dropwise and the reaction mixture was stirred at 66 ℃ for 4 h. The mixture was then quenched with saturated NH_4Cl solution, extracted with EtOAc, washed with brine, dried over Na_2SO_4, and filtered. The filtrate was concentrated under vacuum and submitted to flash column chromatography (hexane/EtOAc) to yield the desired cyclic malonic ester.

<u>Method E</u>

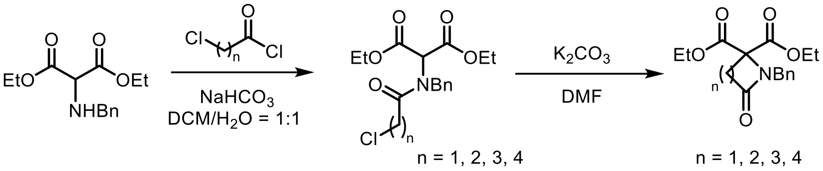

To a stirred solution of diethyl 2-(benzylamino)malonate (100 mol%) and $NaHCO_3$ (400 ml%) in DCM/H_2O (0.5 M, DCM/H_2O = 1:1) was added corresponding acid chloride (200 mol%, 0.5 M in DCM) dropwise. The reaction mixture was stirred at room temperature for 6 h. The mixture was then extracted with EtOAc, washed with brine, dried over Na_2SO_4, and filtered. The filtrate was concentrated under vacuum and the crude product was used in the next step without purification.

To a stirred solution of the above crude product in DMF (0.25 M) was added K_2CO_3 (400 ml%). The resulting reaction mixture was stirred at room temperature overnight. The mixture was then diluted with water, extracted with EtOAc, washed with brine, dried over Na_2SO_4, and filtered. The filtrate was concentrated under vacuum and submitted to flash column chromatography (hexane/EtOAc) to yield the desired lactams.

<u>Method F</u>

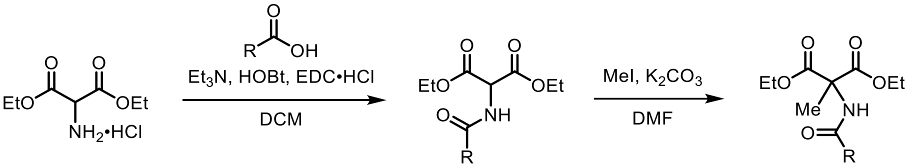

To a stirred solution of diethyl 2-aminomalonate hydrochloride (120 mol%) and corresponding acid (100 mol%) in DCM (0.25 M) were added Et_3N (350 mol%), HOBt (120 mol%), and EDC•HCl (120 mol%). The reaction mixture was stirred at room temperature overnight. The mixture was then diluted with water, extracted with EtOAc, washed with brine, dried over Na_2SO_4, and filtered. The filtrate was concentrated under vacuum and submitted to flash column chromatography (hexane/EtOAc) to yield corresponding acylated aminomalonic esters.

To a stirred solution of the above product in DMF (0.25 M) was added K_2CO_3 (300 mol%) and MeI (150 mol%). The resulting mixture was stirred at room temperature overnight. The reaction mixture was diluted with water, extracted with EtOAc, washed with brine, dried over Na_2SO_4, and filtered. The filtrate was concentrated under vacuum and submitted to flash column chromatography (hexane/EtOAc) to yield the desired disubstituted aminomalonic esters.

<u>Method G</u>

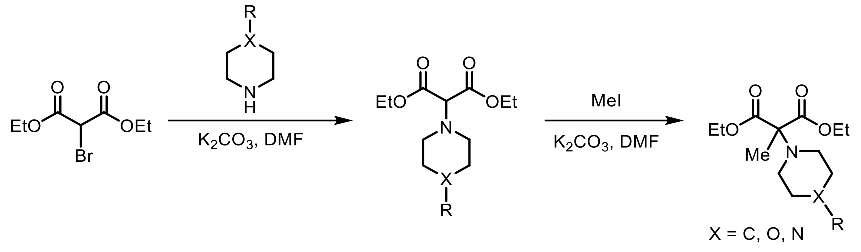

To a stirred solution of corresponding cyclic amine (100 mol%) in DMF (0.25 M) was added K_2CO_3 (200 mol%). After stirring for 10 min, diethyl bromomalonate (110 mol%) was added dropwise and the reaction mixture was stirred at room temperature overnight. The mixture was then diluted with water, extracted with EtOAc, washed with brine, dried over Na_2SO_4, and filtered. The filtrate was concentrated under vacuum and submitted to flash column chromatography (hexane/EtOAc) to yield corresponding malonic esters.

To a stirred solution of the above product in DMF (0.25 M) was added K_2CO_3 (300 mol%) and MeI (200 mol%). The resulting mixture was stirred at room temperature overnight. The reaction mixture was diluted with water, extracted with EtOAc, washed with brine, dried over Na_2SO_4, and filtered. The filtrate was concentrated under vacuum and submitted to flash column chromatography (hexane/EtOAc) to yield the desired tertiary α-amino malonic esters.

Method H

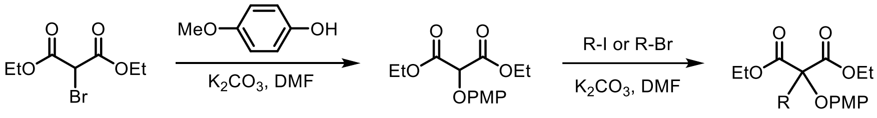

To a stirred solution of *p*-methoxyphenol (100 mol%) in DMF (0.25 M) was added K_2CO_3 (300 mol%). After stirring for 10 min, diethyl bromomalonate (110 mol%) was added dropwise and the reaction mixture was stirred at room temperature overnight. The mixture was then diluted with water, extracted with EtOAc, washed with brine, dried over Na_2SO_4, and filtered. The filtrate was concentrated under vacuum and submitted to flash column chromatography (hexane/EtOAc = 6:1) to yield diethyl 2-(4-methoxyphenoxy) malonate.

To a stirred solution of the above product in DMF (0.25 M) was added K_2CO_3 (300 mol%) and corresponding alkyl bromide or alkyl iodide (200 mol%), which was then stirred at room temperature overnight. The reaction mixture was diluted with water, extracted with EtOAc, washed with brine, dried over Na_2SO_4, and filtered. The filtrate was concentrated under vacuum and submitted to flash column chromatography (hexane/EtOAc) to yield the desired disubstituted oxymalonic esters.

Method I

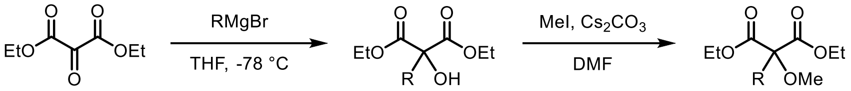

To a solution of diethyl ketomalonate (100 mol%) in dry THF (0.5 M) was added the corresponding freshly prepared Grignard reagent (120 mol%, 0.3 M in THF) dropwise at -78 °C. After stirring at -78 °C for 1 h, the mixture was then quenched with saturated NH_4Cl solution, extracted with EtOAc, washed with brine, dried over Na_2SO_4, and filtered. The filtrate was concentrated under vacuum and submitted to flash column chromatography (hexane/EtOAc) to yield the tertiary α-hydroxyl malonic ester.

To a stirred solution of the above product in DMF (0.25 M) was added Cs_2CO_3 (200 mol%) and MeI (300 mol%). The resulting mixture was stirred at room temperature overnight. The reaction mixture was diluted with water, extracted with EtOAc, washed with brine, dried over Na_2SO_4, and filtered. The filtrate was concentrated under vacuum and submitted to flash column chromatography (hexane/EtOAc) to yield the desired disubstituted oxymalonic esters .

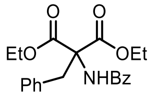

S9

S9 was synthesized using method A (10.0 mmol scale) as colorless oil (2.40 g, 65% yield). R_f = 0.3 (hexane/EtOAc = 4:1).

^{1}H NMR (400 MHz, $CDCl_3$) δ 7.75 (d, J = 7.2 Hz, 2H), 7.53 (m, 1H), 7.44 (dd, J = 8.0, 7.2 Hz, 2H), 7.23-7.21 (m, 4H), 7.03 (m, 2H), 4.30 (m, 4H), 3.78 (s, 2H), 1.31 (t, J = 7.2 Hz, 6H).

^{13}C NMR (100 MHz, $CDCl_3$) δ 167.56, 166.15, 135.18, 133.58, 131.91, 129.94, 128.64, 128.29, 127.19, 127.07, 67.41, 62.70, 37.82, 14.01.

IR (neat, cm^{-1}) 1736, 1665, 1477, 1274, 1198, 1046, 702.

HRMS (ESI) calcd $C_{21}H_{24}NO_5^+$ $[M+H]^+$: 370.1649. Found: 370.1647.

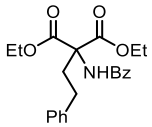

S15

S15 was synthesized using method A (4.0 mmol scale) as colorless oil (0.92 g, 60% yield). R_f = 0.3 (hexane/EtOAc = 4:1).

^{1}H NMR (400 MHz, $CDCl_3$) δ 7.82 (d, J = 7.2 Hz, 2H), 7.56-7.44 (m, 4H), 7.26-7.14 (m, 5H), 4.24 (m,

4H), 2.86-2.82 (m, 2H), 2.58-2.54 (m, 2H), 1.26 (t, *J* = 7.2 Hz, 6H).

^{13}C NMR (100 MHz, $CDCl_3$) δ 168.07, 165.97, 140.41, 133.42, 131.90, 128.57, 128.41, 128.31, 127.07, 126.05, 66.53, 62.60, 33.42, 30.16, 13.95.

IR (neat, cm^{-1}) 1733, 1657, 1478, 1198, 1053, 699.

HRMS (ESI) calcd $C_{18}H_{26}NO_5^+$ $[M+H]^+$: 384.1805. Found: 384.1806.

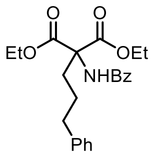

S16

S16 was synthesized using method A (4.0 mmol scale) as colorless oil (0.83 g, 52% yield). R_f = 0.3 (hexane/EtOAc = 4:1).

^{1}H NMR (400 MHz, $CDCl_3$) δ 7.82 (d, *J* = 7.2 Hz, 2H), 7.60 – 7.40 (m, 4H), 7.23 (d, *J* = 7.4 Hz, 2H), 7.16 (d, *J* = 7.1 Hz, 1H), 7.12 (d, *J* = 7.0 Hz, 2H), 4.25 (q, *J* = 7.1 Hz, 4H), 2.62 (t, *J* = 7.8 Hz, 2H), 2.53 (m, 2H), 1.51 (m, 2H), 1.23 (t, *J* = 7.1 Hz, 6H).

^{13}C NMR (100 MHz, $CDCl_3$) δ 168.19, 165.96, 141.73, 133.49, 131.92, 128.63, 128.32, 128.28, 127.10, 125.87, 66.70, 62.58, 35.44, 31.86, 25.74, 13.96.

IR (neat, cm^{-1}) 1725, 1666, 1197, 1019, 1049, 703.

HRMS (ESI) calcd $C_{23}H_{28}NO_5^+$ $[M+H]^+$: 398.1962. Found: 398.1966.

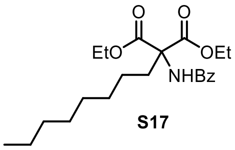

S17

S17 was synthesized using method A (4.0 mmol scale) as colorless oil (1.11 g, 71% yield). R_f = 0.3 (hexane/EtOAc = 4:1).

^{1}H NMR (400 MHz, $CDCl_3$) δ 7.82 (m, 2H), 7.52-7.43 (m, 4H), 4.27 (q, *J* = 7.2 Hz, 4H), 2.45-2.41 (m, 2H), 1.27-1.21 (m, 18H), 0.85-0.82 (m, 3H).

^{13}C NMR (100 MHz, $CDCl_3$) δ 168.31, 165.82, 133.58, 131.81, 128.58, 127.06, 66.80, 62.47, 32.05, 31.71, 29.25, 29.18, 29.06, 23.61, 22.55, 14.00, 13.96.

IR (neat, cm^{-1}) 1733, 1668, 1478, 1202, 1027, 711.

HRMS (ESI) calcd $C_{22}H_{34}NO_5^+$ $[M+H]^+$: 392.2431. Found: 392.2428.

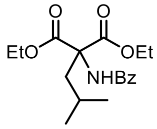

S18

S18 was synthesized using method A (4.0 mmol scale) as colorless oil (0.22 g, 16% yield). R_f = 0.3 (hexane/EtOAc = 4:1).

^{1}H NMR (400 MHz, $CDCl_3$) δ 7.82 (m, 2H), 7.57-7.43 (m, 4H), 4.26 (m, 4H), 2.45 (d, *J* = 6.8 Hz, 2H), 1.60 (m, 1H), 1.25 (t, *J* = 7.2 Hz, 6H), 0.89 (d, *J* = 6.8 Hz, 6H).

^{13}C NMR (100 MHz, $CDCl_3$) δ 168.70, 165.90, 133.67, 131.82, 128.62, 127.04, 66.33, 62.52, 39.96, 24.27, 23.40, 13.94.

IR (neat, cm^{-1}) 1732, 1667, 1478, 1200, 1033, 712.

HRMS (ESI) calcd $C_{18}H_{26}NO_5^+$ $[M+H]^+$: 336.1805. Found: 336.1799.

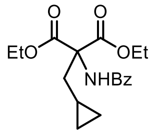

S19

S19 was synthesized using method A (4.0 mmol scale) as colorless oil (0.53 g, 40% yield). R_f = 0.3 (hexane/EtOAc = 4:1).

^{1}H NMR (400 MHz, $CDCl_3$) δ 7.84 (m, 2H), 7.63 (s, 1H), 7.52-7.43 (m, 3H), 4.27 (m, 4H), 2.39 (d, *J* = 6.8 Hz, 2H), 1.26 (t, *J* = 7.2 Hz, 6H), 0.59 (m, 1H), 0.41-0.39 (m, 2H), 0.06 (m, 2H).

^{13}C NMR (100 MHz, $CDCl_3$) δ 168.26, 165.85, 133.62, 131.82, 128.60, 127.06, 66.90, 62.43, 36.74, 13.94, 5.64, 3.70.

IR (neat, cm^{-1}) 1733, 1666, 1478, 1239, 1206, 1015, 711.

HRMS (ESI) calcd $C_{18}H_{24}NO_5^+$ $[M+H]^+$: 334.1649. Found: 334.1647.

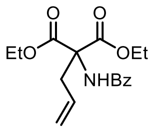

S20

S20 was synthesized using method A (3.6 mmol scale) as a white solid (0.70 g, 61% yield). R_f = 0.3 (hexane/EtOAc = 4:1).

^{1}H NMR (400 MHz, $CDCl_3$) δ 7.82 (d, *J* = 7.2, 2H), 7.55-7.43 (m, 4H), 5.63 (m, 1H), 5.15 – 5.10 (m, 2H), 4.28 (q, *J* = 7.2 Hz, 4H), 3.20 (d, *J* = 7.6, 2H), 1.27 (t, *J* = 7.2 Hz, 6H).

^{13}C NMR (100 MHz, $CDCl_3$) δ 167.72, 165.99, 133.51, 131.90, 131.21, 128.61, 127.07, 120.03, 66.36, 62.65, 36.91, 14.01.

IR (neat, cm^{-1}) 1726, 1657, 1482, 1206, 713, 537.

HRMS (ESI) calcd $C_{17}H_{22}NO_5^+$ $[M+H]^+$: 320.1492. Found: 320.1494.

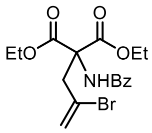

S21

S21 was synthesized using method A (4.0 mmol scale) as colorless oil (1.33 g, 84% yield). R_f = 0.3 (hexane/EtOAc = 4:1).

^{1}H NMR (400 MHz, $CDCl_3$) δ 7.81 (d, *J* = 6.8 Hz, 2H), 7.58 (s, 1H), 7.52 (m, 1H), 7.44 (m, 2H), 5.60 (d, *J* = 0.8 Hz, 1H), 5.51 (d, *J* = 1.2 Hz, 1H), 4.29 (m, 4H), 3.69 (s, 2H), 1.27 (t, *J* = 7.2 Hz, 6H).

^{13}C NMR (100 MHz, $CDCl_3$) δ 167.15, 166.17, 133.33, 131.95, 128.61, 127.05, 126.03, 122.60, 65.45, 62.95, 43.17, 13.86.

IR (neat, cm^{-1}) 1745, 1645, 1484, 1196, 904, 722.

HRMS (ESI) calcd $C_{17}H_{21}NO_5Br^+$ $[M+H]^+$: 398.0598. Found: 398.0596.

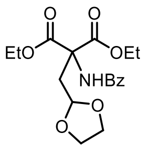

S22

S22 was synthesized using method A (8.0 mmol scale) as yellow oil (0.57 g, 20% yield). R_f = 0.3 (hexane/EtOAc = 4:1).

^{1}H NMR (400 MHz, $CDCl_3$) δ 7.82 (m, 2H), 7.62 (s, 1H), 7.53-7.42 (m, 3H), 4.96 (t, *J* = 4.8 Hz, 1H), 4.27 (m, 4H), 3.88-3.80 (m, 4H), 2.86 (d, *J* = 4.8 Hz, 2H), 1.25 (t, *J* = 7.2 Hz, 6H).

^{13}C NMR (100 MHz, $CDCl_3$) δ 167.67, 165.94, 133.34, 131.71, 128.42, 126.93, 101.32, 64.62, 63.87, 62.47, 35.87, 31.37.

IR (neat, cm^{-1}) 1734, 1652, 1477, 1200, 1029, 722.

HRMS (ESI) calcd $C_{18}H_{24}NO_7^+$ $[M+H]^+$: 366.1547. Found: 366.1546.

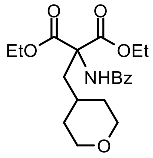

S23

S23 was synthesized using method A (4.0 mmol scale) as a white solid (0.95 g, 63% yield). R_f = 0.3 (hexane/EtOAc = 4:1).

^{1}H NMR (400 MHz, $CDCl_3$) δ 7.81 (m, 2H), 7.57-7.44 (m, 4H), 4.26 (m, 4H), 3.85 (dd, *J* = 11.6, 3.2 Hz, 2H), 3.28 (td, *J* = 11.6, 1.6 Hz, 2H), 2.49 (d, *J* = 6.0 Hz, 2H), 1.51-1.48 (m, 3H), 1.37-1.31 (m, 2H), 1.24 (t, *J* = 7.2 Hz, 6H).

^{13}C NMR (100 MHz, $CDCl_3$) δ 168.56, 166.05, 133.38, 131.97, 128.67, 127.01, 67.67, 65.76, 62.67, 38.39, 33.23, 30.93, 13.90.

IR (neat, cm^{-1}) 1732, 1666, 1478, 1201, 1016, 712.

HRMS (ESI) calcd $C_{20}H_{28}NO_6^+$ $[M+H]^+$: 378.1911. Found: 378.1910.

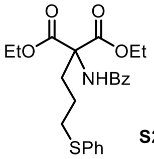

S24

S24 was synthesized using method A (4.0 mmol scale) as colorless oil (0.56 g, 33% yield). R_f = 0.3 (hexane/EtOAc = 4:1).

^{1}H NMR (400 MHz, $CDCl_3$) δ 7.78 (dd, *J* = 7.1, 1.6 Hz, 2H), 7.53 (m, 1H), 7.48 – 7.41 (m, 3H), 7.30 – 7.23 (m, 2H), 7.18 (t, *J* = 7.6 Hz, 2H), 7.11 (m, 1H), 4.24 (m, 4H), 2.90 (t, *J* = 7.3 Hz, 2H), 2.79 – 2.32

(m, 2H), 1.52 (m, 2H), 1.23 (t, J = 7.1 Hz, 6H).

^{13}C NMR (100 MHz, $CDCl_3$) δ 168.01, 166.02, 135.94, 133.39, 131.95, 129.37, 128.81, 128.62, 127.10, 125.96, 66.47, 62.70, 33.24, 31.38, 23.54, 13.96.

IR (neat, cm^{-1}) 1733, 1667, 1477, 1202, 1024, 711.

HRMS (ESI) calcd $C_{23}H_{28}NO_5S^+$ $[M+H]^+$: 430.1683. Found: 430.1680.

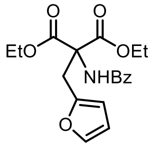

S25

S25 was synthesized using method A (3.0 mmol scale) as yellow oil (0.42 g, 39% yield). R_f = 0.3 (hexane/EtOAc = 4:1).

^{1}H NMR (400 MHz, $CDCl_3$) δ 7.74 (m, 2H), 7.51 (m, 1H), 7.42 (dd, J = 7.6, 7.2 Hz, 2H), 7.35 (s, 1H), 7.26 (m, 1H), 6.23 (dd, J = 2.8, 2.0 Hz, 1H), 6.02 (d, J = 3.2 Hz, 1H), 4.31 (q, J = 7.2 Hz, 4H), 3.85 (s, 2H), 1.29 (t, J = 7.2 Hz, 6H).

^{13}C NMR (100 MHz, $CDCl_3$) δ 167.32, 166.17, 149.69, 142.10, 133.51, 131.84, 127.03, 110.26, 108.79, 65.92, 62.80, 31.32, 13.91.

IR (neat, cm^{-1}) 1737, 1665, 1478, 1199, 1054, 712.

HRMS (ESI) calcd $C_{19}H_{22}NO_6^+$ $[M+H]^+$: 360.1442. Found: 360.1439.

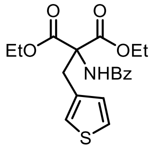

S26

S26 was synthesized using method A (3.0 mmol scale) as yellow oil (0.51 g, 45% yield). R_f = 0.3 (hexane/EtOAc = 4:1).

^{1}H NMR (400 MHz, $CDCl_3$) δ 7.76 (d, J = 7.2 Hz, 2H), 7.53 (m, 1H), 7.45 (dd, J = 8.0, 7.2 Hz, 2H), 7.32 (s, 1H), 7.20 (dd, J = 4.8, 2.8 Hz, 1H), 6.92 (d, J = 1.6 Hz, 1H), 6.77 (dd, J = 4.8, 1.2 Hz, 1H), 4.29 (m, 4H), 3.83 (s, 2H), 1.29 (t, J = 7.2 Hz, 6H).

^{13}C NMR (100 MHz, $CDCl_3$) δ 167.51, 166.07, 135.19, 133.46, 131.96, 128.72, 128.66, 127.04, 125.51,

123.51, 67.07, 62.72, 32.58, 14.00.

IR (neat, cm^{-1}) 1736, 1665, 1448, 1202, 1049, 712.

HRMS (ESI) calcd $C_{19}H_{22}NO_5S^+$ $[M+H]^+$: 376.1213. Found: 376.1213.

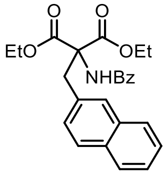

S27

S27 was synthesized using method A (4.0 mmol scale) as white solid (1.26 g, 75% yield). R_f = 0.3 (hexane/EtOAc = 4:1).

^{1}H NMR (400 MHz, $CDCl_3$) δ 7.80-7.64 (m, 5H), 7.55-7.43 (m, 6H), 7.25 (m, 1H), 7.16 (dd, *J* = 8.4, 1.6 Hz, 1H), 4.33 (m, 4H), 3.96 (s, 2H), 1.33 (t, *J* = 7.2 Hz, 6H).

^{13}C NMR (100 MHz, $CDCl_3$) δ 167.54, 166.34, 133.59, 133.25, 132.73, 132.50, 131.95, 128.92, 128.67, 127.97, 127.87, 127.58, 127.48, 127.09, 126.05, 125.73, 67.53, 62.76, 37.89, 14.05.

IR (neat, cm^{-1}) 1732, 1652, 1506, 1198, 1051, 588.

HRMS (ESI) calcd $C_{25}H_{26}NO_5^+$ $[M+H]^+$: 420.1805. Found: 420.1804.

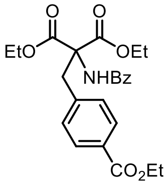

S28

S28 was synthesized using method A (4.0 mmol scale) as colorless oil (1.70 g, 96% yield). R_f = 0.3 (hexane/EtOAc = 4:1).

^{1}H NMR (400 MHz, $CDCl_3$) δ 7.90 (d, *J* = 8.4 Hz, 2H), 7.74 (d, *J* = 7.2 Hz, 2H), 7.54 (dd, *J* = 7.6, 7.2 Hz, 1H), 7.45 (t, *J* = 7.6 Hz, 2H), 7.23 (s, 1H), 7.10 (d, *J* = 8.4 Hz, 2H), 4.32 (m, 6H), 3.83 (s, 2H), 1.36 (t, *J* = 7.2 Hz, 3H), 1.30 (t, *J* = 7.2 Hz, 6H).

^{13}C NMR (100 MHz, $CDCl_3$) δ 167.32, 166.35, 166.25, 140.51, 133.31, 132.08, 129.93, 129.51, 129.47, 128.73, 127.03, 67.19, 62.87, 60.91, 37.74, 14.27, 14.01.

IR (neat, cm^{-1}) 1737, 1666, 1477, 1272, 1048, 707.

HRMS (ESI) calcd $C_{24}H_{28}NO_7^+$ [M+H]$^+$: 442.1860. Found: 442.1858.

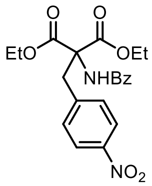

S29

S29 was synthesized using method A (4.0 mmol scale) as yellow solid (1.10 g, 66% yield). R$_f$ = 0.3 (hexane/EtOAc = 3:1).

^{1}H NMR (400 MHz, $CDCl_3$) δ 8.07 (d, *J* = 8.8, 2H), 7.73 (m, 2H), 7.54 (m, 1H), 7.44 (m, 2H), 7.24 (s, 1H), 8.20 (d, *J* = 8.8, 2H), 4.30 (m, 4H), 3.89 (s, 2H), 1.29 (t, *J* = 7.2 Hz, 6H).

^{13}C NMR (100 MHz, $CDCl_3$) δ 167.03, 166.32, 147.20, 143.03, 132.97, 132.23, 130.74, 128.76, 126.92, 123.38, 66.96, 63.02, 37.51, 13.95.

IR (neat, cm^{-1}) 1737, 1659, 1478, 1213, 853, 727.

HRMS (ESI) calcd $C_{21}H_{23}N_2O_7^+$ [M+H]$^+$: 415.1500. Found: 415.1497.

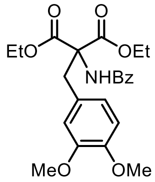

S30

S30 was synthesized using method A (4.0 mmol scale) as white solid (1.52 g, 89% yield). R$_f$ = 0.3 (hexane/EtOAc = 3:1).

^{1}H NMR (400 MHz, $CDCl_3$) δ 7.76 (dd, *J* = 7.2, 1.2 Hz, 2H), 7.52 (m, 1H), 7.43 (m, 2H), 7.24 (s, 1H), 6.72 (d, *J* = 8.4 Hz, 1H), 6.56 (dd, *J* = 8.0, 2.0 Hz, 1H), 6.50 (d, *J* = 2.0 Hz, 1H), 4.30 (m, 4H), 3.82 (s, 3H), 3.71 (s, 2H), 3.55 (s, 3H), 1.31 (t, *J* = 7.2 Hz, 6H).

^{13}C NMR (101 MHz, $CDCl_3$) δ 167.56, 165.97, 148.52, 148.12, 133.42, 132.01, 128.65, 127.53, 127.01, 121.72, 113.15, 110.89, 67.50, 62.63, 55.71, 55.37, 37.30, 14.01.

IR (neat, cm^{-1}) 1737, 1657, 1458, 1256, 1197, 1094, 718.

HRMS (ESI) calcd $C_{23}H_{28}NO_7^+$ [M+H]$^+$: 430.1860. Found: 430.1858.

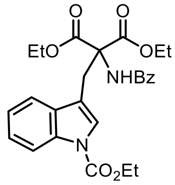

S31

S31 was synthesized using method B (4.2 mmol scale) as yellow solid (1.20 g, 60% yield). R_f = 0.3 (hexane/EtOAc = 3:1).

^{1}H NMR (400 MHz, $CDCl_3$) δ 8.11 (br d, *J* = 7.6 Hz, 1H), 7.71 (dd, *J* = 7.2, 1.2 Hz, 2H), 7.51 (m, 1H), 7.43 – 7.34 (m, 5H), 7.25 (m, 1H), 7.07 (m, 1H), 4.42 (q, *J* = 7.2 Hz, 2H), 4.27 (m, 4H), 3.93 (s, 2H), 1.38 (t, *J* = 7.2 Hz, 3H), 1.29 (t, *J* = 7.2 Hz, 6H).

^{13}C NMR (100 MHz, $CDCl_3$) δ 167.53, 166.54, 150.68, 135.08, 133.56, 131.89, 130.78, 128.55, 127.10, 124.59, 124.29, 122.66, 119.11, 115.05, 114.88, 67.19, 63.05, 62.81, 27.58, 14.27, 13.95.

IR (neat, cm^{-1}) 1737, 1369, 1257, 1129, 1042, 698.

HRMS (ESI) calcd $C_{26}H_{29}N_2O_7^+$ $[M+H]^+$: 481.1969. Found: 481.1969.

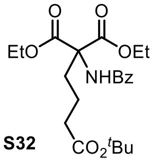

S32

S32 was synthesized using method A (4.0 mmol scale) as colorless oil (0.83 g, 49% yield). R_f = 0.3 (hexane/EtOAc = 4:1).

^{1}H NMR (400 MHz, $CDCl_3$) δ 7.82 (m, 2H), 7.54-7.43 (m, 4H), 4.27 (m, 4H), 2.46 (m, 2H), 2.22 (t, *J* = 7.6 Hz, 2H), 1.48-1.39 (m, 11H), 1.25 (t, *J* = 7.2 Hz, 6H).

^{13}C NMR (100 MHz, $CDCl_3$) δ 172.29, 168.03, 165.93, 133.39, 131.90, 128.58, 127.10, 80.28, 66.53, 62.62, 34.92, 31.57, 28.00, 19.34, 13.94.

IR (neat, cm^{-1}) 1727, 1667, 1478, 1204, 1149, 712.

HRMS (ESI) calcd $C_{22}H_{32}NO_7^+$ $[M+H]^+$: 422.2173. Found: 422.2173.

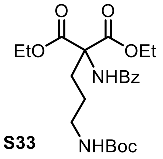

S33

S33 was synthesized using method A (4.0 mmol scale) as white solid (0.52 g, 30% yield). R_f = 0.3 (hexane/EtOAc = 3:1).

^{1}H NMR (400 MHz, $CDCl_3$) δ 7.81 (d, *J* = 7.2, 2H), 7.54-7.43 (m, 4H), 4.59 (br s, 1H), 4.27 (m, 4H), 3.10 (m, 2H), 2.46 (m, 2H), 1.38 (m, 11H), 1.25 (t, *J* = 7.2 Hz, 6H).

^{13}C NMR (100 MHz, $CDCl_3$) δ 168.04, 165.99, 155.73, 133.33, 131.96, 128.62, 127.08, 79.08, 66.43, 62.69, 40.13, 29.67, 28.30, 24.28, 13.96.

IR (neat, cm^{-1}) 1742, 1640, 1520, 1276, 1198, 720.

HRMS (ESI) calcd $C_{22}H_{33}N_2O_7^+$ $[M+H]^+$: 437.2282. Found: 437.2284.

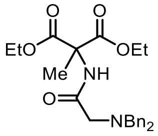

S34

S34 was synthesized using method F (5.0 mmol scale) as colorless oil (0.66 g, 31% yield). R_f = 0.3 (hexane/EtOAc = 3:1).

^{1}H NMR (400 MHz, $CDCl_3$) δ 8.61 (s, 1H), 7.41-7.25 (m, 10H), 4.24 (m, 4H), 3.63 (s, 4H), 3.08 (s, 2H), 1.68 (s, 3H), 1.21 (t, *J* = 7.2 Hz, 6H).

^{13}C NMR (100 MHz, $CDCl_3$) δ 170.34, 168.62, 137.91, 129.06, 128.48, 127.40, 62.50, 62.41, 58.87, 57.41, 20.80, 13.90.

IR (neat, cm^{-1}) 1736, 1682, 1494, 1277, 1113, 699.

HRMS (ESI) calcd $C_{24}H_{31}N_2O_5^+$ $[M+H]^+$: 427.2227. Found: 427.2227.

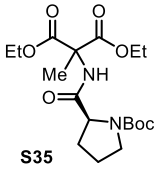

S35

S35 was synthesized using method F (15.0 mmol scale) as colorless oil (2.60 g, 45% yield). R_f = 0.3 (hexane/EtOAc = 3:1).

^{1}H NMR (400 MHz, $CDCl_3$) δ 7.89 (br s, 0.45H), 7.30 (br s, 0.55H), 4.26-4.19 (m, 5H), 3.48-3.45 (m, 2H), 2.23-1.85 (m, 4H), 1.73 (s, 3H), 1.46 (s, 9H), 1.24 (m, 6H).

^{13}C NMR (100 MHz, $CDCl_3$) δ 171.28, 170.91, 168.44, 168.11, 155.15, 154.17, 80.26, 62.28, 60.55, 59.64, 46.71, 30.46, 28.08, 25.40, 24.19, 23.39, 20.68, 13.73.

IR (neat, cm^{-1}) 1739, 1682, 1500, 1366, 1113, 1018, 858.

HRMS (ESI) calcd $C_{18}H_{31}N_2O_7^+$ $[M+H]^+$: 387.2126. Found: 387.2126.

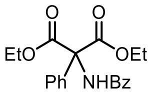

S37

S37 was synthesized using method B (5.0 mmol scale) as white solid (0.75 g, 42% yield). R_f = 0.3 (hexane/EtOAc = 3:1).

^{1}H NMR (400 MHz, $CDCl_3$) δ 7.93-7.90 (m, 3H), 7.68 (m, 2H), 7.58-7.47 (m, 3H), 7.37-7.33 (m, 3H), 4.32 (m, 4H), 1.28 (t, *J* = 7.2 Hz, 6H).

^{13}C NMR (100 MHz, $CDCl_3$) δ 167.20, 165.77, 133.87, 133.19, 132.03, 128.65, 128.43, 128.01, 127.66, 127.17, 68.65, 62.91, 13.84.

IR (neat, cm^{-1}) 1735, 1514, 1263, 1205, 1020, 692.

HRMS (ESI) calcd $C_{20}H_{22}NO_5^+$ $[M+H]^+$: 356.1492. Found: 356.1492.

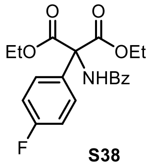

S38

S38 was synthesized using method B (3.0 mmol scale) as yellow solid (0.56 g, 50% yield). R_f = 0.3 (hexane/EtOAc = 3:1).

^{1}H NMR (400 MHz, $CDCl_3$) δ 7.92-7.90 (m, 3H), 7.69-7.65 (m, 2H), 7.57 (m, 1H), 7.51-7.48 (m, 2H), 7.06-7.02 (m, 2H), 7.38-7.25 (m, 4H), 1.27 (t, *J* = 7.2 Hz, 6H).

^{13}C NMR (100 MHz, $CDCl_3$) δ 167.13, 165.85, 162.76 (d, *J* = 247.9 Hz), 133.07, 132.21, 129.80, 129.72, 128.76, 127.24, 114.94 (d, *J* = 21.7 Hz), 68.17, 63.12, 13.90.

^{19}F NMR (376 MHz, $CDCl_3$) δ -113.86.

IR (neat, cm^{-1}) 1736, 1656, 1506, 1233, 729, 522.
HRMS (ESI) calcd $C_{20}H_{21}FNO_5^+$ $[M+H]^+$: 374.1398. Found: 374.1395.

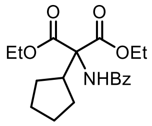

S39

S39 was synthesized using method A (4.0 mmol scale) as colorless oil (0.60 g, 43% yield). R_f = 0.3 (hexane/EtOAc = 4:1).

^{1}H NMR (400 MHz, $CDCl_3$) δ 7.82 (d, *J* = 9.2 Hz, 2H), 7.54-7.40 (m, 4H), 4.27 (q, *J* = 7.2 Hz, 4H), 2.97 (m, 1H), 1.84-1.83 (m, 2H), 1.56-1.54 (m, 4H), 1.43-1.37 (m, 2H), 1.26 (t, *J* = 7.2 Hz, 6H).

^{13}C NMR (100 MHz, $CDCl_3$) δ 168.27, 166.24, 133.82, 131.79, 128.60, 127.06, 67.92, 62.28, 44.74, 27.69, 24.79, 13.99.

IR (neat, cm^{-1}) 1726, 1663, 1484, 1266, 1202, 722.

HRMS (ESI) calcd $C_{19}H_{26}NO_5^+$ $[M+H]^+$: 348.1805. Found: 348.1803.

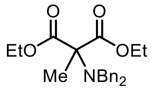

S10

S10[1] was synthesized using method C (2.3 mmol scale) as colorless oil (0.61 g, 72% yield). R_f = 0.3 (hexane/EtOAc = 10:1).

^{1}H NMR (400 MHz, $CDCl_3$) δ 7.32 (d, *J* = 7.2, 4H), 7.19 (dd, *J* = 7.2, 7.6 Hz, 4H), 7.12 (dd, *J* = 7.2, 7.2 Hz, 2H), 4.11 (m, 4H), 3.90 (s, 4H), 1.60 (s, 3H), 1.24 (t, *J* = 7.2 Hz, 6H).

^{13}C NMR (100 MHz, $CDCl_3$) δ 171.16, 140.26, 128.60, 127.81, 126.61, 73.00, 61.27, 55.63, 22.41, 14.04.

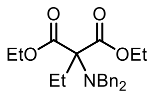

S40

S40 was synthesized using method C (5.0 mmol scale) as colorless oil (1.44 g, 75% yield). R_f = 0.3 (hexane/EtOAc = 10:1).

^{1}H NMR (400 MHz, $CDCl_3$) δ 7.29 (d, *J* = 6.8, 4H), 7.19 (dd, *J* = 6.8, 7.6 Hz, 4H), 7.12 (dd, *J* = 7.2, 7.2

Hz, 2H), 4.14 (m, 4H), 3.84 (s, 4H), 2.15 (q, *J* = 7.2 Hz, 2H), 1.24 (t, *J* = 7.2 Hz, 6H), 0.92 (t, *J* = 7.2 Hz, 3H).

^{13}C NMR (100 MHz, $CDCl_3$) δ 170.32, 139.63, 129.00, 127.76, 126.62, 76.48, 61.00, 55.12, 28.07, 14.13, 8.72.

IR (neat, cm^{-1}) 1725, 1454, 1367, 1233, 1097, 1026, 698.

HRMS (ESI) calcd $C_{23}H_{30}NO_4^+$ $[M+H]^+$: 384.2169. Found: 384.2165.

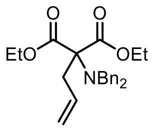

S41

S41 was synthesized using method C (2.0 mmol scale) as colorless oil (655 mg, 83% yield). R_f = 0.3 (hexane/EtOAc = 10:1).

^{1}H NMR (400 MHz, $CDCl_3$) δ 7.32 (d, *J* = 7.2, 4H), 7.21 (dd, *J* = 7.2, 7.2 Hz, 4H), 7.14 (dd, *J* = 7.2, 7.2 Hz, 2H), 5.97 (m, 1H), 5.10-5.06 (m, 2H), 4.13 (m, 4H), 3.90 (s, 4H), 2.90 (d, *J* = 6.8 Hz, 2H), 1.25 (t, *J* = 7.2 Hz, 6H).

^{13}C NMR (100 MHz, $CDCl_3$) δ 169.94, 139.39, 133.25, 128.95, 127.76, 126.66, 117.94, 76.26, 61.11, 55.21, 39.89, 14.07.

IR (neat, cm^{-1}) 1725, 1454, 1228, 1029, 1019, 745, 697.

HRMS (ESI) calcd $C_{24}H_{30}NO_4^+$ $[M+H]^+$: 396.2169. Found: 396.2170.

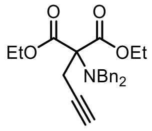

S42

S42 was synthesized using method C (3.0 mmol scale) as colorless oil (1.01 g, 86% yield). R_f = 0.3 (hexane/EtOAc = 10:1).

^{1}H NMR (400 MHz, $CDCl_3$) δ 7.33 (d, *J* = 7.2, 4H), 7.20 (dd, *J* = 7.2, 7.2 Hz, 4H), 7.14 (dd, *J* = 7.2, 7.2 Hz, 2H), 4.12 (m, 4H), 3.96 (s, 4H), 3.02 (d, *J* = 2.8 Hz, 2H), 2.10 (s, 1H), 1.25 (t, *J* = 7.2 Hz, 6H).

^{13}C NMR (100 MHz, $CDCl_3$) δ 169.13, 139.34, 128.82, 127.79, 126.74, 79.43, 75.31, 71.55, 61.55, 55.46, 25.97, 13.97.

IR (neat, cm^{-1}) 1730, 1453, 1371, 1195, 1055, 744, 698.

HRMS (ESI) calcd $C_{24}H_{28}NO_4^+$ $[M+H]^+$: 394.2013. Found: 394.2011.

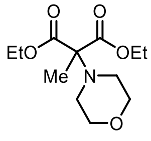

S44

S44 was synthesized using method G (7.7 mmol scale) as colorless oil (1.34 g, 67% yield). R_f = 0.3 (hexane/EtOAc = 5:1).

^{1}H NMR (400 MHz, $CDCl_3$) δ 4.21 (q, J = 7.2, 4H), 3.73-3.71 (m, 4H), 2.67-2.65 (m, 4H), 1.54 (s, 3H), 1.25 (t, J = 7.2 Hz, 6H).

^{13}C NMR (100 MHz, $CDCl_3$) δ 169.50, 71.43, 67.35, 61.29, 48.15, 19.33, 14.08.

IR (neat, cm^{-1}) 2979, 1725, 1448, 1255, 1097, 860.

HRMS (ESI) calcd $C_{12}H_{22}NO_5^+$ $[M+H]^+$: 260.1492. Found: 260.1492.

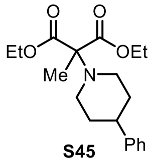

S45

S45 was synthesized using method G (16.0 mmol scale) as colorless oil (1.78 g, 33% yield). R_f = 0.3 (hexane/EtOAc = 4:1).

^{1}H NMR (400 MHz, $CDCl_3$) δ 7.31-7.17 (m, 5H), 4.26 (m, 4H), 3.12 (d, 2H), 2.51 (m, 1H), 2.41 (td, J = 11.2, 2.8 Hz, 2H), 1.91 – 1.84 (m, 4H), 1.61 (s, 3H), 1.30 (t, J = 7.2 Hz, 6H).

^{13}C NMR (100 MHz, $CDCl_3$) δ 170.05, 146.24, 128.34, 126.85, 126.06, 72.17, 61.23, 48.66, 42.98, 33.92, 19.46, 14.14.

IR (neat, cm^{-1}) 1725, 1244, 1097, 1019, 757, 699.

HRMS (ESI) calcd $C_{19}H_{28}NO_4^+$ $[M+H]^+$: 334.2013. Found: 334.2012.

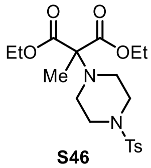

S46

S46 was synthesized using method G (3.2 mmol scale) as colorless oil (0.42 g, 32% yield). R_f = 0.3 (hexane/EtOAc = 3:1).

^{1}H NMR (400 MHz, $CDCl_3$) δ 7.61 (d, *J* = 8.0 Hz, 2H), 7.30 (d, *J* = 8.0 Hz, 2H), 4.20 (m, 4H), 3.04 (s, 4H), 2.74 (t, *J* = 4.8 Hz, 4H), 2.42 (s, 3H), 1.51 (s, 3H), 1.24 (t, *J* = 7.2 Hz, 6H).

^{13}C NMR (100 MHz, $CDCl_3$) δ 169.33, 143.73, 132.11, 129.64, 127.87, 71.17, 61.53, 46.99, 46.56, 21.46, 19.51, 14.08.

IR (neat, cm^{-1}) 1722, 1261, 1166, 1094, 936, 729, 549.

HRMS (ESI) calcd $C_{19}H_{29}N_2O_6S^+$ $[M+H]^+$: 413.1741. Found: 413.1732.

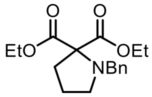

S11

S11 was synthesized using method D (4.0 mmol scale) as colorless oil (0.84 g, 69% yield). R_f = 0.3 (hexane/EtOAc = 10:1).

^{1}H NMR (400 MHz, $CDCl_3$) δ 7.39 (d, *J* = 7.2 Hz, 2H), 7.32-7.21 (m, 3H), 4.26 (q, *J* = 7.2 Hz, 4H), 3.90 (s, 2H), 2.79 (t, *J* = 6.8 Hz, 2H), 2.41 (t, *J* = 7.6 Hz, 2H), 1.85 (m, 2H), 1.29 (t, *J* = 7.2 Hz, 6H).

^{13}C NMR (100 MHz, $CDCl_3$) δ 170.71, 140.02, 128.40, 128.14, 126.77, 74.65, 61.09, 54.90, 51.42, 34.49, 22.15, 14.21.

IR (neat, cm^{-1}) 1725, 1454, 1249, 1103, 1027, 698.

HRMS (ESI) calcd $C_{17}H_{24}NO_4^+$ $[M+H]^+$: 306.1700. Found: 306.1703.

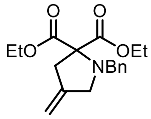

S47

S47 was synthesized using method D (2.3 mmol scale) as colorless oil (0.54 g, 74% yield). R_f = 0.3 (hexane/EtOAc = 12:1).

^{1}H NMR (400 MHz, $CDCl_3$) δ 7.42-7.22 (m, 5H), 4.91 (s, 1H), 4.83 (s, 1H), 4.28 (q, *J* = 7.2 Hz, 4H), 3.92 (s, 2H), 3.41 (s, 2H), 3.07 (s, 2H), 1.30 (t, *J* = 7.2 Hz, 6H).

^{13}C NMR (125 MHz, $CDCl_3$) δ 169.79, 143.61, 139.17, 128.53, 128.27, 127.06, 105.79, 74.69, 61.37, 56.40, 55.19, 41.10, 14.21.

IR (neat, cm^{-1}) 1728, 1237, 1161, 1075, 1042, 741, 699.

HRMS (ESI) calcd $C_{18}H_{24}NO_4^+$ $[M+H]^+$: 318.1700. Found: 318.1700.

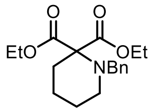

S48

S48 was synthesized using method D (3.0 mmol scale) as colorless oil (0.62 g, 65% yield). R_f = 0.3 (hexane/EtOAc = 10:1).

^{1}H NMR (400 MHz, $CDCl_3$) δ 7.45 (d, *J* = 7.2 Hz, 2H), 7.32-7.20 (m, 3H), 4.25 (q, *J* = 7.2 Hz, 4H), 3.84 (s, 2H), 2.53 (t, *J* = 5.6 Hz, 2H), 2.17 (t, *J* = 6.0 Hz, 2H), 1.58-1.51 (m, 4H), 1.29 (t, *J* = 7.2 Hz, 6H).

^{13}C NMR (100 MHz, $CDCl_3$) δ 171.06, 140.64, 128.25, 127.99, 126.60, 73.78, 61.15, 58.06, 47.05, 33.36, 25.27, 21.15, 14.14.

IR (neat, cm^{-1}) 1726, 1445, 1244, 1116, 1026, 698.

HRMS (ESI) calcd $C_{18}H_{26}NO_4^+$ $[M+H]^+$: 320.1856. Found: 320.1858.

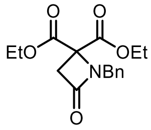

S49

S49 was synthesized using method E (6.9 mmol scale) as colorless oil (1.05 g, 50% yield). R_f = 0.3 (hexane/EtOAc = 4:1).

^{1}H NMR (400 MHz, $CDCl_3$) δ 7.30-7.22 (m, 5H), 4.59 (s, 2H), 3.98 (m, 4H), 3.37 (s, 2H), 1.12 (t, *J* = 7.2 Hz, 6H).

^{13}C NMR (100 MHz, $CDCl_3$) δ 167.28, 165.47, 135.19, 128.41, 128.34, 127.56, 62.50, 62.28, 46.25, 45.64, 13.64.

IR (neat, cm^{-1}) 1737, 1369, 1257, 1129, 1042, 698.

HRMS (ESI) calcd $C_{16}H_{20}NO_5^+$ $[M+H]^+$: 306.1336. Found: 306.1331.

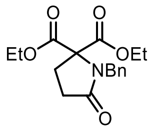

S50

S50 was synthesized using method E (3.8 mmol scale) as colorless oil (0.87 g, 72% yield). R_f = 0.3 (hexane/EtOAc = 3:1).

^{1}H NMR (400 MHz, $CDCl_3$) δ 7.26-7.12 (m, 5H), 4.74 (s, 2H), 3.99-3.85 (m, 4H), 2.57-2.54 (m, 4H), 1.10 (t, *J* = 7.2 Hz, 6H).

^{13}C NMR (100 MHz, $CDCl_3$) δ 175.65, 168.32, 136.99, 128.13, 126.92, 126.90, 71.71, 62.23, 45.67, 28.57, 27.26, 13.62.

IR (neat, cm^{-1}) 1736, 1704, 1371, 1239, 1159, 1018, 704.

HRMS (ESI) calcd $C_{17}H_{22}NO_5^+$ $[M+H]^+$: 320.1492. Found: 320.1494.

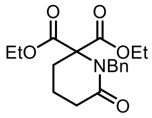

S51

S51 was synthesized using method E (2.7 mmol scale) as white solid (0.75 g, 83% yield). R_f = 0.3 (hexane/EtOAc = 3:1).

^{1}H NMR (400 MHz, $CDCl_3$) δ 7.24-7.08 (m, 5H), 4.77 (s, 2H), 4.00-3.83 (m, 4H), 2.59 (t, *J* = 6.8 Hz, 2H), 2.47-2.44 (m, 2H), 1.79-1.76 (m, 2H), 1.11 (t, *J* = 7.2 Hz, 6H).

^{13}C NMR (100 MHz, $CDCl_3$) δ 170.56, 168.31, 137.52, 127.92, 126.71, 126.54, 72.55, 62.35, 49.11, 32.17, 31.11, 17.94, 13.65.

IR (neat, cm^{-1}) 1727, 1652, 1222, 1179, 1014, 691.

HRMS (ESI) calcd $C_{18}H_{24}NO_5^+$ $[M+H]^+$: 334.1649. Found: 334.1641.

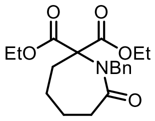

S52

S52 was synthesized using method E (7.9 mmol scale) as white solid (1.92 g, 70% yield). R_f = 0.3 (hexane/EtOAc = 3:1).

^{1}H NMR (400 MHz, $CDCl_3$) δ 7.26-7.13 (m, 5H), 4.65 (s, 2H), 4.04-3.96 (m, 4H), 2.59-2.56 (m, 2H), 2.42-2.39 (m, 2H), 1.81 (m, 2H), 1.72-1.71 (m, 2H), 1.07 (t, J = 7.2 Hz, 6H).

^{13}C NMR (100 MHz, $CDCl_3$) δ 176.37, 168.53, 138.25, 127.83, 126.43, 126.33, 74.00, 62.29, 51.98, 37.53, 35.12, 24.73, 21.64, 13.48.

IR (neat, cm^{-1}) 1720, 1648, 1261, 1181, 1097, 695.

HRMS (ESI) calcd $C_{19}H_{26}NO_5^+$ $[M+H]^+$: 348.1805. Found: 348.1794.

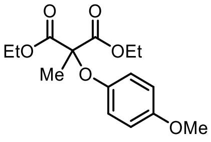

S12

S12 was synthesized using method H (3.0 mmol scale) as colorless oil (0.67 g, 76% yield). R_f = 0.3 (hexane/EtOAc = 5:1).

^{1}H NMR (400 MHz, $CDCl_3$) δ 6.93 (d, J = 9.2 Hz, 2H), 6.75 (d, J = 9.2 Hz, 2H), 4.25 (q, J = 7.2 Hz, 4H), 3.74 (s, 3H), 1.64 (s, 3H), 1.25 (t, J = 7.2 Hz, 6H).

^{13}C NMR (100 MHz, $CDCl_3$) δ 168.87, 155.91, 147.85, 122.24, 114.10, 83.30, 62.05, 55.42, 20.16, 13.89.

IR (neat, cm^{-1}) 1740, 1505, 1209, 1109, 846, 522.

HRMS (ESI) calcd $C_{15}H_{20}NaO_6^+$ $[M+Na]^+$: 319.1152. Found: 319.1152.

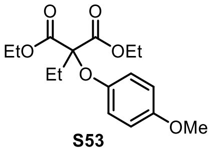

S53

S53 was synthesized using method H (5.5 mmol scale) as colorless oil (1.26 g, 74% yield). R_f = 0.3 (hexane/EtOAc = 5:1).

^{1}**H NMR** (400 MHz, $CDCl_3$) δ 6.90 (d, *J* = 9.2 Hz, 2H), 6.74 (d, *J* = 9.2 Hz, 2H), 4.22 (m, 4H), 3.73 (d, *J* = 0.8 Hz, 3H), 2.21 (q, *J* = 7.2 Hz, 2H), 1.22 (t, *J* = 7.2 Hz, 6H), 0.95 (t, *J* = 7.2 Hz, 3H).

^{13}**C NMR** (100 MHz, $CDCl_3$) δ 168.21, 155.42, 148.37, 120.84, 114.10, 86.45, 61.86, 55.43, 26.58, 13.91, 7.41.

IR (neat, cm^{-1}) 1740, 1505, 1208, 1019, 823.

HRMS (ESI) calcd $C_{16}H_{22}NaO_6^+$ $[M+Na]^+$: 333.1309. Found: 333.1308.

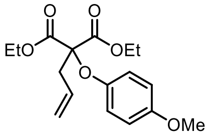

S54

S54 was synthesized using method H (4.0 mmol scale) as colorless oil (0.71 g, 55% yield). R_f = 0.3 (hexane/EtOAc = 5:1).

^{1}**H NMR** (400 MHz, $CDCl_3$) δ 6.93 (d, *J* = 8.8 Hz, 2H), 6.75 (d, *J* = 8.8 Hz, 2H), 5.84 (m, 1H), 5.14-5.09 (m, 2H), 4.22 (m, 4H), 3.73 (s, 3H), 2.93 (d, *J* = 7.2 Hz, 2H), 1.22 (t, *J* = 7.2 Hz, 6H).

^{13}**C NMR** (100 MHz, $CDCl_3$) δ 167.76, 155.66, 148.25, 130.68, 121.31, 119.59, 114.11, 85.84, 61.98, 55.43, 37.97, 13.92.

IR (neat, cm^{-1}) 1739, 1505, 1204, 1033, 845.

HRMS (ESI) calcd $C_{17}H_{22}NaO_6^+$ $[M+Na]^+$: 345.1309. Found: 345.1308.

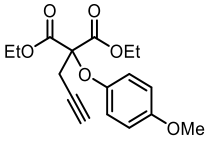

S55

S55 was synthesized using method H (4.0 mmol scale) as colorless oil (0.96 g, 75% yield). R_f = 0.3 (hexane/EtOAc = 5:1).

^{1}**H NMR** (400 MHz, $CDCl_3$) δ 7.03 (d, *J* = 8.8 Hz, 2H), 6.76 (d, *J* = 8.8 Hz, 2H), 4.27 (m, 4H), 3.74 (s, 3H), 2.99 (d, *J* = 1.6 Hz, 2H), 2.13 (t, *J* = 2.4 Hz, 1H), 1.26 (t, *J* = 7.2 Hz, 6H).

^{13}**C NMR** (100 MHz, $CDCl_3$) δ 166.81, 156.47, 147.22, 122.98, 114.19, 85.18, 72.38, 62.38, 55.38, 23.88, 13.89.

IR (neat, cm^{-1}) 1740, 1505, 1201, 1071, 846.

HRMS (ESI) calcd $C_{17}H_{20}NaO_6^+$ $[M+Na]^+$: 343.1152. Found: 343.1150.

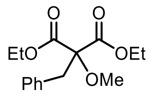

S13

S13 was synthesized using method I (12.0 mmol scale) as colorless oil (1.07 g, 32% yield). R_f = 0.3 (hexane/EtOAc = 10:1).

^{1}H NMR (400 MHz, $CDCl_3$) δ 7.27-7.20 (m, 5H), 4.21 (m, 4H), 3.48 (s, 3H), 3.36 (s, 2H), 1.23 (t, *J* = 7.2 Hz, 6H).

^{13}C NMR (100 MHz, $CDCl_3$) δ 168.16, 134.71, 130.08, 128.08, 126.94, 85.48, 61.70, 53.79, 38.71, 13.98.

IR (neat, cm^{-1}) 1737, 1235, 1084, 1038, 860, 699.

HRMS (ESI) calcd $C_{15}H_{20}NaO_5^+$ $[M+Na]^+$: 303.1203. Found: 303.1211.

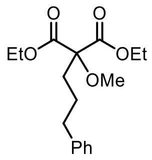

S57

S57 was synthesized using method I (2.3 mmol scale) as colorless oil (0.28 g, 40% yield). R_f = 0.3 (hexane/EtOAc = 10:1).

^{1}H NMR (400 MHz, $CDCl_3$) δ 7.26 (td, *J* = 7.6, 7.2 Hz, 2H), 7.18-7.15 (m, 3H), 4.23 (q, *J* = 7.2 Hz, 4H), 3.30 (s, 3H), 2.64 (t, *J* = 7.6 Hz, 2H), 2.08 (m, 2H), 1.65-1.61 (m, 2H), 1.25 (t, *J* = 7.2 Hz, 6H).

^{13}C NMR (100 MHz, $CDCl_3$) δ 168.51, 141.54, 128.33, 128.25, 125.81, 84.68, 61.66, 53.12, 35.43, 31.67, 24.35, 14.02.

IR (neat, cm^{-1}) 1736, 1453, 1212, 1164, 699.

HRMS (ESI) calcd $C_{17}H_{24}NaO_5^+$ $[M+Na]^+$: 331.1516. Found: 331.1516.

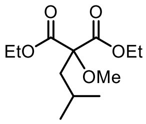

S58

S58 was synthesized using method I (6.4 mmol scale) as colorless oil (1.02 g, 65% yield). R_f = 0.3 (hexane/EtOAc = 10:1).

^{1}H NMR (400 MHz, $CDCl_3$) δ 4.24 (q, *J* = 7.2 Hz, 4H), 3.33 (s, 3H), 2.00 (d, *J* = 6.4 Hz, 2H), 1.71 (m, 1H), 1.27 (t, *J* = 7.2 Hz, 6H), 0.91 (d, *J* = 6.8 Hz, 6H).

^{13}C NMR (100 MHz, $CDCl_3$) δ 168.90, 84.74, 61.62, 53.22, 40.09, 23.65, 23.47, 14.03.

IR (neat, cm^{-1}) 2959, 1737, 1465, 1238, 1028, 860.

HRMS (ESI) calcd $C_{12}H_{22}NaO_5^+$ $[M+Na]^+$: 269.1359. Found: 269.1357.

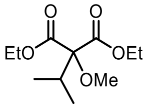

S59

S59 was synthesized using method I (15.0 mmol scale) as colorless oil (1.21 g, 35% yield). R_f = 0.3 (hexane/EtOAc = 10:1).

^{1}H NMR (400 MHz, $CDCl_3$) δ 4.25 (m, 4H), 3.43 (s, 3H), 2.41 (m, 1H), 1.29 (m, 6H), 0.97 (dt, *J* = 6.8, 1.6 Hz, 6H).

^{13}C NMR (100 MHz, $CDCl_3$) δ 168.30, 88.20, 61.24, 54.78, 34.06, 17.21, 14.11.

IR (neat, cm^{-1}) 2981, 1734, 1252, 1093, 860.

HRMS (ESI) calcd $C_{11}H_{20}NaO_5^+$ $[M+Na]^+$: 255.1203. Found: 255.1205.

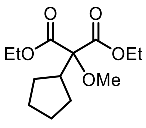

S60

S60 was synthesized using method I (12.0 mmol scale) as colorless oil (1.26 g, 41% yield). R_f = 0.3 (hexane/EtOAc = 10:1).

^{1}H NMR (400 MHz, $CDCl_3$) δ 4.24 (q, *J* = 7.2 Hz, 4H), 3.47 (s, 3H), 2.60 (m, 1H), 1.70-1.48 (m, 8H), 1.28 (t, *J* = 7.2 Hz, 6H).

^{13}C NMR (100 MHz, $CDCl_3$) δ 168.87, 86.80, 61.29, 54.96, 44.71, 27.16, 25.75, 14.09.

IR (neat, cm^{-1}) 2953, 1736, 1447, 1250, 1033, 860.

HRMS (ESI) calcd $C_{13}H_{22}NaO_5^+$ $[M+Na]^+$: 281.1359. Found: 281.1361.

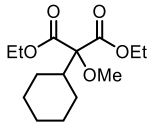

S61

S61 was synthesized using method I (10.0 mmol scale) as colorless oil (0.85 g, 31% yield). R_f = 0.3 (hexane/EtOAc = 10:1).

^{1}H NMR (400 MHz, $CDCl_3$) δ 4.25 (q, *J* = 7.2 Hz, 4H), 3.41 (s, 3H), 2.08 (t, *J* = 12.0 Hz, 1H), 1.74-1.62 (m, 5H), 1.28 (t, *J* = 7.2 Hz, 6H), 1.24-1.07 (m, 5H).

^{13}C NMR (100 MHz, $CDCl_3$) δ 168.25, 88.10, 61.25, 54.81, 44.26, 27.32, 26.39, 26.28, 14.12.

IR (neat, cm^{-1}) 2932, 1734, 1447, 1212, 1031, 862.

HRMS (ESI) calcd $C_{14}H_{24}NaO_5^+$ $[M+Na]^+$: 295.1516. Found: 295.1517.

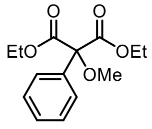

S62

S62 was synthesized using method I (14.0 mmol scale) as colorless oil (2.2 g, 59% yield). R_f = 0.3 (hexane/EtOAc = 5:1).

^{1}H NMR (400 MHz, $CDCl_3$) δ 7.56 (dd, *J* = 8.0, 1.6 Hz, 2H), 7.37-7.34 (m, 3H), 4.27 (m, 4H), 3.41 (s, 3H), 1.26 (t, *J* = 7.2 Hz, 6H).

^{13}C NMR (100 MHz, $CDCl_3$) δ 167.76, 134.68, 128.61, 128.04, 127.47, 86.19, 62.02, 54.27, 13.93.

IR (neat, cm^{-1}) 1737, 1448, 1242, 1028, 696.

HRMS (ESI) calcd $C_{14}H_{18}NaO_5^+$ $[M+Na]^+$: 289.1046. Found: 289.1046.

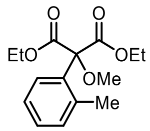

S63

S63 was synthesized using method I (12.0 mmol scale) as colorless oil (2.24 g, 67% yield). R_f = 0.3 (hexane/EtOAc = 6:1).

^{1}H NMR (400 MHz, $CDCl_3$) δ 7.29-7.17 (m, 4H), 4.31 (m, 4H), 3.35 (s, 3H), 2.30 (s, 3H), 1.29 (t, *J* = 7.2 Hz, 6H).

^{13}C NMR (100 MHz, $CDCl_3$) δ 168.46, 137.90, 132.97, 131.83, 128.86, 128.85, 125.57, 87.66, 62.09, 54.12, 19.94, 13.92.

IR (neat, cm^{-1}) 1737, 1446, 1239, 1103, 1026, 750.

HRMS (ESI) calcd $C_{15}H_{20}NaO_5^+$ $[M+Na]^+$: 303.1203. Found: 303.1202.

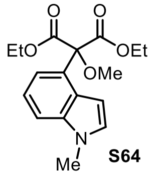

S64

S64 was synthesized using method I (12.0 mmol scale) as yellow oil (2.00 g, 52% yield). R_f = 0.3 (hexane/EtOAc = 5:1).

^{1}H NMR (400 MHz, $CDCl_3$) δ 7.34 (d, *J* = 8.0 Hz, 1H), 7.26-7.19 (m, 2H), 7.05 (d, *J* = 3.2 Hz, 1H), 6.60 (dd, *J* = 3.2, 0.8 Hz, 1H), 4.28 (m, 4H), 3.77 (s, 3H), 3.38 (s, 3H), 1.25 (t, *J* = 7.2 Hz, 6H).

^{13}C NMR (100 MHz, $CDCl_3$) δ 168.06, 136.98, 129.05, 126.66, 126.36, 120.80, 119.51, 110.28, 101.13, 87.00, 61.82, 54.09, 32.81, 13.94.

IR (neat, cm^{-1}) 1733, 1442, 1237, 1104, 1047, 750.

HRMS (ESI) calcd $C_{17}H_{21}NNaO_5^+$ $[M+Na]^+$: 342.1312. Found: 342.1314.

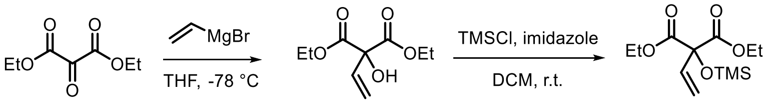

S66

To a solution of diethyl ketomalonate (1.74 g, 10.0 mmol) in dry THF (0.5 M) was added vinylmagnesium

bromide (11.0 ml, 11.0 mmol, 1.0 M in THF) dropwise at -78 °C. After stirring at -78 °C for 1 h, The mixture was then quenched with saturated NH_4Cl solution, extracted with EtOAc, washed with brine, dried over Na_2SO_4, and filtered. The filtrate was concentrated under vacuum and submitted to flash column chromatography (hexane/EtOAc = 5:1) to yield the malonic ester as colorless oil (1.62 g, 80% yield).

To a stirred solution of the above product (1.62 g, 8.0 mmol) in DCM (0.25 M) were added TMSCl (1.5 ml, 12 mmol) and imidazole (1.09 g, 16.0 mmol). The resulting mixture was stirred at room temperature for 4 h. The reaction mixture was diluted with water, extracted with EtOAc, washed with brine, dried over Na_2SO_4, and filtered. The filtrate was concentrated under vacuum and submitted to flash column chromatography (hexane/EtOAc = 20:1) to yield **S66** (1.50 g, 68% yield) as colorless oil.

^{1}H NMR (400 MHz, $CDCl_3$) δ 6.28 (dd, *J* = 16.8, 10.4 Hz, 1H), 5.55 (dd, *J* = 16.8, 0.8 Hz, 1H), 5.31 (dd, *J* = 10.4, 0.8 Hz, 1H), 4.22 (q, *J* = 7.2 Hz, 4H), 1.28 (t, *J* = 7.2 Hz, 6H), 0.19 (s, 9H).

^{13}C NMR (100 MHz, $CDCl_3$) δ 169.14, 135.03, 116.61, 81.80, 62.03, 13.94, 1.68.

IR (neat, cm^{-1}) 1744, 1196, 1048, 843, 756.

HRMS (ESI) calcd $C_{12}H_{22}NaO_5Si^+$ $[M+Na]^+$: 297.1129. Found: 297.1130.

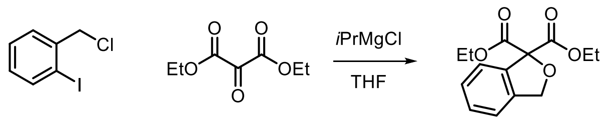

S14

S14[3] was synthesized from *o*-iodobenzyl chloride and diethyl ketomalonate following a literature procedure (0.61 g, 56% Yield) as colorless oil. R_f = 0.3 (hexane/EtOAc = 5:1).

^{1}H NMR (400 MHz, $CDCl_3$) δ 7.63 (d, *J* = 7.2 Hz, 1H), 7.39-7.32 (m, 2H), 7.24 (d, *J* = 7.2 Hz, 1H), 5.31 (s, 2H), 4.26 (m, 4H), 1.28 (m, 6H).

^{13}C NMR (100 MHz, $CDCl_3$) δ 167.83, 139.28, 135.08, 129.36, 127.69, 124.44, 120.72, 90.68, 74.36, 62.17, 13.91.

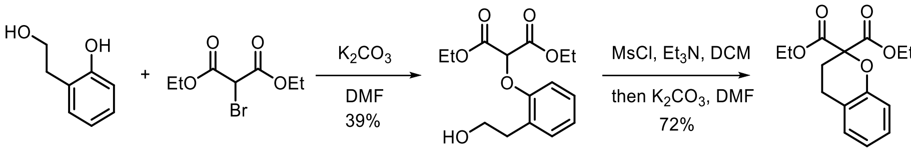

S67

To a stirred solution of *o*-(2-hydroxyethyl)phenol[3] (2.59 g, 18.8 mmol) in DMF (60 mL) was added K_2CO_3 (5.19 g, 37.6 mmol). After stirring for 10 min, diethyl bromomalonate (6.71 g, 28.2 mmol) was added dropwise and the reaction mixture was stirred at room temperature overnight. The mixture was then diluted

with water, extracted with EtOAc, washed with brine, dried over Na_2SO_4, and filtered. The filtrate was concentrated under vacuum and submitted to flash column chromatography (hexane/EtOAc = 3:1) to yield the intermediate malonate (2.17g, 39% yield).

To a stirred solution of the above product (2.17g, 7.3 mmol) in DCM (30 mL) was added methanesulfonyl chloride (1.1 mL, 14.6 mmol) and Et_3N (3.0 mL, 21.9 mmol), which was then stirred at room temperature overnight. The reaction mixture was diluted with water, extracted with EtOAc, washed with brine, dried over Na_2SO_4, and filtered. The filtrate was concentrated under vacuum and the resulting crude product was used in the next step without purification.

To a stirred solution of the above product in DMF (30 mL) was added K_2CO_3 (5.19 g, 37.6 mmol). The reaction mixture was stirred at room temperature overnight. The mixture was then diluted with water, extracted with EtOAc, washed with brine, dried over Na_2SO_4, and filtered. The filtrate was concentrated under vacuum and submitted to flash column chromatography (hexane/EtOAc = 5:1) to yield **S67** as colorless oil (1.47 g, 72% yield).

^{1}H NMR (400 MHz, $CDCl_3$) δ 7.13 (m, 1H), 7.03-7.00 (m, 2H), 6.88 (t, *J* = 7.2 Hz, 1H), 4.27 (q, *J* = 7.2 Hz, 4H), 2.78 (t, *J* = 6.8 Hz, 2H), 2.46 (t, *J* = 6.8 Hz, 2H), 1.27 (t, *J* = 7.2 Hz, 6H).

^{13}C NMR (100 MHz, $CDCl_3$) δ 167.75, 152.59, 129.16, 127.64, 121.26, 120.59, 117.05, 81.39, 62.32, 26.38, 21.57, 13.94.

IR (neat, cm^{-1}) 2983, 1743, 1211, 1058, 752.

HRMS (ESI) calcd $C_{15}H_{18}NaO_5^+$ $[M+Na]^+$: 301.1046. Found: 301.1047.

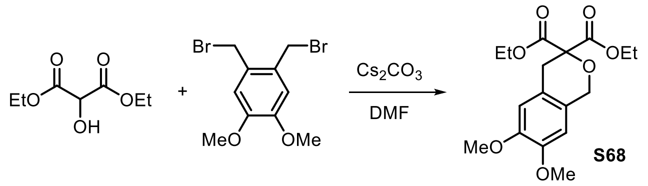

S68

To a stirred solution of diethyl 2-hydroxymalonate (1.00 g, 5.7 mmol) and 1,2-di(bromomethyl)-4,5-dimethoxybenzene (2.19 g, 6.8 mmol) in DMF (17 mL) was added Cs_2CO_3 (6.09 g, 17.1 mmol). The reaction mixture was stirred at room temperature overnight. The mixture was then diluted with water, extracted with EtOAc, washed with brine, dried over Na_2SO_4, and filtered. The filtrate was concentrated under vacuum and submitted to flash column chromatography (hexane/EtOAc = 4:1) to yield **S68** (0.82 g, 43% Yield) as white solid.

^{1}H NMR (400 MHz, $CDCl_3$) δ 6.62 (s, 1H), 6.45 (s, 1H), 4.91 (s, 2H), 4.23 (q, *J* = 7.2 Hz, 4H), 3.83 (s, 3H), 3.80 (s, 3H), 3.25 (s, 2H), 1.23 (t, *J* = 7.2 Hz, 6H).

^{13}C NMR (100 MHz, $CDCl_3$) δ 168.08, 147.99, 147.96, 124.18, 121.99, 111.01, 106.71, 80.14, 65.38, 62.11, 55.80, 55.77, 32.16, 13.94.

IR (neat, cm^{-1}) 1741, 1518, 1208, 1069, 863.

HRMS (ESI) calcd $C_{17}H_{22}NaO_7^+$ $[M+Na]^+$: 361.1258. Found: 361.1258.

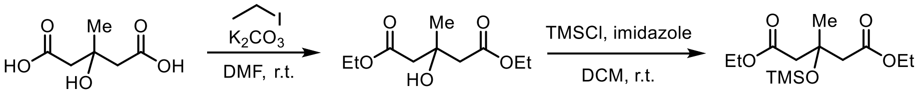

71

To a solution of dicrotalic acid (0.50 g, 3.1 mmol) in DMF (40 mL) was added K_2CO_3 (2.76 g, 20 mmol) and ethyl iodide (3.22 g, 20 mmol). After stirring at room temperature for overnight, the mixture was then diluted with water, extracted with EtOAc, washed with brine, dried over Na_2SO_4, and filtered. The filtrate was concentrated under vacuum to yield the malonic ester as colorless oil, which was directly used for next step.

To a stirred solution of the above malonic ester (218 mg, 1.0 mmol) in DCM (5 mL) were added TMSCl (164 mg, 1.5 mmol) and imidazole (136 mg, 2.0 mmol). The resulting mixture was stirred at room temperature for 4 h. The reaction mixture was diluted with water, extracted with DCM, washed with brine, dried over Na_2SO_4, and filtered. The filtrate was concentrated under vacuum and submitted to flash column chromatography (hexane/EtOAc = 20:1) to yield **71** (0.25 g, 86% yield) as colorless oil. R_f = 0.4 (hexane/EtOAc = 20:1)

^{1}H NMR (500 MHz, $CDCl_3$) δ 4.12 (q, *J* = 7.1 Hz, 4H), 2.69 (m, 4H), 1.48 (s, 3H), 1.25 (t, *J* = 7.1 Hz, 6H), 0.12 (s, 9H).

^{13}C NMR (125 MHz, $CDCl_3$) δ 170.80, 73.30, 60.14, 46.61, 28.20, 14.22, 2.29.

IR (neat, cm^{-1}) 2981, 1732, 1250, 1097, 839, 753.

HRMS (ESI) calcd $C_{13}H_{27}O_5Si^+$ $[M+H]^+$: 291.1622. Found: 291.1624.

1.4 Procedures for the Reductive Desymmetrization of Amino/oxymalonic esters and Characterization of Chiral Products

Procedure A

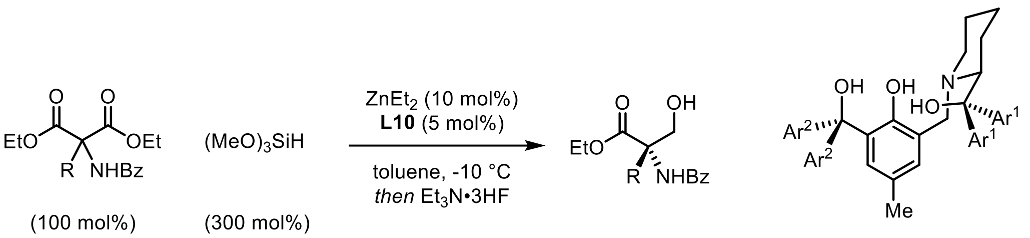

L10 Ar^1 = 3,5-di*t*Bu (Ph)
Ar^2 = 7-*N*-Me-indolyl

To an oven-dried 10 mL round bottom flask with a stir bar was added **L10** (45.0 mg, 0.05 mmol), then the flask was sealed with a rubber septum and evacuated/refilled with nitrogen for three times. Freshly distilled toluene (1 mL) was added to the flask via syringe and the mixture was stirred at room temperature for 5 min. Diethylzinc (0.10 mL, 1.0 M solution in hexane, 0.10 mmol) was added to the flask via syringe slowly. The resulting catalyst solution was stirred at room temperature for 30 min before use.

To a separate oven-dried 5 mL Schlenk tube with a stir bar was added aminomalonic ester (0.30 mmol, 100 mol%), sealed with a rubber septum, and evacuated/refilled with nitrogen for three times. Trimethoxysilane (110 mg, 0.90 mmol, 300 mol%) and freshly distilled toluene (2.7 mL) were added via syringe. The mixture was stirred at room temperature while 0.3 mL of aforementioned catalyst solution was added via syringe. The reaction mixture was stirred at -10 °C for 36-96 h, monitored by TLC. After the starting material was consumed, 0.2 mL triethylamine trihydrofluoride was added dropwise to quench the reaction. The mixture was diluted with 5 mL diethyl ether, and stirred for 30 min. Subsequently, the reaction mixture was filtered through a short pad of silica gel, eluted with diethyl ether slowly (as the remaining triethylamine trihydrofluoride reacts with silica gel to release heat). The filtrate was evaporated and purified by flash column chromatography (hexane/EtOAc) to yield the desymmetrization product.

Procedure B

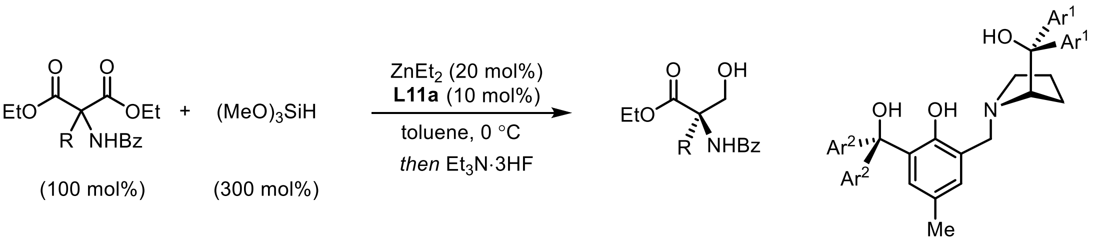

L11a: Ar^1 = 3,5-di*t*Bu (Ph)
Ar^2 = 7-*N*-Me-indolyl

To an oven-dried 10 mL round bottom flask with a stir bar was added **L11a** (44.0 mg, 0.05 mmol), then the flask was sealed with a rubber septum and evacuated/refilled with nitrogen for three times. Freshly

distilled toluene (1 mL) was added to the flask via syringe and the mixture was stirred at room temperature for 5 min. Diethylzinc (0.10 mL, 1.0 M solution in hexane, 0.10 mmol) was added to the flask via syringe slowly. The resulting catalyst solution was stirred at room temperature for 30 min before use.

To a separate oven-dried 5 mL Schlenk tube with a stir bar was added aminomalonic ester (0.30 mmol, 100 mol%), sealed with a rubber septum, and evacuated/refilled with nitrogen for three times. Trimethoxysilane (110 mg, 0.90 mmol, 300 mol%) and freshly distilled toluene (0.9 mL) were added via syringe. The mixture was stirred at room temperature while 0.6 mL of aforementioned catalyst solution was added via syringe. The reaction mixture was stirred at 0 °C for 7-8 d, monitored by TLC. After the starting material was consumed, 0.2 mL triethylamine trihydrofluoride was added dropwise to quench the reaction. The mixture was diluted with 5 mL diethyl ether, and stirred for 30 min. Subsequently, the reaction mixture was filtered through a short pad of silica gel, eluted with diethyl ether slowly (as the remaining triethylamine trihydrofluoride reacts with silica gel to release heat). The filtrate was evaporated and purified by flash column chromatography (hexane/EtOAc) to yield the desymmetrization product.

Procedure C

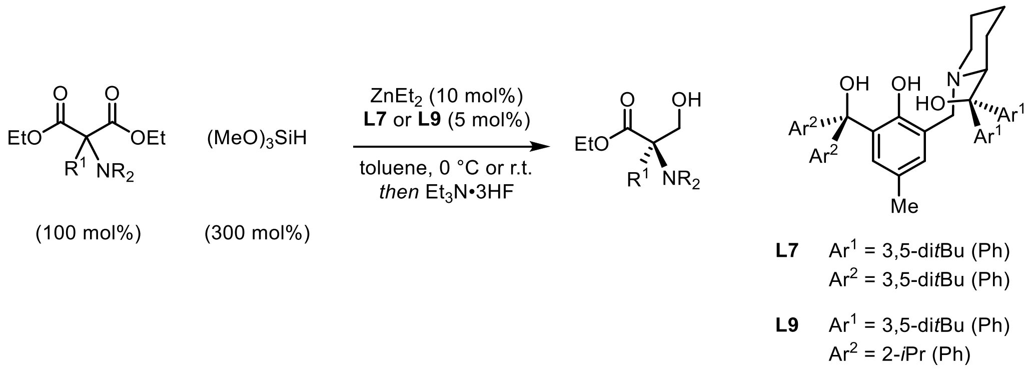

To an oven-dried 10 mL round bottom flask with a stir bar was added **L7** (51.0 mg, 0.05 mmol) or **L9** (44.0 mg, 0.05 mmol), then the flask was sealed with a rubber septum and evacuated/refilled with nitrogen for three times. Freshly distilled toluene (1 mL) was added to the flask via syringe and the mixture was stirred at room temperature for 5 min. Diethylzinc (0.10 mL, 1.0 M solution in hexane, 0.10 mmol) was added to the flask via syringe slowly. The resulting catalyst solution was stirred at room temperature for 30 min before use.

To a separate oven-dried 5 mL Schlenk tube with a stir bar was added aminomalonic ester (0.30 mmol, 100 mol%), sealed with a rubber septum, and evacuated/refilled with nitrogen for three times. Trimethoxysilane (110 mg, 0.90 mmol, 300 mol%) and freshly distilled toluene (2.7 mL) were added via syringe. The mixture was stirred at room temperature while 0.3 mL of aforementioned catalyst solution was added via syringe. The reaction mixture was stirred at 0 °C or room temperature for 18-72 h, monitored by TLC. After the starting material was consumed, 0.4 mL triethylamine and 0.2 mL triethylamine trihydrofluoride was added dropwise to quench the reaction. The mixture was diluted with 5 mL diethyl ether, and stirred for 30 min. Subsequently, the reaction mixture was filtered through a short pad of silica gel, eluted with diethyl ether slowly (as the remaining triethylamine trihydrofluoride reacts

with silica gel to release heat). The filtrate was evaporated and purified by flash column chromatography (hexane/EtOAc) to yield the desymmetrization product.

Procedure D

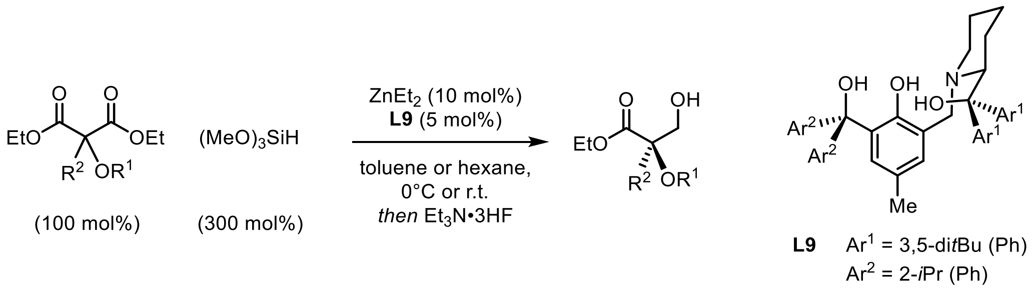

To an oven-dried 10 mL round bottom flask with a stir bar was added **L9** (44.0 mg, 0.05 mmol), then the flask was sealed with a rubber septum and evacuated/refilled with nitrogen for three times. Freshly distilled toluene (1 mL) was added to the flask via syringe and the mixture was stirred at room temperature for 5 min. Diethylzinc (0.10 mL, 1.0 M solution in hexane, 0.10 mmol) was added to the flask via syringe slowly. The resulting catalyst solution was stirred at room temperature for 30 min before use.

To a separate oven-dried 5 mL Schlenk tube with a stir bar was added oxymalonic esters (0.30 mmol, 100 mol%), sealed with a rubber septum, and evacuated/refilled with nitrogen for three times. Trimethoxysilane (110 mg, 0.90 mmol, 300 mol%) and freshly distilled toluene (2.7 mL) were added via syringe. The mixture was stirred at room temperature while 0.3 mL of aforementioned catalyst solution was added via syringe. The reaction mixture was stirred at 0 °C or room temperature for 18-72 h, monitored by TLC. After the starting material was consumed, 0.4 mL triethylamine and 0.2 mL triethylamine trihydrofluoride was added dropwise to quench the reaction. The mixture was diluted with 5 mL diethyl ether, and stirred for 30 min. Subsequently, the reaction mixture was filtered through a short pad of silica gel, eluted with diethyl ether slowly (as the remaining triethylamine trihydrofluoride reacts with silica gel to release heat). The filtrate was evaporated and purified by flash column chromatography (hexane/EtOAc) to yield the desymmetrization product.

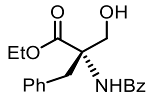

(–)-**9**

(–)-**9** was obtained as colorless oil (80.3 mg, 82% Yield) from the reductive desymmetrization of malonic ester **S9** (110.7 mg, 0.3 mmol) with **L10** in toluene at -10 °C for 36 h using the general procedure A. R_f = 0.2 (hexane/EtOAc = 3:1). $[\alpha]_D^{25}$ = –75.1 (*c* = 1.0, $CHCl_3$). The absolute configuration of (–)-**9** was determined by comparison with a know *N*-Boc-protected amino acid via hydrolysis and Boc protection. ($[\alpha]_D^{22}$ = –38.9 (*c* = 1.0, $CHCl_3$, *S* configuration) $[\alpha]_D^{25}$ lit. = –46 (*c* = 1.0, $CHCl_3$, *S* configuration).).[4]

^{1}H NMR (400 MHz, $CDCl_3$) δ 7.66 (d, *J* = 7.2 Hz, 2H), 7.50 (m, 1H), 7.40 (t, *J* = 7.6 Hz, 2H), 7.24-7.22 (m, 3H), 7.08-7.07 (m, 3H), 4.50 (d, *J* = 10.4 Hz, 1H), 4.29 (m, 2H), 4.05-4.02 (m, 2H), 3.67 (d, *J* = 13.6

Hz, 1H), 3.20 (d, J = 13.6 Hz, 1H), 1.36 (t, J = 7.2 Hz, 3H).

^{13}C NMR (100 MHz, $CDCl_3$) δ 171.98, 167.79, 135.26, 134.24, 131.83, 129.74, 128.62, 128.44, 127.20, 126.95, 67.86, 65.98, 62.48, 37.09, 14.08.

IR (neat, cm^{-1}) 3405, 1732, 1644, 1519, 1224, 701.

HRMS (ESI) calcd $C_{19}H_{22}NO_4^+$ $[M+H]^+$: 328.1543. Found: 328.1541.

HPLC analysis (Chiralpak IF-3, hexane/^{i}PrOH = 90/10, 1.0 mL/min, 230 nm; t_r (major) = 12.87 min, t_r (minor) = 14.77 min) gave the isomeric composition of the product: 91% *e.e.*.

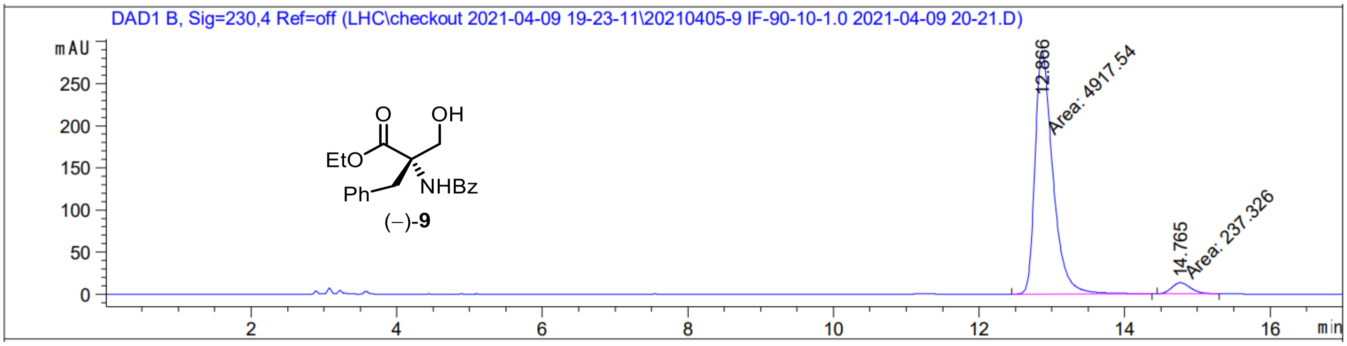

Signal 2: DAD1 B, Sig=230,4 Ref=off

Peak #	RetTime [min]	Type	Width [min]	Area [mAU*s]	Height [mAU]	Area %
1	12.866	MM	0.2837	4917.53711	288.89731	95.3961
2	14.765	MM	0.2992	237.32613	13.22001	4.6039
Totals :				5154.86324	302.11732	

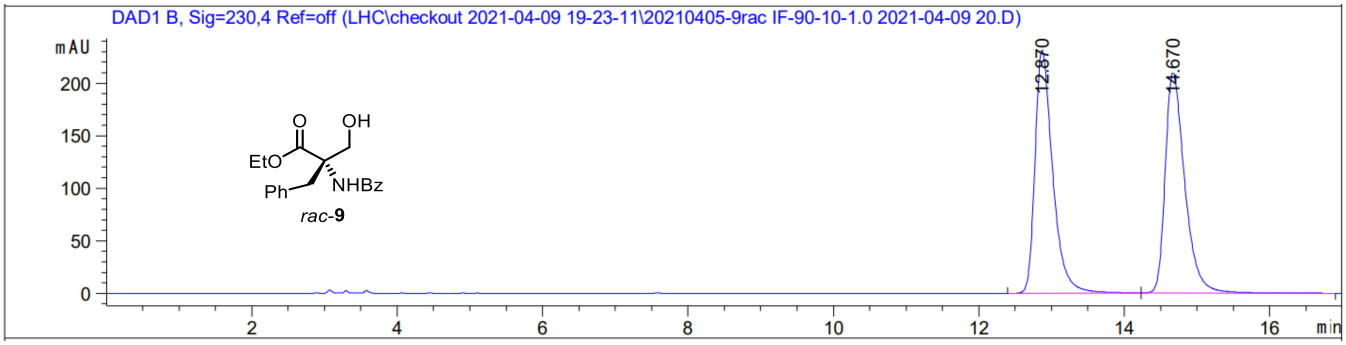

Signal 2: DAD1 B, Sig=230,4 Ref=off

Peak #	RetTime [min]	Type	Width [min]	Area [mAU*s]	Height [mAU]	Area %
1	12.870	BB	0.2555	3916.59985	231.35139	50.0538
2	14.670	BBA	0.2834	3908.18555	209.68886	49.9462
Totals :				7824.78540	441.04025	

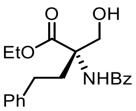

(–)-**15**

(–)-**15** was obtained as colorless oil (83.5 mg, 82% Yield) from the reductive desymmetrization of malonic ester **S15** (114.9 mg, 0.3 mmol) with **L10** in toluene at -10 °C for 48 h using the general procedure A. R_f = 0.2 (hexane/EtOAc = 3:1). $[\alpha]_D^{25}$ = –13.6 (c = 1.0, $CHCl_3$).

^{1}H NMR (400 MHz, $CDCl_3$) δ 7.76 (d, J = 7.6 Hz, 2H), 7.53 (m, 1H), 7.46-7.40 (m, 3H), 7.26-7.22 (m, 2H), 7.17-7.13 (m, 3H), 4.43 (d, J = 11.6 Hz, 1H), 4.26 (m, 2H), 3.93 (d, J = 11.2 Hz, 1H), 3.58 (br s, 1H), 2.83 (m, 1H), 2.66 (m, 1H), 2.47 (m, 1H), 2.17 (m, 1H), 1.34 (t, J = 7.2 Hz, 3H).

^{13}C NMR (100 MHz, $CDCl_3$) δ 172.77, 167.18, 140.58, 134.00, 131.88, 128.62, 128.43, 128.39, 126.97, 126.13, 66.97, 66.01, 62.51, 32.72, 30.31, 14.14.

IR (neat, cm^{-1}) 3405, 1728, 1645, 1519, 1227, 698.

HRMS (ESI) calcd $C_{20}H_{24}NO_4^+$ $[M+H]^+$: 342.1700. Found: 342.1700.

HPLC analysis (Chiralpak IF-3, hexane/^{i}PrOH = 90/10, 1.0 mL/min, 205 nm; t_r (major) = 12.46 min, t_r (minor) = 16.07 min) gave the isomeric composition of the product: 88% *e.e.*.

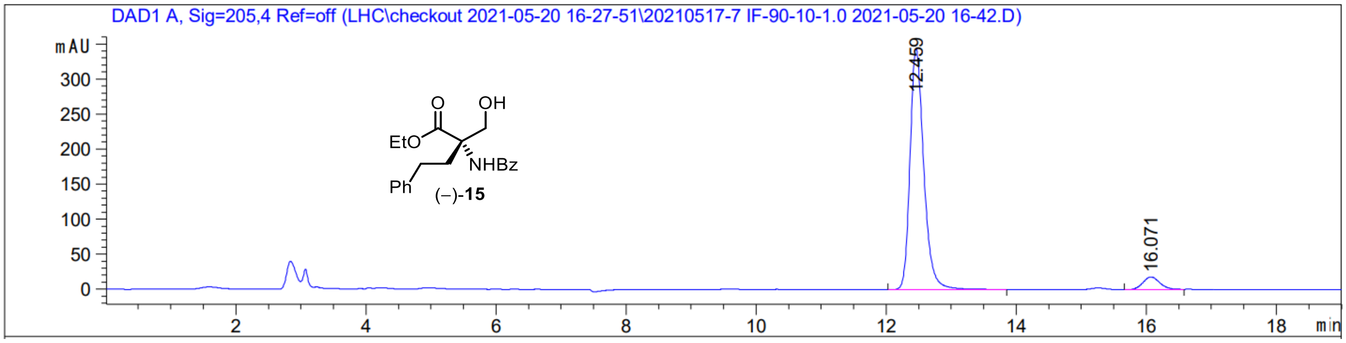

Signal 1: DAD1 A, Sig=205,4 Ref=off

Peak #	RetTime [min]	Type	Width [min]	Area [mAU*s]	Height [mAU]	Area %
1	12.459	BB	0.2255	5058.67090	344.09467	94.0227
2	16.071	BB	0.2749	321.59210	17.95704	5.9773

Totals :				5380.26300	362.05171	

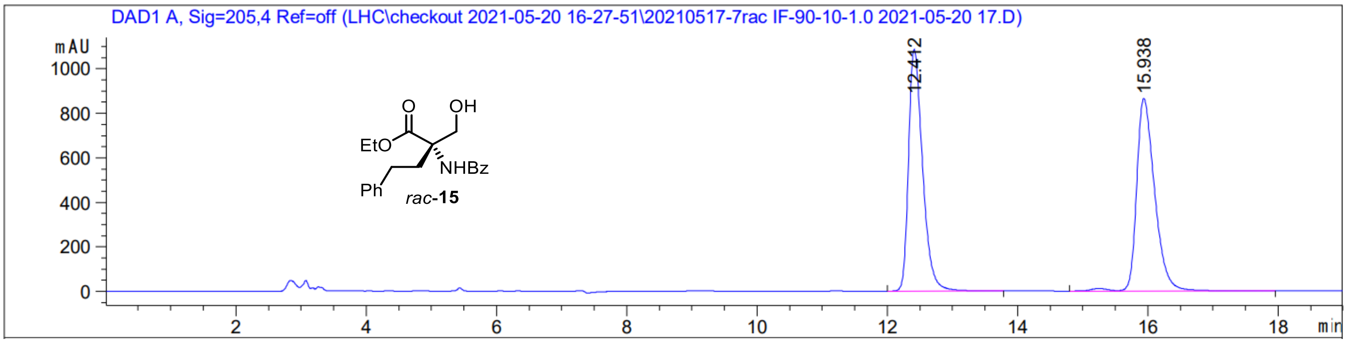

Signal 1: DAD1 A, Sig=205,4 Ref=off

Peak #	RetTime [min]	Type	Width [min]	Area [mAU*s]	Height [mAU]	Area %
1	12.412	BB	0.2309	1.62799e4	1085.92041	49.3460
2	15.938	VB R	0.2927	1.67114e4	864.24005	50.6540

Totals :				3.29913e4	1950.16046	

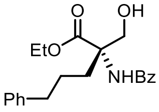

(–)-**16**

(–)-**16** was obtained as colorless oil (80.6 mg, 76% Yield) from the reductive desymmetrization of malonic ester **S16** (119.1 mg, 0.3 mmol) with **L10** in toluene at -10 °C for 96 h using the general procedure A. R_f = 0.2 (hexane/EtOAc = 3:1). $[\alpha]_D^{25}$ = –19.2 (c = 1.0, $CHCl_3$).

^{1}H NMR (400 MHz, $CDCl_3$) δ 7.80 (d, J = 7.2 Hz, 2H), 7.54 (m, 1H), 7.45 (m, 2H), 7.38 (s, 1H), 7.27-7.11 (m, 5H), 4.37 (d, J = 11.2 Hz, 1H), 4.27 (m, 2H), 3.91 (d, J = 11.6 Hz, 1H), 3.58 (br s, 1H), 2.69-2.47 (m, 3H), 1.69-1.66 (m, 1H), 1.44-1.41 (m, 1H), 1.27 (t, J = 7.2 Hz, 3H).

^{13}C NMR (100 MHz, $CDCl_3$) δ 172.85, 167.23, 141.56, 134.08, 131.86, 128.64, 128.31, 128.26, 126.97, 125.89, 67.11, 65.86, 62.43, 35.44, 30.80, 25.62, 14.05.

IR (neat, cm^{-1}) 3405, 2933, 1728, 1645, 1520, 1224, 697.

HRMS (ESI) calcd $C_{21}H_{26}NO_4^+$ $[M+H]^+$: 356.1856. Found: 356.1853.

HPLC analysis (Chiralpak IF-3, hexane/^{i}PrOH = 90/10, 1.0 mL/min, 230 nm; t_r (major) = 11.73 min, t_r (minor) = 14.99 min) gave the isomeric composition of the product: 90% *e.e.*.

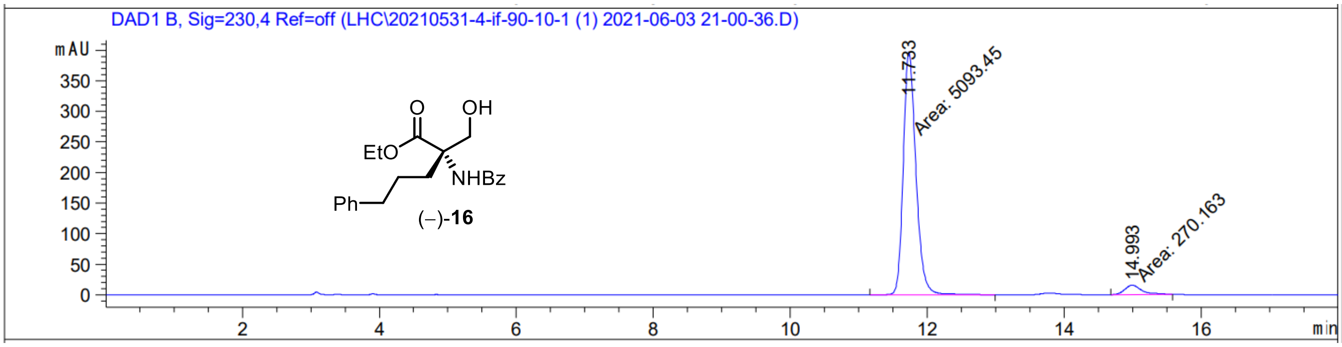

Signal 2: DAD1 B, Sig=230,4 Ref=off

Peak #	RetTime [min]	Type	Width [min]	Area [mAU*s]	Height [mAU]	Area %
1	11.733	MM	0.2138	5093.44629	397.06296	94.9630
2	14.993	MM	0.2889	270.16266	15.58380	5.0370

Totals : 5363.60895 412.64675

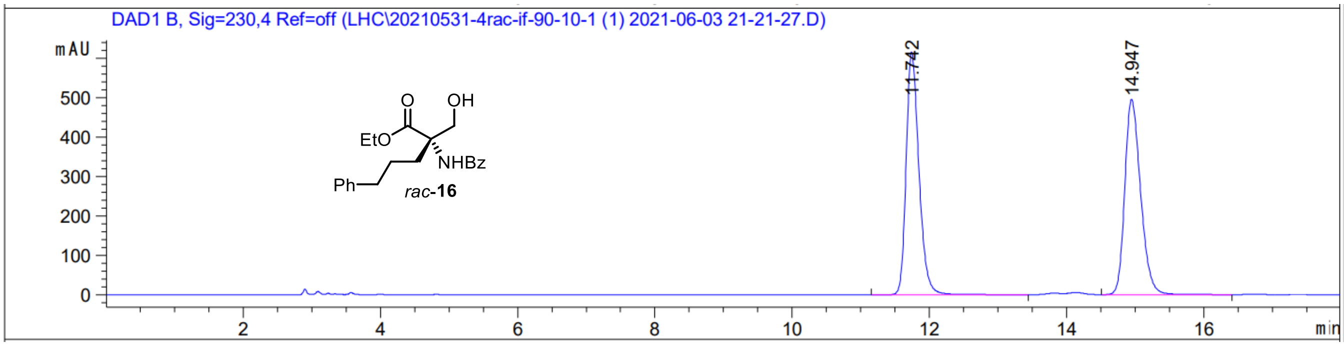

Signal 2: DAD1 B, Sig=230,4 Ref=off

Peak #	RetTime [min]	Type	Width [min]	Area [mAU*s]	Height [mAU]	Area %
1	11.742	BV R	0.1967	7957.54346	615.06427	50.0426
2	14.947	VB	0.2470	7944.00781	495.51120	49.9574

Totals : 1.59016e4 1110.57547

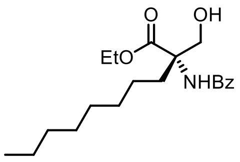

(–)-**17**

(–)-**17** was obtained as colorless oil (79.5 mg, 76% Yield) from the reductive desymmetrization of malonic ester **S17** (117.3 mg, 0.3 mmol) with **L10** in toluene at -10 °C for 48 h using the general procedure A. R_f = 0.2 (hexane/EtOAc = 4:1). $[\alpha]_D^{25}$ = –13.0 (*c* = 1.0, $CHCl_3$).

^{1}H NMR (400 MHz, $CDCl_3$) δ 7.80 (d, *J* = 7.6 Hz, 2H), 7.52 (m, 1H), 7.44 (m, 2H), 7.37 (s, 1H), 4.38 (d, *J* = 11.2 Hz, 1H), 4.30 (m, 2H), 3.92 (d, *J* = 11.6 Hz, 1H), 3.67 (br s, 1H), 2.40 (m, 1H), 1.80 (m, 1H), 1.33 (t, *J* = 7.2 Hz, 3H), 1.25-1.21 (m, 12H), 0.84 (m, 3H).

^{13}C NMR (100 MHz, $CDCl_3$) δ 173.04, 167.23, 134.24, 131.82, 128.64, 126.99, 67.28, 66.06, 62.38, 31.73, 31.28, 29.33, 29.22, 29.07, 23.70, 22.58, 14.12, 14.02.

IR (neat, cm^{-1}) 3400, 2925, 1730, 1649, 1520, 1227, 711.

HRMS (ESI) calcd $C_{20}H_{32}NO_4^+$ $[M+H]^+$: 350.2326. Found: 350.2324.

HPLC analysis (Chiralpak IF-3, hexane/^{i}PrOH = 90/10, 1.0 mL/min, 230 nm; t_r (major) = 8.60 min, t_r (minor) = 10.39 min) gave the isomeric composition of the product: 92% *e.e.*.

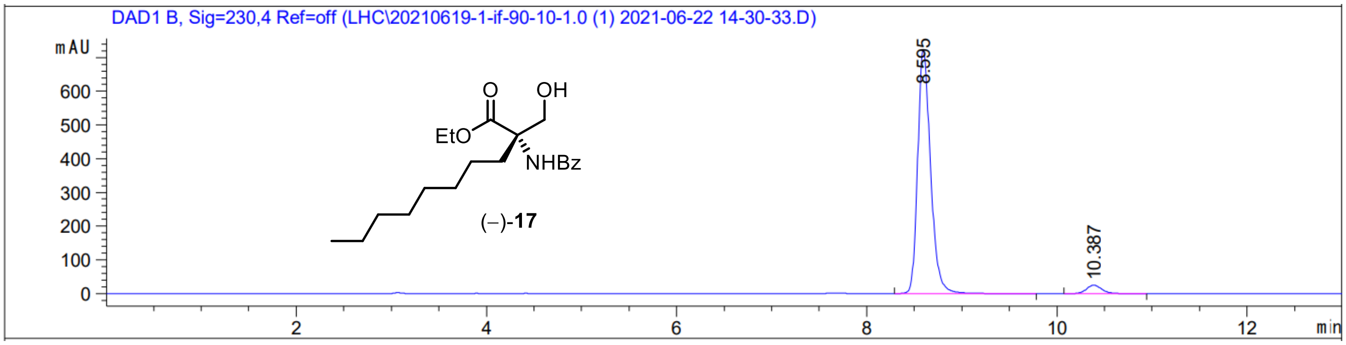

Signal 2: DAD1 B, Sig=230,4 Ref=off

Peak #	RetTime [min]	Type	Width [min]	Area [mAU*s]	Height [mAU]	Area %
1	8.595	BB	0.1443	6821.10645	721.65973	96.0362
2	10.387	BB	0.1728	281.53085	25.11744	3.9638

Totals : 7102.63730 746.77716

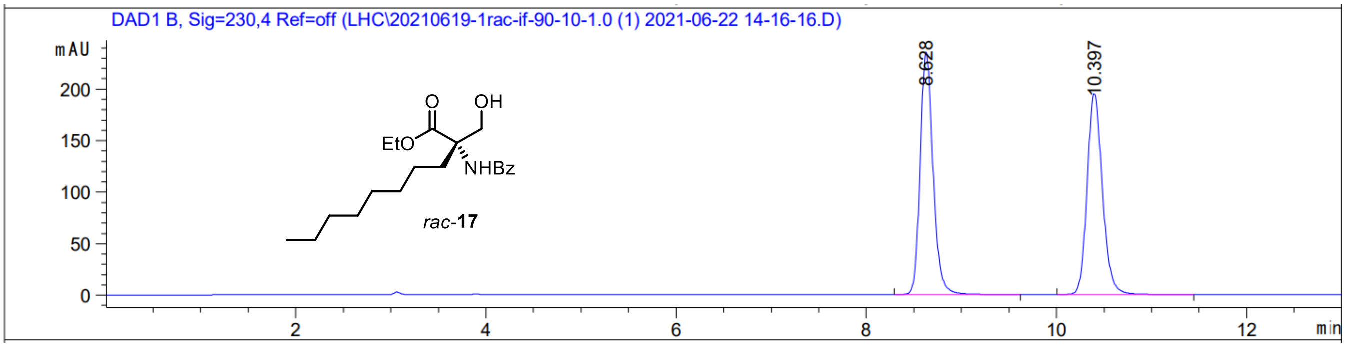

Signal 2: DAD1 B, Sig=230,4 Ref=off

Peak #	RetTime [min]	Type	Width [min]	Area [mAU*s]	Height [mAU]	Area %
1	8.628	BB	0.1439	2218.93701	235.48634	50.3246
2	10.397	BB	0.1730	2190.31152	195.02808	49.6754
Totals :				4409.24854	430.51442	

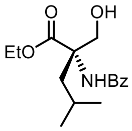

(–)-**18**

(–)-**18** was obtained as colorless oil (73.6 mg, 84% Yield) from the reductive desymmetrization of malonic ester **S18** (100.5 mg, 0.3 mmol) with **L10** in toluene at -10 °C for 36 h using the general procedure A. R_f = 0.2 (hexane/EtOAc = 3:1). $[\alpha]_D^{25}$ = –16.1 (*c* = 1.0, $CHCl_3$).

^{1}H NMR (400 MHz, $CDCl_3$) δ 7.80 (d, *J* = 7.2 Hz, 2H), 7.51-7.42 (m, 4H), 4.40 (d, *J* = 11.2 Hz, 1H), 4.30 (q, *J* = 7.2 Hz, 2H), 3.85 (d, *J* = 11.2 Hz, 1H), 3.45 (br s, 1H), 2.47 (dd, *J* = 14.0, 5.6 Hz, 1H), 1.71 (dd, *J* = 14.0, 7.6 Hz, 1H), 1.62 (m, 1H), 1.34 (t, *J* = 7.2 Hz, 3H), 0.90 (d, *J* = 6.8 Hz, 3H), 0.84 (d, *J* = 6.8 Hz, 3H).

^{13}C NMR (100 MHz, $CDCl_3$) δ 173.67, 167.09, 134.30, 131.79, 128.66, 126.95, 66.73, 62.41, 39.10, 24.52, 23.80, 22.78, 14.05.

IR (neat, cm^{-1}) 3405, 2933, 1728, 1650, 1519, 1226, 712.

HRMS (ESI) calcd $C_{16}H_{24}NO_4^+$ $[M+H]^+$: 294.1700. Found: 294.1700.

HPLC analysis (Chiralpak IF-3, hexane/^{i}PrOH = 90/10, 1.0 mL/min, 205 nm; t_r (major) = 8.56 min, t_r (minor) = 10.65 min) gave the isomeric composition of the product: 94% *e.e.*.

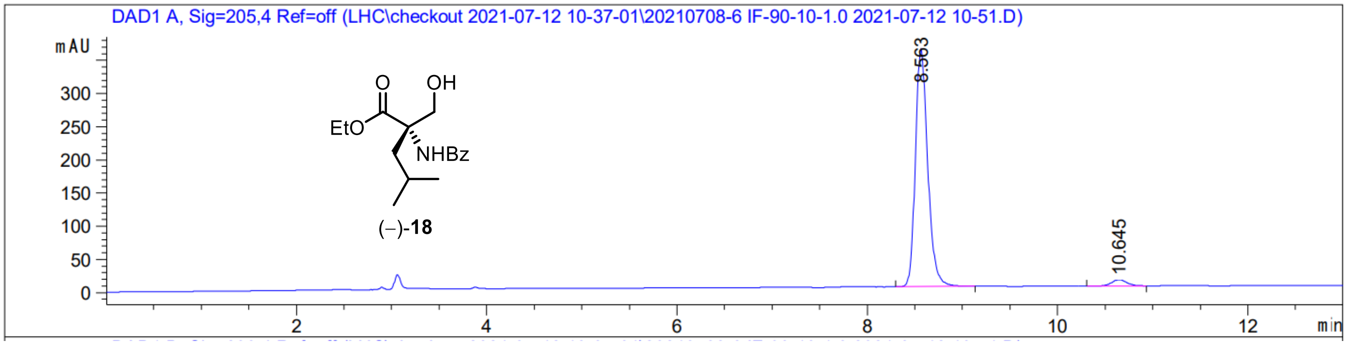

Signal 1: DAD1 A, Sig=205,4 Ref=off

Peak #	RetTime [min]	Type	Width [min]	Area [mAU*s]	Height [mAU]	Area %
1	8.563	BB	0.1391	3228.74512	358.24582	96.8148
2	10.645	BB	0.1721	106.22578	9.52251	3.1852
Totals :				3334.97089	367.76833	

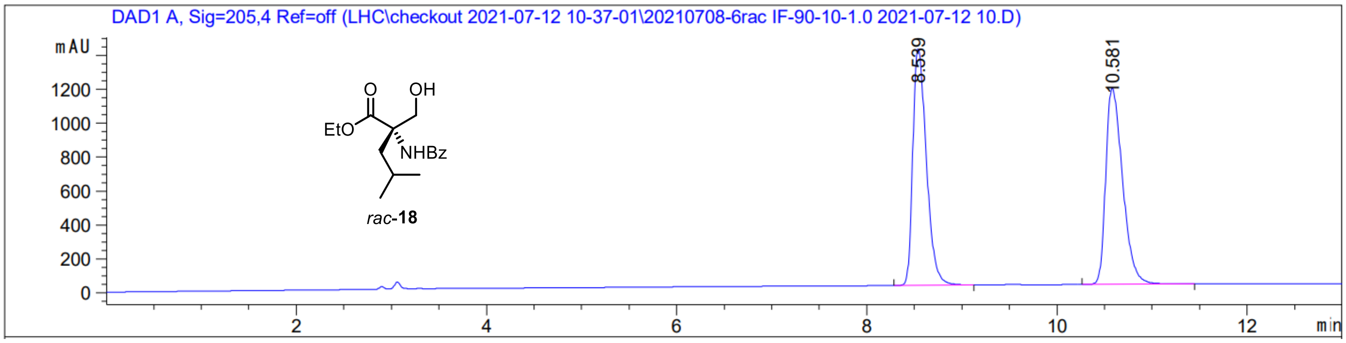

Signal 1: DAD1 A, Sig=205,4 Ref=off

Peak #	RetTime [min]	Type	Width [min]	Area [mAU*s]	Height [mAU]	Area %
1	8.539	BB	0.1562	1.38795e4	1393.71277	49.3059
2	10.581	BB	0.1897	1.42703e4	1158.53638	50.6941
Totals :				2.81499e4	2552.24915	

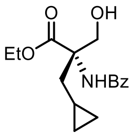

(–)-**19**

(–)-**19** was obtained as colorless oil (64.6 mg, 74% Yield) from the reductive desymmetrization of malonic

ester **S19** (99.9 mg, 0.3 mmol) with **L10** in toluene at -10 °C for 48 h using the general procedure A. R_f = 0.2 (hexane/EtOAc = 3:1). $[\alpha]_D^{25}$ = –17.3 (*c* = 1.0, $CHCl_3$).

¹H NMR (400 MHz, $CDCl_3$) δ 7.81 (m, 2H), 7.52-7.42 (m, 4H), 4.31 (m, 3H), 3.93-3.90 (m, 2H), 2.31 (dd, *J* = 14.4, 6.8 Hz, 1H), 1.76 (dd, *J* = 14.4, 6.8 Hz, 1H), 1.33 (t, *J* = 7.2 Hz, 3H), 0.59 (m, 1H), 0.44-0.42 (m, 2H), 0.10-0.05 (m, 2H).

¹³C NMR (100 MHz, $CDCl_3$) δ 172.88, 167.49, 134.27, 131.80, 128.64, 126.98, 67.37, 65.94, 62.30, 36.17, 14.06, 5.75, 4.00, 3.59.

IR (neat, cm^{-1}) 3405, 2933, 1728, 1645, 1520, 1229, 714.

HRMS (ESI) calcd $C_{16}H_{22}NO_4^+$ $[M+H]^+$: 292.1543. Found: 292.1547.

HPLC analysis (Chiralpak IF-3, hexane/^{i}PrOH = 90/10, 1.0 mL/min, 205 nm; t_r (major) = 13.06 min, t_r (minor) = 14.37 min) gave the isomeric composition of the product: 92% *e.e.*.

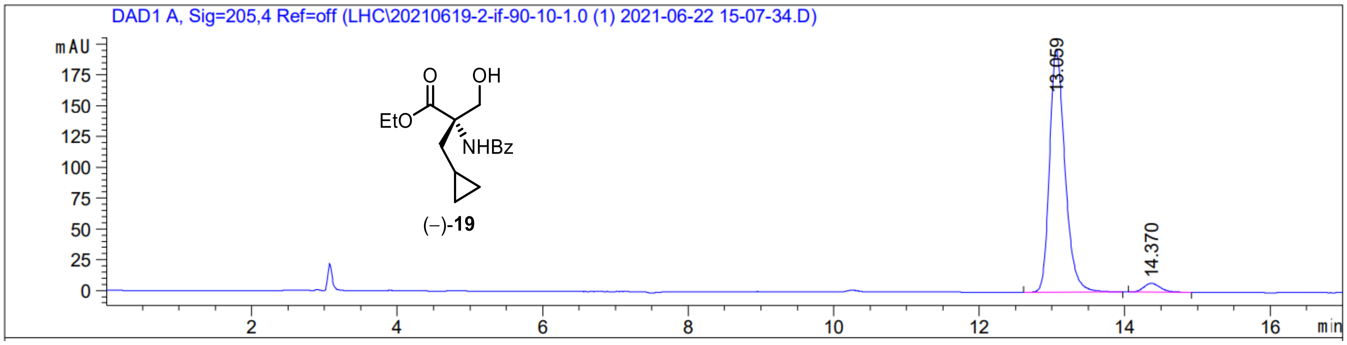

Signal 1: DAD1 A, Sig=205,4 Ref=off

Peak #	RetTime [min]	Type	Width [min]	Area [mAU*s]	Height [mAU]	Area %
1	13.059	BB	0.2242	2913.41650	197.36920	96.1633
2	14.370	BB	0.2474	116.23983	7.23688	3.8367

Totals : 3029.65633 204.60608

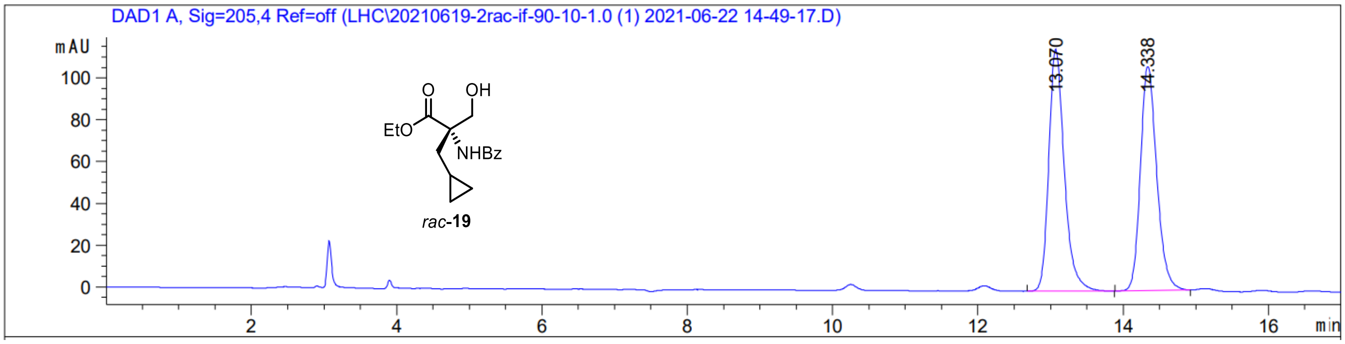

Signal 1: DAD1 A, Sig=205,4 Ref=off

Peak #	RetTime [min]	Type	Width [min]	Area [mAU*s]	Height [mAU]	Area %
1	13.070	BB	0.2270	1716.49243	115.73311	50.6635
2	14.338	BB	0.2378	1671.53149	107.26960	49.3365
Totals :				3388.02393	223.00271	

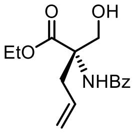

(–)-**20**

(–)-**20** was obtained as colorless oil (70.4 mg, 85% Yield) from the reductive desymmetrization of malonic ester **S20** (95.7 mg, 0.3 mmol) with **L10** in toluene at -10 °C for 48 h using the general procedure A. R_f = 0.2 (hexane/EtOAc = 3:1). $[\alpha]_D^{25}$ = –13.6 (*c* = 1.0, $CHCl_3$).

^{1}H NMR (400 MHz, $CDCl_3$) δ 7.77 (d, *J* = 7.2 Hz, 2H), 7.51 (m, 1H), 7.43 (m, 2H), 7.22 (s, 1H), 5.66 (m, 1H), 5.18-5.13 (m, 2H), 4.34-4.27 (m, 3H), 4.00 (br s, 1H), 3.93 (d, *J* = 11.2 Hz, 1H), 3.09 (dd, *J* = 14.0, 7.6 Hz, 1H), 2.62 (dd, *J* = 14.0, 6.8 Hz, 1H), 1.31 (t, *J* = 7.2 Hz, 3H).

^{13}C NMR (100 MHz, $CDCl_3$) δ 172.16, 167.57, 134.12, 131.86, 131.43, 128.63, 126.97, 120.11, 66.22, 65.92, 62.40, 36.40, 14.13.

IR (neat, cm^{-1}) 3400, 2928, 1732, 1645, 1518, 1227, 712.

HRMS (ESI) calcd $C_{15}H_{20}NO_4^+$ $[M+H]^+$: 278.1387. Found: 278.1387.

HPLC analysis (Chiralpak ID-3, hexane/^{i}PrOH = 90/10, 1.0 mL/min, 230 nm; t_r (major) = 13.05 min, t_r (minor) = 16.26 min) gave the isomeric composition of the product: 84% *e.e.*.

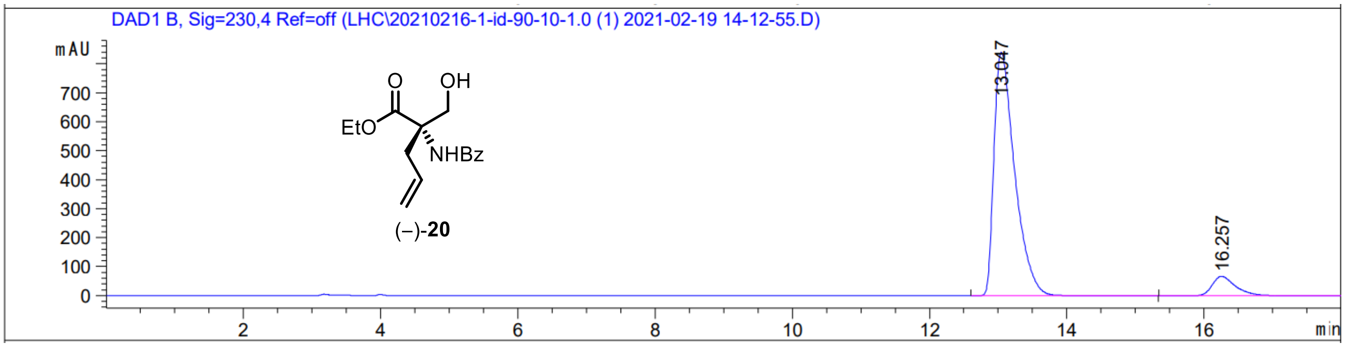

Signal 2: DAD1 B, Sig=230,4 Ref=off

Peak #	RetTime [min]	Type	Width [min]	Area [mAU*s]	Height [mAU]	Area %
1	13.047	BB	0.3187	1.76125e4	839.64526	91.8198
2	16.257	VV R	0.3512	1569.09949	66.61512	8.1802
Totals :				1.91816e4	906.26038	

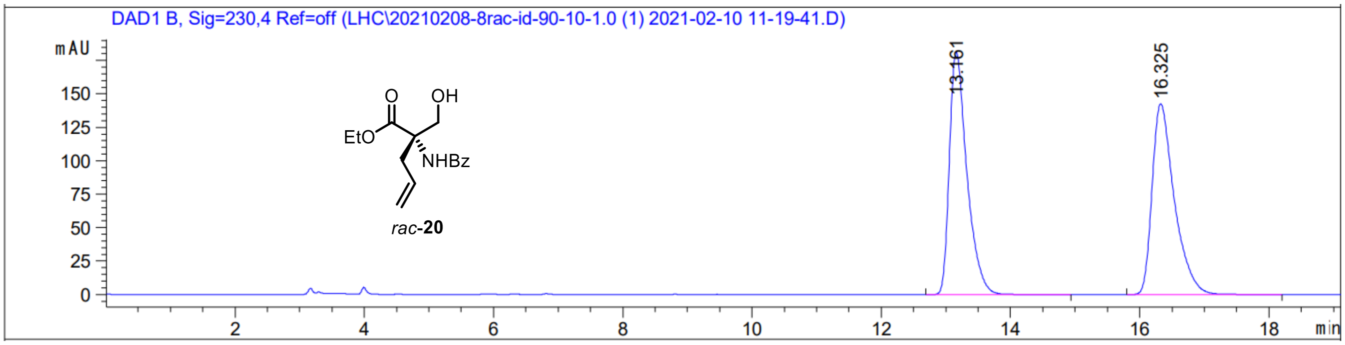

Signal 2: DAD1 B, Sig=230,4 Ref=off

Peak #	RetTime [min]	Type	Width [min]	Area [mAU*s]	Height [mAU]	Area %
1	13.161	BV R	0.2809	3415.96729	181.94101	50.0257
2	16.325	BB	0.3591	3412.45068	142.66536	49.9743
Totals :				6828.41797	324.60637	

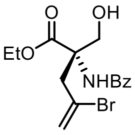

(–)-**21**

(–)-**21** was obtained as colorless oil (87.0 mg, 82% Yield) from the reductive desymmetrization of malonic

ester **S21** (119.1 mg, 0.3 mmol) with **L10** in toluene at -10 °C for 48 h using the general procedure A. R_f = 0.2 (hexane/EtOAc = 3:1). $[\alpha]_D^{25}$ = –52.5 (c = 1.0, $CHCl_3$).

^{1}H NMR (400 MHz, $CDCl_3$) δ 7.79 (m, 2H), 7.53-7.50 (m, 2H), 7.44 (m, 2H), 5.66 (s, 1H), 5.52 (d, J = 1.2 Hz, 1H), 4.41 (d, J = 9.6 Hz, 1H), 4.33 (m, 2H), 3.93-3.90 (m, 2H), 3.73 (d, J = 14.8 Hz, 1H), 3.04 (d, J = 14.8 Hz, 1H), 1.36 (t, J = 7.2 Hz, 3H).

^{13}C NMR (100 MHz, $CDCl_3$) δ 171.75, 167.79, 134.08, 131.96, 128.68, 127.04, 126.25, 122.53, 66.25, 66.05, 62.92, 42.01, 13.98.

IR (neat, cm^{-1}) 3405, 2933, 1732, 1645, 1518, 1227, 712.

HRMS (ESI) calcd $C_{15}H_{19}NO_4Br^+$ $[M+H]^+$: 356.0492. Found: 356.0491.

HPLC analysis (Chiralpak IF-3, hexane/^{i}PrOH = 90/10, 1.0 mL/min, 230 nm; t_r (minor) = 11.77 min, t_r (major) = 9.62 min) gave the isomeric composition of the product: 88% *e.e.*.

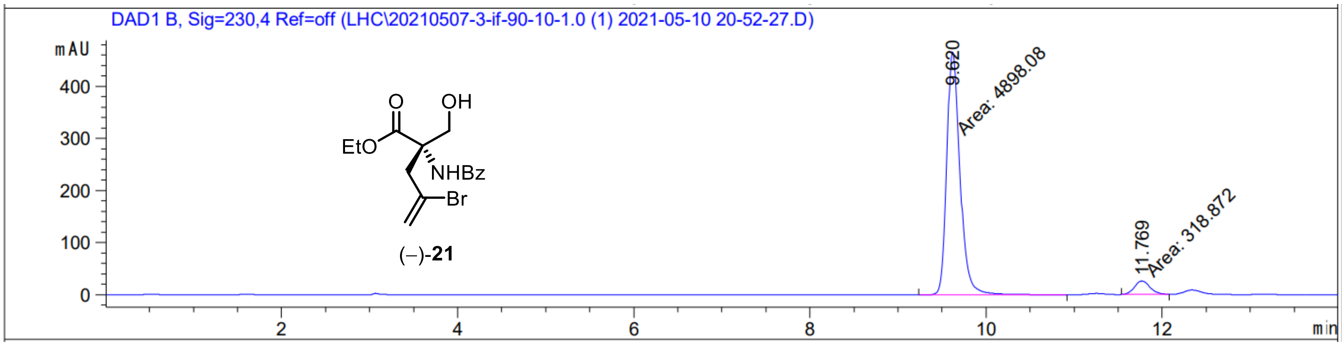

Signal 2: DAD1 B, Sig=230,4 Ref=off

Peak #	RetTime [min]	Type	Width [min]	Area [mAU*s]	Height [mAU]	Area %
1	9.620	MM	0.1749	4898.07520	466.63571	93.8878
2	11.769	MM	0.2055	318.87201	25.85943	6.1122

Totals : 5216.94720 492.49514

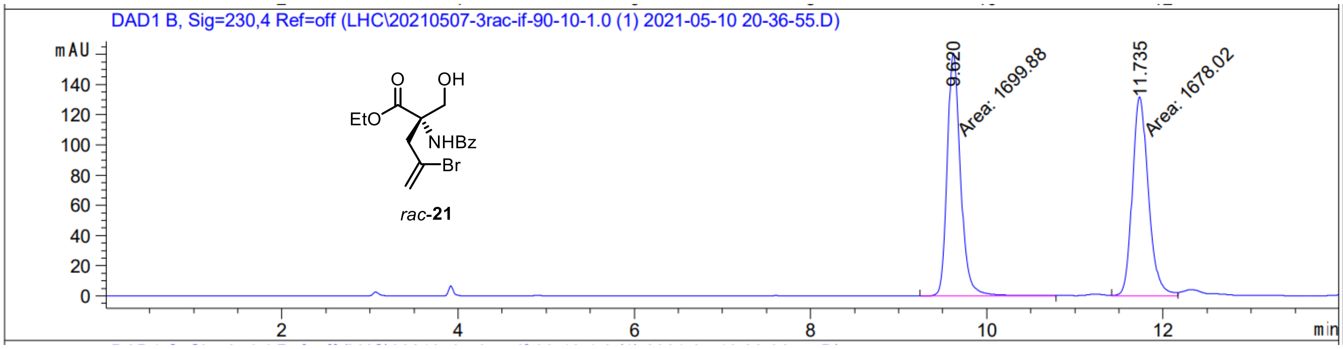

Signal 2: DAD1 B, Sig=230,4 Ref=off

Peak #	RetTime [min]	Type	Width [min]	Area [mAU*s]	Height [mAU]	Area %
1	9.620	MM	0.1759	1699.87524	161.05203	50.3235
2	11.735	MM	0.2121	1678.01758	131.83376	49.6765
Totals :				3377.89282	292.88579	

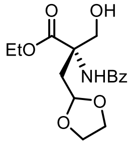

(–)-**22**

(–)-**22** was obtained as colorless oil (68.0 mg, 70% Yield) from the reductive desymmetrization of malonic ester **S22** (109.5 mg, 0.3 mmol) with **L10** in toluene at -10 °C for 60 h using the general procedure A. R_f = 0.2 (hexane/EtOAc = 3:1). $[\alpha]_D^{25}$ = –4.3 (c = 1.0, $CHCl_3$).

^{1}H NMR (400 MHz, $CDCl_3$) δ 7.87 (s, 1H), 7.81 (m, 2H), 7.51 (m, 1H), 7.44 (m, 2H), 5.03 (t, J = 4.4 Hz, 1H), 4.45 (br s, 1H), 4.29-4.20 (m, 3H), 3.97-3.85 (m, 5H), 2.53 (dd, J = 14.8, 4.8 Hz, 1H), 2.32 (dd, J = 14.8, 4.4 Hz, 1H), 1.29 (t, J = 7.2 Hz, 3H).

^{13}C NMR (100 MHz, $CDCl_3$) δ 171.70, 167.63, 134.02, 131.77, 128.57, 127.04, 101.80, 66.76, 64.98, 64.74, 63.63, 62.03, 36.21, 14.04.

IR (neat, cm^{-1}) 3400, 2898, 1731, 1649, 1520, 1226, 1108, 713.

HRMS (ESI) calcd $C_{16}H_{22}NO_6^+$ $[M+H]^+$: 324.1442. Found: 324.1442.

HPLC analysis (Chiralpak IF-3, hexane/^{i}PrOH = 70/30, 0.7 mL/min, 230 nm; t_r (major) = 13.49 min, t_r (minor) = 14.97 min) gave the isomeric composition of the product: 89% *e.e.*.

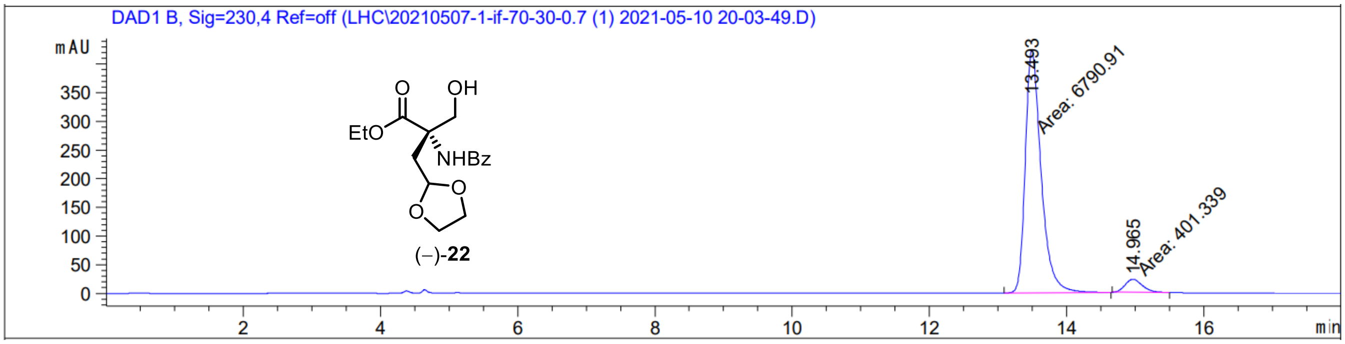

Signal 2: DAD1 B, Sig=230,4 Ref=off

Peak #	RetTime [min]	Type	Width [min]	Area [mAU*s]	Height [mAU]	Area %
1	13.493	MM	0.2675	6790.90918	423.09805	94.4198
2	14.965	MM	0.2935	401.33917	22.79396	5.5802
Totals :				7192.24835	445.89201	

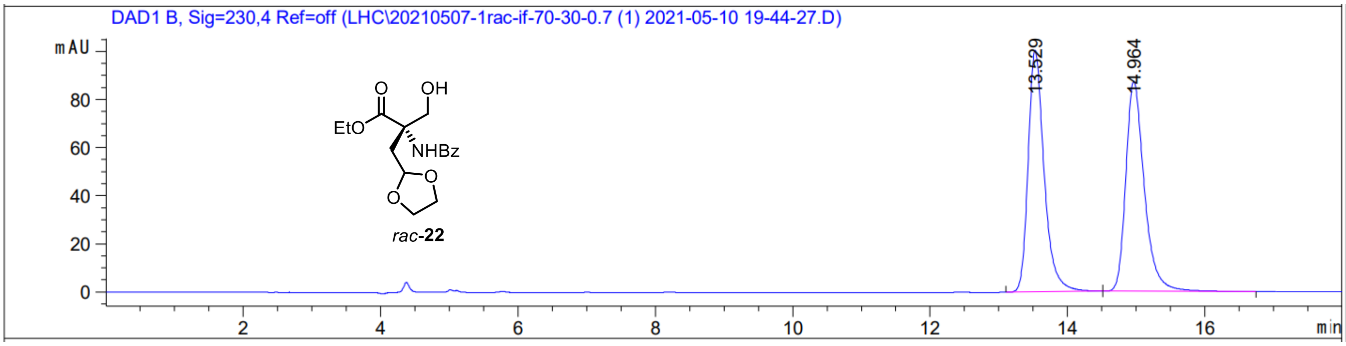

Signal 2: DAD1 B, Sig=230,4 Ref=off

Peak #	RetTime [min]	Type	Width [min]	Area [mAU*s]	Height [mAU]	Area %
1	13.529	BB	0.2470	1626.99646	100.43127	49.8887
2	14.964	BB	0.2845	1634.25696	87.25481	50.1113
Totals :				3261.25342	187.68609	

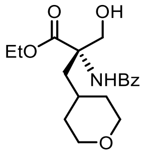

(–)-**23**

(–)-**23** was obtained as colorless oil (74.0 mg, 74% Yield) from the reductive desymmetrization of malonic

ester **S23** (113.1 mg, 0.3 mmol) with **L10** in toluene at -10 °C for 48 h using the general procedure A. R_f = 0.2 (hexane/EtOAc = 3:1). $[\alpha]_D^{25}$ = –14.8 (*c* = 1.0, $CHCl_3$).

¹H NMR (400 MHz, $CDCl_3$) δ 7.76-7.74 (m, 2H), 7.49-7.39 (m, 4H), 4.39 (d, *J* = 11.2 Hz, 1H), 4.28 (q, *J* = 7.2 Hz, 2H), 3.81-3.78 (m, 3H), 3.58 (br s, 1H), 3.23 (m, 2H), 2.49 (dd, *J* = 14.4, 4.8 Hz, 1H), 1.72 (dd, *J* = 14.4, 7.6 Hz, 1H), 1.51-1.39 (m, 3H), 1.32 (t, *J* = 7.2 Hz, 3H), 1.28-1.23 (m, 2H).

¹³C NMR (100 MHz, $CDCl_3$) δ 173.69, 166.87, 134.11, 131.79, 128.63, 126.84, 67.76, 67.52, 65.99, 65.82, 62.46, 37.25, 33.40, 32.75, 31.08, 14.01.

IR (neat, cm^{-1}) 3405, 2913, 1726, 1661, 1482, 1228, 710.

HRMS (ESI) calcd $C_{18}H_{26}NO_5^+$ $[M+H]^+$: 336.1805. Found: 336.1806.

HPLC analysis (Chiralpak IF-3, hexane/^{i}PrOH = 90/10, 1.0 mL/min, 230 nm; t_r (major) = 14.75 min, t_r (minor) = 17.06 min) gave the isomeric composition of the product: 94% *e.e.*.

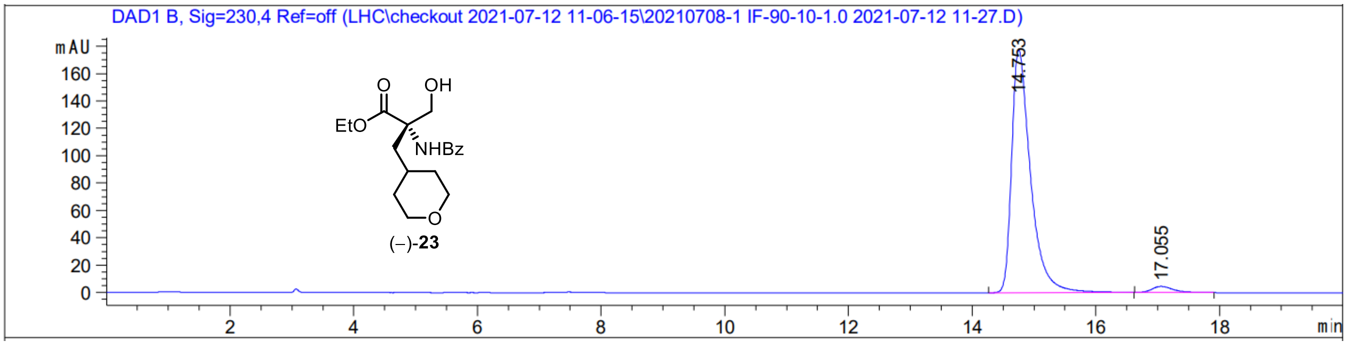

Signal 2: DAD1 B, Sig=230,4 Ref=off

Peak #	RetTime [min]	Type	Width [min]	Area [mAU*s]	Height [mAU]	Area %
1	14.753	BB	0.3197	3772.40430	177.68062	97.2719
2	17.055	BBA	0.3564	105.80177	4.43374	2.7281
Totals :				3878.20607	182.11435	

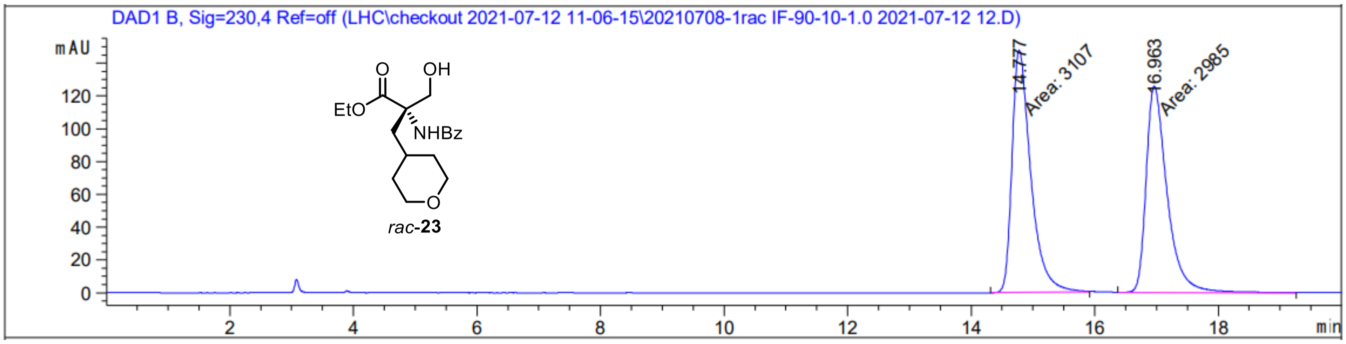

Signal 2: DAD1 B, Sig=230,4 Ref=off

Peak #	RetTime [min]	Type	Width [min]	Area [mAU*s]	Height [mAU]	Area %
1	14.777	MM	0.3508	3107.00024	147.60500	51.0013
2	16.963	MM	0.3949	2985.00122	125.96828	48.9987
Totals :				6092.00146	273.57328	

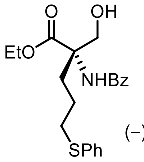

(–)-**24**

(–)-**24** was obtained as colorless oil (89.3 mg, 77% Yield) from the reductive desymmetrization of malonic ester **S24** (128.7 mg, 0.3 mmol) with **L10** in toluene at -10 °C for 96 h using the general procedure A. R_f = 0.2 (hexane/EtOAc = 3:1). $[\alpha]_D^{25}$ = –20.3 (c = 1.0, $CHCl_3$).

^{1}H NMR (400 MHz, $CDCl_3$) δ 7.75 (m, 2H), 7.51 (m, 1H), 7.42 (m, 2H), 7.35 (s, 1H), 7.25 (d, J = 7.2 Hz, 2H), 7.18 (m, 2H), 7.11 (m, 1H), 4.38 (d, J = 11.2 Hz, 1H), 4.24 (m, 2H), 3.90 (d, J = 11.2 Hz, 1H), 3.58 (br s, 1H), 2.92-2.86 (m, 2H), 2.58 (m, 1H), 2.01 (m, 1H), 1.65 (m, 1H), 1.43 (m, 1H), 1.28 (m, 3H).

^{13}C NMR (100 MHz, $CDCl_3$) δ 172.69, 167.23, 135.82, 133.98, 131.87, 129.27, 128.81, 128.63, 126.97, 125.96, 66.81, 65.84, 62.56, 33.29, 30.26, 23.40, 14.04.

IR (neat, cm^{-1}) 3400, 2938, 1728, 1645, 1519, 1227, 690.

HRMS (ESI) calcd $C_{21}H_{26}NO_4S^+$ $[M+H]^+$: 388.1577. Found: 388.1579.

HPLC analysis (Chiralpak IF-3, hexane/^{i}PrOH = 90/10, 1.0 mL/min, 205 nm; t_r (minor) = 15.38 min, t_r (major) = 18.13 min) gave the isomeric composition of the product: 85% *e.e.*.

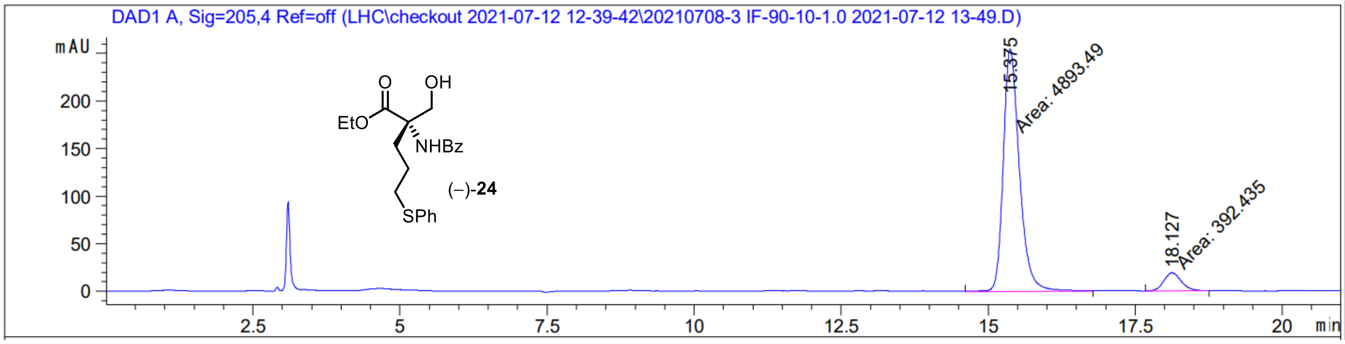

Signal 1: DAD1 A, Sig=205,4 Ref=off

Peak #	RetTime [min]	Type	Width [min]	Area [mAU*s]	Height [mAU]	Area %
1	15.375	MM	0.3200	4893.48535	254.85773	92.5758
2	18.127	MM	0.3452	392.43509	18.94995	7.4242
Totals :				5285.92044	273.80767	

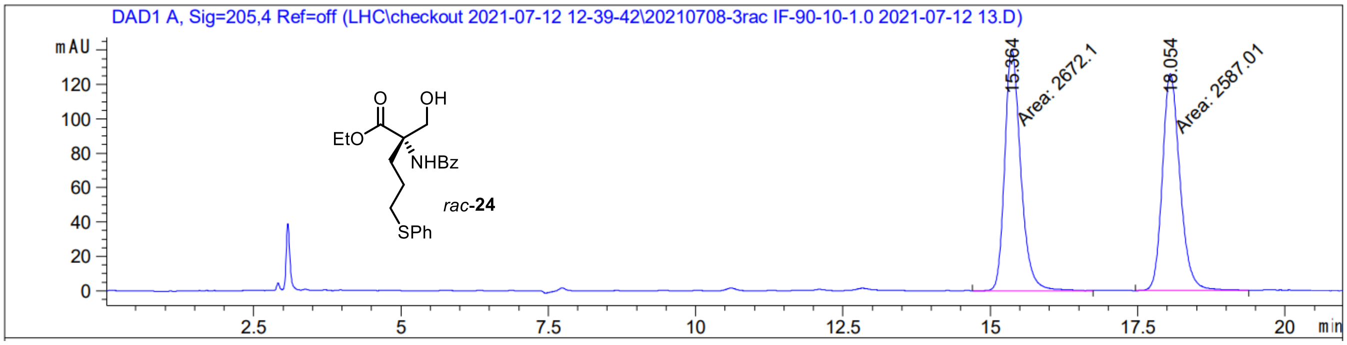

Signal 1: DAD1 A, Sig=205,4 Ref=off

Peak #	RetTime [min]	Type	Width [min]	Area [mAU*s]	Height [mAU]	Area %
1	15.364	MM	0.3168	2672.10376	140.57170	50.8091
2	18.054	MM	0.3423	2587.00513	125.94640	49.1909
Totals :				5259.10889	266.51810	

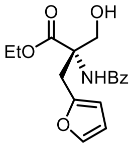

(–)-**25**

(–)-**25** was obtained as colorless oil (77.8 mg, 82% Yield) from the reductive desymmetrization of malonic

ester **S25** (107.7 mg, 0.3 mmol) with **L10** in toluene at -10 °C for 48 h using the general procedure A. R_f = 0.2 (hexane/EtOAc = 3:1). $[\alpha]_D^{25}$ = –57.2 (*c* = 1.0, $CHCl_3$).

^{1}H NMR (400 MHz, $CDCl_3$) δ 7.69 (d, *J* = 7.2 Hz, 2H), 7.50 (m, 1H), 7.41 (dd, *J* = 7.6, 7.2 Hz, 2H), 7.29 (d, *J* = 1.2 Hz, 1H), 7.22 (s, 1H), 6.25 (dd, *J* = 3.2, 2.0 Hz, 1H), 6.07 (d, *J* = 3.2 Hz, 1H), 4.39-4.30 (m, 3H), 4.09 (br s, 1H), 3.97 (d, *J* = 11.6 Hz, 1H), 3.74 (d, *J* = 14.8 Hz, 1H), 3.28 (d, *J* = 14.8 Hz, 1H), 1.34 (t, *J* = 7.2 Hz, 3H).

^{13}C NMR (100 MHz, $CDCl_3$) δ 171.84, 167.92, 149.95, 142.17, 134.17, 131.84, 128.61, 127.01, 110.43, 108.80, 66.24, 65.99, 62.58, 30.57, 14.04.

IR (neat, cm^{-1}) 3405, 2978, 1733, 1644, 1520, 1229, 1009, 714.

HRMS (ESI) calcd $C_{17}H_{20}NO_5^+$ $[M+H]^+$: 318.1336. Found: 318.1338.

HPLC analysis (Chiralpak ID-3, hexane/^{i}PrOH = 80/20, 0.8 mL/min, 205 nm; t_r (minor) = 16.57 min, t_r (major) = 17.36 min) gave the isomeric composition of the product: 90% *e.e.*.

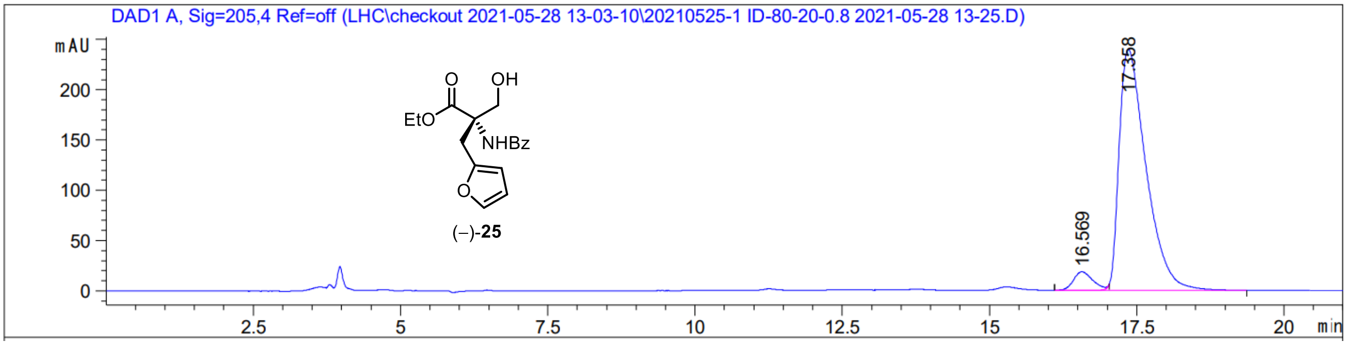

Signal 1: DAD1 A, Sig=205,4 Ref=off

Peak #	RetTime [min]	Type	Width [min]	Area [mAU*s]	Height [mAU]	Area %
1	16.569	BV E	0.3439	416.99969	18.43332	5.1922
2	17.358	VB R	0.4780	7614.29883	240.08292	94.8078
Totals :				8031.29852	258.51623	

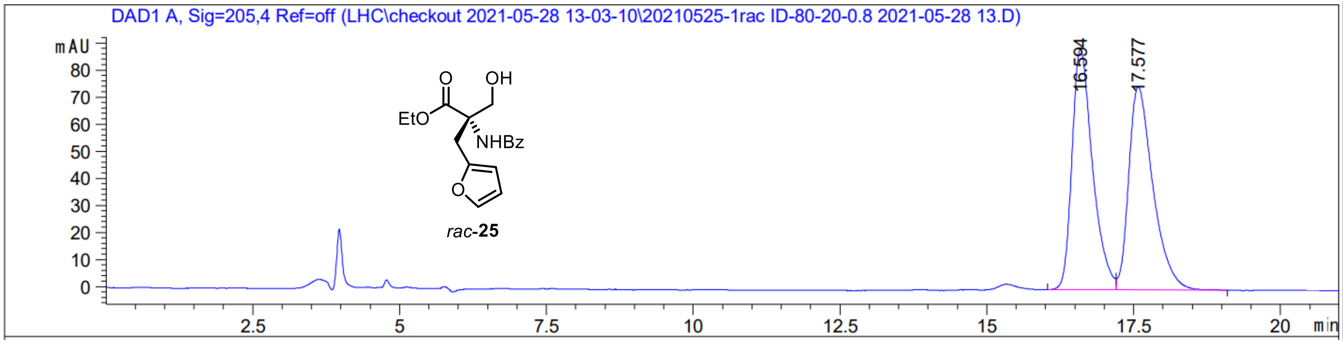

Signal 1: DAD1 A, Sig=205,4 Ref=off

Peak #	RetTime [min]	Type	Width [min]	Area [mAU*s]	Height [mAU]	Area %
1	16.594	BV	0.3683	2168.55957	88.97462	50.1021
2	17.577	VB	0.4337	2159.71973	75.05688	49.8979

Totals : 4328.27930 164.03149

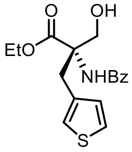

(–)-**26**

(–)-**26** was obtained as colorless oil (78.7 mg, 79% Yield) from the reductive desymmetrization of malonic ester **S26** (112.5 mg, 0.3 mmol) with **L10** in toluene at -10 °C for 48 h using the general procedure A. R_f = 0.2 (hexane/EtOAc = 3:1). $[\alpha]_D^{25}$ = –74.5 (*c* = 1.0, $CHCl_3$).

^{1}H NMR (400 MHz, $CDCl_3$) δ 7.67 (d, *J* = 7.2 Hz, 2H), 7.51 (m, 1H), 7.41 (dd, *J* = 7.6, 7.2 Hz, 2H), 7.22 (d, *J* = 2.0 Hz, 1H), 7.13 (s, 1H), 6.96 (s, 1H), 6.80 (dd, *J* = 4.8, 1.2 Hz, 1H), 4.44 (d, *J* = 9.2 Hz, 1H), 4.29 (m, 2H), 4.02-3.99 (m, 2H), 3.74 (d, *J* = 14.0 Hz, 1H), 3.25 (d, *J* = 14.0 Hz, 1H), 1.35 (t, *J* = 7.2 Hz, 3H).

^{13}C NMR (100 MHz, $CDCl_3$) δ 172.08, 167.75, 135.31, 134.15, 131.91, 128.68, 128.57, 126.97, 125.87, 123.44, 67.49, 66.07, 62.54, 31.84, 14.12.

IR (neat, cm^{-1}) 3395, 2928, 1730, 1644, 1519, 1229, 713.

HRMS (ESI) calcd $C_{17}H_{20}NO_4S^+$ $[M+H]^+$: 334.1108. Found: 334.1110.

HPLC analysis (Chiralpak IF-3, hexane/^{i}PrOH = 80/20, 0.9 mL/min, 205 nm; t_r (minor) = 9.17 min, t_r (major) = 8.26 min) gave the isomeric composition of the product: 92% *e.e.*.

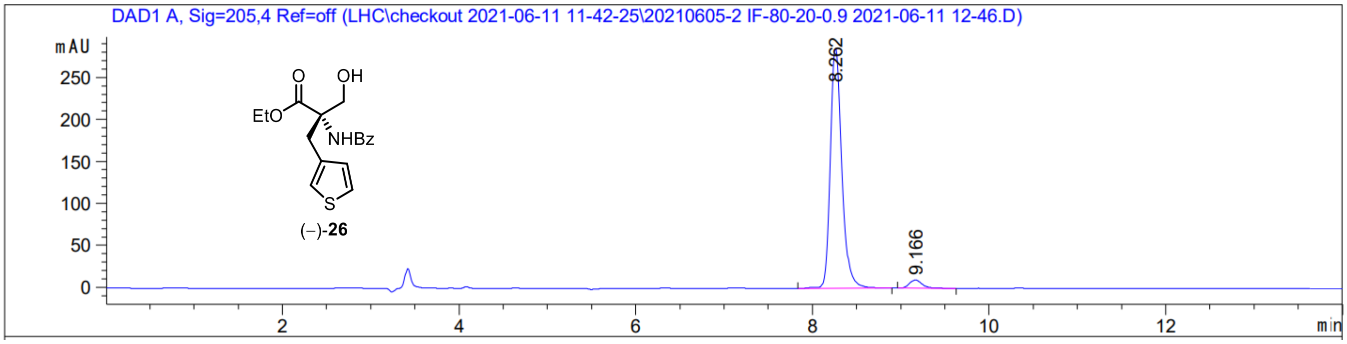

Peak #	RetTime [min]	Type	Width [min]	Area [mAU*s]	Height [mAU]	Area %
1	8.262	VB R	0.1370	2580.57983	285.54913	96.3414
2	9.166	BB	0.1523	97.99881	9.83071	3.6586

Totals : 2678.57864 295.37984

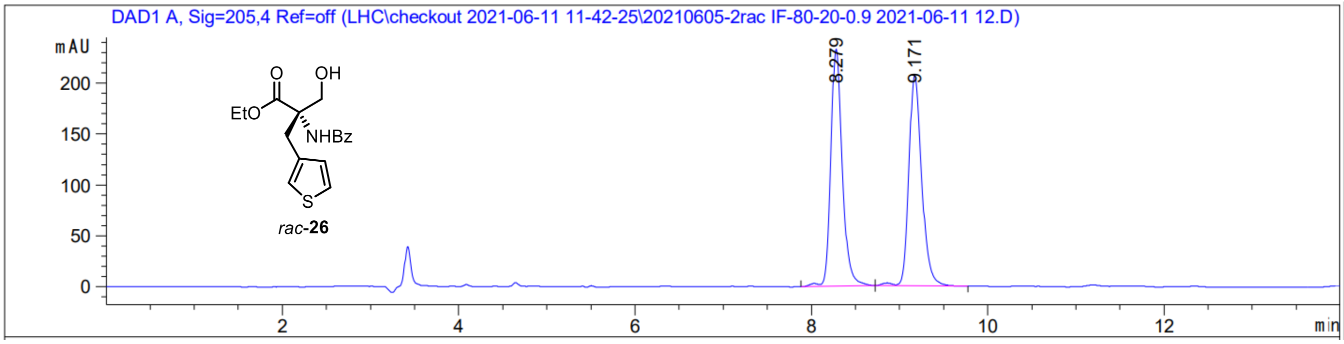

Signal 1: DAD1 A, Sig=205,4 Ref=off

Peak #	RetTime [min]	Type	Width [min]	Area [mAU*s]	Height [mAU]	Area %
1	8.279	VB R	0.1370	2117.68970	232.96631	50.0530
2	9.171	VB R	0.1540	2113.20630	206.98236	49.9470

Totals : 4230.89600 439.94867

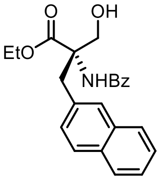

(–)-**27**

(–)-**27** was obtained as colorless oil (95.0 mg, 84% Yield) from the reductive desymmetrization of malonic ester **S27** (125.7 mg, 0.3 mmol) with **L10** in toluene at -10 °C for 48 h using the general procedure A. R_f = 0.2 (hexane/EtOAc = 3:1). $[\alpha]_D^{25}$ = –95.1 (*c* = 1.0, $CHCl_3$).

^{1}H NMR (400 MHz, $CDCl_3$) δ 7.79-7.36 (m, 11H), 7.21 (dd, *J* = 8.4, 1.6 Hz, 1H), 7.13 (s, 1H), 4.56 (dd, *J* = 10.4, 4.8 Hz, 1H), 4.31 (q, *J* = 7.2 Hz, 2H), 4.11-4.05 (m, 2H), 3.87 (d, *J* = 14.0 Hz, 1H), 3.38 (d, *J* = 14.0 Hz, 1H), 1.38 (t, *J* = 7.2 Hz, 3H).

^{13}C NMR (100 MHz, $CDCl_3$) δ 171.95, 167.97, 134.23, 133.30, 132.84, 132.46, 131.89, 128.72, 128.66, 128.07, 127.78, 127.58, 127.45, 127.00, 126.15, 125.78, 68.03, 66.19, 62.58, 37.15, 14.16.

IR (neat, cm^{-1}) 3405, 2933, 1728, 1645, 1520, 1222, 713.

HRMS (ESI) calcd $C_{23}H_{24}NO_4^+$ $[M+H]^+$: 378.1700. Found: 378.1696.

HPLC analysis (Chiralpak IF-3, hexane/^{i}PrOH = 90/10, 1.0 mL/min, 254 nm; t_r (major) = 14.88 min, t_r (minor) = 16.32 min) gave the isomeric composition of the product: 91% *e.e.*.

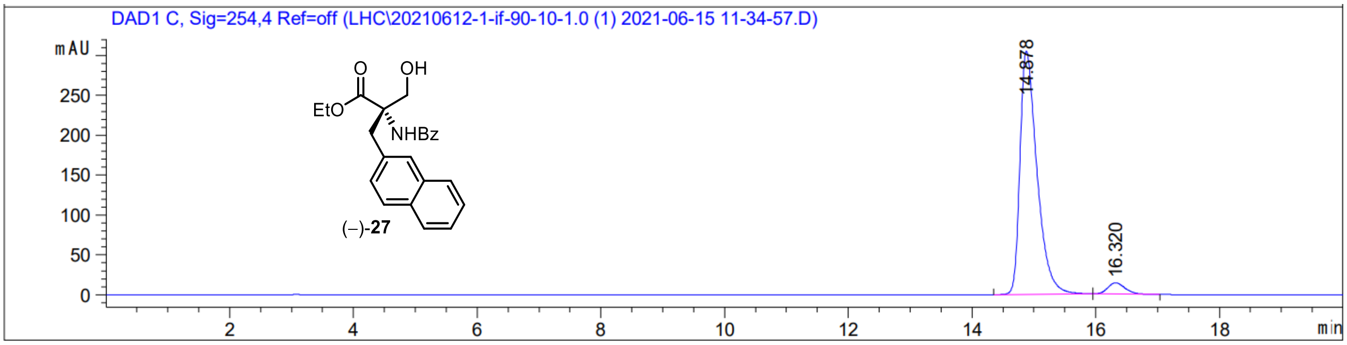

Signal 3: DAD1 C, Sig=254,4 Ref=off

Peak #	RetTime [min]	Type	Width [min]	Area [mAU*s]	Height [mAU]	Area %
1	14.878	BB	0.2866	5826.33398	305.37320	95.4353
2	16.320	BB	0.3010	278.67508	14.19747	4.5647

Totals : 6105.00906 319.57067

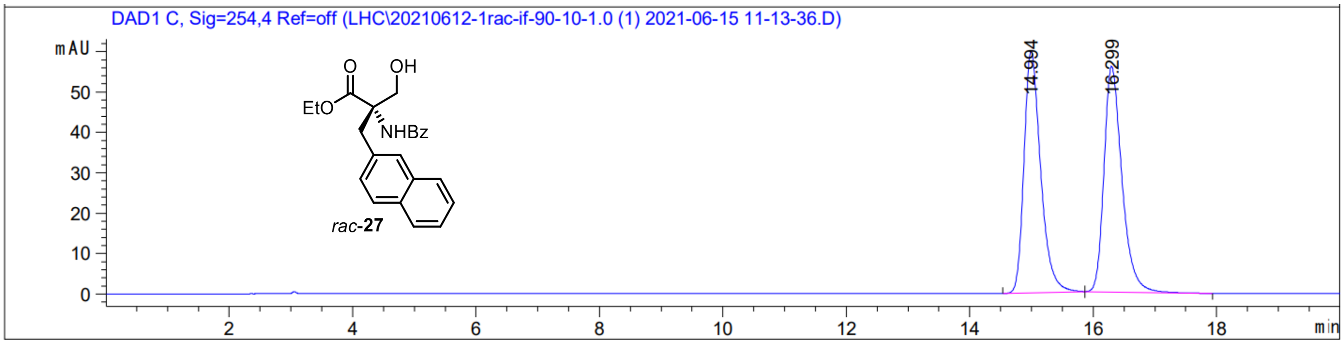

Signal 3: DAD1 C, Sig=254,4 Ref=off

Peak #	RetTime [min]	Type	Width [min]	Area [mAU*s]	Height [mAU]	Area %
1	14.994	BB	0.2869	1123.56213	59.89293	49.9202
2	16.299	BB	0.3075	1127.15259	55.83214	50.0798
Totals :				2250.71472	115.72507	

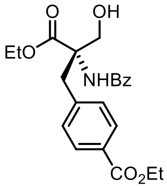

(–)-**28**

(–)-**28** was obtained as colorless oil (91.0 mg, 76% Yield) from the reductive desymmetrization of malonic ester **S28** (132.3 mg, 0.3 mmol) with **L10** in toluene at -10 °C for 48 h using the general procedure A. R_f = 0.2 (hexane/EtOAc = 3:1). $[\alpha]_D^{25}$ = –99.7 (c = 1.0, $CHCl_3$).

^{1}H NMR (400 MHz, $CDCl_3$) δ 7.89 (d, J = 8.4, Hz, 2H), 7.67 (d, J = 6.8 Hz, 2H), 7.52-7.49 (m, 1H), 7.43-7.39 (m, 2H), 7.15-7.12 (m, 3H), 4.53 (d, J = 10.0 Hz, 1H), 4.35-4.26 (m, 4H), 4.03 (d, J = 11.6 Hz, 1H), 3.87 (br s, 1H), 3.81 (d, J = 13.6 Hz, 1H), 3.22 (d, J = 13.6 Hz, 1H), 1.36 (m, 6H).

^{13}C NMR (100 MHz, $CDCl_3$) δ 171.75, 167.75, 166.35, 140.73, 134.02, 131.99, 129.72, 129.58, 129.37, 128.72, 126.92, 67.84, 65.96, 62.72, 60.93, 36.67, 14.26, 14.11.

IR (neat, cm^{-1}) 3405, 2983, 1714, 1645, 1274, 1104, 707.

HRMS (ESI) calcd $C_{22}H_{26}NO_6^+$ [M+H]$^+$: 400.1755. Found: 400.1750.

HPLC analysis (Chiralpak IA-3, hexane/^{i}PrOH = 90/10, 1.0 mL/min, 230 nm; t_r (major) = 13.10 min, t_r (minor) = 14.19 min) gave the isomeric composition of the product: 90% *e.e.*.

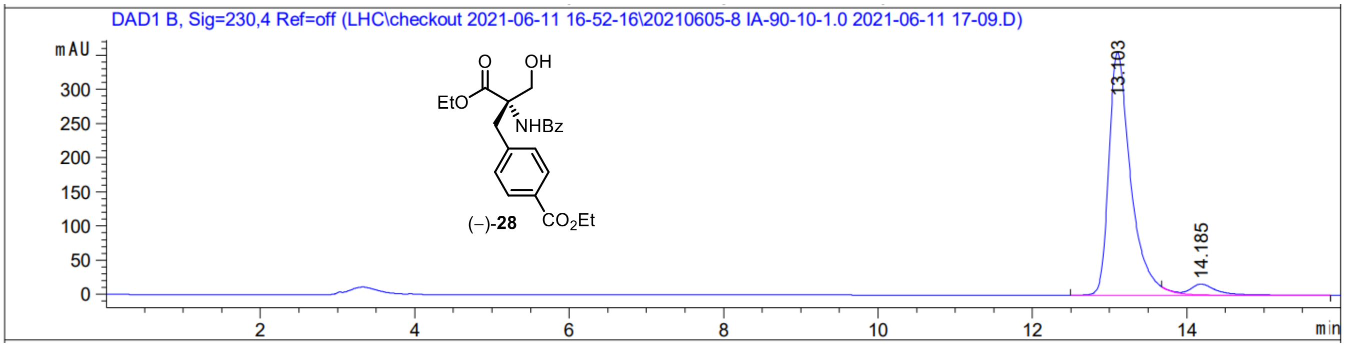

Signal 2: DAD1 B, Sig=230,4 Ref=off

Peak #	RetTime [min]	Type	Width [min]	Area [mAU*s]	Height [mAU]	Area %
1	13.103	BV R	0.2951	7053.62500	356.14578	94.9480
2	14.185	VB E	0.3441	375.31348	15.85776	5.0520

Totals : 7428.93848 372.00354

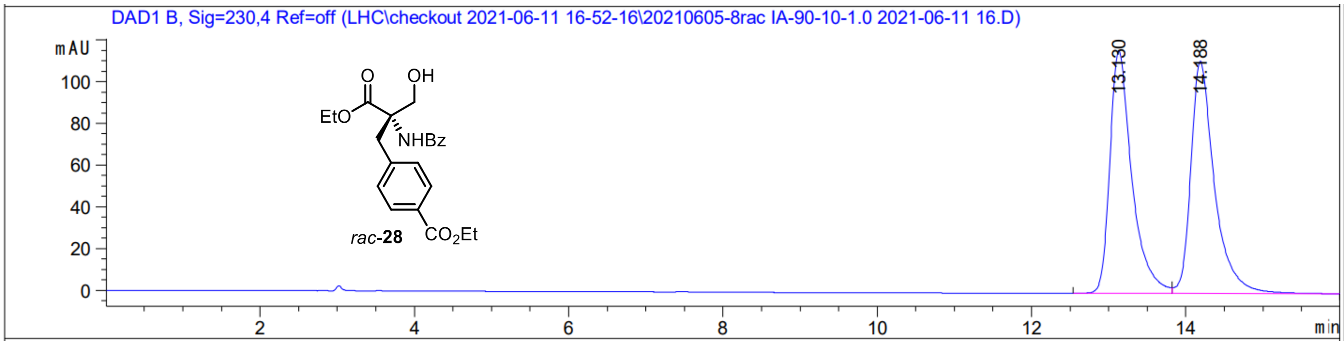

Signal 2: DAD1 B, Sig=230,4 Ref=off

Peak #	RetTime [min]	Type	Width [min]	Area [mAU*s]	Height [mAU]	Area %
1	13.130	BV	0.2957	2313.35107	116.46119	49.2680
2	14.188	VBA	0.3176	2382.09497	111.33614	50.7320

Totals : 4695.44604 227.79733

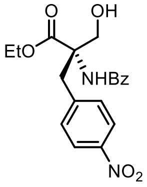

(–)-**29**

(–)-**29** was obtained as colorless oil (83.6 mg, 75% Yield) from the reductive desymmetrization of malonic ester **S29** (124.2 mg, 0.3 mmol) with **L10** in toluene at -10 °C for 48 h using the general procedure A. R_f = 0.2 (hexane/EtOAc = 3:1). $[\alpha]_D^{25}$ = –89.0 (*c* = 1.0, $CHCl_3$).

^{1}H NMR (400 MHz, $CDCl_3$) δ 8.06 (d, *J* = 8.8 Hz, 2H), 7.68 (d, *J* = 7.2 Hz, 2H), 7.52 (m, 1H), 7.42 (m, 2H), 7.23-7.20 (m, 3H), 4.54 (d, *J* = 11.2 Hz, 1H), 4.31 (m, 2H), 4.05 (d, *J* = 11.6 Hz, 1H), 3.92 (d, *J* = 13.6 Hz, 1H), 3.68 (br s, 1H), 3.28 (d, *J* = 13.6 Hz, 1H), 1.38 (t, *J* = 7.2 Hz, 3H).

^{13}C NMR (100 MHz, $CDCl_3$) δ 171.56, 167.75, 147.14, 143.37, 133.75, 132.21, 130.57, 128.82, 126.86, 123.49, 67.74, 65.83, 62.98, 36.27, 14.15.

IR (neat, cm^{-1}) 3405, 2923, 1645, 1517, 1344, 1223, 715.

HRMS (ESI) calcd $C_{19}H_{21}N_2O_6^+$ $[M+H]^+$: 373.1394. Found: 373.1392.

HPLC analysis (Chiralpak IF-3, hexane/^{i}PrOH = 90/10, 1.0 mL/min, 205 nm; t_r (major) = 18.48 min, t_r (minor) = 19.98 min) gave the isomeric composition of the product: 91% *e.e.*.

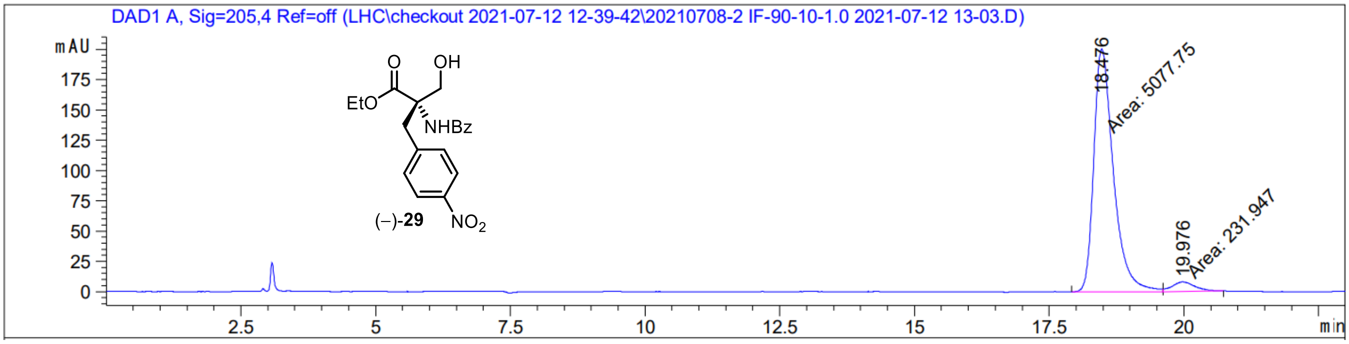

Signal 1: DAD1 A, Sig=205,4 Ref=off

Peak #	RetTime [min]	Type	Width [min]	Area [mAU*s]	Height [mAU]	Area %
1	18.476	MM	0.4222	5077.74756	200.42705	95.6316
2	19.976	MM	0.4921	231.94737	7.85516	4.3684
Totals :				5309.69493	208.28221	

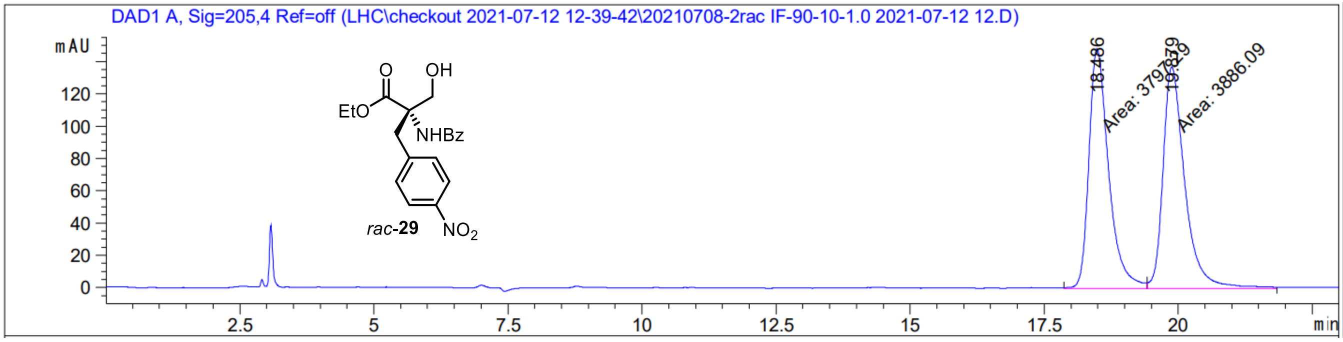

Signal 1: DAD1 A, Sig=205,4 Ref=off

Peak #	RetTime [min]	Type	Width [min]	Area [mAU*s]	Height [mAU]	Area %
1	18.486	MM	0.4263	3797.29272	148.46487	49.4222
2	19.879	MM	0.4718	3886.08813	137.28329	50.5778
Totals :				7683.38086	285.74817	

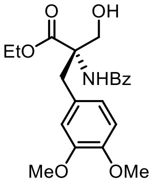

(–)-**30**

(–)-**30** was obtained as colorless oil (97.4 mg, 84% Yield) from the reductive desymmetrization of malonic ester **S30** (128.7 mg, 0.3 mmol) with **L10** in toluene at -10 °C for 48 h using the general procedure A. R_f = 0.2 (hexane/EtOAc = 2:1). $[\alpha]_D^{25}$ = -70.4 (c = 1.0, $CHCl_3$).

^{1}H NMR (400 MHz, $CDCl_3$) δ 7.68 (d, J = 7.2 Hz, 2H), 7.50 (m, 1H), 7.40 (t, J = 7.6 Hz, 2H), 7.12 (s, 1H), 6.73 (d, J = 8.0 Hz, 1H), 6.61 (dd, J = 8.0, 1.6 Hz, 1H), 6.57 (s, 1H), 4.48 (dd, J = 10.8, 4.4 Hz, 1H), 4.30 (m, 2H), 4.03-3.96 (m, 2H), 3.82 (s, 3H), 3.61 (d, J = 14.0 Hz, 1H), 3.57 (s, 3H), 3.15 (d, J = 14.0 Hz, 1H), 1.37 (t, J = 7.2 Hz, 3H).

^{13}C NMR (100 MHz, $CDCl_3$) δ 172.06, 167.65, 148.69, 148.11, 134.08, 131.98, 128.67, 127.62, 126.94, 121.63, 112.83, 111.02, 68.07, 66.02, 62.46, 55.75, 55.39, 53.39, 36.57, 14.14.

IR (neat, cm^{-1}) 3405, 2933, 1733, 1647, 1514, 1233, 1024, 715.

HRMS (ESI) calcd $C_{21}H_{26}NO_6^+$ $[M+H]^+$: 388.1755. Found: 388.1748.

HPLC analysis (Chiralpak IG-3, hexane/^{i}PrOH = 70/30, 0.7 mL/min, 230 nm; t_r (major) = 16.33 min, t_r

(minor) = 18.08 min) gave the isomeric composition of the product: 91% *e.e.*.

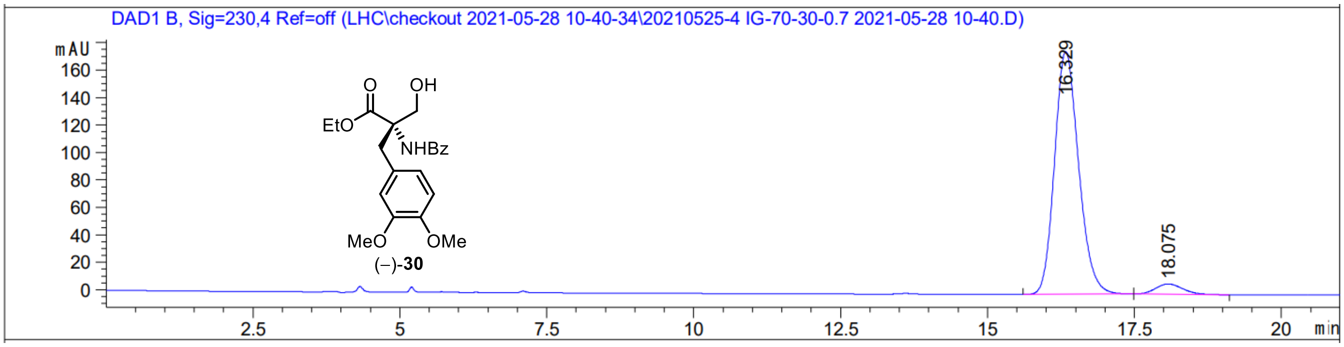

Signal 2: DAD1 B, Sig=230,4 Ref=off

Peak #	RetTime [min]	Type	Width [min]	Area [mAU*s]	Height [mAU]	Area %
1	16.329	BB	0.4399	5029.75293	176.80701	95.4405
2	18.075	BB	0.4887	240.28520	7.48218	4.5595

Totals : 5270.03813 184.28919

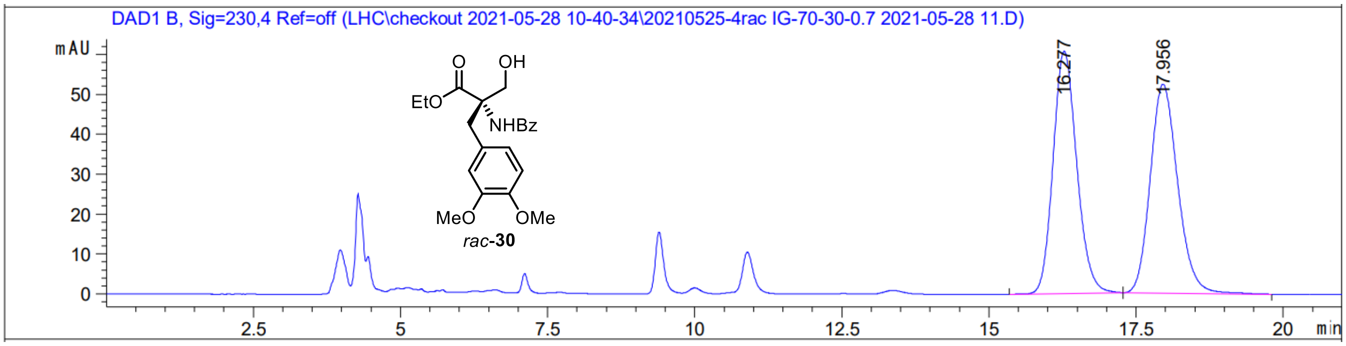

Signal 2: DAD1 B, Sig=230,4 Ref=off

Peak #	RetTime [min]	Type	Width [min]	Area [mAU*s]	Height [mAU]	Area %
1	16.277	BB	0.4322	1698.05164	60.72791	50.0032
2	17.956	BB	0.5005	1697.83191	52.32623	49.9968

Totals : 3395.88354 113.05414

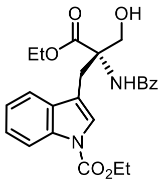

(–)-**31**

(–)-**31** was obtained as colorless oil (92.0 mg, 70% Yield) from the reductive desymmetrization of malonic ester **S31** (144.0 mg, 0.3 mmol) with **L10** in toluene at -10 °C for 48 h using the general procedure A. R_f = 0.2 (hexane/EtOAc = 3:1). $[\alpha]_D^{25}$ = –70.6 (c = 1.0, $CHCl_3$).

^{1}H NMR (400 MHz, $CDCl_3$) δ 8.13 (d, J = 8.0 Hz, 1H), 7.64 (d, J = 7.2 Hz, 2H), 7.50-7.46 (m, 2H), 7.39-7.34 (m, 3H), 7.30-7.26 (m, 1H), 7.21 (s, 1H), 7.14 (dd, J = 7.6, 7.2 Hz, 1H), 4.53 (m, 1H), 4.40 (q, J = 7.2 Hz, 2H), 4.25-4.07 (m, 4H), 3.81 (d, J = 14.8 Hz, 1H), 3.35 (d, J = 14.8 Hz, 1H), 1.36 (t, J = 7.2 Hz, 3H), 1.30 (t, J = 7.2 Hz, 3H).

^{13}C NMR (100 MHz, $CDCl_3$) δ 172.15, 167.97, 150.63, 135.13, 134.12, 131.84, 130.61, 128.54, 126.99, 124.74, 124.38, 122.78, 118.95, 115.14, 114.80, 67.40, 65.97, 63.08, 62.64, 26.83, 14.25, 13.96.

IR (neat, cm^{-1}) 3400, 2928, 1731, 1645, 1251, 1074, 747.

HRMS (ESI) calcd $C_{24}H_{27}N_2O_6^+$ $[M+H]^+$: 439.1864. Found: 439.1865.

HPLC analysis (Chiralpak IF-3, hexane/^{i}PrOH = 70/30, 0.7 mL/min, 254 nm; t_r (minor) = 11.13 min, t_r (major) = 9.69 min) gave the isomeric composition of the product: 92% *e.e.*.

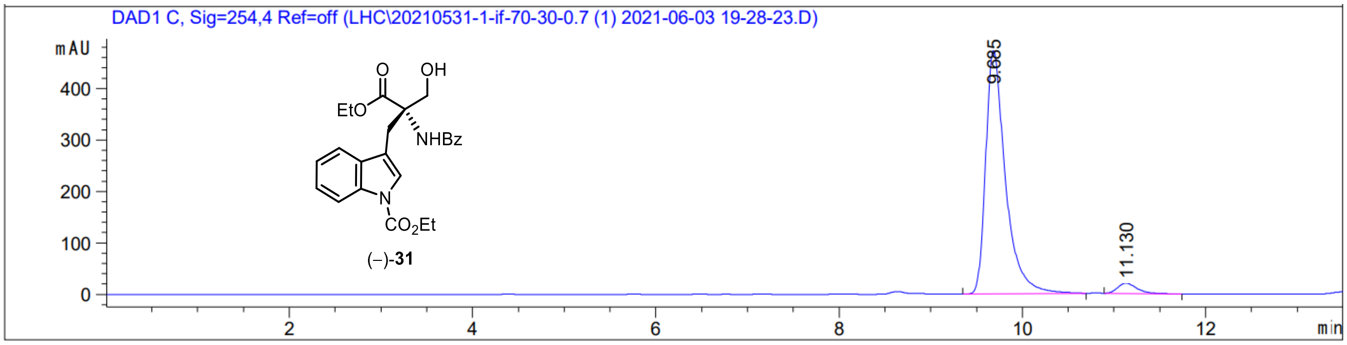

Signal 3: DAD1 C, Sig=254,4 Ref=off

Peak #	RetTime [min]	Type	Width [min]	Area [mAU*s]	Height [mAU]	Area %
1	9.685	BB	0.2252	7097.26855	472.53409	96.0859
2	11.130	BB	0.2206	289.10770	20.00516	3.9141

Totals : 7386.37625 492.53925

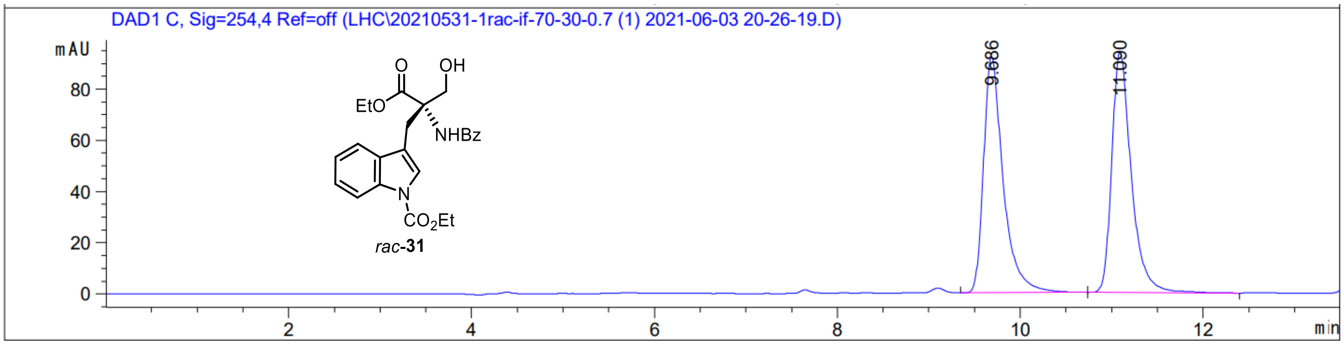

Signal 3: DAD1 C, Sig=254,4 Ref=off

Peak #	RetTime [min]	Type	Width [min]	Area [mAU*s]	Height [mAU]	Area %
1	9.686	BB	0.2248	1378.12964	91.99305	49.6893
2	11.090	BB	0.2233	1395.36157	93.93636	50.3107

Totals : 2773.49121 185.92941

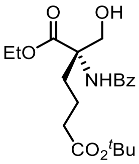

(–)-**32**

(–)-**32** was obtained as colorless oil (79.7 mg, 70% Yield) from the reductive desymmetrization of malonic ester **S32** (126.3 mg, 0.3 mmol) with **L10** in toluene at -10 °C for 60 h using the general procedure A. R_f = 0.2 (hexane/EtOAc = 3:1). $[\alpha]_D^{25}$ = –1.6 (*c* = 1.0, $CHCl_3$).

^{1}H NMR (400 MHz, $CDCl_3$) δ 7.84 (dd, *J* = 8.0, 1.6 Hz, 2H), 7.53-7.42 (m, 4H), 4.36-4.27 (m, 3H), 3.90 (d, *J* = 11.2 Hz, 1H), 3.77 (br s, 1H), 2.35 (m, 1H), 2.22 (m, 2H), 1.87 (m, 1H), 1.62-1.58 (m, 1H), 1.47-1.41 (m, 1H), 1.41 (s, 9H), 1.32 (t, *J* = 7.2 Hz, 3H).

^{13}C NMR (100 MHz, $CDCl_3$) δ 172.67, 172.64, 167.43, 133.99, 131.85, 128.59, 127.11, 80.55, 66.49, 66.05, 62.39, 34.74, 31.11, 28.03, 19.08, 14.10.

IR (neat, cm^{-1}) 3405, 2978, 1726, 1648, 1229, 1150, 713.

HRMS (ESI) calcd $C_{20}H_{30}NO_6^+$ $[M+H]^+$: 380.2068. Found: 380.2066.

HPLC analysis (Chiralpak IF-3, hexane/^{i}PrOH = 90/10, 1.0 mL/min, 205 nm; t_r (major) = 12.17 min, t_r (minor) = 14.25 min) gave the isomeric composition of the product: 81% *e.e.*.

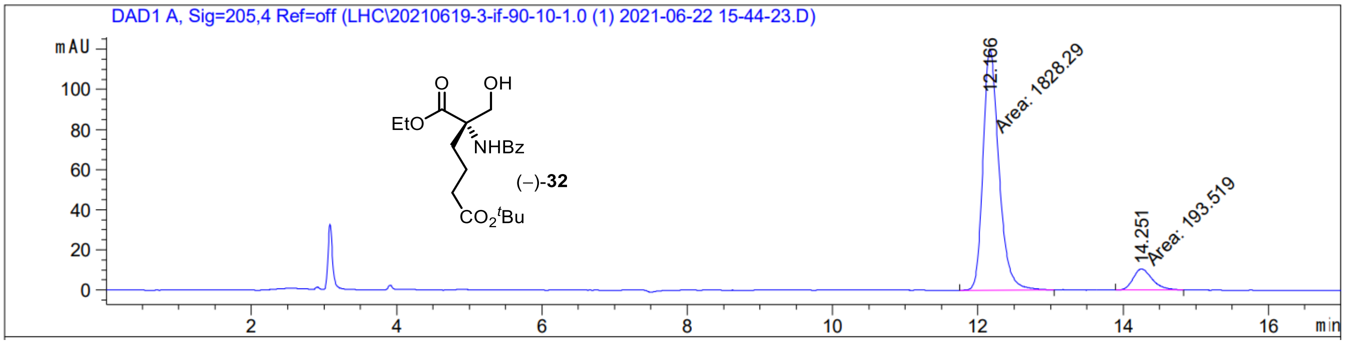

Signal 1: DAD1 A, Sig=205,4 Ref=off

Peak #	RetTime [min]	Type	Width [min]	Area [mAU*s]	Height [mAU]	Area %
1	12.166	MM	0.2535	1828.29419	120.22159	90.4284
2	14.251	MM	0.3064	193.51932	10.52547	9.5716

Totals : 2021.81351 130.74706

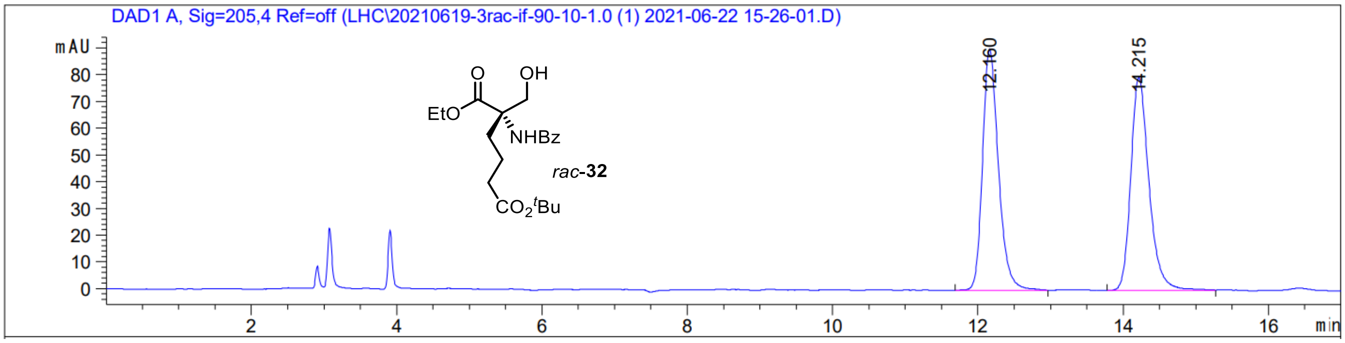

Signal 1: DAD1 A, Sig=205,4 Ref=off

Peak #	RetTime [min]	Type	Width [min]	Area [mAU*s]	Height [mAU]	Area %
1	12.160	BB	0.2310	1367.18860	90.12041	49.7263
2	14.215	BB	0.2645	1382.23804	79.63391	50.2737

Totals : 2749.42664 169.75433

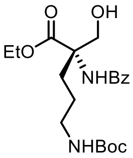

(–)-**33**

(–)-**33** was obtained as colorless oil (95.4 mg, 81% Yield) from the reductive desymmetrization of malonic

ester **S33** (130.8 mg, 0.3 mmol) with **L10** in toluene at -10 °C for 72 h using the general procedure A. R_f = 0.2 (hexane/EtOAc = 3:1). $[\alpha]_D^{25}$ = –15.2 (c = 1.0, $CHCl_3$).

^{1}H NMR (400 MHz, $CDCl_3$) δ 7.79 (d, J = 7.2 Hz, 2H), 7.51 (m, 1H), 7.42 (m, 3H), 4.63 (br s, 1H), 4.36-4.28 (m, 3H), 3.90 (d, J = 11.2 Hz, 1H), 3.66 (br s, 1H), 3.08 (m, 2H), 2.44 (m, 1H), 1.86 (m, 1H), 1.47-1.44 (m, 2H), 1.38 (s, 9H), 1.31 (t, J = 7.2 Hz, 3H).

^{13}C NMR (100 MHz, $CDCl_3$) δ 172.73, 167.28, 155.90, 134.00, 131.88, 128.62, 127.02, 79.18, 66.60, 65.74, 62.52, 40.17, 28.60, 28.30, 24.42, 14.10.

IR (neat, cm^{-1}) 3405, 2924, 1649, 1519, 1166, 714.

HRMS (ESI) calcd $C_{20}H_{31}N_2O_6^+$ $[M+H]^+$: 395.2177. Found: 395.2178.

HPLC analysis (Chiralpak IF-3, hexane/^{i}PrOH = 90/10, 1.0 mL/min, 230 nm; t_r (major) = 16.38 min, t_r (minor) = 18.22 min) gave the isomeric composition of the product: 91% *e.e.*.

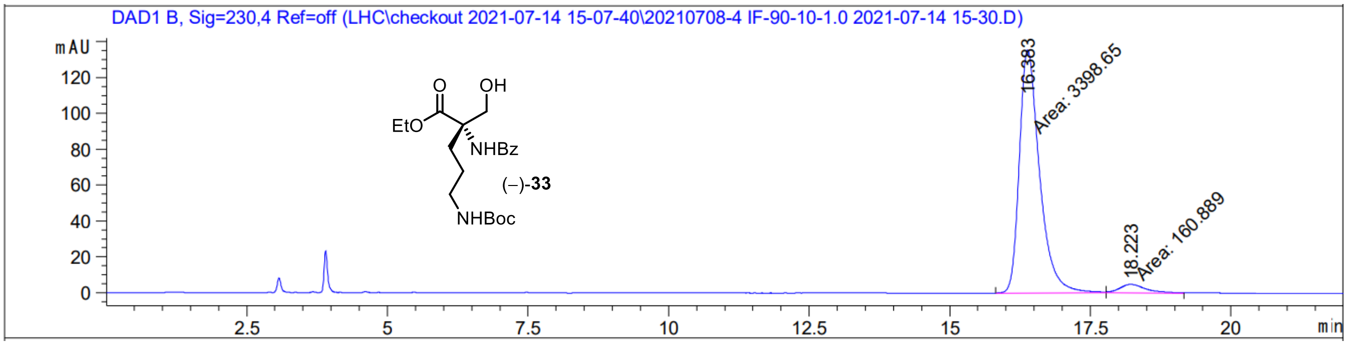

Signal 2: DAD1 B, Sig=230,4 Ref=off

Peak #	RetTime [min]	Type	Width [min]	Area [mAU*s]	Height [mAU]	Area %
1	16.383	MM	0.4180	3398.64771	135.52779	95.4801
2	18.223	MM	0.5643	160.88892	4.75179	4.5199

Totals : 3559.53662 140.27958

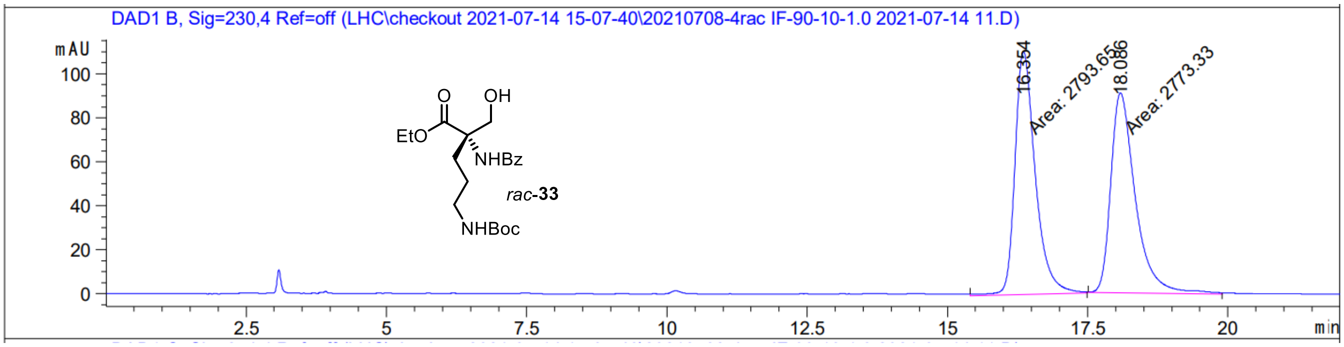

Signal 2: DAD1 B, Sig=230,4 Ref=off

Peak #	RetTime [min]	Type	Width [min]	Area [mAU*s]	Height [mAU]	Area %
1	16.354	MM	0.4212	2793.64722	110.53867	50.1824
2	18.086	MM	0.5085	2773.33447	90.90450	49.8176

Totals : 5566.98169 201.44316

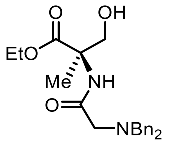

(–)-**34**

(–)-**34** was obtained as colorless oil (86.3 mg, 75% Yield) from the reductive desymmetrization of malonic ester **S34** (127.8 mg, 0.3 mmol) with **L9** in toluene at 0 °C for 96 h using the general procedure C. R_f = 0.2 (hexane/EtOAc = 1:1). $[\alpha]_D^{25}$ = –8.0 (c = 1.0, $CHCl_3$).

^{1}H NMR (400 MHz, $CDCl_3$) δ 8.28 (br s, 1H), 7.39-7.26 (m, 10H), 4.26 (m, 2H), 3.97 (d, J = 11.2 Hz, 1H), 3.73 (br s, 1H), 3.73-3.62 (m, 5H), 3.11 (s, 2H), 1.43 (s, 3H), 1.28 (t, J = 7.2 Hz, 3H).

^{13}C NMR (100 MHz, $CDCl_3$) δ 172.81, 171.70, 137.79, 129.06, 128.54, 127.55, 66.75, 62.17, 62.03, 59.41, 57.68, 20.20, 14.05.

IR (neat, cm^{-1}) 2933, 1733, 1659, 1454, 1127, 699.

HRMS (ESI) calcd $C_{22}H_{29}N_2O_4^+$ $[M+H]^+$: 385.2122. Found: 385.2119.

HPLC analysis (Chiralpak IB-3, hexane/^{i}PrOH = 97/3, 1.0 mL/min, 205 nm; t_r (minor) = 18.25 min, t_r (major) = 19.28 min) gave the isomeric composition of the product: 92% *e.e.*.

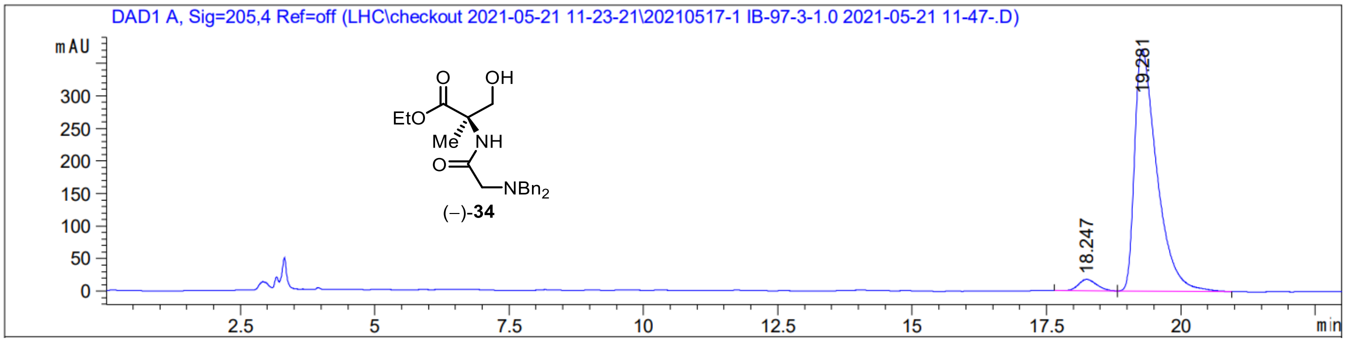

Signal 1: DAD1 A, Sig=205,4 Ref=off

Peak #	RetTime [min]	Type	Width [min]	Area [mAU*s]	Height [mAU]	Area %
1	18.247	BV	0.3586	424.98911	17.79537	3.8134
2	19.281	VB	0.4303	1.07195e4	371.89532	96.1866

Totals : 1.11445e4 389.69070

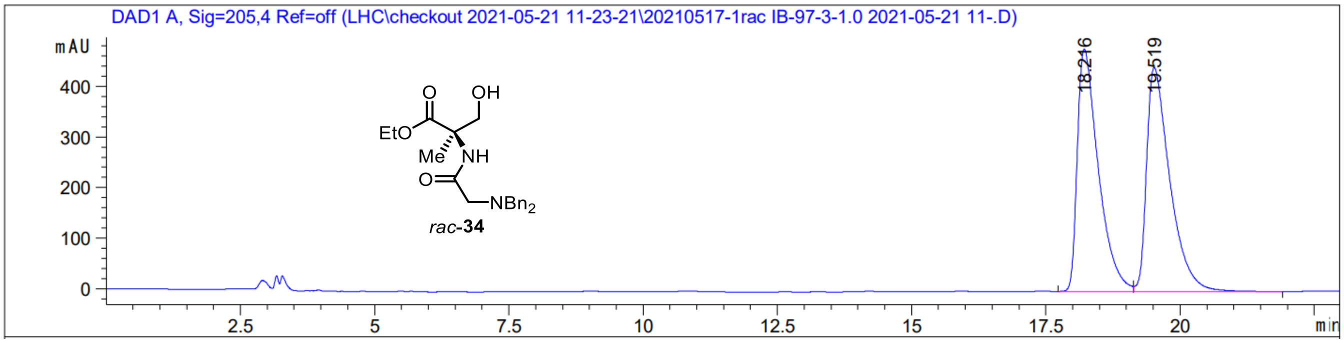

Signal 1: DAD1 A, Sig=205,4 Ref=off

Peak #	RetTime [min]	Type	Width [min]	Area [mAU*s]	Height [mAU]	Area %
1	18.216	BV	0.4082	1.30581e4	478.75275	49.3581
2	19.519	VB	0.4508	1.33977e4	443.06128	50.6419

Totals : 2.64558e4 921.81403

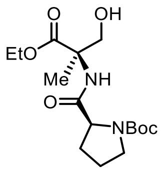

(–)-**35**

(–)-**35** was obtained as colorless oil (83.5 mg, 81% Yield) from the reductive desymmetrization of malonic ester **S35** (115.8 mg, 0.3 mmol) with (*S*)-**L9** in toluene at 0 °C for 72 h using the general procedure C. R_f = 0.2 (hexane/EtOAc = 1:1). Crude ^{1}H NMR analysis revealed that the *d.r.* was about 23:1. $[\alpha]_D^{25}$ = –82.3 (*c* = 1.0, $CHCl_3$).

^{1}H NMR (400 MHz, $CDCl_3$) (mixture of rotamers, 3:1) δ 7.14 (br s, 0.75H), 6.96 (br s, 0.25H), 4.24-4.12 (m, 4H), 3.81-3.43 (m, 4H), 2.17-2.07 (m, 2H), 1.90-1.85 (m, 2H), 1.53 (s, 3H), 1.44 (s, 9H), 1.27 (t, *J* = 7.2 Hz, 3H).

^{13}C NMR (100 MHz, $CDCl_3$) δ 173.17, 171.98, 155.61, 80.86, 66.56, 64.67, 61.88, 61.75, 61.17, 47.07, 31.45, 30.88, 29.42, 28.21, 24.47, 23.51, 19.65, 13.92.

IR (neat, cm^{-1}) 2978, 1734, 1667, 1393, 1124.

HRMS (ESI) calcd $C_{16}H_{29}N_2O_4^+$ $[M+H]^+$: 345.2020. Found: 345.2019.

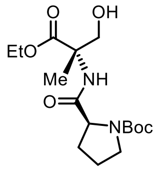

(–)-**36**

(–)-**36** was obtained as colorless oil (75.3 mg, 73% Yield) from the reductive desymmetrization of malonic ester **S36** (115.8 mg, 0.3 mmol) with (*R*)-**L9** in toluene at 0 °C for 72 h using the general procedure C. R_f = 0.2 (hexane/EtOAc = 1:1). Crude ^{1}H NMR analysis revealed that the *d.r.* was about 1:10. $[\alpha]_D^{25}$ = –52.4 (*c* = 1.0, $CHCl_3$).

^{1}H NMR (400 MHz, $CDCl_3$) (mixture of rotamers, 7:3) δ 7.15 (br s, 0.7H), 6.81 (br s, 0.3H), 4.23-4.11 (m, 5H), 3.77 (m, 1H), 3.44-3.38 (m, 2H), 2.10-1.84 (m, 4H), 1.48 (s, 3H), 1.42 (s, 9H), 1.25 (t, *J* = 7.2 Hz, 3H).

^{13}C NMR (100 MHz, $CDCl_3$) δ 173.25, 172.38, 155.32, 80.59, 65.04, 62.39, 61.83, 60.86, 47.06, 29.02, 28.26, 24.57, 19.66, 13.98.

IR (neat, cm^{-1}) 2933, 1749, 1658, 1415, 1133.

HRMS (ESI) calcd $C_{16}H_{29}N_2O_4^+$ $[M+H]^+$: 345.2020. Found: 345.2019.

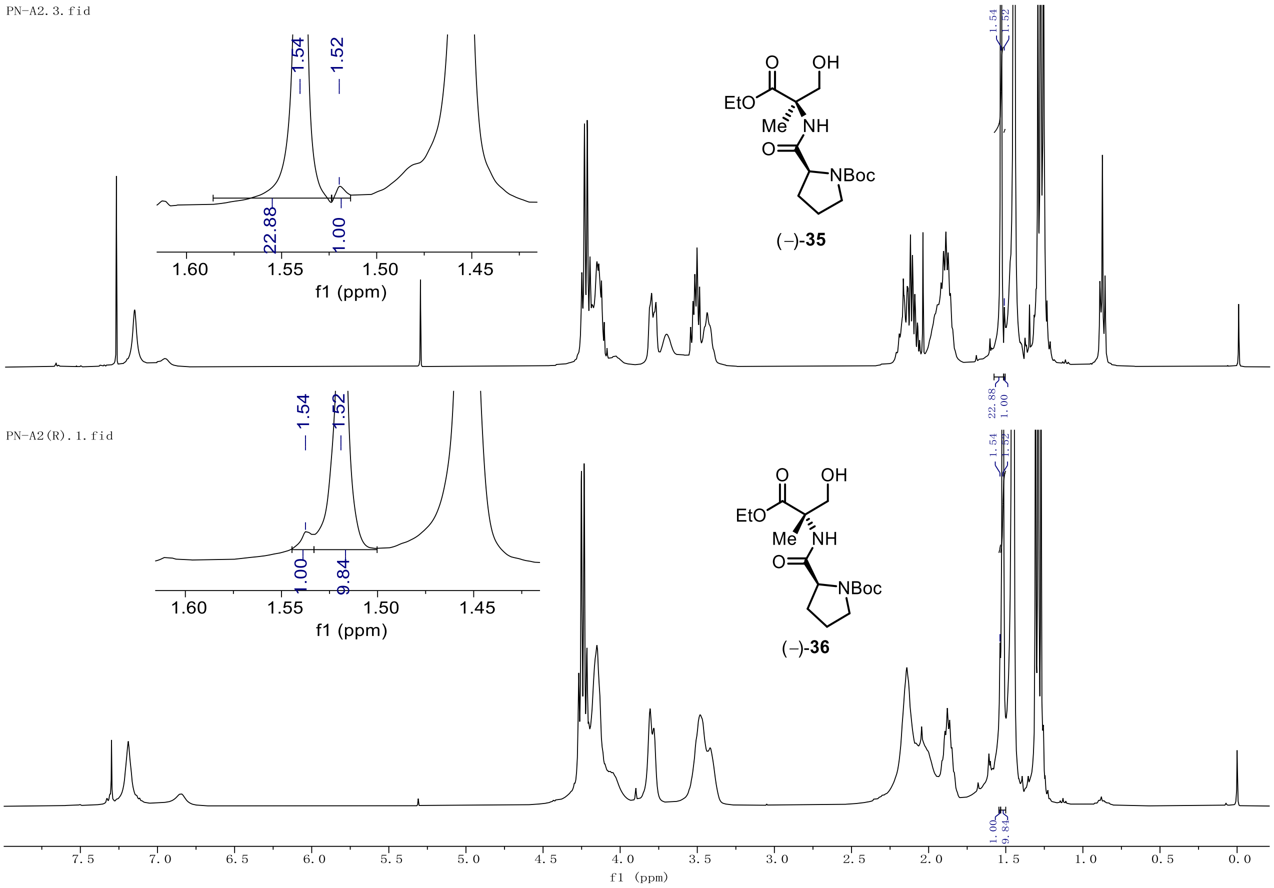

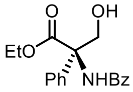

(+)-**37**

(+)-**37** was obtained as colorless oil (68.2 mg, 73% Yield) from the reductive desymmetrization of malonic ester **S37** (106.5 mg, 0.3 mmol) with **L11a** in toluene at 0 °C for 7 d using the general procedure B. R_f = 0.2 (Hexane /EtOAc 3:1). $[\alpha]_D^{25}$ = +10.4 (*c* = 0.5, $CHCl_3$).

^{1}H NMR (400 MHz, $CDCl_3$) δ 7.87 (d, *J* = 8.4 Hz, 2H), 7.75 (s, 1H), 7.55 (m, 1H), 7.48-7.46 (m, 4H), 7.39-7.32 (m, 3H), 4.85 (d, *J* = 11.6 Hz, 1H), 4.45 (d, *J* = 11.6 Hz, 1H), 4.27 (m, 2H), 3.76 (br s, 1H), 1.24 (t, *J* = 7.2 Hz, 3H).

^{13}C NMR (100 MHz, $CDCl_3$) δ 171.46, 167.15, 136.65, 133.89, 132.04, 128.74, 128.70, 128.29, 127.13, 126.21, 68.48, 65.08, 62.84, 13.86.

IR (neat, cm^{-1}) 3405, 2923, 1728, 1651, 1482, 1229, 694.

HRMS (ESI) calcd $C_{18}H_{20}NO_4^+$ $[M+H]^+$: 314.1387. Found: 314.1386.

HPLC analysis (Chiralpak IG-3, hexane/^{i}PrOH = 80/20, 1.0 mL/min, 230 nm; t_r (major) = 14.02 min, t_r (minor) = 16.57 min) gave the isomeric composition of the product: 92% *e.e.*.

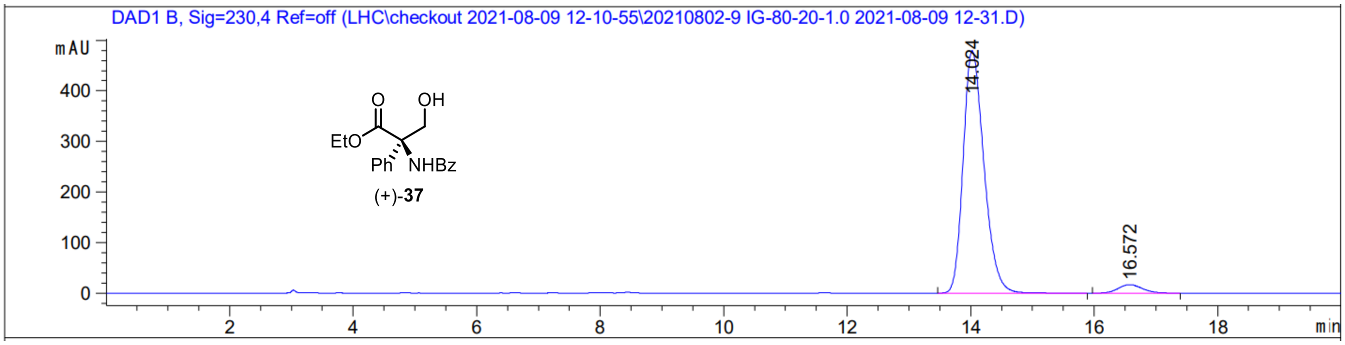

Signal 2: DAD1 B, Sig=230,4 Ref=off

Peak #	RetTime [min]	Type	Width [min]	Area [mAU*s]	Height [mAU]	Area %
1	14.024	BB	0.3610	1.12265e4	479.79285	96.0774
2	16.572	BB	0.4172	458.34875	16.96486	3.9226

Totals : 1.16849e4 496.75770

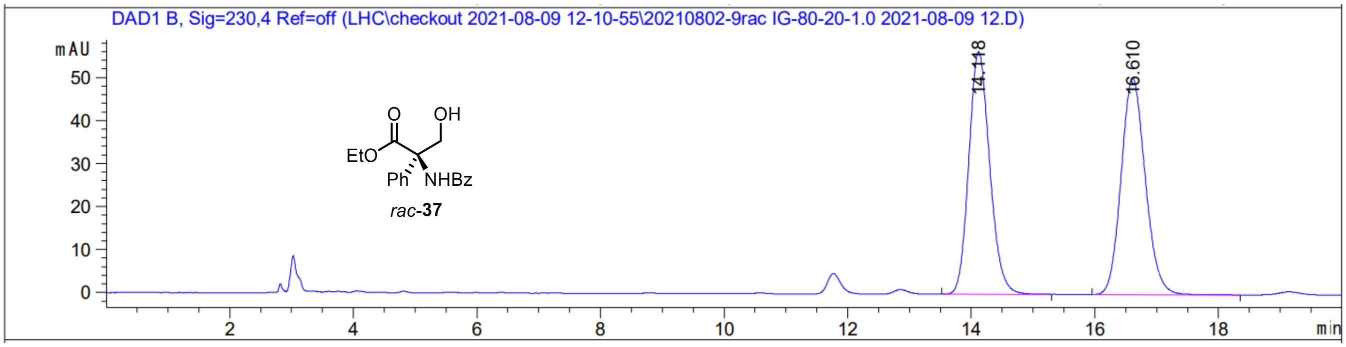

Signal 2: DAD1 B, Sig=230,4 Ref=off

Peak #	RetTime [min]	Type	Width [min]	Area [mAU*s]	Height [mAU]	Area %
1	14.118	BB	0.3645	1326.39221	56.38259	49.1975
2	16.610	BB	0.4195	1369.66248	50.33546	50.8025

Totals : 2696.05469 106.71805

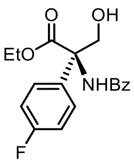

(+)-**38**

(+)-**38** was obtained as colorless oil (61.3 mg, 62% Yield) from the reductive desymmetrization of malonic ester **S38** (111.9 mg, 0.3 mmol) with **L11a** in toluene at 0 °C for 8 d using the general procedure B. R_f = 0.2 (hexane/EtOAc = 3:1). $[\alpha]_D^{25}$ = +13.5 (c = 0.5, $CHCl_3$).

^{1}**H NMR** (400 MHz, $CDCl_3$) δ 7.86 (m, 3H), 7.55 (m, 1H), 7.49-7.43 (m, 4H), 7.05 (t, J = 8.8 Hz, 2H), 4.83 (d, J = 11.6 Hz, 1H), 4.42 (d, J = 11.6 Hz, 1H), 4.26 (q, J = 7.2 Hz, 2H), 1.24 (t, J = 7.2 Hz, 3H).

^{13}**C NMR** (100 MHz, $CDCl_3$) δ 171.36, 167.08, 163.65, 161.18, 133.67, 132.18, 128.76, 128.24, 128.16, 127.12, 115.75, 115.54, 67.91, 64.98, 63.05, 13.86.

^{19}**F NMR** (376 MHz, $CDCl_3$) δ −113.77.

IR (neat, cm^{-1}) 3405, 2958, 1731, 1654, 1508, 1229, 1015, 711.

HRMS (ESI) calcd $C_{18}H_{19}FNO_4^+$ $[M+H]^+$: 332.1293. Found: 332.1292.

HPLC analysis (Chiralpak IF-3, hexane/^{i}PrOH = 85/15, 1.0 mL/min, 230 nm; t_r (major) = 10.66 min, t_r (minor) = 11.72 min) gave the isomeric composition of the product: 92% *e.e.*.

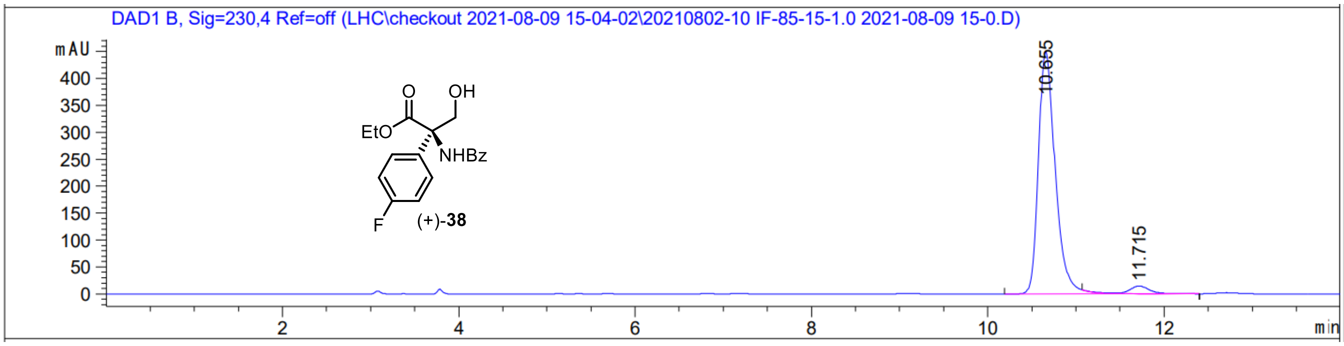

Signal 2: DAD1 B, Sig=230,4 Ref=off

Peak #	RetTime [min]	Type	Width [min]	Area [mAU*s]	Height [mAU]	Area %
1	10.655	BV R	0.2031	5987.64014	450.51721	96.1334
2	11.715	VB E	0.2446	240.83237	14.58678	3.8666

Totals : 6228.47250 465.10399

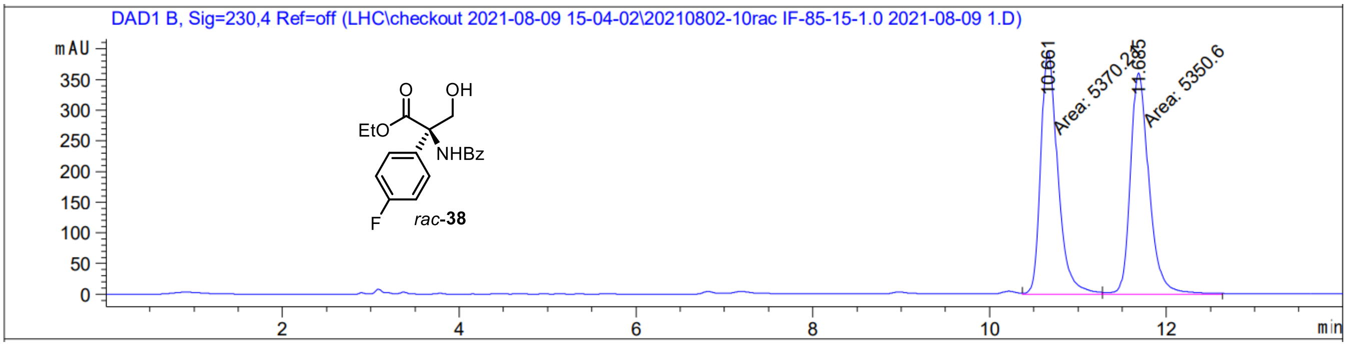

Signal 2: DAD1 B, Sig=230,4 Ref=off

Peak #	RetTime [min]	Type	Width [min]	Area [mAU*s]	Height [mAU]	Area %
1	10.661	MF	0.2252	5370.20947	397.38171	50.0915
2	11.685	FM	0.2470	5350.60010	361.02454	49.9085
Totals :				1.07208e4	758.40625	

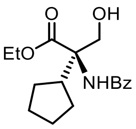

(+)-**39**

(+)-**39** was obtained as white solid (60.3 mg, 66% Yield) from the reductive desymmetrization of malonic ester **S39** (104.1 mg, 0.3 mmol) with **L11a** in toluene at 0 °C for 7 d using the general procedure B. R_f = 0.2 (hexane/EtOAc = 3:1). $[\alpha]_D^{25}$ = +12.9 (c = 1.0, $CHCl_3$).

^{1}H NMR (400 MHz, $CDCl_3$) δ 7.78 (m, 2H), 7.52 (m, 1H), 7.44 (m, 2H), 7.07 (s, 1H), 4.46 (d, J = 11.6 Hz, 1H), 4.30-4.26 (m, 3H), 4.04 (d, J = 11.6 Hz, 1H), 2.70 (m, 1H), 1.79-1.73 (m, 2H), 1.58-1.32 (m, 6H), 1.31 (t, J = 7.2 Hz, 3H).

^{13}C NMR (100 MHz, $CDCl_3$) δ 172.21, 168.08, 134.56, 131.80, 128.66, 126.94, 69.01, 65.28, 62.15, 43.67, 27.20, 27.04, 25.10, 25.08, 14.15.

IR (neat, cm^{-1}) 3400, 2953, 1724, 1646, 1519, 1228, 713.

HRMS (ESI) calcd $C_{17}H_{24}NO_4^+$ $[M+H]^+$: 306.1700. Found: 306.1699.

HPLC analysis (Chiralpak IF-3, hexane/^{i}PrOH = 85/15, 1.0 mL/min, 205 nm; t_r (minor) = 10.50 min, t_r (major) = 12.48 min) gave the isomeric composition of the product: 86% *e.e.*.

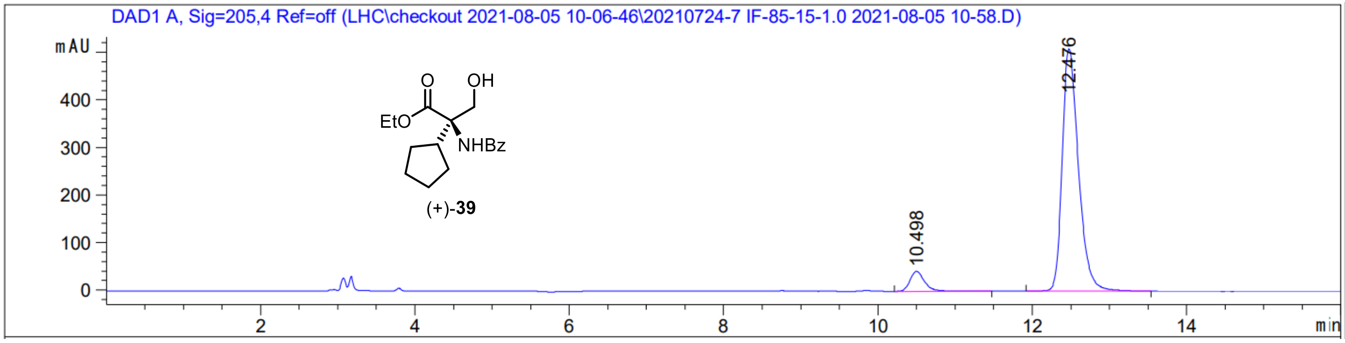

Signal 1: DAD1 A, Sig=205,4 Ref=off

Peak #	RetTime [min]	Type	Width [min]	Area [mAU*s]	Height [mAU]	Area %
1	10.498	BB	0.2010	565.57062	42.01753	7.0432
2	12.476	BB	0.2248	7464.46631	509.84152	92.9568
Totals :				8030.03693	551.85905	

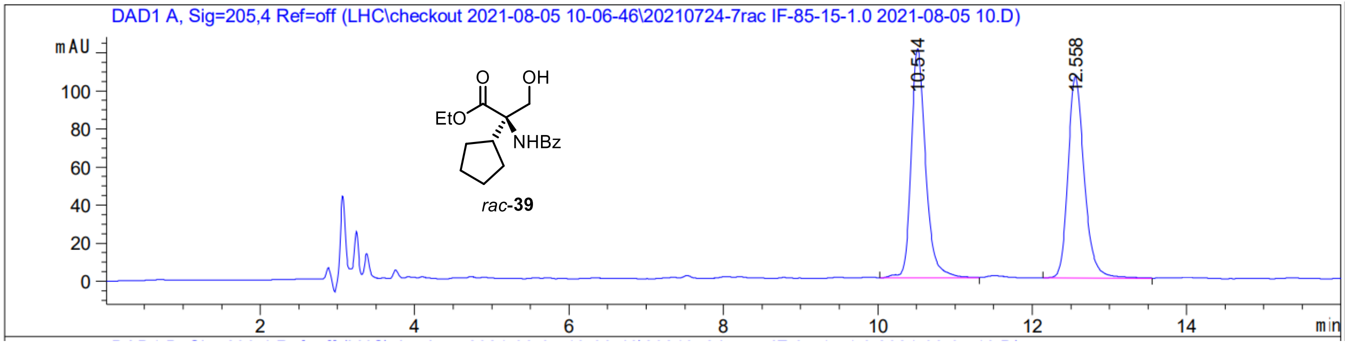

Signal 1: DAD1 A, Sig=205,4 Ref=off

Peak #	RetTime [min]	Type	Width [min]	Area [mAU*s]	Height [mAU]	Area %
1	10.514	BB	0.1986	1573.36743	120.31033	50.4167
2	12.558	BB	0.2244	1547.36218	105.93291	49.5833
Totals :				3120.72961	226.24325	

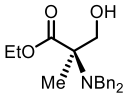

(+)-**10**

(+)-**10** was obtained as colorless oil (79.3 mg, 81% Yield) from the reductive desymmetrization of malonic ester **S10** (110.7 mg, 0.3 mmol) with **L7** in toluene at room temperature for 18 h using the general

procedure C. R_f = 0.2 (hexane/EtOAc = 5:1). $[\alpha]_D^{25}$ = +39.4 (*c* = 1.0, $CHCl_3$). The absolute stereochemistry of (+)-**10** was assigned according to literature after transesterification to corresponding methyl ester ($[\alpha]_D^{25}$ = +27.3 (*c* = 1.0, $CHCl_3$, *R* configuration) $[\alpha]_{D\ lit.}^{25}$ = –34.3 (*c* = 1.17, $CHCl_3$, *S* configuration).).[5]

^{1}H NMR (400 MHz, $CDCl_3$) δ 7.23-7.16 (m, 10H), 4.23 (q, *J* = 7.2 Hz, 2H), 3.95 (d, *J* = 14.8 Hz, 2H), 3.84 (d, *J* = 14.8 Hz, 2H), 3.78 (d, *J* = 11.2 Hz, 1H), 3.71 (d, *J* = 11.2 Hz, 1H), 2.49 (br s, 1H), 1.40 (s, 3H), 1.35 (t, *J* = 7.2 Hz, 3H).

^{13}C NMR (100 MHz, $CDCl_3$) δ 174.36, 140.77, 128.38, 128.25, 126.85, 67.98, 65.36, 60.70, 54.95, 20.91, 14.36.

IR (neat, cm^{-1}) 2928, 1721, 1454, 1206, 1028, 697.

HRMS (ESI) calcd $C_{20}H_{26}NO_3^+$ $[M+H]^+$: 328.1907. Found: 328.1908.

HPLC analysis (Chiralpak IF-3, hexane/^{i}PrOH = 95/5, 1.0 mL/min, 205 nm; t_r (minor) = 9.03 min, t_r (major) = 10.46 min) gave the isomeric composition of the product: 93% *e.e.*.

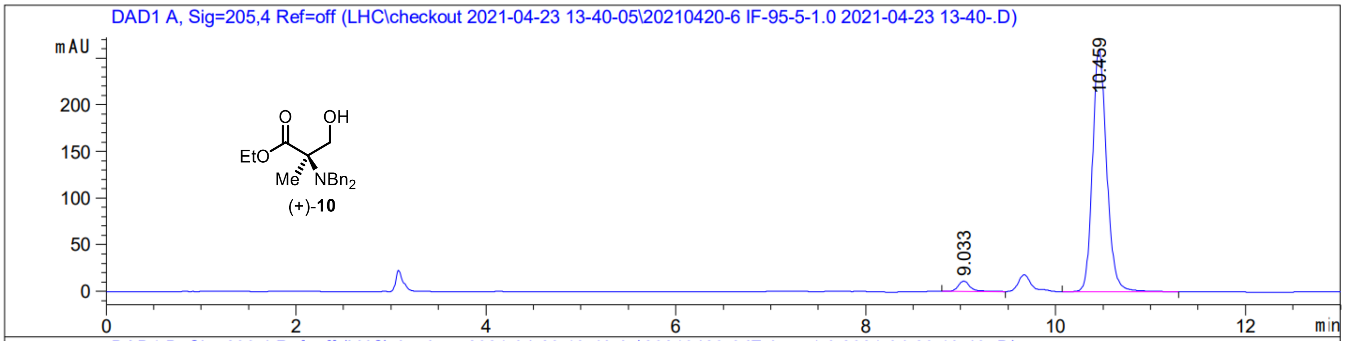

Signal 1: DAD1 A, Sig=205,4 Ref=off

Peak #	RetTime [min]	Type	Width [min]	Area [mAU*s]	Height [mAU]	Area %
1	9.033	BV	0.1370	100.20853	11.13543	3.5822
2	10.459	BB	0.1590	2697.20361	259.98105	96.4178

Totals : 2797.41214 271.11648

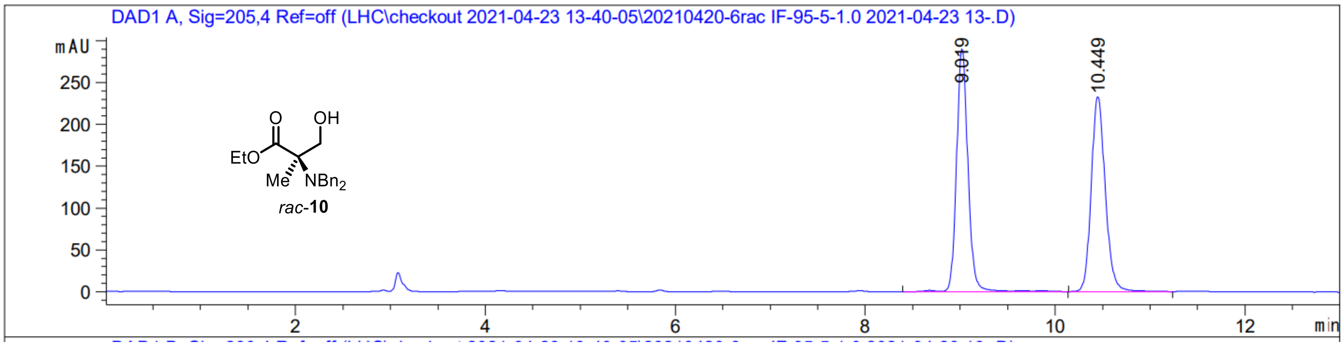

Signal 1: DAD1 A, Sig=205,4 Ref=off

Peak #	RetTime [min]	Type	Width [min]	Area [mAU*s]	Height [mAU]	Area %
1	9.019	VV R	0.1275	2445.55444	289.71313	50.5001
2	10.449	BB	0.1581	2397.12012	232.90848	49.4999
Totals :				4842.67456	522.62161	

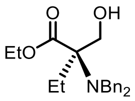

(+)-**40**

(+)-**40** was obtained as colorless oil (86.8 mg, 85% Yield) from the reductive desymmetrization of malonic ester **S40** (114.9 mg, 0.3 mmol) with **L7** in toluene at 0 °C for 48 h using the general procedure C. R_f = 0.2 (hexane/EtOAc = 4:1). $[\alpha]_D^{25}$ = +46.8 (*c* = 1.0, $CHCl_3$).

^{1}H NMR (400 MHz, $CDCl_3$) δ 7.25-7.14 (m, 10H), 4.26 (q, *J* = 7.2 Hz, 2H), 3.96 (d, *J* = 14.8 Hz, 2H), 3.87-3.76 (m, 4H), 2.50 (br s, 1H), 1.95 (m, 1H), 1.60 (m, 1H), 1.36 (t, *J* = 7.2 Hz, 3H), 0.85 (t, *J* = 7.2 Hz, 3H).

^{13}C NMR (100 MHz, $CDCl_3$) δ 173.39, 140.81, 128.37, 128.32, 126.85, 71.62, 60.46, 59.94, 54.57, 25.61, 14.49, 9.04.

IR (neat, cm^{-1}) 2970, 1721, 1454, 1228, 1027, 697.

HRMS (ESI) calcd $C_{21}H_{28}NO_3^+$ [M+H]$^+$: 342.2064. Found: 342.2061.

HPLC analysis (Chiralpak ID-3, hexane/^{i}PrOH = 95/5, 1.0 mL/min, 230 nm; t_r (minor) = 7.81 min, t_r (major) = 8.64 min) gave the isomeric composition of the product: 90% *e.e.*.

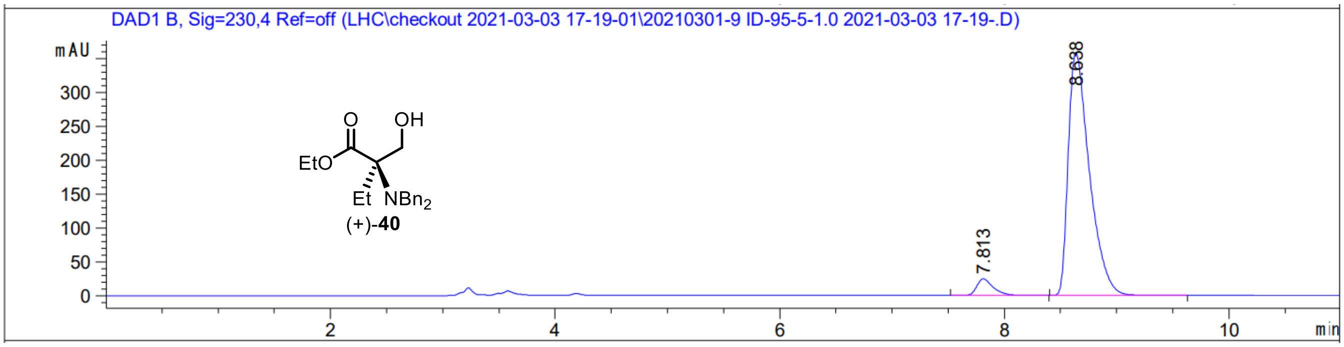

Signal 2: DAD1 B, Sig=230,4 Ref=off

Peak #	RetTime [min]	Type	Width [min]	Area [mAU*s]	Height [mAU]	Area %
1	7.813	BB	0.1539	252.76208	24.59902	5.0839
2	8.638	BB	0.1994	4719.02783	358.80902	94.9161
Totals :				4971.78992	383.40804	

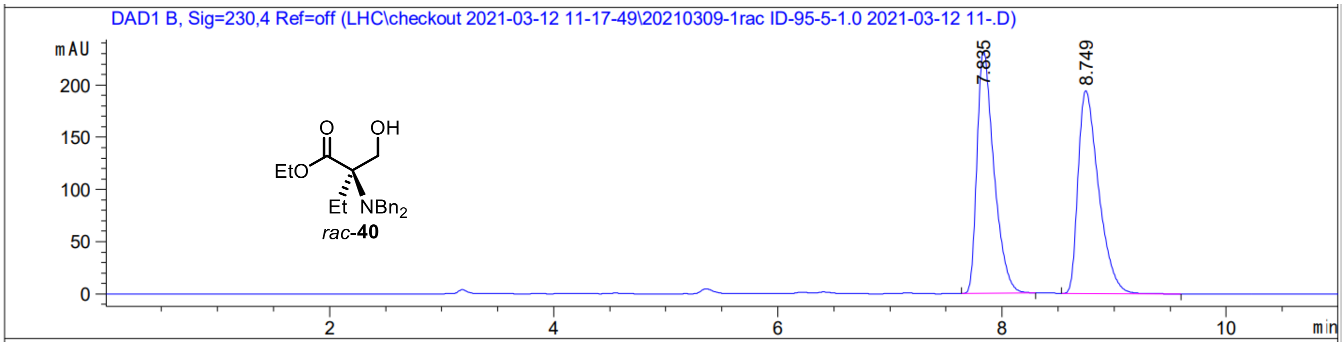

Signal 2: DAD1 B, Sig=230,4 Ref=off

Peak #	RetTime [min]	Type	Width [min]	Area [mAU*s]	Height [mAU]	Area %
1	7.835	BB	0.1594	2452.01489	231.82492	49.8051
2	8.749	BB	0.1925	2471.20532	194.08125	50.1949
Totals :				4923.22021	425.90617	

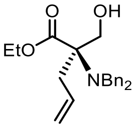

(+)-**41**

(+)-**41** was obtained as colorless oil (81.5 mg, 77% Yield) from the reductive desymmetrization of malonic ester **S41** (118.5 mg, 0.3 mmol) with **L7** in toluene at 0 °C for 48 h using the general procedure C. R_f =

0.2 (hexane/EtOAc = 4:1). $[\alpha]_D^{25}$ = +61.4 (c = 1.0, $CHCl_3$).

^{1}H NMR (400 MHz, $CDCl_3$) δ 7.24-7.17 (m, 10H), 5.73 (m, 1H), 5.12 (s, 1H), 5.09 (s, 1H), 4.24 (q, J = 7.2 Hz, 2H), 3.97 (d, J = 14.8 Hz, 2H), 3.88-3.75 (m, 4H), 2.70 (dd, J = 13.2, 6.8 Hz, 1H), 2.45-2.38 (m, 2H), 1.35 (t, J = 7.2 Hz, 3H).

^{13}C NMR (100 MHz, $CDCl_3$) δ 172.78, 140.54, 132.81, 128.39, 128.31, 126.90, 119.12, 70.86, 61.03, 60.58, 54.53, 37.32, 14.46.

IR (neat, cm^{-1}) 2925, 1721, 1454, 1212, 1028, 697.

HRMS (ESI) calcd $C_{22}H_{28}NO_3^+$ $[M+H]^+$: 354.2064. Found: 354.2063.

HPLC analysis (Chiralpak ID-3, hexane/^{i}PrOH = 95/5, 1.0 mL/min, 230 nm; t_r (minor) = 7.42 min, t_r (major) = 8.12 min) gave the isomeric composition of the product: 86% *e.e.*.

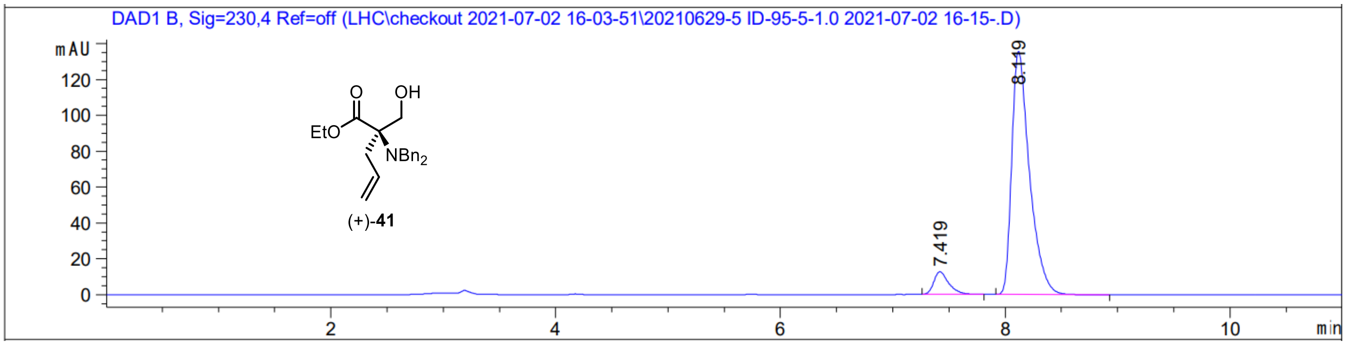

Signal 2: DAD1 B, Sig=230,4 Ref=off

Peak #	RetTime [min]	Type	Width [min]	Area [mAU*s]	Height [mAU]	Area %
1	7.419	BB	0.1363	114.15176	12.53196	7.1500
2	8.119	BB	0.1635	1482.38110	135.61621	92.8500
Totals :				1596.53287	148.14817	

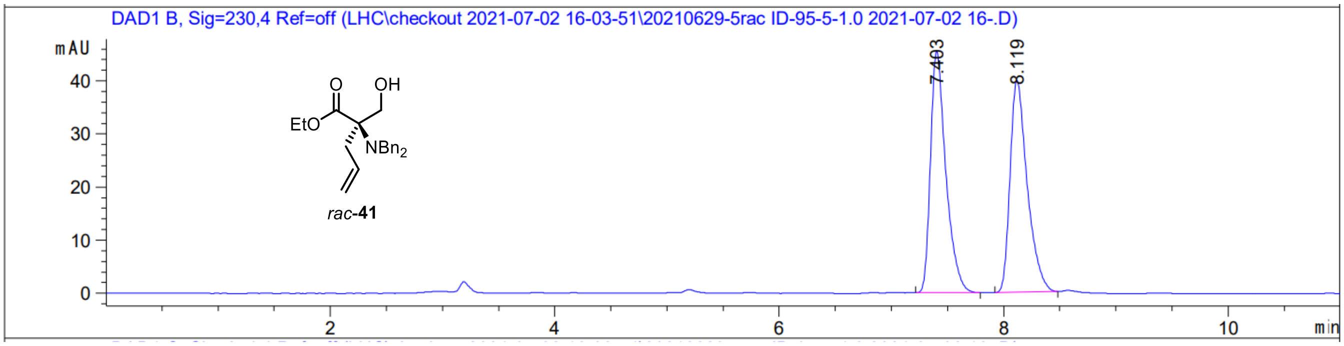

```
Signal 2: DAD1 B, Sig=230,4 Ref=off

Peak RetTime Type  Width     Area      Height     Area
  #   [min]        [min]   [mAU*s]     [mAU]        %
----|-------|----|-------|----------|----------|--------|
   1   7.403 BB    0.1405  431.08130   45.52182  50.4206
   2   8.119 BB    0.1578  423.88980   39.95563  49.5794

Totals :                   854.97110   85.47745
```

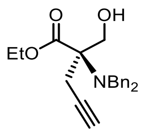

(+)-**42**

(+)-**42** was obtained as colorless oil (85.2 mg, 81% Yield) from the reductive desymmetrization of malonic ester **S42** (117.9 mg, 0.3 mmol) with **L7** in toluene at 0 °C for 48 h using the general procedure C. R_f = 0.2 (hexane/EtOAc = 4:1). $[\alpha]_D^{25}$ = +26.3 (*c* = 1.0, $CHCl_3$).

^{1}H NMR (400 MHz, $CDCl_3$) δ 7.26-7.17 (m, 10H), 4.27 (q, *J* = 7.2 Hz, 2H), 3.96-3.85 (m, 6H), 2.89 (dd, *J* = 16.4, 2.4 Hz, 1H), 2.63 (dd, *J* = 16.4, 2.4 Hz, 1H), 2.28 (dd, *J* = 8.4, 4.0 Hz, 1H), 2.08 (m, 1H), 1.36 (t, *J* = 7.2 Hz, 3H).

^{13}C NMR (100 MHz, $CDCl_3$) δ 171.80, 140.19, 128.36, 128.31, 127.02, 79.82, 71.57, 70.49, 62.38, 61.01, 54.53, 23.39, 14.40.

IR (neat, cm^{-1}) 2979, 1721, 1454, 1212, 1028, 697.

HRMS (ESI) calcd $C_{22}H_{26}NO_3^+$ [M+H]$^+$: 352.1907. Found: 352.1904.

HPLC analysis (Chiralpak ID-3, hexane/^{i}PrOH = 95/5, 1.0 mL/min, 205 nm; t_r (minor) = 10.95 min, t_r (major) = 12.28 min) gave the isomeric composition of the product: 91% *e.e.*.

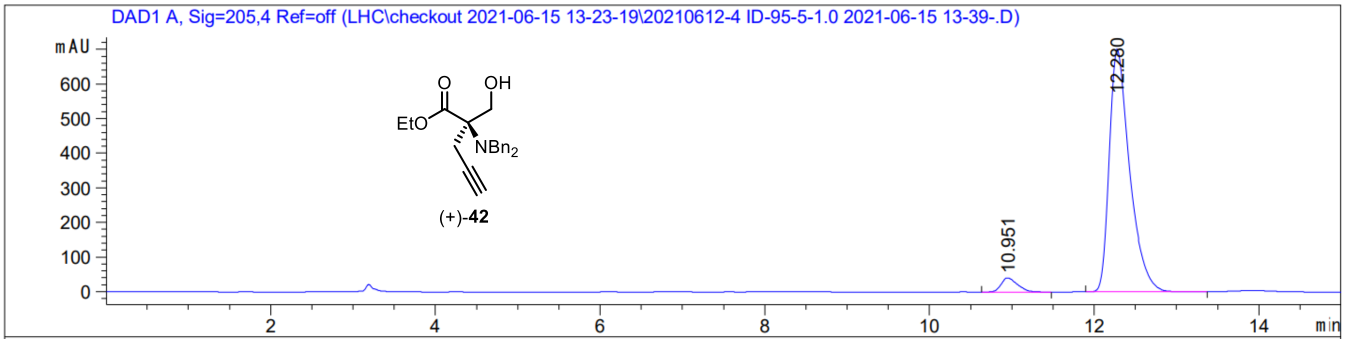

```
Signal 1: DAD1 A, Sig=205,4 Ref=off

Peak RetTime Type  Width     Area      Height     Area
  #   [min]        [min]    [mAU*s]     [mAU]       %
----|-------|----|-------|----------|----------|--------|
   1  10.951 BB    0.2066  571.03101   40.97878   4.5096
   2  12.280 BB    0.2612 1.20917e4   701.12756  95.4904

Totals :                  1.26627e4   742.10634
```

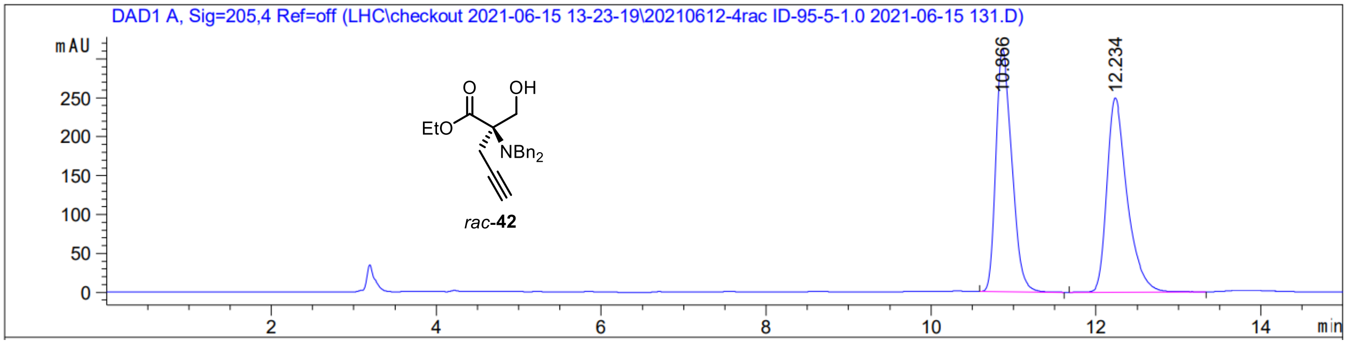


```
Signal 1: DAD1 A, Sig=205,4 Ref=off

Peak RetTime Type  Width     Area      Height     Area
  #   [min]        [min]    [mAU*s]     [mAU]       %
----|-------|----|-------|----------|----------|--------|
   1  10.866 BB    0.2103 4182.02930  312.09964  49.5243
   2  12.234 BB    0.2551 4262.36377  249.71147  50.4757

Totals :                  8444.39307  561.81111
```

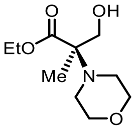

(+)-**44**

(+)-**44** was obtained as colorless oil (54.0 mg, 83% Yield) from the reductive desymmetrization of malonic ester **S44** (77.7 mg, 0.3 mmol) with **L9** in toluene at room temperature for 72 h using the general procedure C. R_f = 0.2 (hexane/EtOAc = 2:1). $[\alpha]_D^{25}$ = +21.8 (*c* = 1.0, $CHCl_3$).

^{1}H NMR (400 MHz, $CDCl_3$) δ 4.19 (m, 2H), 3.74-3.68 (m, 5H), 3.55 (d, *J* = 11.6 Hz, 1H), 2.76-2.74 (m, 2H), 2.63-2.59 (m, 2H), 1.35 (s, 3H), 1.29 (t, *J* = 7.2 Hz, 3H).

^{13}C NMR (100 MHz, $CDCl_3$) δ 172.97, 67.66, 65.63, 63.51, 60.67, 46.99, 18.60, 14.34.

IR (neat, cm^{-1}) 2961, 1721, 1242, 1112, 1046.

HRMS (ESI) calcd $C_{10}H_{20}NO_4^+$ $[M+H]^+$: 218.1387. Found: 218.1387.

HPLC analysis (Chiralpak IF-3, hexane/^{i}PrOH = 90/10, 1.0 mL/min, 205 nm; t_r (minor) = 13.60 min, t_r (major) = 17.19 min) gave the isomeric composition of the product: 92% *e.e.*.

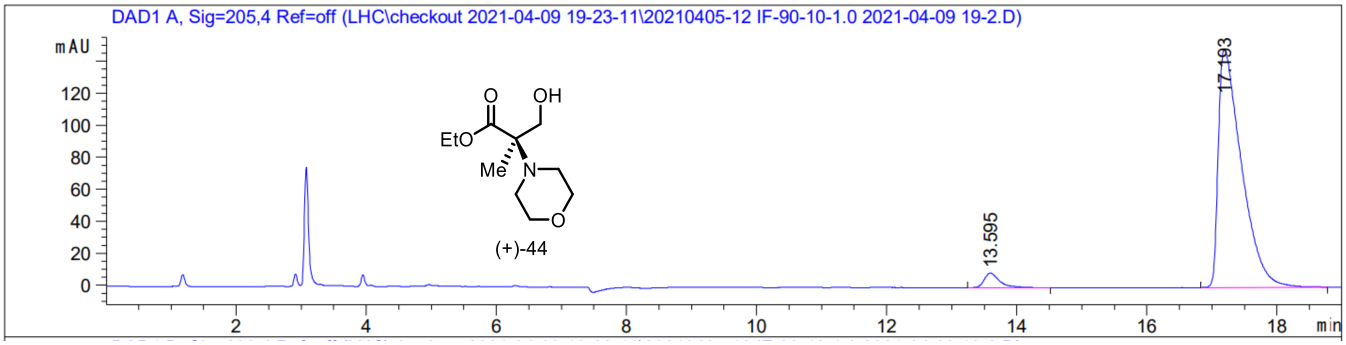

Signal 1: DAD1 A, Sig=205,4 Ref=off

Peak #	RetTime [min]	Type	Width [min]	Area [mAU*s]	Height [mAU]	Area %
1	13.595	BB	0.2588	159.24626	9.15891	4.1443
2	17.193	BB	0.3707	3683.29004	148.85684	95.8557

Totals : 3842.53630 158.01575

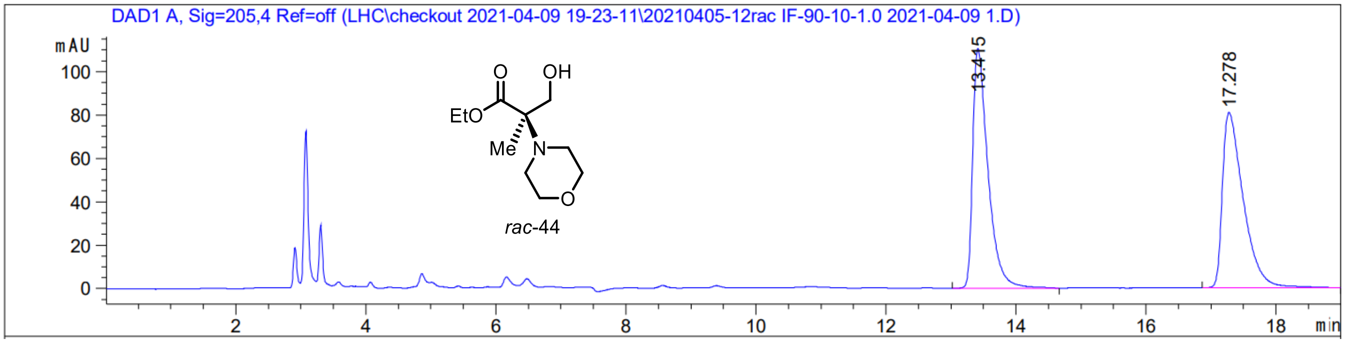

Signal 1: DAD1 A, Sig=205,4 Ref=off

Peak #	RetTime [min]	Type	Width [min]	Area [mAU*s]	Height [mAU]	Area %
1	13.415	BB	0.2466	1806.59448	110.57774	50.0669
2	17.278	BBA	0.3360	1801.76807	80.85511	49.9331

Totals : 3608.36255 191.43285

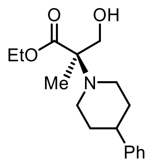

(+)-**45**

(+)-**45** was obtained as colorless oil (66.4 mg, 76% Yield) from the reductive desymmetrization of malonic ester **S45** (99.9 mg, 0.3 mmol) with **L9** in toluene at room temperature for 72 h using the general procedure C. R_f = 0.2 (hexane/EtOAc = 3:1). $[\alpha]_D^{25}$ = +13.1 (c = 1.0, $CHCl_3$).

^{1}H NMR (400 MHz, $CDCl_3$) δ 7.32-7.18 (m, 5H), 7.25 – 7.16 (m, 3H), 4.22 (m, 2H), 3.81 (d, J = 11.2 Hz, 1H), 3.56 (d, J = 11.2 Hz, 1H), 3.19 (dd, J = 11.2, 2.4 Hz, 1H), 3.02 (dd, J = 11.2, 2.4 Hz, 1H), 2.70-2.53 (m, 3H), 2.30 (td, J = 11.6, 2.4 Hz, 1H), 1.89 (m, 2H), 1.79-1.63 (m, 2H), 1.41 (s, 3H), 1.34 (t, J = 7.2 Hz, 3H).

^{13}C NMR (100 MHz, $CDCl_3$) δ 173.53, 146.00, 128.39, 126.75, 126.17, 65.86, 63.86, 60.49, 47.59, 46.97, 42.71, 34.48, 34.18, 19.30, 14.39.

IR (neat, cm^{-1}) 3430, 2926, 1723, 1229, 1044, 699.

HRMS (ESI) calcd $C_{17}H_{26}NO_3^+$ $[M+H]^+$: 292.1907. Found: 292.1906.

HPLC analysis (Chiralpak ID-3, hexane/^{i}PrOH = 90/10, 1.0 mL/min, 205 nm; t_r (minor) = 6.76 min, t_r (major) = 7.83 min) gave the isomeric composition of the product: 84% *e.e.*.

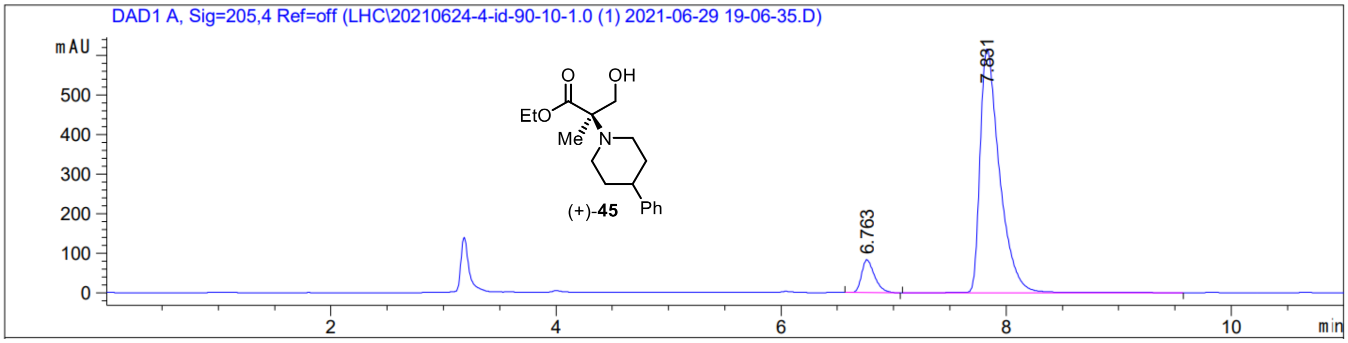

Signal 1: DAD1 A, Sig=205,4 Ref=off

Peak #	RetTime [min]	Type	Width [min]	Area [mAU*s]	Height [mAU]	Area %
1	6.763	BB	0.1209	662.88550	83.24586	8.1379
2	7.831	BB	0.1815	7482.75684	616.97070	91.8621
Totals :				8145.64233	700.21656	

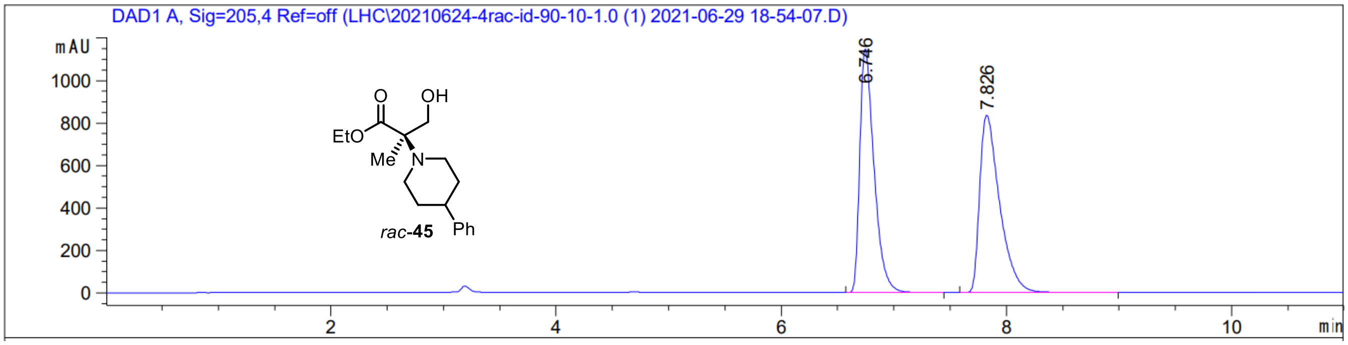

Signal 1: DAD1 A, Sig=205,4 Ref=off

Peak #	RetTime [min]	Type	Width [min]	Area [mAU*s]	Height [mAU]	Area %
1	6.746	BB	0.1324	1.00622e4	1146.00745	49.2567
2	7.826	BB	0.1869	1.03659e4	834.85437	50.7433

Totals : 2.04281e4 1980.86182

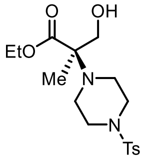

(–)-**46**

(–)-**46** was obtained as colorless oil (92.2 mg, 83% Yield) from the reductive desymmetrization of malonic ester **S46** (123.6 mg, 0.3 mmol) with **L9** in toluene at room temperature for 72 h using the general procedure C. R_f = 0.2 (hexane/EtOAc = 1:1). $[\alpha]_D^{25}$ = –1.2 (*c* = 1.0, $CHCl_3$).

^{1}H NMR (400 MHz, $CDCl_3$) δ 7.61 (d, *J* = 8.0 Hz, 2H), 7.32 (d, *J* = 8.0 Hz, 2H), 4.17 (q, *J* = 7.2 Hz, 2H), 3.63 (d, *J* = 11.2 Hz, 1H), 3.53 (d, *J* = 11.2 Hz, 1H), 2.97 (m, 4H), 2.79 (m, 2H), 2.69 (m, 2H), 2.43 (s, 3H), 1.29-1.25 (m, 6H).

^{13}C NMR (100 MHz, $CDCl_3$) δ 172.85, 143.79, 132.13, 129.67, 127.79, 65.57, 63.96, 60.89, 46.77, 45.86, 21.47, 18.55, 14.30.

IR (neat, cm^{-1}) 2983, 1723, 1163, 937, 731, 549.

HRMS (ESI) calcd $C_{17}H_{27}N_2O_5S^+$ $[M+H]^+$: 371.1635. Found: 371.1632.

HPLC analysis (Chiralpak IF-3, hexane/^{i}PrOH = 70/30, 0.7 mL/min, 230 nm; t_r (minor) = 25.30 min, t_r (major) = 29.34 min) gave the isomeric composition of the product: 89% *e.e.*.

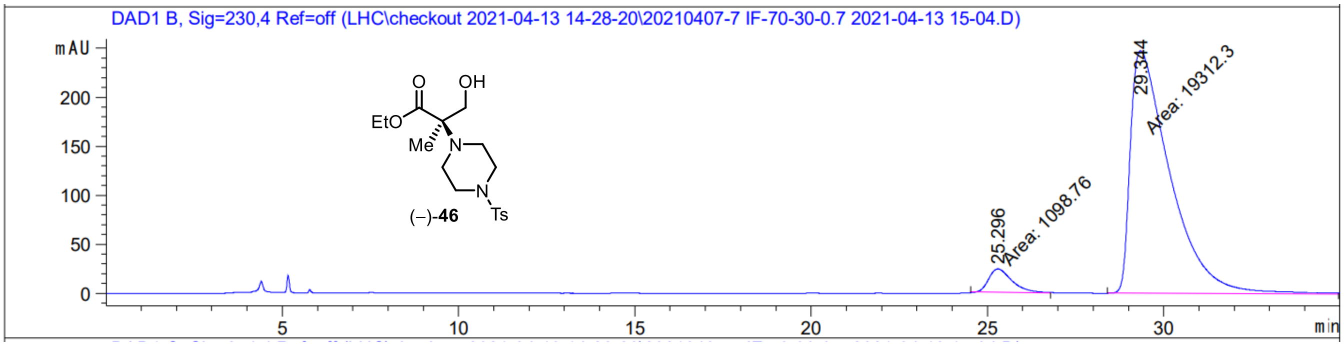

Signal 2: DAD1 B, Sig=230,4 Ref=off

Peak #	RetTime [min]	Type	Width [min]	Area [mAU*s]	Height [mAU]	Area %
1	25.296	MM	0.7665	1098.75818	23.89090	5.3831
2	29.344	MM	1.3023	1.93123e4	247.16141	94.6169

Totals : 2.04111e4 271.05231

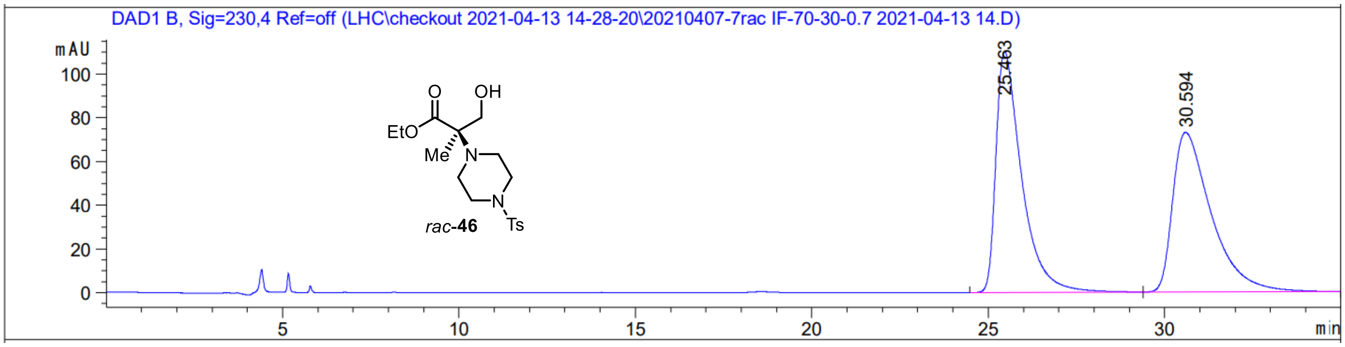

Signal 2: DAD1 B, Sig=230,4 Ref=off

Peak #	RetTime [min]	Type	Width [min]	Area [mAU*s]	Height [mAU]	Area %
1	25.463	BB	0.7535	5589.27441	110.24074	50.1505
2	30.594	BBA	1.1217	5555.73389	73.02016	49.8495

Totals : 1.11450e4 183.26089

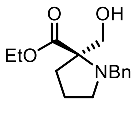

(+)-**11**

(+)-**11** was obtained as colorless oil (66.4 mg, 84% Yield) from the reductive desymmetrization of malonic ester **S11** (79.5 mg, 0.3 mmol) with **L9** in toluene at room temperature for 18 h using the general procedure

C. R_f = 0.2 (hexane/EtOAc = 5:1). $[\alpha]_D^{25}$ = +33.4 (*c* = 1.0, $CHCl_3$).

^{1}H NMR (400 MHz, $CDCl_3$) δ 7.34-7.25 (m, 5H), 4.24 (m, 2H), 3.84 (d, *J* = 13.2 Hz, 1H), 3.72 (s, 2H), 3.50 (d, *J* = 13.2 Hz, 1H), 3.00 (td, *J* = 8.4, 2.4 Hz, 1H), 2.87-2.80 (m, 2H), 2.24-2.04 (m, 2H), 1.87-1.72 (m, 2H), 1.33 (t, *J* = 7.2 Hz, 3H).

^{13}C NMR (100 MHz, $CDCl_3$) δ 174.15, 139.33, 128.40, 127.07, 70.94, 61.83, 60.44, 53.39, 52.29, 33.22, 22.49, 14.39.

IR (neat, cm^{-1}) 2977, 1721, 1179, 1026, 699.

HRMS (ESI) calcd $C_{15}H_{22}NO_3^+$ [M+H]$^+$: 264.1594. Found: 264.1595.

HPLC analysis (Chiralpak IF-3, hexane/^{i}PrOH = 95/5, 1.0 mL/min, 205 nm; t_r (minor) = 8.66 min, t_r (major) = 10.45 min) gave the isomeric composition of the product: 99% *e.e.*.

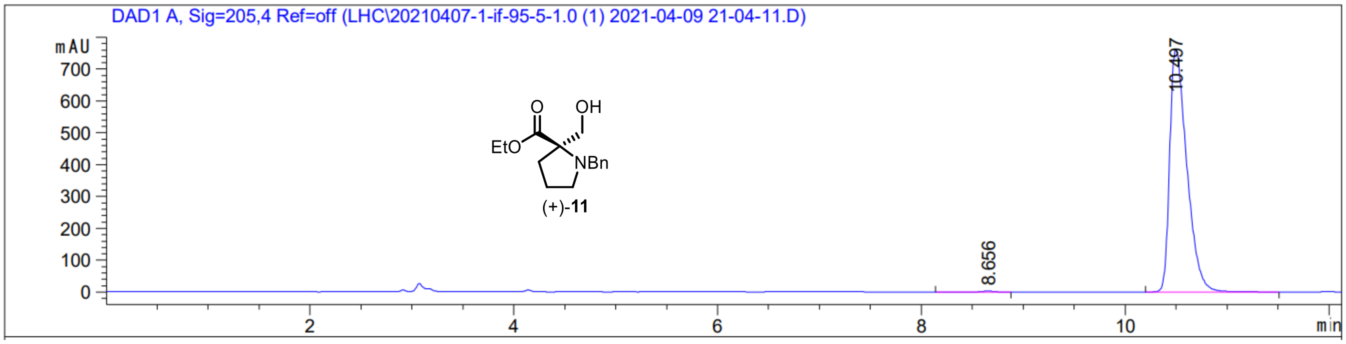

Signal 1: DAD1 A, Sig=205,4 Ref=off

Peak #	RetTime [min]	Type	Width [min]	Area [mAU*s]	Height [mAU]	Area %
1	8.656	BB	0.1424	32.36530	3.30008	0.3620
2	10.497	BB	0.1782	8908.04688	763.13696	99.6380

Totals : 8940.41217 766.43705

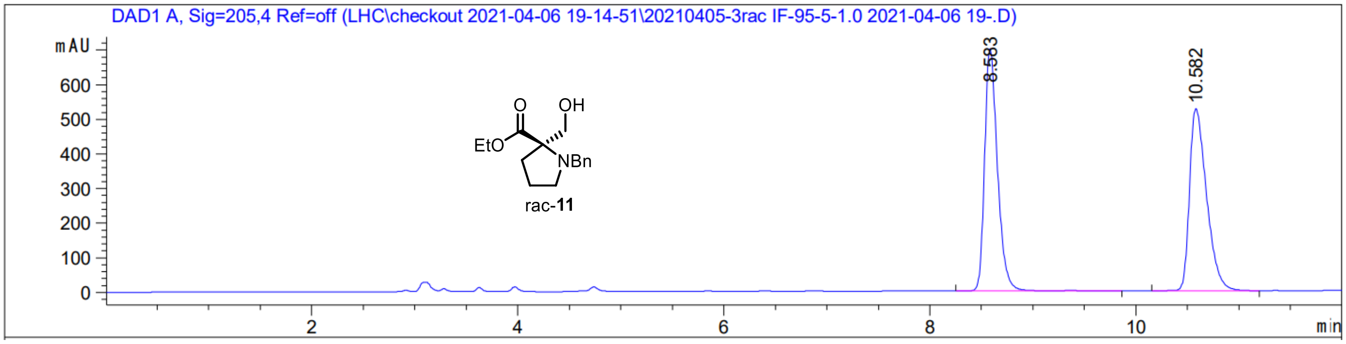

Signal 1: DAD1 A, Sig=205,4 Ref=off

Peak #	RetTime [min]	Type	Width [min]	Area [mAU*s]	Height [mAU]	Area %
1	8.583	BV R	0.1312	6100.93555	700.39795	50.1142
2	10.582	BB	0.1764	6073.11963	527.00159	49.8858

Totals : 1.21741e4 1227.39954

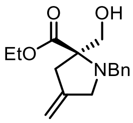

(+)-**47**

(+)-**47** was obtained as colorless oil (61.4 mg, 74% Yield) from the reductive desymmetrization of malonic ester **S47** (95.1 mg, 0.3 mmol) with **L9** in toluene at room temperature for 24 h using the general procedure C. R_f = 0.2 (hexane/EtOAc = 5:1). $[\alpha]_D^{25}$ = +80.6 (c = 1.0, $CHCl_3$).

^{1}H NMR (400 MHz, $CDCl_3$) δ 7.35-7.26 (m, 5H), 4.90 (s, 1H), 4.85 (s, 1H), 4.25 (m, 2H), 3.91 (d, J = 13.2 Hz, 1H), 3.82 (d, J = 11.2 Hz, 1H), 3.75 (d, J = 11.2 Hz, 1H), 3.58 (d, J = 13.2 Hz, 1H), 3.49 (d, J = 13.2 Hz, 2H), 2.94 (dd, J = 16.8, 2.0 Hz, 1H), 2.74-2.70 (m, 2H), 1.32 (t, J = 7.2 Hz, 3H).

^{13}C NMR (100 MHz, $CDCl_3$) δ 173.35, 144.63, 138.69, 128.50, 128.37, 127.25, 105.57, 71.49, 60.99, 60.66, 57.38, 53.46, 39.16, 14.39.

IR (neat, cm^{-1}) 3435, 2934, 1721, 1179, 1036, 698.

HRMS (ESI) calcd $C_{16}H_{22}NO_3^+$ $[M+H]^+$: 276.1594. Found: 276.1595.

HPLC analysis (Chiralpak ID-3, hexane/^{i}PrOH = 95/5, 1.0 mL/min, 205 nm; t_r (minor) = 7.54 min, t_r (major) = 8.64 min) gave the isomeric composition of the product: 95% *e.e.*.

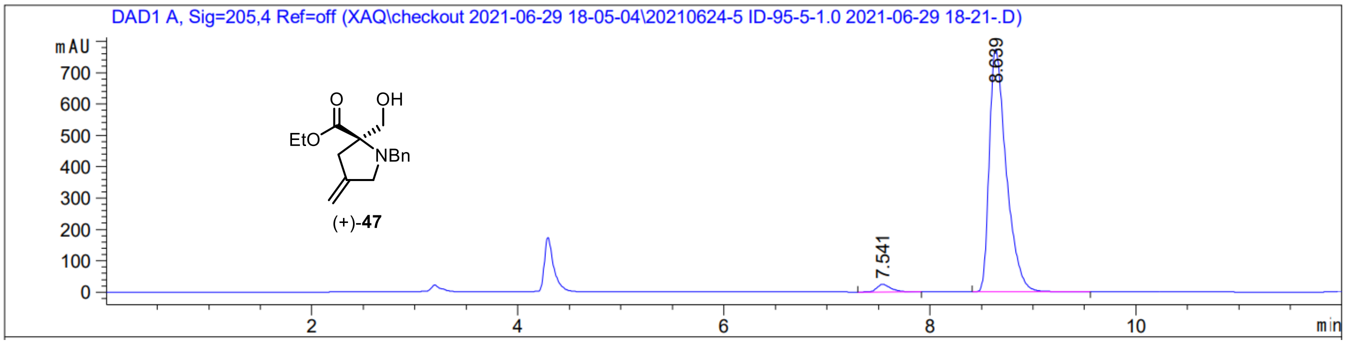

Signal 1: DAD1 A, Sig=205,4 Ref=off

Peak #	RetTime [min]	Type	Width [min]	Area [mAU*s]	Height [mAU]	Area %
1	7.541	BB	0.1409	235.81845	24.81211	2.5908
2	8.639	BB	0.1717	8866.31445	773.58954	97.4092

Totals : 9102.13290 798.40165

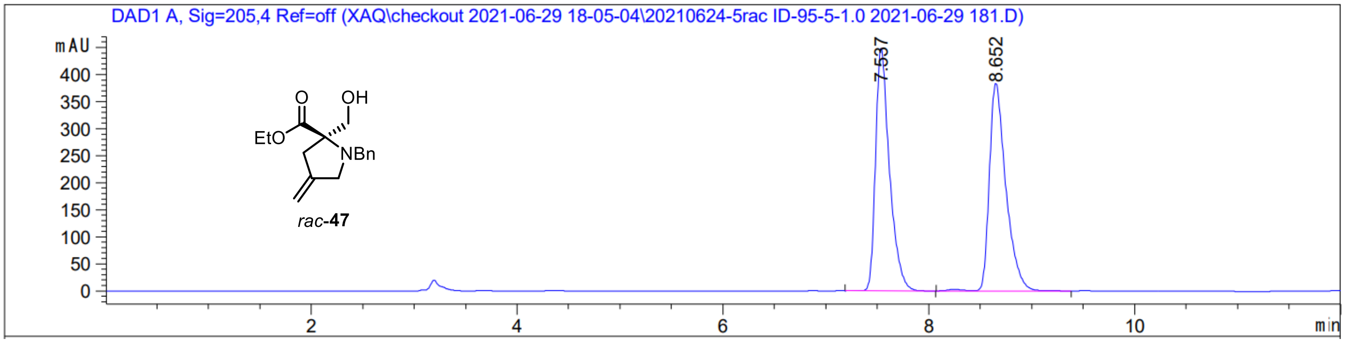

Signal 1: DAD1 A, Sig=205,4 Ref=off

Peak #	RetTime [min]	Type	Width [min]	Area [mAU*s]	Height [mAU]	Area %
1	7.537	BB	0.1396	4214.03662	448.55856	49.5721
2	8.652	VB R	0.1654	4286.79004	383.83865	50.4279

Totals : 8500.82666 832.39722

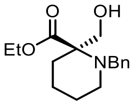

(+)-**48**

(+)-**48** was obtained as colorless oil (73.2 mg, 88% Yield) from the reductive desymmetrization of malonic ester **S48** (95.7 mg, 0.3 mmol) with **L7** in toluene at 0 °C for 24 h using the general procedure C. R_f = 0.2 (hexane/EtOAc = 5:1). $[\alpha]_D^{25}$ = +14.0 (*c* = 1.0, $CHCl_3$).

^{1}H NMR (400 MHz, $CDCl_3$) δ 7.32-7.24 (m, 5H), 4.24 (q, *J* = 7.2 Hz, 2H), 3.98 (d, *J* = 14.4 Hz, 1H), 3.92 (d, *J* = 11.2 Hz, 1H), 3.63 (d, *J* = 11.2 Hz, 1H), 3.52 (d, *J* = 14.4 Hz, 1H), 2.87-2.74 (m, 3H), 2.00-1.92 (m, 2H), 1.61 (m, 2H), 1.50-1.42 (m, 2H), 1.32 (t, *J* = 7.2 Hz, 3H).

^{13}C NMR (100 MHz, $CDCl_3$) δ 173.84, 139.50, 128.40, 128.24, 126.89, 66.19, 63.41, 60.40, 54.96, 47.49, 31.86, 25.46, 20.69, 14.37.

IR (neat, cm^{-1}) 2933, 1721, 1224, 1053, 698.

HRMS (ESI) calcd $C_{16}H_{24}NO_3^+$ [M+H]$^+$: 278.1751. Found: 278.1753.

HPLC analysis (Chiralpak ID-3, hexane/^{i}PrOH = 95/5, 1.0 mL/min, 205 nm; t_r (major) = 7.37 min, t_r (minor) = 6.61 min) gave the isomeric composition of the product: 90% *e.e.*.

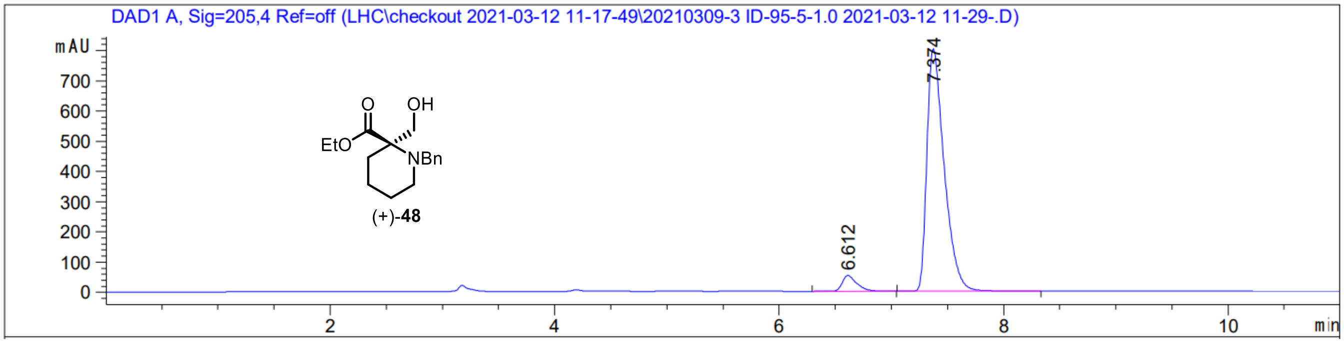

Signal 1: DAD1 A, Sig=205,4 Ref=off

Peak #	RetTime [min]	Type	Width [min]	Area [mAU*s]	Height [mAU]	Area %
1	6.612	BB	0.1289	465.44598	52.78571	5.1624
2	7.374	BB	0.1604	8550.61621	801.69220	94.8376

Totals : 9016.06219 854.47791

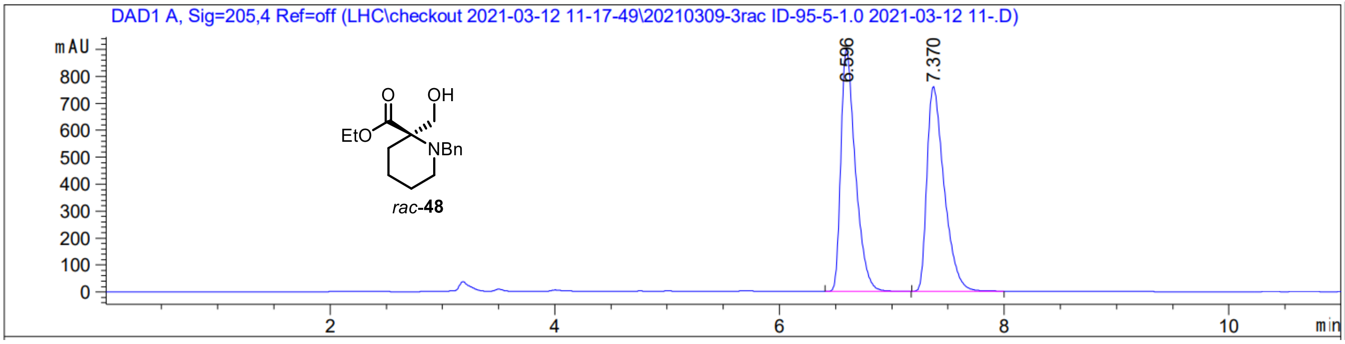

Signal 1: DAD1 A, Sig=205,4 Ref=off

Peak #	RetTime [min]	Type	Width [min]	Area [mAU*s]	Height [mAU]	Area %
1	6.596	BB	0.1322	8043.62988	900.87677	49.9115
2	7.370	BB	0.1601	8072.16211	758.86816	50.0885

Totals : 1.61158e4 1659.74493

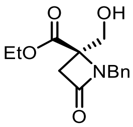

(–)-**49**

(–)-**49** was obtained as colorless oil (49.5 mg, 63% Yield) from the reductive desymmetrization of malonic ester **S49** (91.5 mg, 0.3 mmol) with **L9** in toluene at room temperature for 24 h using the general procedure C. R_f = 0.2 (Hexane/EtOAc = 2:1). $[\alpha]_D^{25}$ = –11.1 (*c* = 1.0, $CHCl_3$).

^{1}H NMR (400 MHz, $CDCl_3$) δ 7.34-7.31 (m, 5H), 4.67 (d, *J* = 15.2 Hz, 1H), 4.30 (d, *J* = 15.2 Hz, 1H), 4.15-4.01 (m, 2H), 3.72 (s, 2H), 3.09 (d, *J* = 14.8 Hz, 1H), 3.03 (d, *J* = 14.8 Hz, 1H), 1.88 (br s, 1H), 1.21 (t, *J* = 7.2 Hz, 3H).

^{13}C NMR (100 MHz, $CDCl_3$) δ 170.87, 166.00, 136.07, 128.93, 128.57, 128.08, 63.15, 62.67, 61.86, 45.19, 44.24, 13.93.

IR (neat, cm^{-1}) 2916, 1729, 1392, 1041, 716.

HRMS (ESI) calcd $C_{14}H_{18}NO_4^+$ $[M+H]^+$: 264.1230. Found: 264.1228.

HPLC analysis (Chiralpak IF-3, hexane/^{i}PrOH = 80/20, 0.8 mL/min, 205 nm; t_r (major) = 9.85 min, t_r (minor) = 10.68 min) gave the isomeric composition of the product: 83% *e.e.*.

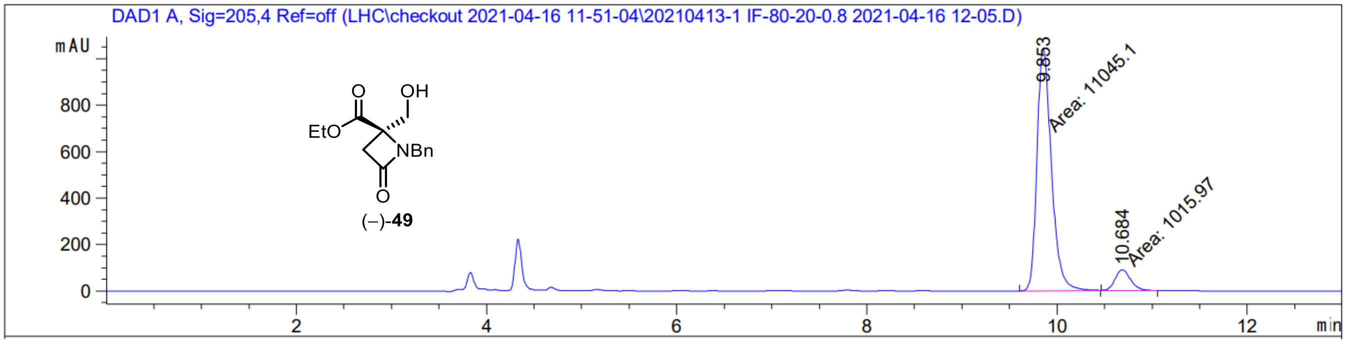

Signal 1: DAD1 A, Sig=205,4 Ref=off

Peak #	RetTime [min]	Type	Width [min]	Area [mAU*s]	Height [mAU]	Area %
1	9.853	MM	0.1763	1.10451e4	1043.98975	91.5765
2	10.684	MM	0.1885	1015.96625	89.83105	8.4235
Totals :				1.20611e4	1133.82079	

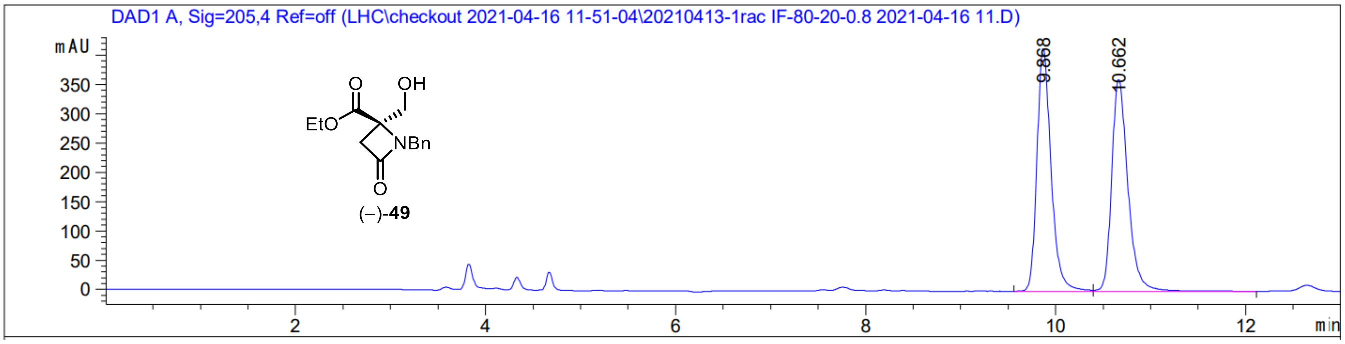

Signal 1: DAD1 A, Sig=205,4 Ref=off

Peak #	RetTime [min]	Type	Width [min]	Area [mAU*s]	Height [mAU]	Area %
1	9.868	BV	0.1569	4290.18359	414.08258	50.1157
2	10.662	VB	0.1796	4270.38135	362.16962	49.8843

Totals : 8560.56494 776.25220

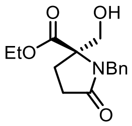

(+)-**50**

(+)-**50** was obtained as colorless oil (65.7 mg, 79% Yield) from the reductive desymmetrization of malonic ester **S50** (95.7 mg, 0.3 mmol) with **L9** in toluene at 50 °C for 48 h using the general procedure C. R_f = 0.2 (Hexane/EtOAc = 2:1). $[\alpha]_D^{25}$ = +24.8 (*c* = 1.0, $CHCl_3$).

^{1}H NMR (400 MHz, $CDCl_3$) δ 7.34-7.25 (m, 5H), 4.81 (d, *J* = 15.2 Hz, 1H), 4.20 (d, *J* = 15.2 Hz, 1H), 4.08 (m, 1H), 3.91 (m, 1H), 3.74 (d, *J* = 12.4 Hz, 1H), 3.70 (d, *J* = 12.4 Hz, 1H), 2.53-2.50 (m, 2H), 2.17-2.12 (m, 2H), 1.84 (br s, 1H), 1.18 (t, *J* = 7.2 Hz, 3H).

^{13}C NMR (100 MHz, $CDCl_3$) δ 176.61, 172.45, 137.52, 128.74, 128.16, 127.76, 70.16, 63.84, 61.73, 44.86, 29.23, 26.17, 13.92.

IR (neat, cm^{-1}) 3383, 2938, 1730, 1667, 1050, 704.

HRMS (ESI) calcd $C_{15}H_{20}NO_4^+$ [M+H]$^+$: 278.1387. Found: 278.1388.

HPLC analysis (Chiralpak IF-3, hexane/^{i}PrOH = 90/10, 1.0 mL/min, 205 nm; t_r (major) = 22.33 min, t_r (minor) = 24.15 min) gave the isomeric composition of the product: 89% *e.e.*.

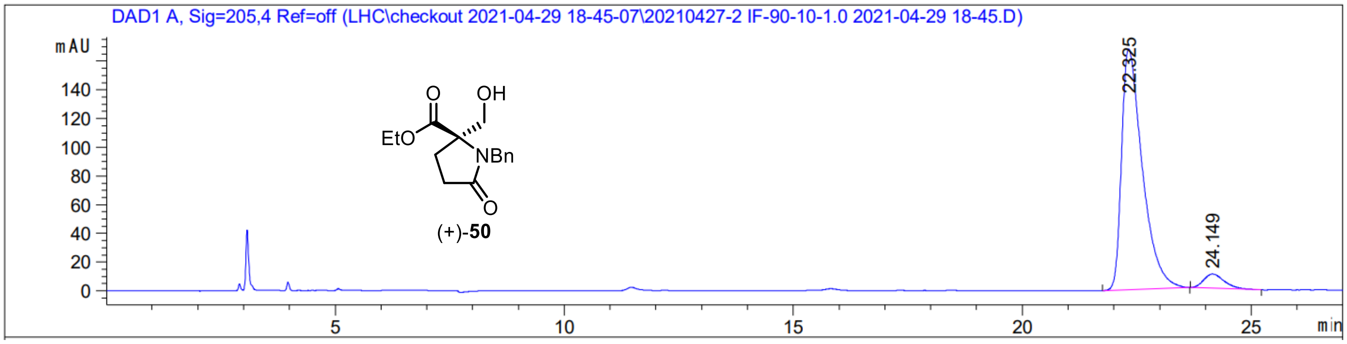

Signal 1: DAD1 A, Sig=205,4 Ref=off

Peak #	RetTime [min]	Type	Width [min]	Area [mAU*s]	Height [mAU]	Area %
1	22.325	BB	0.4728	5269.92285	167.62117	94.4086
2	24.149	BB	0.4780	312.11166	9.89574	5.5914
Totals :				5582.03452	177.51691	

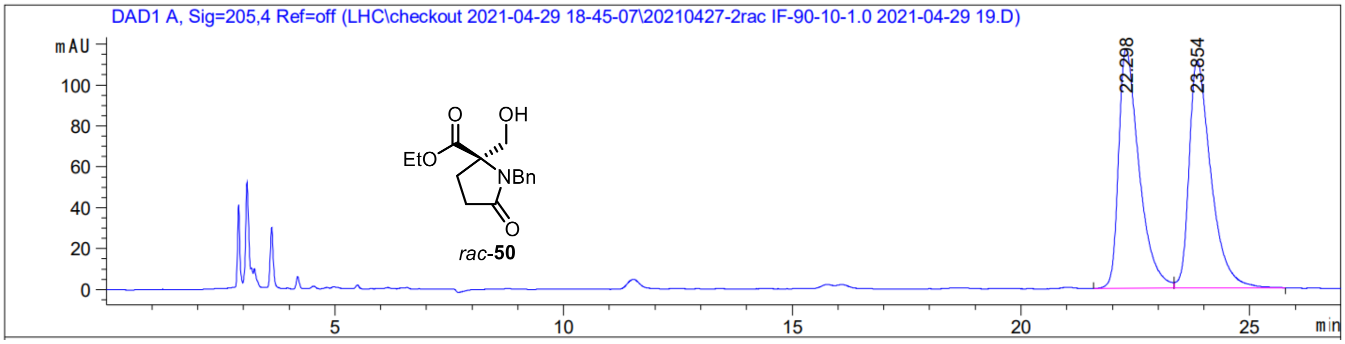

Signal 1: DAD1 A, Sig=205,4 Ref=off

Peak #	RetTime [min]	Type	Width [min]	Area [mAU*s]	Height [mAU]	Area %
1	22.298	BV	0.4678	3648.74316	117.01311	49.6075
2	23.854	VB	0.5030	3706.47656	111.16956	50.3925
Totals :				7355.21973	228.18267	

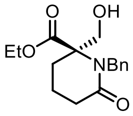

(+)-**51**

(+)-**51** was obtained as colorless oil (65.4 mg, 75% Yield) from the reductive desymmetrization of malonic ester **S51** (99.9 mg, 0.3 mmol) with **L9** in toluene at 50 °C for 60 h using the general procedure C. R_f =

0.2 (hexane/EtOAc = 1:1). $[\alpha]_D^{25}$ = +93.4 (c = 1.0, $CHCl_3$).

^{1}H NMR (400 MHz, $CDCl_3$) δ 7.33-7.24 (m, 5H), 5.37 (d, J = 16.0 Hz, 1H), 4.22-4.15 (m, 2H), 3.81 (d, J = 16.0 Hz, 1H), 3.73 (s, 2H), 2.63-2.46 (m, 2H), 2.25 (m, 1H), 2.06 (d, J = 14 Hz, 1H), 1.83 – 1.80 (m, 3H), 1.26 (t, J = 7.2 Hz, 3H).

^{13}C NMR (100 MHz, $CDCl_3$) δ 172.70, 172.37, 138.60, 128.88, 127.41, 127.12, 69.85, 64.60, 61.94, 48.25, 32.23, 30.12, 17.35, 14.13.

IR (neat, cm^{-1}) 3383, 2953, 1732, 1618, 1069, 724.

HRMS (ESI) calcd $C_{16}H_{22}NO_4^+$ $[M+H]^+$: 292.1543. Found: 292.1549.

HPLC analysis (Chiralpak ID-3, hexane/^{i}PrOH = 70/30, 0.7 mL/min, 205 nm; t_r (major) = 15.82 min, t_r (minor) = 18.27 min) gave the isomeric composition of the product: 88% *e.e.*.

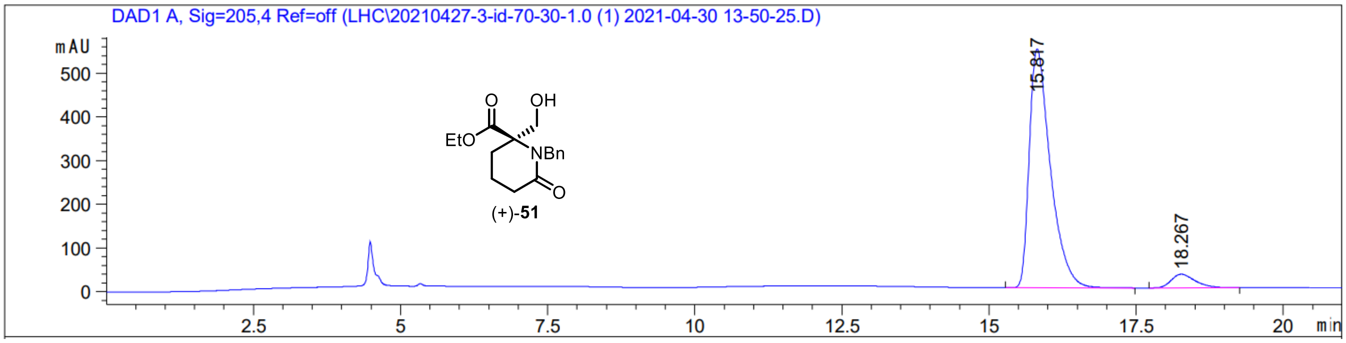

Signal 1: DAD1 A, Sig=205,4 Ref=off

Peak #	RetTime [min]	Type	Width [min]	Area [mAU*s]	Height [mAU]	Area %
1	15.817	BB	0.3778	1.36670e4	546.51880	93.8584
2	18.267	BB	0.4145	894.30200	31.38239	6.1416
Totals :				1.45613e4	577.90118	

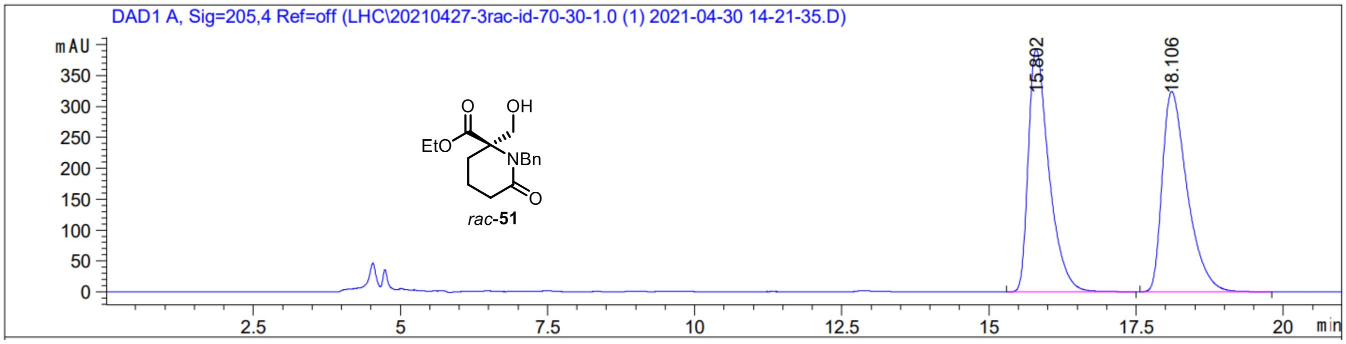

```
Signal 1: DAD1 A, Sig=205,4 Ref=off

Peak RetTime Type  Width     Area      Height     Area
  #   [min]        [min]   [mAU*s]     [mAU]       %
----|-------|----|-------|----------|----------|--------|
   1  15.802 BB    0.3661 9555.64648  392.38715  49.9537
   2  18.106 BB    0.4491 9573.36230  323.64963  50.0463

Totals :                  1.91290e4   716.03677
```

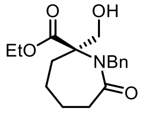

(+)-**52**

(+)-**52** was obtained as colorless oil (71.4 mg, 77% Yield) from the reductive desymmetrization of malonic ester **S52** (104.1 mg, 0.3 mmol) with **L9** in toluene at room temperature for 96 h using the general procedure C. R_f = 0.2 (hexane/EtOAc = 1:1). $[\alpha]_D^{25}$ = +15.0 (*c* = 1.0, $CHCl_3$).

^{1}H NMR (400 MHz, $CDCl_3$) δ 7.30-7.19 (m, 5H), 5.05 (d, *J* = 16.4 Hz, 1H), 4.43 (d, *J* = 16.4 Hz, 1H), 4.20-4.12 (m, 2H), 3.90 (d, *J* = 12.0 Hz, 1H), 3.77 (d, *J* = 12.0 Hz, 1H), 2.76 (m, 1H), 2.53 (m, 1H), 2.31-1.71 (m, 7H), 1.22 (t, *J* = 7.2 Hz, 3H).

^{13}C NMR (100 MHz, $CDCl_3$) δ 176.73, 172.81, 139.18, 128.49, 126.84, 126.67, 70.30, 67.00, 61.88, 49.31, 36.36, 32.80, 22.73, 21.57, 13.98.

IR (neat, cm^{-1}) 3383, 2928, 1742, 1612, 1175, 717.

HRMS (ESI) calcd $C_{17}H_{24}NO_4^+$ $[M+H]^+$: 306.1700. Found: 306.1708.

HPLC analysis (Chiralpak IF-3, hexane/^{i}PrOH = 90/10, 1.0 mL/min, 205 nm; t_r (major) = 22.74 min, t_r (minor) = 24.62 min) gave the isomeric composition of the product: 87% *e.e.*.

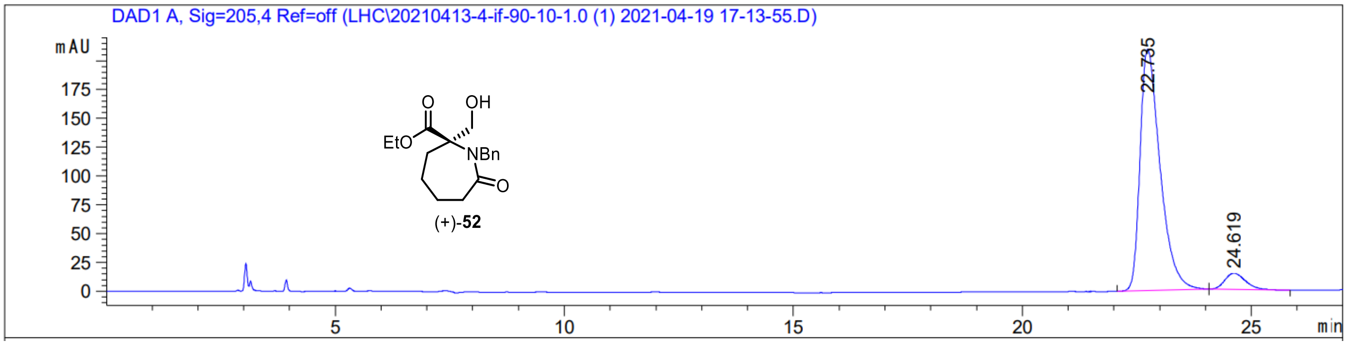

Signal 1: DAD1 A, Sig=205,4 Ref=off

Peak #	RetTime [min]	Type	Width [min]	Area [mAU*s]	Height [mAU]	Area %
1	22.735	BB	0.4623	6362.27490	209.48628	93.4348
2	24.619	BB	0.4766	447.04248	14.14957	6.5652
Totals :				6809.31738	223.63586	

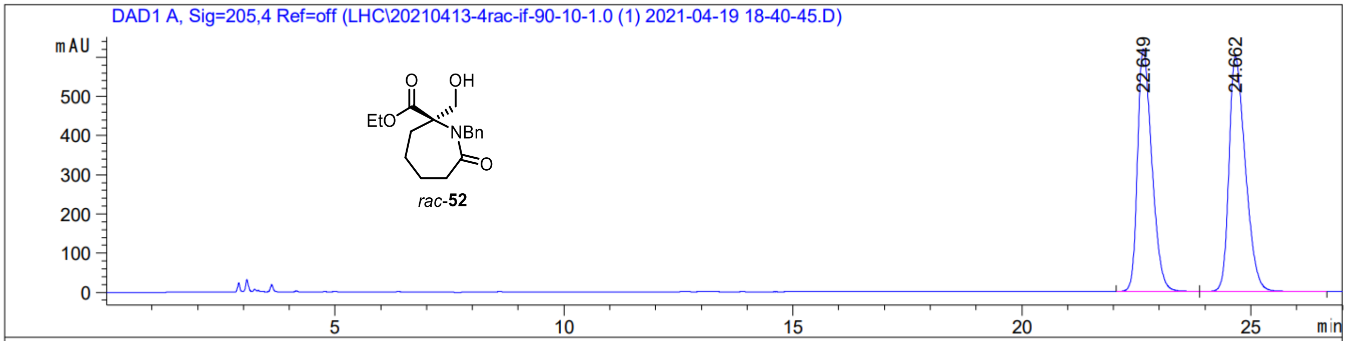

Signal 1: DAD1 A, Sig=205,4 Ref=off

Peak #	RetTime [min]	Type	Width [min]	Area [mAU*s]	Height [mAU]	Area %
1	22.649	BB	0.3343	1.34987e4	619.19879	47.4732
2	24.662	BB	0.3827	1.49356e4	599.44678	52.5268
Totals :				2.84343e4	1218.64557	

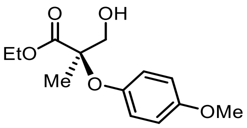

(–)-**12**

(–)-**12** was obtained as colorless oil (58.5 mg, 77% Yield) from the reductive desymmetrization of malonic ester **S12** (88.8 mg, 0.3 mmol) with **L9** in toluene at 0 °C for 16 h using the general procedure D. R_f = 0.2 (hexane/EtOAc = 3:1). $[\alpha]_D^{25}$ = –31.1 (*c* = 1.0, $CHCl_3$).

^{1}H NMR (400 MHz, $CDCl_3$) δ 6.91 (d, *J* = 8.8 Hz, 2H), 6.74 (d, *J* = 8.8 Hz, 2H), 4.25 (m, 2H), 3.84 (m, 2H), 3.75 (s, 3H), 2.52 (br s, 1H), 1.42 (s, 3H), 1.28 (t, *J* = 7.2 Hz, 3H).

^{13}C NMR (100 MHz, $CDCl_3$) δ 172.61, 155.57, 148.09, 122.19, 114.15, 82.70, 68.01, 61.55, 55.47, 18.74, 14.06.

IR (neat, cm^{-1}) 3470, 2937, 1731, 1504, 1211, 1033, 843.

HRMS (ESI) calcd $C_{13}H_{18}NaO_5^+$ $[M+Na]^+$: 277.1046. Found: 277.1046.

HPLC analysis (Chiralpak IF-3, hexane/^{i}PrOH = 90/10, 1.0 mL/min, 230 nm; t_r (minor) = 11.21 min, t_r (major) = 14.00 min) gave the isomeric composition of the product: 90% *e.e.*.

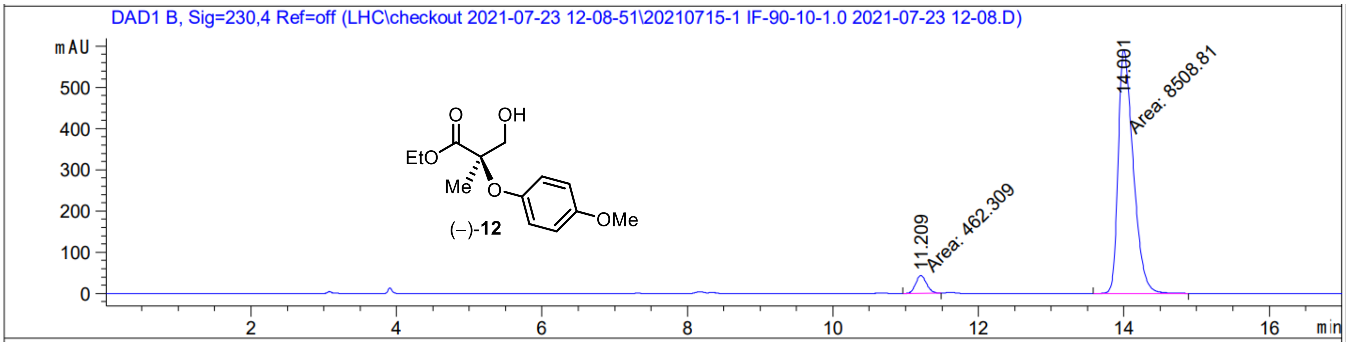


```
Signal 2: DAD1 B, Sig=230,4 Ref=off

Peak RetTime Type  Width     Area      Height     Area
  #   [min]        [min]   [mAU*s]     [mAU]       %
----|-------|----|-------|----------|----------|--------|
   1  11.209 MM    0.1750  462.30927   44.03387   5.1533
   2  14.001 MM    0.2403 8508.80859  590.23315  94.8467

Totals :                  8971.11786  634.26702
```

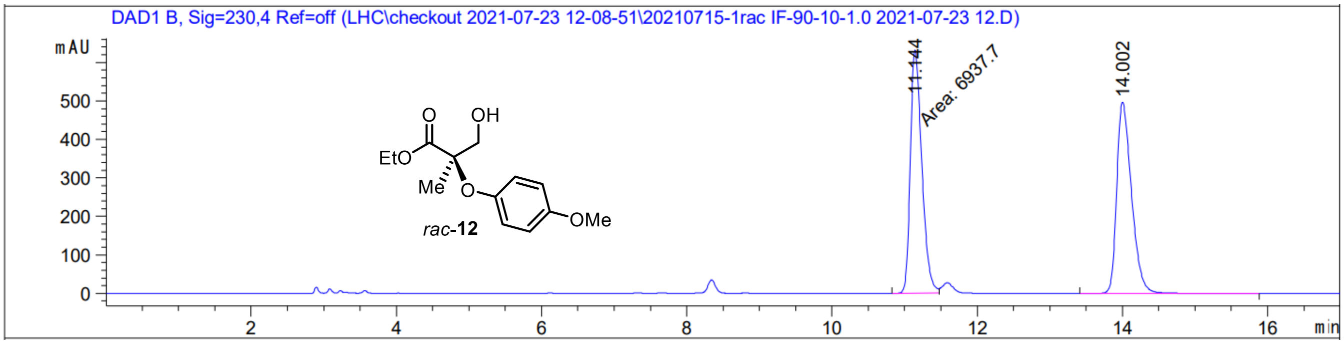


```
Signal 2: DAD1 B, Sig=230,4 Ref=off

Peak RetTime Type  Width     Area      Height     Area
  #   [min]        [min]   [mAU*s]     [mAU]       %
----|-------|----|-------|----------|----------|--------|
   1  11.144 MM    0.1830 6937.70117  632.00909  49.6838
   2  14.002 BB    0.2150 7025.99512  496.73145  50.3162

Totals :                  1.39637e4  1128.74054
```

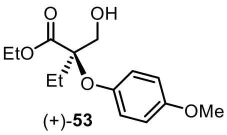

(+)-**53** was obtained as colorless oil (58.6 mg, 73% Yield) from the reductive desymmetrization of malonic ester **S53** (93.0 mg, 0.3 mmol) with **L9** in toluene at 0 °C for 48 h using the general procedure D. R_f = 0.2 (hexane/EtOAc = 2:1). $[\alpha]_D^{25}$ = +1.6 (c = 1.0, $CHCl_3$).

^{1}H NMR (400 MHz, $CDCl_3$) δ 6.92 (d, J = 8.8 Hz, 2H), 6.76 (d, J = 8.8 Hz, 2H), 4.24 (m, 2H), 3.88 (s, 2H), 3.74 (s, 3H), 2.27 (br s, 1H), 1.94 (m, 2H), 1.26 (t, J = 7.2 Hz, 3H), 0.93 (t, J = 7.6 Hz, 3H).

^{13}C NMR (100 MHz, $CDCl_3$) δ 172.30, 155.41, 148.11, 121.82, 114.19, 85.39, 63.60, 61.34, 55.43, 25.90, 14.06, 7.70.

IR (neat, cm^{-1}) 3475, 2932, 1728, 1505, 1210, 1033, 844.

HRMS (ESI) calcd $C_{14}H_{20}NaO_5^+$ $[M+Na]^+$: 291.1203. Found: 291.1205.

HPLC analysis (Chiralpak IF-3, hexane/^{i}PrOH = 90/10, 1.0 mL/min, 230 nm; t_r (minor) = 11.18 min, t_r (major) = 11.76 min) gave the isomeric composition of the product: 99% *e.e.*.

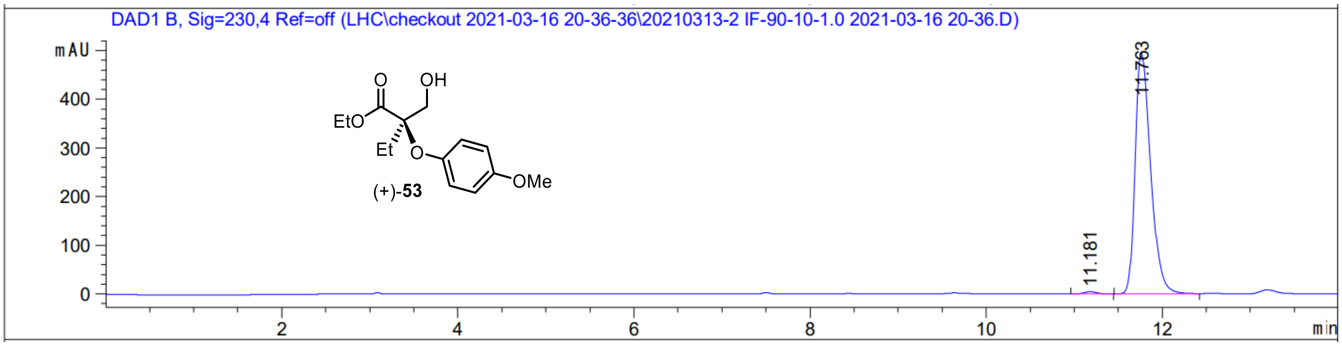

Signal 2: DAD1 B, Sig=230,4 Ref=off

Peak #	RetTime [min]	Type	Width [min]	Area [mAU*s]	Height [mAU]	Area %
1	11.181	BB	0.1598	47.62820	4.63795	0.7802
2	11.763	BB	0.1864	6056.62988	496.37155	99.2198

Totals : 6104.25808 501.00950

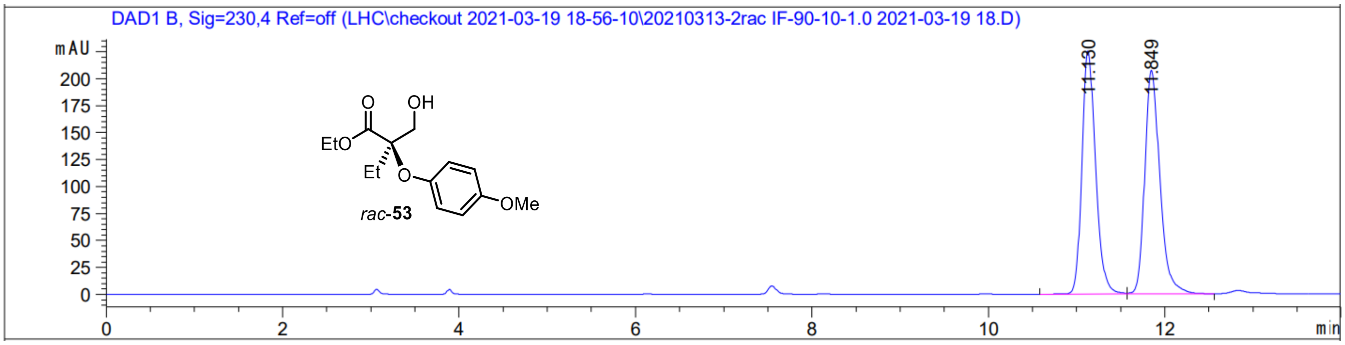

Signal 2: DAD1 B, Sig=230,4 Ref=off

Peak #	RetTime [min]	Type	Width [min]	Area [mAU*s]	Height [mAU]	Area %
1	11.130	BV	0.1659	2431.92578	225.44341	49.4570
2	11.849	VB	0.1837	2485.32568	207.48729	50.5430
Totals :				4917.25146	432.93069	

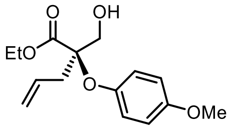

(+)-**54**

(+)-**54** was obtained as colorless oil (53.9 mg, 64% Yield) from the reductive desymmetrization of malonic ester **S54** (96.6 mg, 0.3 mmol) with **L9** in toluene at 0 °C for 72 h using the general procedure D. R_f = 0.2 (hexane/EtOAc = 3:1). $[\alpha]_D^{25}$ = +4.3 (*c* = 1.0, $CHCl_3$).

^{1}H NMR (400 MHz, $CDCl_3$) δ 6.93 (dd, *J* = 6.8, 2.4 Hz, 2H), 6.78 (dd, *J* = 6.8, 2.4 Hz, 2H), 5.79 (m, 1H), 5.16-5.11 (m, 2H), 4.24 (m, 2H), 3.90 (s, 2H), 3.75 (s, 3H), 2.71 (m, 2H), 2.17 (br s, 1H), 1.27 (m, 3H).

^{13}C NMR (100 MHz, $CDCl_3$) δ 171.78, 155.59, 148.08, 131.49, 121.91, 119.39, 114.26, 84.45, 64.05, 61.50, 55.49, 37.52, 14.10.

IR (neat, cm^{-1}) 3460, 2917, 1730, 1504, 1207, 1031, 843.

HRMS (ESI) calcd $C_{15}H_{20}NaO_5^+$ $[M+Na]^+$: 303.1203. Found: 303.1199.

HPLC analysis (Chiralpak IF-3, hexane/^{i}PrOH = 90/10, 1.0 mL/min, 230 nm; t_r (minor) = 10.74 min, t_r (major) = 11.40 min) gave the isomeric composition of the product: 96% *e.e.*.

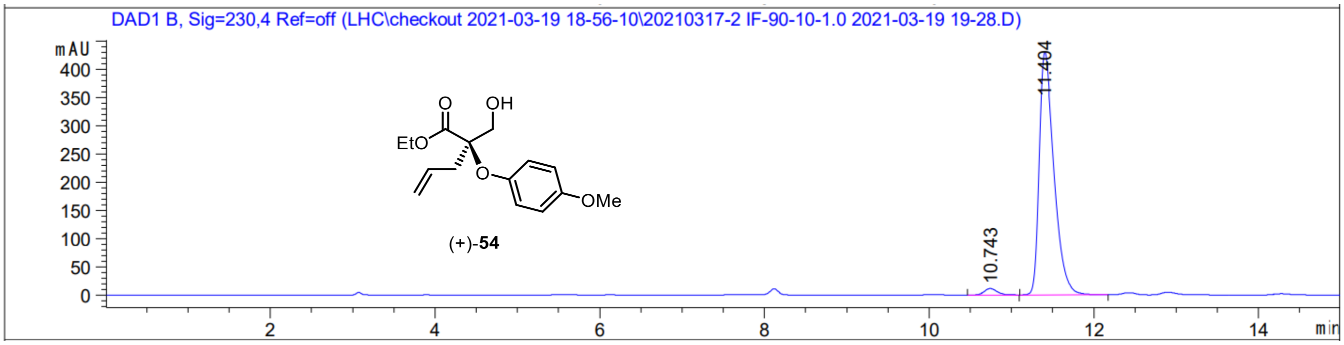


```
Signal 2: DAD1 B, Sig=230,4 Ref=off
```

Peak #	RetTime [min]	Type	Width [min]	Area [mAU*s]	Height [mAU]	Area %
1	10.743	BB	0.1648	122.55660	11.45557	2.2407
2	11.404	BB	0.1887	5347.11670	431.04926	97.7593
Totals :				5469.67330	442.50483	

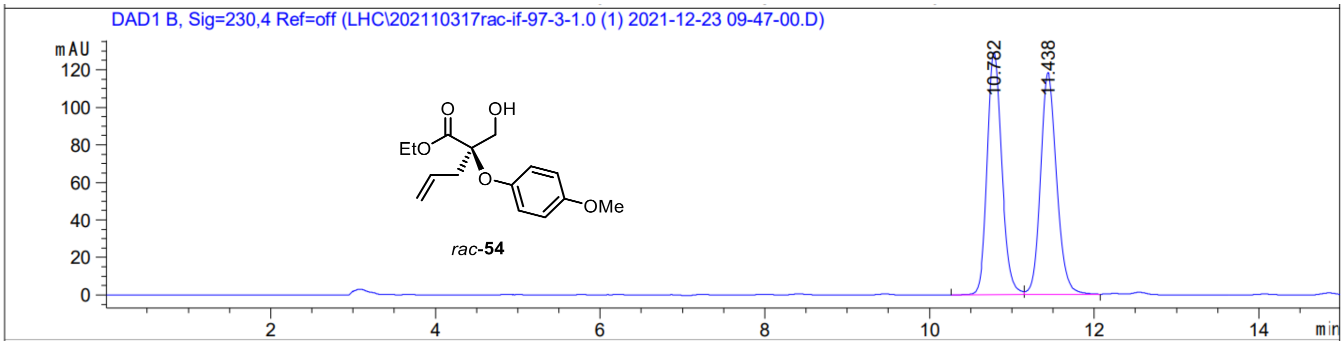


```
Signal 2: DAD1 B, Sig=230,4 Ref=off
```

Peak #	RetTime [min]	Type	Width [min]	Area [mAU*s]	Height [mAU]	Area %
1	10.782	BV	0.1896	1590.54846	129.23941	49.7044
2	11.438	VB	0.2085	1609.46570	118.47380	50.2956
Totals :				3200.01416	247.71321	

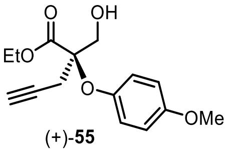

(+)-**55**

(+)-**55** was obtained as colorless oil (59.1 mg, 71% Yield) from the reductive desymmetrization of malonic ester **S55** (96.0 mg, 0.3 mmol) with **L9** in toluene at 0 °C for 48 h using the general procedure D. R_f = 0.2 (hexane/EtOAc = 3:1). $[\alpha]_D^{25}$ = +18.4 (c = 1.0, $CHCl_3$).

^{1}H NMR (400 MHz, $CDCl_3$) δ 6.99 (d, *J* = 8.8 Hz, 2H), 6.78 (d, *J* = 8.8 Hz, 2H), 4.26 (t, *J* = 7.2 Hz, 2H), 4.06 (m, 2H), 3.76 (s, 3H), 2.85 (m, 2H), 2.24 (br s, 1H), 2.09 (t, *J* = 2.8 Hz, 1H), 1.28 (t, *J* = 7.2 Hz, 3H).

^{13}C NMR (100 MHz, $CDCl_3$) δ 170.59, 156.09, 147.54, 122.69, 114.30, 83.82, 78.33, 71.90, 64.03, 61.83, 55.48, 22.96, 14.02.

IR (neat, cm^{-1}) 3288, 2915, 1732, 1504, 1205, 1034, 845.

HRMS (ESI) calcd $C_{15}H_{18}NaO_5^+$ $[M+Na]^+$: 301.1046. Found: 301.1044.

HPLC analysis (Chiralpak IF-3, hexane/^{i}PrOH = 90/10, 1.0 mL/min, 230 nm; t_r (major) = 12.61 min, t_r (minor) = 14.02 min) gave the isomeric composition of the product: 96% *e.e.*.

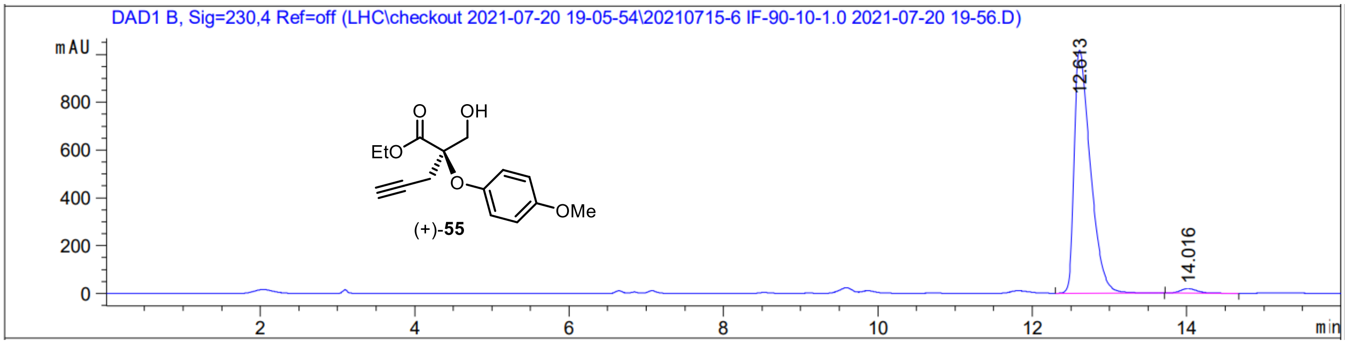


```
Signal 2: DAD1 B, Sig=230,4 Ref=off
```

Peak #	RetTime [min]	Type	Width [min]	Area [mAU*s]	Height [mAU]	Area %
1	12.613	BB	0.2211	1.49125e4	1016.97412	98.1218
2	14.016	BB	0.2302	285.45532	19.11911	1.8782
Totals :				1.51980e4	1036.09323	

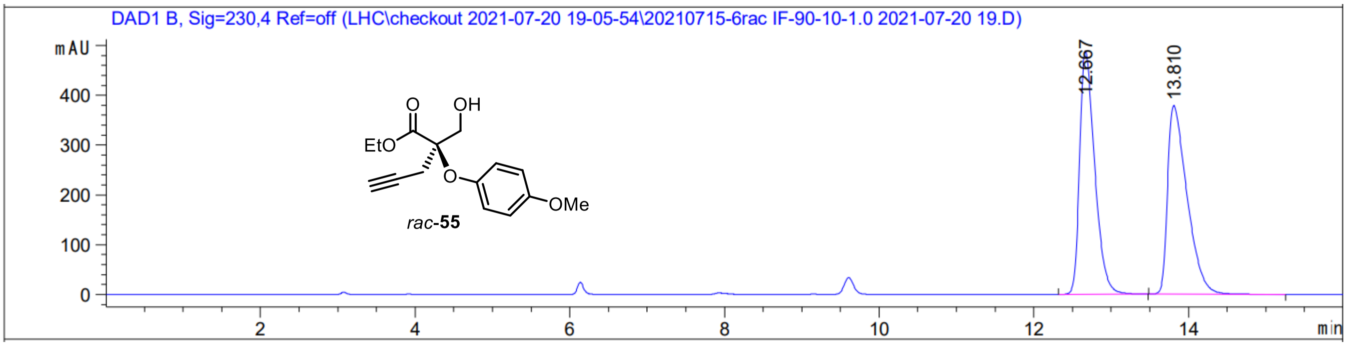

Signal 2: DAD1 B, Sig=230,4 Ref=off

Peak #	RetTime [min]	Type	Width [min]	Area [mAU*s]	Height [mAU]	Area %
1	12.667	BB	0.2064	6632.70996	488.61401	50.0433
2	13.810	BB	0.2637	6621.23389	379.27399	49.9567

Totals : 1.32539e4 867.88800

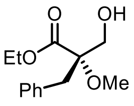

(+)-**13**

(+)-**13** was obtained as colorless oil (54.3 mg, 76% Yield) from the reductive desymmetrization of malonic ester **S13** (84.0 mg, 0.3 mmol) with **L9** in hexane at room temperature for 24 h using the general procedure D. R_f = 0.2 (hexane/EtOAc = 4:1). $[\alpha]_D^{25}$ = +5.4 (*c* = 1.0, $CHCl_3$).

^{1}H NMR (400 MHz, $CDCl_3$) δ 7.29-7.21 (m, 5H), 4.18 (q, *J* = 7.2 Hz, 2H), 3.81 (d, *J* = 12.0 Hz, 1H), 3.74 (d, *J* = 12.0 Hz, 1H), 3.43 (s, 3H), 3.15 (d, *J* = 13.6 Hz, 1H), 3.09 (d, *J* = 13.6 Hz, 1H), 2.13 (br s, 1H), 1.22 (t, *J* = 7.2 Hz, 3H).

^{13}C NMR (100 MHz, $CDCl_3$) δ 171.94, 135.23, 130.18, 128.17, 126.82, 83.49, 62.29, 61.18, 52.54, 38.73, 14.07.

IR (neat, cm^{-1}) 3450, 2937, 1729, 1195, 1028, 700.

HRMS (ESI) calcd $C_{13}H_{18}NaO_4^+$ $[M+Na]^+$: 261.1097. Found: 261.1099.

HPLC analysis (Chiralpak IF-3, hexane/^{i}PrOH = 90/10, 1.0 mL/min, 205 nm; t_r (minor) = 8.07 min, t_r (major) = 9.68 min) gave the isomeric composition of the product: 96% *e.e.*.

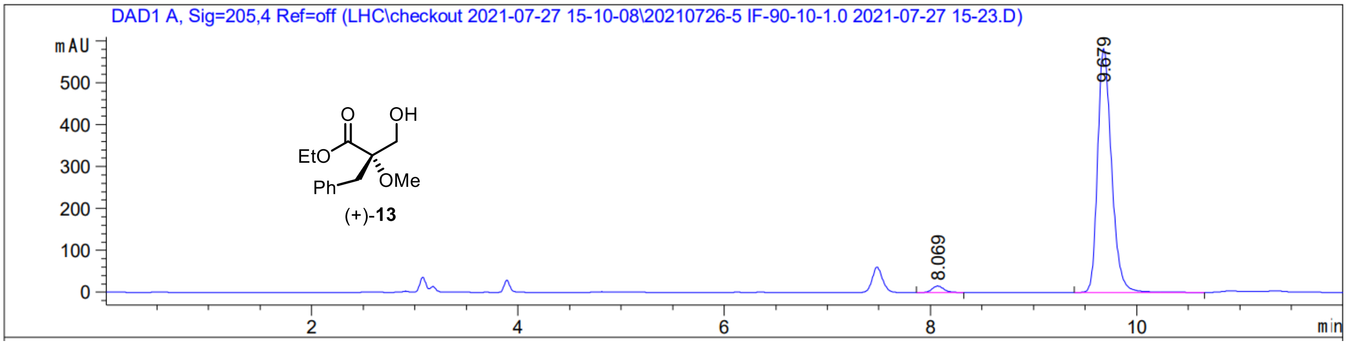

Signal 1: DAD1 A, Sig=205,4 Ref=off

Peak #	RetTime [min]	Type	Width [min]	Area [mAU*s]	Height [mAU]	Area %
1	8.069	BB	0.1184	119.66702	15.43672	2.1424
2	9.679	BB	0.1437	5465.87598	581.33179	97.8576
Totals :				5585.54300	596.76850	

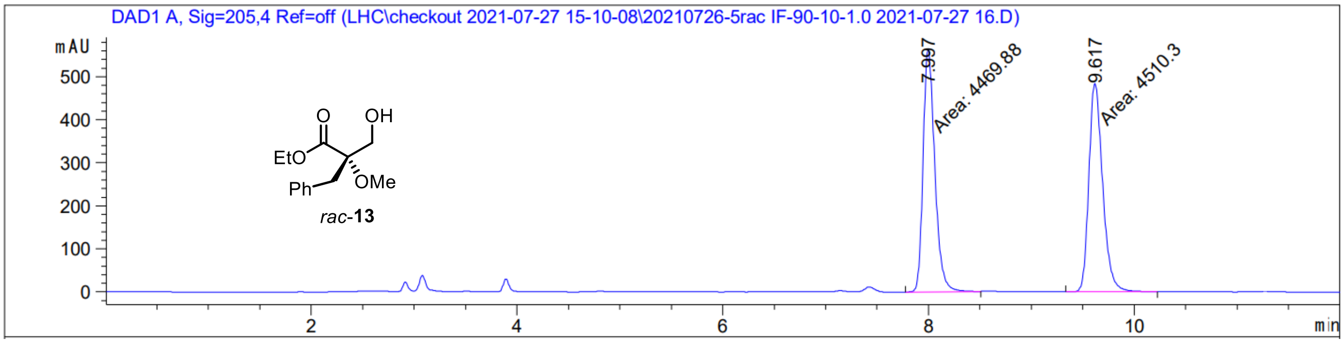

Signal 1: DAD1 A, Sig=205,4 Ref=off

Peak #	RetTime [min]	Type	Width [min]	Area [mAU*s]	Height [mAU]	Area %
1	7.997	MM	0.1318	4469.87939	565.38104	49.7749
2	9.617	MM	0.1551	4510.30273	484.69589	50.2251
Totals :				8980.18213	1050.07693	

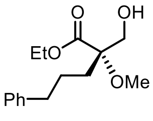

(+)-**57**

(+)-**57** was obtained as colorless oil (42.2 mg, 53% Yield) from the reductive desymmetrization of malonic ester **S57** (92.4 mg, 0.3 mmol) with **L9** in hexane at room temperature for 24 h using the general procedure D. R_f = 0.2 (hexane/EtOAc = 4:1). $[\alpha]_D^{25}$ = +3.3 (*c* = 1.0, $CHCl_3$).

^{1}H NMR (400 MHz, $CDCl_3$) δ 7.29-7.15 (m, 5H), 4.22 (q, *J* = 7.2 Hz, 2H), 3.78 (s, 2H), 3.31 (s, 3H), 2.62 (t, *J* = 7.2 Hz, 2H), 2.13 (br s, 1H), 1.80-1.61 (m, 4H), 1.28 (t, *J* = 7.2 Hz, 3H).

^{13}C NMR (100 MHz, $CDCl_3$) δ 172.45, 141.63, 128.32, 128.29, 125.85, 82.80, 63.91, 61.15, 51.80, 35.79, 31.47, 24.74, 14.19.

IR (neat, cm^{-1}) 3450, 2936, 1727, 1206, 1076, 699.

HRMS (ESI) calcd $C_{15}H_{22}NaO_4^+$ $[M+Na]^+$: 289.1410. Found: 289.1409.

HPLC analysis (Chiralpak ID-3, hexane/^{i}PrOH = 90/10, 1.0 mL/min, 205 nm; t_r (minor) = 11.06 min, t_r (major) = 14.71 min) gave the isomeric composition of the product: 91% *e.e.*.

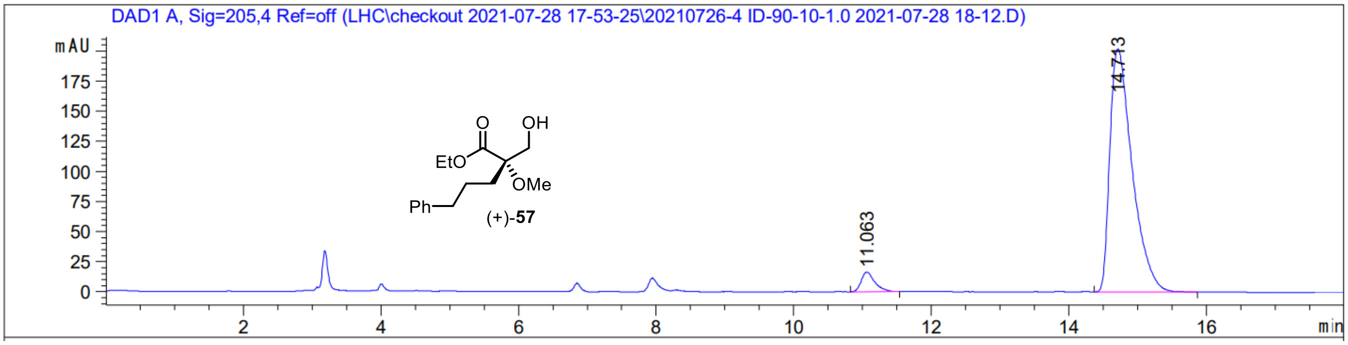

Signal 1: DAD1 A, Sig=205,4 Ref=off

Peak #	RetTime [min]	Type	Width [min]	Area [mAU*s]	Height [mAU]	Area %
1	11.063	BB	0.2052	223.73886	16.39262	4.6907
2	14.713	BB	0.3426	4546.13135	202.00134	95.3093
Totals :				4769.87021	218.39396	

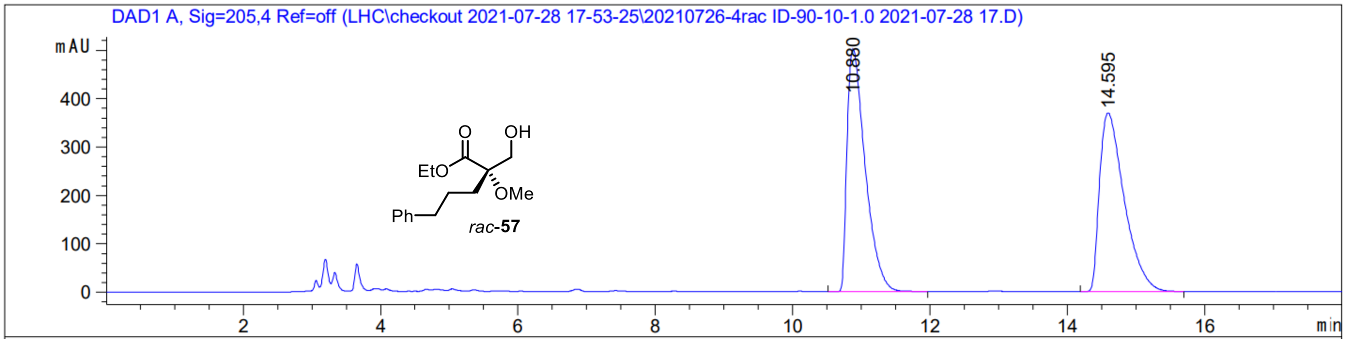

Signal 1: DAD1 A, Sig=205,4 Ref=off

Peak #	RetTime [min]	Type	Width [min]	Area [mAU*s]	Height [mAU]	Area %
1	10.880	BB	0.2819	9195.11035	501.50339	49.9433
2	14.595	BB	0.3833	9215.97656	369.10843	50.0567
Totals :				1.84111e4	870.61182	

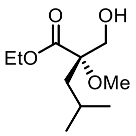

(–)-**58**

(–)-**58** was obtained as colorless oil (36.8 mg, 60% Yield) from the reductive desymmetrization of malonic ester **S58** (73.8 mg, 0.3 mmol) with **L9** in hexane at room temperature for 24 h using the general procedure D. R_f = 0.2 (hexane/EtOAc = 4:1). $[\alpha]_D^{25}$ = –6.0 (c = 1.0, $CHCl_3$).

^{1}H NMR (400 MHz, $CDCl_3$) δ 4.22 (m, 2H), 3.80 (s, 2H), 3.34 (s, 3H), 2.12 (br s, 1H), 1.77-1.65 (m, 3H), 1.29 (m, 3H), 0.92 (d, J = 6.4 Hz, 3H), 0.89 (d, J = 6.4 Hz, 3H).

^{13}C NMR (100 MHz, $CDCl_3$) δ 172.77, 82.97, 64.03, 61.15, 51.55, 40.40, 24.01, 23.56, 23.48, 14.15.

IR (neat, cm^{-1}) 3450, 2956, 1728, 1206, 1084.

HRMS (ESI) calcd $C_{10}H_{20}NaO_4^+$ $[M+Na]^+$: 227.1254. Found: 227.1255.

HPLC analysis (Chiralpak ID-3, hexane/^{i}PrOH = 97/3, 1.0 mL/min, 230 nm; t_r (minor) = 8.31 min, t_r (major) = 8.79 min) of benzoyl ester of (–)-**58** gave the isomeric composition of the product: 90% *e.e.*.

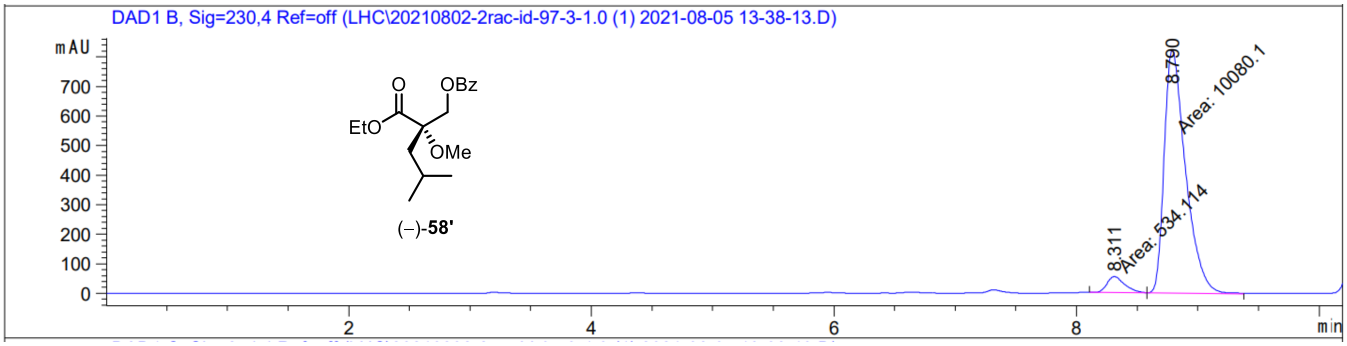

Signal 2: DAD1 B, Sig=230,4 Ref=off

Peak #	RetTime [min]	Type	Width [min]	Area [mAU*s]	Height [mAU]	Area %
1	8.311	MF	0.1658	534.11407	53.68959	5.0321
2	8.790	FM	0.2041	1.00801e4	823.14142	94.9679

Totals : 1.06142e4 876.83101

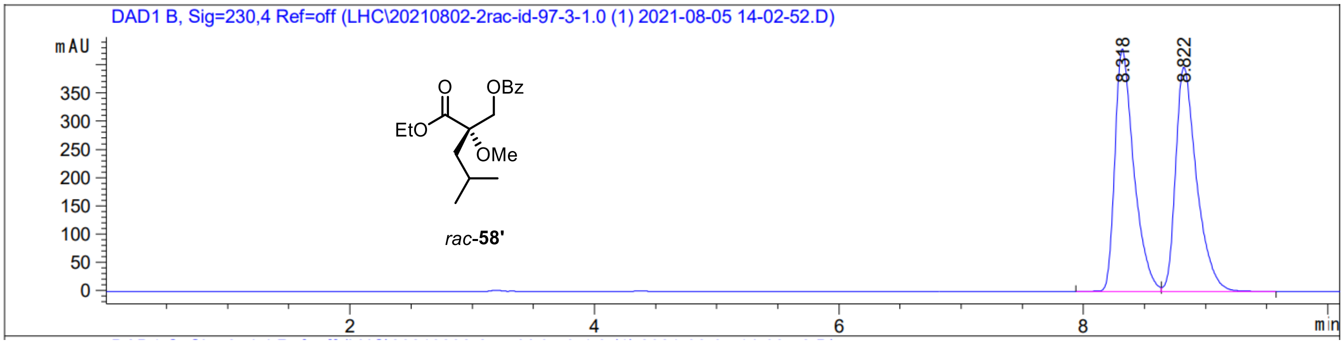

Signal 2: DAD1 B, Sig=230,4 Ref=off

Peak #	RetTime [min]	Type	Width [min]	Area [mAU*s]	Height [mAU]	Area %
1	8.318	BV	0.1619	4633.11475	429.36264	49.4443
2	8.822	VB	0.1793	4737.25781	396.88974	50.5557
Totals :				9370.37256	826.25238	

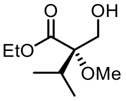

(+)-**59**

(+)-**59** was obtained as colorless oil (46.0 mg, 81% Yield) from the reductive desymmetrization of malonic ester **S59** (69.6 mg, 0.3 mmol) with **L9** in hexane at room temperature for 24 h using the general procedure D. R_f = 0.2 (hexane/EtOAc = 4:1). $[\alpha]_D^{25}$ = +22.5 (*c* = 1.0, $CHCl_3$).

^{1}H NMR (400 MHz, $CDCl_3$) δ 4.24 (m, 2H), 3.93 (d, *J* = 12.0 Hz, 1H), 3.86 (d, *J* = 12.0 Hz, 1H), 3.40 (s, 3H), 2.18-2.11 (m, 2H), 1.30 (t, *J* = 7.2 Hz, 3H), 0.94-0.92 (m, 6H).

^{13}C NMR (100 MHz, $CDCl_3$) δ 172.40, 85.18, 62.54, 60.89, 52.99, 32.54, 17.37, 16.72, 14.29.

IR (neat, cm^{-1}) 3450, 2966, 1723, 1208, 1092, 1018.

HRMS (ESI) calcd $C_9H_{18}NaO_4^+$ $[M+Na]^+$: 213.1097. Found: 213.1108.

HPLC analysis (Chiralpak ID-3, hexane/^{i}PrOH = 97/3, 1.0 mL/min, 230 nm; t_r (minor) = 6.80 min, t_r (major) = 7.25 min) of benzoyl ester of (+)-**59** gave the isomeric composition of the product: 99% *e.e.*.

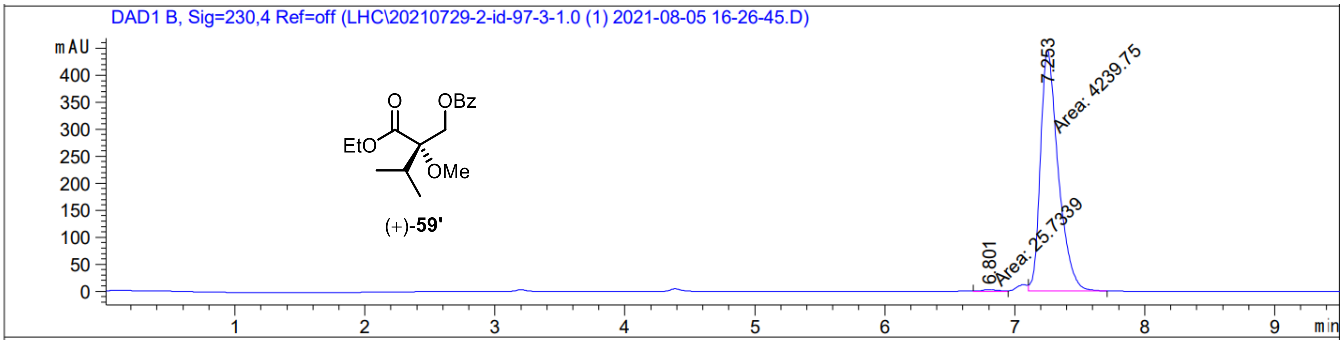

Signal 2: DAD1 B, Sig=230,4 Ref=off

Peak #	RetTime [min]	Type	Width [min]	Area [mAU*s]	Height [mAU]	Area %
1	6.801	MM	0.1401	25.73392	3.06171	0.6033
2	7.253	MM	0.1582	4239.74658	446.53534	99.3967

Totals : 4265.48050 449.59705

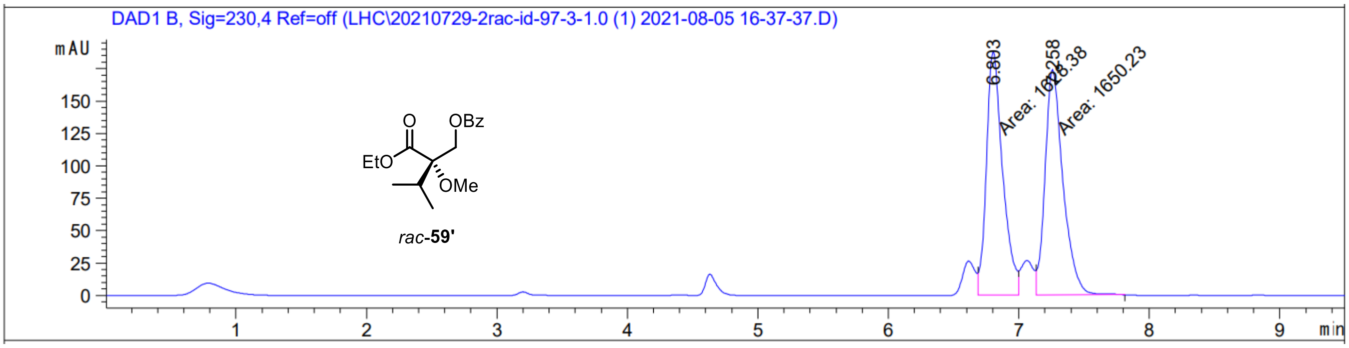

Signal 2: DAD1 B, Sig=230,4 Ref=off

Peak #	RetTime [min]	Type	Width [min]	Area [mAU*s]	Height [mAU]	Area %
1	6.803	MF	0.1442	1628.37500	188.16798	49.6666
2	7.258	FM	0.1581	1650.23376	173.93074	50.3334

Totals : 3278.60876 362.09872

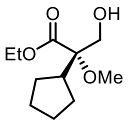

(+)-**60**

(+)-**60** was obtained as colorless oil (52.1 mg, 80% Yield) from the reductive desymmetrization of malonic

ester **S60** (77.4 mg, 0.3 mmol) with **L9** in hexane at room temperature for 24 h using the general procedure D. R_f = 0.5 (Hexane /EtOAc 3:1). $[\alpha]_D^{25}$ = +21.1 (*c* = 1.0, $CHCl_3$).

^{1}H NMR (400 MHz, $CDCl_3$) δ 4.24 (m, 2H), 3.91 (d, *J* = 12.0 Hz, 1H), 3.84 (d, *J* = 12.0 Hz, 1H), 3.41 (s, 3H), 2.28-2.26 (m, 2H), 1.57-1.49 (m, 8H), 1.30 (m, 3H).

^{13}C NMR (100 MHz, $CDCl_3$) δ 172.80, 84.24, 64.27, 60.93, 53.17, 44.18, 26.99, 26.41, 25.44, 25.26, 14.28.

IR (neat, cm^{-1}) 3450, 2950, 1723, 1208, 1022.

HRMS (ESI) calcd $C_{11}H_{20}NaO_4^+$ $[M+Na]^+$: 239.1254. Found: 239.1256.

HPLC analysis (Chiralpak IC-3, hexane/^{i}PrOH = 97/3, 1.0 mL/min, 230 nm; t_r (major) = 7.91 min, t_r (minor) = 8.46 min) of benzoyl ester of (+)-**60** gave the isomeric composition of the product: 96% *e.e.*.

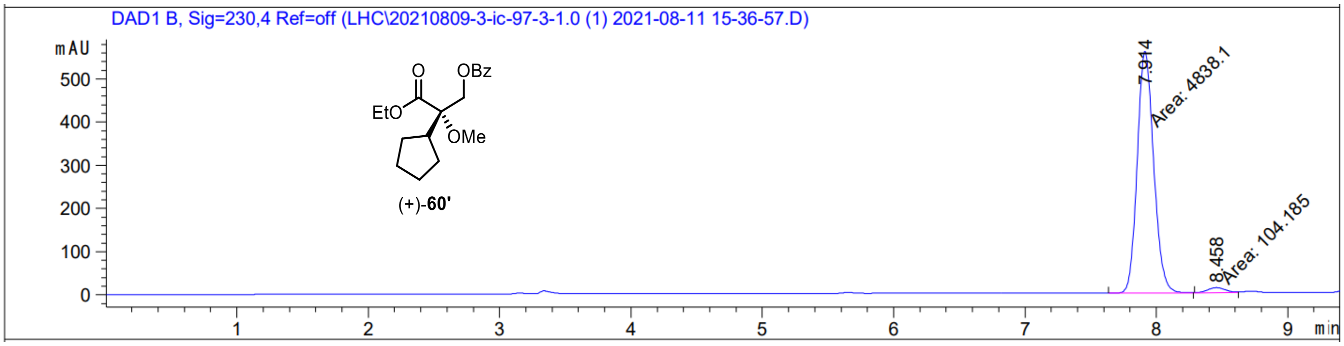

Signal 2: DAD1 B, Sig=230,4 Ref=off

Peak #	RetTime [min]	Type	Width [min]	Area [mAU*s]	Height [mAU]	Area %
1	7.914	MM	0.1440	4838.09814	559.87244	97.8920
2	8.458	MM	0.1512	104.18452	11.48683	2.1080
Totals :				4942.28267	571.35927	

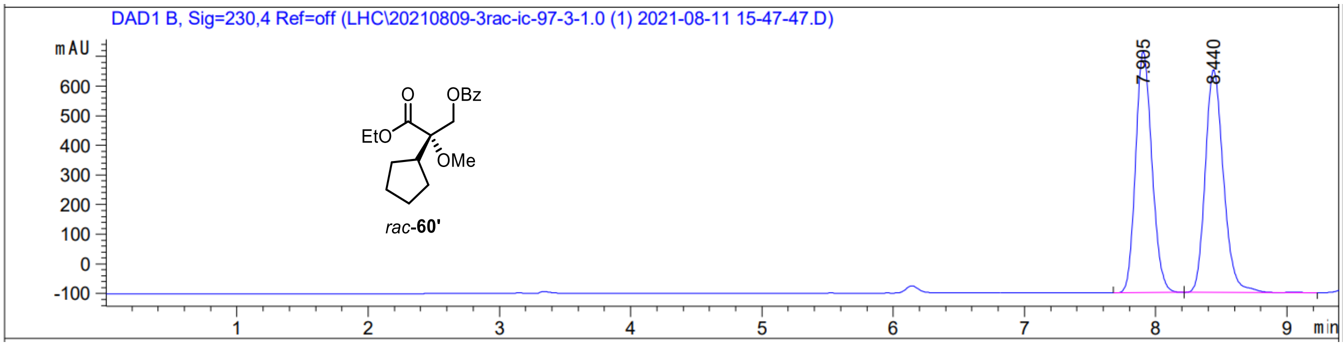

```
Signal 2: DAD1 B, Sig=230,4 Ref=off

Peak RetTime Type  Width     Area      Height     Area
  #   [min]        [min]   [mAU*s]     [mAU]        %
----|-------|----|-------|----------|----------|--------|
   1   7.905 BB    0.1327 7036.97021  815.08429  49.2774
   2   8.440 BV R  0.1484 7243.34326  750.98138  50.7226

Totals :                  1.42803e4  1566.06567
```

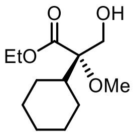

(+)-**61**

(+)-**61** was obtained as colorless oil (57.6 mg, 83% Yield) from the reductive desymmetrization of malonic ester **S61** (81.6 mg, 0.3 mmol) with **L9** in hexane at room temperature for 24 h using the general procedure D. R_f = 0.2 (hexane/EtOAc = 3:1). $[\alpha]_D^{25}$ = +18.5 (*c* = 1.0, $CHCl_3$).

^{1}H NMR (400 MHz, $CDCl_3$) δ 4.23 (m, 2H), 3.93 (d, *J* = 12.4 Hz, 1H), 3.86 (d, *J* = 12.4 Hz, 1H), 3.38 (s, 3H), 2.20 (s, 1H), 1.78-1.63 (m, 6H), 1.29 (t, *J* = 7.2 Hz, 3H), 1.17-1.09 (m, 5H).

^{13}C NMR (100 MHz, $CDCl_3$) δ 172.40, 85.12, 62.35, 60.87, 52.90, 42.72, 27.41, 26.88, 26.54, 26.44, 26.24, 14.28.

IR (neat, cm^{-1}) 3450, 2928, 1723, 1449, 1211, 1041.

HRMS (ESI) calcd $C_{12}H_{22}NaO_4^+$ $[M+Na]^+$: 253.1410. Found: 253.1408.

HPLC analysis (Chiralpak IG-3, hexane/^{i}PrOH = 97/3, 1.0 mL/min, 205 nm; t_r (minor) = 9.82 min, t_r (major) = 11.46 min) of benzoyl ester of (+)-**61** gave the isomeric composition of the product: 93% *e.e.*.

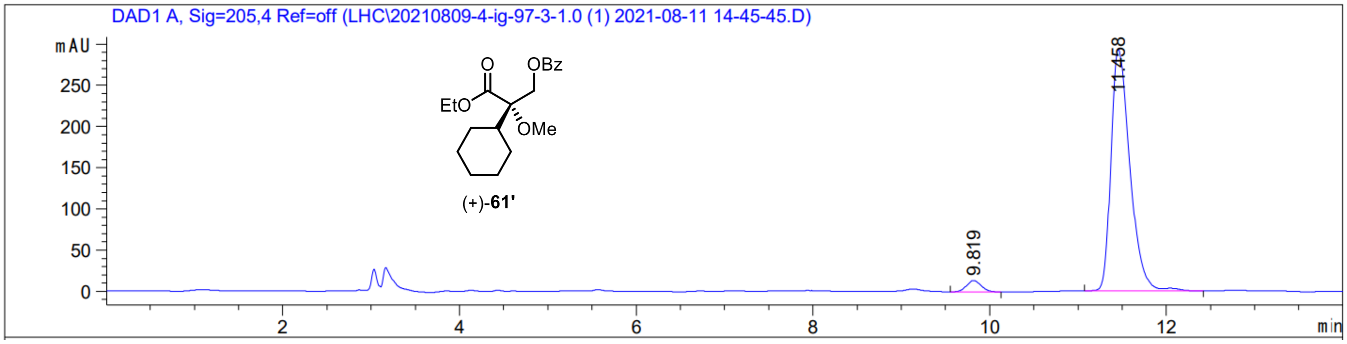

Signal 1: DAD1 A, Sig=205,4 Ref=off

Peak #	RetTime [min]	Type	Width [min]	Area [mAU*s]	Height [mAU]	Area %
1	9.819	BB	0.1788	160.13002	13.86373	3.5368
2	11.458	BV R	0.2259	4367.41406	293.68353	96.4632

Totals : 4527.54408 307.54726

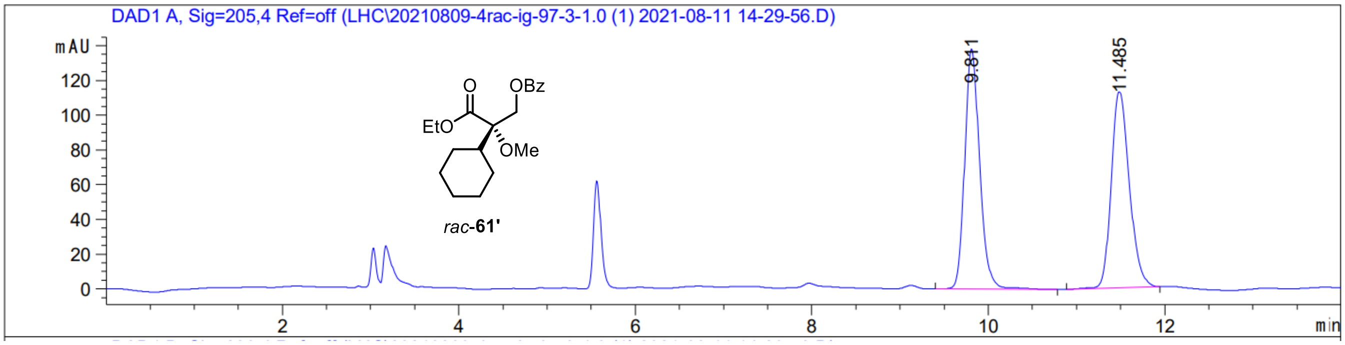

Signal 1: DAD1 A, Sig=205,4 Ref=off

Peak #	RetTime [min]	Type	Width [min]	Area [mAU*s]	Height [mAU]	Area %
1	9.811	BB	0.1821	1637.24780	138.28317	50.3521
2	11.485	BB	0.2207	1614.34692	113.00902	49.6479

Totals : 3251.59473 251.29219

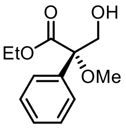

(+)-**62**

(+)-**62** was obtained as colorless oil (47.6 mg, 71% Yield) from the reductive desymmetrization of malonic ester **S62** (79.8 mg, 0.3 mmol) with **L9** in hexane at 0 °C for 24 h using the general procedure D. R_f = 0.2 (hexane/EtOAc = 3:1). $[\alpha]_D^{25}$ = +4.5 (*c* = 1.0, $CHCl_3$).

^{1}H NMR (400 MHz, $CDCl_3$) δ 7.43-7.33 (m, 5H), 4.29 (q, *J* = 7.2 Hz, 2H), 4.19 (d, *J* = 11.6 Hz, 1H), 4.07 (d, *J* = 11.6 Hz, 1H), 3.34 (s, 3H), 2.37 (br s, 1H), 1.28 (t, *J* = 7.2 Hz, 3H).

^{13}C NMR (100 MHz, $CDCl_3$) δ 171.38, 136.17, 128.51, 126.64, 85.08, 66.22, 61.55, 52.96, 14.08.

IR (neat, cm^{-1}) 3450, 2933, 1727, 1202, 1027, 699.

HRMS (ESI) calcd $C_{12}H_{16}NaO_4^+$ $[M+Na]^+$: 247.0941. Found: 247.0940.

HPLC analysis (Chiralpak ID-3, hexane/^{i}PrOH = 90/10, 1.0 mL/min, 205 nm; t_r (minor) = 11.32 min, t_r (major) = 13.92 min) gave the isomeric composition of the product: 86% *e.e.*.

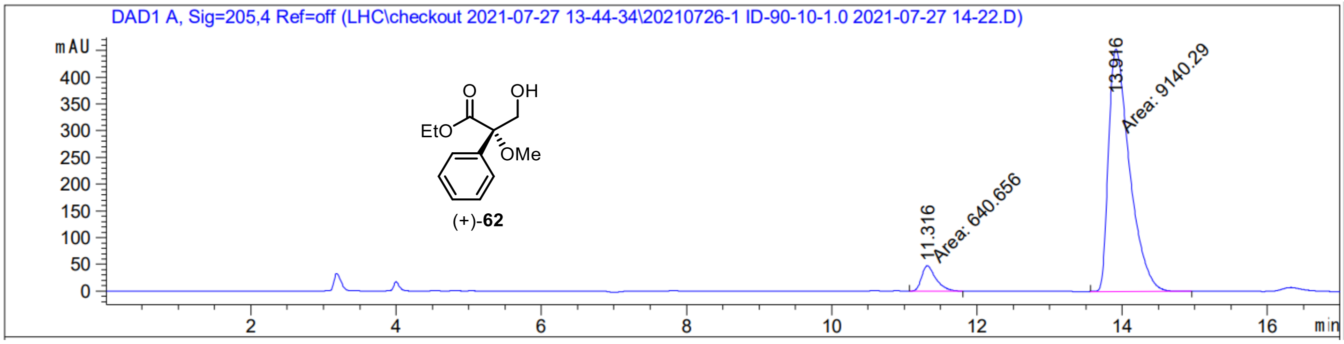

Signal 1: DAD1 A, Sig=205,4 Ref=off

Peak #	RetTime [min]	Type	Width [min]	Area [mAU*s]	Height [mAU]	Area %
1	11.316	MM	0.2255	640.65558	47.34708	6.5500
2	13.916	MM	0.3360	9140.28906	453.44269	93.4500

Totals : 9780.94464 500.78977

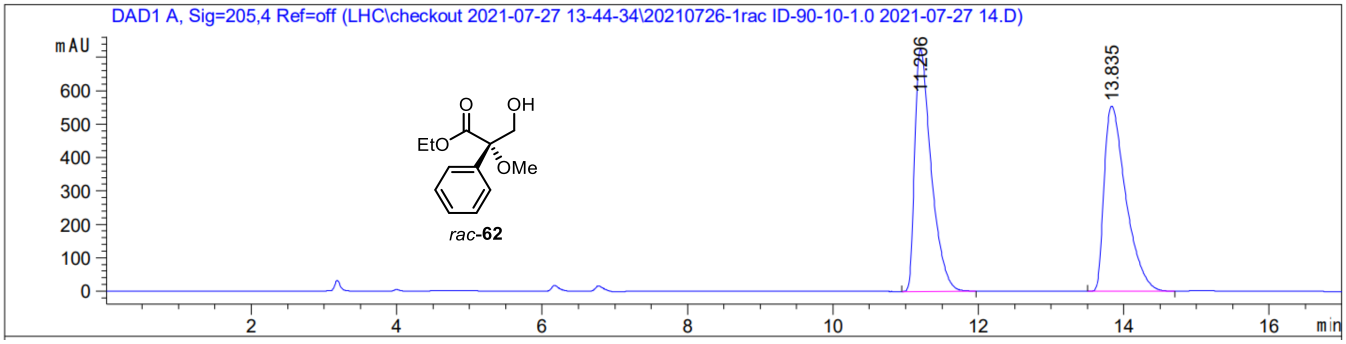

Signal 1: DAD1 A, Sig=205,4 Ref=off

Peak #	RetTime [min]	Type	Width [min]	Area [mAU*s]	Height [mAU]	Area %
1	11.206	BB	0.2371	1.14041e4	726.61755	49.8284
2	13.835	BB	0.3154	1.14827e4	555.02161	50.1716

Totals : 2.28868e4 1281.63916

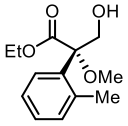

(–)-**63**

(–)-**63** was obtained as colorless oil (49.8 mg, 70% Yield) from the reductive desymmetrization of malonic ester **S63** (84.0 mg, 0.3 mmol) with **L9** in toluene at room temperature for 24 h using the general procedure D. R_f = 0.2 (hexane/EtOAc = 3:1). $[\alpha]_D^{25}$ = –53.7 (*c* = 1.0, $CHCl_3$).

^{1}H NMR (400 MHz, $CDCl_3$) δ 7.24-7.16 (m, 4H), 4.29-4.22 (m, 4H), 3.13 (s, 3H), 2.35 (br s, 1H), 2.35 (s, 3H), 1.22 (m, 3H).

^{13}C NMR (100 MHz, $CDCl_3$) δ 171.40, 137.53, 133.59, 132.11, 128.59, 127.13, 125.83, 84.13, 63.32, 61.62, 51.06, 19.80, 14.02.

IR (neat, cm^{-1}) 3439, 2931, 1732, 1200, 1114, 740.

HRMS (ESI) calcd $C_{13}H_{18}NaO_4^+$ $[M+Na]^+$: 261.1097. Found: 261.1097.

HPLC analysis (Chiralpak IG-3, hexane/^{i}PrOH = 80/20, 1.0 mL/min, 205 nm; t_r (minor) = 7.24 min, t_r (major) = 14.44 min) gave the isomeric composition of the product: 81% *e.e.*.

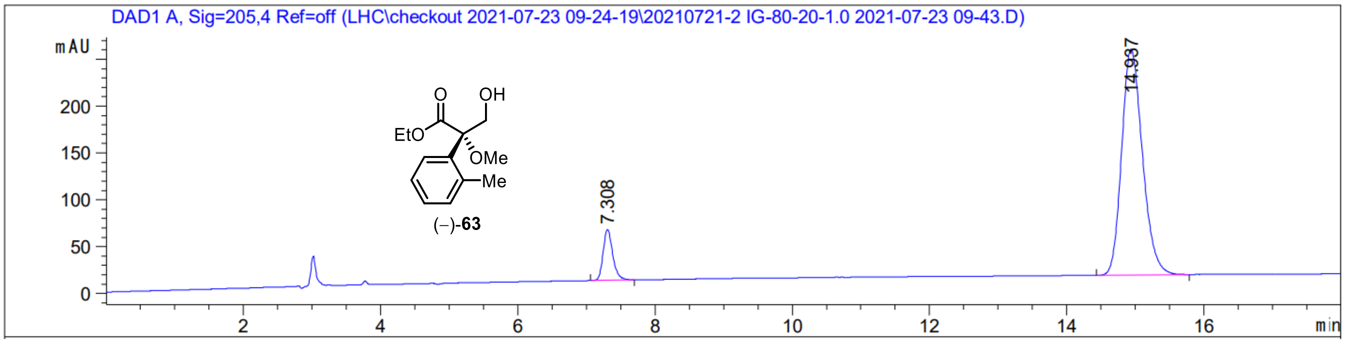

Signal 1: DAD1 A, Sig=205,4 Ref=off

Peak #	RetTime [min]	Type	Width [min]	Area [mAU*s]	Height [mAU]	Area %
1	7.308	BB	0.1491	527.13867	54.39416	9.4302
2	14.937	BB	0.3249	5062.77783	241.17352	90.5698

Totals : 5589.91650 295.56768

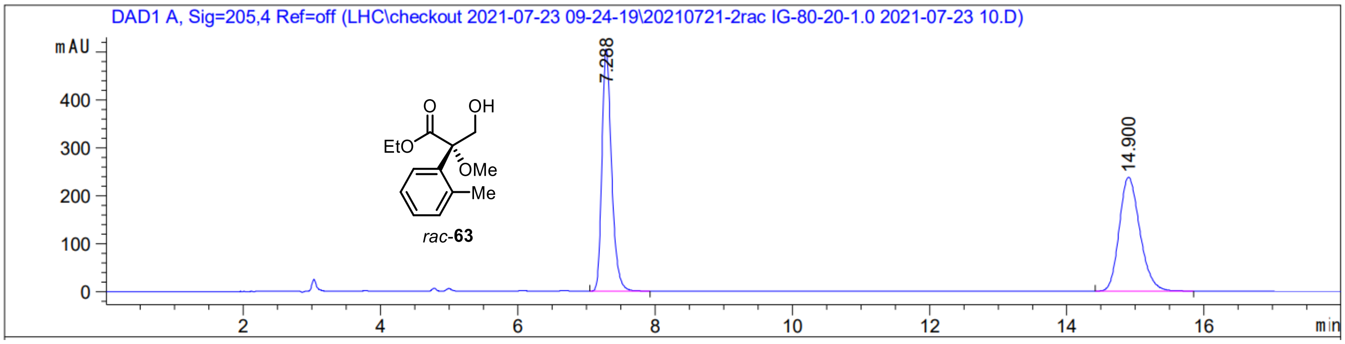

Signal 1: DAD1 A, Sig=205,4 Ref=off

Peak #	RetTime [min]	Type	Width [min]	Area [mAU*s]	Height [mAU]	Area %
1	7.288	BB	0.1477	4920.62305	504.92517	49.7757
2	14.900	BB	0.3219	4964.97559	237.48944	50.2243
Totals :				9885.59863	742.41461	

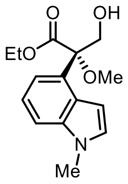

(+)-**64**

(+)-**64** was obtained as colorless oil (67.3 mg, 81% Yield) from the reductive desymmetrization of malonic ester **S64** (95.7 mg, 0.3 mmol) with **L9** in toluene at 0 °C for 24 h using the general procedure D. R_f = 0.2 (hexane/EtOAc = 3:1). $[\alpha]_D^{25}$ = +2.2 (c = 1.0, $CHCl_3$).

^{1}H NMR (400 MHz, $CDCl_3$) δ 7.33 (d, J = 8.4 Hz, 1H), 7.20 (dd, J = 8.4, 7.2 Hz, 1H), 7.07 (m, 2H), 6.72 (dd, J = 3.2, 0.8 Hz, 1H), 4.37-4.24 (m, 4H), 3.79 (s, 3H), 3.29 (s, 3H), 2.41 (br s, 1H), 1.24 (t, J = 7.2 Hz, 3H).

^{13}C NMR (100 MHz, $CDCl_3$) δ 171.70, 137.11, 129.12, 127.70, 126.16, 121.08, 118.00, 110.10, 101.04, 85.53, 64.69, 61.42, 52.35, 32.86, 14.09.

IR (neat, cm^{-1}) 3515, 2923, 1746, 1225, 1115, 740.

HRMS (ESI) calcd $C_{15}H_{19}NNaO_4^+$ $[M+Na]^+$: 300.1206. Found: 300.1209.

HPLC analysis (Chiralpak ID-3, hexane/^{i}PrOH = 70/30, 0.8 mL/min, 230 nm; t_r (minor) = 13.14 min, t_r (major) = 20.03 min) gave the isomeric composition of the product: 88% *e.e.*.

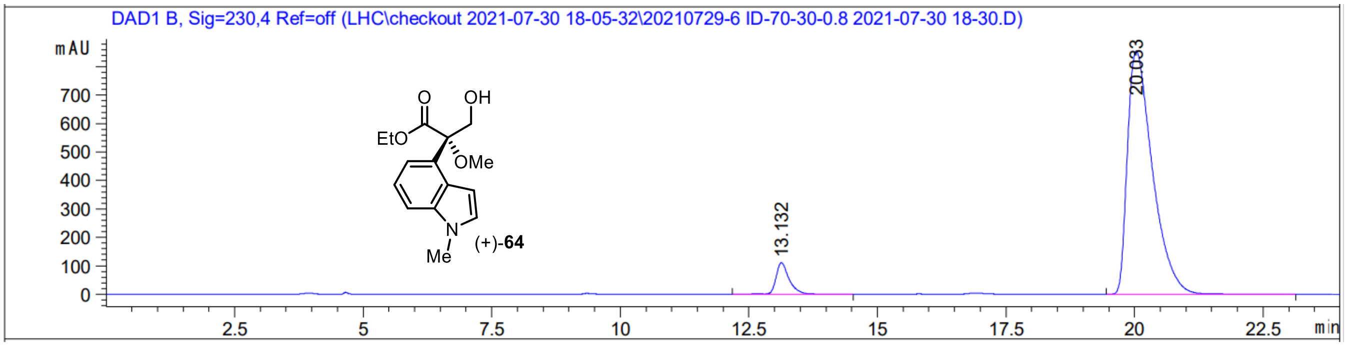

Signal 2: DAD1 B, Sig=230,4 Ref=off

Peak #	RetTime [min]	Type	Width [min]	Area [mAU*s]	Height [mAU]	Area %
1	13.132	VB R	0.2614	1954.80823	110.93648	6.3368
2	20.033	BB	0.5161	2.88937e4	855.36243	93.6632

Totals : 3.08485e4 966.29890

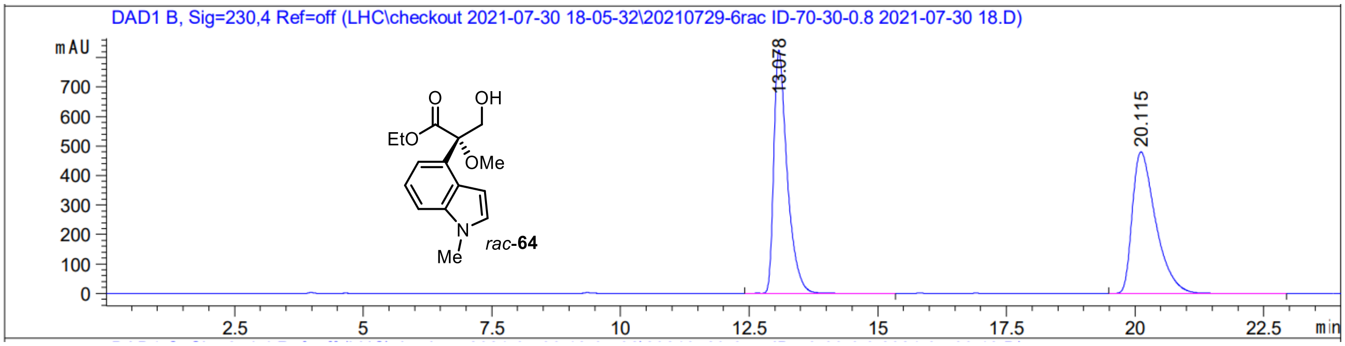

Signal 2: DAD1 B, Sig=230,4 Ref=off

Peak #	RetTime [min]	Type	Width [min]	Area [mAU*s]	Height [mAU]	Area %
1	13.078	BB	0.2717	1.49801e4	825.66809	49.5848
2	20.115	BB	0.4822	1.52309e4	479.98120	50.4152

Totals : 3.02110e4 1305.64929

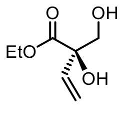

(+)-**66**

(+)-**66** was obtained as colorless oil (26.0 mg, 54% Yield) from the reductive desymmetrization of malonic ester **S66** (82.2 mg, 0.3 mmol) with **L9** in toluene at room temperature for 36 h using the general procedure

D. R_f = 0.2 (hexane/EtOAc = 2:1). $[\alpha]_D^{25}$ = +26.3 (c = 1.0, $CHCl_3$).

^{1}H NMR (400 MHz, $CDCl_3$) δ 5.90 (dd, J = 17.2, 10.8 Hz, 1H), 5.57 (dd, J = 17.2, 1.2 Hz, 1H), 5.27 (dd, J = 10.8, 1.2 Hz, 1H), 4.27 (q, J = 7.2 Hz, 2H), 3.88 (d, J = 11.2 Hz, 1H), 3.79 (br d, J = 10.8 Hz, 1H), 3.54 (d, J = 11.2 Hz, 1H), 2.65 (m, 1H), 1.31 (t, J = 7.2 Hz, 3H).

^{13}C NMR (100 MHz, $CDCl_3$) δ 173.46, 134.56, 117.20, 79.10, 67.60, 62.60, 14.04.

IR (neat, cm^{-1}) 3442, 2928, 1731, 1216, 1013, 932.

HRMS (ESI) calcd $C_7H_{12}NaO_4^+$ $[M+Na]^+$: 183.0628. Found: 183.0626.

HPLC analysis (Chiralpak ID-3, hexane/^{i}PrOH = 90/10, 1.0 mL/min, 230 nm; t_r (minor) = 10.15 min, t_r (major) = 12.52 min) of benzoyl ester of (+)-**66** gave the isomeric composition of the product: 86% *e.e.*.

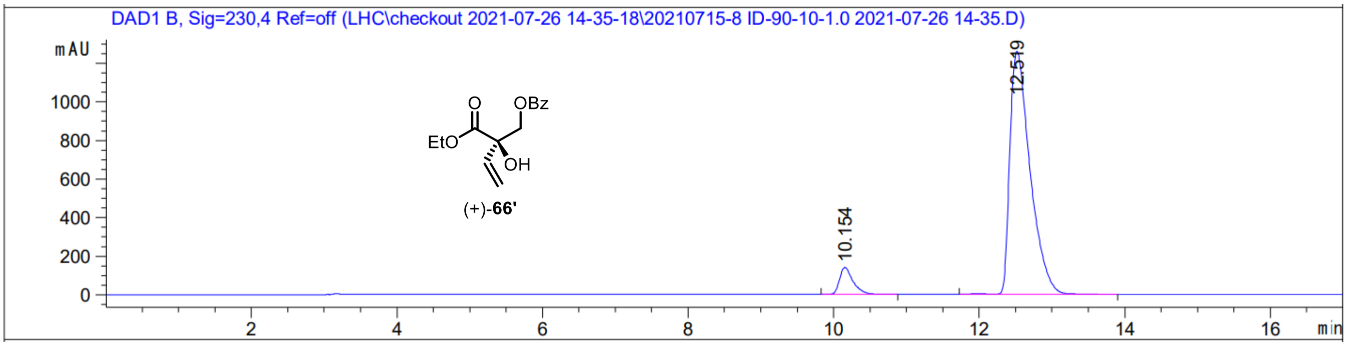

Signal 2: DAD1 B, Sig=230,4 Ref=off

Peak #	RetTime [min]	Type	Width [min]	Area [mAU*s]	Height [mAU]	Area %
1	10.154	BB	0.1902	1773.41846	139.65031	6.8199
2	12.519	VB R	0.2925	2.42302e4	1257.93152	93.1801
Totals :				2.60036e4	1397.58183	

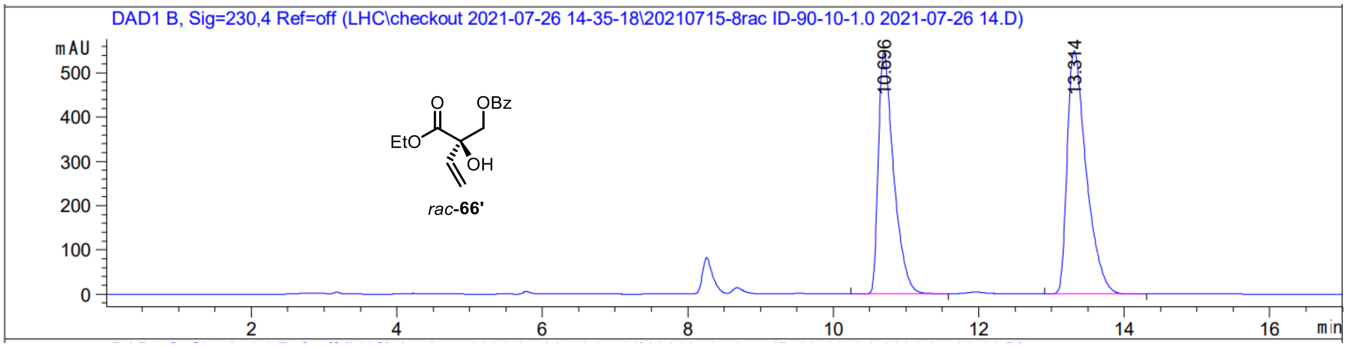

Signal 2: DAD1 B, Sig=230,4 Ref=off

Peak #	RetTime [min]	Type	Width [min]	Area [mAU*s]	Height [mAU]	Area %
1	10.696	BB	0.2217	8013.41846	544.63110	44.4725
2	13.314	BB	0.2745	1.00054e4	549.41180	55.5275
Totals :				1.80188e4	1094.04291	

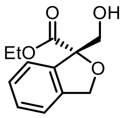

(+)-**14**

(+)-**14** was obtained as colorless oil (52.2 mg, 78% Yield) from the reductive desymmetrization of malonic ester **S14** (79.2 mg, 0.3 mmol) with **L9** in hexane at 0 ℃ for 18 h using the general procedure D. R_f = 0.2 (hexane/EtOAc = 3:1). $[\alpha]_D^{25}$ = +26.4 (*c* = 1.0, $CHCl_3$).

^{1}H NMR (400 MHz, $CDCl_3$) δ 7.42-7.23 (m, 4H), 5.34 (d, *J* = 12.4 Hz, 1H), 5.22 (d, *J* = 12.4 Hz, 1H), 4.23-4.14 (m, 3H), 3.92 (d, *J* = 12.0 Hz, 1H), 2.34 (br s, 1H), 1.26 (t, *J* = 7.2 Hz, 3H).

^{13}C NMR (100 MHz, $CDCl_3$) δ 171.28, 139.44, 136.68, 129.05, 127.78, 122.34, 121.12, 91.35, 73.63, 67.05, 61.67, 14.06.

IR (neat, cm^{-1}) 3465, 2928, 1732, 1215, 1014, 775, 588.

HRMS (ESI) calcd $C_{12}H_{14}NaO_4^+$ $[M+Na]^+$: 245.0784. Found: 245.0786.

HPLC analysis (Chiralpak IF-3, hexane/^{i}PrOH = 90/10, 1.0 mL/min, 205 nm; t_r (minor) = 13.04 min, t_r (major) = 15.33 min) gave the isomeric composition of the product: 91% *e.e.*.

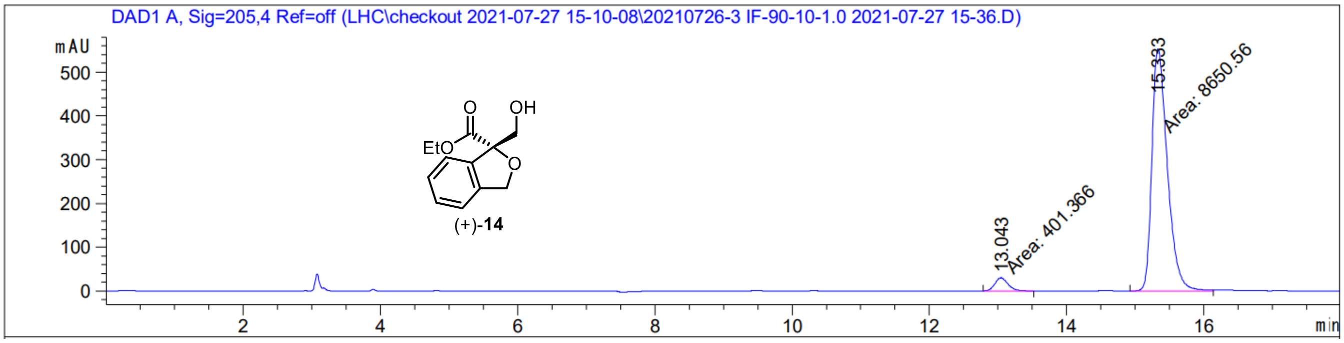

Signal 1: DAD1 A, Sig=205,4 Ref=off

Peak #	RetTime [min]	Type	Width [min]	Area [mAU*s]	Height [mAU]	Area %
1	13.043	BB	0.2082	410.50873	30.26416	4.5305
2	15.333	MM	0.2609	8650.56152	552.57104	95.4695

Totals : 9061.07025 582.83521

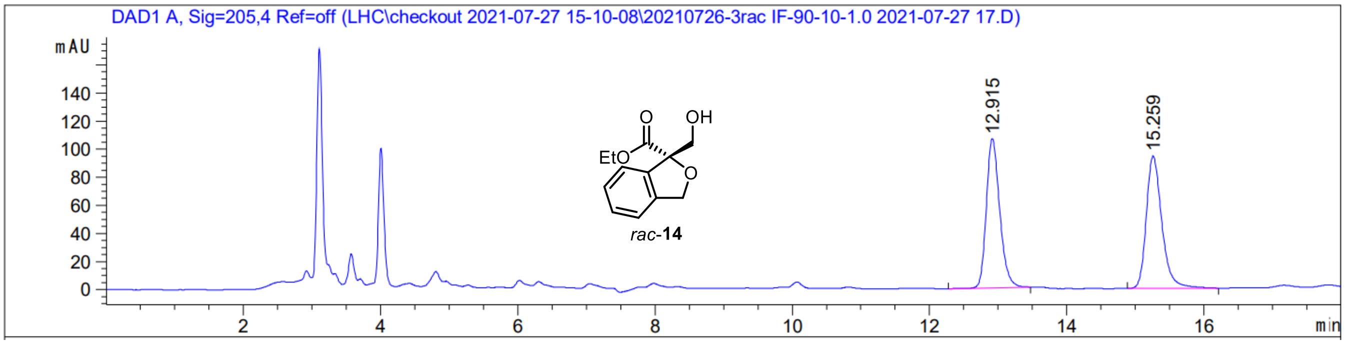

Signal 1: DAD1 A, Sig=205,4 Ref=off

Peak #	RetTime [min]	Type	Width [min]	Area [mAU*s]	Height [mAU]	Area %
1	12.915	BB	0.2077	1436.09009	106.23291	49.1658
2	15.259	BB	0.2397	1484.81982	94.27620	50.8342

Totals : 2920.90991 200.50911

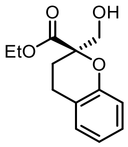

(+)-**67**

(+)-**67** was obtained as colorless oil (48.2 mg, 68% Yield) from the reductive desymmetrization of malonic ester **S67** (83.4 mg, 0.3 mmol) with **L9** in toluene at 0 ℃ for 4 h using the general procedure D. R_f = 0.2 (hexane/EtOAc = 3:1). $[\alpha]_D^{25}$ = +39.8 (*c* = 1.0, $CHCl_3$).

^{1}H NMR (400 MHz, $CDCl_3$) δ 7.13 (m, 1H), 7.02 (d, *J* = 7.6 Hz, 1H), 6.94 (m, 1H), 6.87 (m, 1H), 4.20 (m, 2H), 3.98 (d, *J* = 11.6 Hz, 1H), 3.87 (d, *J* = 11.6 Hz, 1H), 2.74-2.69 (m, 2H), 2.53 (br s, 1H), 2.30 (m, 1H), 2.00 (m, 1H), 1.21 (d, *J* = 7.2 Hz, 3H).

^{13}C NMR (100 MHz, $CDCl_3$) δ 171.55, 153.19, 129.20, 127.58, 120.79, 120.74, 116.61, 81.53, 67.47, 61.63, 25.35, 21.88, 14.06.

IR (neat, cm^{-1}) 3450, 2933, 1727, 1180, 1049, 752.

HRMS (ESI) calcd $C_{13}H_{16}NaO_4^+$ $[M+Na]^+$: 259.0941. Found: 259.0940.

HPLC analysis (Chiralpak IF-3, hexane/^{i}PrOH = 90/10, 1.0 mL/min, 205 nm; t_r (minor) = 10.10 min, t_r (major) = 13.33 min) gave the isomeric composition of the product: 88% *e.e.*.

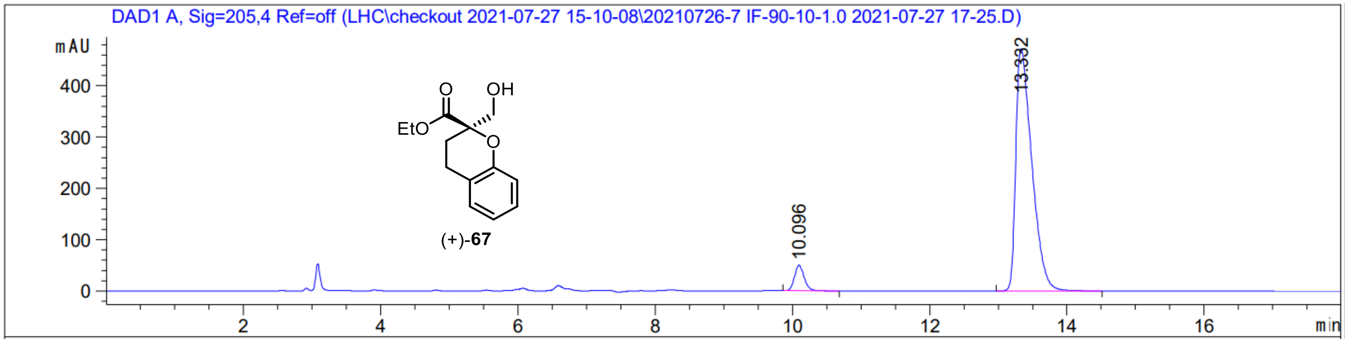

Signal 1: DAD1 A, Sig=205,4 Ref=off

Peak #	RetTime [min]	Type	Width [min]	Area [mAU*s]	Height [mAU]	Area %
1	10.096	BB	0.1513	491.45432	49.72747	6.0236
2	13.332	BB	0.2475	7667.42627	472.06955	93.9764

Totals : 8158.88058 521.79702

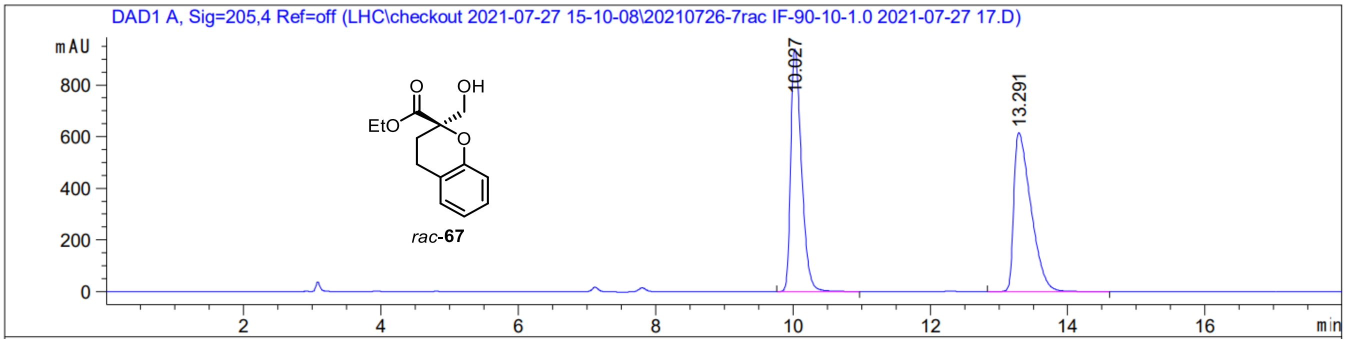

Signal 1: DAD1 A, Sig=205,4 Ref=off

Peak #	RetTime [min]	Type	Width [min]	Area [mAU*s]	Height [mAU]	Area %
1	10.027	BB	0.1689	1.02125e4	938.66241	48.8817
2	13.291	BB	0.2621	1.06798e4	616.38867	51.1183

Totals : 2.08923e4 1555.05109

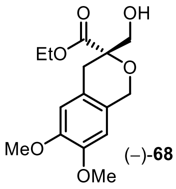

(–)-68

(–)-**68** was obtained as colorless oil (53.3 mg, 60% Yield) from the reductive desymmetrization of malonic ester **S68** (101.4 mg, 0.3 mmol) with **L9** in toluene at 0 ℃ for 24 h using the general procedure D. R_f = 0.2 (hexane/EtOAc = 1:1). $[\alpha]_D^{25}$ = –55.3 (*c* = 0.5, $CHCl_3$).

^{1}H NMR (400 MHz, $CDCl_3$) δ 6.59 (s, 1H), 6.48 (s, 1H), 5.09 (d, *J* = 14.8 Hz, 1H), 4.79 (d, *J* = 14.8 Hz, 1H), 4.14 (m, 2H), 3.83-3.81 (m, 7H), 3.75 (d, *J* = 9.6 Hz, 1H), 3.04 (d, *J* = 16.0 Hz, 1H), 2.89 (d, *J* = 16.0 Hz, 1H), 2.44 (br s, 1H), 1.18 (t, *J* = 7.2 Hz, 3H).

^{13}C NMR (100 MHz, $CDCl_3$) δ 171.99, 147.90, 147.84, 125.01, 122.29, 111.24, 106.87, 79.01, 67.79, 64.73, 61.32, 55.85, 55.83, 31.10, 14.09.

IR (neat, cm^{-1}) 3480, 2938, 1728, 1516, 1226, 1080.

HRMS (ESI) calcd $C_{15}H_{20}NaO_6^+$ $[M+Na]^+$: 319.1152. Found: 319.1151.

HPLC analysis (Chiralpak IB-3, hexane/^{i}PrOH = 80/20, 1.0 mL/min, 205 nm; t_r (major) = 10.90 min, t_r (minor) = 12.33 min) gave the isomeric composition of the product: 82% *e.e.*.

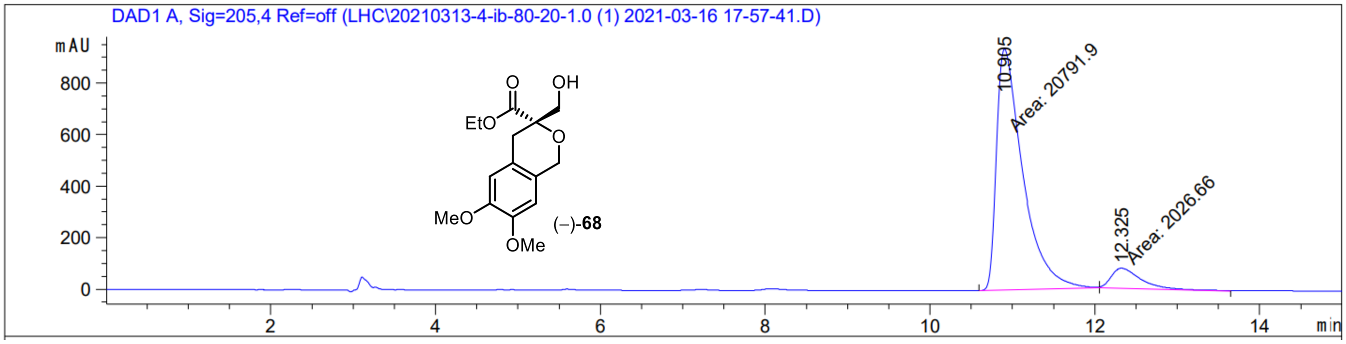

Signal 1: DAD1 A, Sig=205,4 Ref=off

Peak #	RetTime [min]	Type	Width [min]	Area [mAU*s]	Height [mAU]	Area %
1	10.905	MM	0.3697	2.07919e4	937.29993	91.1183
2	12.325	MM	0.4317	2026.66382	78.24919	8.8817

Totals : 2.28185e4 1015.54912

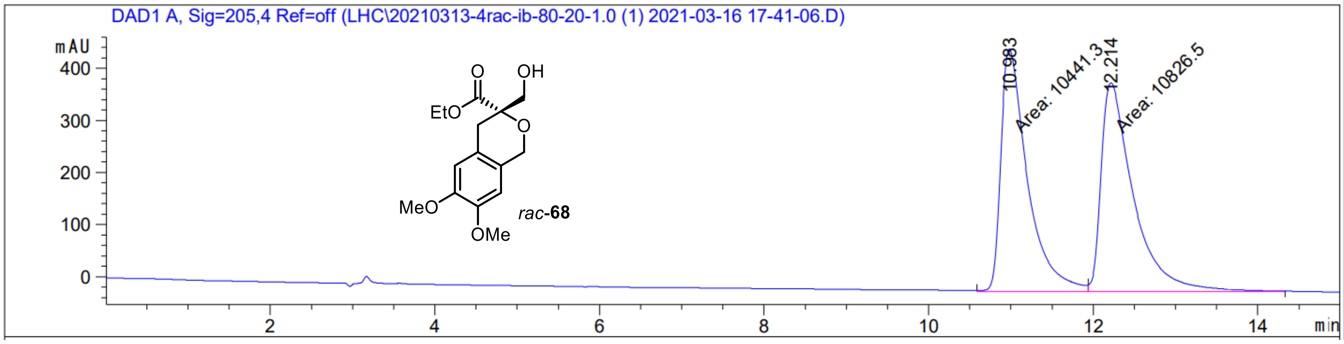


```
Signal 1: DAD1 A, Sig=205,4 Ref=off

Peak RetTime Type  Width      Area       Height      Area
  #    [min]        [min]   [mAU*s]     [mAU]         %
----|-------|----|-------|----------|----------|--------|
   1  10.983 MM    0.3742 1.04413e4  465.00397  49.0944
   2  12.214 MM    0.4510 1.08265e4  400.08005  50.9056

Totals :                  2.12678e4  865.08401
```

<u>Kinetic resolution of oxymalonic esters of (±)-**69**</u>

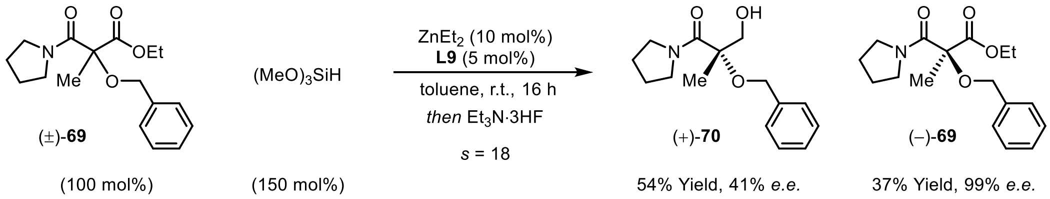

Reductive desymmetrization of malonic ester (±)-**69** (30.5 mg, 0.1 mmol) with **L9** in toluene at room temperature for 16 h using the general procedure D gave (+)-**70** in 54% yield and recovered (–)-**69** in 37% yield.

For (–)-**69**: R_f = 0.6 (hexane/EtOAc = 3:1). $[\alpha]_D^{25}$ = –11.4 (*c* = 0.5, $CHCl_3$).

^{1}H NMR (400 MHz, $CDCl_3$) δ 7.49 – 7.12 (m, 5H), 4.63 (d, *J* = 11.2 Hz, 1H), 4.40 (d, *J* = 11.2 Hz, 1H), 4.26 (q, *J* = 7.1 Hz, 2H), 3.65 (dt, *J* = 11.7, 6.3 Hz, 1H), 3.53 (dt, *J* = 13.0, 6.5 Hz, 1H), 3.43 (dt, *J* = 12.6, 6.6 Hz, 1H), 3.26 (m, 1H), 1.76 (s, 3H), 1.79 – 1.68 (m, 4H),1.28 (t, *J* = 7.1 Hz, 3H).

^{13}C NMR (100 MHz, $CDCl_3$) δ 170.79, 166.44, 137.53, 128.34, 127.74, 127.69, 82.79, 66.74, 61.63, 47.10, 46.48, 26.46, 23.28, 19.90, 14.14.

IR (neat, cm^{-1}) 2978, 2878, 1733, 1643, 1131, 753.

HRMS (ESI) calcd $C_{17}H_{24}NO_4^+$ $[M+H]^+$: 306.1700. Found: 306.1701.

HPLC analysis (Chiralpak ID-3, hexane/^{i}PrOH = 90/10, 1.0 mL/min, 205 nm; t_r (major) = 25.77 min, t_r (minor) = 24.80 min) gave the isomeric composition of the product: 99% *e.e.*.

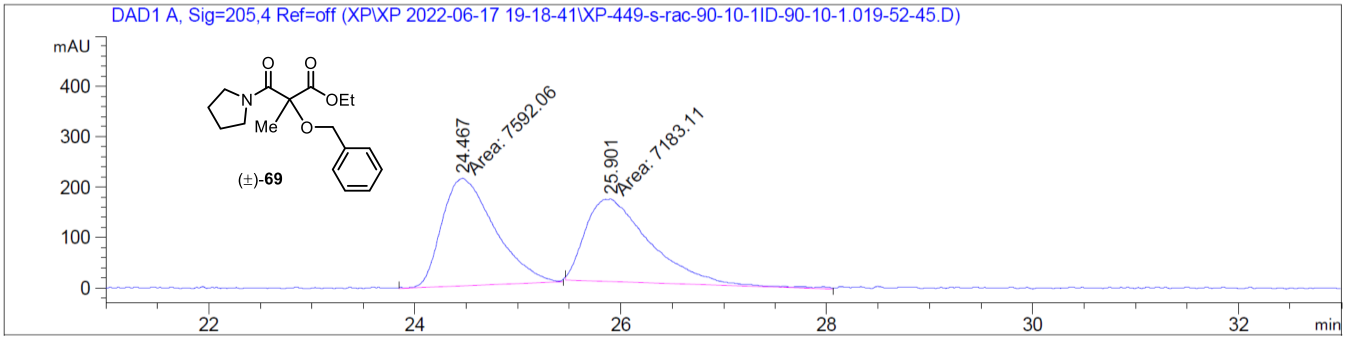

Signal 1: DAD1 A, Sig=205,4 Ref=off

Peak #	RetTime [min]	Type	Width [min]	Area [mAU*s]	Height [mAU]	Area %
1	24.467	MM	0.5930	7592.06152	213.36739	51.3839
2	25.901	MM	0.7311	7183.10547	163.76167	48.6161
Totals :				1.47752e4	377.12906	

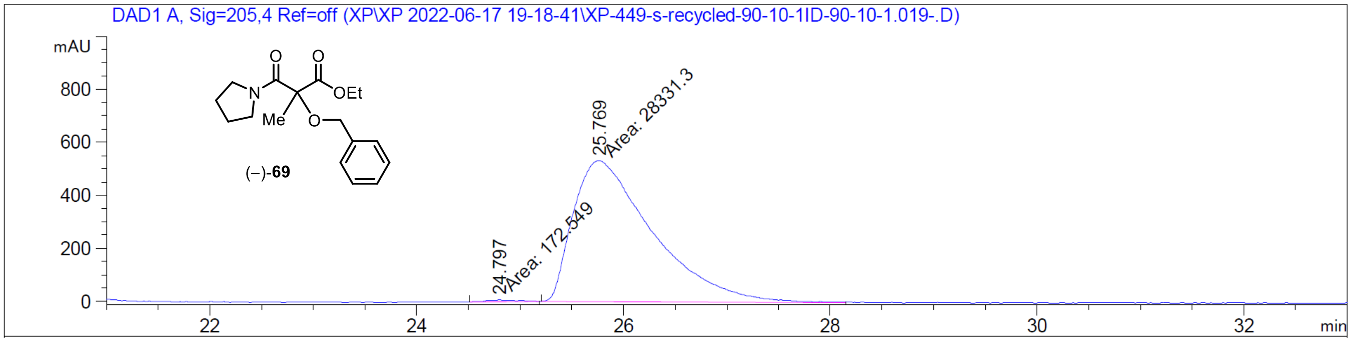

Signal 1: DAD1 A, Sig=205,4 Ref=off

Peak #	RetTime [min]	Type	Width [min]	Area [mAU*s]	Height [mAU]	Area %
1	24.797	MM	0.3860	172.54924	7.45056	0.6054
2	25.769	MM	0.8887	2.83313e4	531.31885	99.3946
Totals :				2.85039e4	538.76941	

For (+)-**70**: R_f = 0.2 (hexane/EtOAc = 3:1). $[\alpha]_D^{25}$ = +7.8 (*c* = 0.5, $CHCl_3$).

^{1}H NMR (400 MHz, $CDCl_3$) δ 7.40 – 7.23 (m, 5H), 4.72 (d, *J* = 11.3 Hz, 1H), 4.37 (d, *J* = 11.3 Hz, 1H), 4.01 (dd, *J* = 11.6, 6.2 Hz, 1H), 3.79 (dd, *J* = 11.5, 6.0 Hz, 2H), 3.61 – 3.48 (m, 3H), 3.39 (t, *J* = 6.9 Hz, 1H), 1.81 (ddt, *J* = 9.0, 6.2, 2.9 Hz, 4H), 1.50 (s, 3H).

^{13}C NMR (100 MHz, $CDCl_3$) δ 172.17, 138.01, 128.44, 127.63, 127.29, 80.86, 67.24, 66.74, 47.21, 47.03,

26.82, 22.98, 20.04.

IR (neat, cm^{-1}) 3412, 2929, 2877, 1605, 1059, 736, 697.

HRMS (ESI) calcd $C_{15}H_{22}NO_3^+$ [M+H]$^+$: 264.1594. Found: 264.1596.

HPLC analysis (Chiralpak IC-3, hexane/^{i}PrOH = 70/30, 0.8 mL/min, 210 nm; t_r (major) = 29.08 min, t_r (minor) = 34.21 min) gave the isomeric composition of the product: 41% *e.e.*.

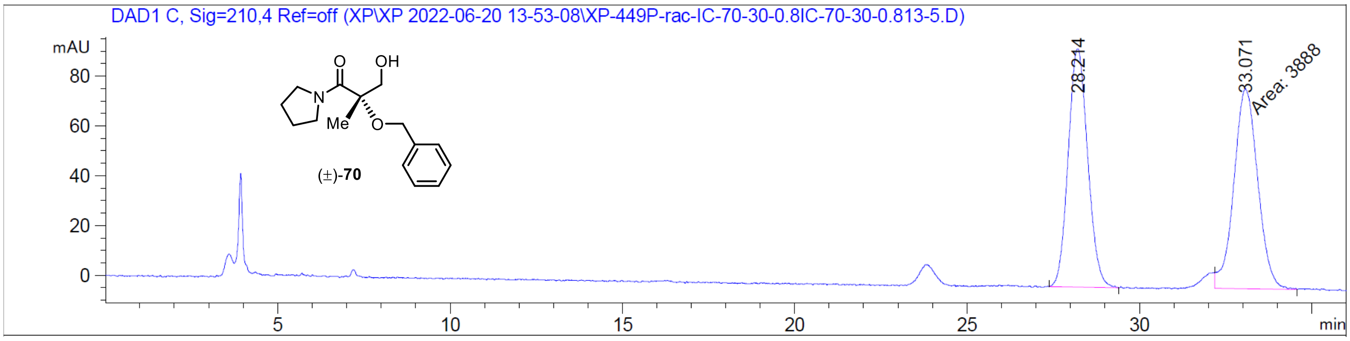

Signal 3: DAD1 C, Sig=210,4 Ref=off

Peak #	RetTime [min]	Type	Width [min]	Area [mAU*s]	Height [mAU]	Area %
1	28.214	BV R	0.5313	3768.60840	95.72402	49.2203
2	33.071	FM	0.8054	3888.00073	80.45589	50.7797

Totals : 7656.60913 176.17991

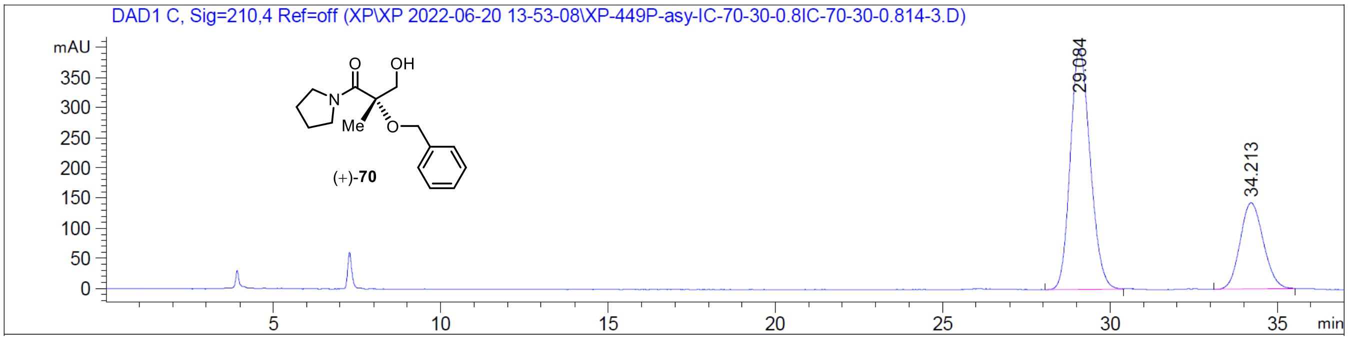

Signal 3: DAD1 C, Sig=210,4 Ref=off

Peak #	RetTime [min]	Type	Width [min]	Area [mAU*s]	Height [mAU]	Area %
1	29.084	BV R	0.5915	1.64786e4	399.25961	70.4821
2	34.213	BV R	0.6551	6901.21240	143.50174	29.5179

Totals : 2.33798e4 542.76135

1.5 Procedures for the Application of the Desymmetrization Products

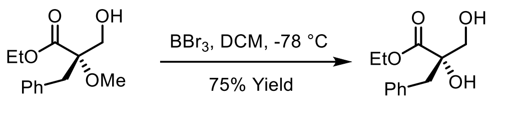

(+)-**13** (96% *e.e.*) (+)-**72** (95% *e.e.*)

A solution of alcohol (+)-**13** (21.0 mg, 0.089 mmol) in DCM (2 mL) was cooled to -78 °C and BBr_3 (0.18 mL, 1.0 M in DCM, 0.18 mmol) was added dropwise. The resulting mixture was stirred at -78 °C for 2 h. The reaction was quenched with saturated $NaHCO_3$ solution, extracted with EtOAc, washed with brine, dried over Na_2SO_4, and filtered. The filtrate was concentrated under vacuum and submitted to flash column chromatography (hexane/EtOAc = 3:1) to afford the desired diol (+)-**72** (15.0 mg, 75% yield) as colorless oil. $[\alpha]_D^{25}$ = +12.3 (*c* = 1.0, $CHCl_3$). $[\alpha]_D^{13}$ lit. = +10.43 (*c* = 0.86, $CHCl_3$).[6]

^{1}H NMR (400 MHz, $CDCl_3$) δ 7.29-7.17 (m, 3H), 4.20 (m, 2H), 3.91 (d, *J* = 11.2 Hz, 1H), 3.69 (d, *J* = 11.2 Hz, 1H), 3.52-3.48 (m, 1H), 2.99 (d, *J* = 13.6 Hz, 1H), 2.89 (d, *J* = 13.6 Hz, 1H), 1.28 (t, *J* = 7.2 Hz, 3H).

^{13}C NMR (100 MHz, $CDCl_3$) δ 174.09, 134.92, 129.99, 128.22, 127.02, 78.99, 67.66, 62.27, 41.21, 14.12.

HPLC analysis (Chiralpak IF-3, hexane/^{i}PrOH = 85/15, 1.0 mL/min, 205 nm; t_r (major) = 9.81 min, t_r (minor) = 11.88 min) gave the isomeric composition of the product: 95% *e.e.*.

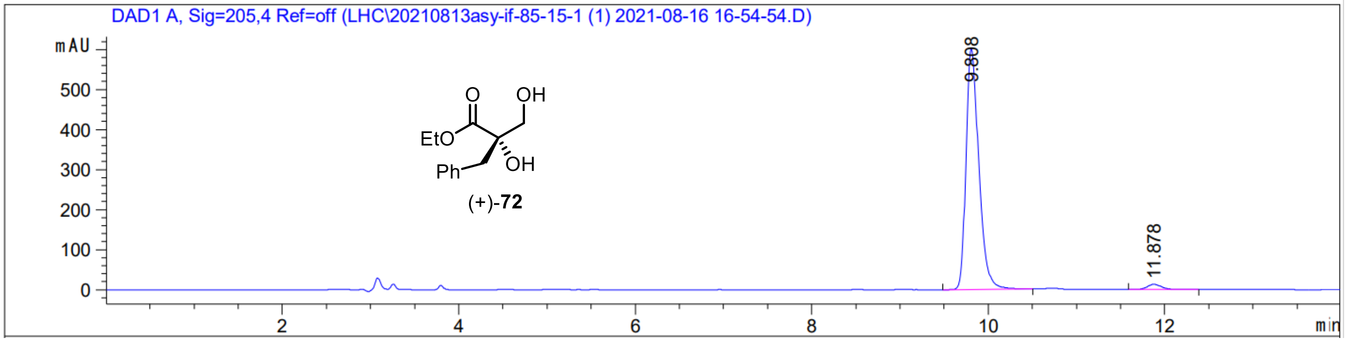

Signal 1: DAD1 A, Sig=205,4 Ref=off

Peak #	RetTime [min]	Type	Width [min]	Area [mAU*s]	Height [mAU]	Area %
1	9.808	BB	0.1558	6079.09668	602.04932	97.5712
2	11.878	BB	0.1775	151.32246	13.03250	2.4288

Totals : 6230.41914 615.08181

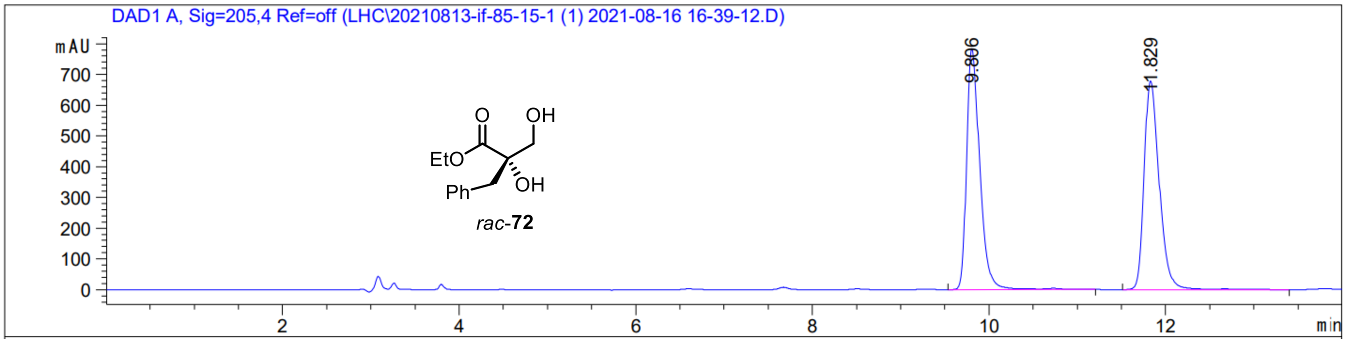

Signal 1: DAD1 A, Sig=205,4 Ref=off

Peak #	RetTime [min]	Type	Width [min]	Area [mAU*s]	Height [mAU]	Area %
1	9.806	BV R	0.1590	8144.58887	783.28009	50.1952
2	11.829	BV R	0.1823	8081.25000	678.89478	49.8048
Totals :				1.62258e4	1462.17487	

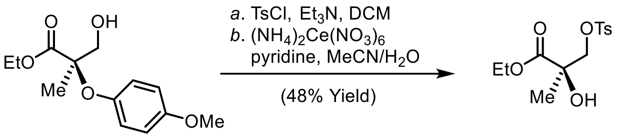

(–)-**12** (90% *e.e.*) (+)-**73** (91% *e.e.*)

To a stirred solution of alcohol (–)-**12** (1.02 g, 4.0 mmol) in DCM (12 mL) was added tosyl chloride (2.30 g, 12 mmol) and Et_3N (1.7 mL, 12 mmol), which was then stirred at room temperature overnight. The reaction mixture was diluted with water, extracted with EtOAc, washed with brine, dried over Na_2SO_4, and filtered. The filtrate was evaporated and purified by flash column chromatography (hexane/EtOAc = 4:1) to yield the tosyl protected product as colorless oil.

To a stirred solution of the above product in MeCN/H_2O (12.0 mL, MeCN/H_2O = 3:1) were added $(NH_4)_2Ce(NO_3)_6$ (4.38 g, 8.0 mmol) and pyridine (0.72 mL, 8.0 mmol). The reaction mixture was stirred at 0 °C for 2 h. The mixture was then quenched with saturated NaCl solution, extracted with EtOAc, washed with brine, dried over Na_2SO_4, and filtered. The filtrate was concentrated under vacuum and submitted to flash column chromatography (hexane/EtOAc = 4:1) to yield (+)-**73** (584.0 mg, 48%) as yellow oil. $[\alpha]_D^{25}$ = +1.15 (c = 1.0, $CHCl_3$).

^{1}H NMR (400 MHz, $CDCl_3$) δ 7.76 (d, J = 8.0 Hz, 2H), 7.34 (d, J = 8.0 Hz, 2H), 4.23-4.18 (m, 3H), 3.98 (d, J = 9.6 Hz, 1H), 3.39 (br s, 1H), 2.44 (s, 3H), 1.35 (s, 3H), 1.25 (t, J = 7.2 Hz, 3H).

^{13}C NMR (100 MHz, $CDCl_3$) δ 173.36, 145.01, 132.59, 129.83, 127.92, 74.07, 73.32, 62.62, 21.78, 21.61,

13.98.

IR (neat, cm^{-1}) 3535, 2933, 1730, 1447, 1184, 1105, 757.

HRMS (ESI) calcd $C_{13}H_{19}O_6S^+$ $[M+H]^+$: 303.0897. Found: 303.0898.

HPLC analysis (Chiralpak IF-3, hexane/^{i}PrOH = 70/30, 0.7 mL/min, 230 nm; t_r (major) = 20.08 min, t_r (minor) = 23.81 min) gave the isomeric composition of the product: 91% *e.e.*

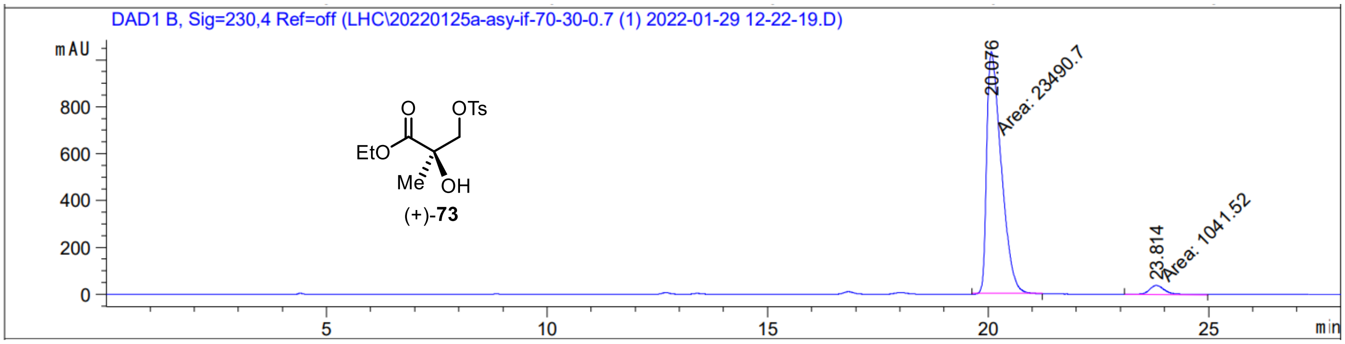

Signal 2: DAD1 B, Sig=230,4 Ref=off

Peak #	RetTime [min]	Type	Width [min]	Area [mAU*s]	Height [mAU]	Area %
1	20.076	MM	0.3795	2.34907e4	1031.69263	95.7545
2	23.814	MM	0.4469	1041.52393	38.84243	4.2455

Totals : 2.45322e4 1070.53505

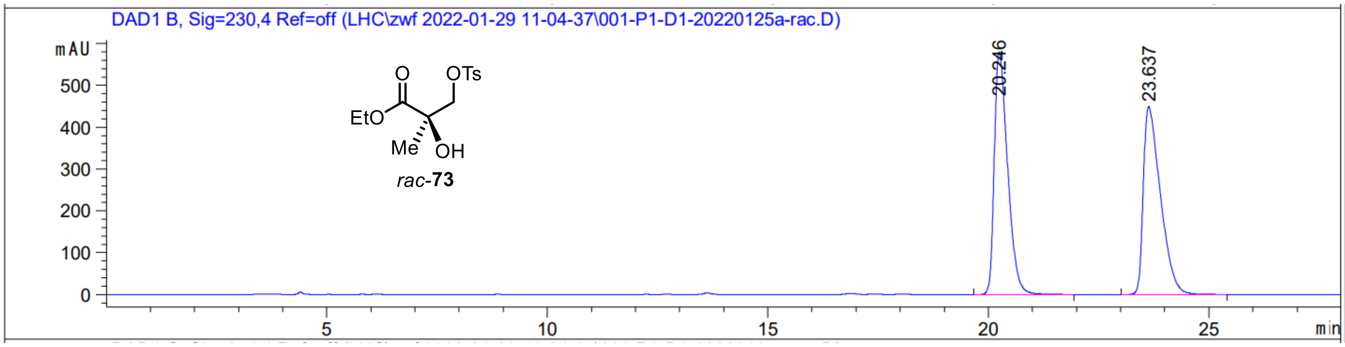

Signal 2: DAD1 B, Sig=230,4 Ref=off

Peak #	RetTime [min]	Type	Width [min]	Area [mAU*s]	Height [mAU]	Area %
1	20.246	BB	0.3206	1.21630e4	580.24200	50.0386
2	23.637	BB	0.4095	1.21443e4	449.04391	49.9614

Totals : 2.43073e4 1029.28592

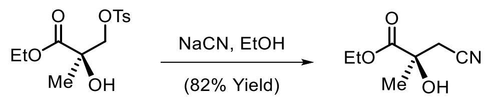

(+)-**73** (-)-**S74**

To a stirred solution of (+)-**73** (220 mg, 0.73 mmol) in EtOH (3 mL) was added NaCN (71 mg, 1.46 mmol), which was then stirred at room temperature for 2h. The reaction mixture was diluted with water, extracted with EtOAc, washed with brine, dried over Na_2SO_4, and filtered. The filtrate was evaporated and purified by flash column chromatography (hexane/EtOAc = 3:1) to yield (–)-**S74** (94.0 mg, 82%) as colorless oil. $[\alpha]_D^{25}$ = –13.9 (c = 1.0, $CHCl_3$).

^{1}H NMR (400 MHz, $CDCl_3$) δ 4.32 (m, 2H), 3.53 (br s, 1H), 2.79 (d, J = 16.4 Hz, 1H), 2.73 (d, J = 16.4 Hz, 1H), 1.54 (s, 3H), 1.34 (m, 3H).

^{13}C NMR (100 MHz, $CDCl_3$) δ 173.86, 116.01, 71.97, 63.09, 29.13, 25.55, 14.04.

IR (neat, cm^{-1}) 2933, 1730, 1444, 1105, 757.

HRMS (ESI) calcd $C_7H_{12}NO_3^+$ $[M+H]^+$: 158.0812. Found: 158.0812.

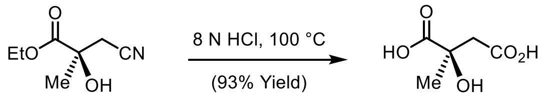

(-)-**S74** **74**, (–)-citramalic acid

A solution of ester (–)-**S74** (70 mg, 0.45 mmol) in 8 N HCl (2.0 mL) was stirred at 100 °C for 24 h. The solvent was then removed under vacuum. The remaining solid was washed with MeOH and the resulting solution was concentrated under vacuum to yield the (–)-citramalic acid **74** (61.3 mg, 93%) as a white solid. $[\alpha]_D^{25}$ = –14.7 (c = 1.0, H_2O). $[\alpha]_D^{13}$ lit = –21.0 (c = 1.0, H_2O).[7]

^{1}H NMR (500 MHz, MeOD) δ 2.94 (d, J = 16.0 Hz, 1H), 2.65 (d, J = 16.0 Hz, 1H), 1.44 (s, 3H).

^{13}C NMR (125 MHz, MeOD) δ 178.81, 174.28, 73.41, 45.10, 26.76.

IR (neat, cm^{-1}) 3535, 2933, 1730, 1447, 1184, 1105, 757.

HRMS (ESI) calcd $C_5H_8NaO_5^+$ $[M+Na]^+$: 171.0264. Found: 171.0266.

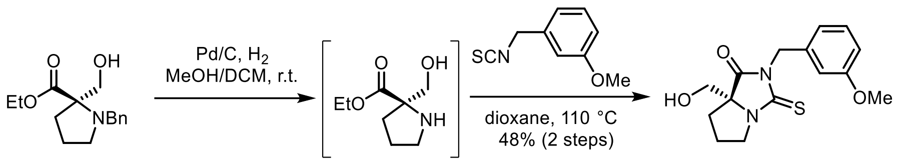

(+)-**11** **75**, (–)-macahydantoin B

To a stirred solution of (+)-**11** (24.0 mg, 0.091 mmol) in MeOH (3 mL) was added Pd/C (37 mg, 10% Pd/C with 55% H_2O). The mixture was stirred at room temperature under 1 atm hydrogen for 24 h. Then the mixture was filtered through Celite. The filtrate was concentrated under vacuum and the crude product was used in the next step without purification.

To a stirred solution of the above crude product in 1,4-dioxane (3 mL) was added isothiocyanate (32.6 mg, 0.182 mmol). The mixture was stirred at 110 °C for 48 h. The reaction mixture was diluted with water, extracted with EtOAc, washed with brine, dried over Na_2SO_4, and filtered. The filtrate was concentrated under vacuum and submitted to flash column chromatography (hexane/EtOAc = 1:1) to yield macahydantoin B (13.3 mg, 48% yield) as yellow solid. $[\alpha]_D^{25}$ = –88.7 (c = 1.0, $CHCl_3$).

Note: the NMR spectroscopies matched those reported in the literature.[8]

^{1}H NMR (400 MHz, $CDCl_3$) δ 7.19 (t, J = 8.0 Hz, 1H), 6.94-6.91 (m, 2H), 6.76 (dd, J = 8.0, 2.4 Hz, 1H), 4.94 (s, 2H), 4.10 (m, 1H), 3.83 (d, J = 11.6 Hz, 1H), 3.74 (s, 3H), 3.63 (d, J = 11.6 Hz, 1H), 3.43 (m, 1H), 3.03 (br s, 1H), 2.08-2.04 (m, 2H), 1.86-1.83 (m, 2H).

^{13}C NMR (100 MHz, $CDCl_3$) δ 187.53, 175.58, 159.50, 137.00, 129.39, 120.14, 113.22, 113.15, 75.15, 63.55, 55.12, 48.25, 44.94, 27.57, 25.36.

IR (neat, cm^{-1}) 3460, 2928, 1742, 1416, 1047, 566.

HRMS (ESI) calcd $C_{15}H_{19}N_2O_3S^+$ $[M+H]^+$: 307.1111. Found: 307.1109.

HPLC analysis (Chiralpak ID-3, hexane/^{i}PrOH = 70/30, 0.7 mL/min, 230 nm; t_r (major) = 13.56 min, t_r (minor) = 18.79 min) gave the isomeric composition of the product: 99% *e.e.*.

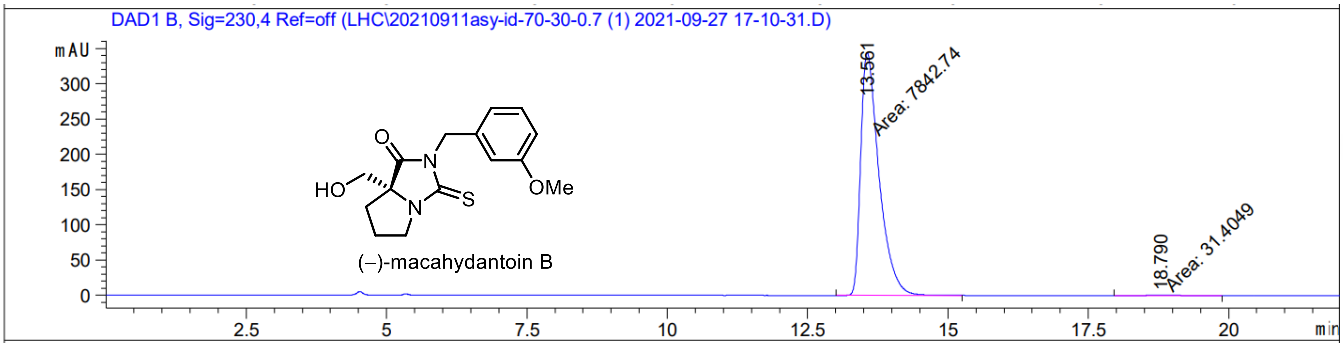

Signal 2: DAD1 B, Sig=230,4 Ref=off

Peak #	RetTime [min]	Type	Width [min]	Area [mAU*s]	Height [mAU]	Area %
1	13.561	MM	0.3794	7842.73877	344.56873	99.6012
2	18.790	MM	0.5749	31.40489	9.10374e-1	0.3988

Totals : 7874.14366 345.47910

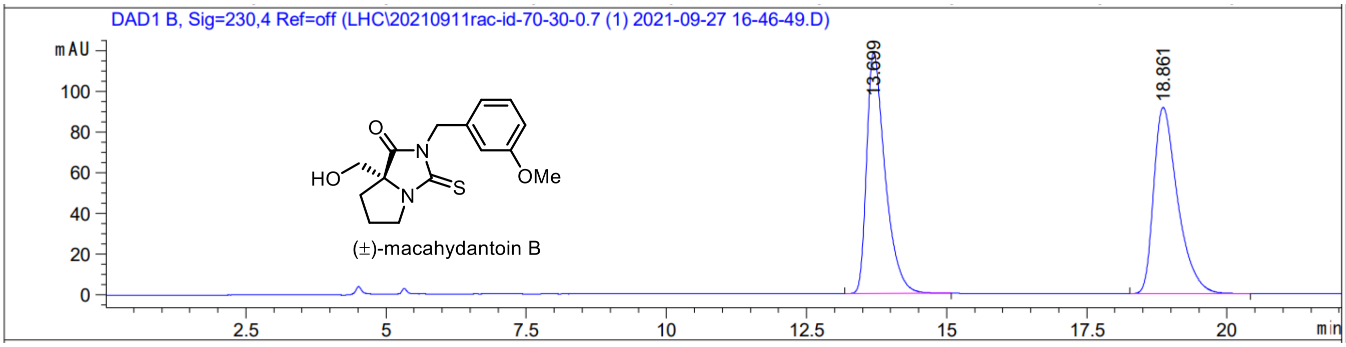

Signal 2: DAD1 B, Sig=230,4 Ref=off

Peak #	RetTime [min]	Type	Width [min]	Area [mAU*s]	Height [mAU]	Area %
1	13.699	BB	0.3436	2721.76245	118.65401	50.0488
2	18.861	BB	0.4484	2716.45923	91.49113	49.9512

Totals : 5438.22168 210.14514

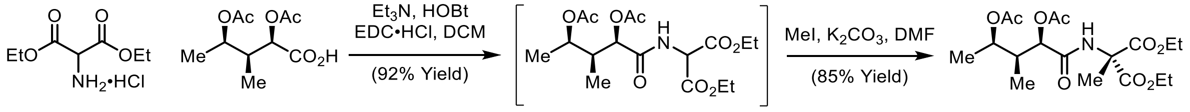

1 **78** (+)-**79**

To a stirred solution of diethyl aminomalonate hydrochloride (1.13 g, 5.35 mmol) and acid **78** (827 mg, 3.56 mmol) in DCM (14 mL) was added Et_3N (1.49 mL, 10.7 mmol), HOBt (722 mg, 5.35 mmol) and EDC•HCl (1.03 g, 5.35 mmol). The reaction mixture was stirred at room temperature overnight. The mixture was then diluted with water, extracted with EtOAc, washed with brine, dried over Na_2SO_4, and filtered. The filtrate was concentrated under vacuum and submitted to flash column chromatography (hexane/EtOAc = 3:1) to yield malonic esters (1.27 g, 92% yield) as colorless oil.

To a stirred solution of malonic esters (1.18 g, 3.03 mmol) in DMF (0.25 M) was added K_2CO_3 (828 mg, 6 mmol) and MeI (0.27 mL, 6.0 mmol). The resulting mixture was stirred at room temperature overnight. The reaction mixture was diluted with water, extracted with EtOAc, washed with brine, dried over Na_2SO_4,

and filtered. The filtrate was concentrated under vacuum and submitted to flash column chromatography (hexane/EtOAc = 3:1) to yield (+)-**79** as colorless oil (1.04 g, 85% yield). $[\alpha]_D^{25}$ = +10.2 (*c* = 1.0, $CHCl_3$).

Note: acid **78** (*d.r.* = 9:1) was synthesized following literature procedure.[9]

^{1}H NMR (400 MHz, $CDCl_3$) δ 7.34 (s, 1H), 5.17 (d, *J* = 3.6 Hz, 1H), 4.75 (m, 1H), 4.15-4.12 (m, 4H), 2.19 (m, 1H), 2.10 (s, 3H), 1.93 (s, 3H), 1.62 (s, 3H), 1.16-1.12 (m, 9H), 0.89 (m, 3H).

^{13}C NMR (100 MHz, $CDCl_3$) δ 169.94, 169.11, 168.07, 167.65, 167.34, 73.59, 71.20, 62.40, 62.19, 39.67, 20.77, 20.39, 20.12, 17.41, 13.52, 9.32.

IR (neat, cm^{-1}) 2983, 1734, 1444, 1215, 1018, 551.

HRMS (ESI) calcd $C_{18}H_{30}NO_9^+$ $[M+H]^+$: 404.1915. Found: 404.1941.

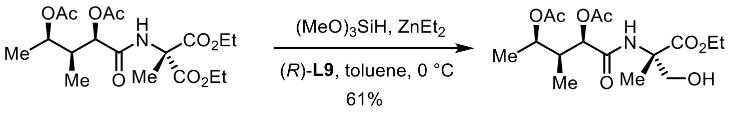

(+)-**79** (+)-**S80**

(+)-**S80** was obtained as colorless oil (110.0 mg, 61% Yield) from the reductive desymmetrization of malonic ester **S76** (200.0 mg, 0.50 mmol) with (*R*)-**L9** in toluene at 0 °C for 48 h using the general procedure C. R_f = 0.2 (hexane/EtOAc = 1:1). $[\alpha]_D^{25}$ = +24.1 (*c* = 1.0, $CHCl_3$).

^{1}H NMR (400 MHz, $CDCl_3$) δ 7.14 (s, 1H), 5.02 (d, *J* = 5.2 Hz, 1H), 4.97 (m, 1H), 4.23 (m, 2H), 4.12 (d, *J* = 10.8 Hz, 1H), 3.79 (d, *J* = 10.8 Hz, 1H), 3.25 (br s, 1H), 2.27 (m, 1H), 2.17 (s, 3H), 2.05 (s, 3H), 1.52 (s, 3H), 1.28 (t, *J* = 7.2 Hz, 3H), 1.24 (d, *J* = 6.4 Hz, 3H), 1.00 (d, *J* = 7.2 Hz, 3H).

^{13}C NMR (100 MHz, $CDCl_3$) δ 172.93, 170.88, 170.37, 168.78, 75.07, 71.06, 65.33, 62.29, 62.17, 39.63, 21.18, 20.75, 19.50, 17.92, 13.98, 9.6.

IR (neat, cm^{-1}) 3450, 2983, 1732, 1226, 1021, 755.

HRMS (ESI) calcd $C_{16}H_{28}NO_8^+$ $[M+H]^+$: 362.1809. Found: 362.1787.

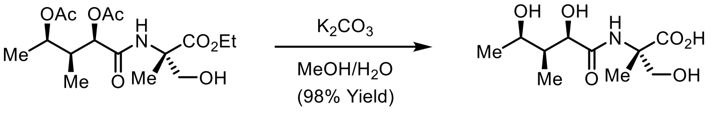

(+)-**S80** **80**, (+)-conagenin

To a stirred solution of (+)-**S80** (78.5 mg, 0.22 mmol) in MeOH (3 mL) was added K_2CO_3 (0.66 mL, 1.0

M in H_2O). The resulting mixture was stirred at room temperature overnight. The reaction mixture was added 2 N HCl (2.0 mL) and stirred for 10 min. Then the resulting mixture was extracted with EtOAc, washed with brine, dried over Na_2SO_4, and filtered. The filtrate was concentrated under vacuum and submitted to flash column chromatography (DCM/MeOH = 5:1) to yield (+)-conagenin (54.1 mg, 98% yield). $[\alpha]_D^{25}$ = +38.7 (*c* = 0.5, MeOH). $[\alpha]_{D\ lit.}^{25}$ = +48.4 (*c* = 0.4, MeOH).[8]

^{1}H NMR (400 MHz, MeOD) δ 4.17 (d, *J* = 2.4 Hz, 1H), 4.02 (d, *J* = 10.8 Hz, 1H), 3.86 (q, *J* = 6.0 Hz, 1H), 3.83 (d, *J* = 10.8 Hz, 1H), 1.90 (m, 1H), 1.51 (s, 3H), 1.22 (d, *J* = 6.4 Hz, 3H), 0.93 (d, *J* = 7.2 Hz, 3H).

^{13}C NMR (100 MHz, MeOD) δ 176.58, 175.75, 75.12, 71.13, 65.99, 62.49, 43.60, 21.17, 19.91, 8.27.

IR (neat, cm^{-1}) 3329, 2983, 2471, 1628, 1446, 1055, 545.

HRMS (ESI) calcd $C_{10}H_{20}NO_6^+$ [M+H]$^+$: 250.1285. Found: 250.1285.

Comparison of NMR data for reported and synthetic (+)-conagenin.[9,10]

δ_C (reported)	δ_C (synthetic)	δ_H (reported)	δ_H (synthetic)
176.9	176.6	4.15 (d, *J* = 2.4 Hz)	4.17 (d, *J* = 2.4 Hz)
175.8	175.8	4.04 (d, *J* = 11.0 Hz)	4.02 (d, *J* = 10.8 Hz)
75.1	75.1	3.85 (q, *J* = 6.1 Hz)	3.86 (q, *J* = 6.0 Hz)
71.2	71.1	3.84 (d, *J* = 11.0 Hz)	3.83 (d, *J* = 10.8 Hz)
66.1	66.0	1.89 (dq, *J* = 2.4, 6.3 Hz)	1.90 m
62.8	62.5	1.50 s	1.51 s
43.7	43.6	1.22 (d, *J* = 6.3 Hz)	1.22 (d, *J* = 6.4 Hz)
21.2	21.2	0.93 (d, *J* = 7.1 Hz)	0.93 (d, *J* = 7.2 Hz)
20.0	19.9		
8.4	8.3		

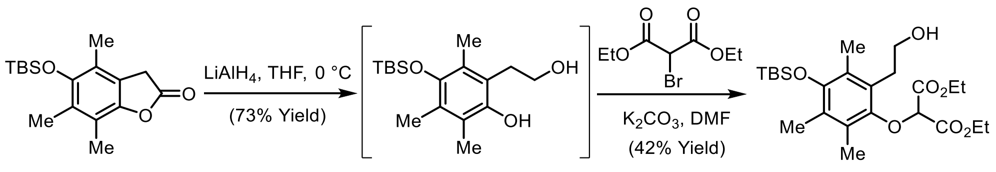

81 **S82** **82**

To a stirred solution of **81** (1.53 g, 5.0 mmol) in dry THF (0.25 M) was added $LiAlH_4$ (380 mg, 10.0 mmol) at 0 °C, which was then stirred at 0 °C for 2 h. The reaction mixture was quenched with saturated NH_4Cl solution and then the mixture was filtered through celite. The filtrate was concentrated under vacuum and submitted to flash column chromatography (hexane/EtOAc = 3:1) to yield **S82** as white solid (1.13 g, 73%

yield).

To a stirred solution of phenol **S82** (1.10 g, 3.5 mmol) in DMF (0.25 M) was added K_2CO_3 (966 mg, 5.25 mmol). After stirring for 10 min, diethyl bromomalonate (1.67 g, 7.0 mmol) was added dropwise and the reaction mixture was stirred at room temperature overnight. The mixture was then diluted with water, extracted with EtOAc, washed with brine, dried over Na_2SO_4, and filtered. The filtrate was concentrated under vacuum and submitted to flash column chromatography (hexane/EtOAc = 3:1) to yield **82** (688.0 mg, 42% yield).

Note: compound **81** was synthesized following literature procedure.[11]

^{1}H NMR (400 MHz, $CDCl_3$) δ 4.79 (s, 1H), 4.26 (m, 4H), 3.69 (t, *J* = 7.2 Hz, 2H), 2.90 (t, *J* = 7.2 Hz, 2H), 2.25 (br s, 1H), 2.12 (s, 3H), 2.08 (s, 3H), 2.05 (s, 3H), 1.26 (t, *J* = 7.2 Hz, 6H), 1.01 (s, 9H), 0.09 (s, 6H).

^{13}C NMR (100 MHz, $CDCl_3$) δ 166.42, 149.78, 148.61, 127.97, 127.28, 126.69, 125.75, 81.30, 62.33, 62.12, 31.07, 25.93, 18.50, 14.45, 14.04, 13.86, 13.64, -3.35.

IR (neat, cm^{-1}) 2933, 1742, 1241, 1089, 778.

HRMS (ESI) calcd $C_{24}H_{41}O_7Si^+$ $[M+H]^+$: 469.2616. Found: 469.2611.

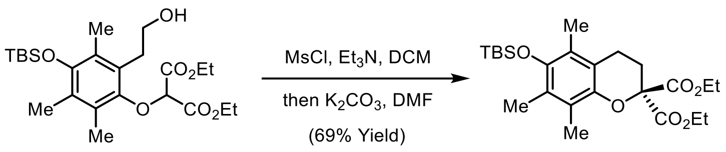

82 **83**

To a stirred solution of **82** (1.60 g, 3.4 mmol) in DCM (0.25 M) was added methanesulfonyl chloride (0.6 mL, 7.8 mmol) and Et_3N (1.4 mL, 10.2 mmol), which was then stirred at room temperature overnight. The reaction mixture was diluted with water, extracted with EtOAc, washed with brine, dried over Na_2SO_4, and filtered. The filtrate was concentrated under vacuum and the resulting crude product was used in the next step without purification.

To a stirred solution of the above crude product in DMF (0.25 M) was added K_2CO_3 (1.40 g, 10.2 mmol). The reaction mixture was stirred at room temperature overnight. The mixture was then diluted with water, extracted with EtOAc, washed with brine, dried over Na_2SO_4, and filtered. The filtrate was concentrated under vacuum and submitted to flash column chromatography (hexane/EtOAc = 5:1) to yield **83** as colorless oil (1.05 g, 69% yield).

^{1}H NMR (400 MHz, $CDCl_3$) δ 4.24 (m, 4H), 2.62 (t, *J* = 6.8 Hz, 2H), 2.44 (t, *J* = 6.8 Hz, 2H), 2.18 (s, 3H), 2.10 (s, 3H), 2.01 (s, 3H), 1.24 (t, *J* = 7.2 Hz, 6H), 1.03 (s, 9H), 0.10 (s, 6H).

^{13}C NMR (100 MHz, $CDCl_3$) δ 168.26, 145.35, 144.96, 126.35, 123.40, 123.08, 117.10, 80.84, 61.94, 26.82, 26.04, 20.25, 18.56, 14.26, 13.95, 13.28, 11.96, -3.40.

IR (neat, cm^{-1}) 2933, 1723, 1461, 1250, 1089, 836.

HRMS calcd $C_{24}H_{39}O_6Si^+$ $[M+H]^+$: 451.2510. Found: 451.2509.

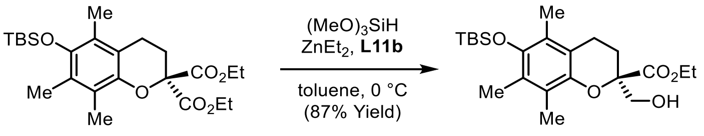

83 (–)-**84**

To an oven-dried 10 mL round bottom flask with a stir bar was added **L11b** (44.0 mg, 0.05 mmol), then the flask was sealed with a rubber septum and evacuated/refilled with nitrogen for three times. Freshly distilled toluene (1 mL) was added to the flask via syringe and the mixture was stirred at room temperature for 5 min. Diethylzinc (0.10 mL, 1.0 M solution in hexane, 0.10 mmol) was added to the flask via syringe slowly. The resulting catalyst solution was stirred at room temperature for 30 min before use.

To a separate oven-dried 5 mL Schlenk tube with a stir bar was added **83** (395 mg, 0.88 mmol), sealed with a rubber septum, and evacuated/refilled with nitrogen for three times. Trimethoxysilane (321 mg, 2.63 mmol) and freshly distilled toluene (8.0 mL) were added via syringe. The mixture was stirred at room temperature while 0.88 mL of aforementioned catalyst solution was added via syringe. The reaction mixture was stirred at 0 °C for 24 h, monitored by TLC. After the starting material was consumed, 0.2 mL triethylamine trihydrofluoride was added dropwise to quench the reaction. The mixture was diluted with 5 mL diethyl ether, and stirred for 30 min. Subsequently, the reaction mixture was filtered through a short pad of silica gel, eluted with diethyl ether slowly (as the remaining triethylamine trihydrofluoride reacts with silica gel to release heat). The filtrate was evaporated and purified by flash column chromatography (hexanes/ethyl acetate = 5:1) to yield (–)-**84** (310.6 mg, 87%) as colorless oil. $[\alpha]_D^{25}$ = –1.45 (c = 1.0, $CHCl_3$).

^{1}H NMR (400 MHz, $CDCl_3$) δ 4.15 (q, J = 7.2 Hz, 2H), 3.96 (dd, J = 11.2, 8.4 Hz, 1H), 3.85 (dd, J = 11.2, 4.8 Hz, 1H), 2.64 (m, 1H), 2.46 (m, 1H), 2.35-2.29 (m, 2H), 2.16 (s, 3H), 2.11 (s, 3H), 2.02 (s, 3H), 2.00-1.95 (m, 1H), 1.17 (t, J = 7.2 Hz, 3H), 1.04 (s, 9H), 0.11 (s, 3H), 0.11 (s, 3H).

^{13}C NMR (100 MHz, $CDCl_3$) δ 171.93, 145.37, 145.04, 126.26, 123.55, 122.48, 117.23, 80.41, 67.76, 61.33, 26.05, 25.53, 20.35, 18.57, 14.29, 14.06, 13.34, 12.03, -3.36, -3.41.

IR (neat, cm^{-1}) 2938, 1746, 1462, 1248, 1089, 778.

HRMS (ESI) calcd $C_{22}H_{37}O_5Si^+$ $[M+H]^+$: 409.2405. Found: 409.2404.

HPLC analysis (Chiralpak IF-3, hexane/^{i}PrOH = 97/3, 1.0 mL/min, 230 nm; t_r (major) = 9.26 min, t_r (minor) = 11.06 min) gave the isomeric composition of the product: 80% *e.e.*.

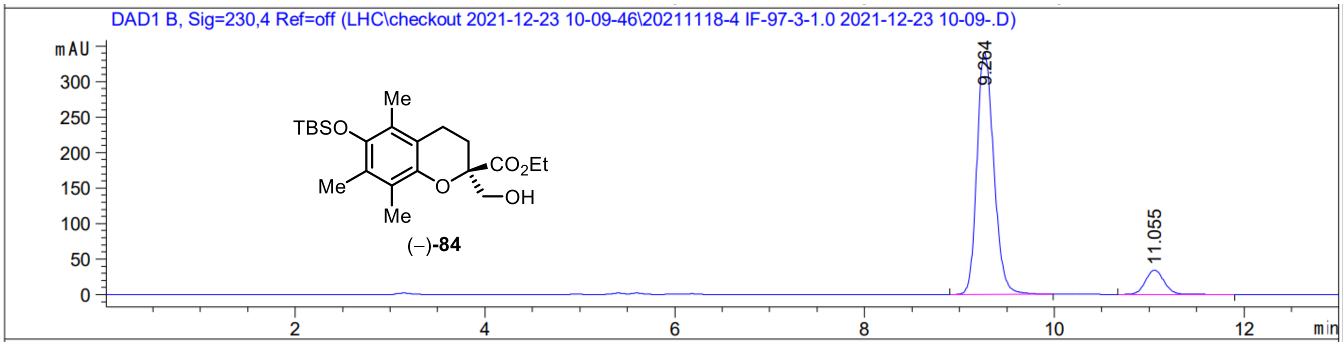

Signal 2: DAD1 B, Sig=230,4 Ref=off

Peak #	RetTime [min]	Type	Width [min]	Area [mAU*s]	Height [mAU]	Area %
1	9.264	BB	0.1916	4206.26807	341.68503	89.9870
2	11.055	BB	0.2108	468.03931	34.37884	10.0130
Totals :				4674.30737	376.06387	

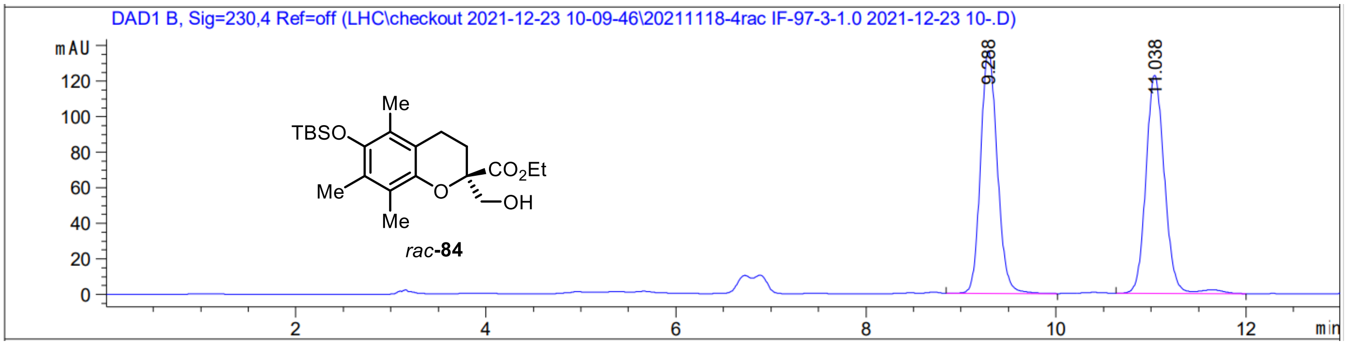

Signal 2: DAD1 B, Sig=230,4 Ref=off

Peak #	RetTime [min]	Type	Width [min]	Area [mAU*s]	Height [mAU]	Area %
1	9.288	BB	0.1905	1665.67493	136.35959	49.7426
2	11.038	BV R	0.2082	1682.91028	122.72286	50.2574
Totals :				3348.58521	259.08245	

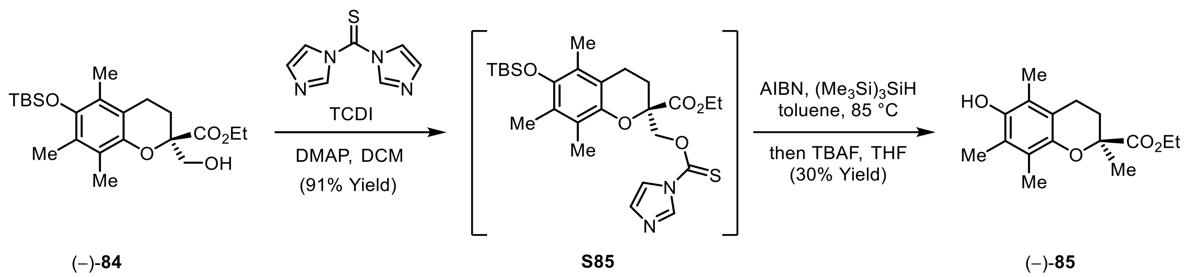

To a stirred solution of (–)-**84** (264.0 mg, 0.65 mmol) in DCM (4.0 mL) was added thiocarbonyl diimidazole (TCDI, 231.4 mg, 1.30 mmol) and DMAP (79.3 mg, 0.65 mmol), which was then stirred at room temperature overnight. The reaction mixture was diluted with water, extracted with EtOAc, washed with brine, dried over Na_2SO_4, and filtered. The filtrate was evaporated and purified by flash column chromatography (hexanes/ethyl acetate = 5:1) to yield **S85** (306.0 mg, 91%) as colorless oil.

To a stirred solution of the above product in dry toluene (3.0 mL) was added $(Me_3Si)_3SiH$ (879 mg, 3.54 mmol) and AIBN (30.0 mg, 0.18 mmol). The reaction mixture was stirred at 85 ℃ for 4 h. The mixture was then quenched with saturated $NaHCO_3$ solution, extracted with EtOAc, washed with brine, dried over Na_2SO_4, and filtered. The filtrate was concentrated under vacuum and submitted to flash column chromatography (hexane/EtOAc = 30:1) to yield crude product tro4. Then tro4 was dissolved in THF (2 mL) and TBAF (0.6 mL, 1.0 M in THF) was added and stirred at room temperature 10 min. The resulting mixture solution was concentrated under vacuum and purified by flash column chromatography (hexanes/ethyl acetate = 5:1) to yield the (–)-**85** (49.3 mg, 30%) as white solid and the product gave 96% *e.e.* value after recrystallization. $[\alpha]_D^{25}$ = –17.4 (c = 1.0, $CHCl_3$).

^{1}H NMR (400 MHz, $CDCl_3$) δ 4.37 (s, 1H), 4.12 (m, 2H), 2.65 (m, 1H), 2.56-2.42 (m, 2H), 2.19 (s, 3H), 2.15 (s, 3H), 2.05 (s, 3H), 1.88 (m, 1H), 1.60 (s, 3H), 1.20 (t, J = 7.2 Hz, 3H).

^{13}C NMR (100 MHz, $CDCl_3$) δ 173.84, 145.57, 145.21, 122.50, 121.19, 118.39, 116.85, 76.85, 60.98, 30.53, 25.28, 20.91, 14.03, 12.14, 11.74, 11.17.

IR (neat, cm^{-1}) 3535, 2933, 1730, 1447, 1184, 1105, 757.

HRMS (ESI) calcd $C_{16}H_{23}O_4^+$ $[M+H]^+$: 279.1591. Found: 279.1591.

HPLC analysis (Chiralpak IF-3, hexane/^{i}PrOH = 97/3, 1.0 mL/min, 205 nm; t_r (minor) = 10.62 min, t_r (major) = 11.03 min) gave the isomeric composition of the product: 96% *e.e.*.

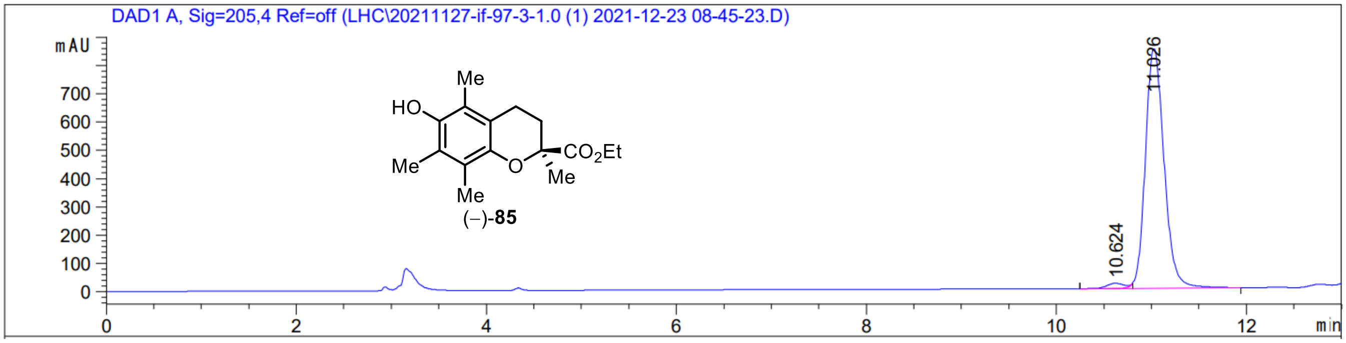

Signal 1: DAD1 A, Sig=205,4 Ref=off

Peak #	RetTime [min]	Type	Width [min]	Area [mAU*s]	Height [mAU]	Area %
1	10.624	BV E	0.1891	219.75893	18.16903	1.8611
2	11.026	VB R	0.2137	1.15881e4	846.42621	98.1389

Totals : 1.18078e4 864.59524

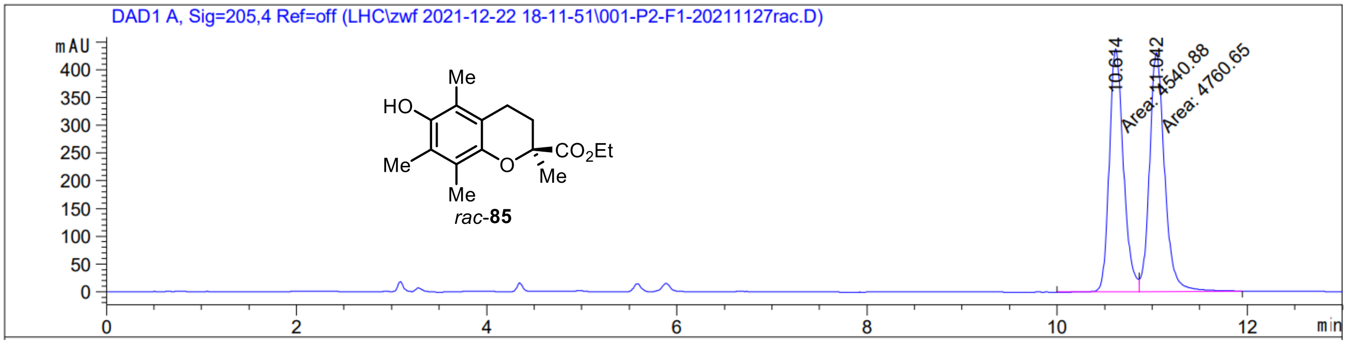

Signal 1: DAD1 A, Sig=205,4 Ref=off

Peak #	RetTime [min]	Type	Width [min]	Area [mAU*s]	Height [mAU]	Area %
1	10.614	MF	0.1726	4540.87695	438.54102	48.8186
2	11.042	FM	0.1839	4760.64990	431.51401	51.1814

Totals : 9301.52686 870.05502

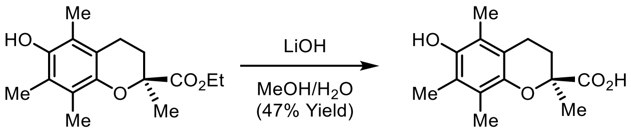

(–)-**85** **86**, (–)-Trolox

To a stirred solution of (–)-**85** (17.6 mg, 0.063 mmol) in MeOH/H_2O (0.9 mL/0.3 mL) was added LiOH•H_2O (7.4 mg, 0.19 mmol). The resulting mixture was stirred at room temperature overnight. The reaction mixture was added 2 N $NaHSO_4$ (0.2 mL) and stirred for 10 min. Then the resulting mixture was extracted with EtOAc, washed with brine, dried over Na_2SO_4, and filtered. The filtrate was concentrated under vacuum and submitted to flash column chromatography (hexanes/EtOAc = 1:1) to yield Trolox (7.4 mg, 47%). $[\alpha]_D^{25}$ = –32.2 (*c* = 0.4, EtOH). $[\alpha]_{D\ lit.}^{25}$ = –54.9 (*c* = 1.01, EtOH)[8].

Note: the NMR data matched those reported in the literature.[12]

^{1}H NMR (500 MHz, $CDCl_3$) δ 2.66-2.61 (m, 2H), 2.40-2.35 (m, 1H), 2.17 (s, 6H), 2.09 (s, 3H), 1.95-1.91 (m, 1H), 1.61 (s, 3H).

^{13}C NMR (125 MHz, $CDCl_3$) δ 176.65, 145.76, 144.48, 122.47, 121.49, 118.68, 117.12, 70.09, 29.95, 24.33, 20.54, 12.19, 11.88, 11.27.

IR (neat, cm^{-1}) 2922, 1703, 1455, 1138, 943.

HRMS (ESI) calcd $C_{13}H_{15}NO_2^+$ $[M+H]^+$: 251.1278, Found: 251.1271.

1.6 Copies of NMR Spectra

Supplementary Fig. 1. ^{1}H NMR of compound II (CDCl$_3$, 400 MHz, 25 °C)

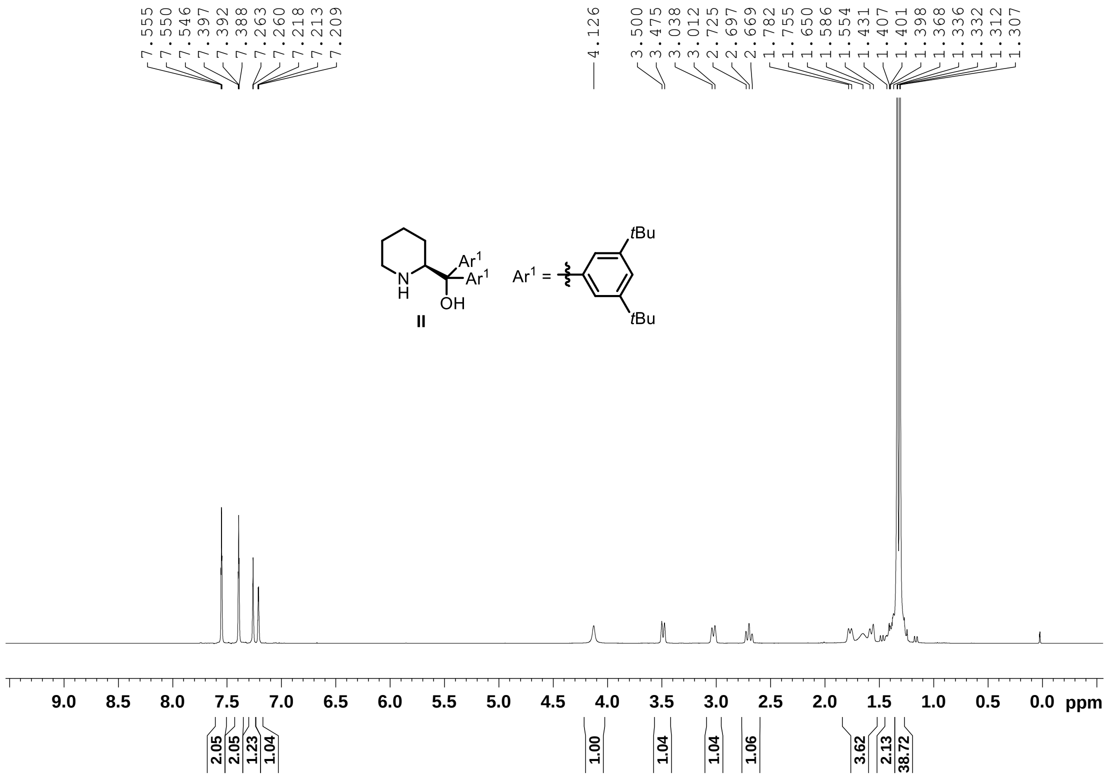

Supplementary Fig. 2. ^{13}C NMR of compound II (CDCl$_3$, 100 MHz, 25 °C)

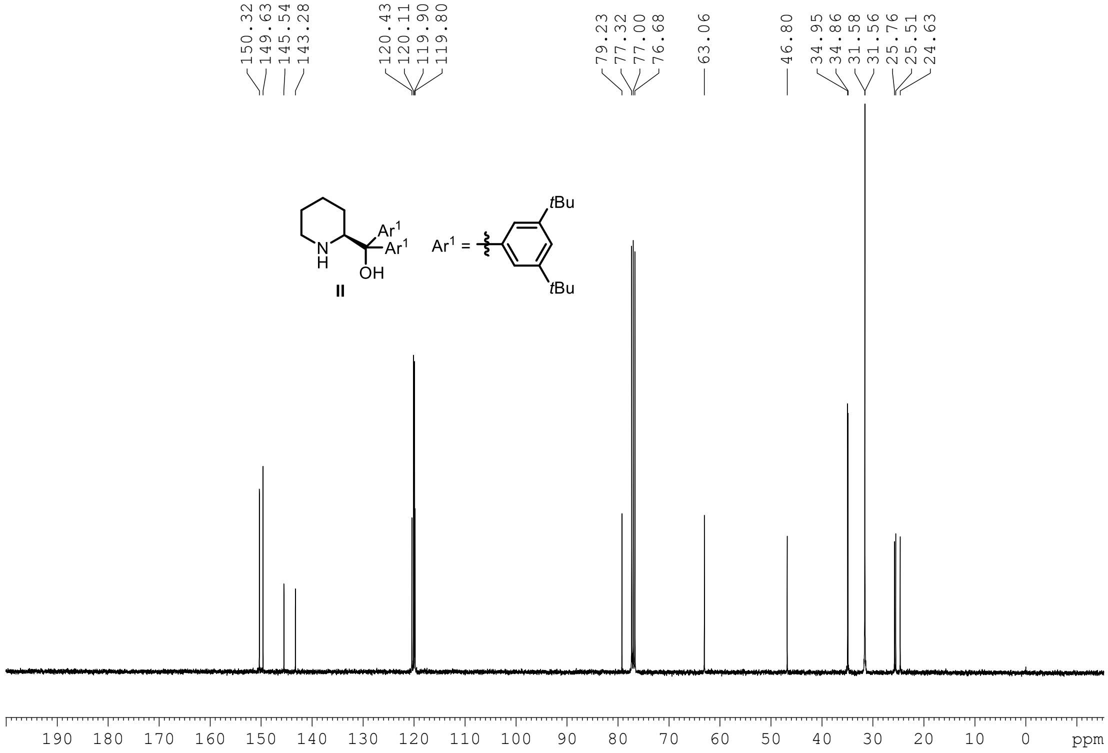

Supplementary Fig. 3. ^{1}H NMR of compound IV ($CDCl_3$, 400 MHz, 25 °C)

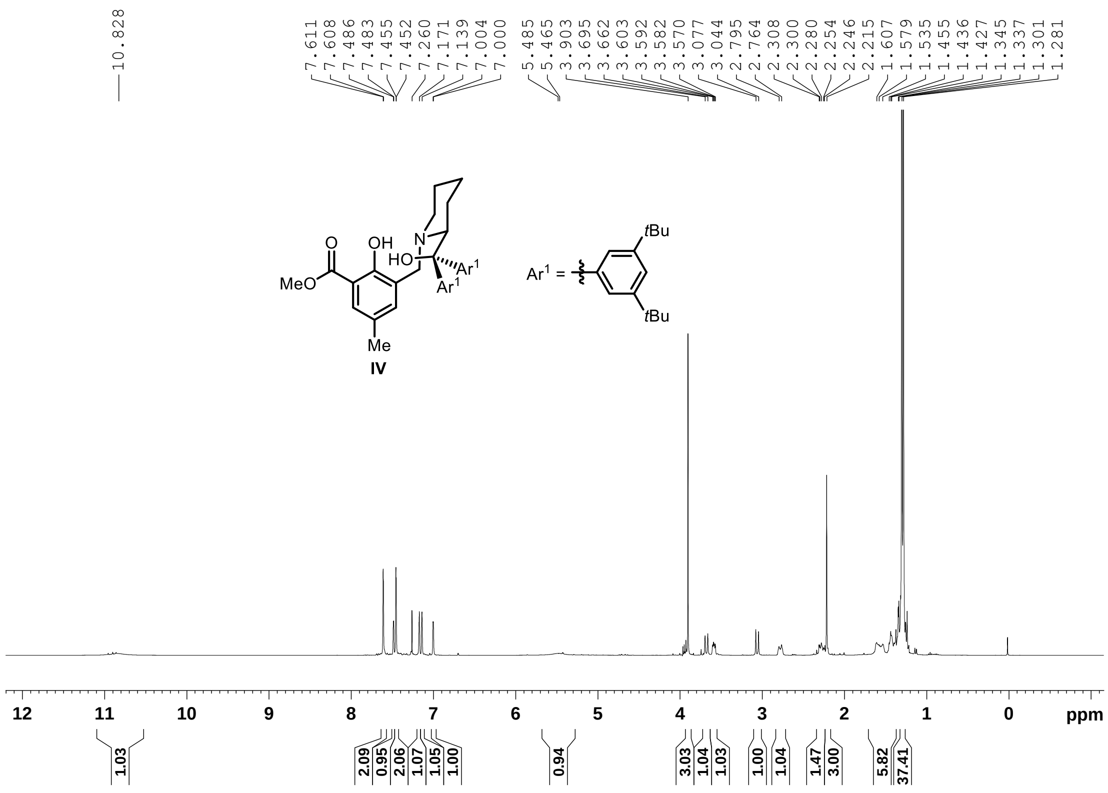

Supplementary Fig. 4. ^{13}C NMR of compound IV ($CDCl_3$, 100 MHz, 25 °C)

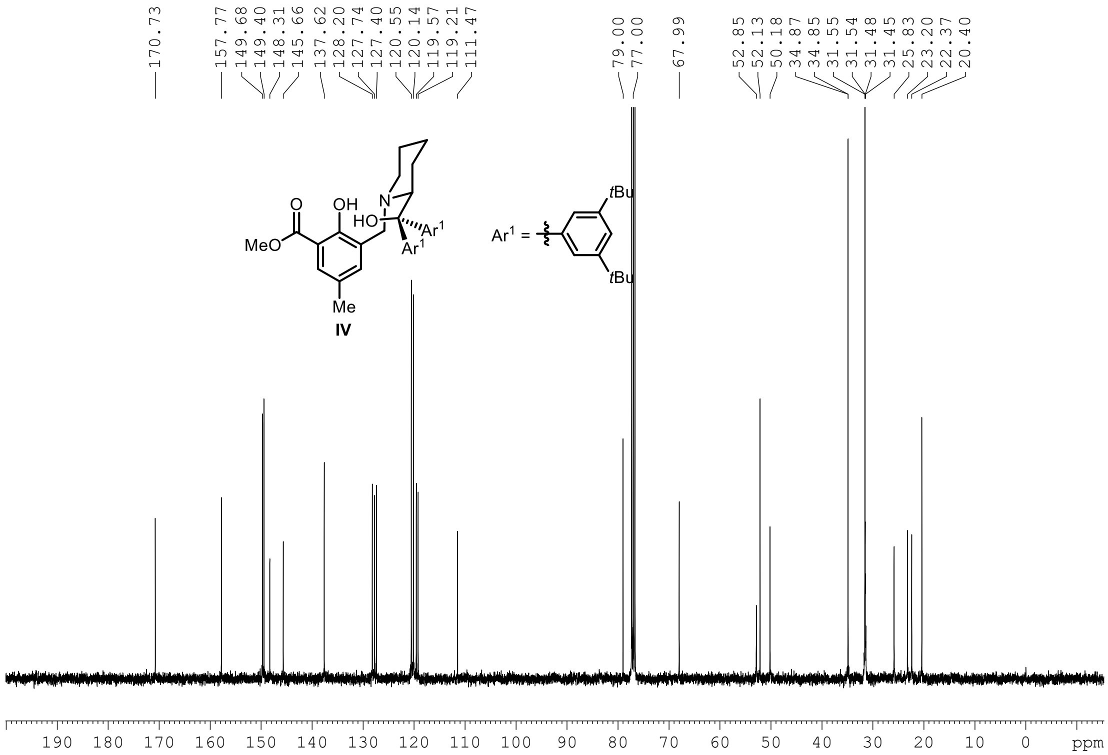

Supplementary Fig. 5. ^{1}H NMR of L7 ($CDCl_3$, 400 MHz, 25 °C)

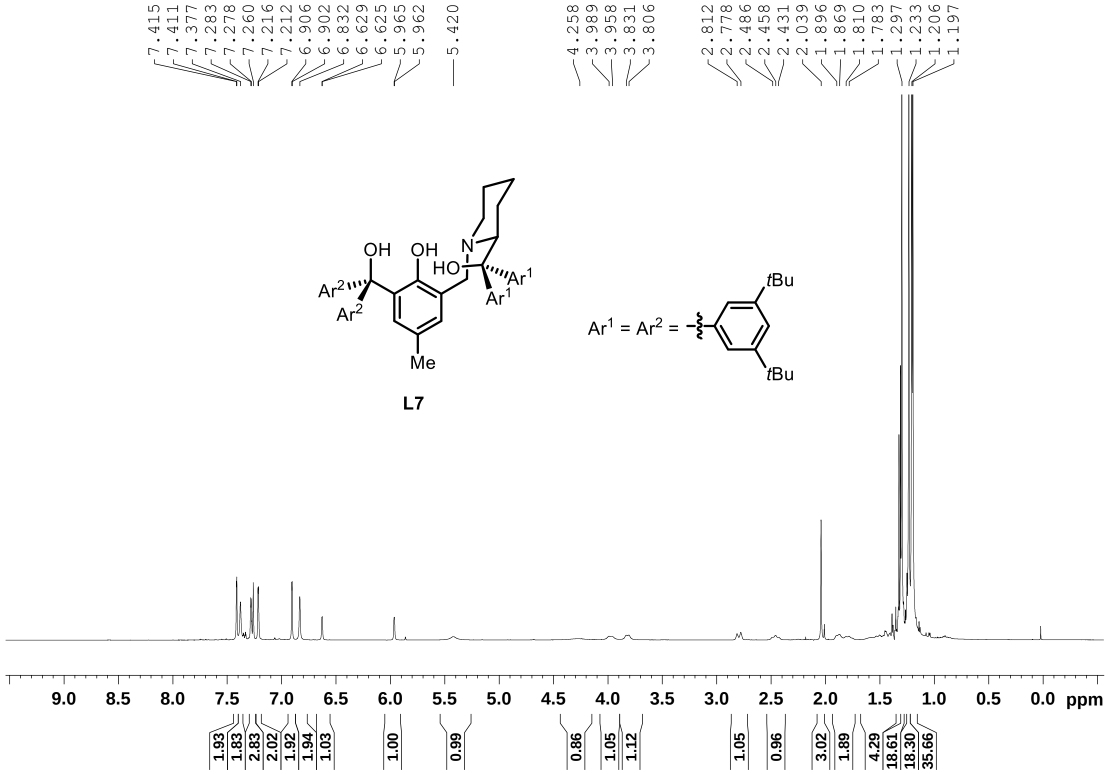

Supplementary Fig. 6. ^{13}C NMR of L7 ($CDCl_3$, 100 MHz, 25 °C)

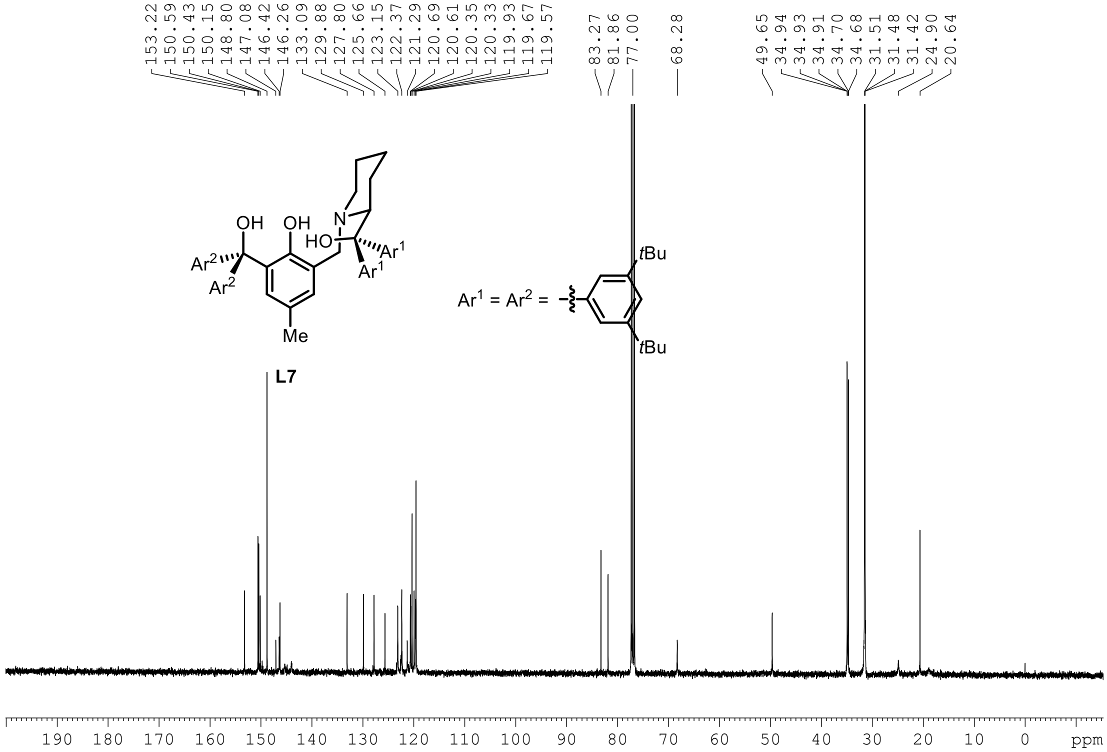

Supplementary Fig. 7. ^{1}H NMR of L9 ($CDCl_3$, 400 MHz, 25 °C)

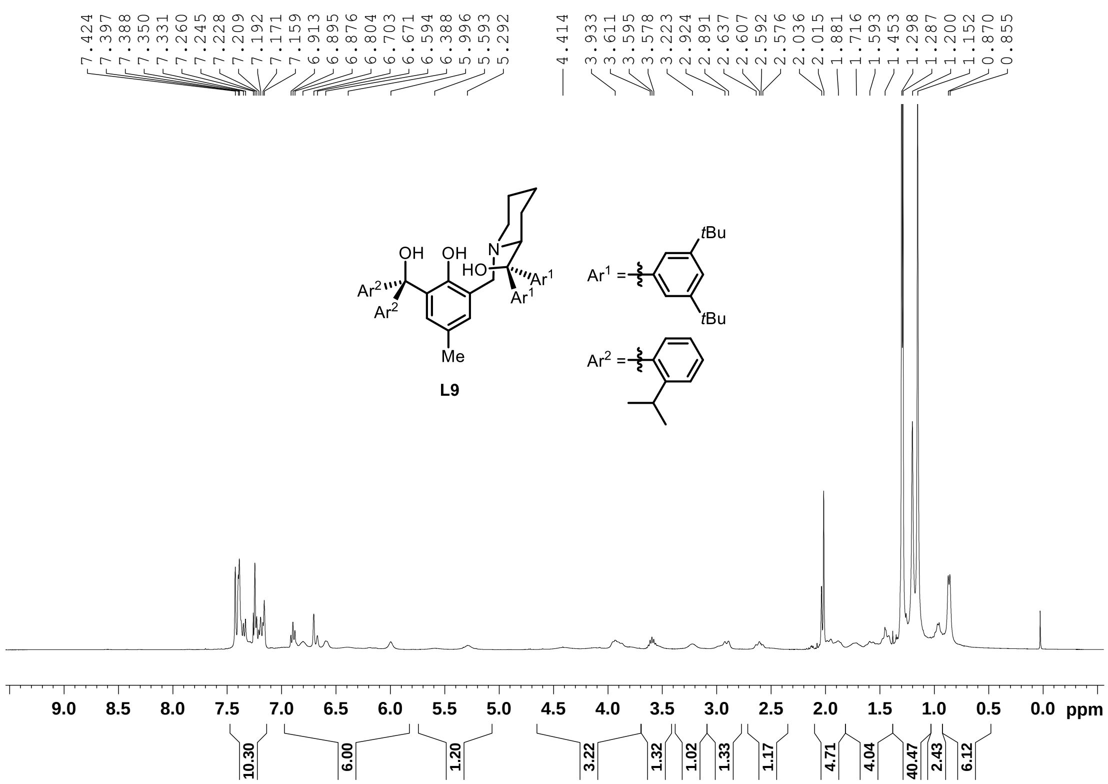

Supplementary Fig. 8. ^{13}C NMR of L9 ($CDCl_3$, 100 MHz, 25 °C)

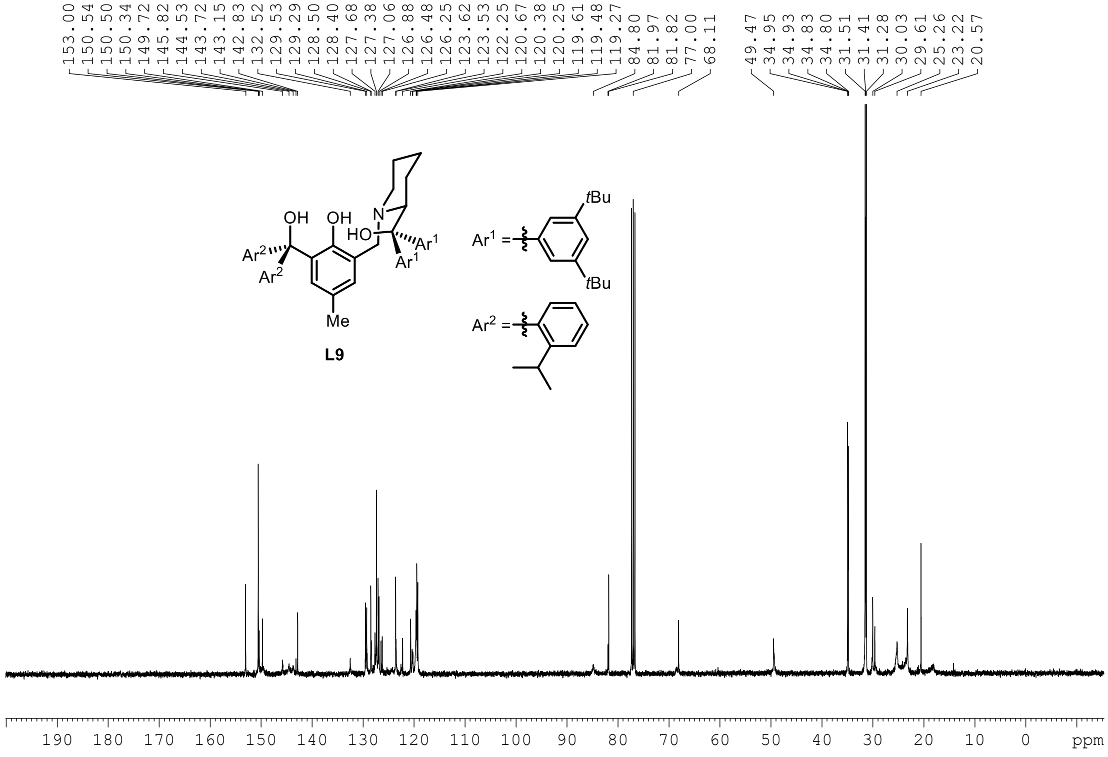

Supplementary Fig. 9. ^{1}H NMR of L10 ($CDCl_3$, 400 MHz, 25 °C)

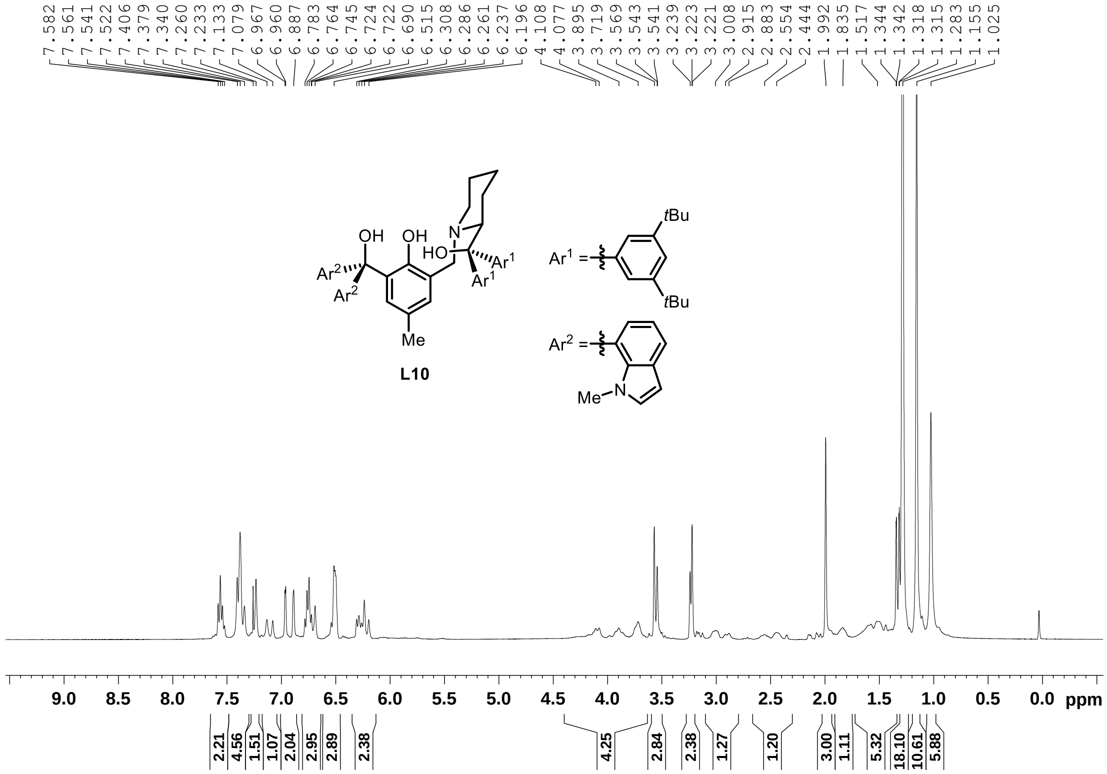

Supplementary Fig. 10. ^{13}C NMR of L10 ($CDCl_3$, 100 MHz, 25 °C)

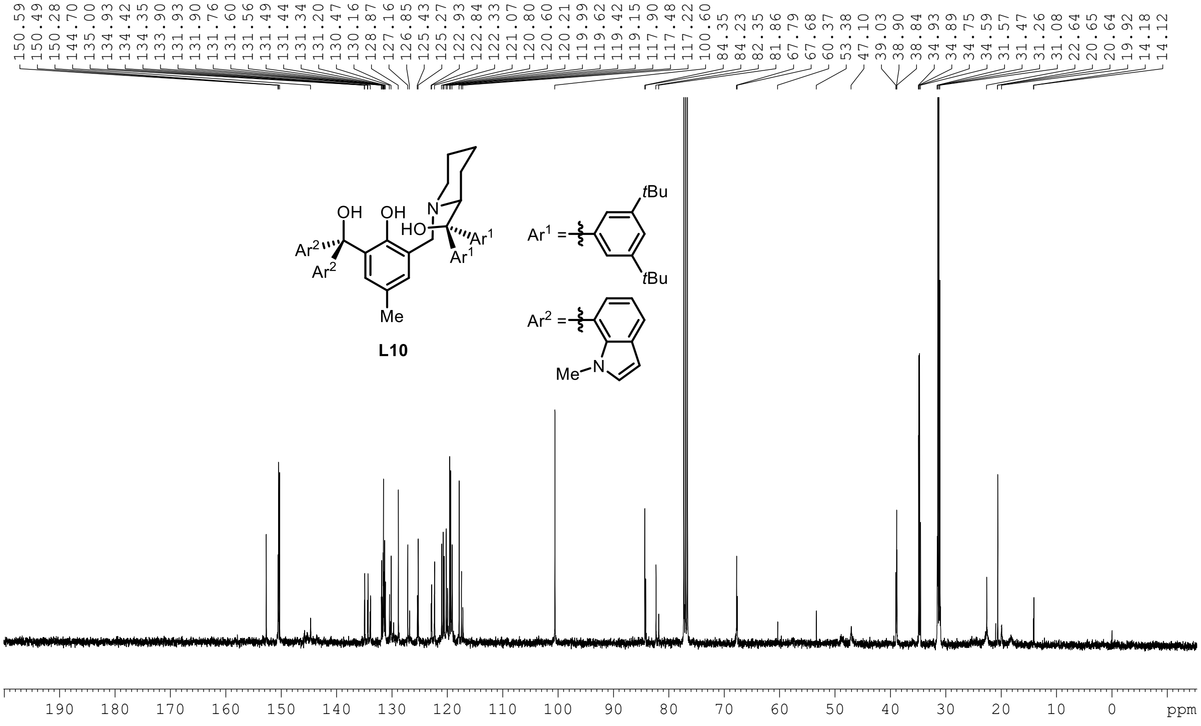

Supplementary Fig. 11. ^{1}H NMR of VI (CDCl$_3$, 400 MHz, 25 °C)

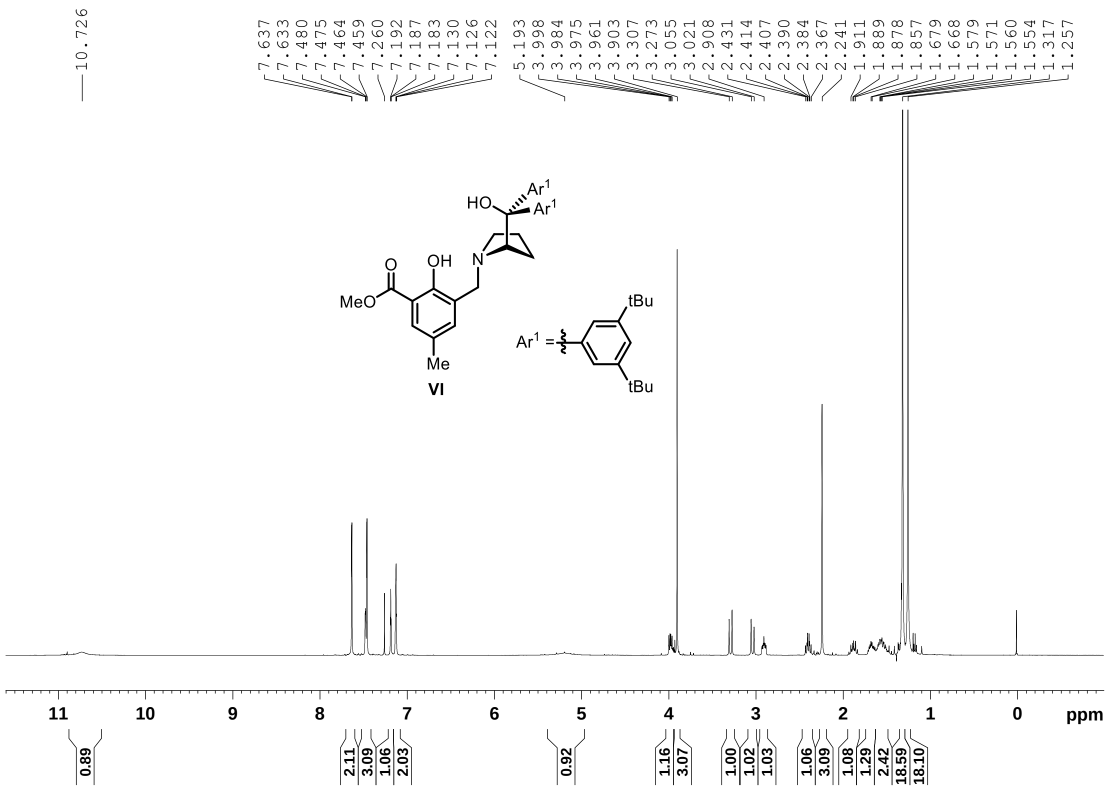

Supplementary Fig. 12. ^{13}C NMR of VI (CDCl$_3$, 100 MHz, 25 °C)

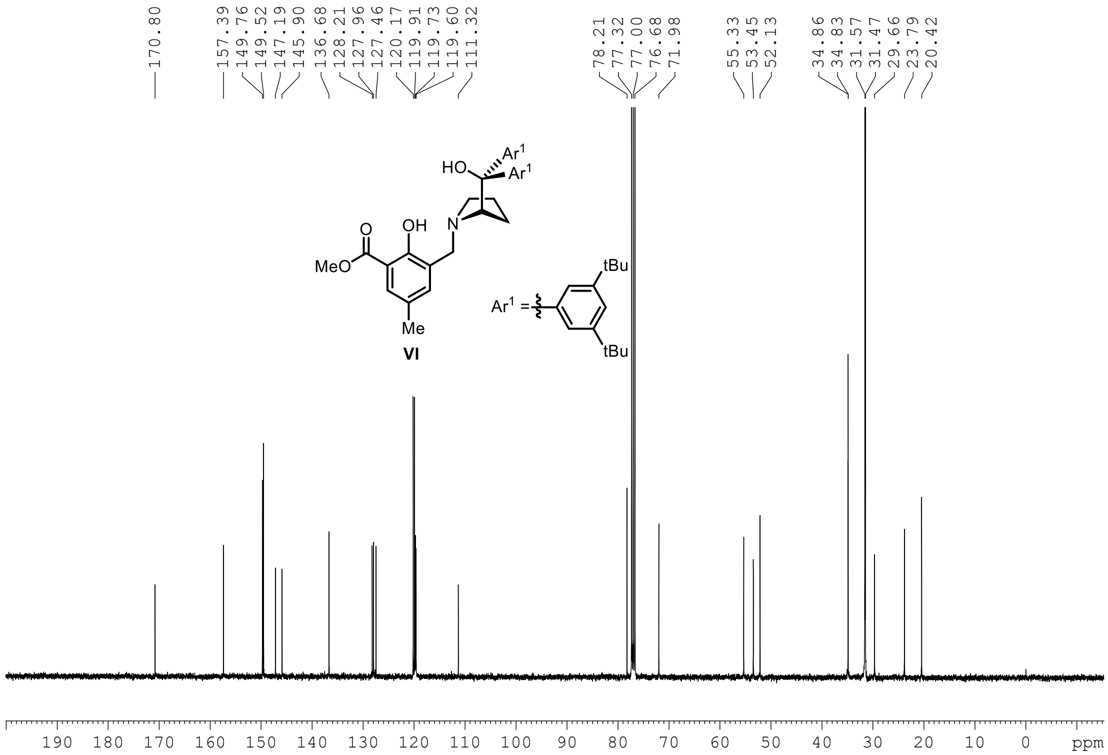

Supplementary Fig. 13. ¹H NMR of L11a (CDCl₃, 400 MHz, 25 °C)

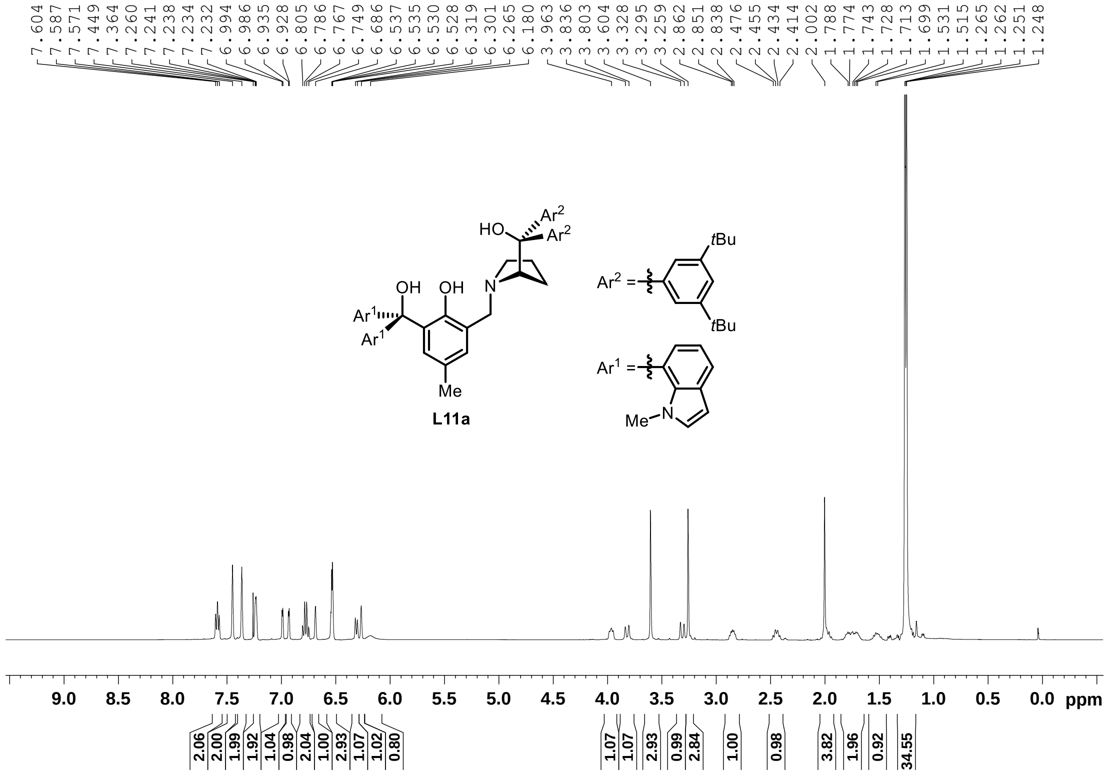

Supplementary Fig. 14. ¹³C NMR of L11a (CDCl₃, 100 MHz, 25 °C)

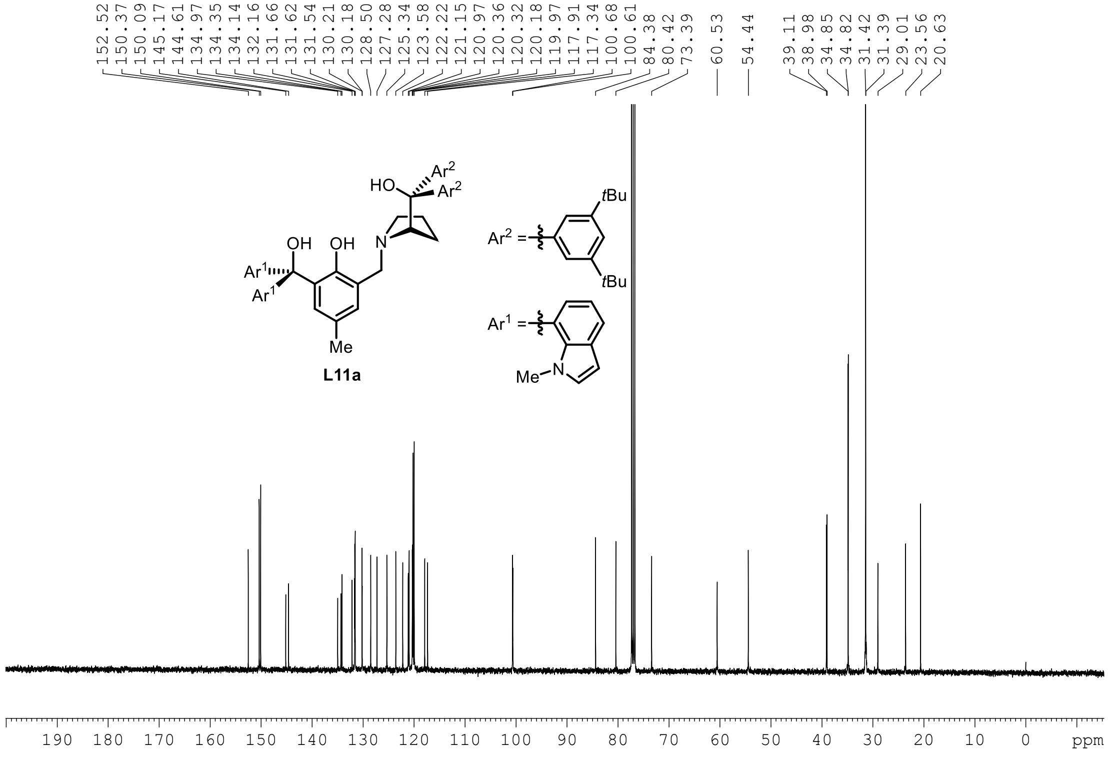

Supplementary Fig. 15. ¹H NMR of L11b (CDCl₃, 400 MHz, 25 °C)

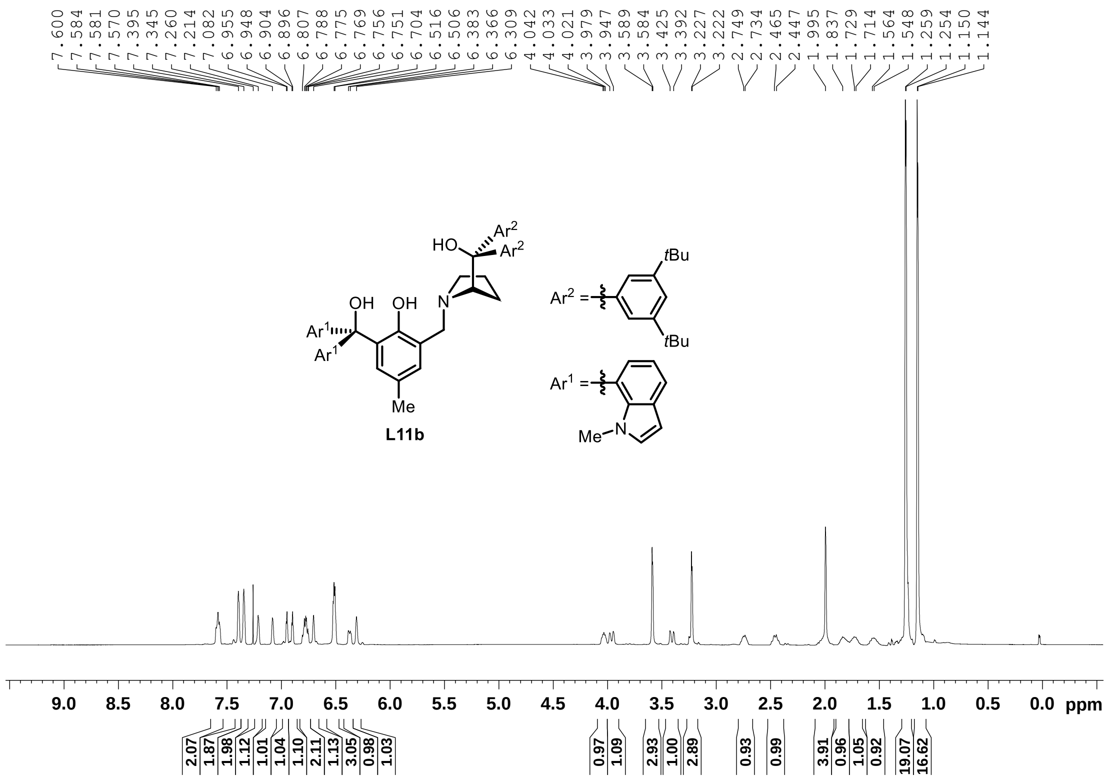

Supplementary Fig. 16. ¹³C NMR of compound 11b (CDCl₃, 100 MHz, 25 °C)

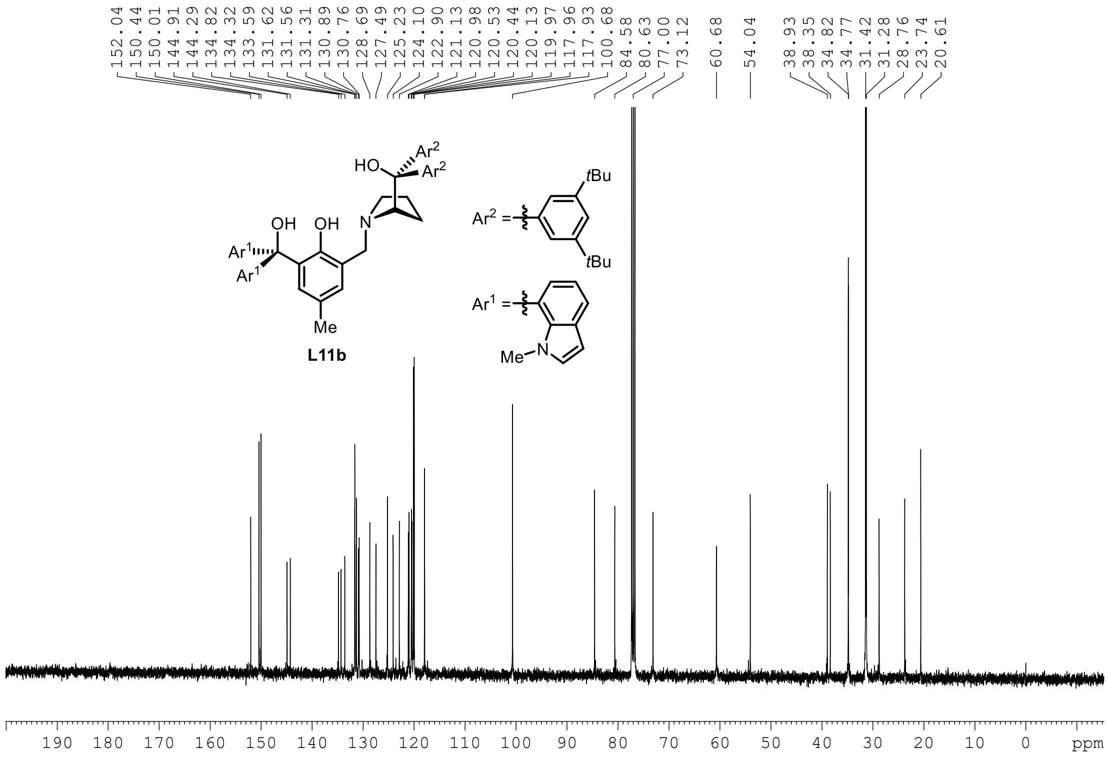

Supplementary Fig. 17. ^{1}H **NMR of compound S9 ($CDCl_3$, 400 MHz, 25 °C)**

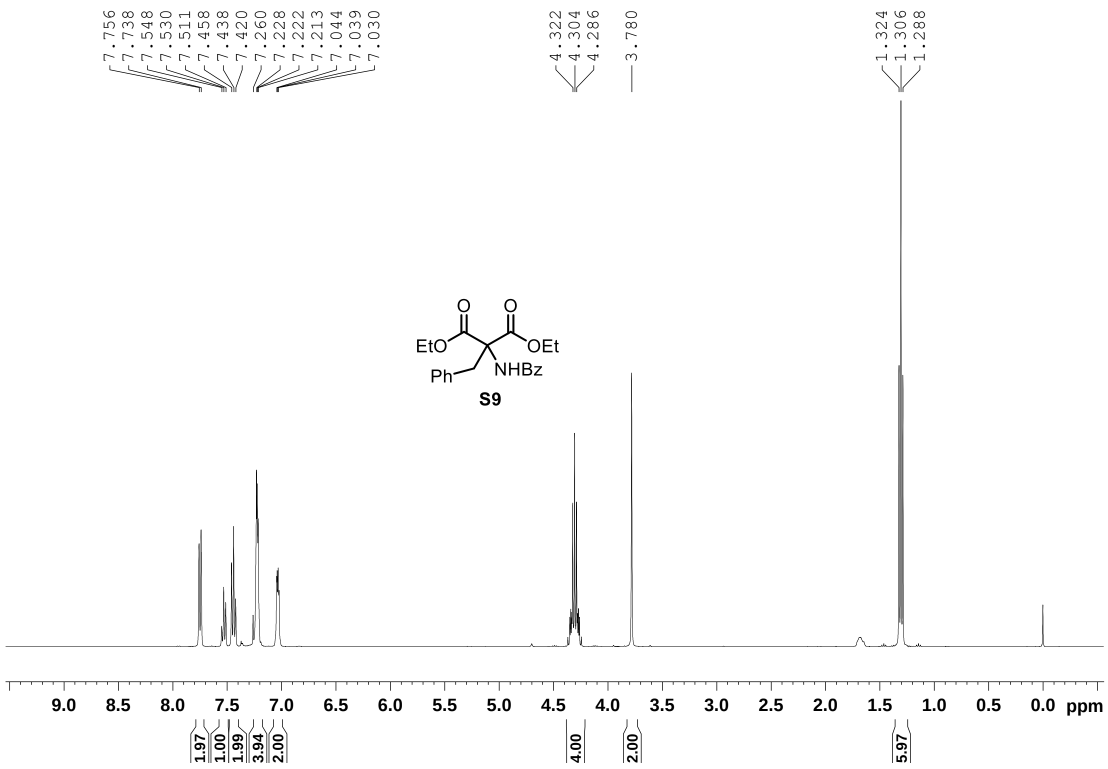

Supplementary Fig. 18. ^{13}C **NMR of compound S9 ($CDCl_3$, 100 MHz, 25 °C)**

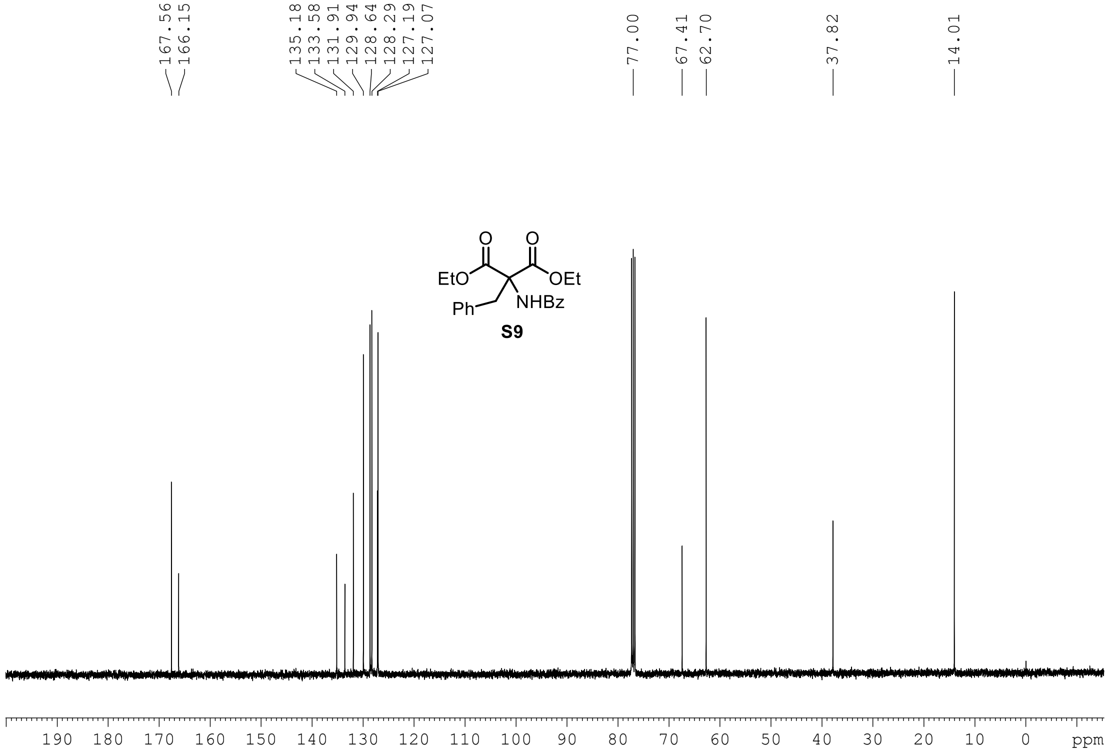

Supplementary Fig. 19. ^{1}H NMR of compound S15 ($CDCl_3$, 400 MHz, 25 °C)

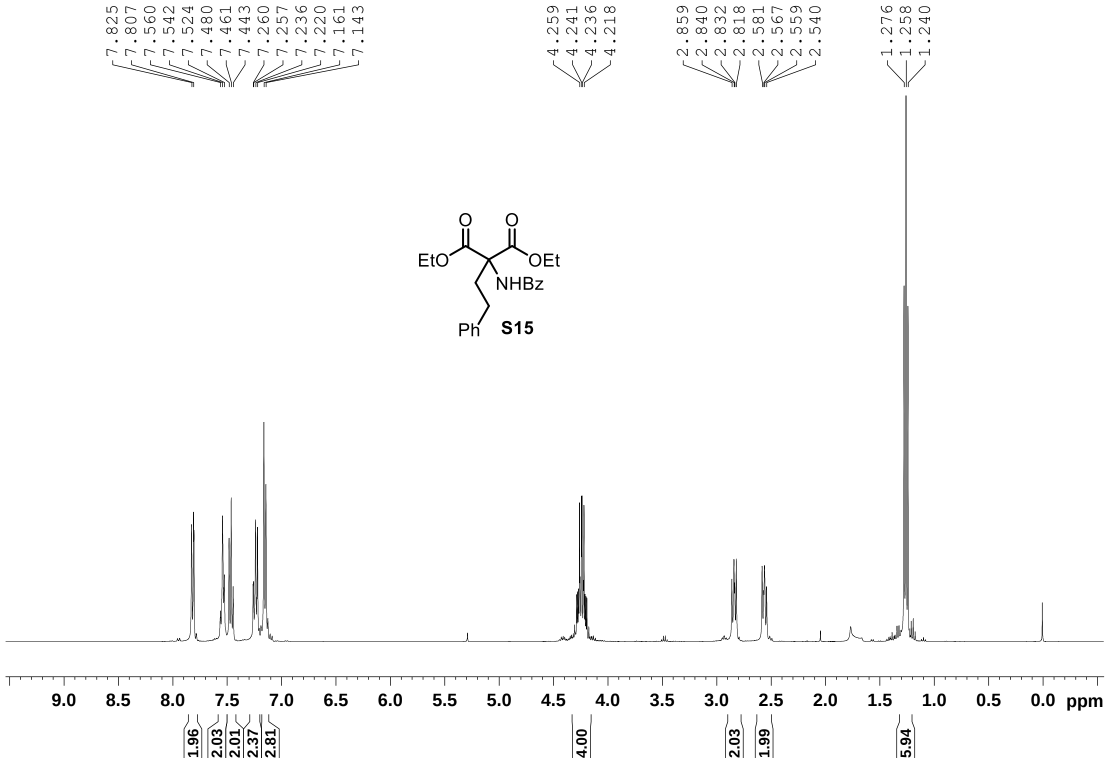

Supplementary Fig. 20. ^{13}C NMR of compound S15 ($CDCl_3$, 100 MHz, 25 °C)

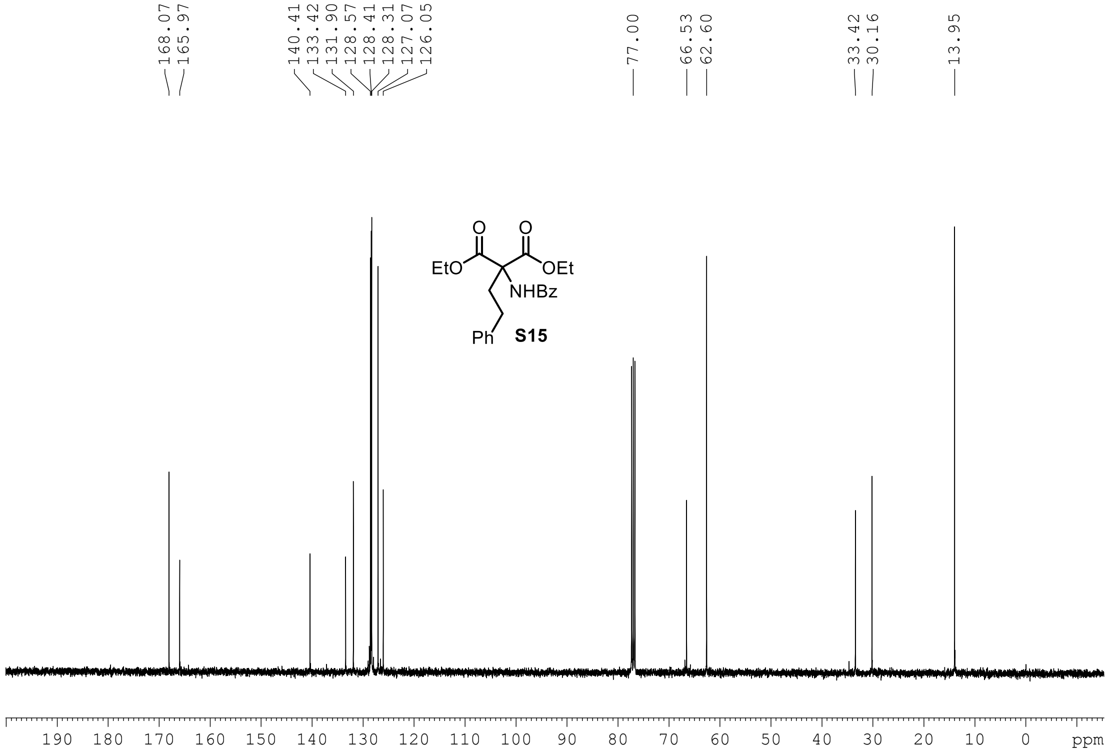

Supplementary Fig. 21. ^{1}**H NMR of compound S16 (**$CDCl_3$**, 400 MHz, 25 °C)**

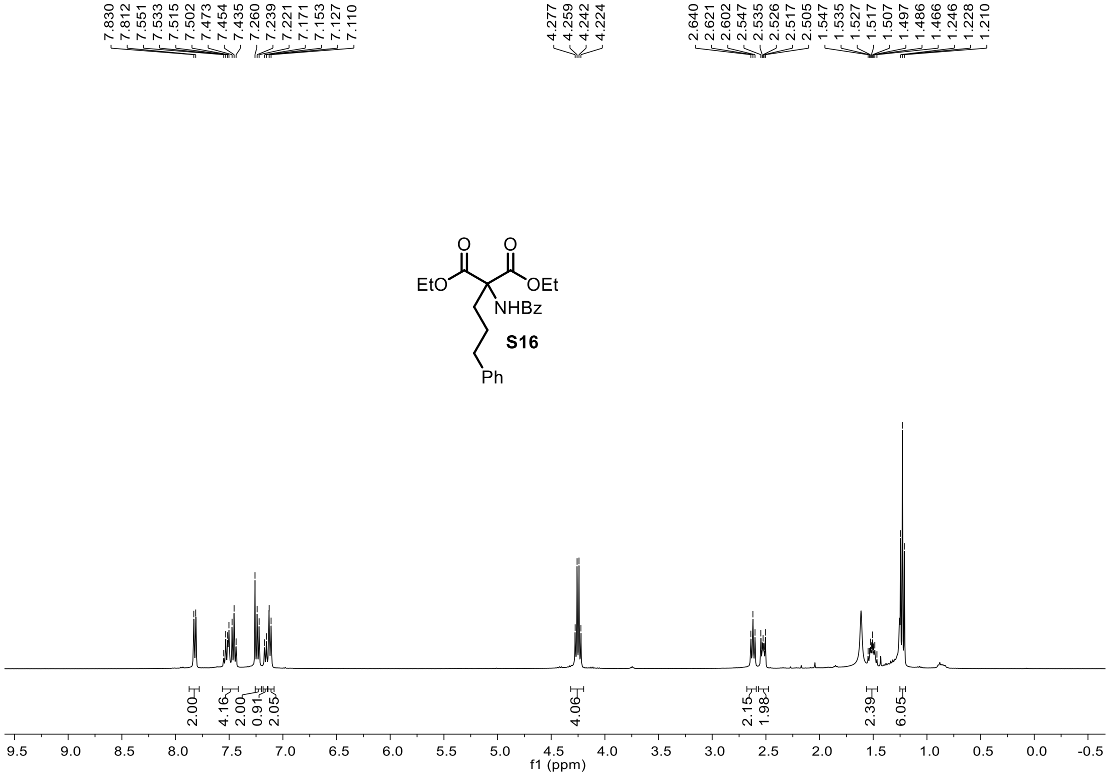

Supplementary Fig. 22. ^{13}**C NMR of compound S16 (**$CDCl_3$**, 100 MHz, 25 °C)**

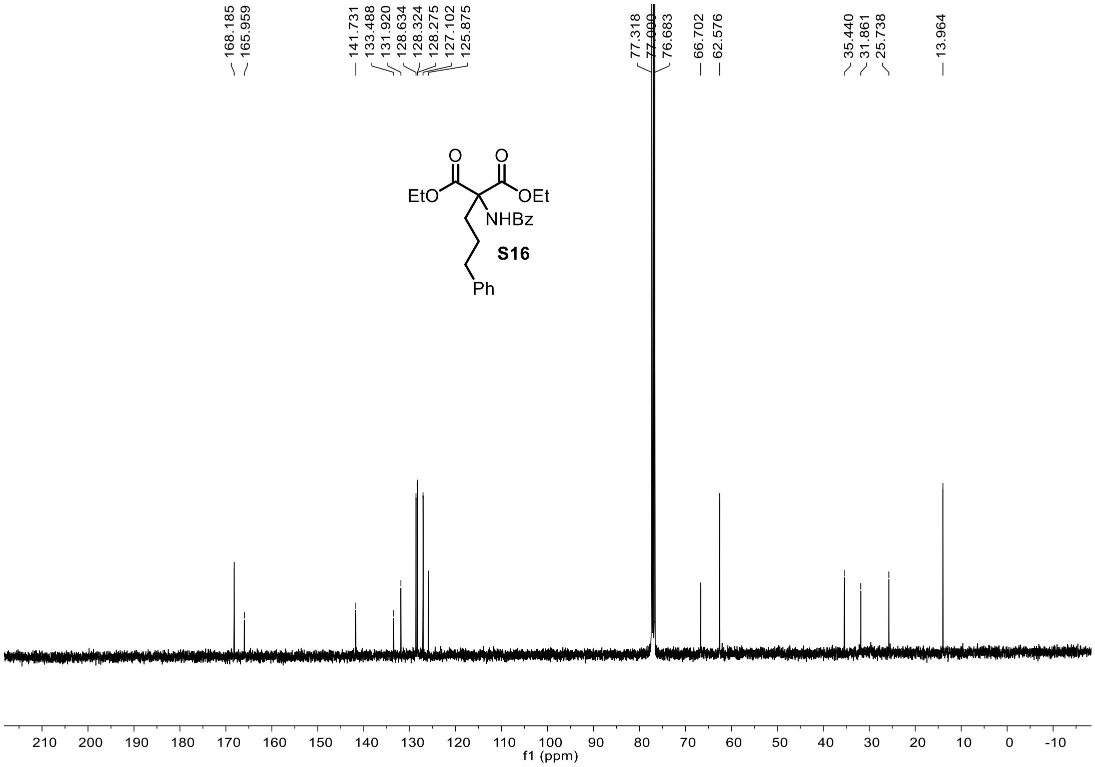

Supplementary Fig. 23. ^{1}H NMR of compound S17 ($CDCl_3$, 400 MHz, 25 °C)

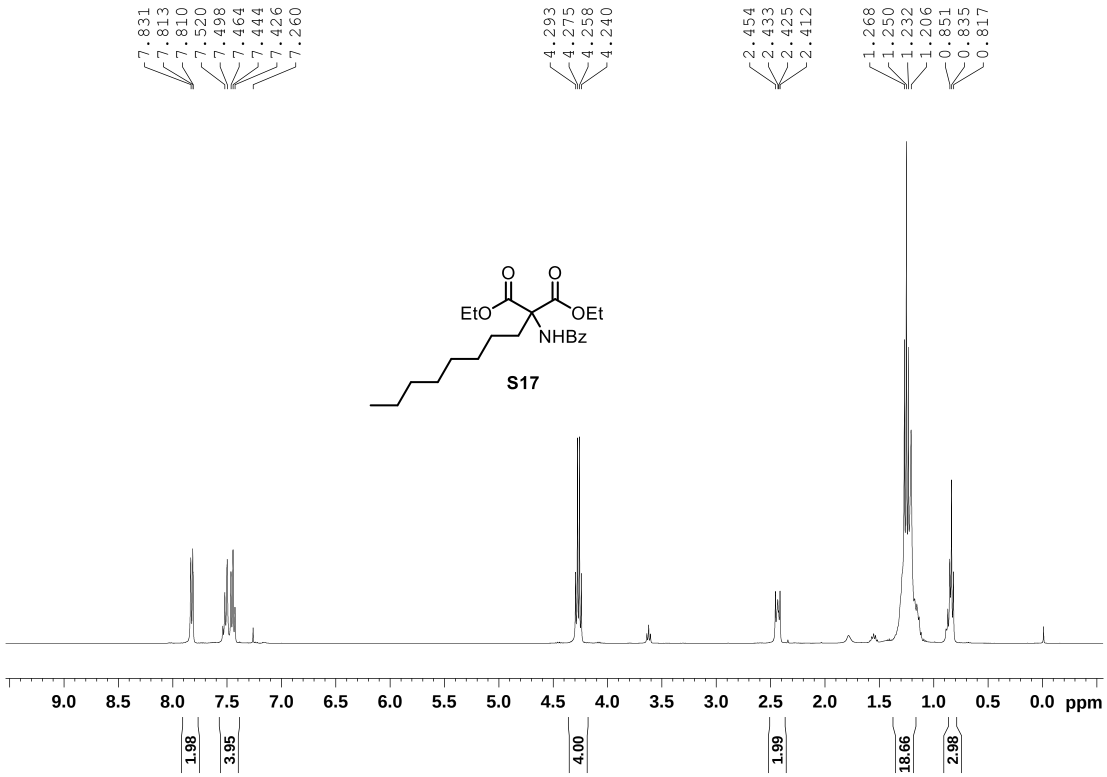

Supplementary Fig. 24. ^{13}C NMR of compound S17 ($CDCl_3$, 100 MHz, 25 °C)

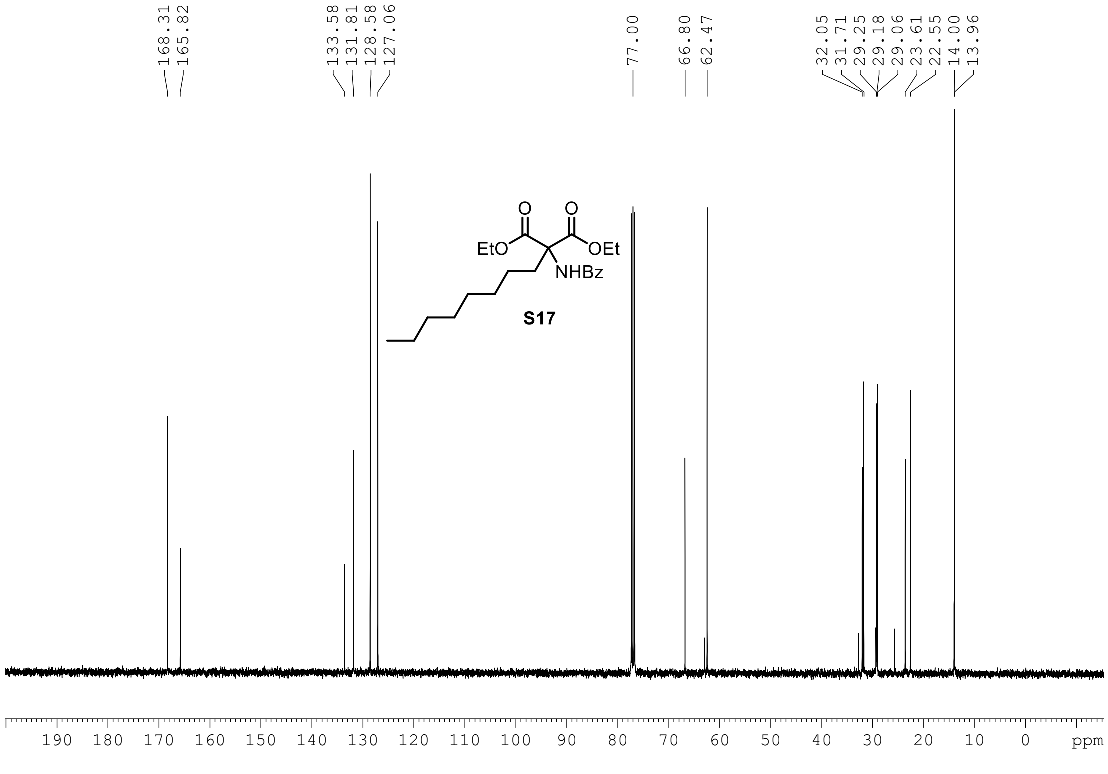

Supplementary Fig. 25. ^1H NMR of compound S18 ($CDCl_3$, 400 MHz, 25 °C)

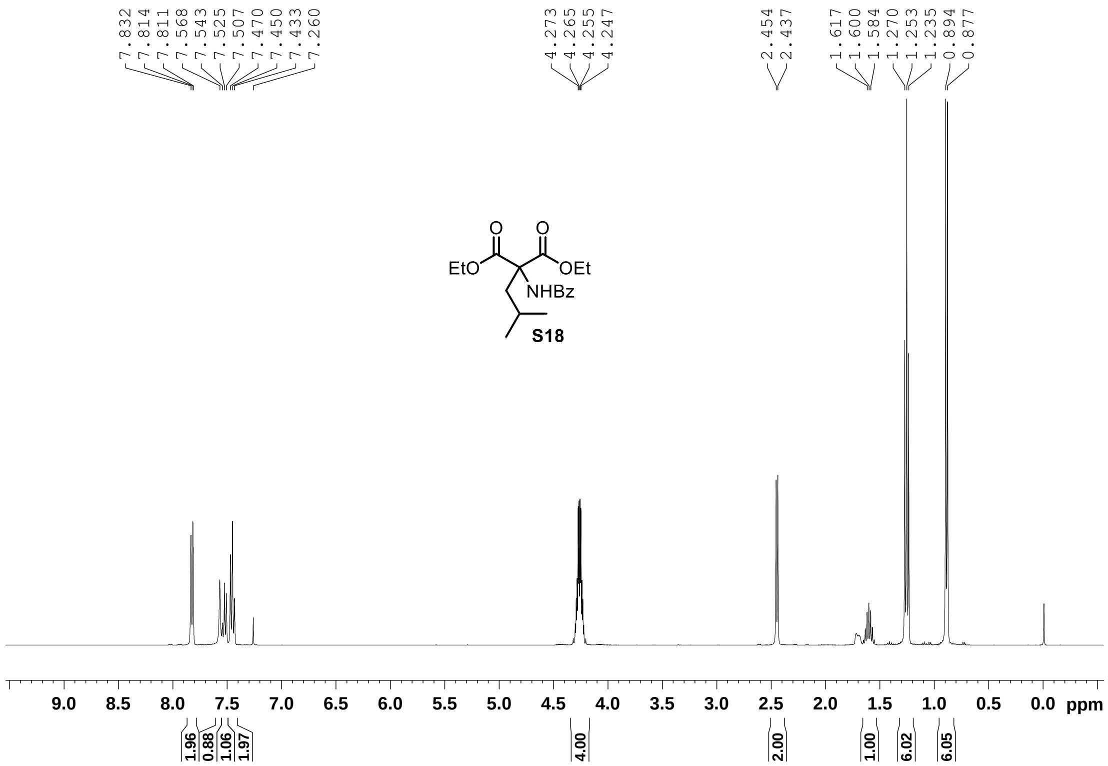

Supplementary Fig. 26. ^{13}C NMR of compound S18 ($CDCl_3$, 100 MHz, 25 °C)

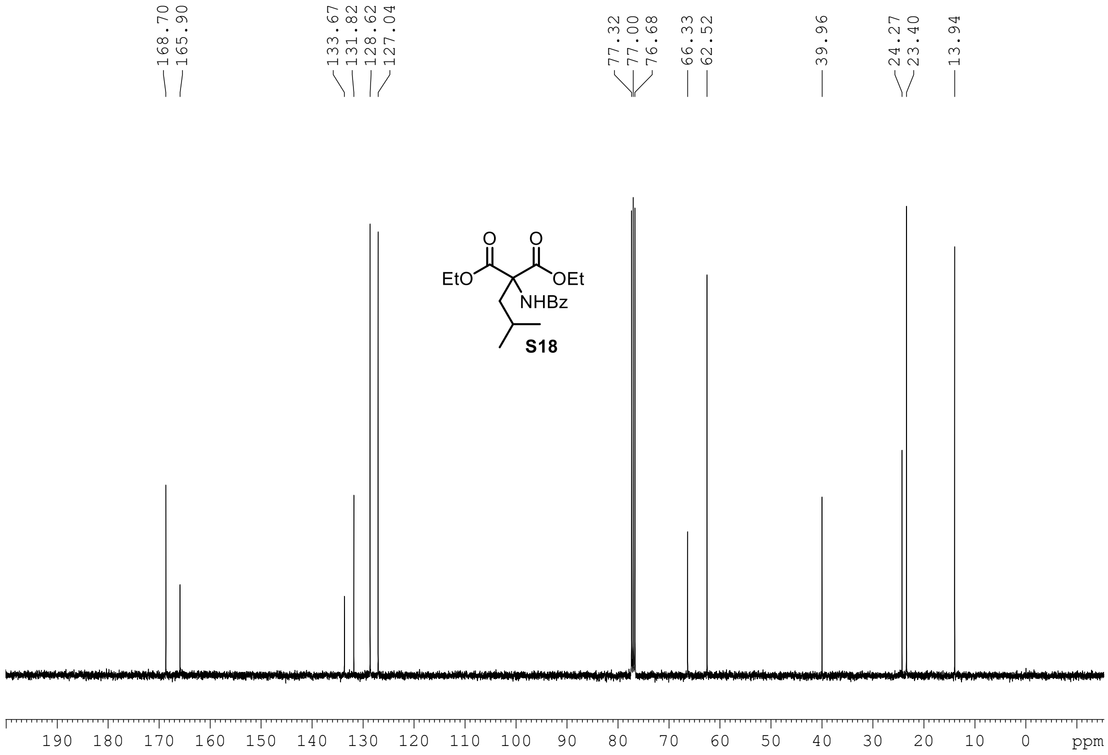

Supplementary Fig. 27. ^{1}**H NMR of compound S19 (CDCl**$_3$**, 400 MHz, 25 °C)**

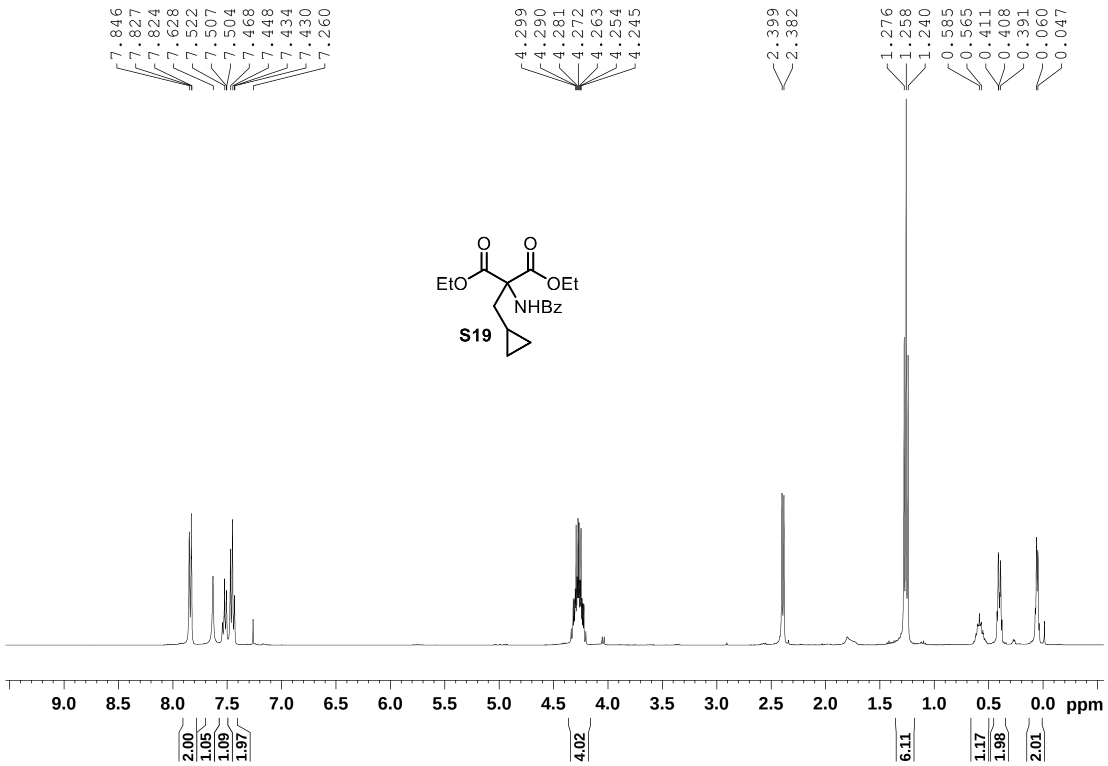

Supplementary Fig. 28. ^{13}**C NMR of compound S19 (CDCl**$_3$**, 100 MHz, 25 °C)**

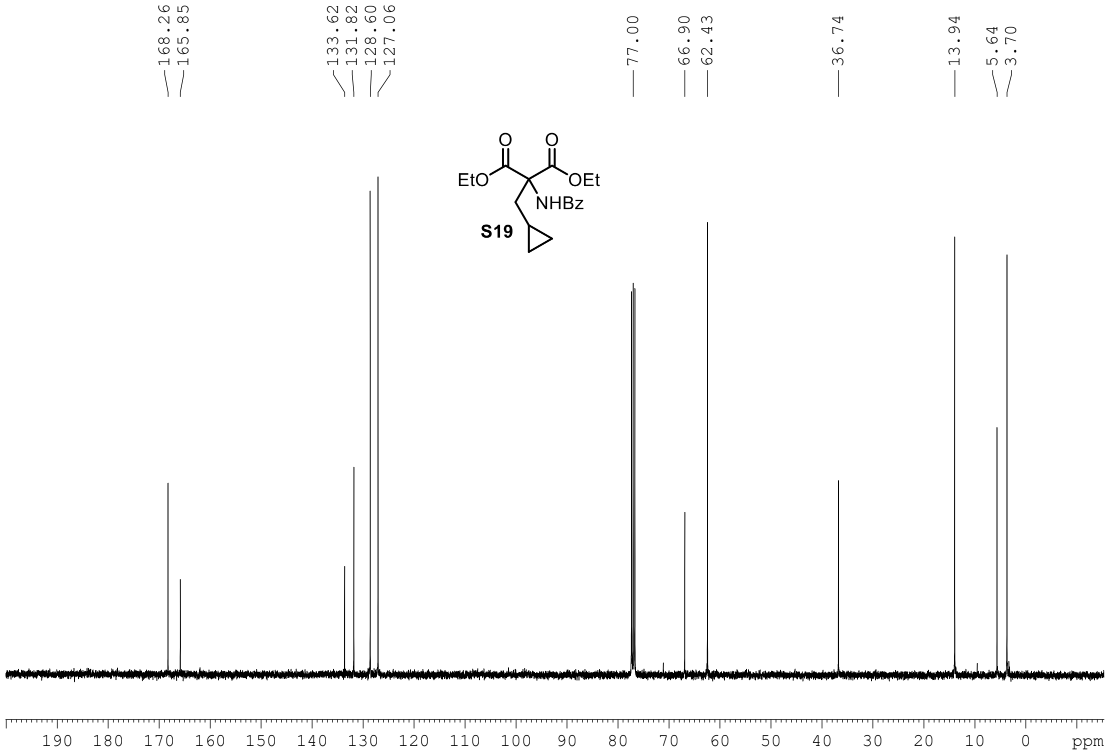

Supplementary Fig. 29. ^{1}H NMR of compound S20 ($CDCl_3$, 400 MHz, 25 °C)

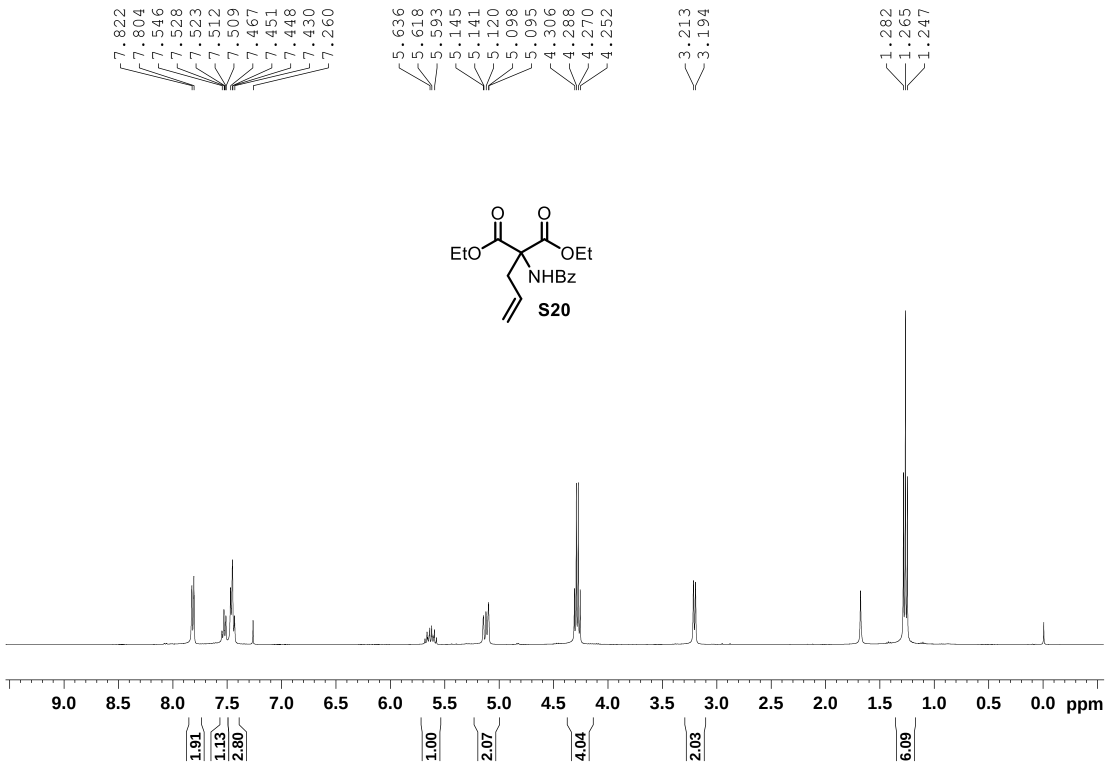

Supplementary Fig. 30. ^{13}C NMR of compound S20 ($CDCl_3$, 100 MHz, 25 °C)

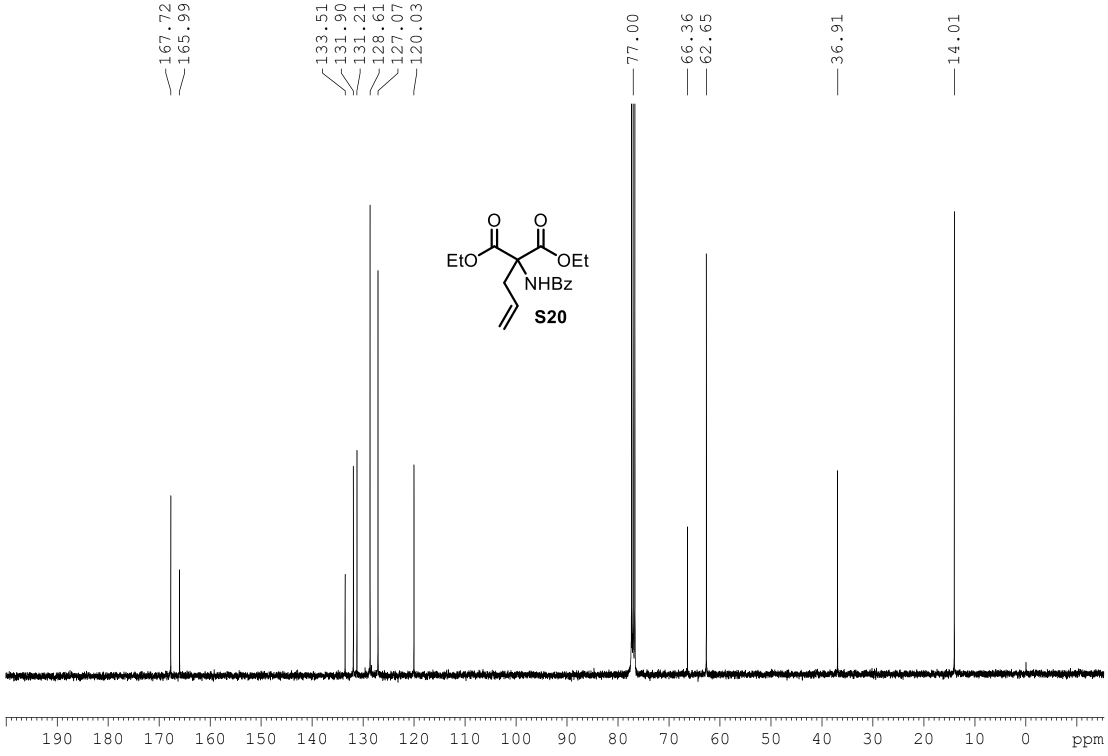

Supplementary Fig. 31. ^{1}H NMR of compound S21 ($CDCl_3$, 400 MHz, 25 °C)

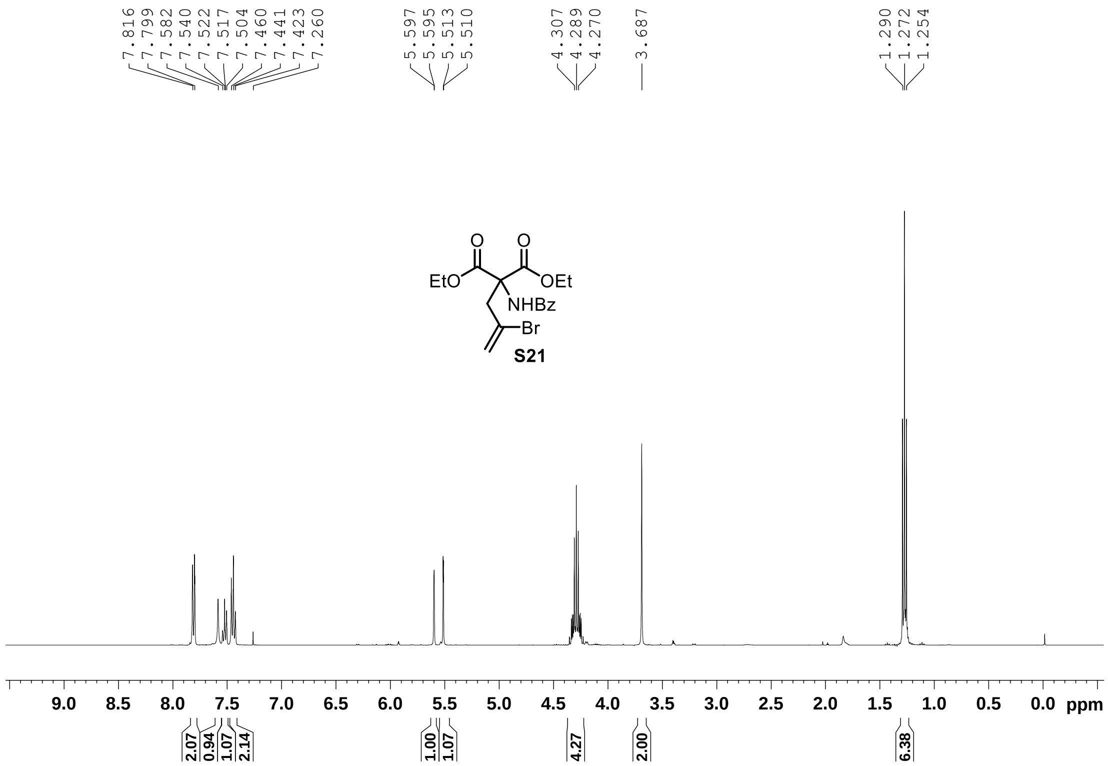

Supplementary Fig. 32. ^{13}C NMR of compound S21 ($CDCl_3$, 100 MHz, 25 °C)

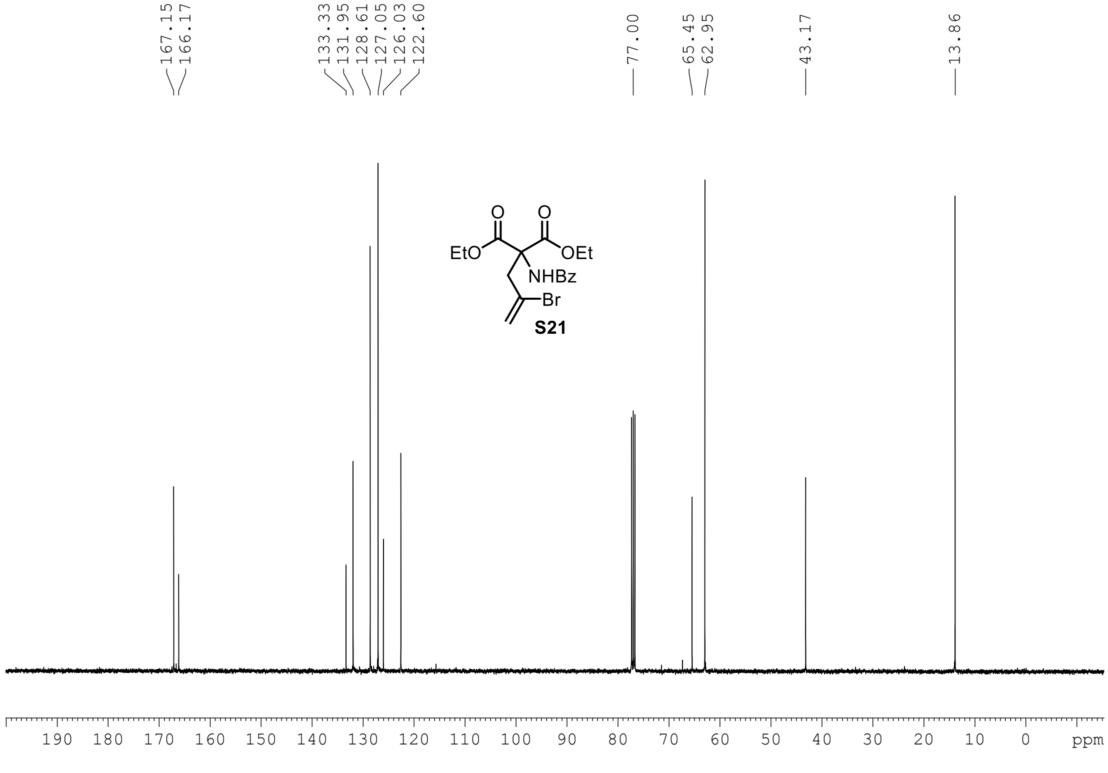

Supplementary Fig. 33. ^{1}H NMR of compound S22 ($CDCl_3$, 400 MHz, 25 °C)

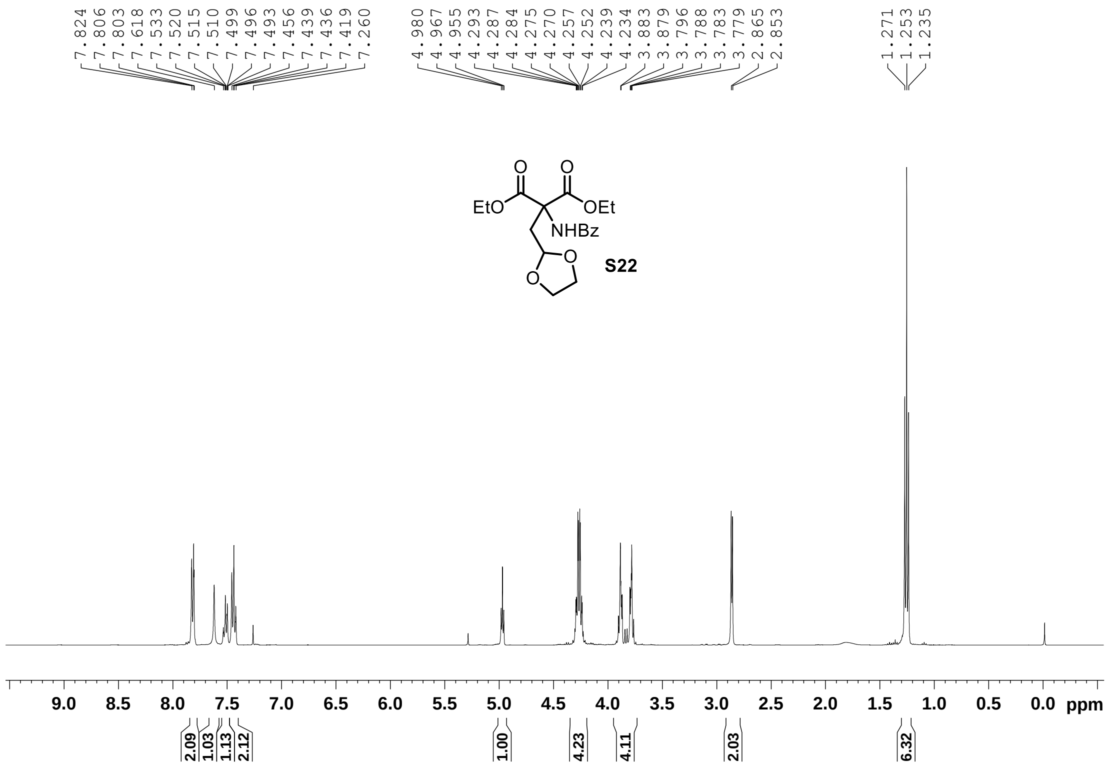

Supplementary Fig. 34. ^{13}C NMR of compound S22 ($CDCl_3$, 100 MHz, 25 °C)

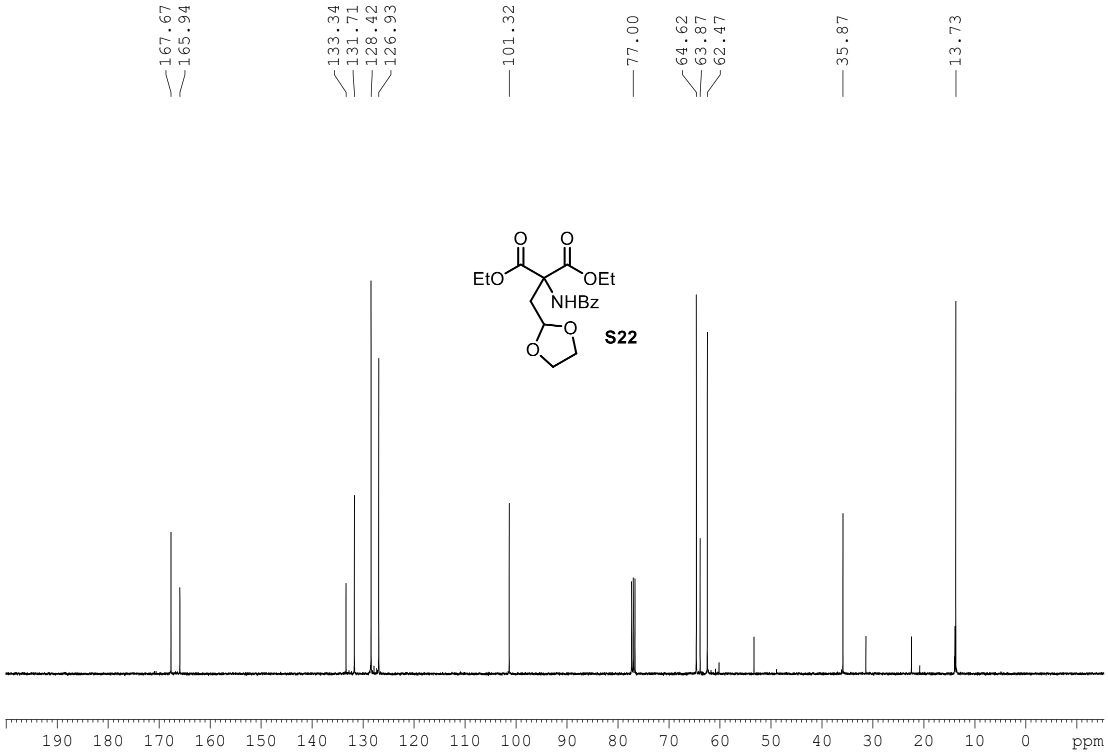

Supplementary Fig. 35. ^{1}H NMR of compound S23 ($CDCl_3$, 400 MHz, 25 °C)

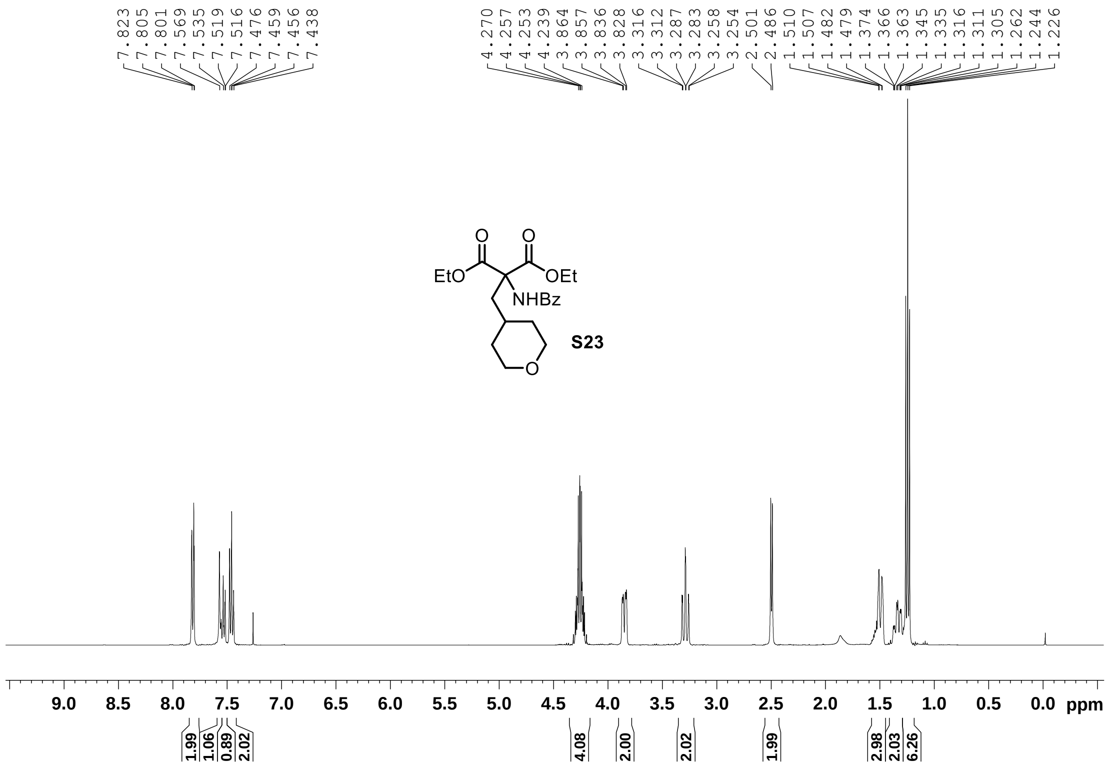

Supplementary Fig. 36. ^{13}C NMR of compound S23 ($CDCl_3$, 100 MHz, 25 °C)

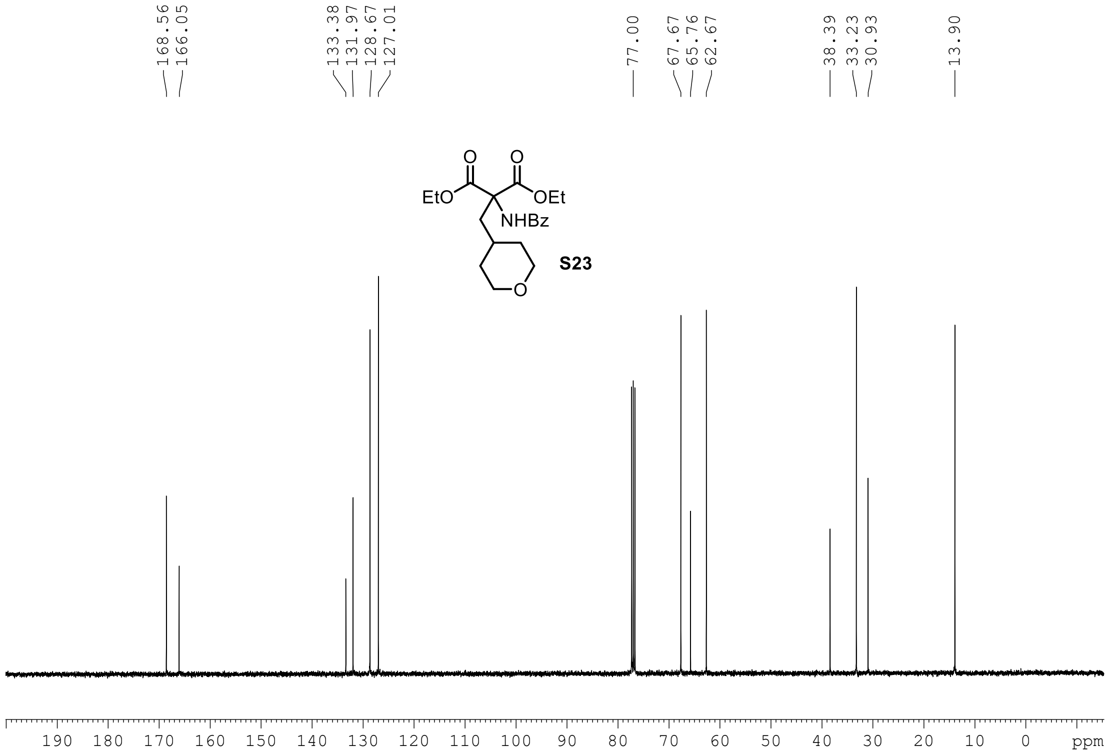

Supplementary Fig. 37. ^{1}H NMR of compound S24 ($CDCl_3$, 400 MHz, 25 °C)

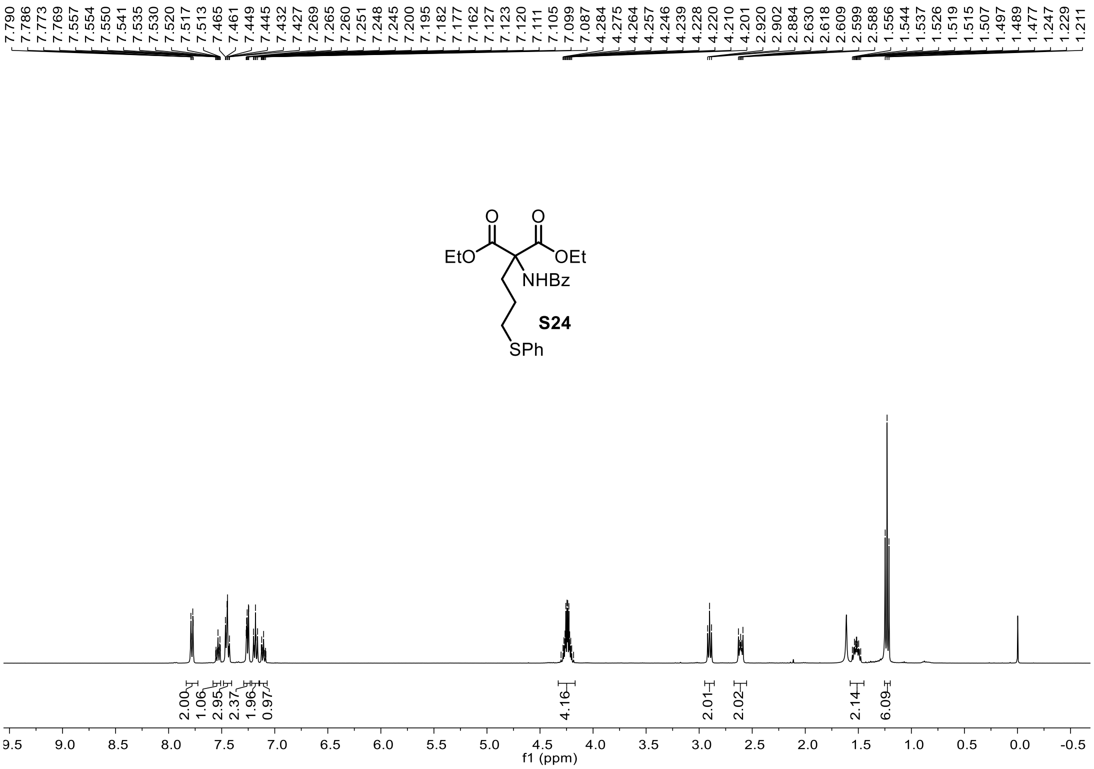

Supplementary Fig. 38. ^{13}C NMR of compound S24 ($CDCl_3$, 100 MHz, 25 °C)

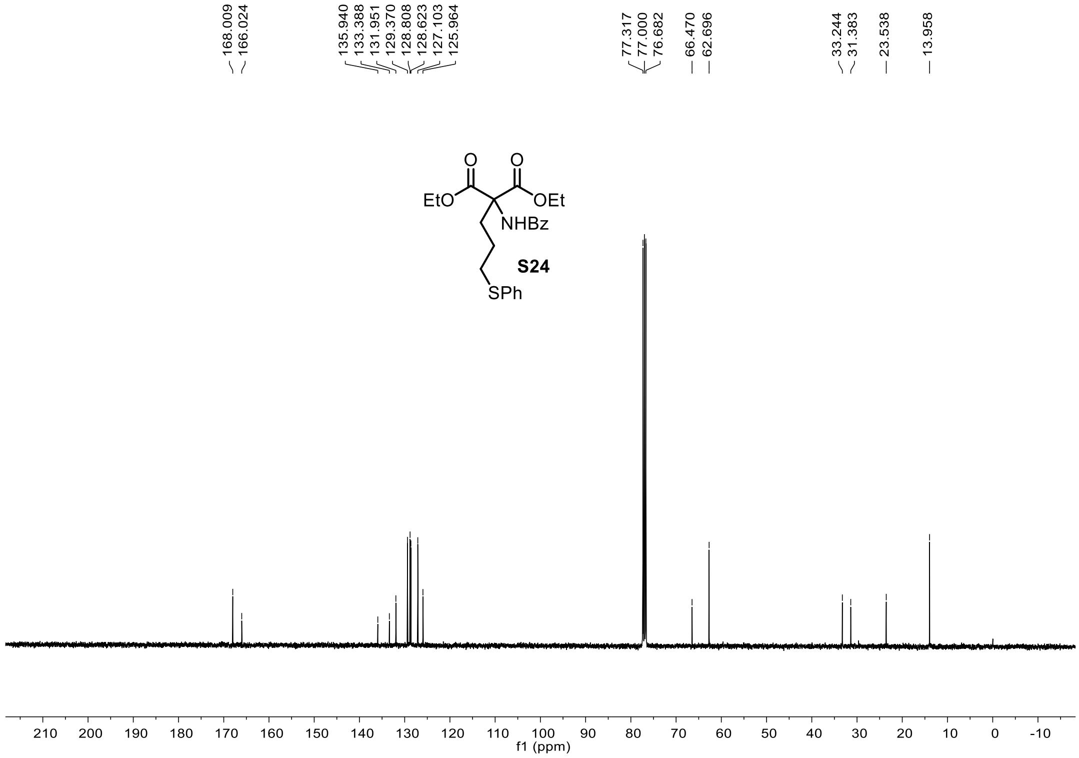

Supplementary Fig. 39. ^{1}H **NMR of compound S25 ($CDCl_3$, 400 MHz, 25 °C)**

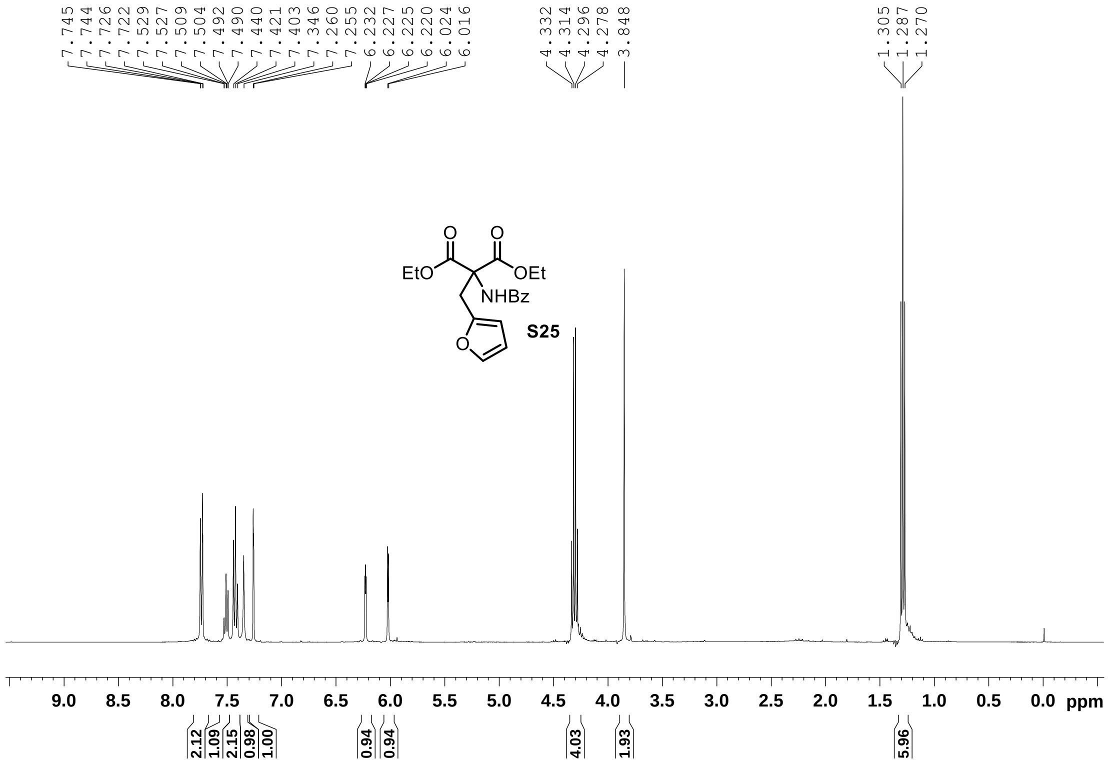

Supplementary Fig. 40. ^{13}C **NMR of compound S25 ($CDCl_3$, 100 MHz, 25 °C)**

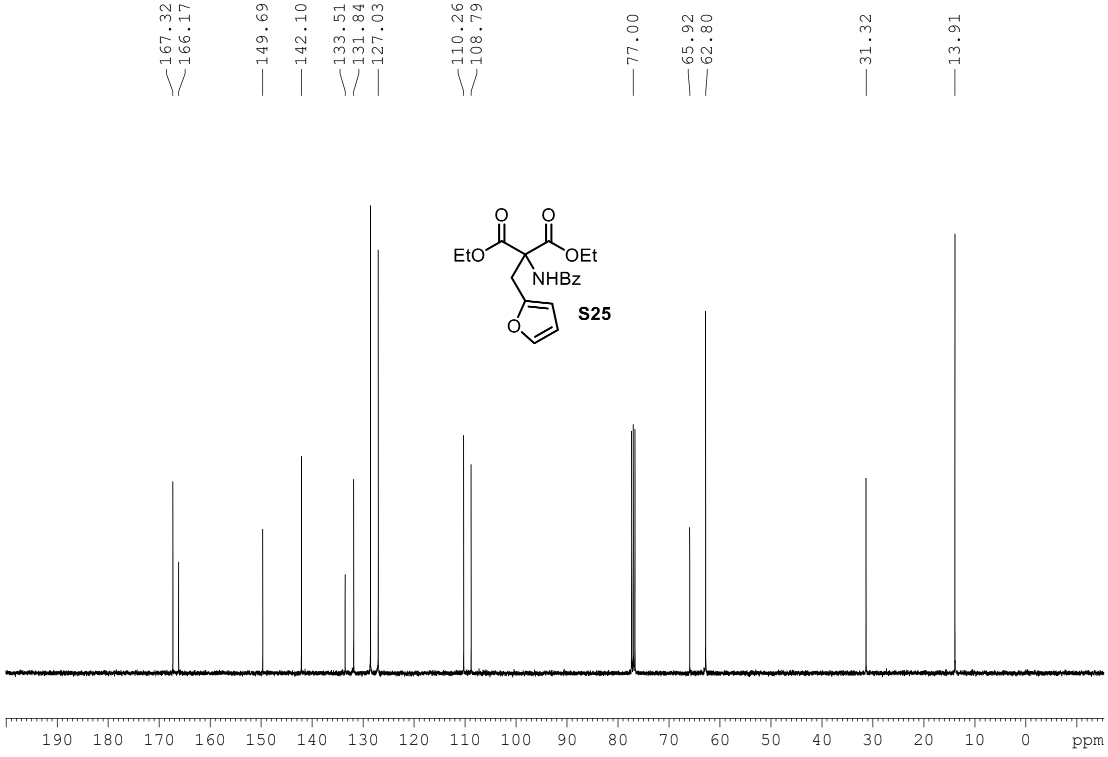

Supplementary Fig. 41. ^{1}H NMR of compound S26 ($CDCl_3$, 400 MHz, 25 °C)

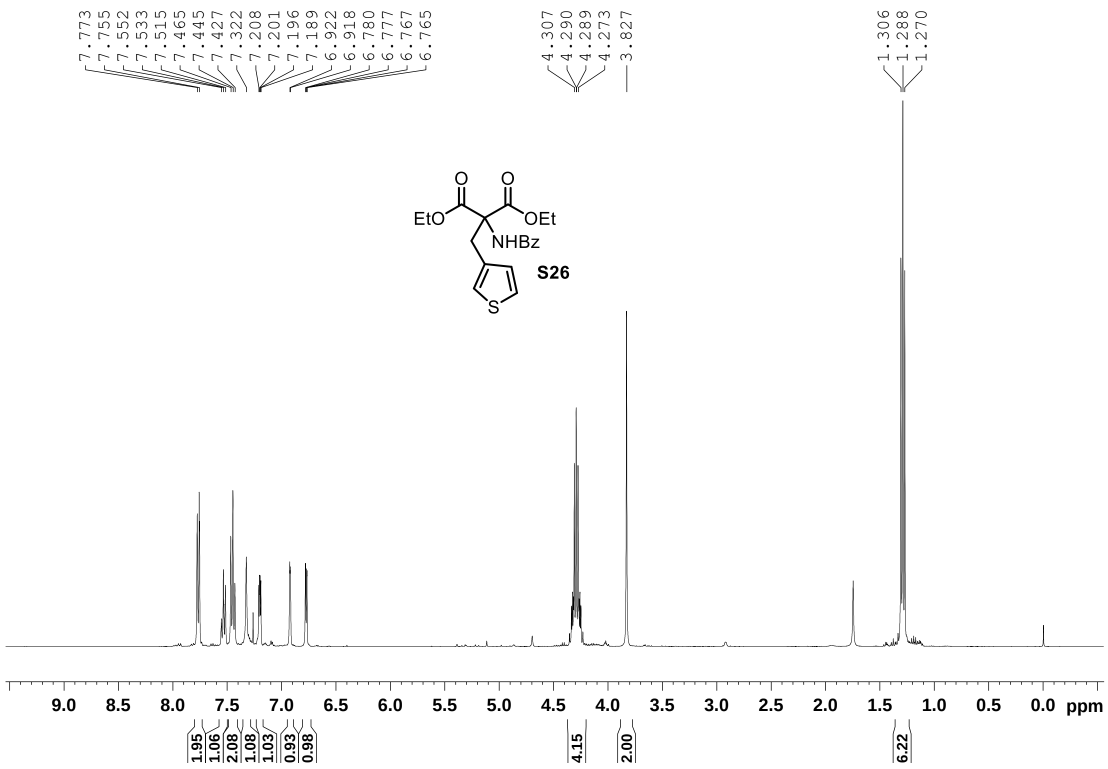

Supplementary Fig. 42. ^{13}C NMR of compound S26 ($CDCl_3$, 100 MHz, 25 °C)

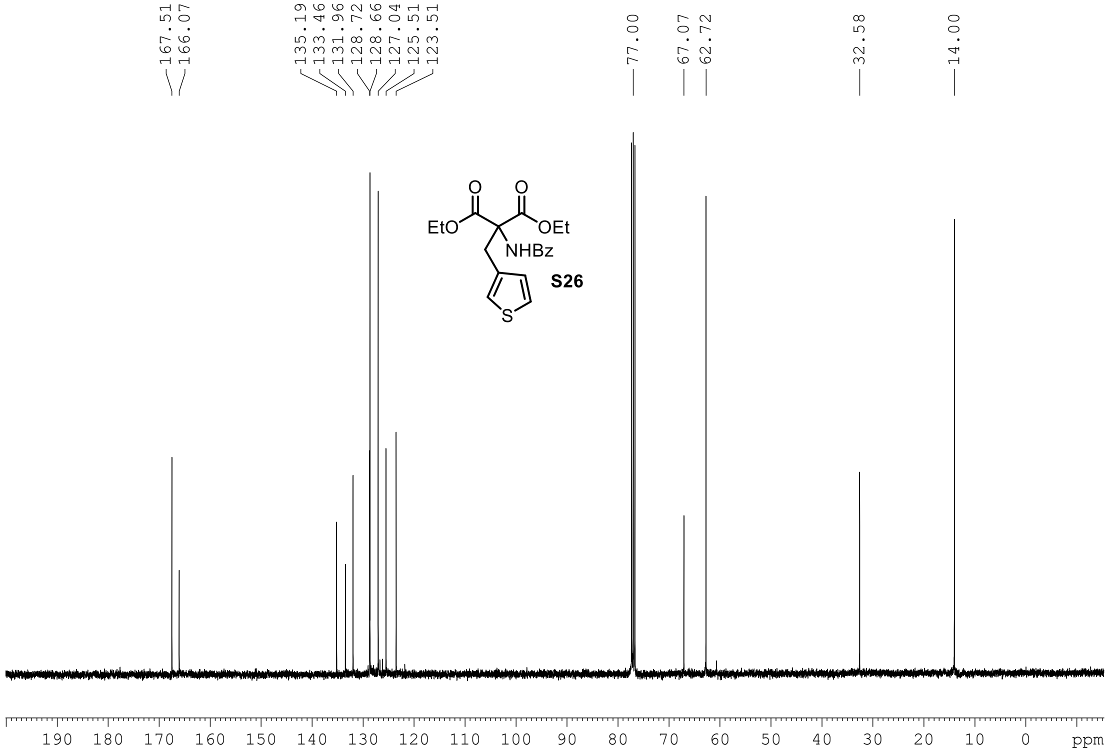

Supplementary Fig. 43. **^{1}H NMR of compound S27 ($CDCl_3$, 400 MHz, 25 °C)**

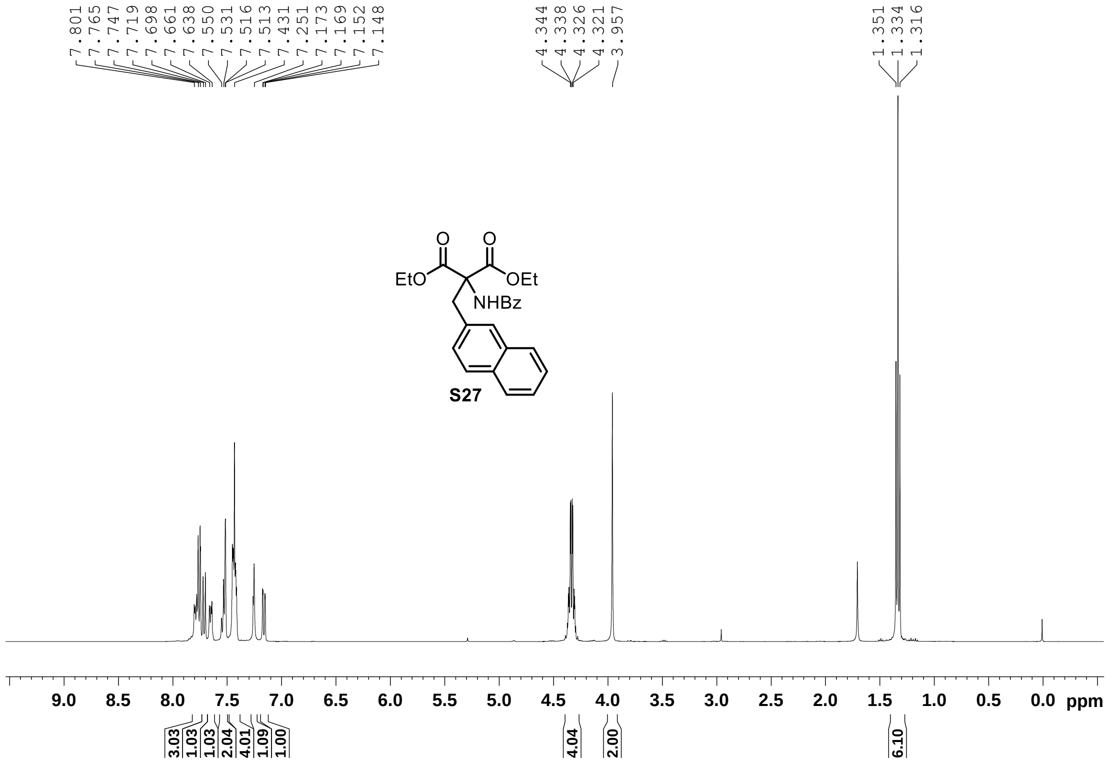

Supplementary Fig. 44. **^{13}C NMR of compound S27 ($CDCl_3$, 100 MHz, 25 °C)**

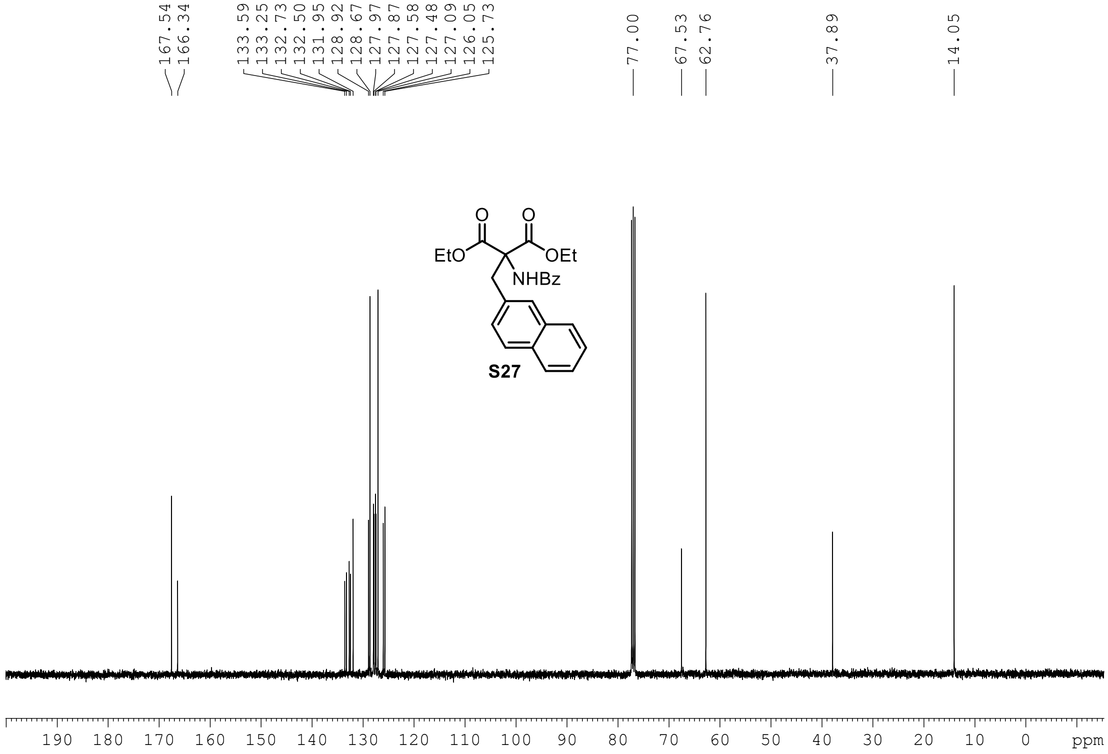

Supplementary Fig. 45. ¹H NMR of compound S28 ($CDCl_3$, 400 MHz, 25 °C)

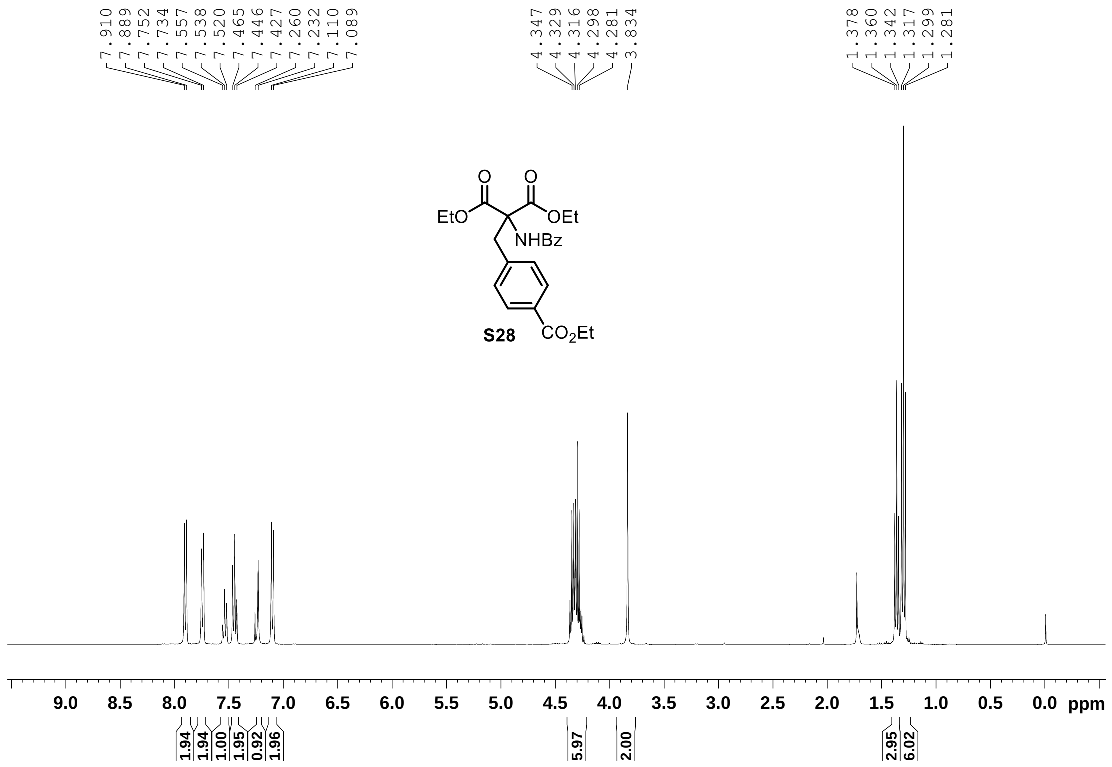

Supplementary Fig. 46. ¹³C NMR of compound S28 ($CDCl_3$, 100 MHz, 25 °C)

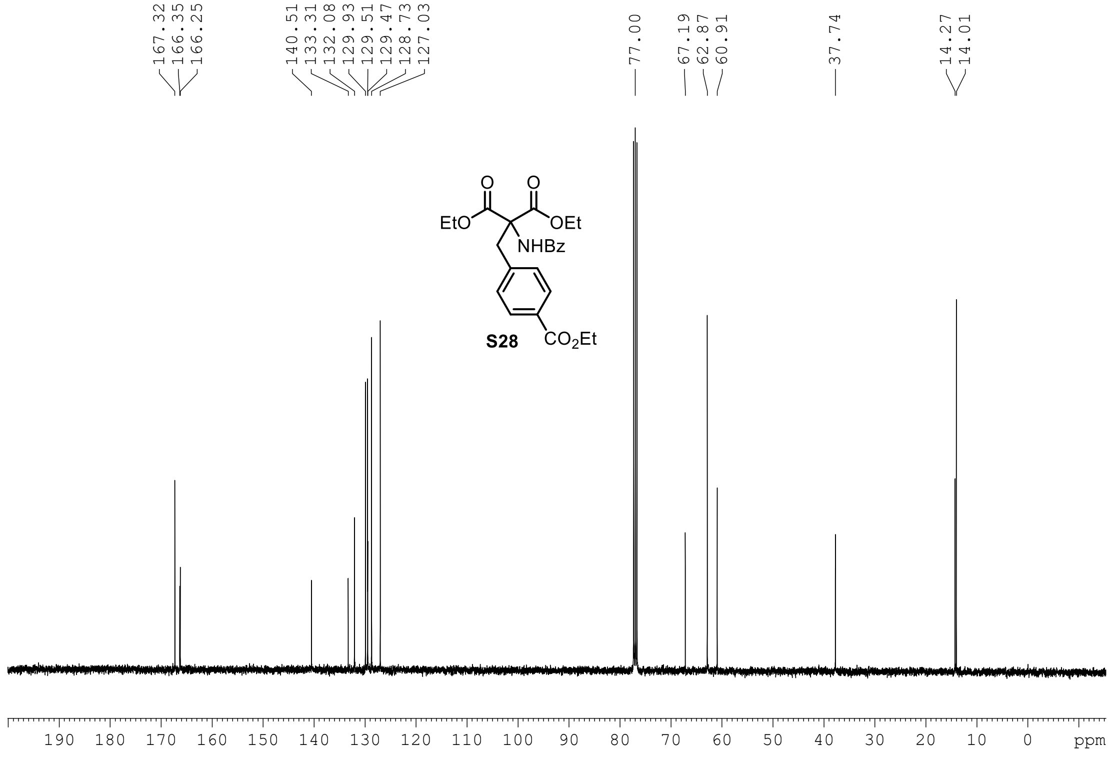

Supplementary Fig. 47. ^{1}H NMR of compound S29 ($CDCl_3$, 400 MHz, 25 °C)

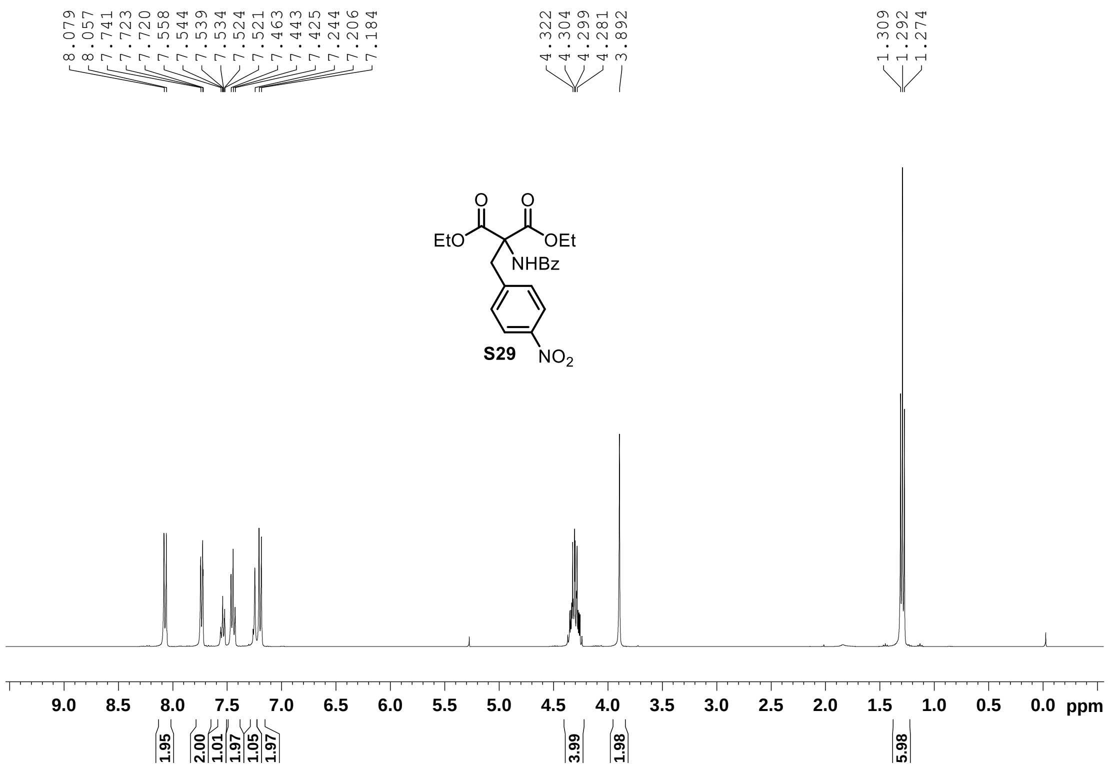

Supplementary Fig. 48. ^{13}C NMR of compound S29 ($CDCl_3$, 100 MHz, 25 °C)

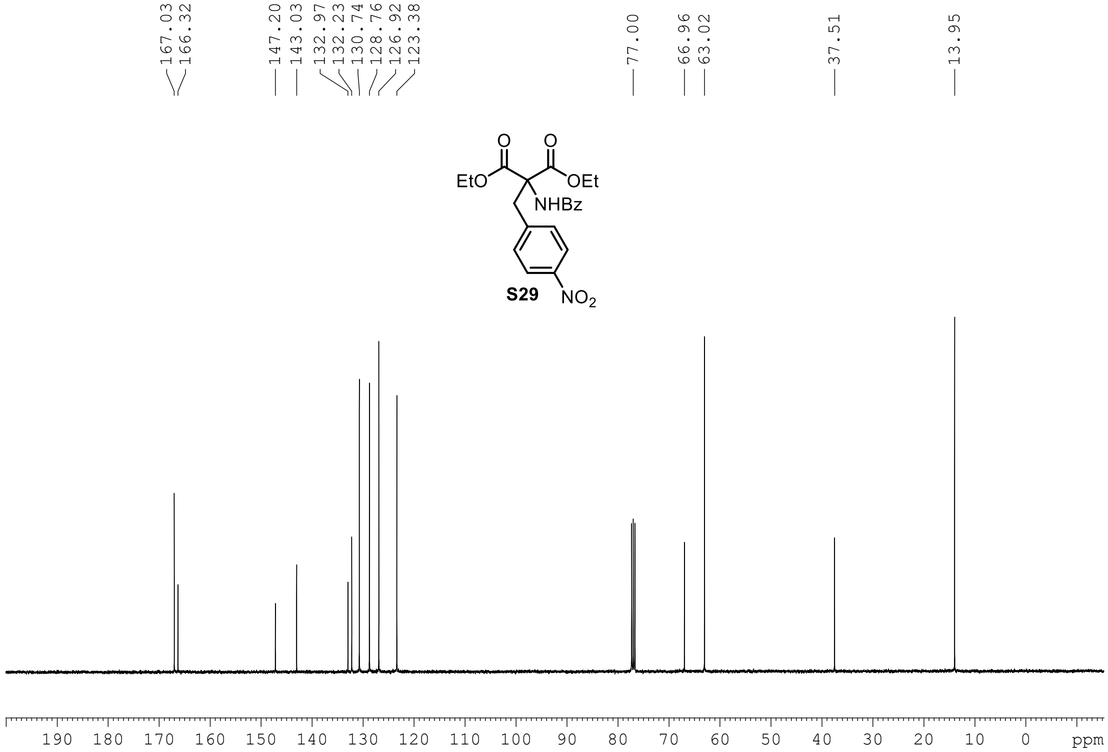

Supplementary Fig. 49. ^{1}H NMR of compound S30 ($CDCl_3$, 400 MHz, 25 °C)

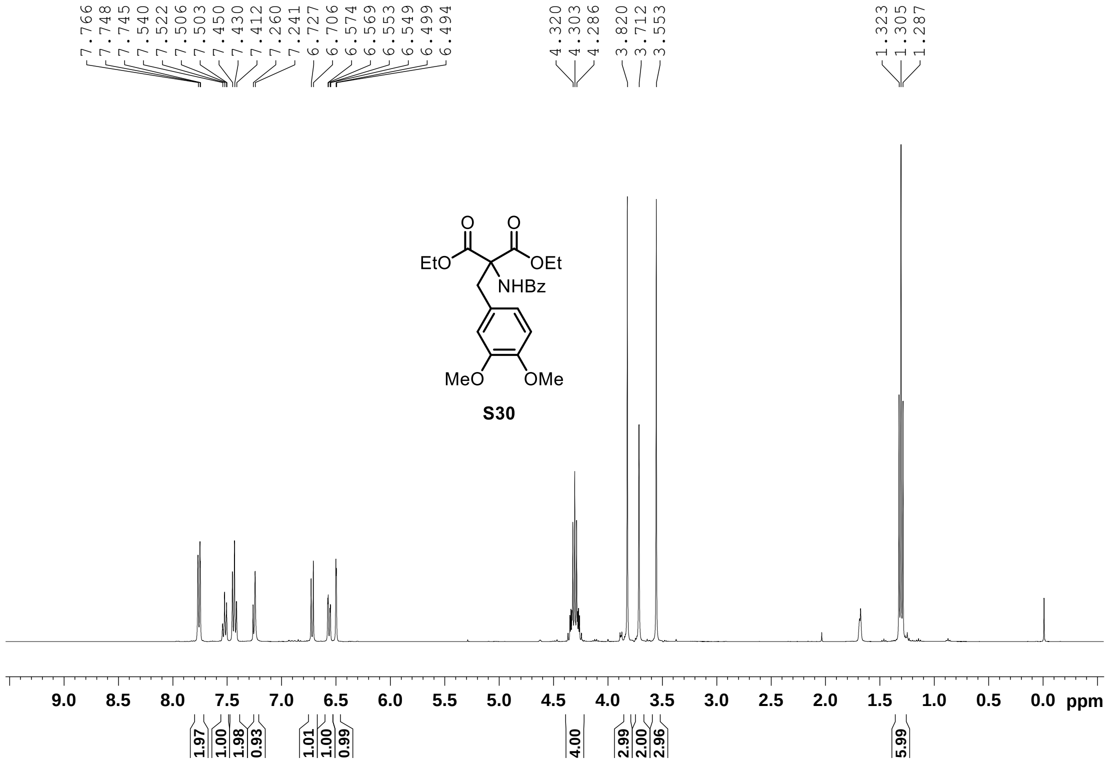

Supplementary Fig. 50. ^{13}C NMR of compound S30 ($CDCl_3$, 100 MHz, 25 °C)

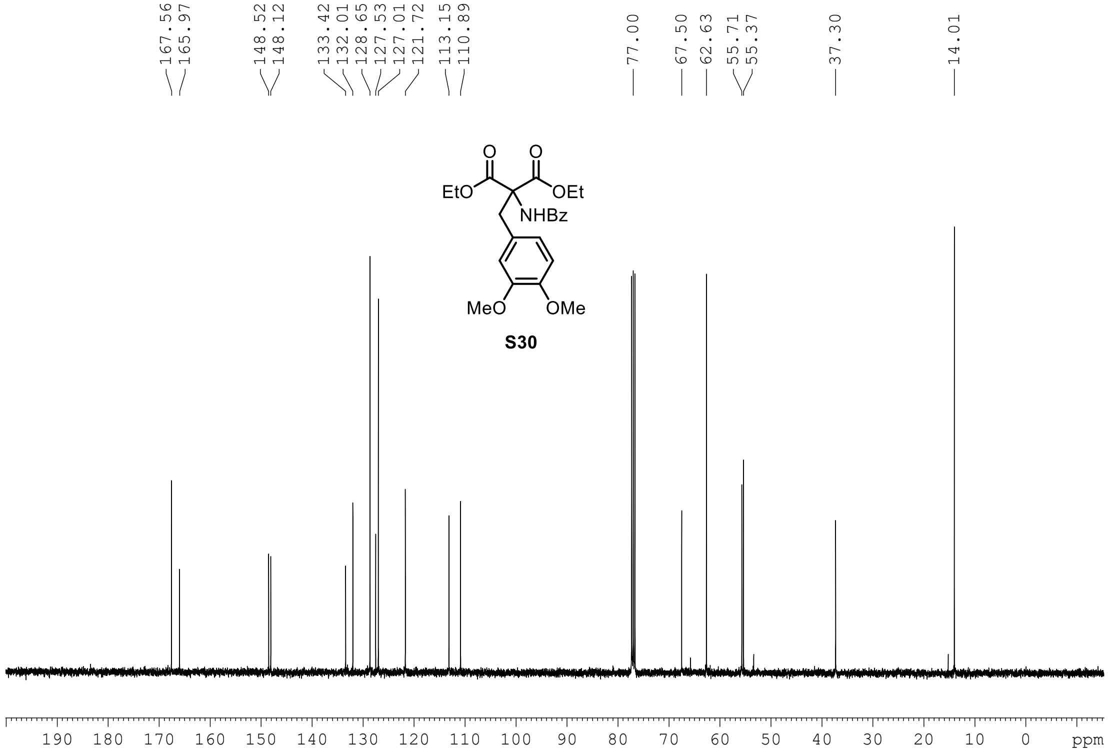

Supplementary Fig. 51. ^{1}H **NMR of compound S31 ($CDCl_3$, 400 MHz, 25 °C)**

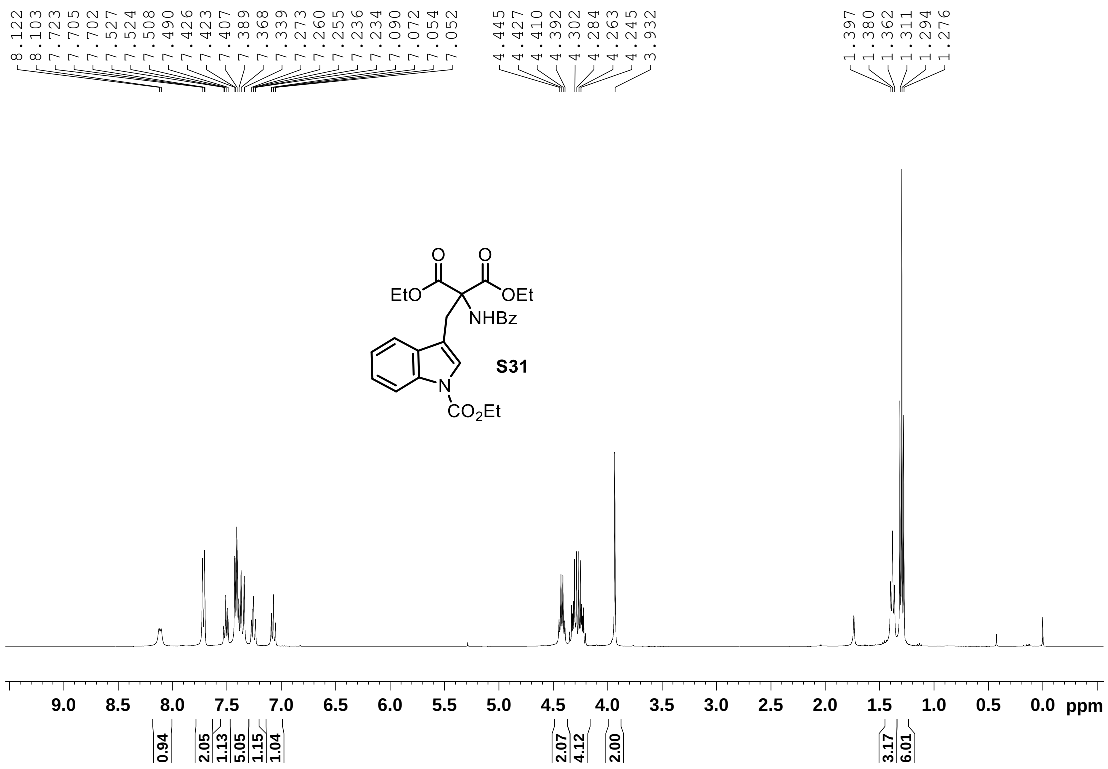

Supplementary Fig. 52. ^{13}C **NMR of compound S31 ($CDCl_3$, 100 MHz, 25 °C)**

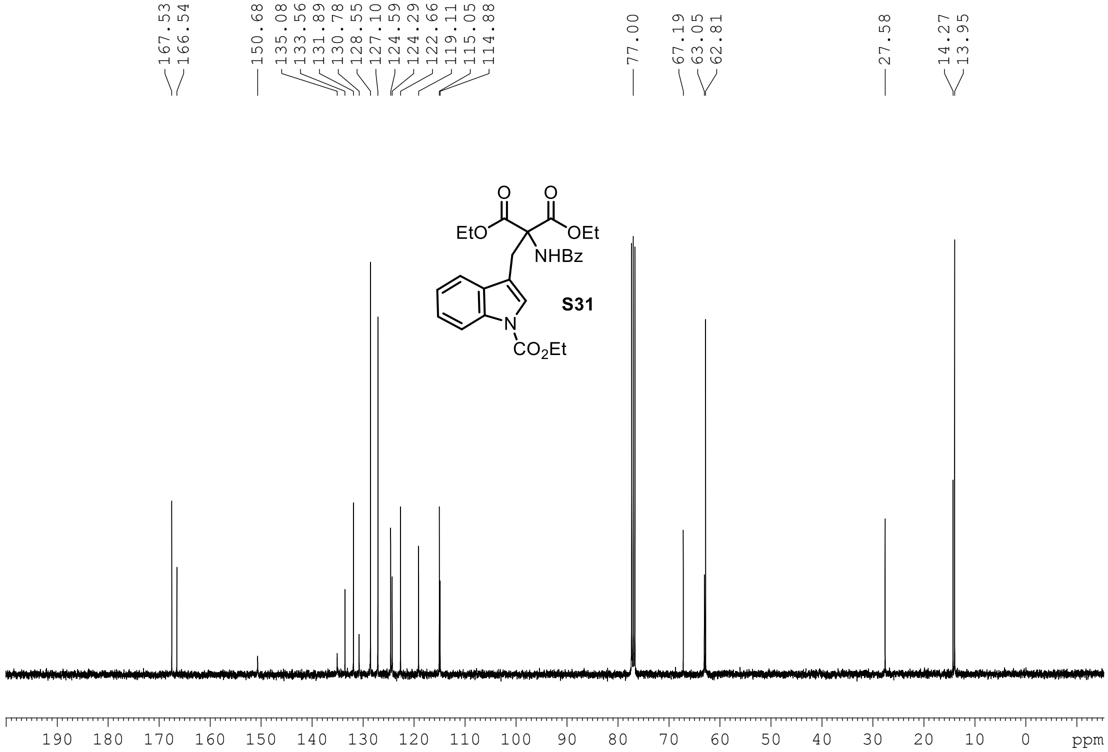

Supplementary Fig. 53. ^{1}H NMR of compound S32 ($CDCl_3$, 400 MHz, 25 °C)

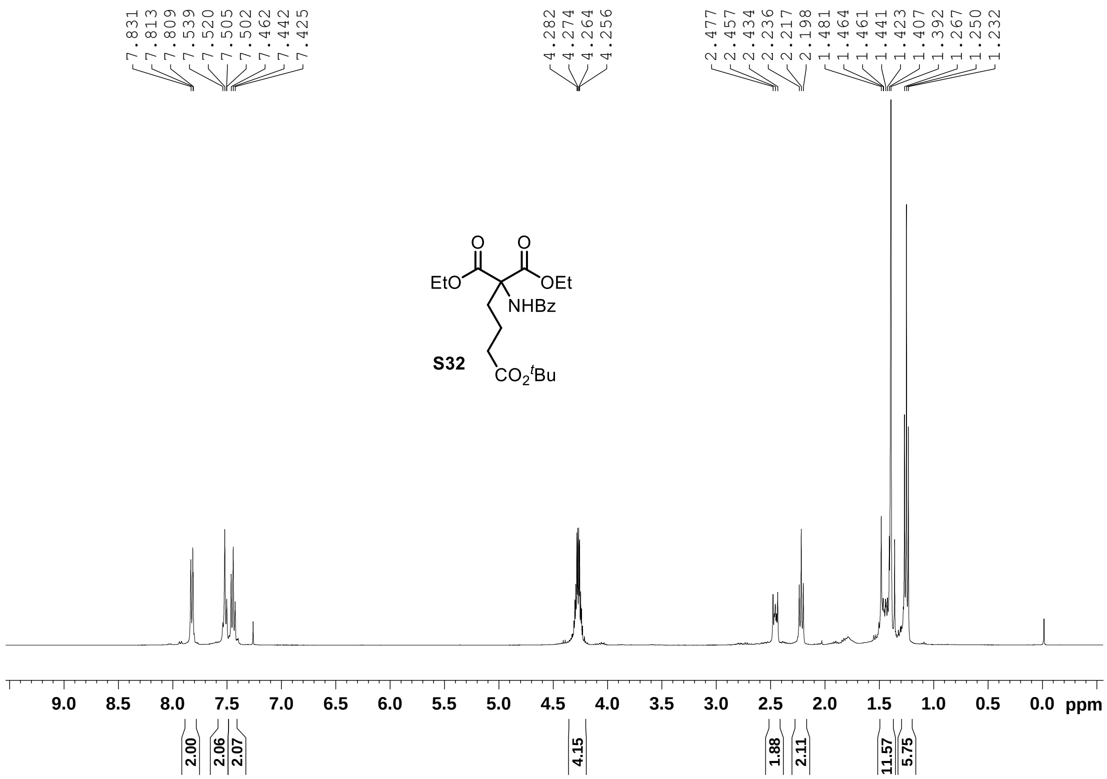

Supplementary Fig. 54. ^{13}C NMR of compound S32 ($CDCl_3$, 100 MHz, 25 °C)

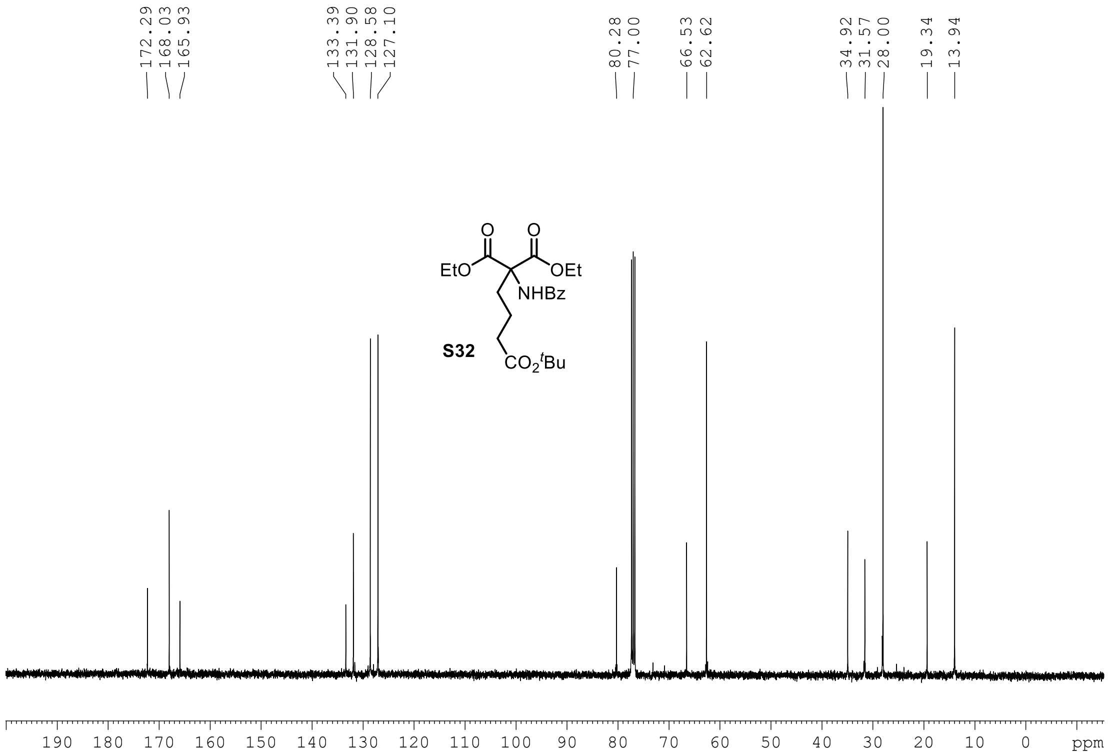

Supplementary Fig. 55. ^{1}H NMR of compound S33 ($CDCl_3$, 400 MHz, 25 °C)

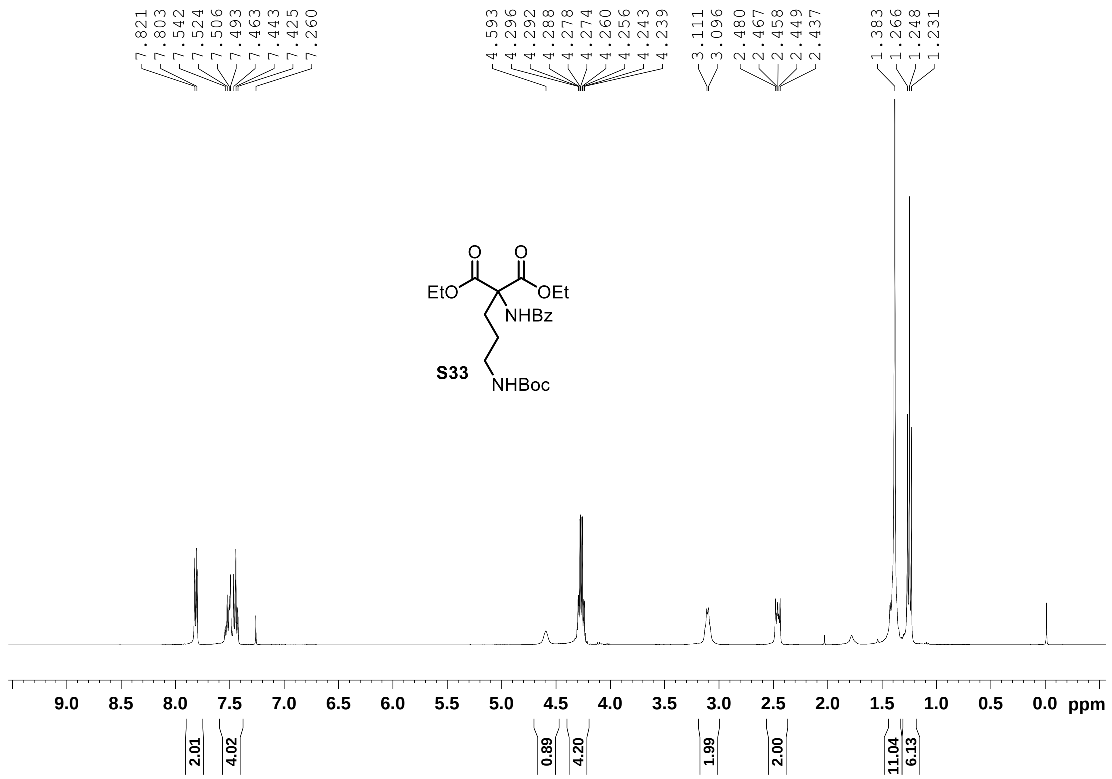

Supplementary Fig. 56. ^{13}C NMR of compound S33 ($CDCl_3$, 100 MHz, 25 °C)

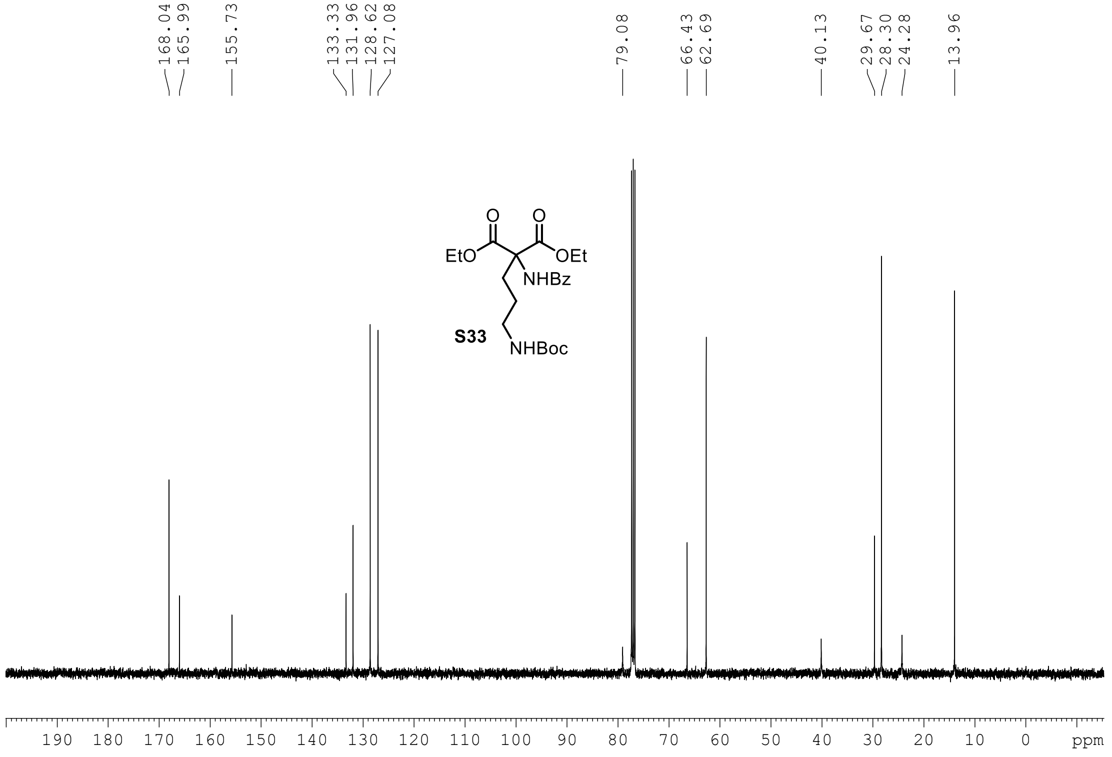

Supplementary Fig. 57. ^{1}H NMR of compound S34 ($CDCl_3$, 400 MHz, 25 °C)

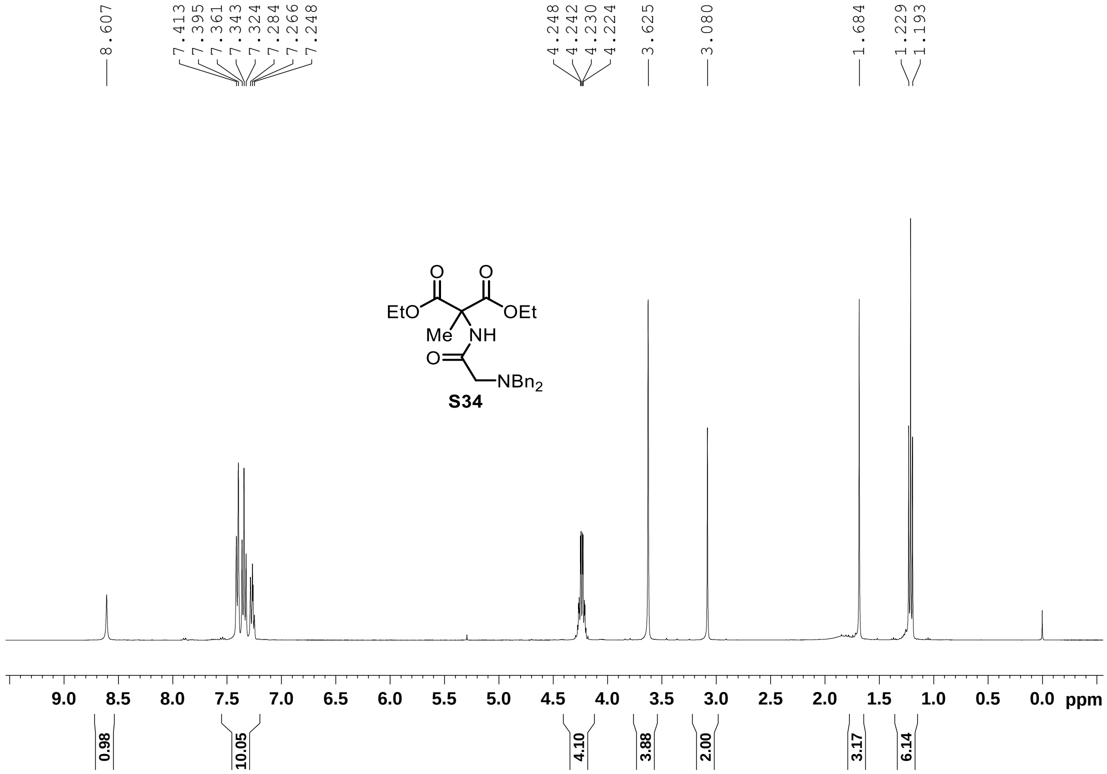

Supplementary Fig. 58. ^{13}C NMR of compound S34 ($CDCl_3$, 100 MHz, 25 °C)

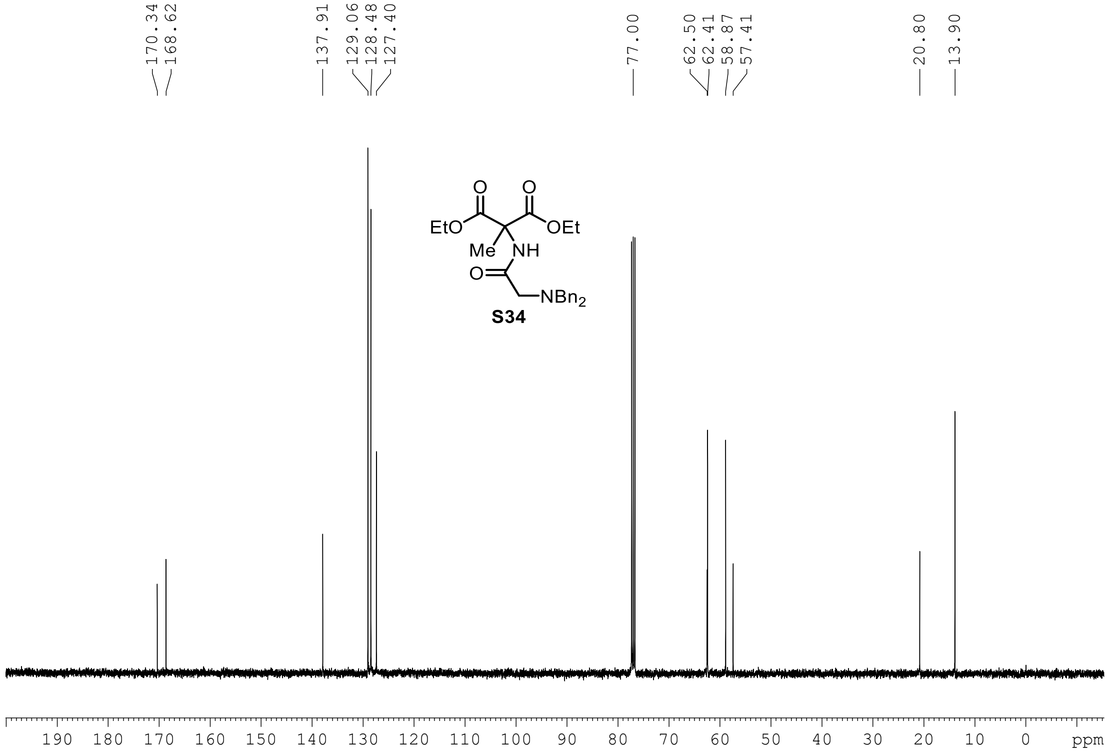

Supplementary Fig. 59. ^{1}H NMR of compound S35 ($CDCl_3$, 400 MHz, 25 °C)

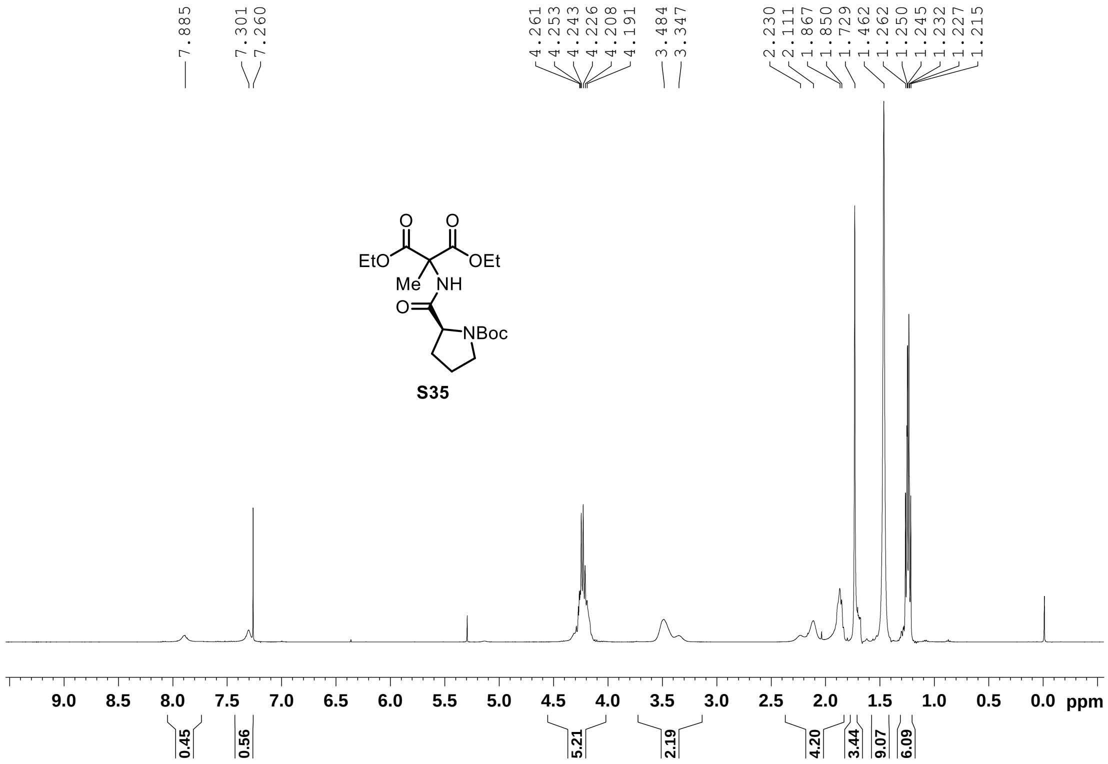

Supplementary Fig. 60. ^{13}C NMR of compound S35 ($CDCl_3$, 100 MHz, 25 °C)

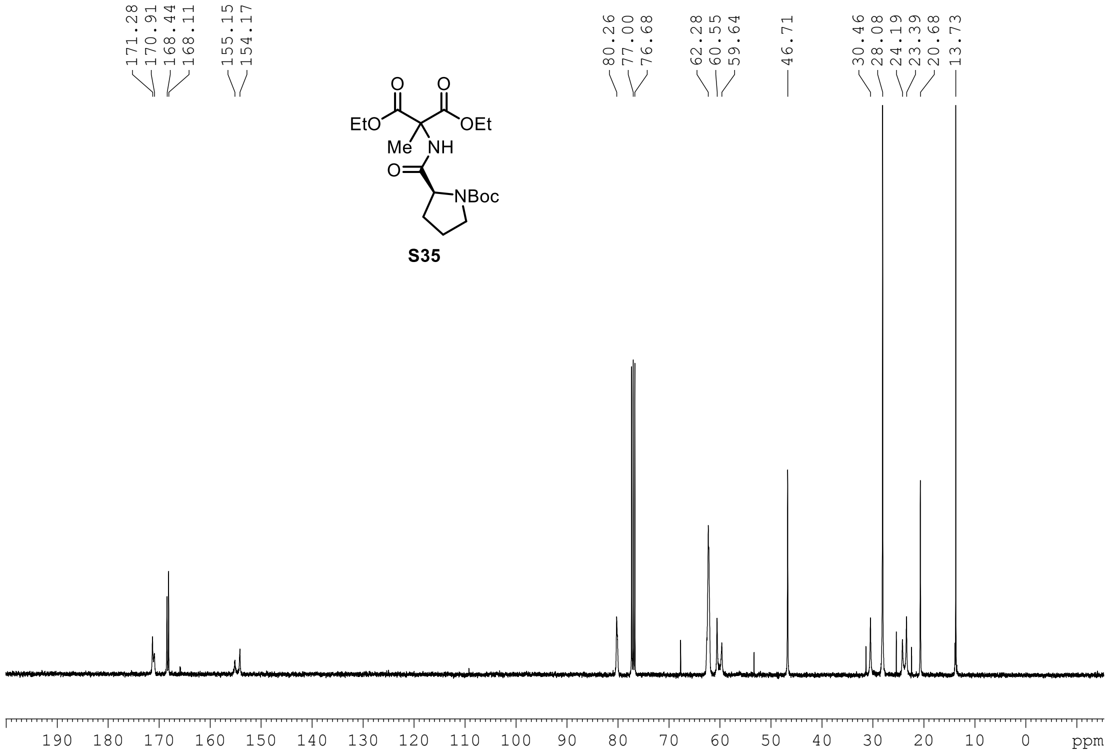

Supplementary Fig. 61. ^{1}H NMR of compound S37 ($CDCl_3$, 400 MHz, 25 °C)

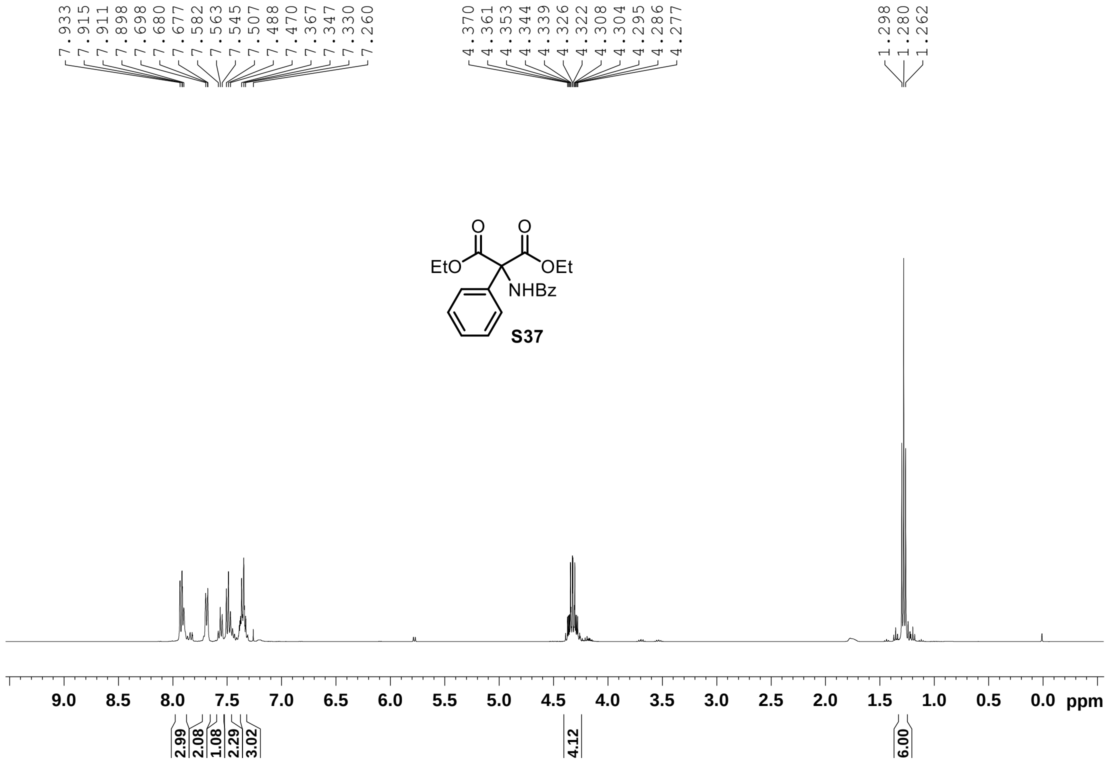

Supplementary Fig. 62. ^{13}C NMR of compound S37 ($CDCl_3$, 100 MHz, 25 °C)

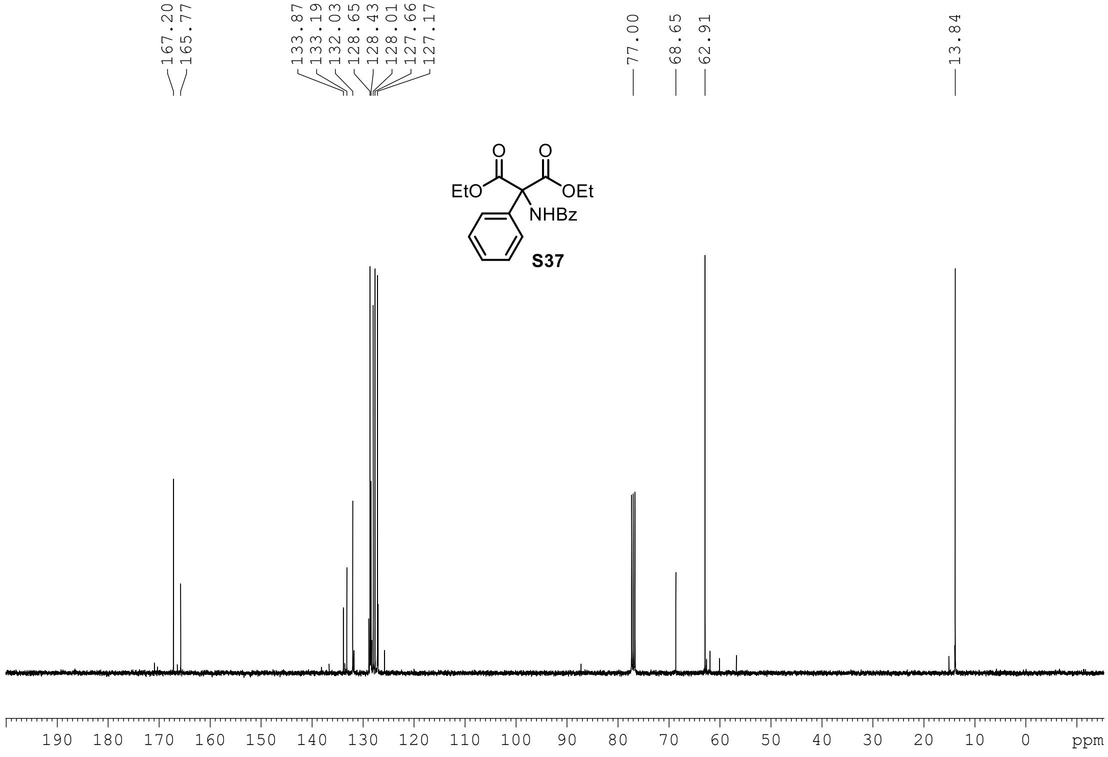

Supplementary Fig. 63. ^{1}H NMR of compound S38 ($CDCl_3$, 400 MHz, 25 °C)

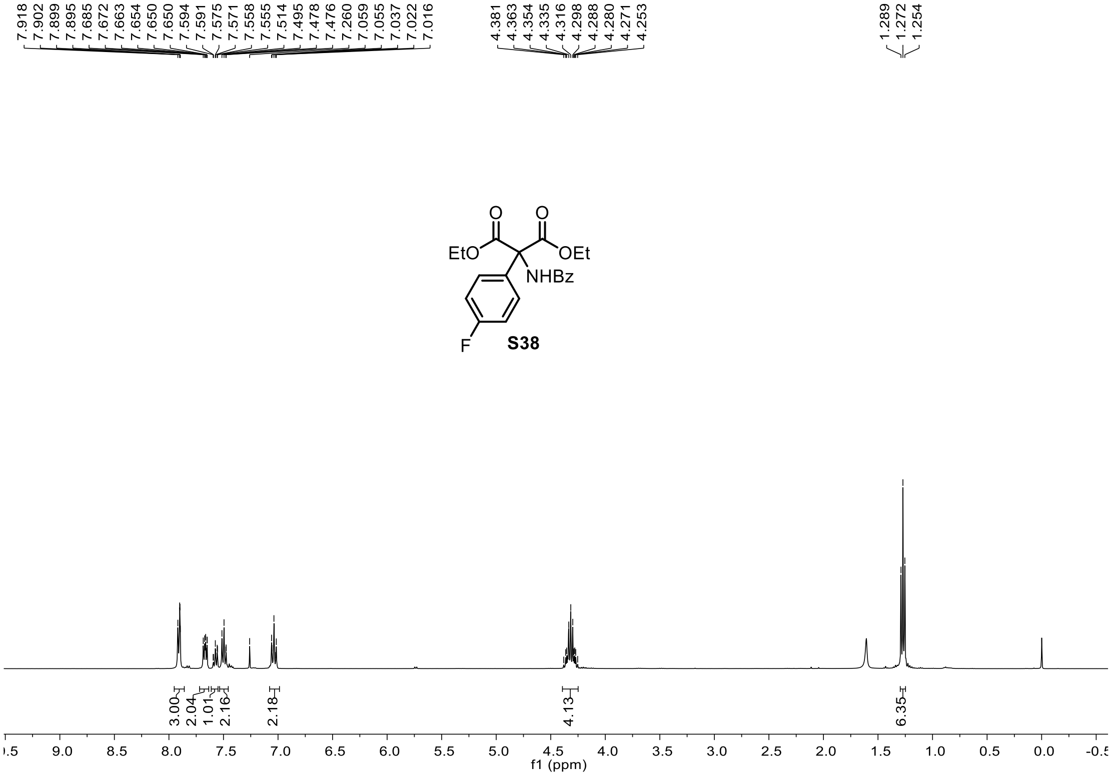

Supplementary Fig. 64. ^{13}C NMR of compound S38 ($CDCl_3$, 100 MHz, 25 °C)

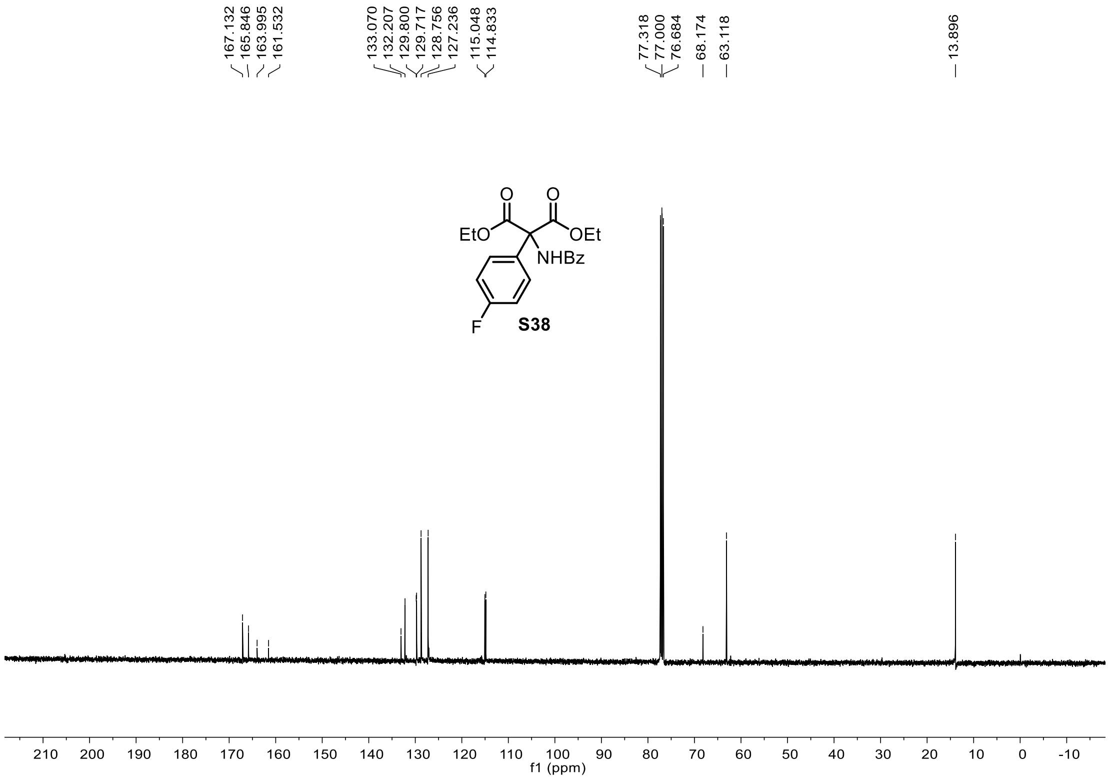

Supplementary Fig. 65. ^{1}H NMR of compound S39 ($CDCl_3$, 400 MHz, 25 °C)

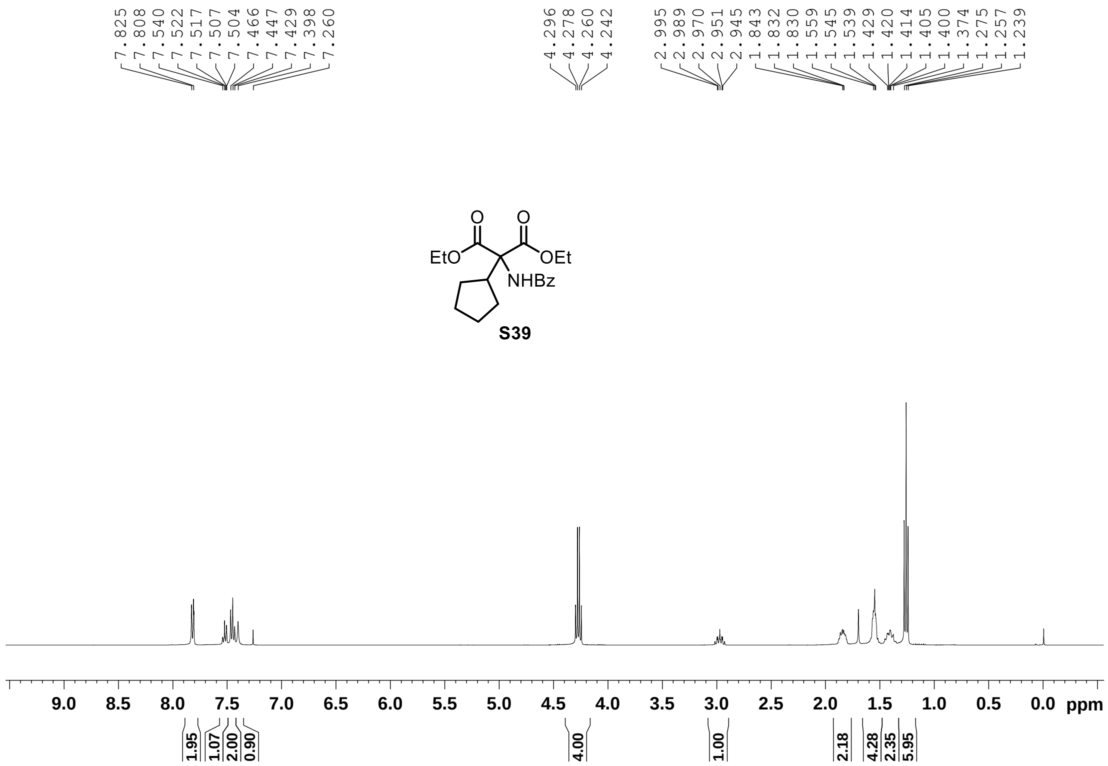

Supplementary Fig. 66. ^{13}C NMR of compound S39 ($CDCl_3$, 100 MHz, 25 °C)

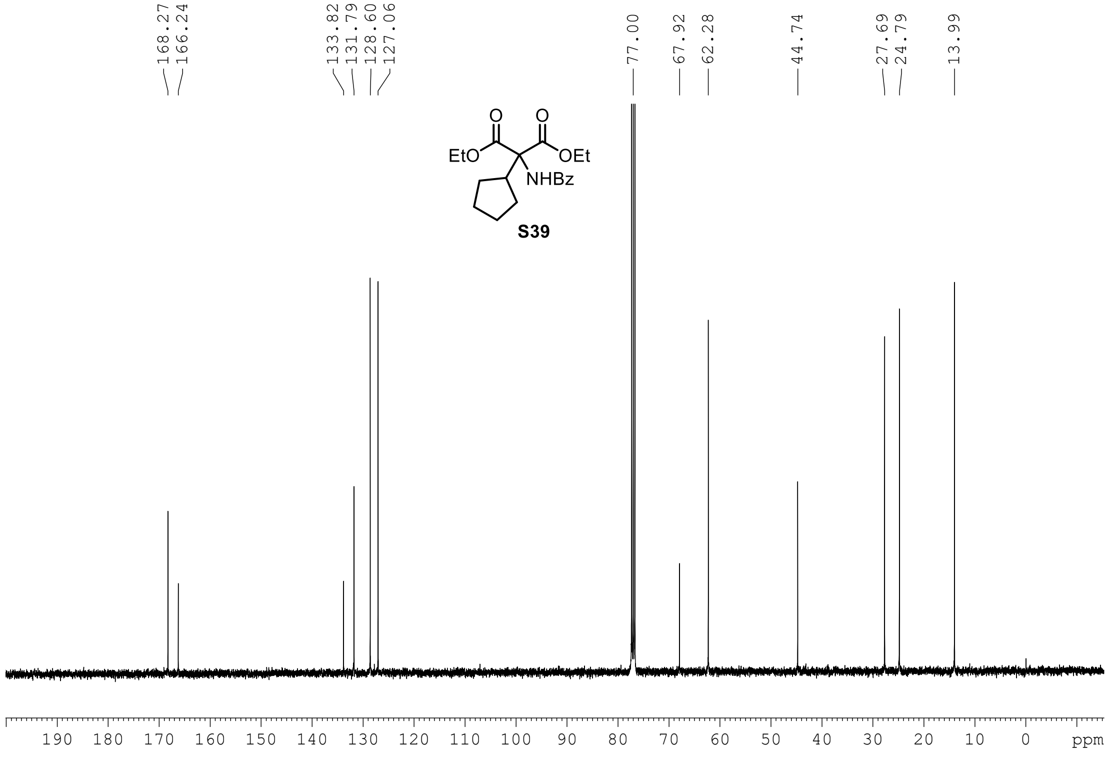

Supplementary Fig. 67. ^{1}H NMR of compound S10 ($CDCl_3$, 400 MHz, 25 °C)

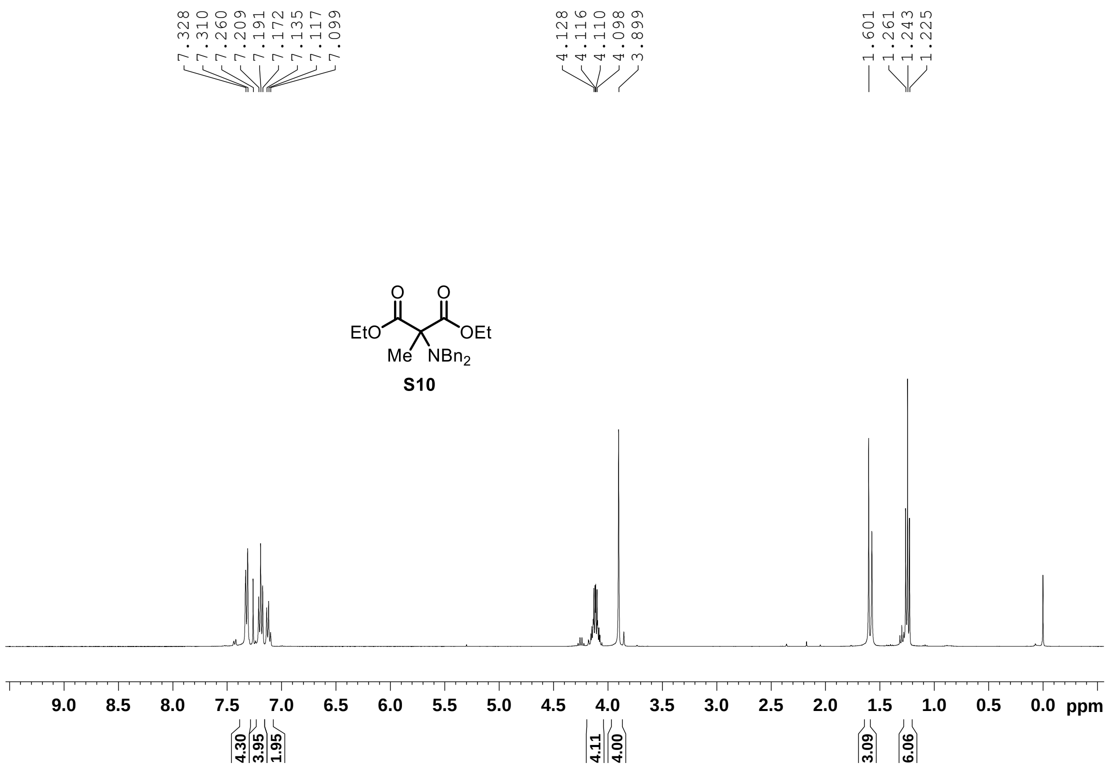

Supplementary Fig. 68. ^{13}C NMR of compound S10 ($CDCl_3$, 100 MHz, 25 °C)

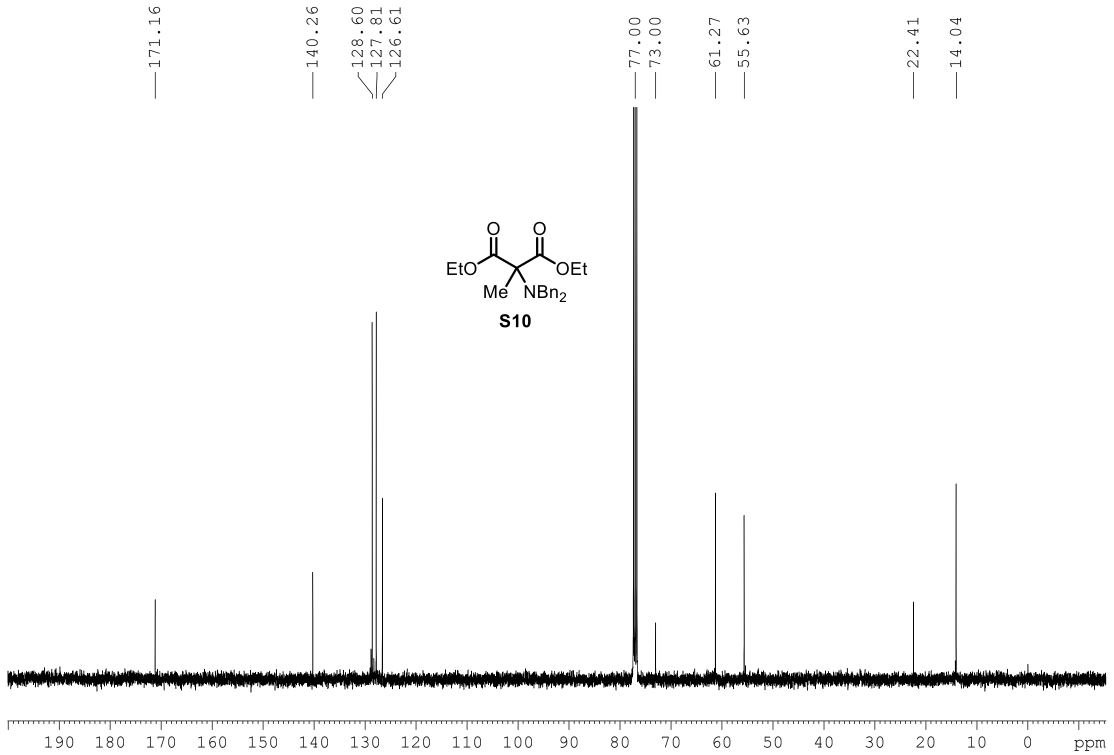

Supplementary Fig. 69. ^{1}H NMR of compound S40 ($CDCl_3$, 400 MHz, 25 °C)

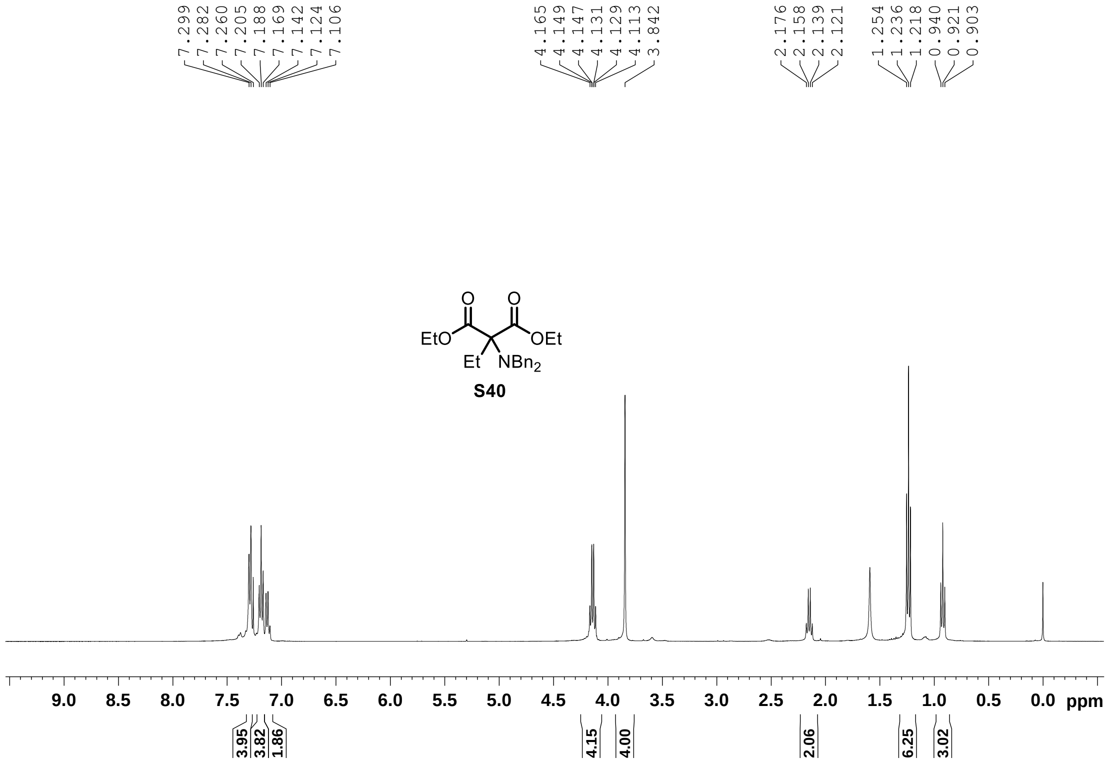

Supplementary Fig. 70. ^{13}C NMR of compound 40 ($CDCl_3$, 100 MHz, 25 °C)

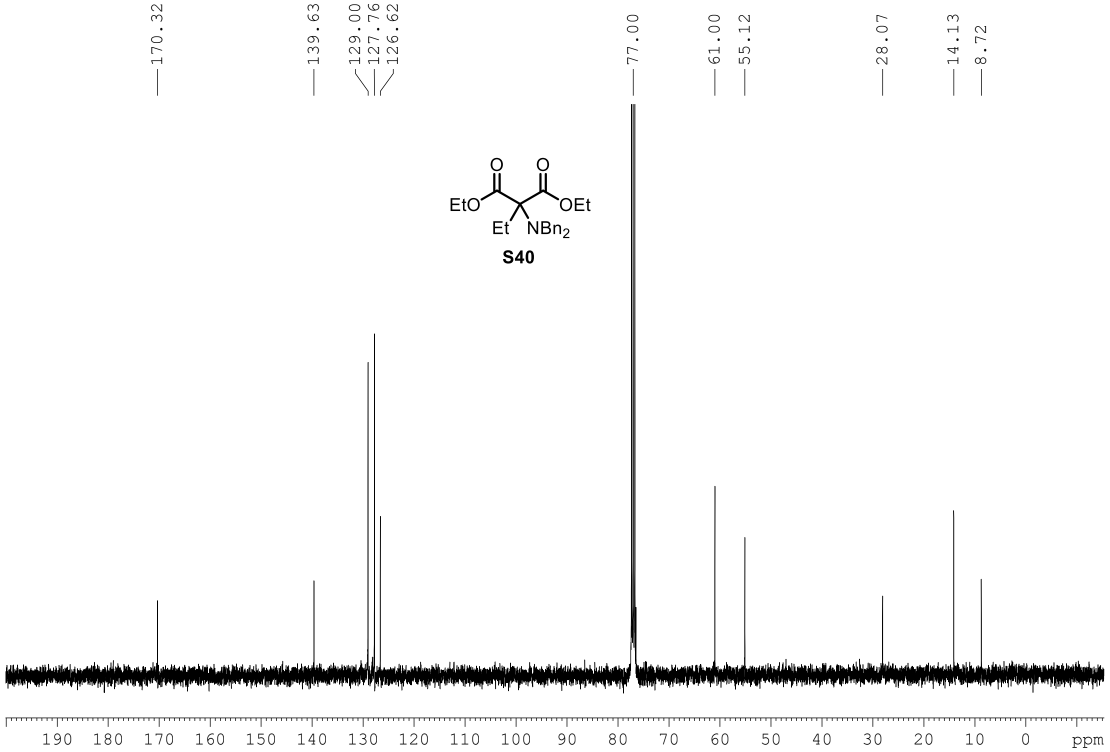

Supplementary Fig. 71. ^{1}H **NMR of compound S41 ($CDCl_3$, 400 MHz, 25 °C)**

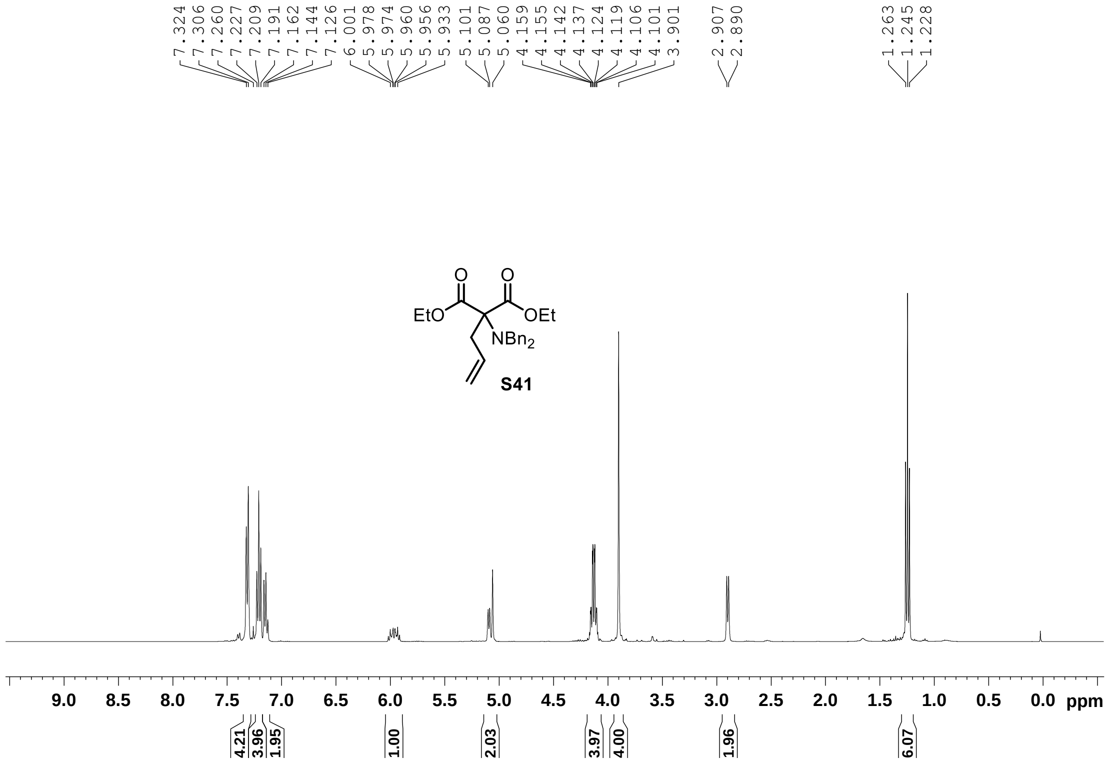

Supplementary Fig. 72. ^{13}C **NMR of compound S41 ($CDCl_3$, 100 MHz, 25 °C)**

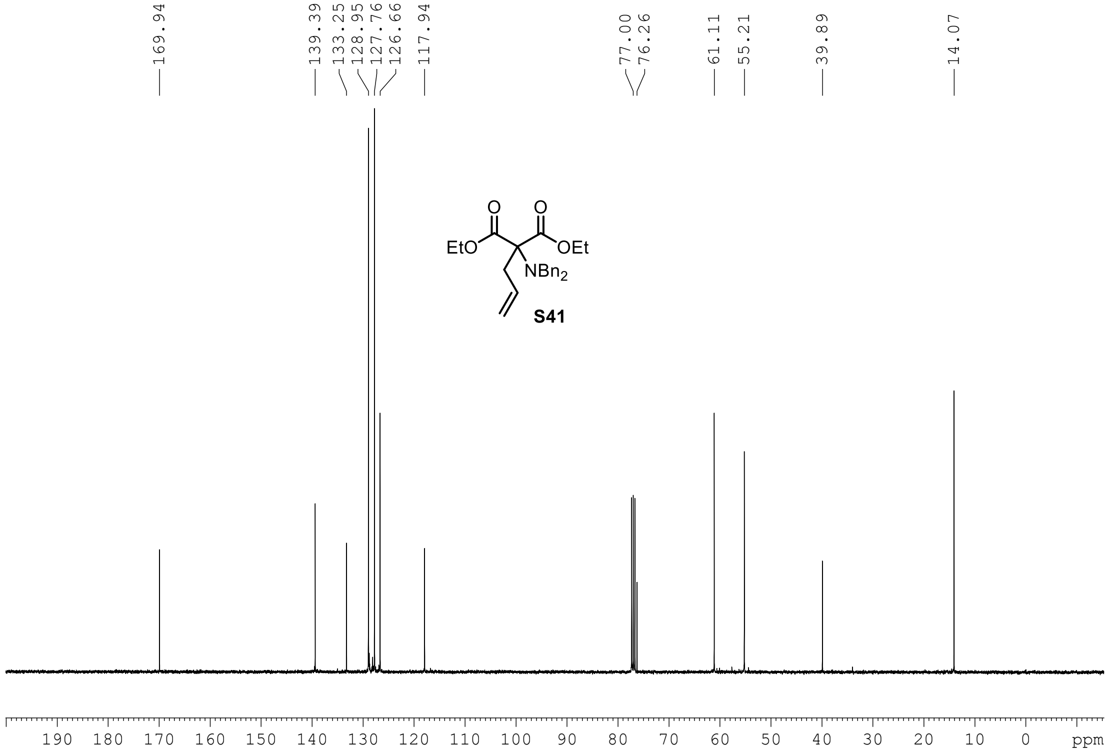

Supplementary Fig. 73. ^{1}H NMR of compound S42 ($CDCl_3$, 400 MHz, 25 °C)

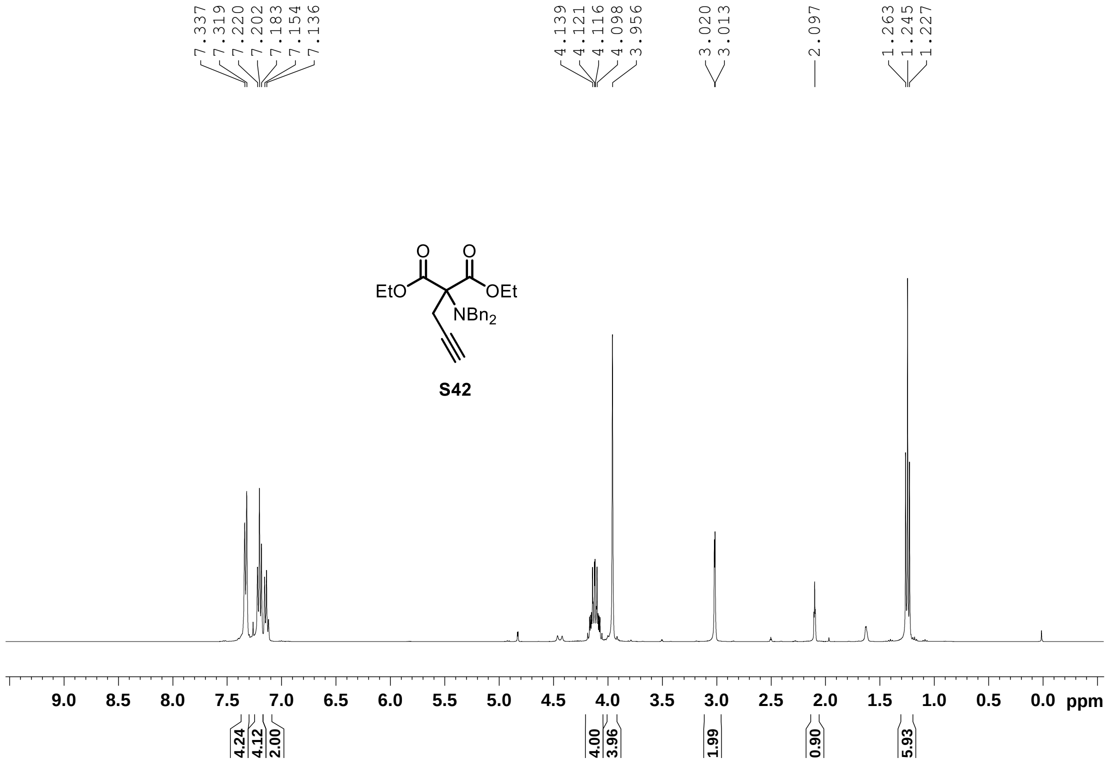

Supplementary Fig. 74. ^{13}C NMR of compound S42 ($CDCl_3$, 100 MHz, 25 °C)

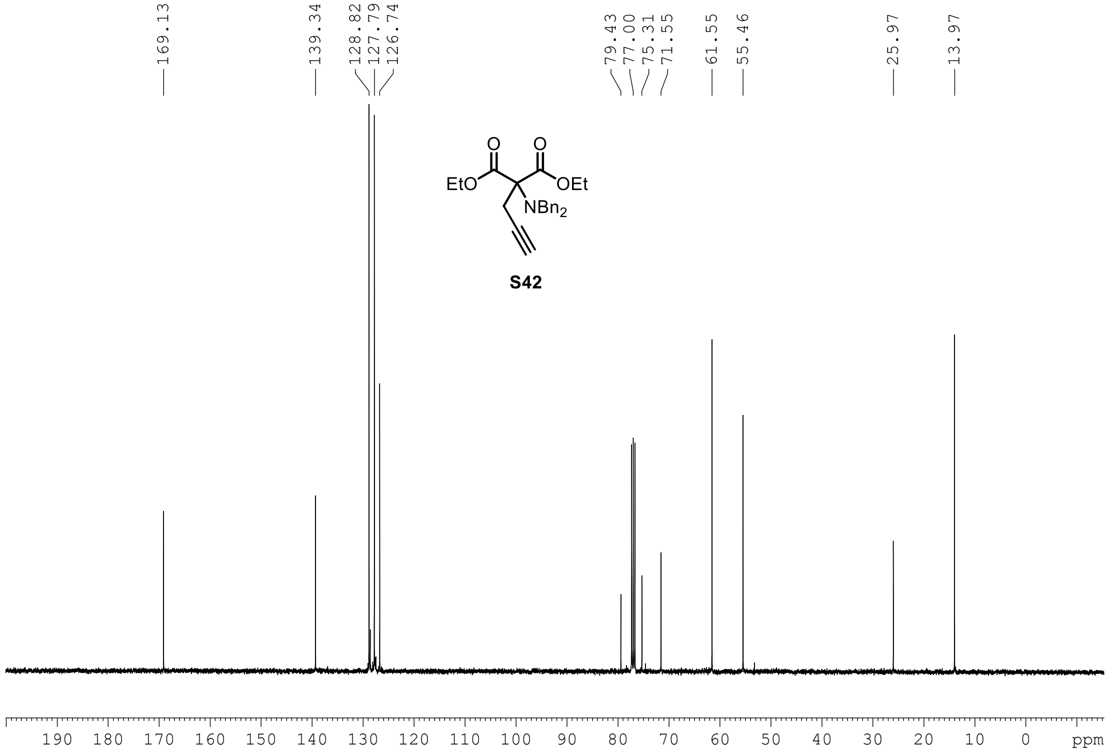

Supplementary Fig. 75. ^{1}H NMR of compound S44 ($CDCl_3$, 400 MHz, 25 °C)

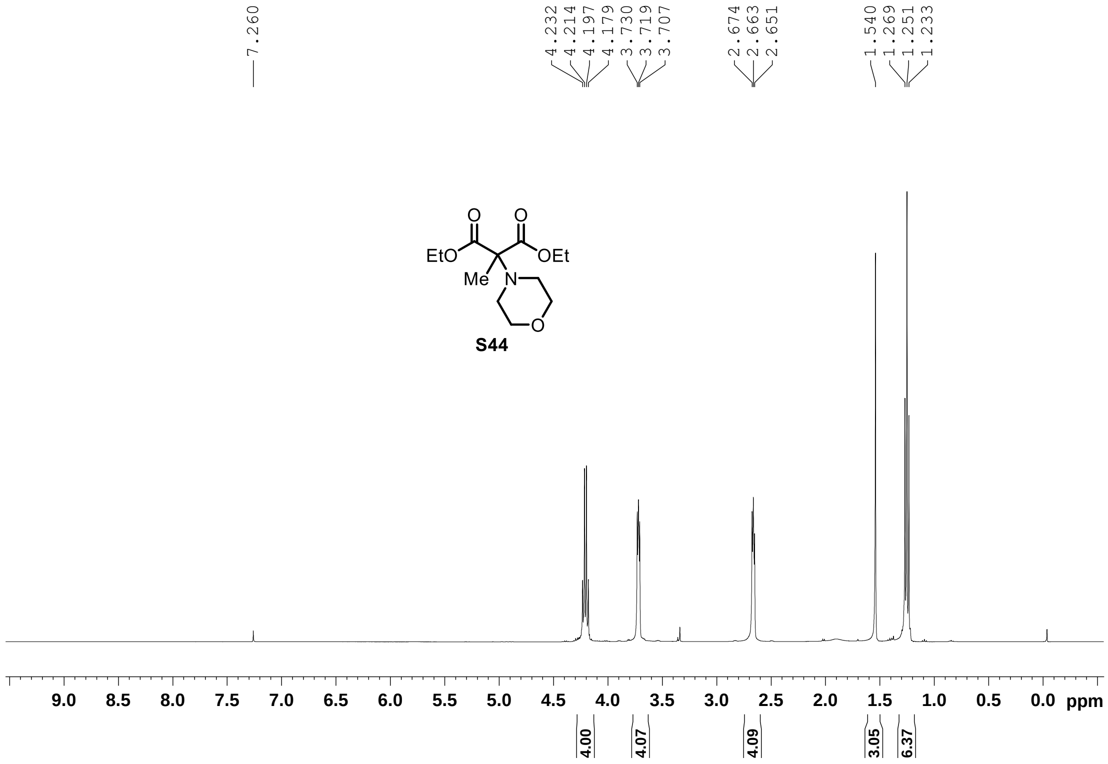

Supplementary Fig. 76. ^{13}C NMR of compound S44 ($CDCl_3$, 100 MHz, 25 °C)

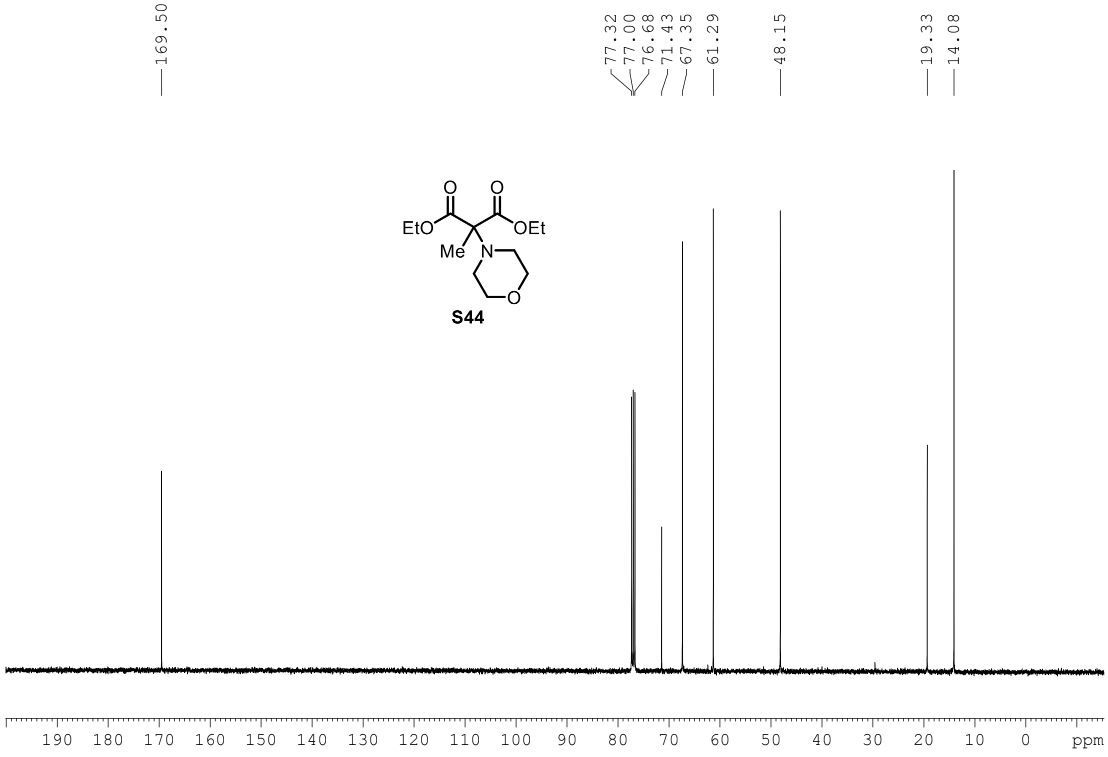

Supplementary Fig. 77. ^{1}H NMR of compound S45 ($CDCl_3$, 400 MHz, 25 °C)

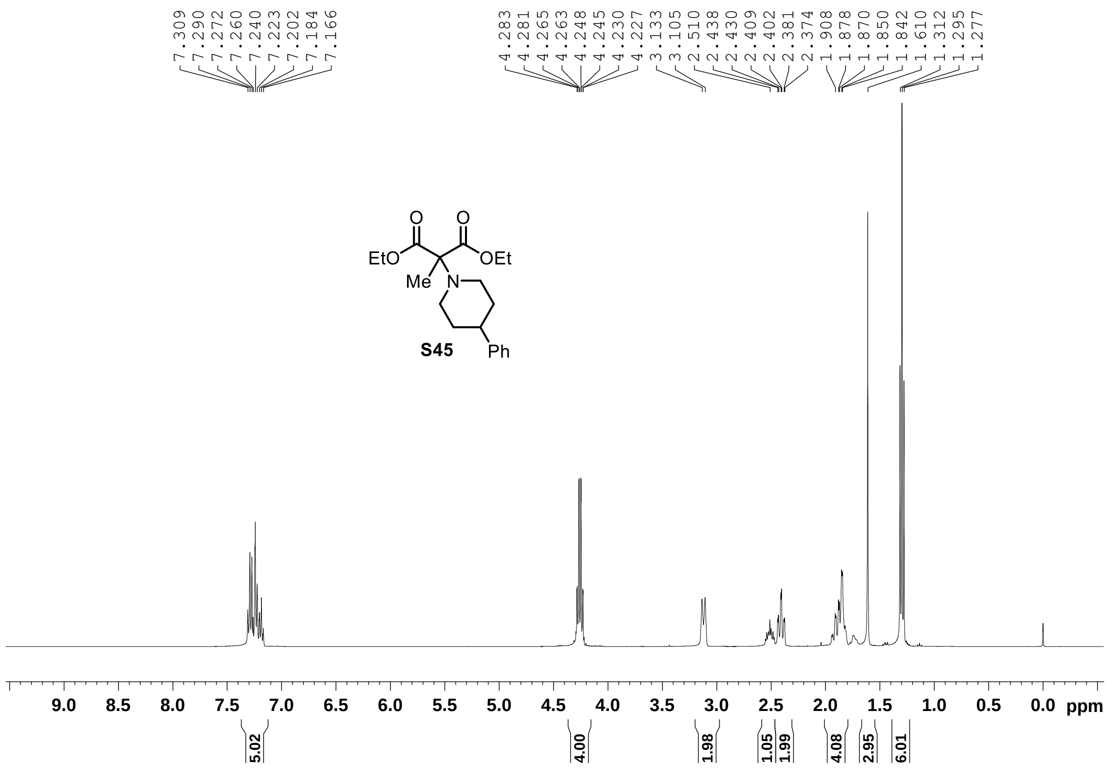

Supplementary Fig. 78. ^{13}C NMR of compound S45 ($CDCl_3$, 100 MHz, 25 °C)

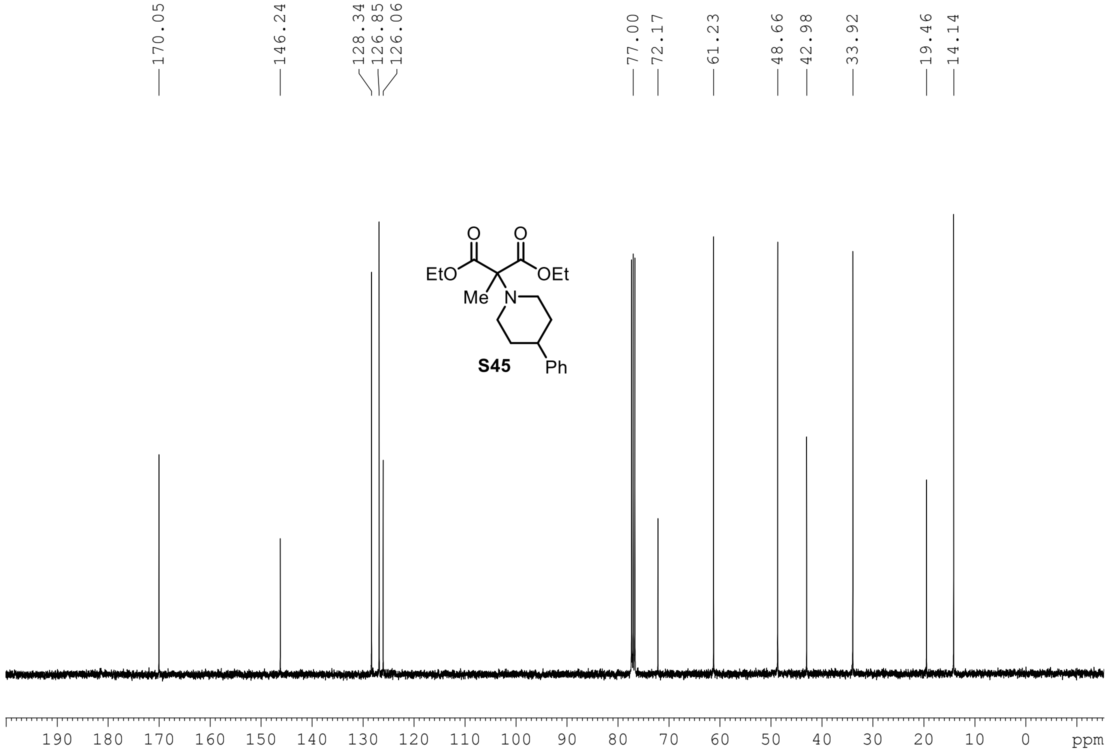

Supplementary Fig. 79. ^{1}H NMR of compound S46 ($CDCl_3$, 400 MHz, 25 °C)

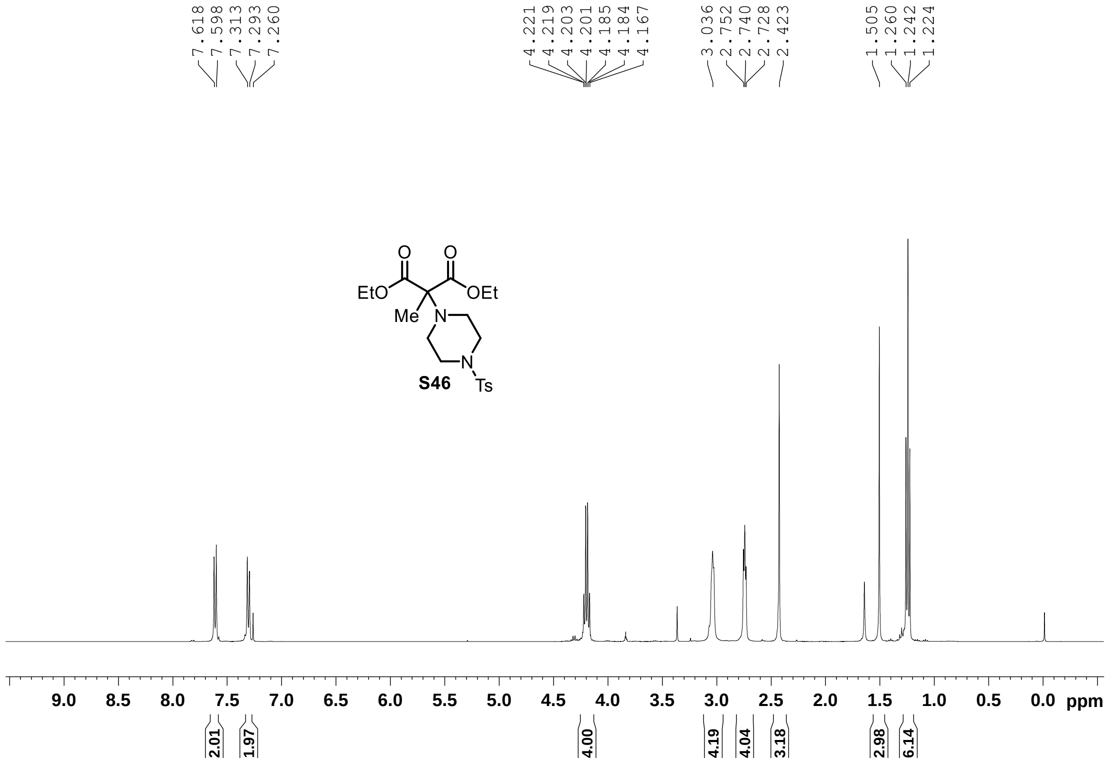

Supplementary Fig. 80. ^{13}C NMR of compound S46 ($CDCl_3$, 100 MHz, 25 °C)

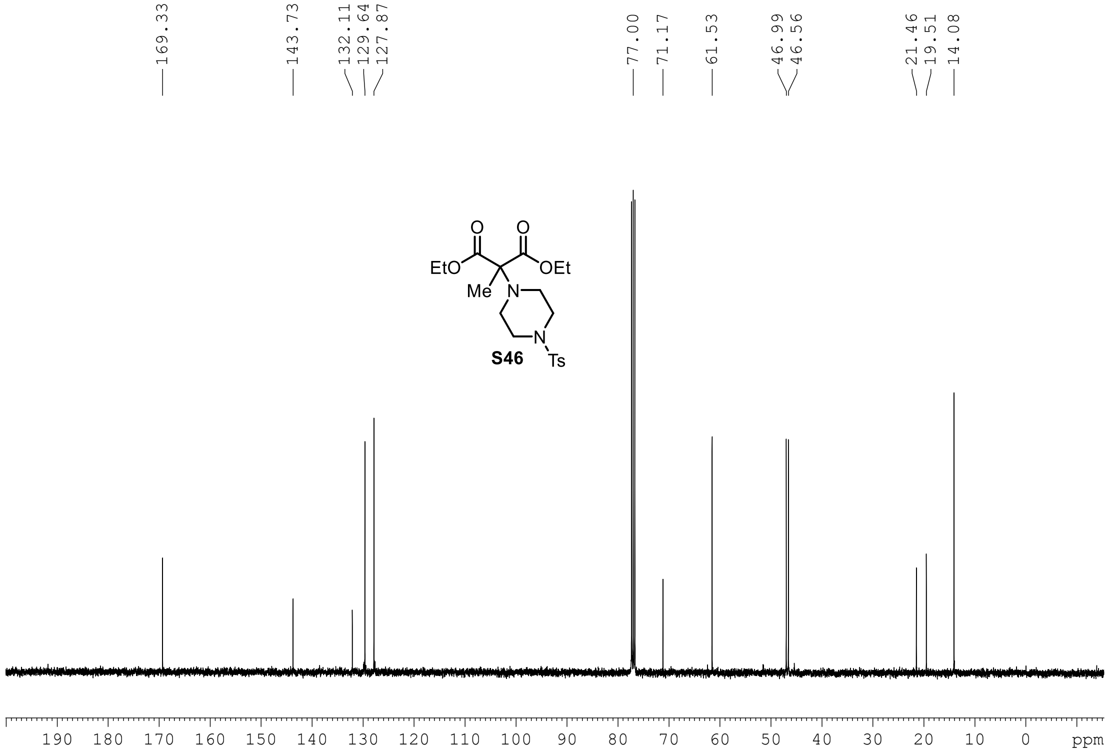

Supplementary Fig. 81. ^{1}H NMR of compound S11 ($CDCl_3$, 400 MHz, 25 °C)

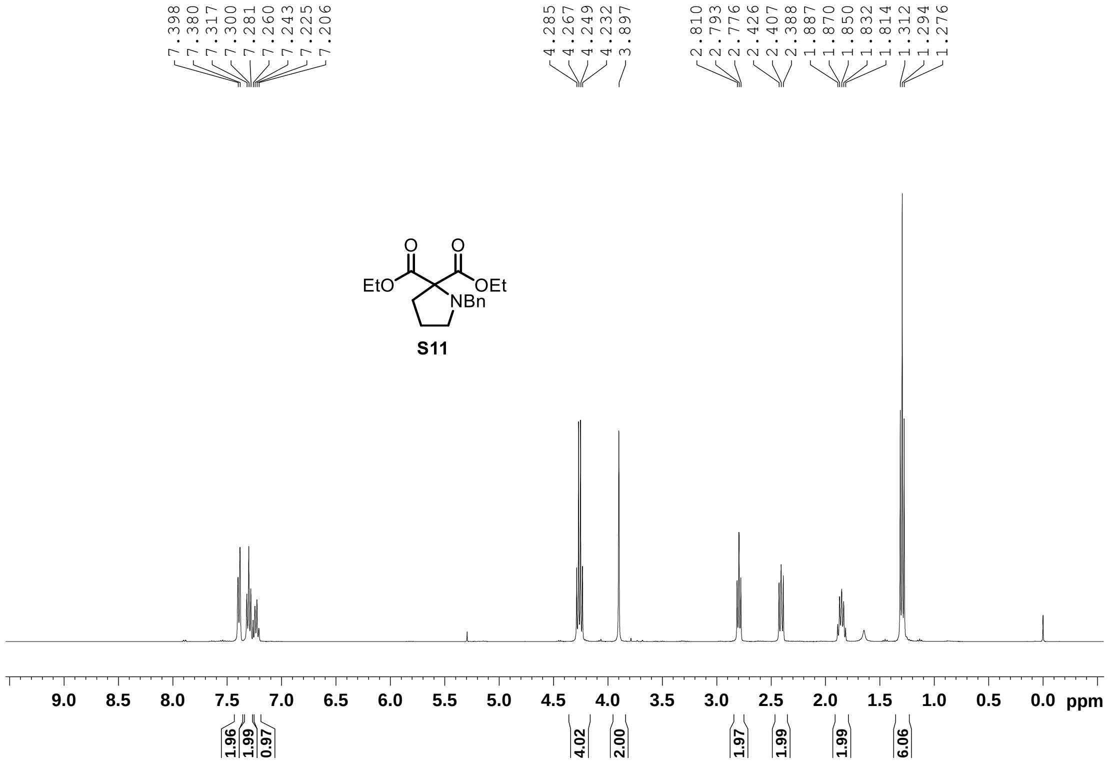

Supplementary Fig. 82. ^{13}C NMR of compound S11 ($CDCl_3$, 100 MHz, 25 °C)

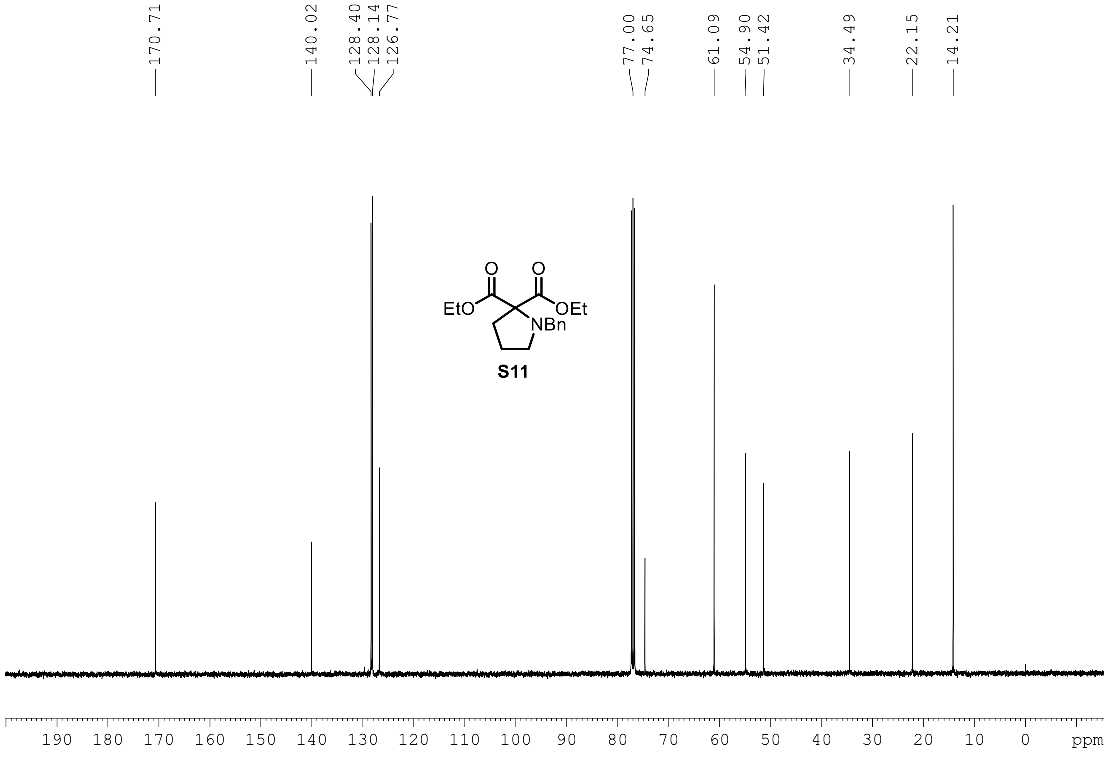

Supplementary Fig. 83. ^{1}H NMR of compound S47 ($CDCl_3$, 400 MHz, 25 °C)

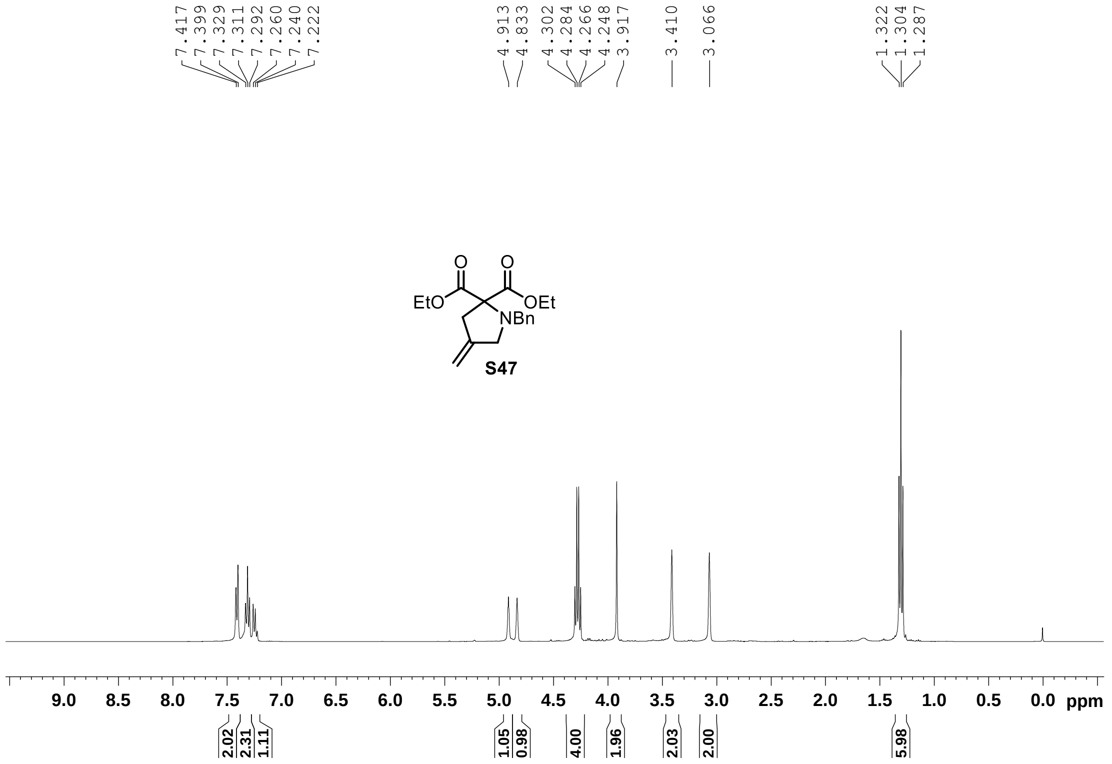

Supplementary Fig. 84. ^{13}C NMR of compound S47 ($CDCl_3$, 125 MHz, 25 °C)

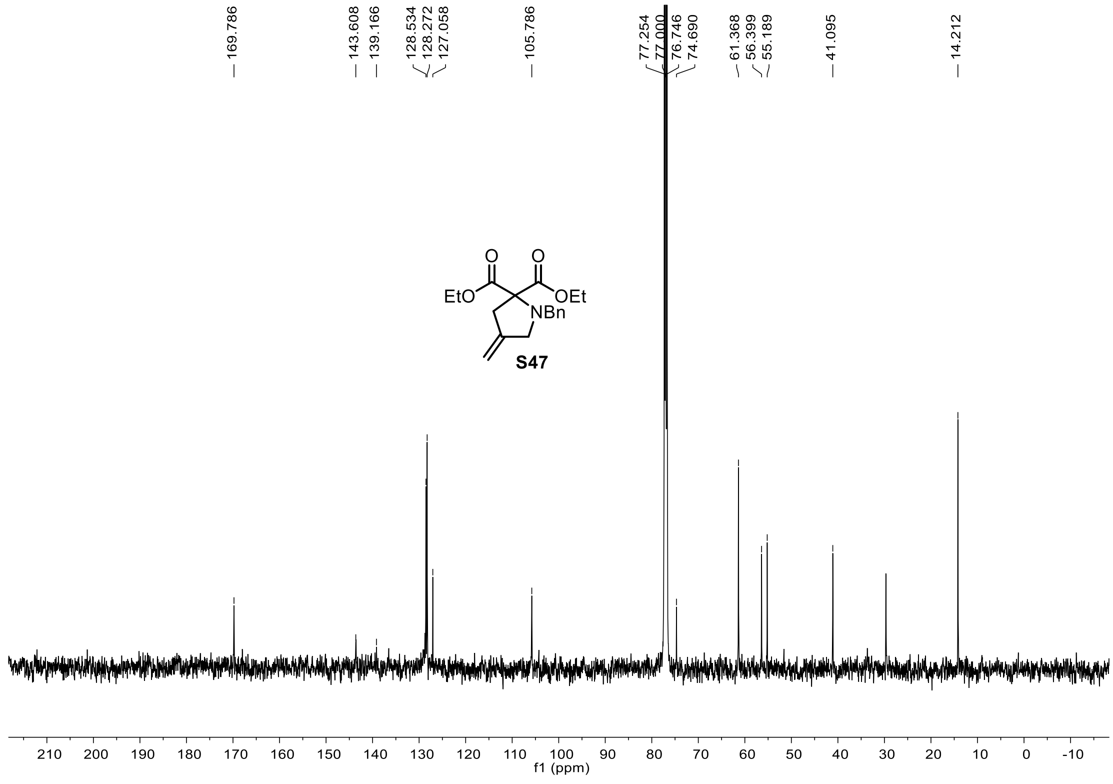

Supplementary Fig. 85. **^{1}H NMR of compound S48 ($CDCl_3$, 400 MHz, 25 °C)**

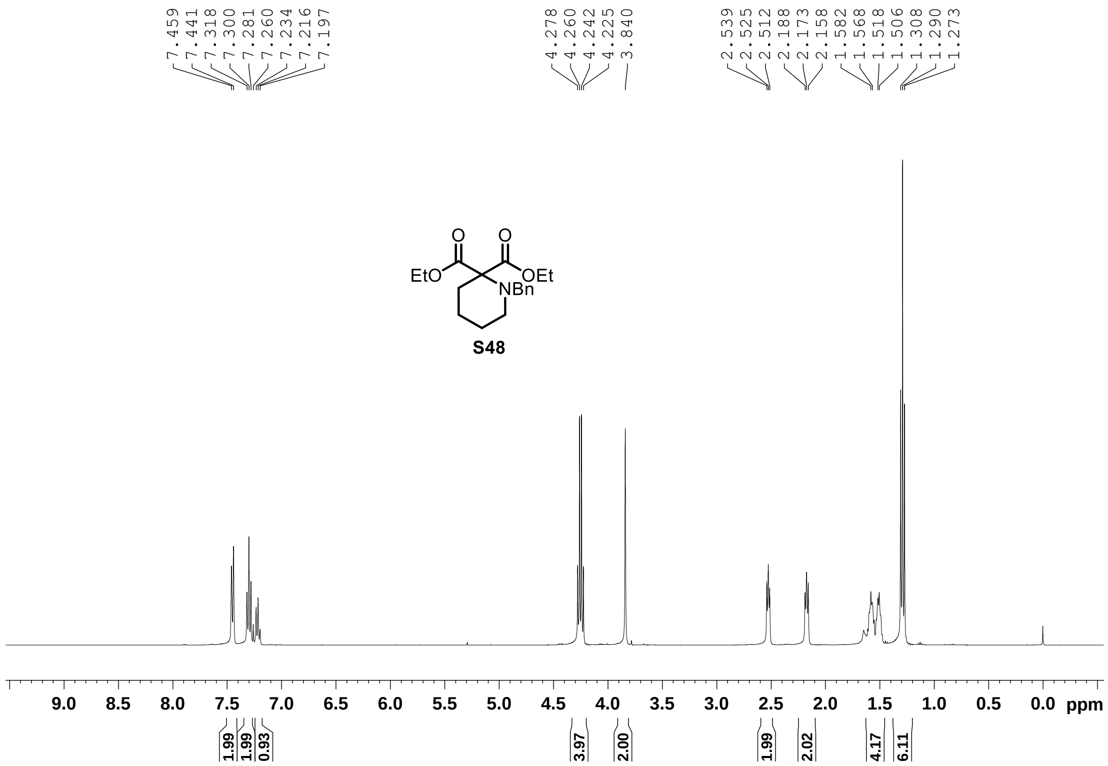

Supplementary Fig. 86. **^{13}C NMR of compound S48 ($CDCl_3$, 100 MHz, 25 °C)**

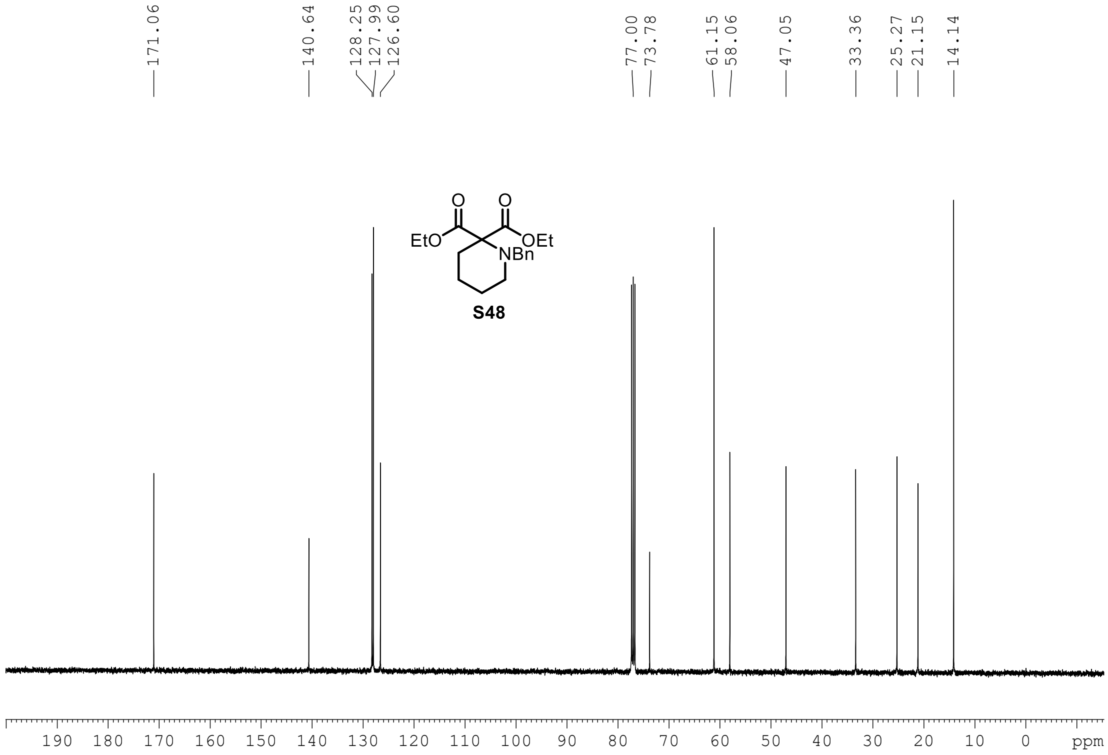

Supplementary Fig. 87. ^{1}H NMR of compound S49 ($CDCl_3$, 400 MHz, 25 °C)

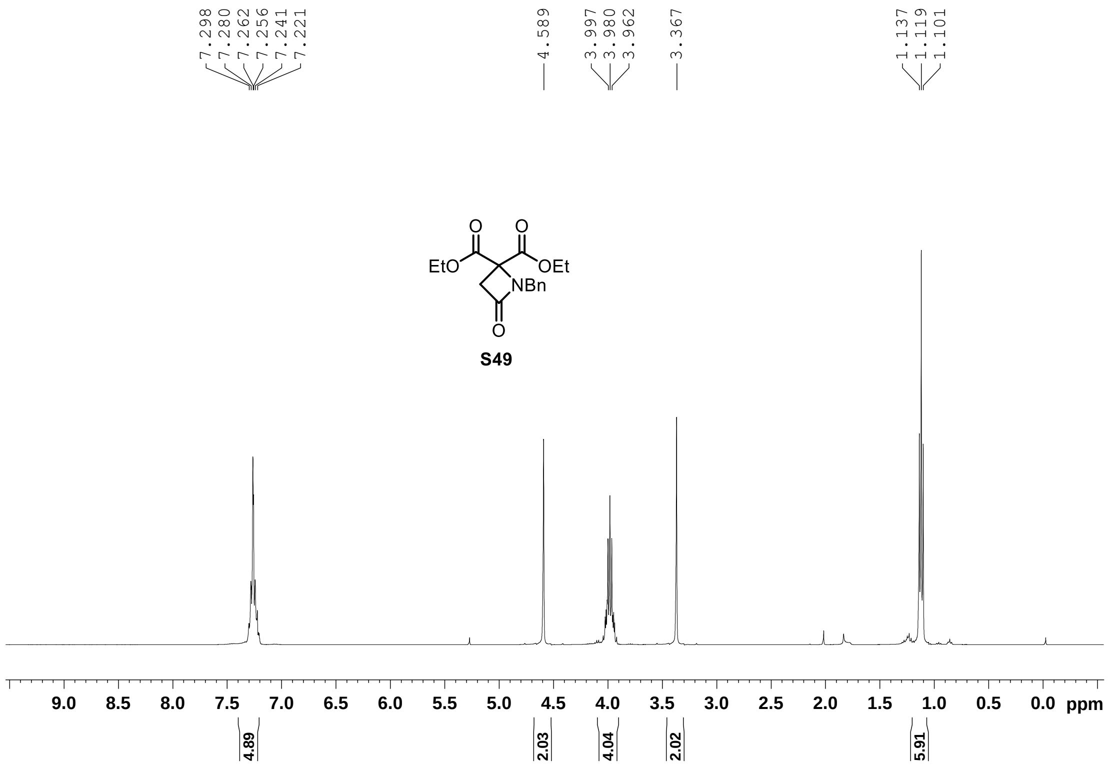

Supplementary Fig. 88. ^{13}C NMR of compound S49 ($CDCl_3$, 100 MHz, 25 °C)

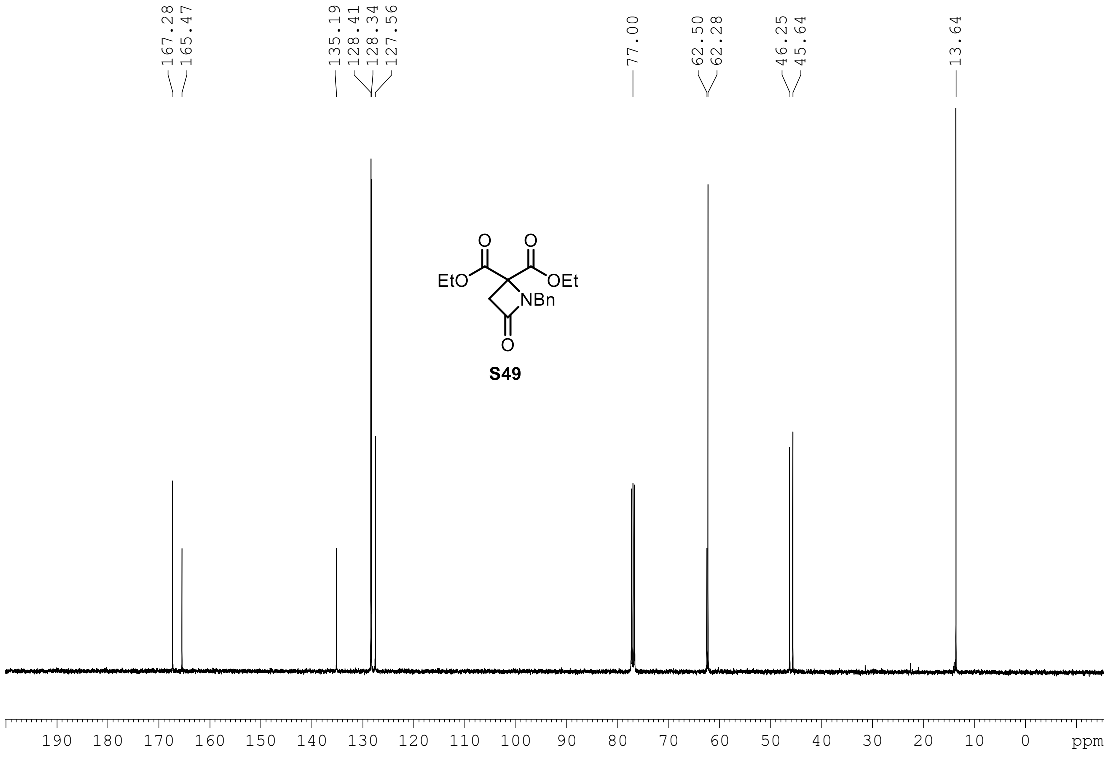

Supplementary Fig. 89. ^{1}H NMR of compound S50 ($CDCl_3$, 400 MHz, 25 °C)

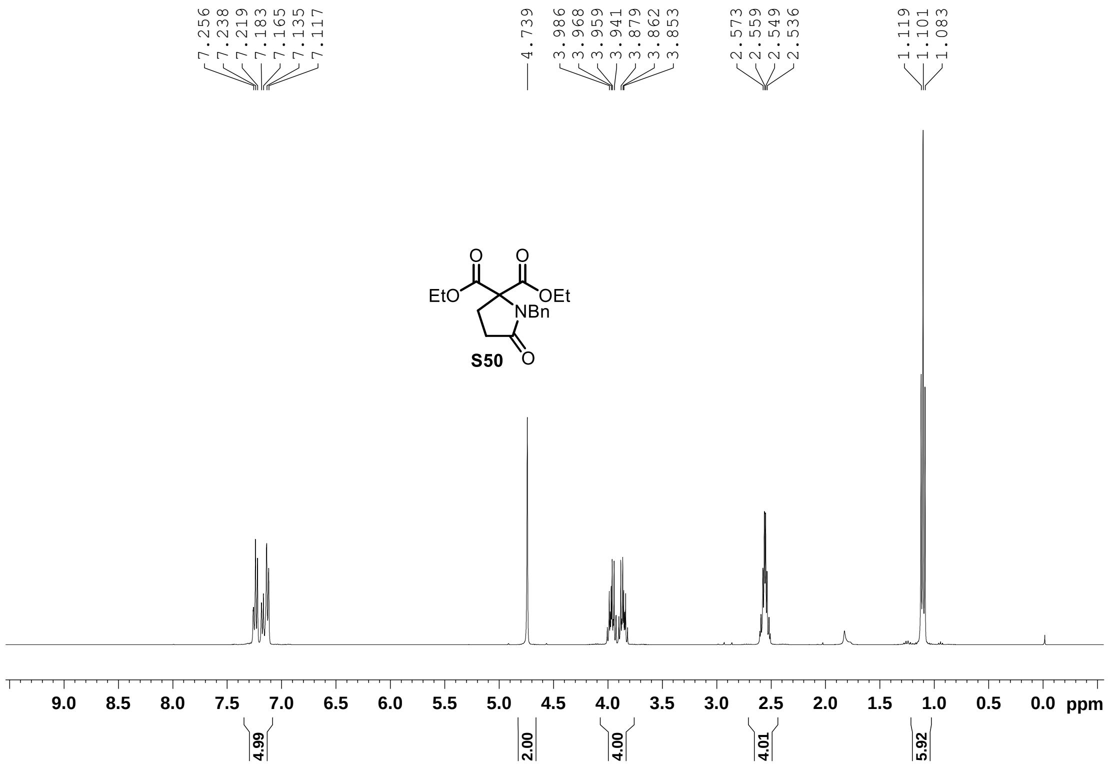

Supplementary Fig. 90. ^{13}C NMR of compound S50 ($CDCl_3$, 100 MHz, 25 °C)

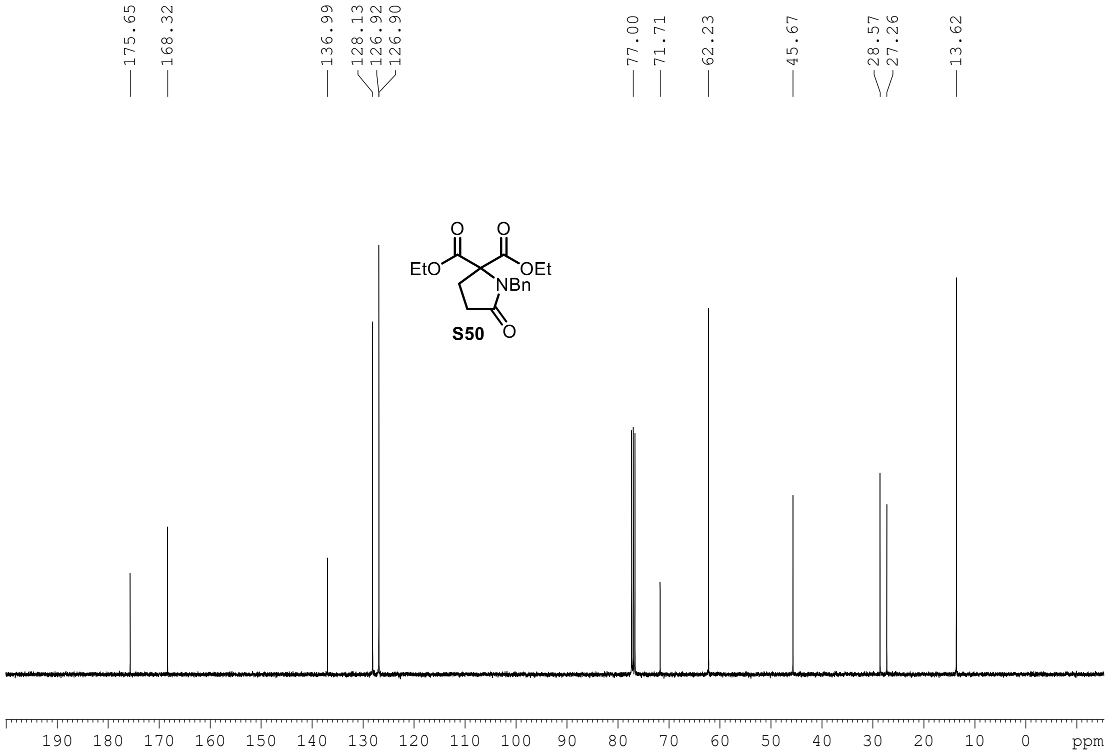

Supplementary Fig. 91. [1]**H NMR of compound S51 ($CDCl_3$, 400 MHz, 25 °C)**

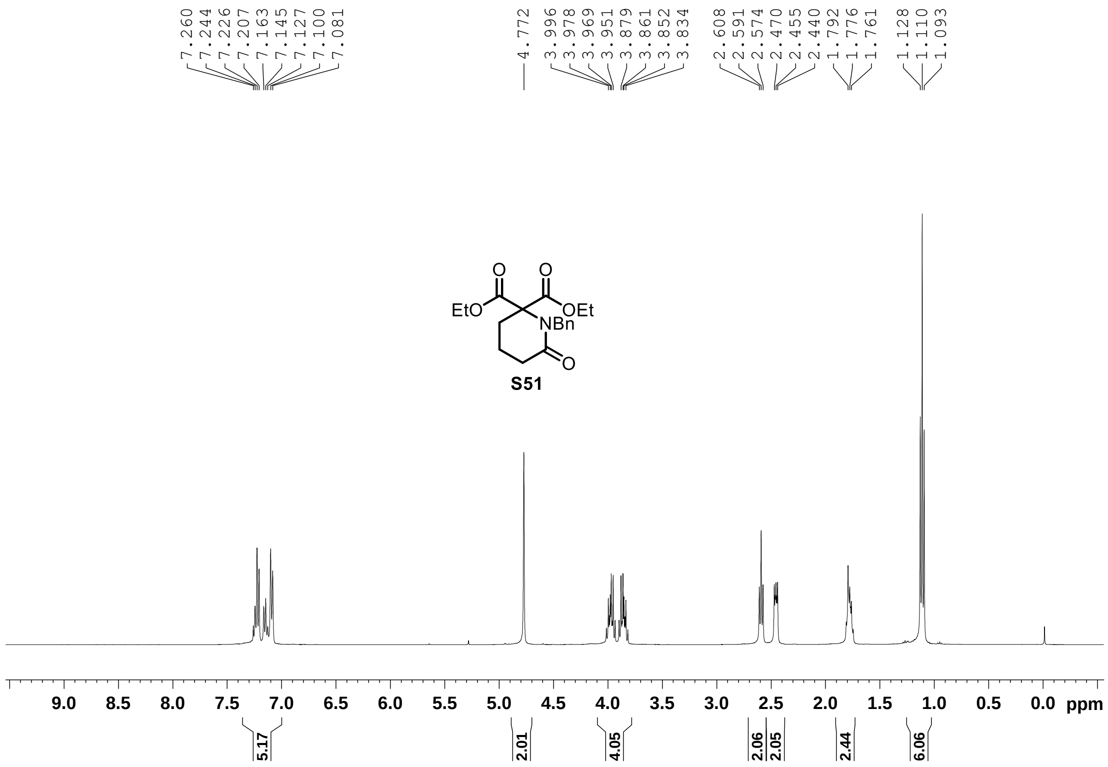

Supplementary Fig. 92. [13]**C NMR of compound S51 ($CDCl_3$, 100 MHz, 25 °C)**

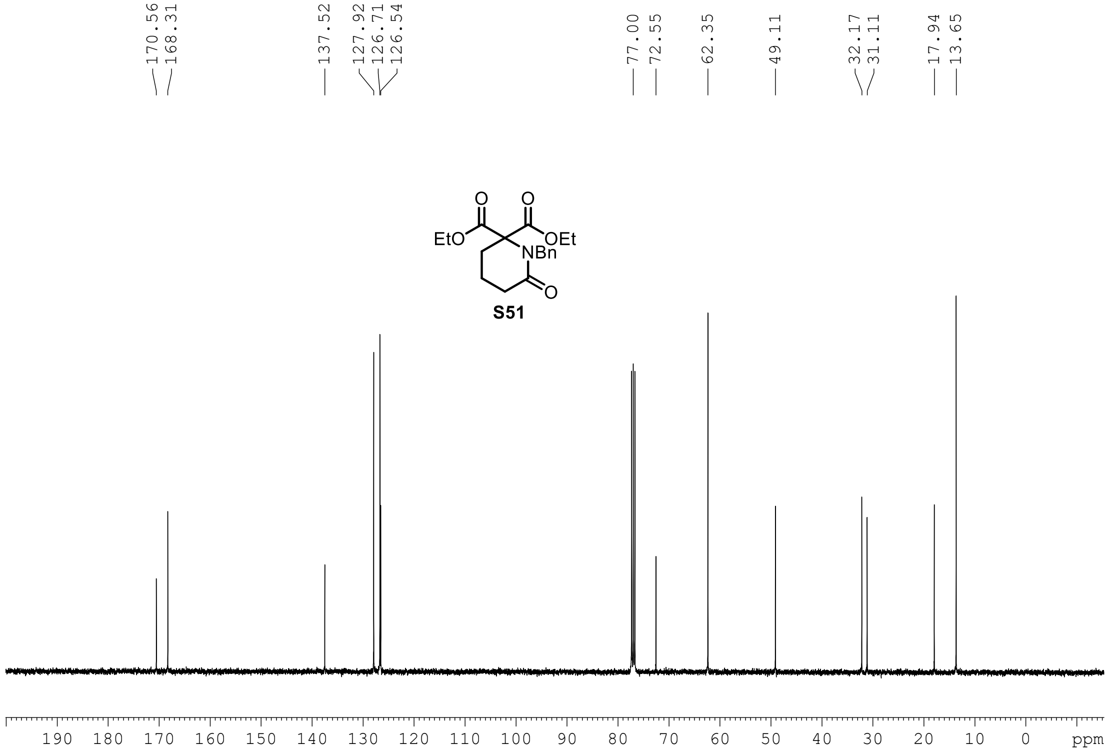

Supplementary Fig. 93. ^{1}H NMR of compound S52 ($CDCl_3$, 400 MHz, 25 °C)

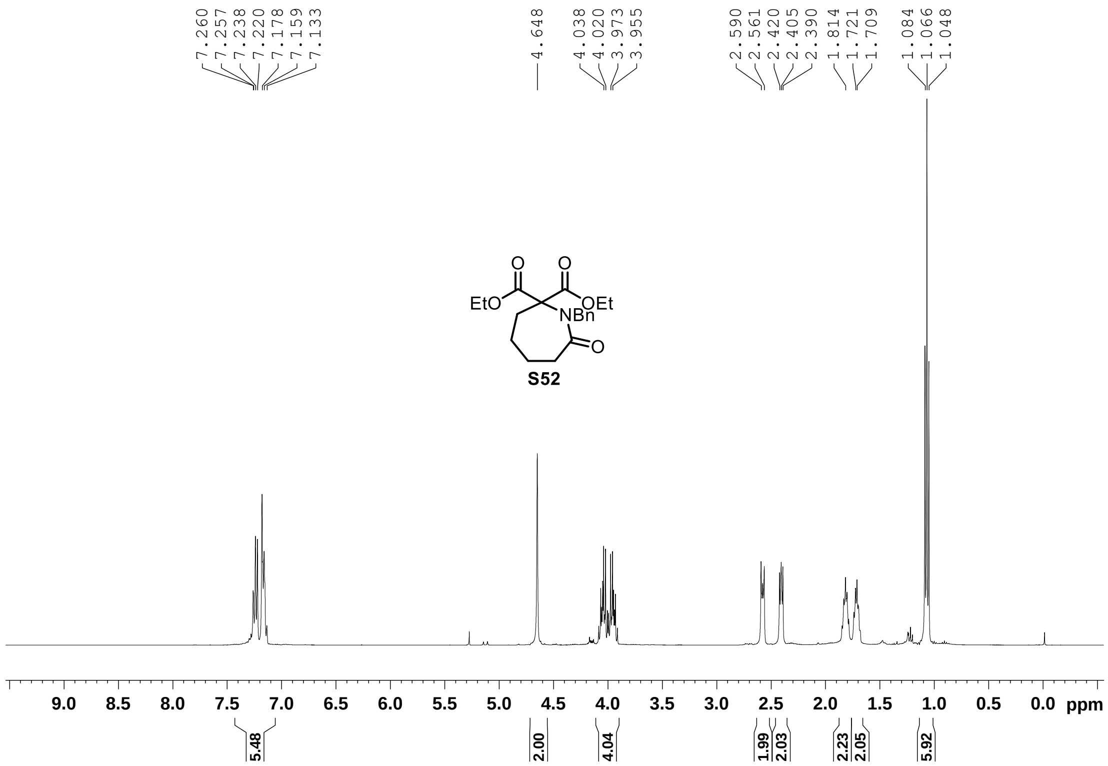

Supplementary Fig. 94. ^{13}C NMR of compound S52 ($CDCl_3$, 100 MHz, 25 °C)

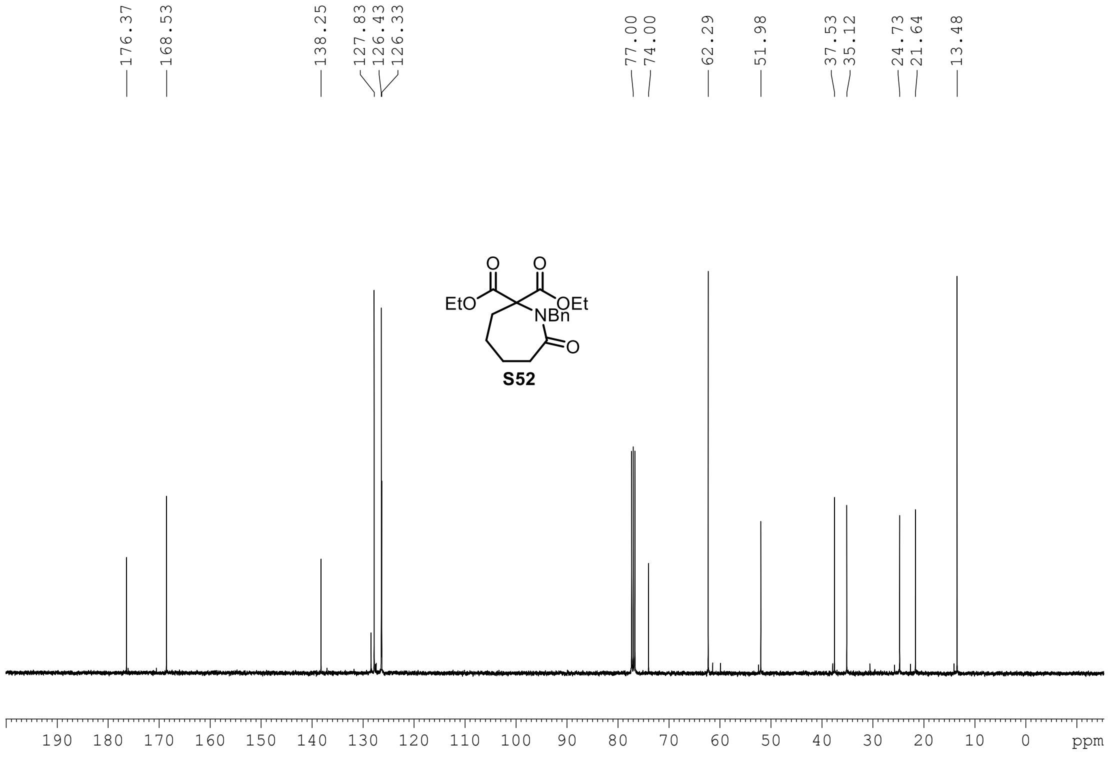

Supplementary Fig. 95. ^{1}H NMR of compound S12 ($CDCl_3$, 400 MHz, 25 °C)

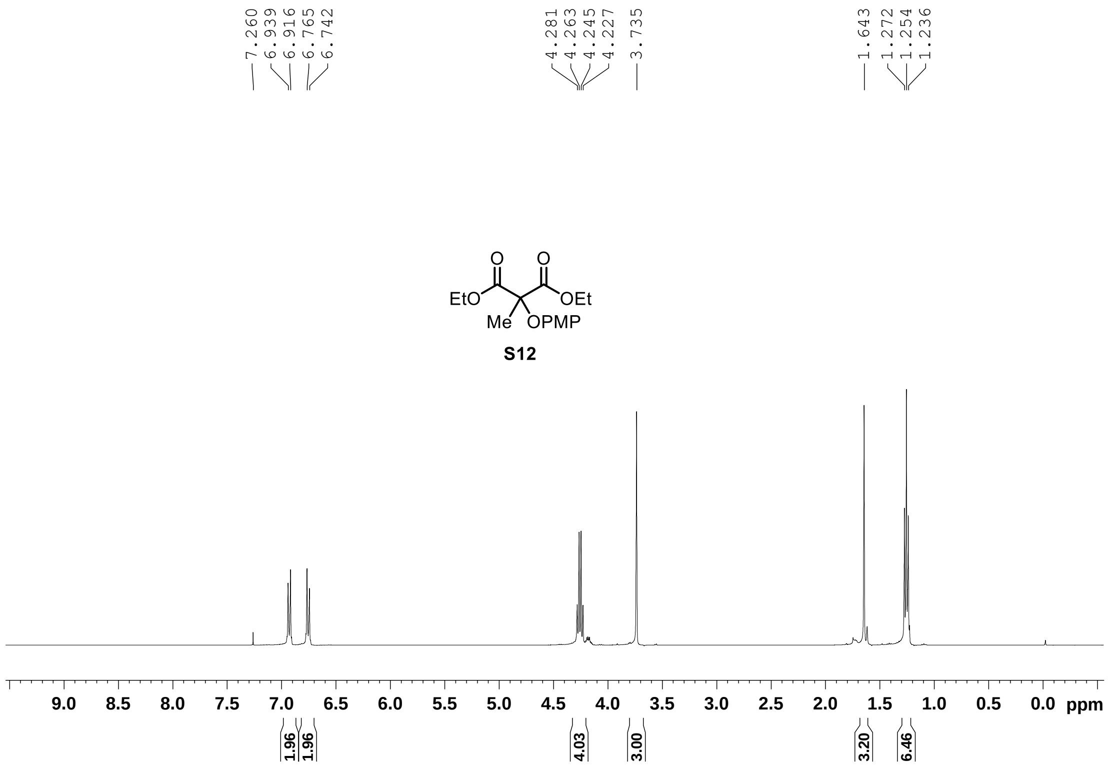

Supplementary Fig. 96. ^{13}C NMR of compound S12 ($CDCl_3$, 100 MHz, 25 °C)

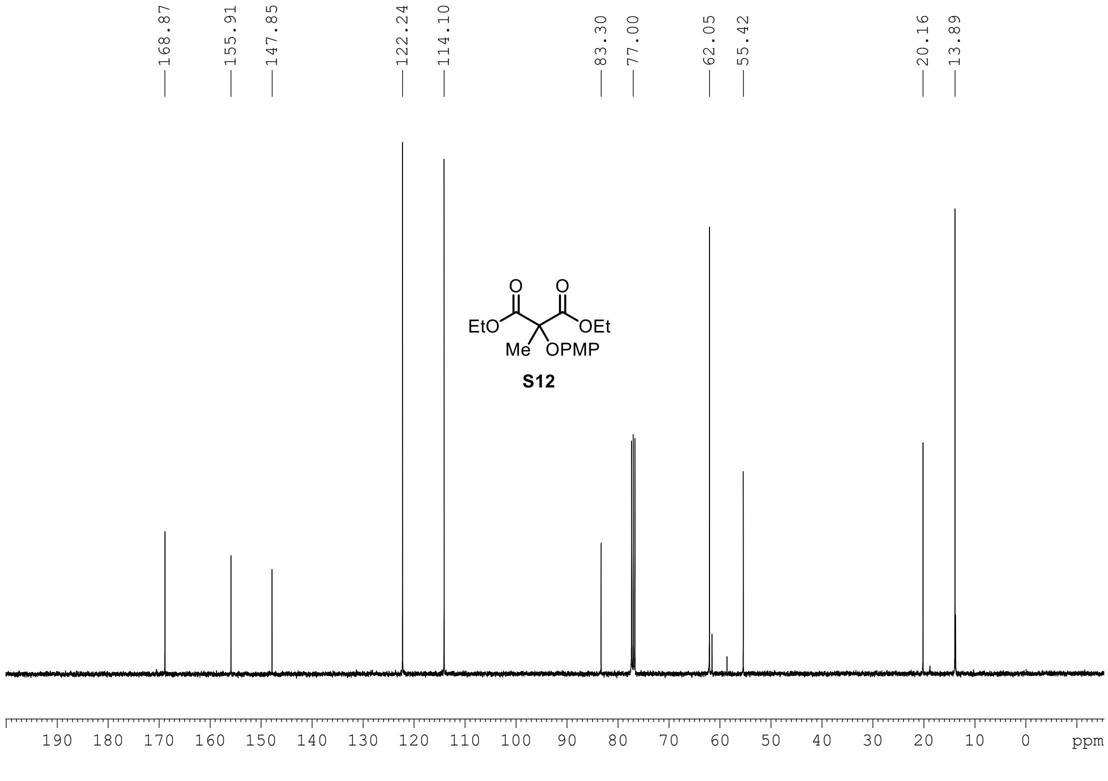

Supplementary Fig. 97. ^{1}H NMR of compound S53 ($CDCl_3$, 400 MHz, 25 °C)

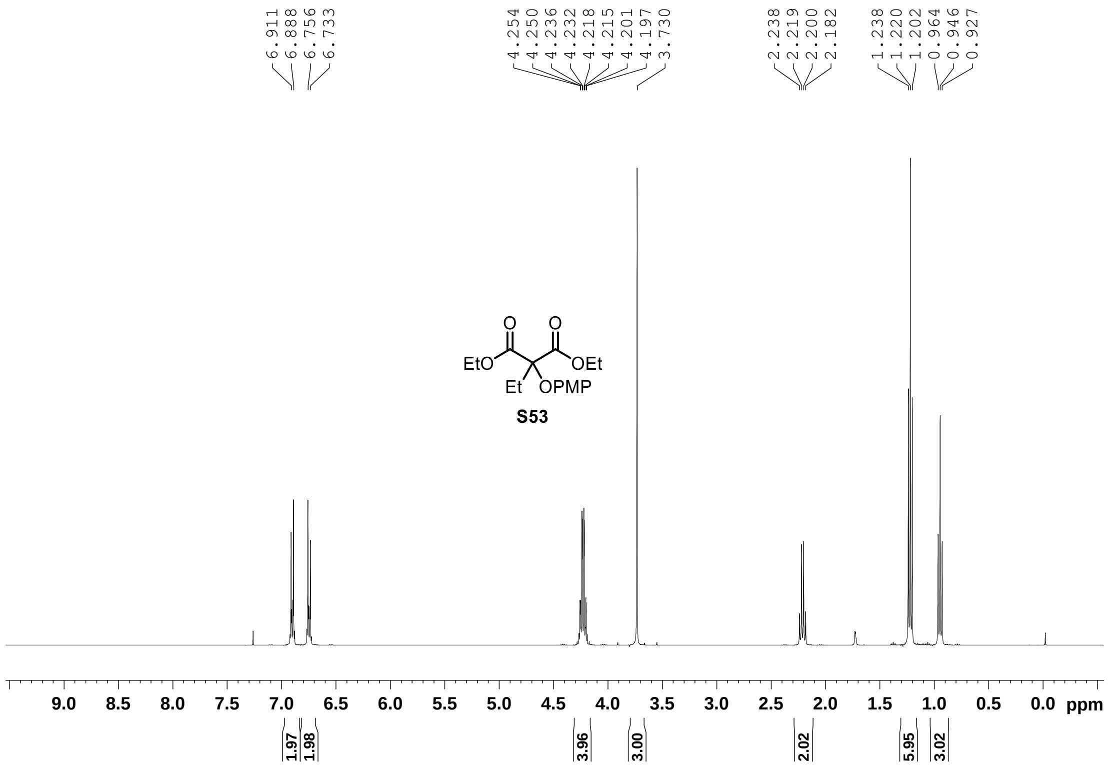

Supplementary Fig. 98. ^{13}C NMR of compound S53 ($CDCl_3$, 100 MHz, 25 °C)

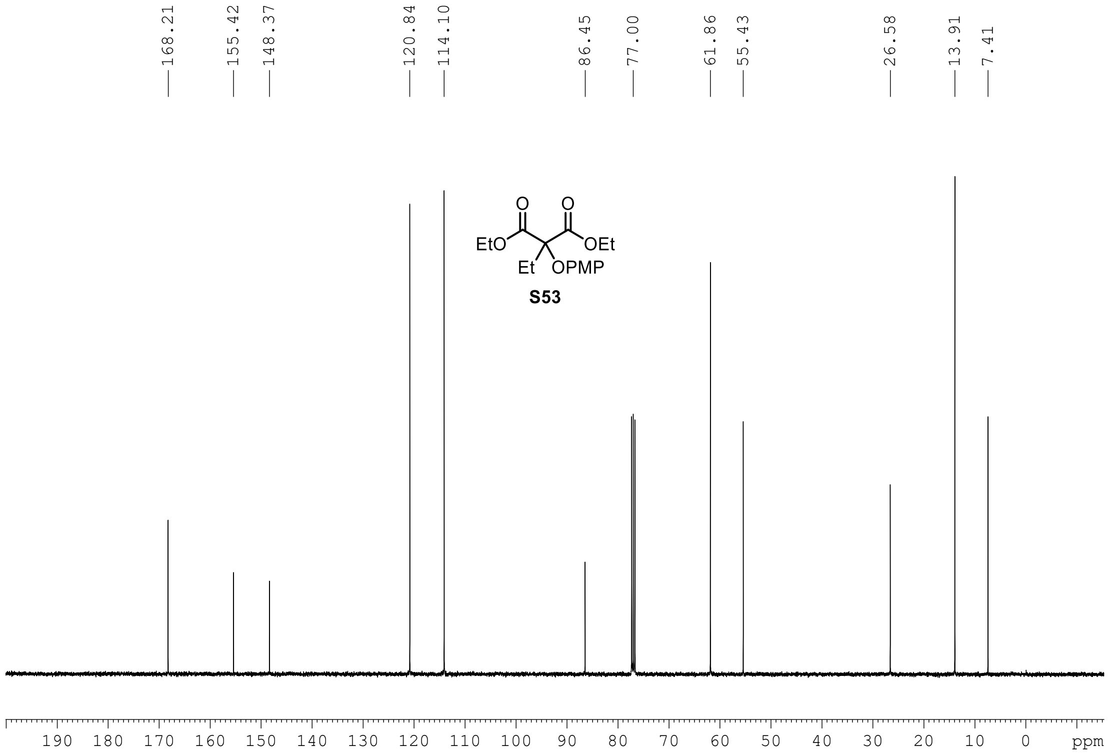

Supplementary Fig. 99. ^{1}H NMR of compound S54 ($CDCl_3$, 400 MHz, 25 °C)

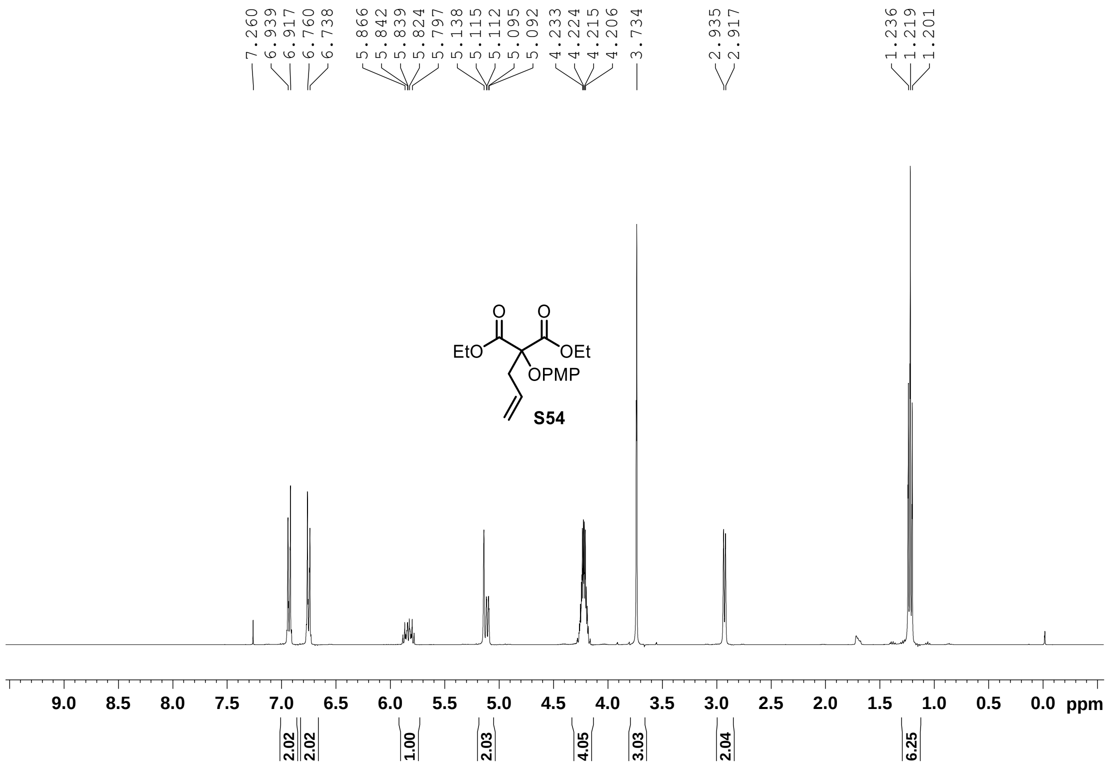

Supplementary Fig. 100. ^{13}C NMR of compound S54 ($CDCl_3$, 100 MHz, 25 °C)

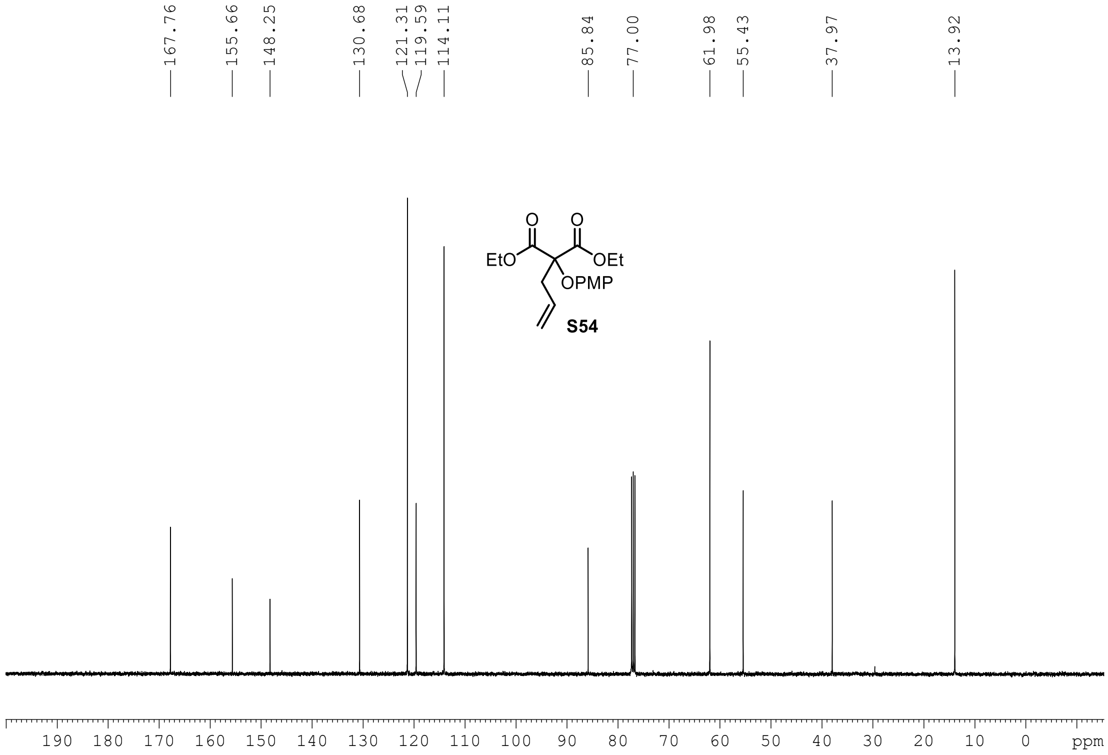

Supplementary Fig. 101. ^{1}H NMR of compound S55 ($CDCl_3$, 400 MHz, 25 °C)

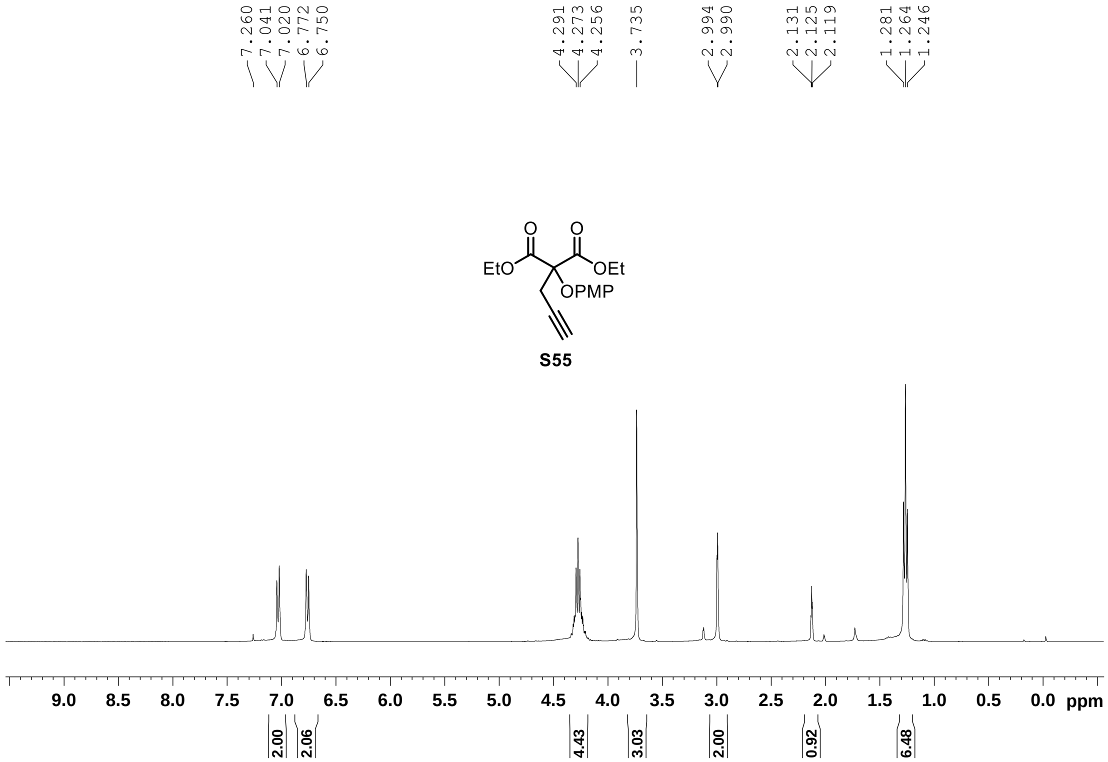

Supplementary Fig. 102. ^{13}C NMR of compound S55 ($CDCl_3$, 100 MHz, 25 °C)

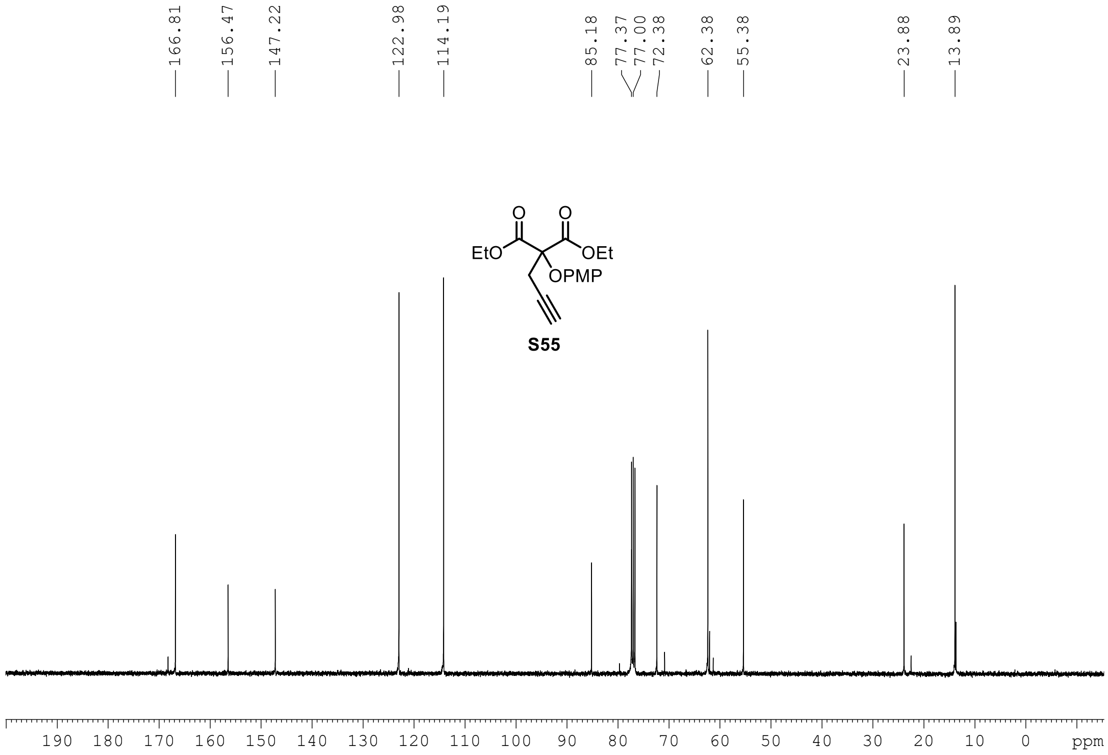

Supplementary Fig. 103. ^{1}H NMR of compound S13 (CDCl$_3$, 400 MHz, 25 °C)

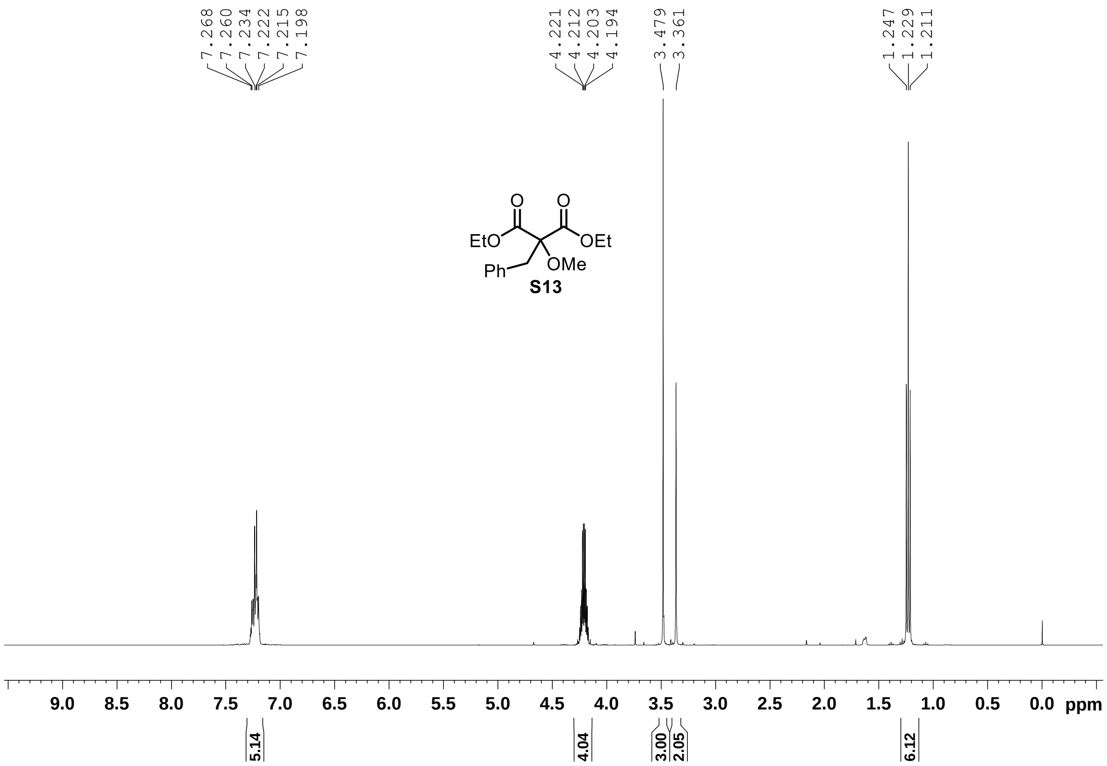

Supplementary Fig. 104. ^{13}C NMR of compound S13 (CDCl$_3$, 100 MHz, 25 °C)

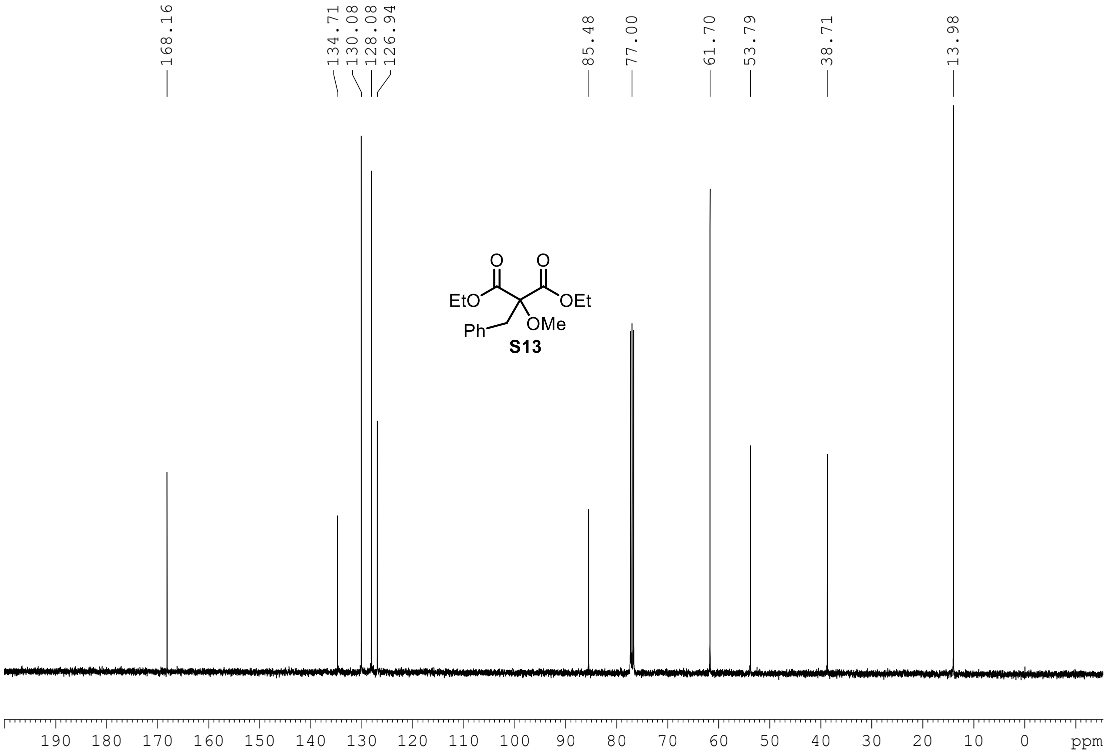

Supplementary Fig. 105. ^{1}H NMR of compound S57 ($CDCl_3$, 400 MHz, 25 °C)

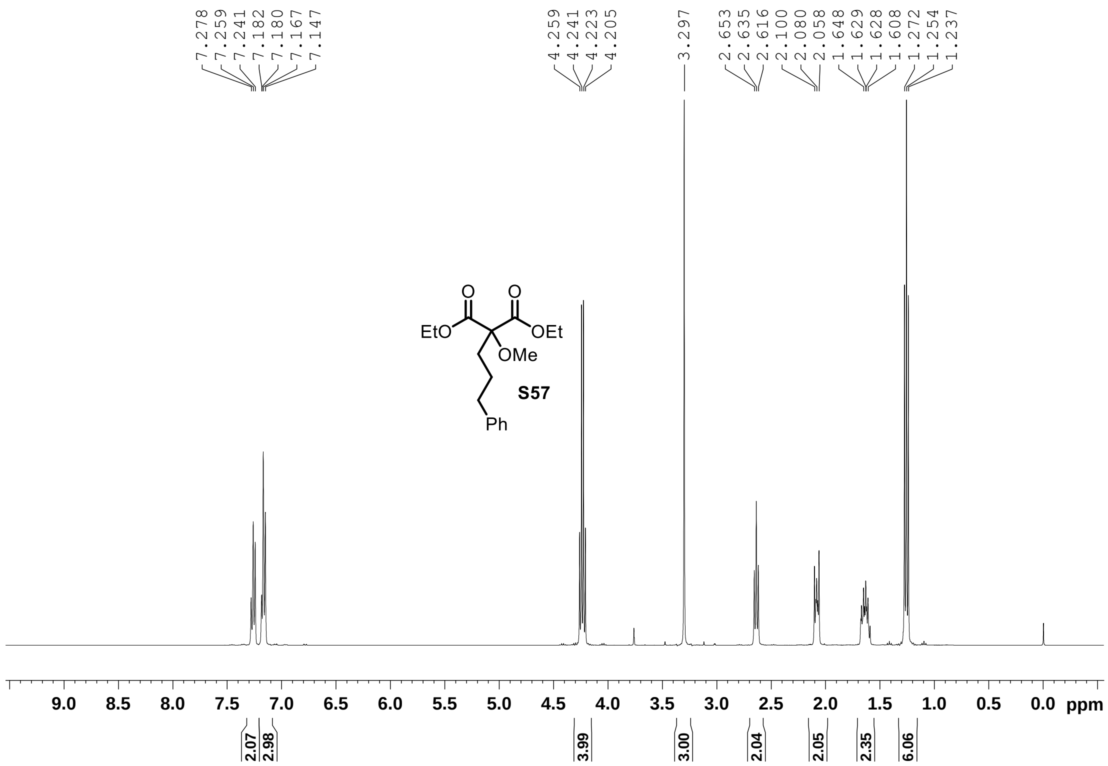

Supplementary Fig. 106. ^{13}C NMR of compound 57 ($CDCl_3$, 100 MHz, 25 °C)

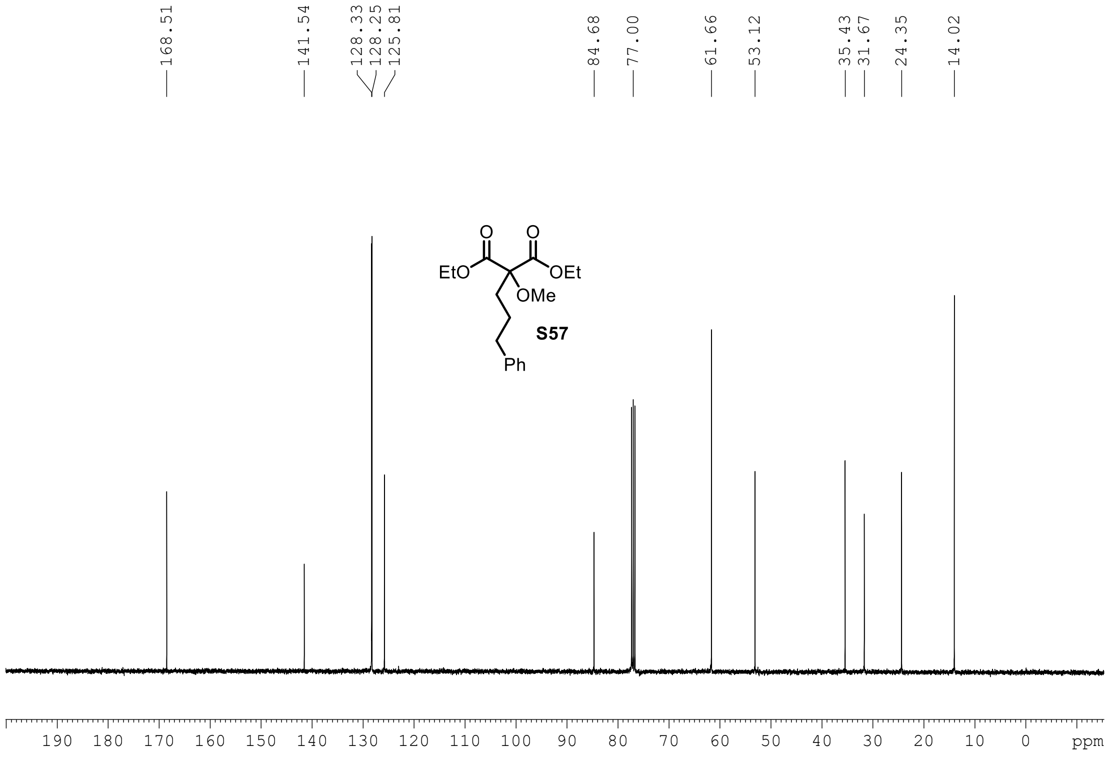

Supplementary Fig. 107. ^{1}H NMR of compound S58 ($CDCl_3$, 400 MHz, 25 °C)

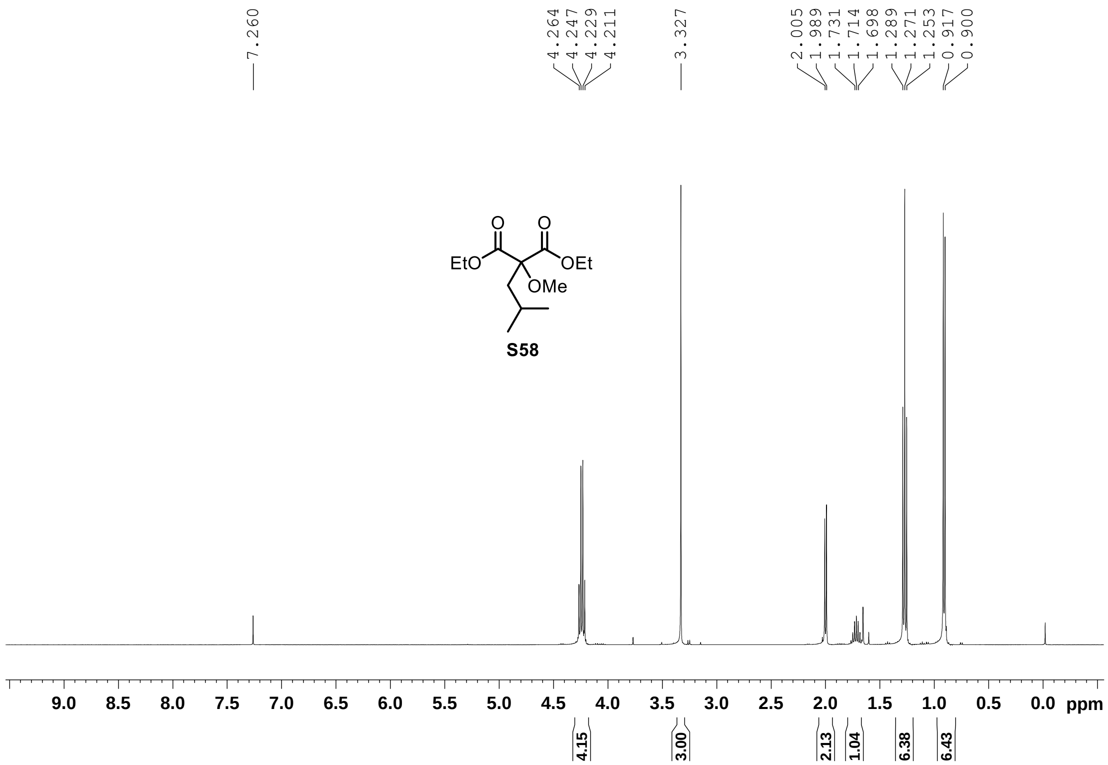

Supplementary Fig. 108. ^{13}C NMR of compound S58 ($CDCl_3$, 100 MHz, 25 °C)

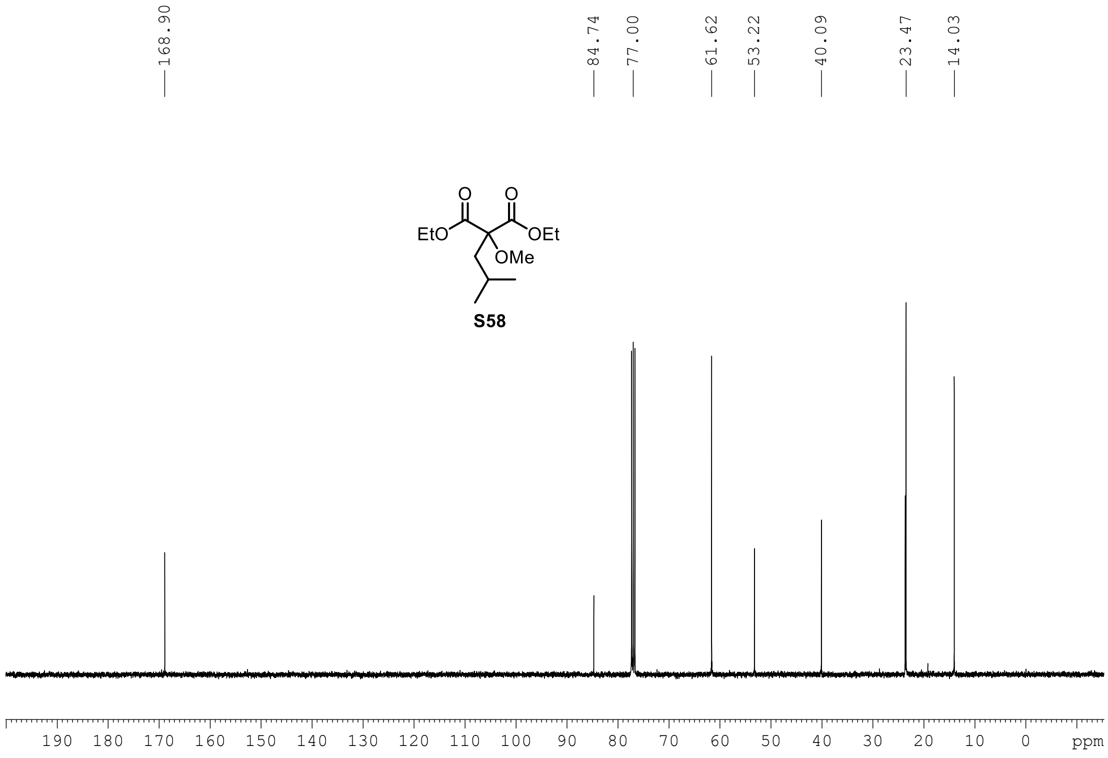

Supplementary Fig. 109. ¹H NMR of compound S59 ($CDCl_3$, 400 MHz, 25 °C)

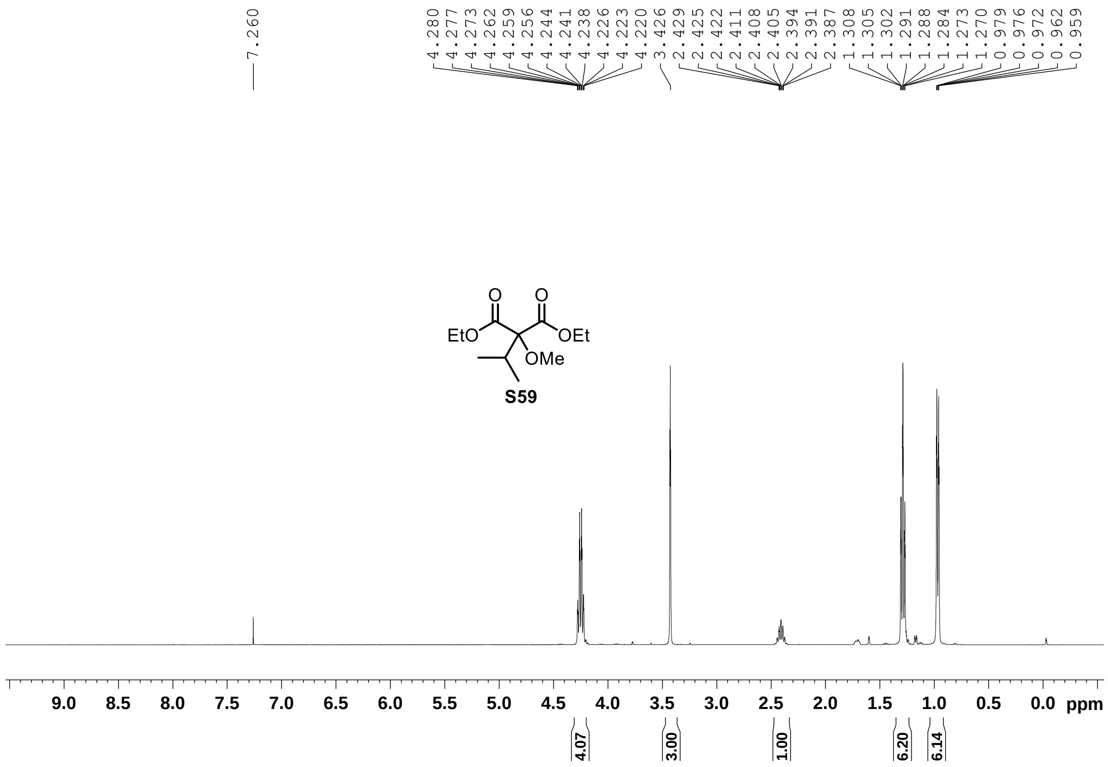

Supplementary Fig. 110. ¹³C NMR of compound S59 ($CDCl_3$, 100 MHz, 25 °C)

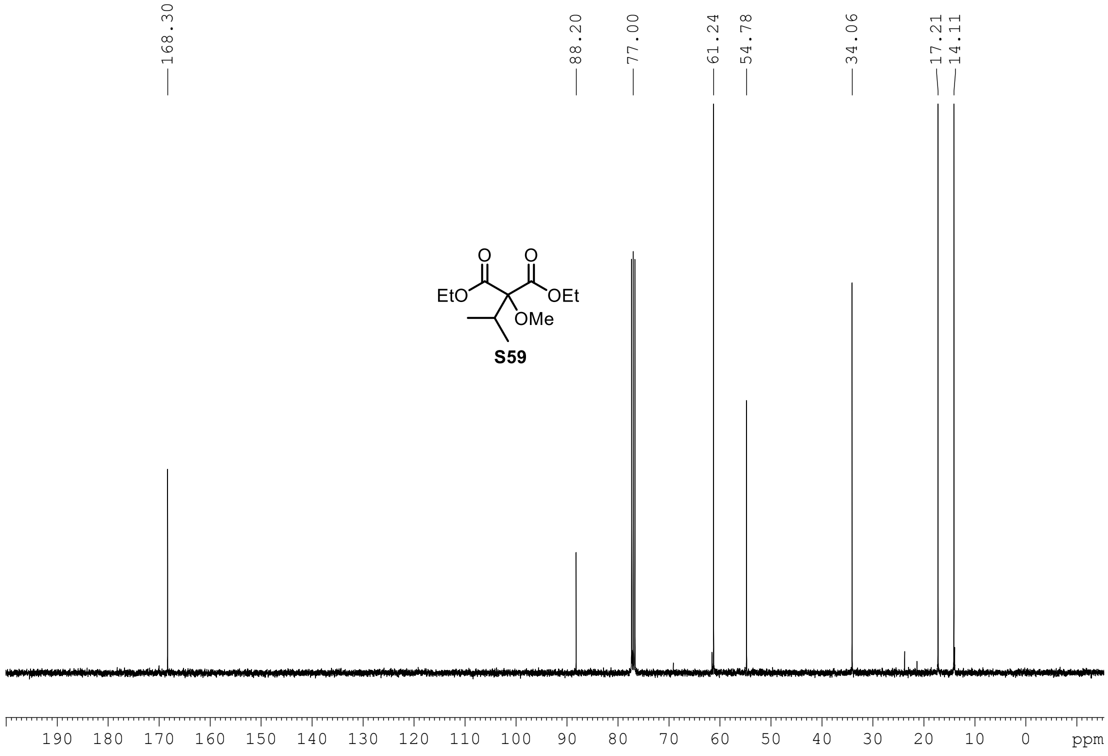

Supplementary Fig. 111. ^{1}H **NMR of compound S60 ($CDCl_3$, 400 MHz, 25 °C)**

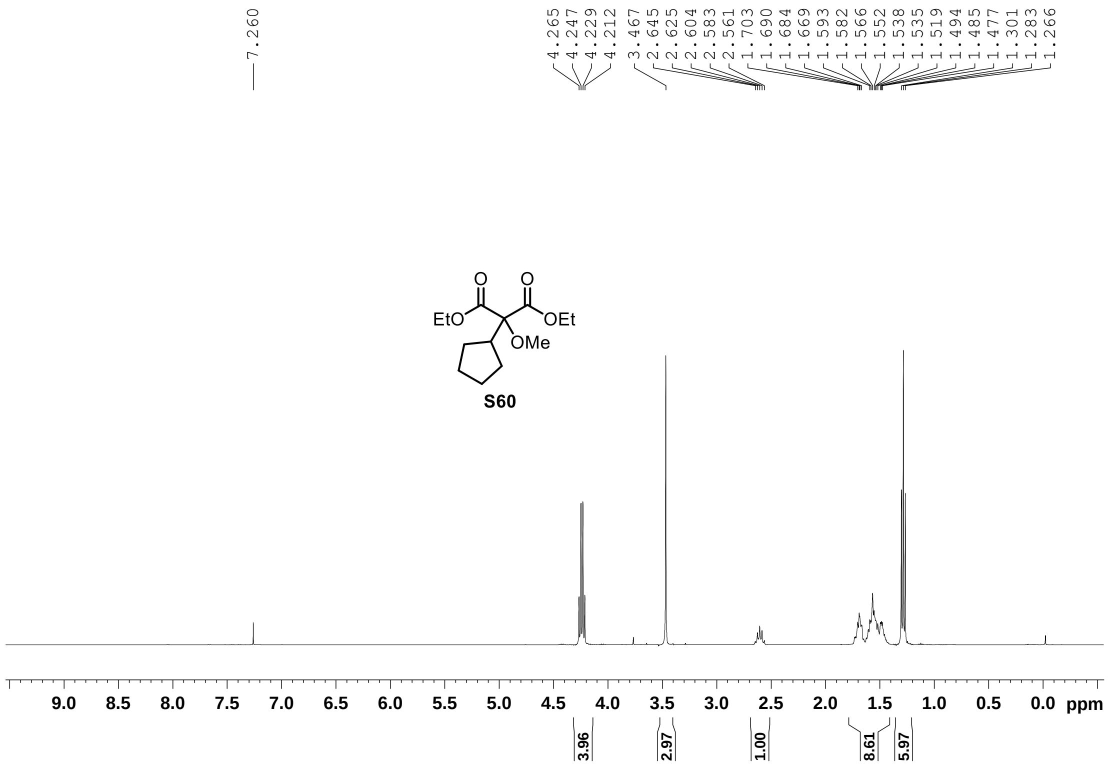

Supplementary Fig. 112. ^{13}C **NMR of compound S60 ($CDCl_3$, 100 MHz, 25 °C)**

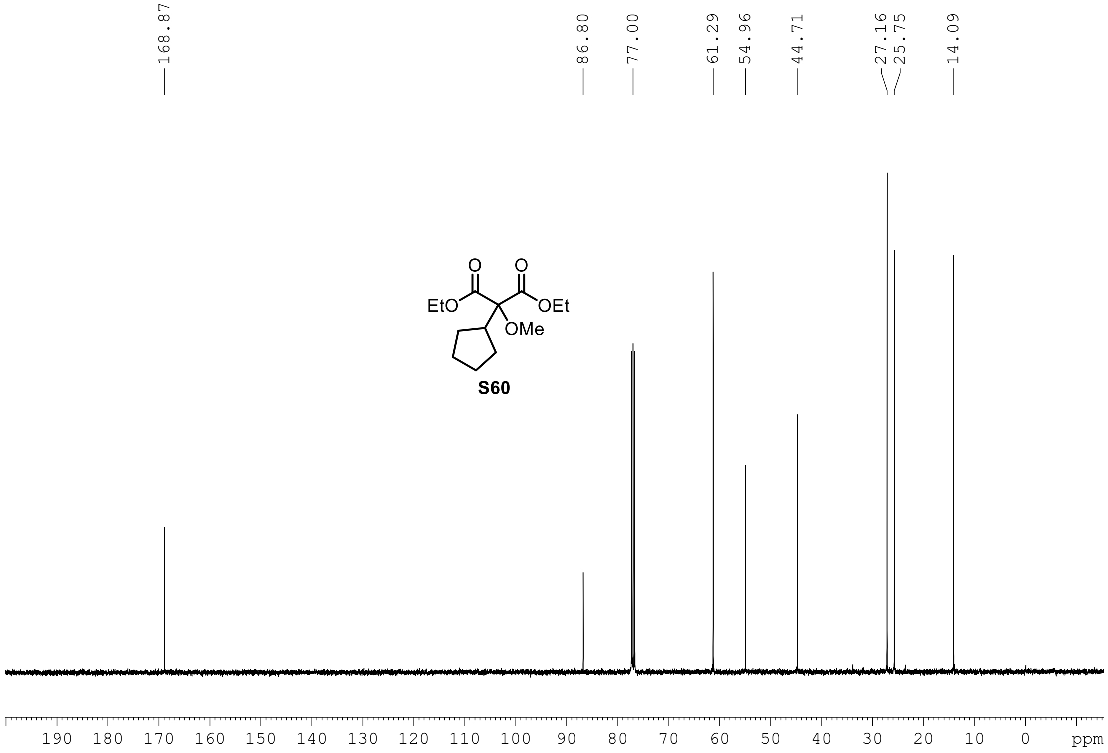

Supplementary Fig. 113. ^{1}H NMR of compound S61 ($CDCl_3$, 400 MHz, 25 °C)

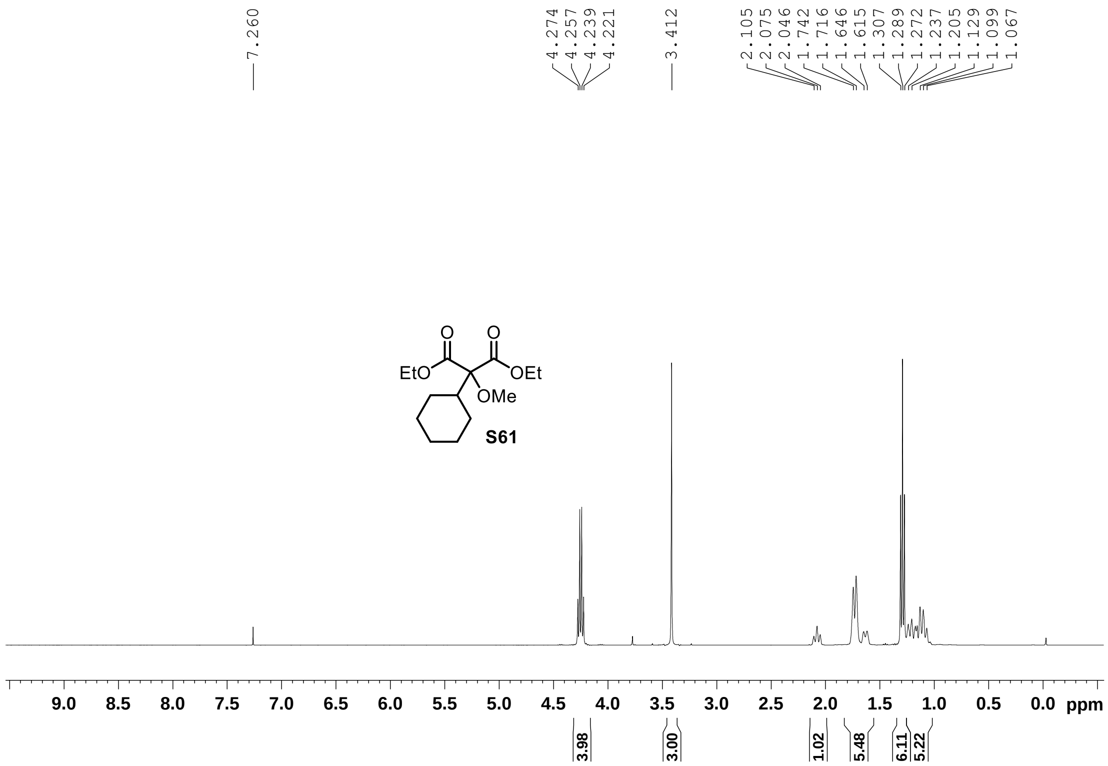

Supplementary Fig. 114. ^{13}C NMR of compound S61 ($CDCl_3$, 100 MHz, 25 °C)

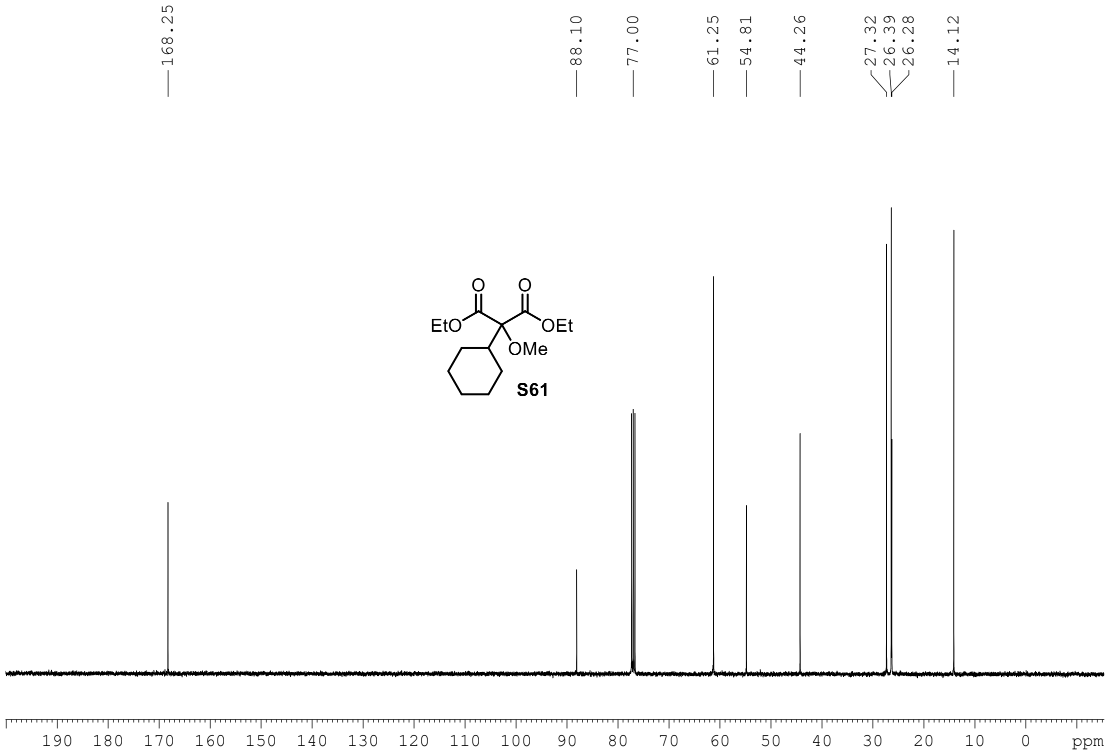

Supplementary Fig. 115. ^{1}H NMR of compound S62 ($CDCl_3$, 400 MHz, 25 °C)

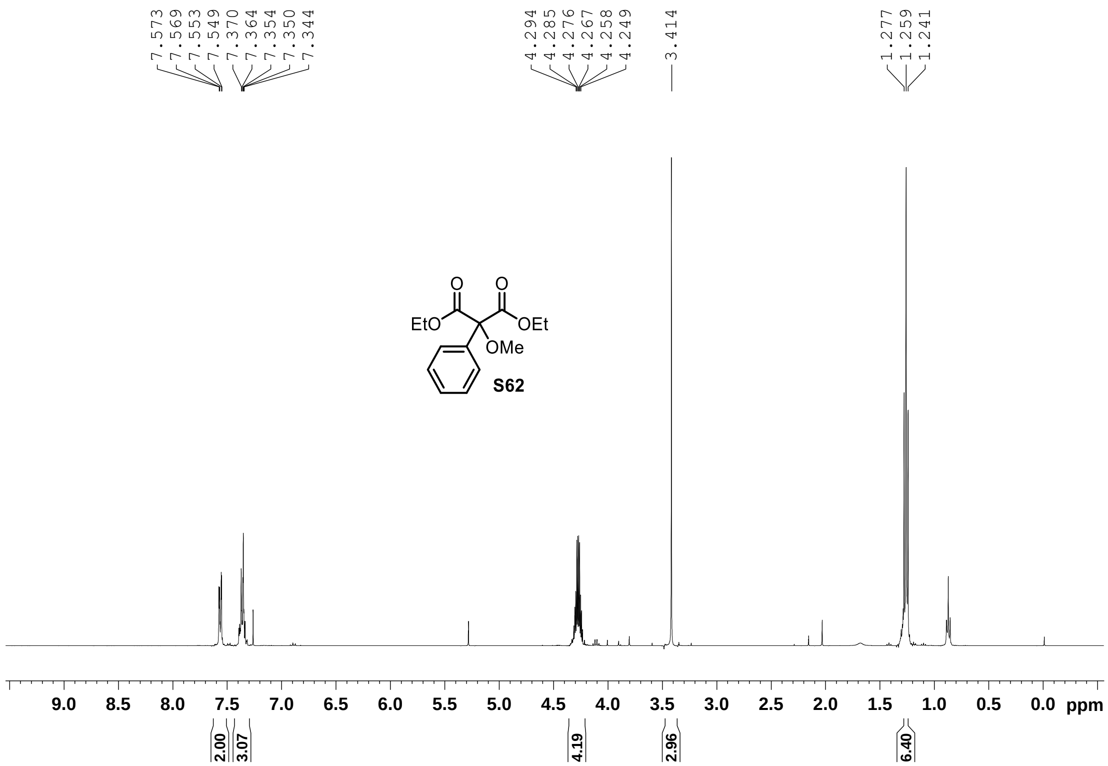

Supplementary Fig. 116. ^{13}C NMR of compound S62 ($CDCl_3$, 100 MHz, 25 °C)

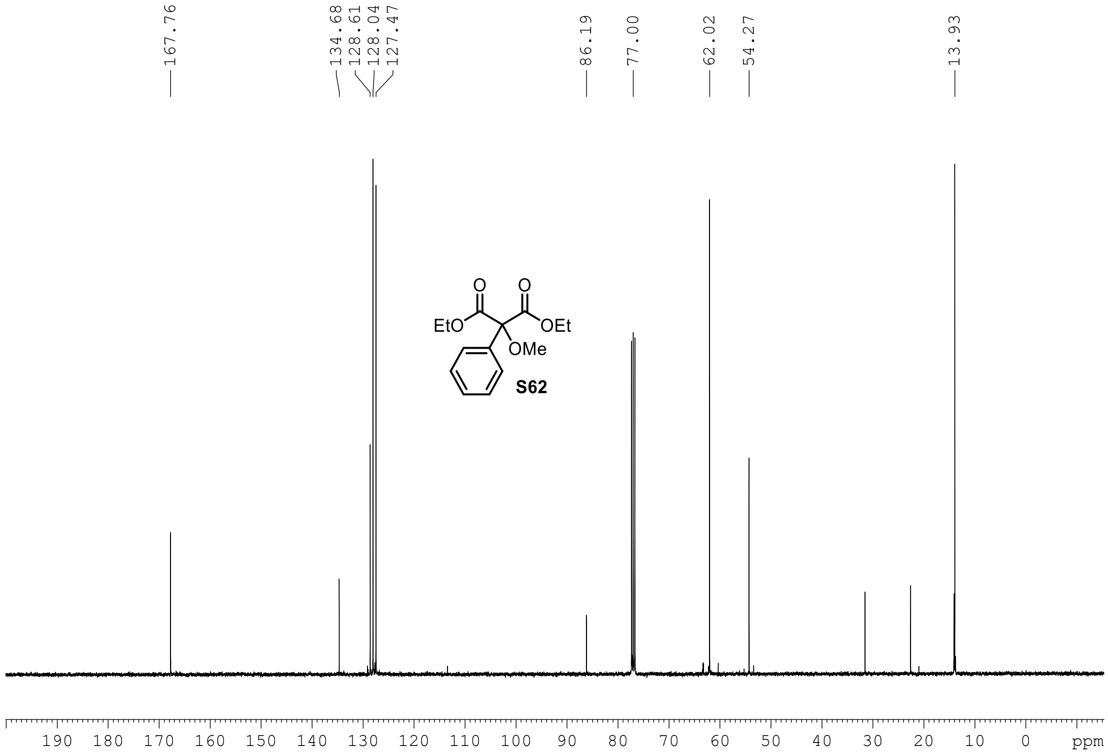

Supplementary Fig. 117. ^{1}H NMR of compound S63 ($CDCl_3$, 400 MHz, 25 °C)

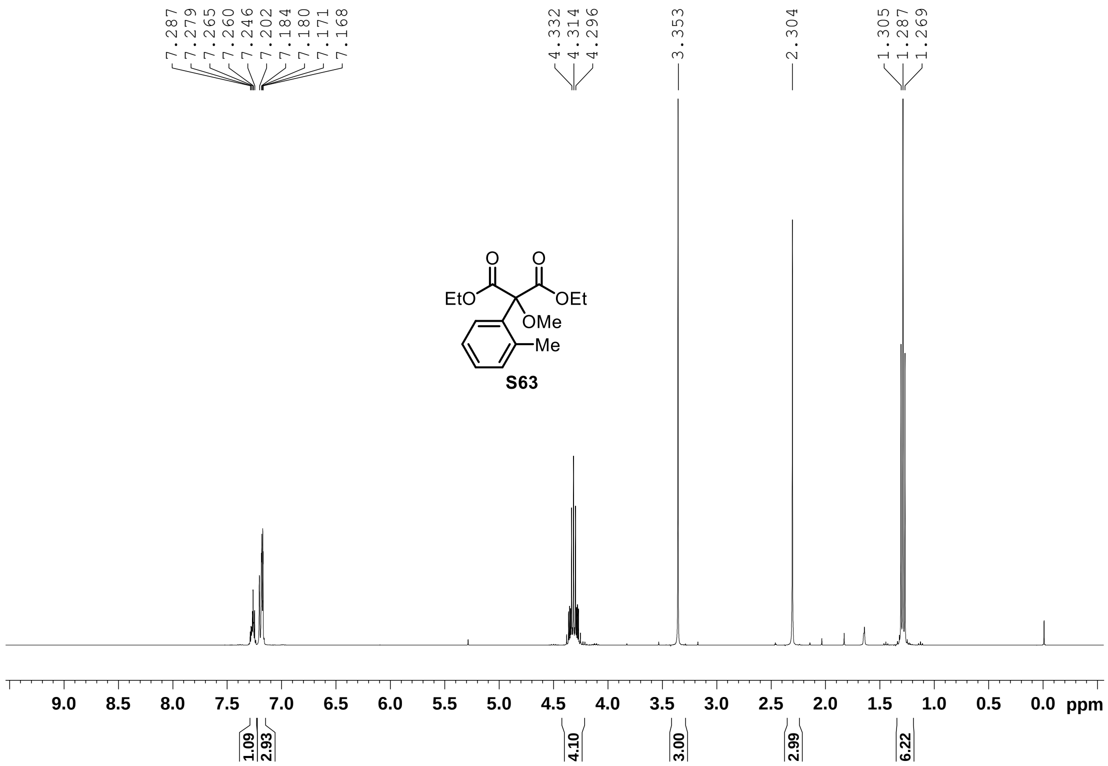

Supplementary Fig. 118. ^{13}C NMR of compound S63 ($CDCl_3$, 100 MHz, 25 °C)

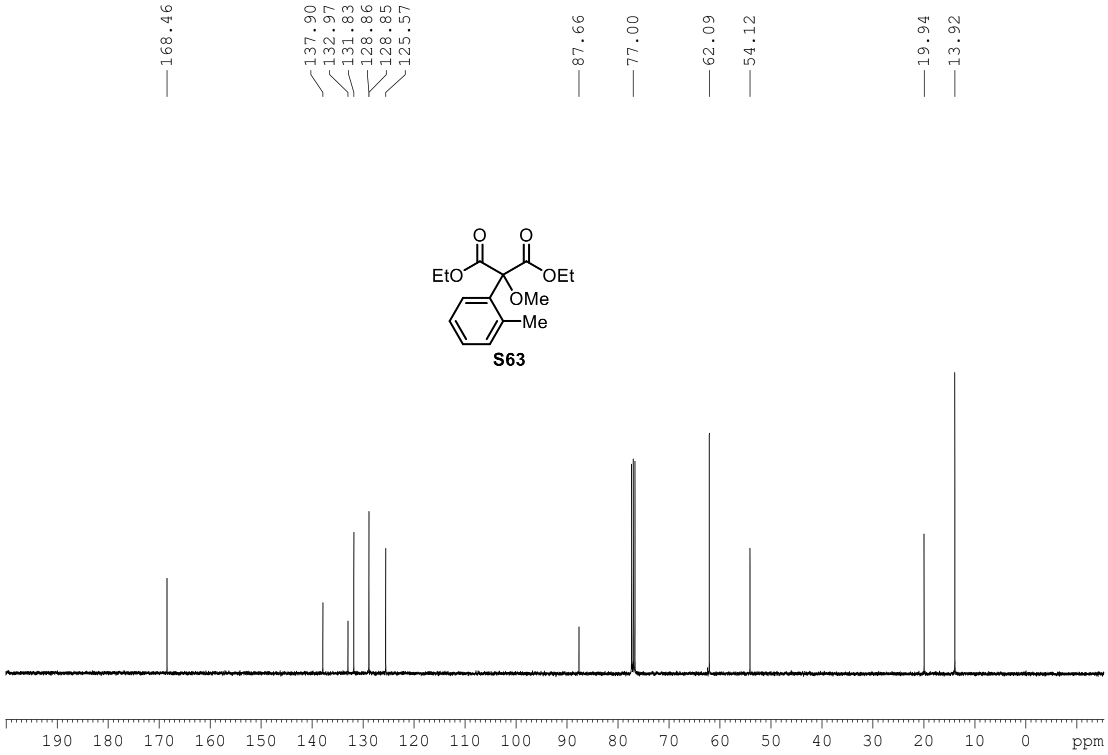

Supplementary Fig. 119. ^{1}H NMR of compound S64 ($CDCl_3$, 400 MHz, 25 °C)

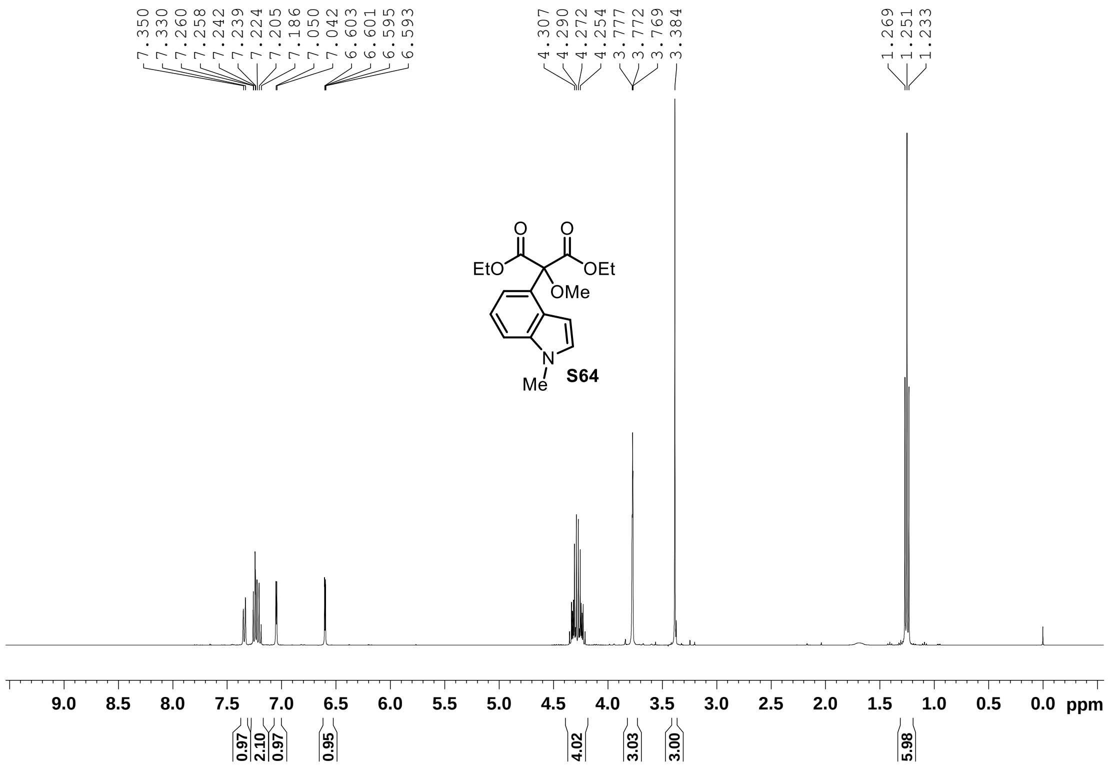

Supplementary Fig. 120. ^{13}C NMR of compound S64 ($CDCl_3$, 100 MHz, 25 °C)

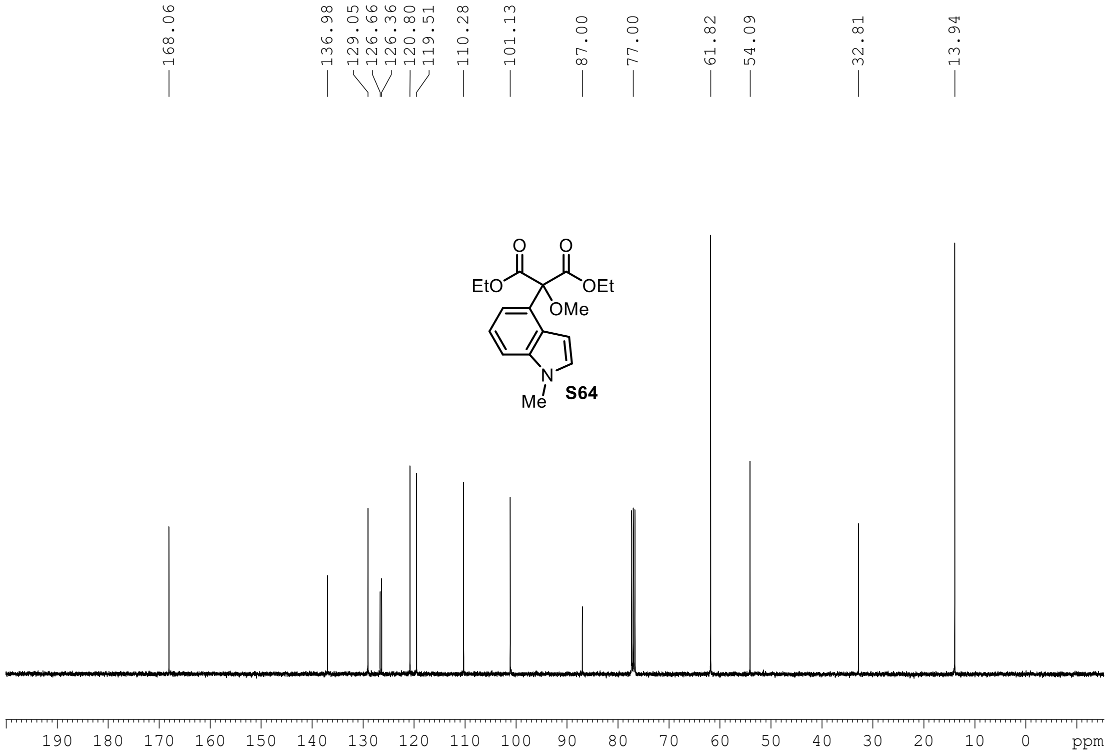

Supplementary Fig. 121. ^{1}H NMR of compound S66 ($CDCl_3$, 400 MHz, 25 °C)

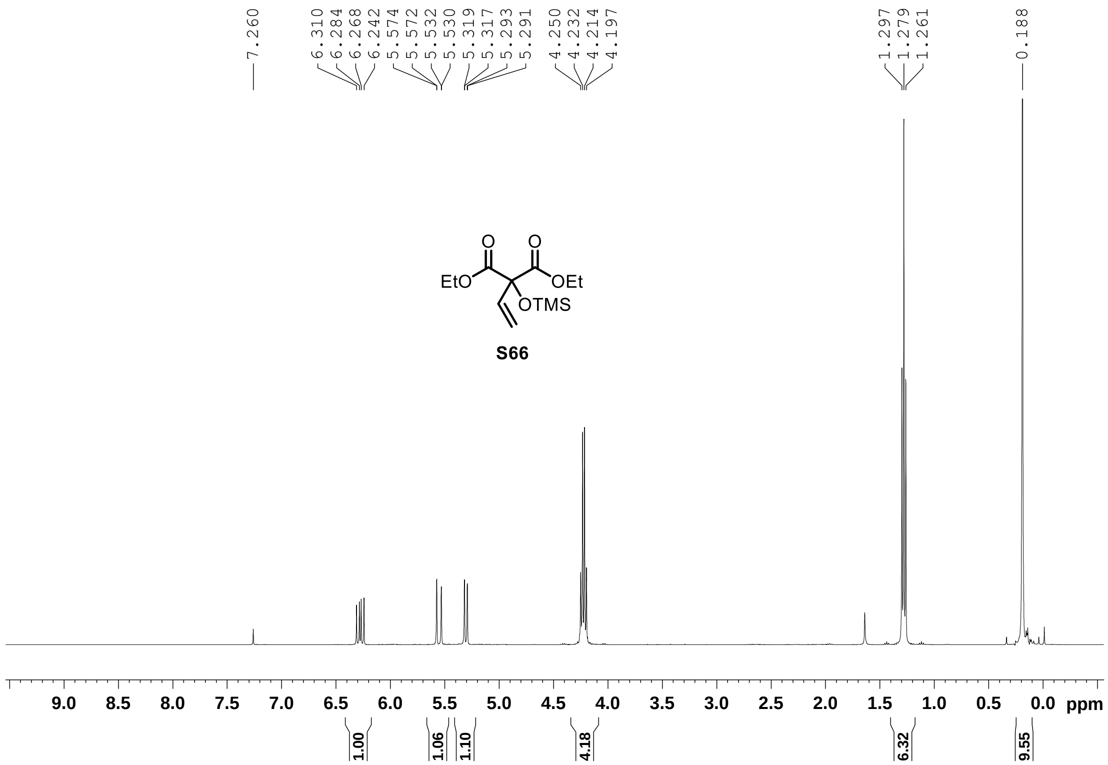

Supplementary Fig. 122. ^{13}C NMR of compound S66 ($CDCl_3$, 100 MHz, 25 °C)

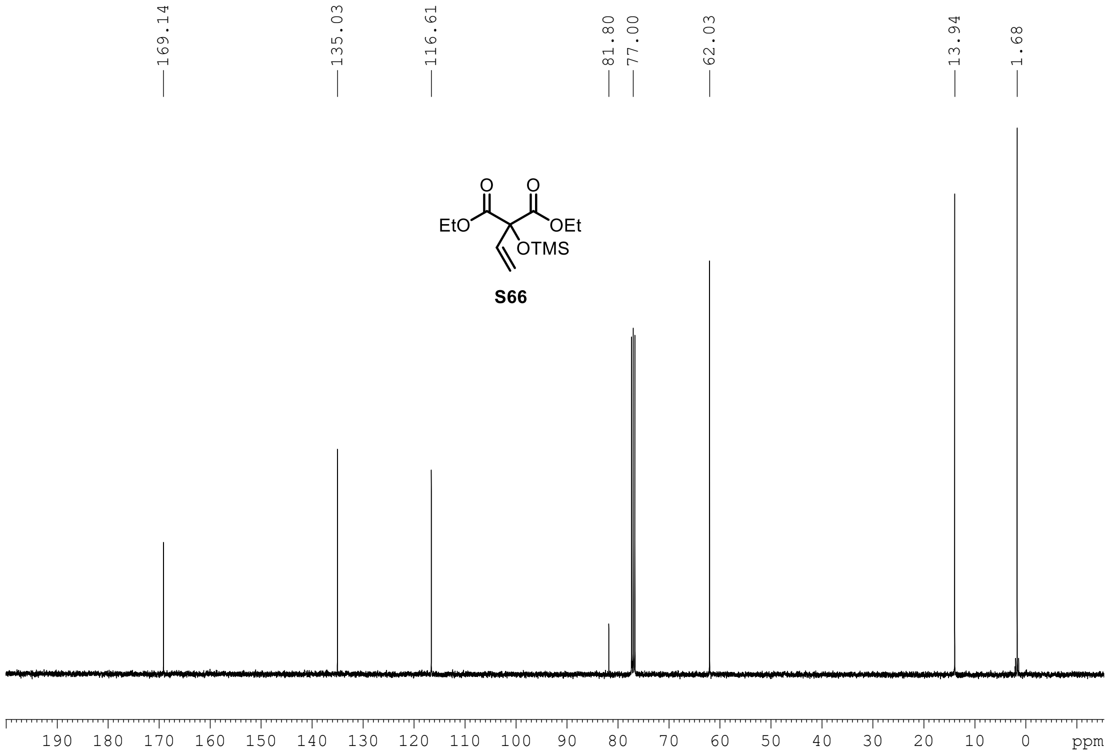

Supplementary Fig. 123. ^{1}H NMR of compound S14 ($CDCl_3$, 400 MHz, 25 °C)

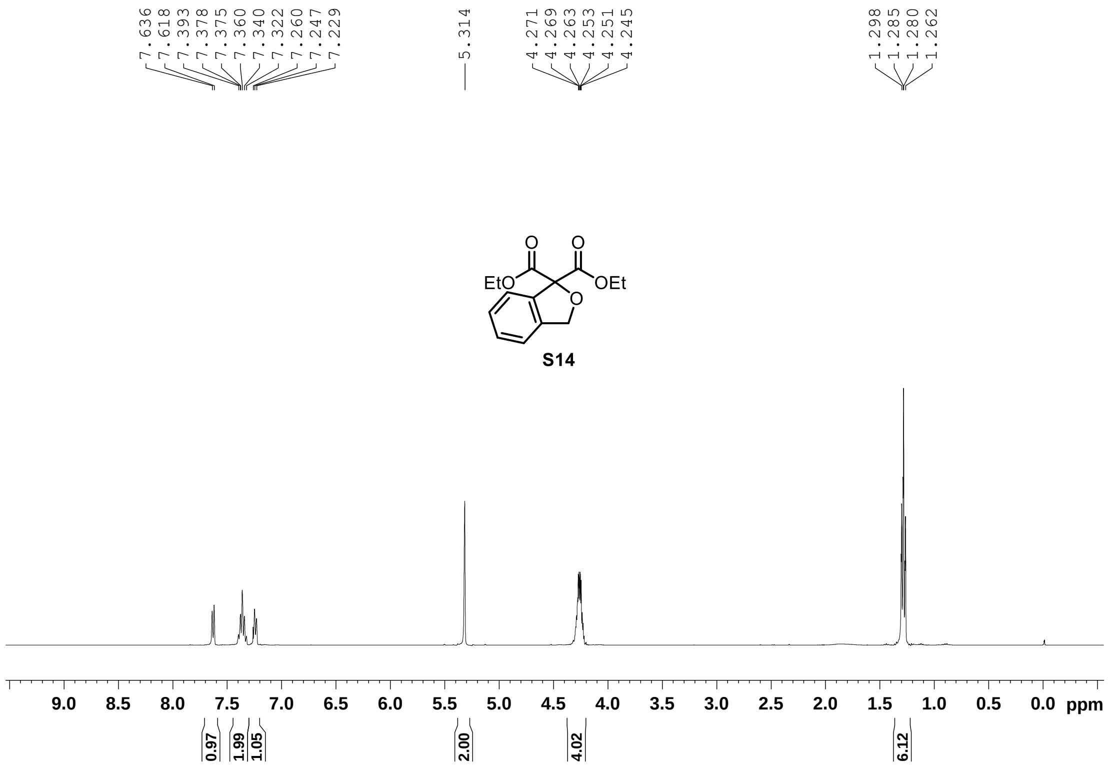

Supplementary Fig. 124. ^{13}C NMR of compound S14 ($CDCl_3$, 100 MHz, 25 °C)

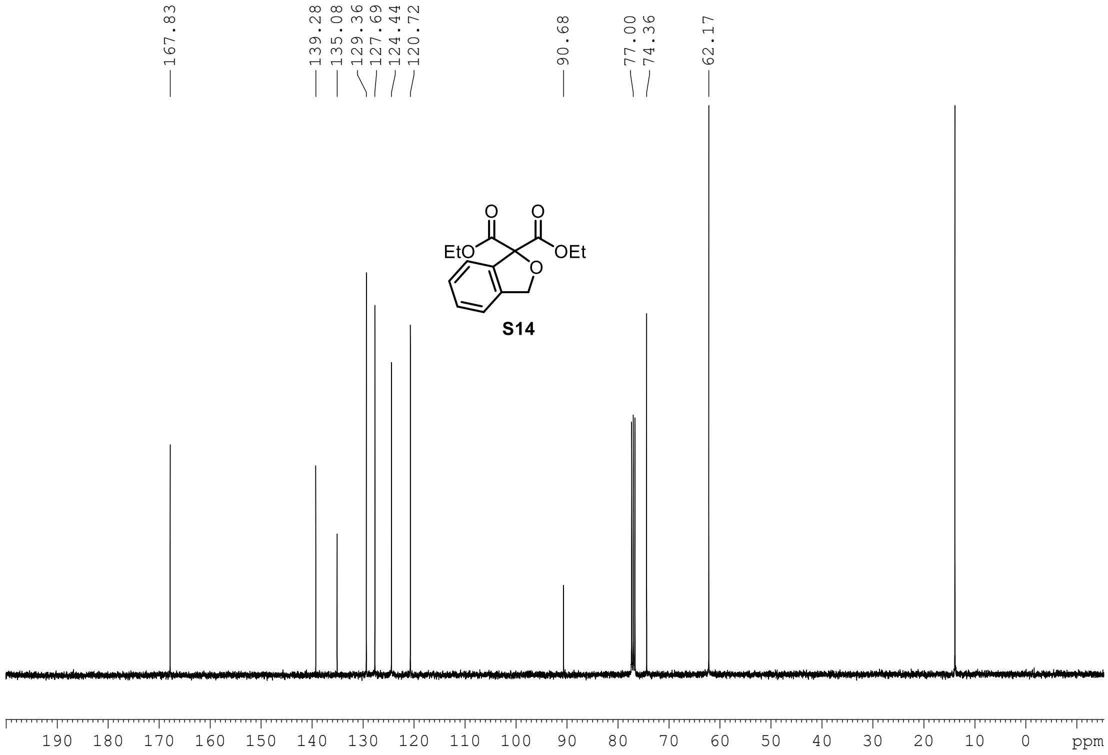

Supplementary Fig. 125. ^{1}H NMR of compound S67 ($CDCl_3$, 400 MHz, 25 °C)

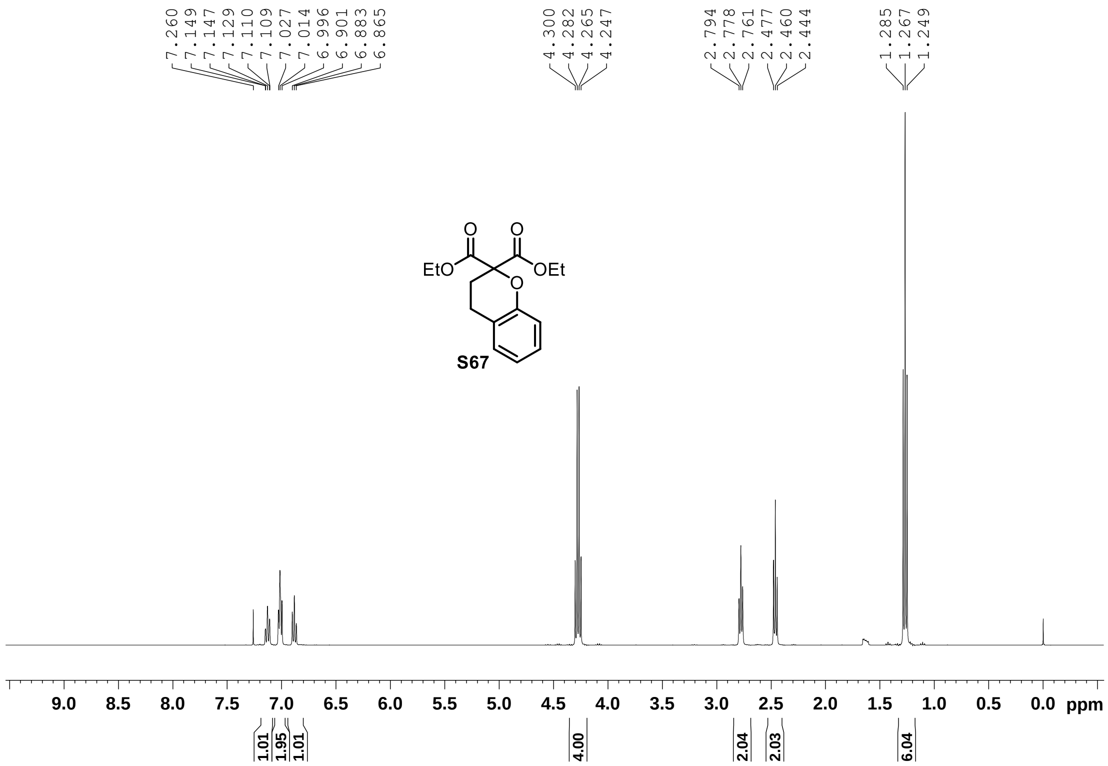

Supplementary Fig. 126. ^{13}C NMR of compound S67 ($CDCl_3$, 100 MHz, 25 °C)

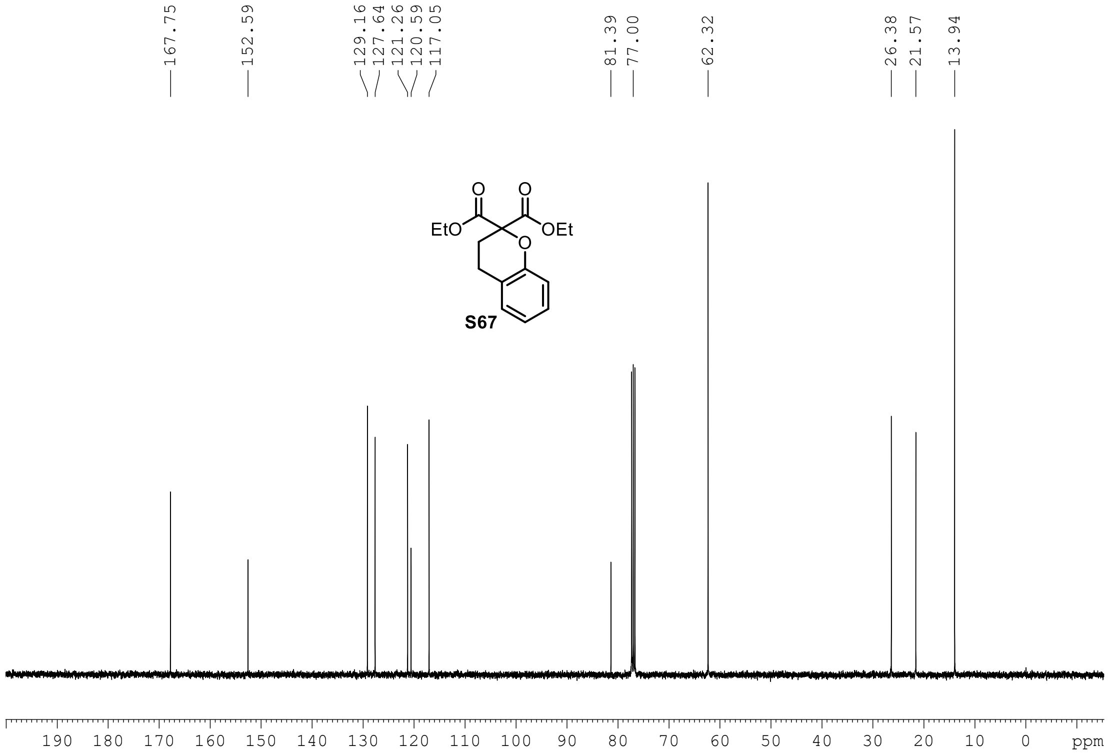

Supplementary Fig. 127. ^{1}H NMR of compound S68 ($CDCl_3$, 400 MHz, 25 °C)

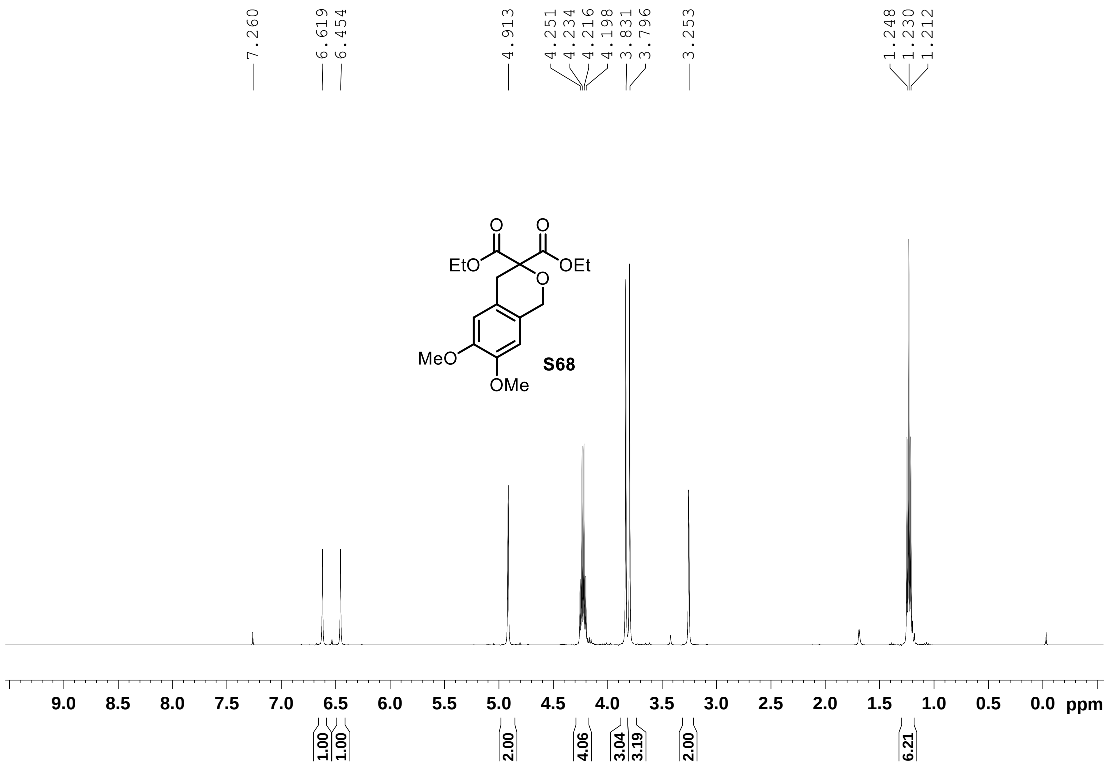

Supplementary Fig. 128. ^{13}C NMR of compound S68 ($CDCl_3$, 100 MHz, 25 °C)

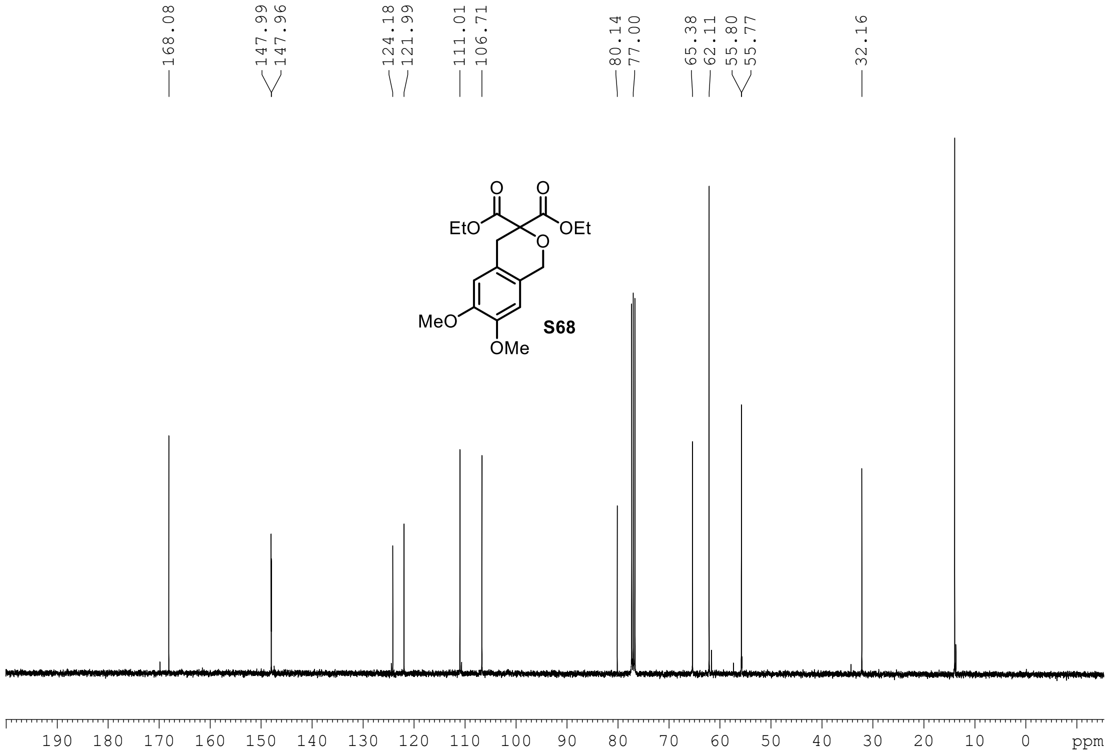

Supplementary Fig. 129. ^{1}H NMR of compound 71 ($CDCl_3$, 500 MHz, 25 °C)

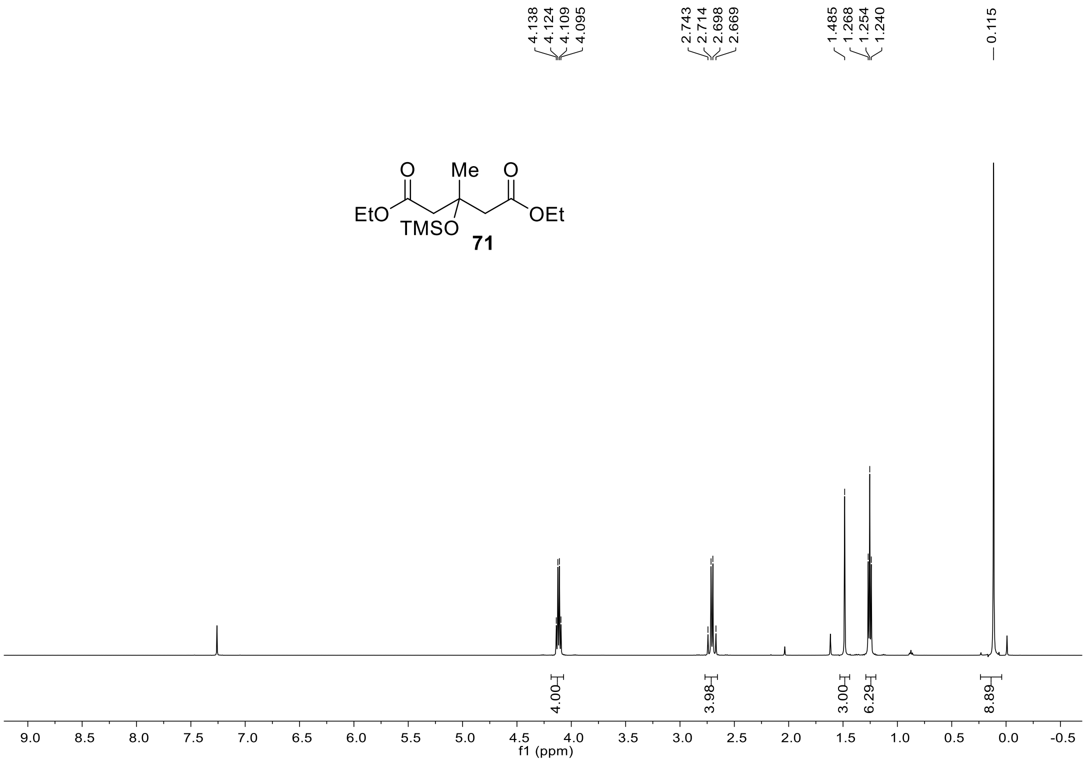

Supplementary Fig. 130. ^{13}C NMR of compound 71 ($CDCl_3$, 125 MHz, 25 °C)

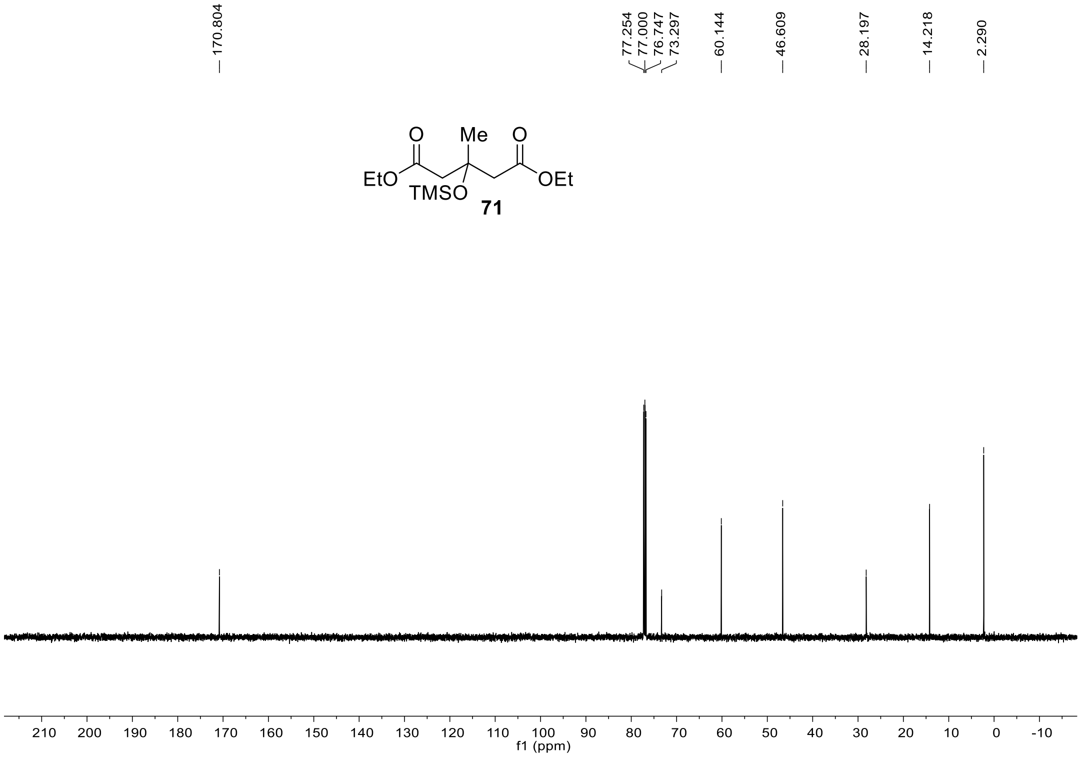

Supplementary Fig. 131. ^{1}H NMR of compound (–)-9 ($CDCl_3$, 400 MHz, 25 °C)

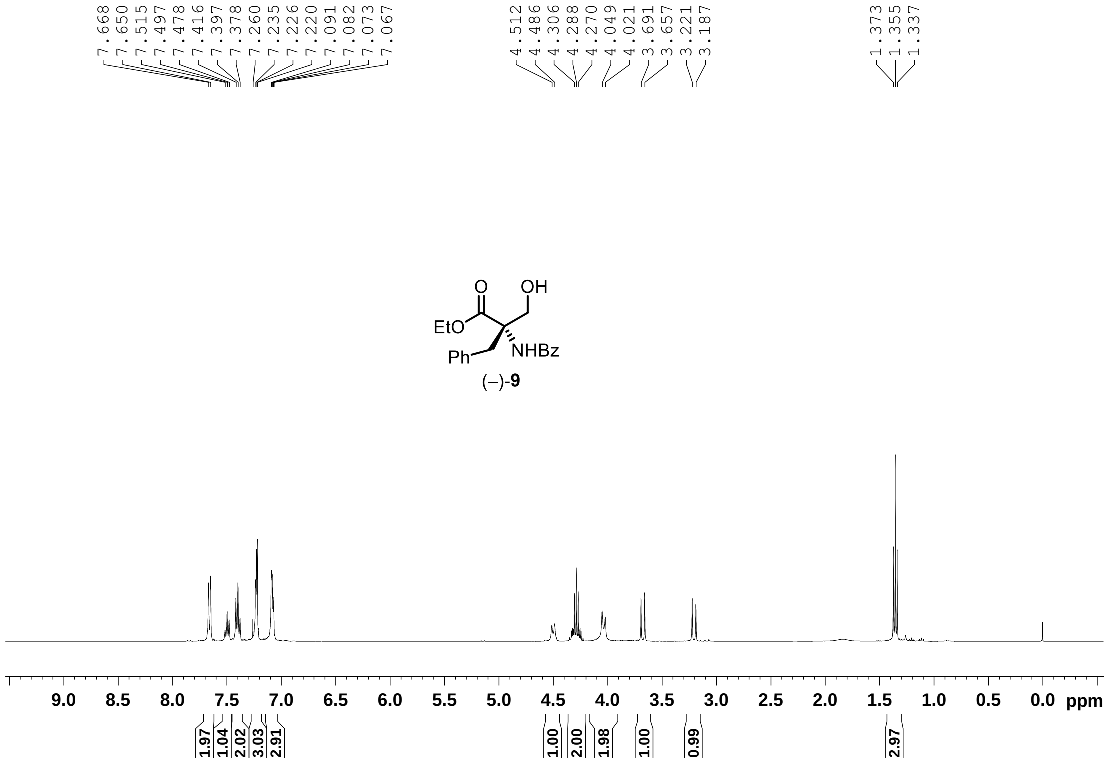

Supplementary Fig. 132. ^{13}C NMR of compound (–)-9 ($CDCl_3$, 100 MHz, 25 °C)

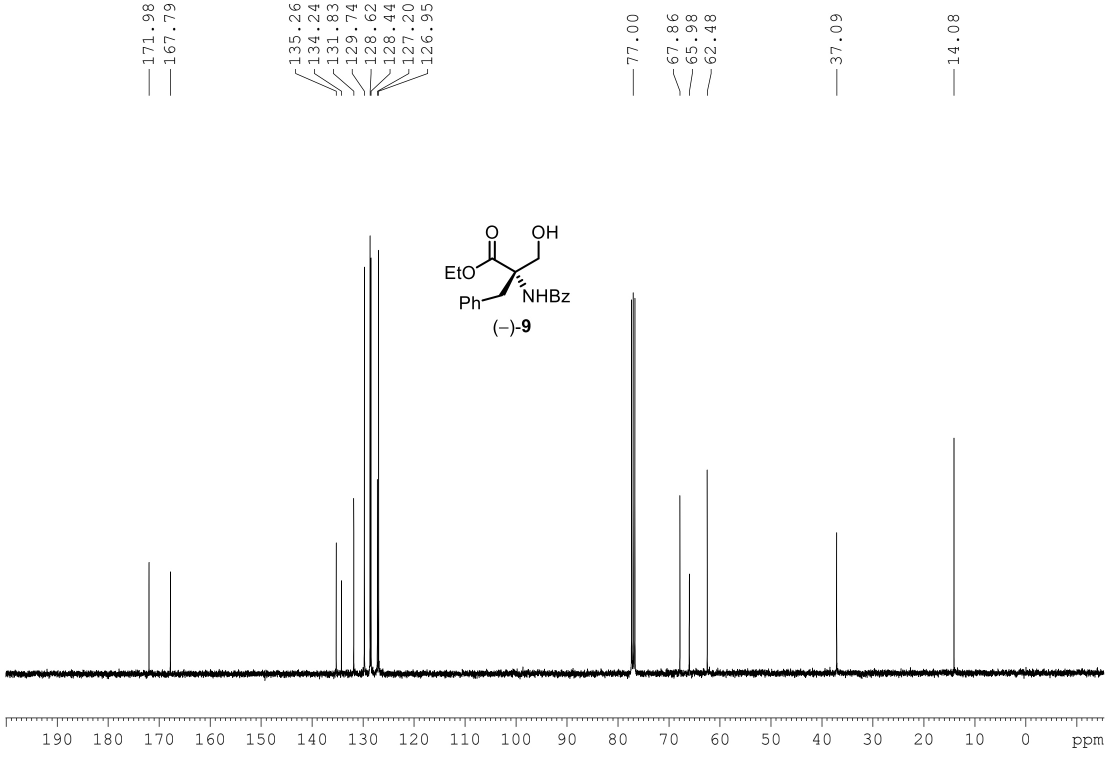

Supplementary Fig. 133. ¹H NMR of compound (–)-15 (CDCl₃, 400 MHz, 25 °C)

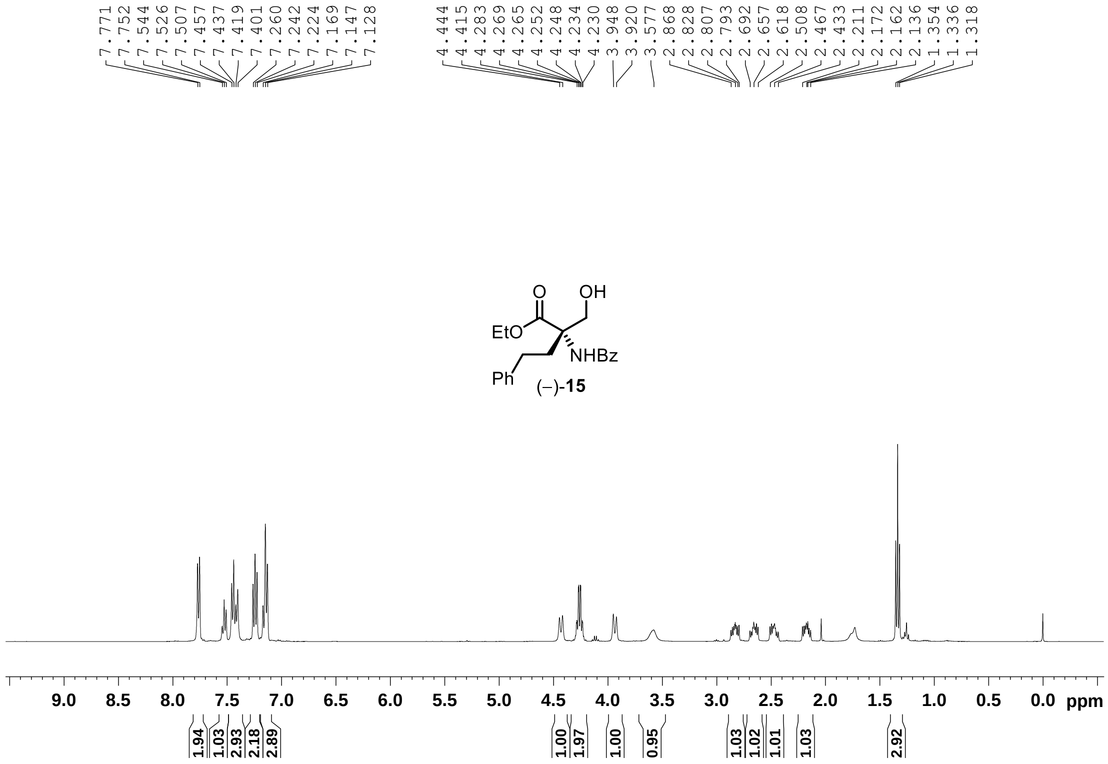

Supplementary Fig. 134. ¹³C NMR of compound (–)-15 (CDCl₃, 100 MHz, 25 °C)

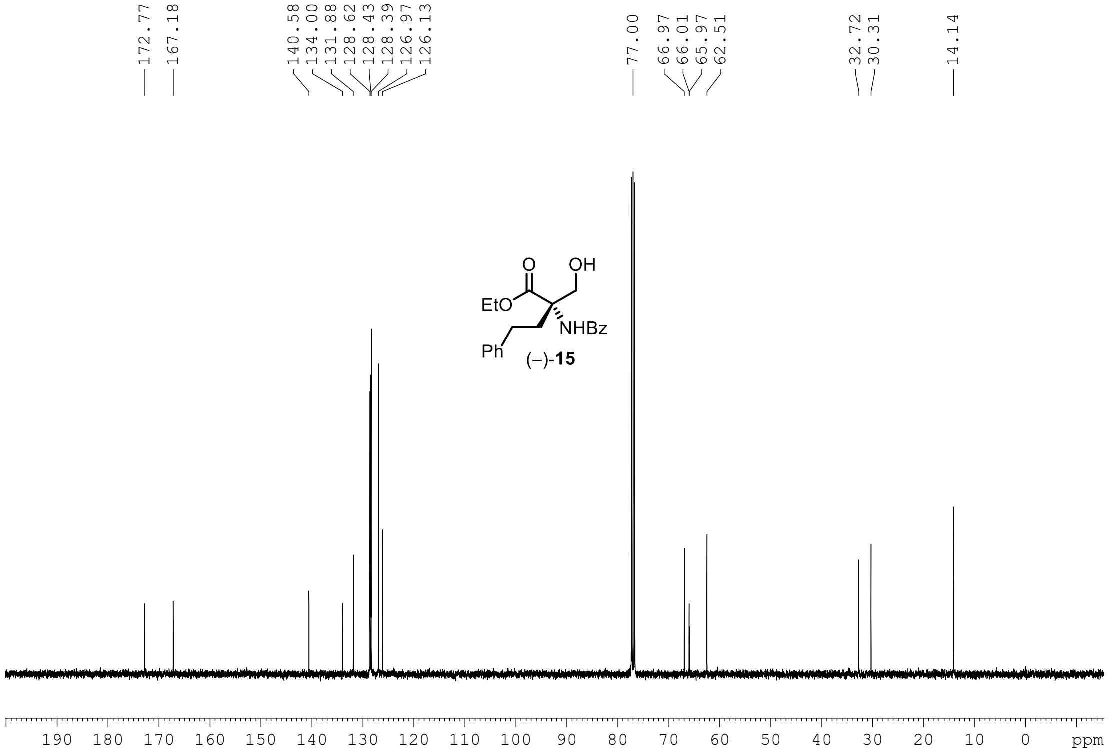

Supplementary Fig. 135. ¹H NMR of compound (–)-16 ($CDCl_3$, 400 MHz, 25 °C)

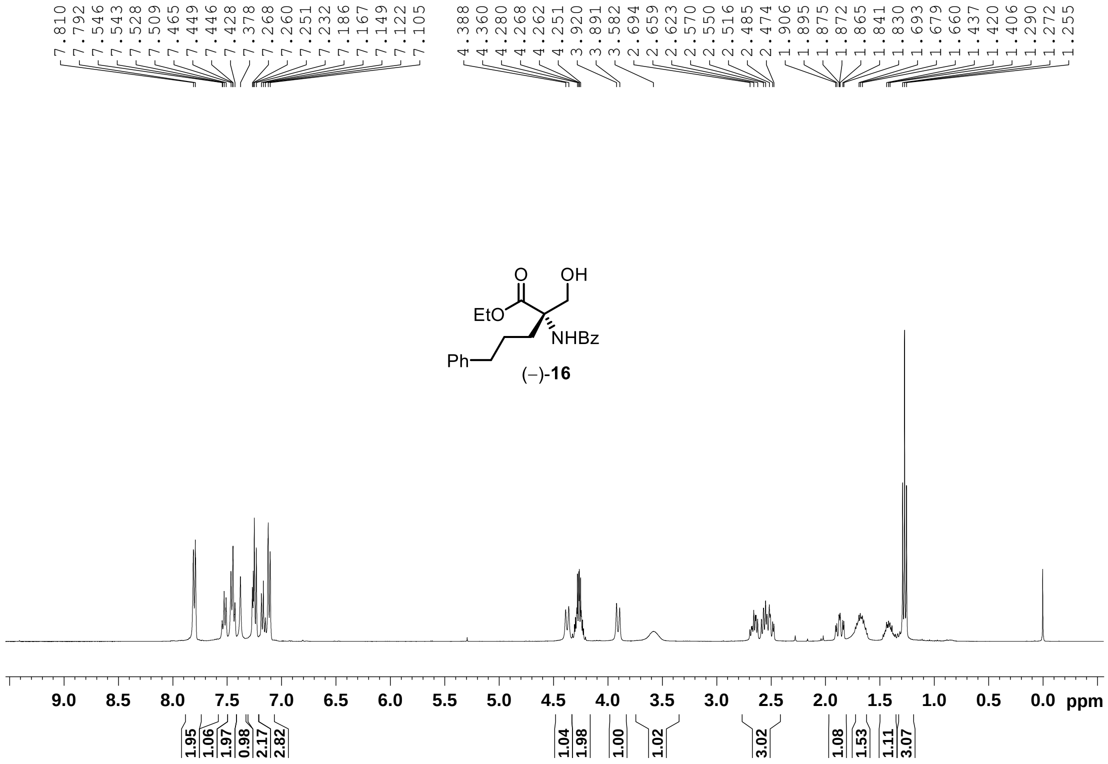

Supplementary Fig. 136. ¹³C NMR of compound (–)-16 ($CDCl_3$, 100 MHz, 25 °C)

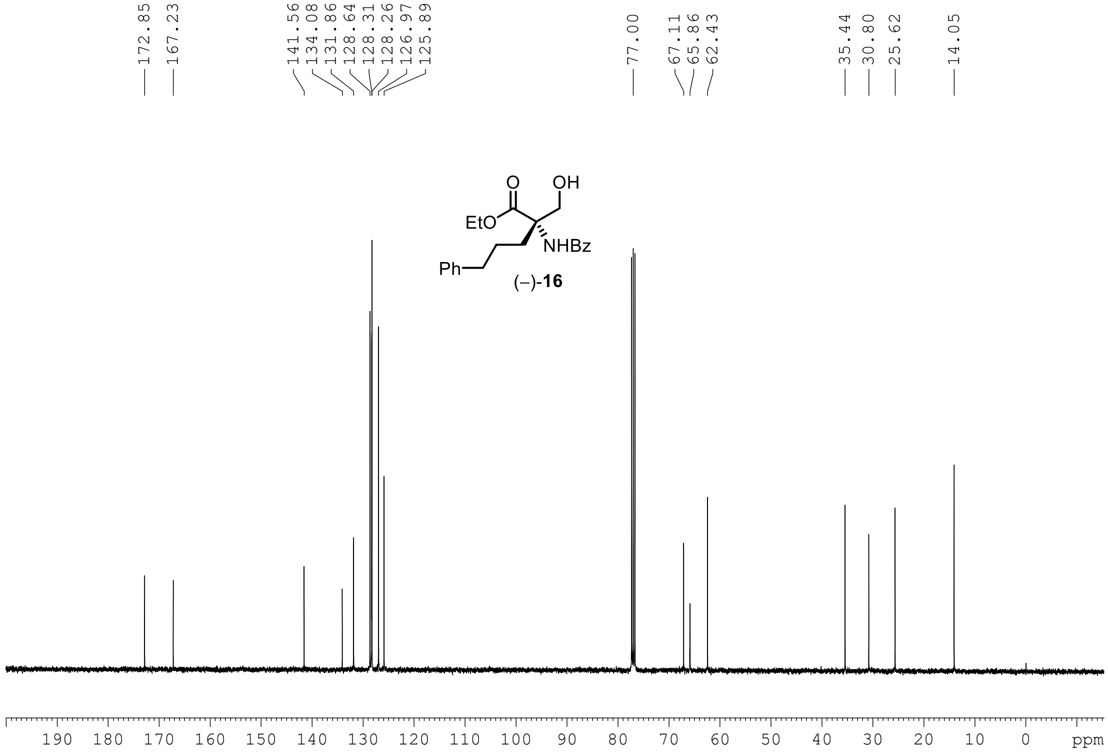

Supplementary Fig. 137. ^{1}H NMR of compound (–)-17 (CDCl$_3$, 400 MHz, 25 °C)

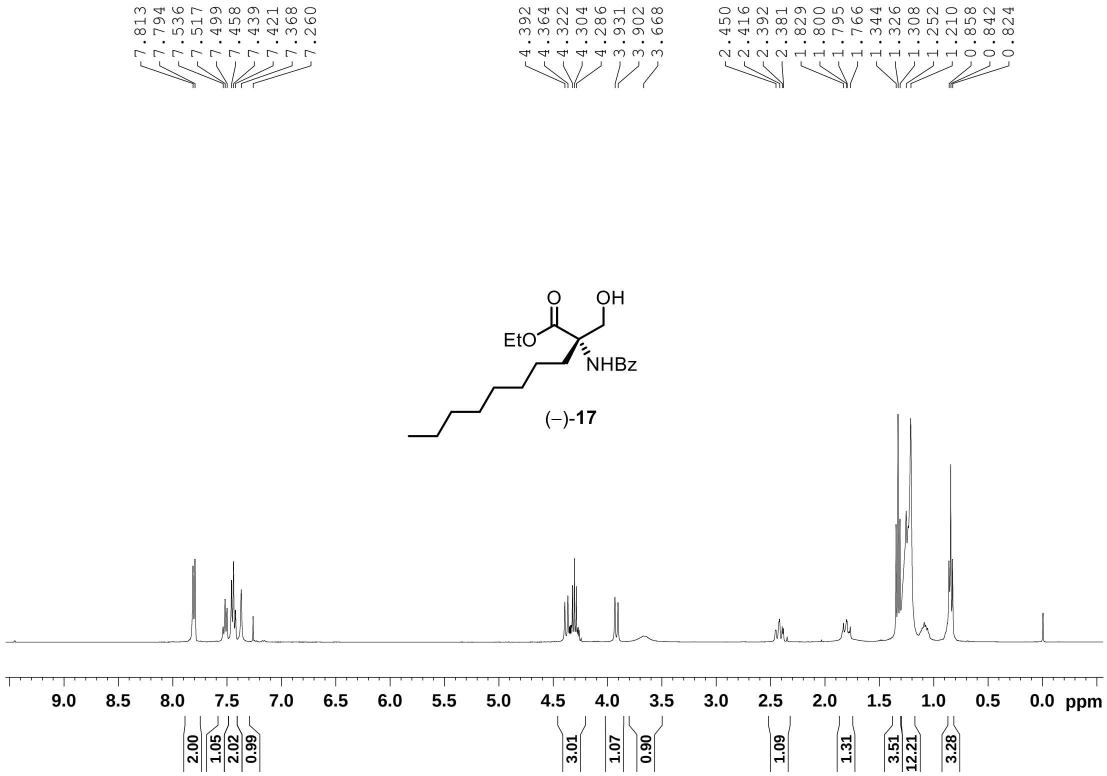

Supplementary Fig. 138. ^{13}C NMR of compound (–)-17 (CDCl$_3$, 100 MHz, 25 °C)

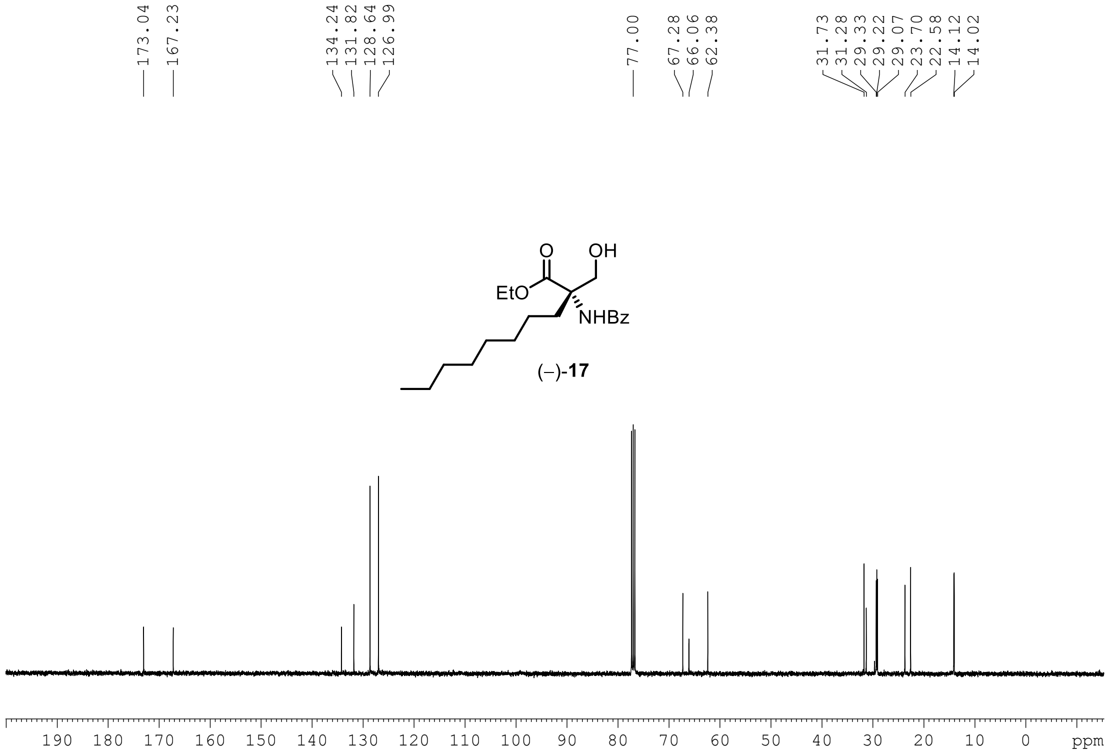

Supplementary Fig. 139. ^{1}H NMR of compound (–)-18 (CDCl₃, 400 MHz, 25 °C)

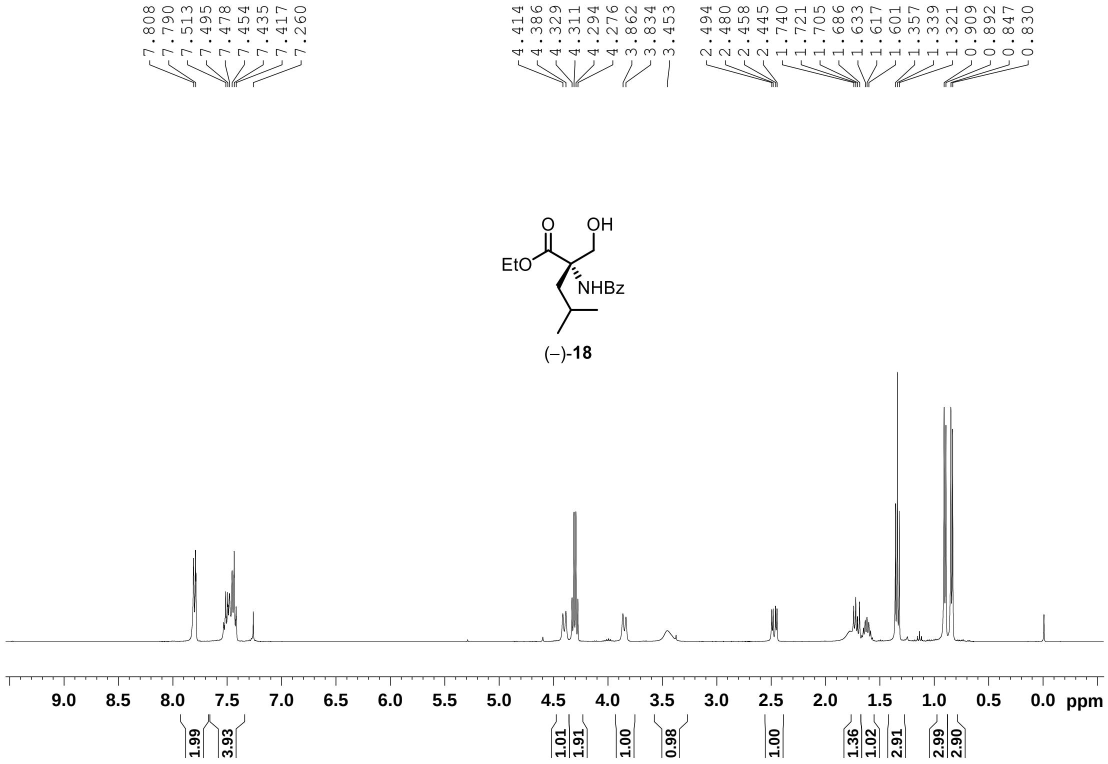

Supplementary Fig. 140. ^{13}C NMR of compound (–)-18 (CDCl₃, 100 MHz, 25 °C)

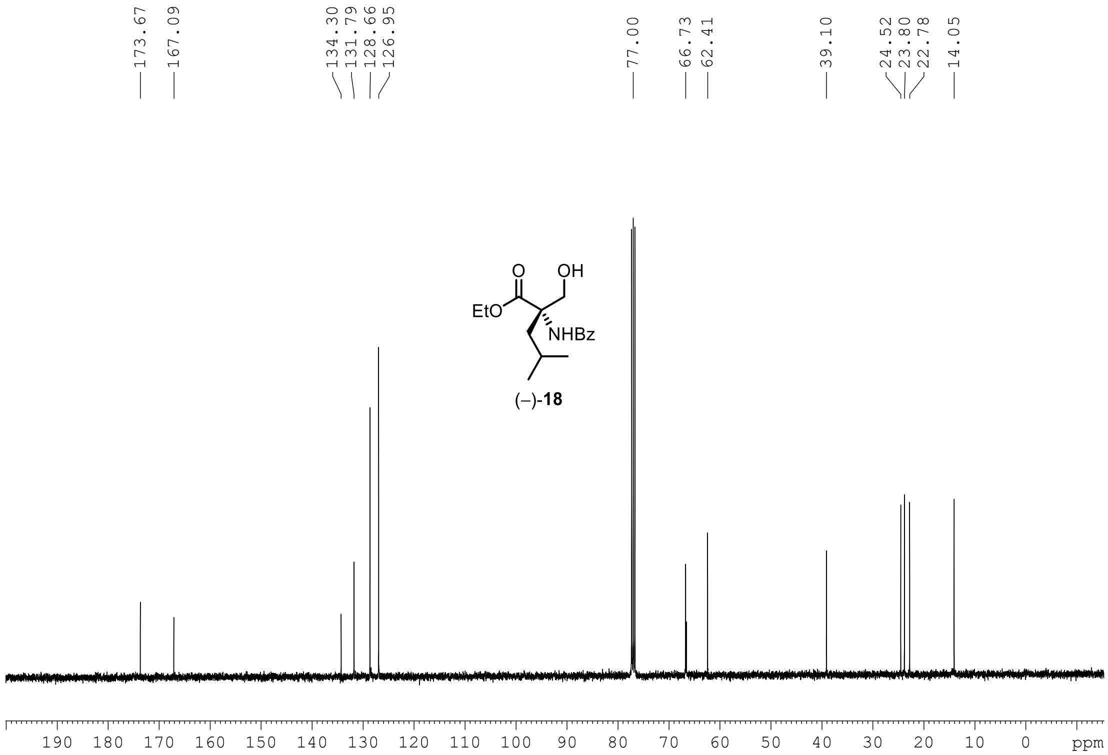

Supplementary Fig. 141. ^{1}H NMR of compound (–)-19 (CDCl$_3$, 400 MHz, 25 °C)

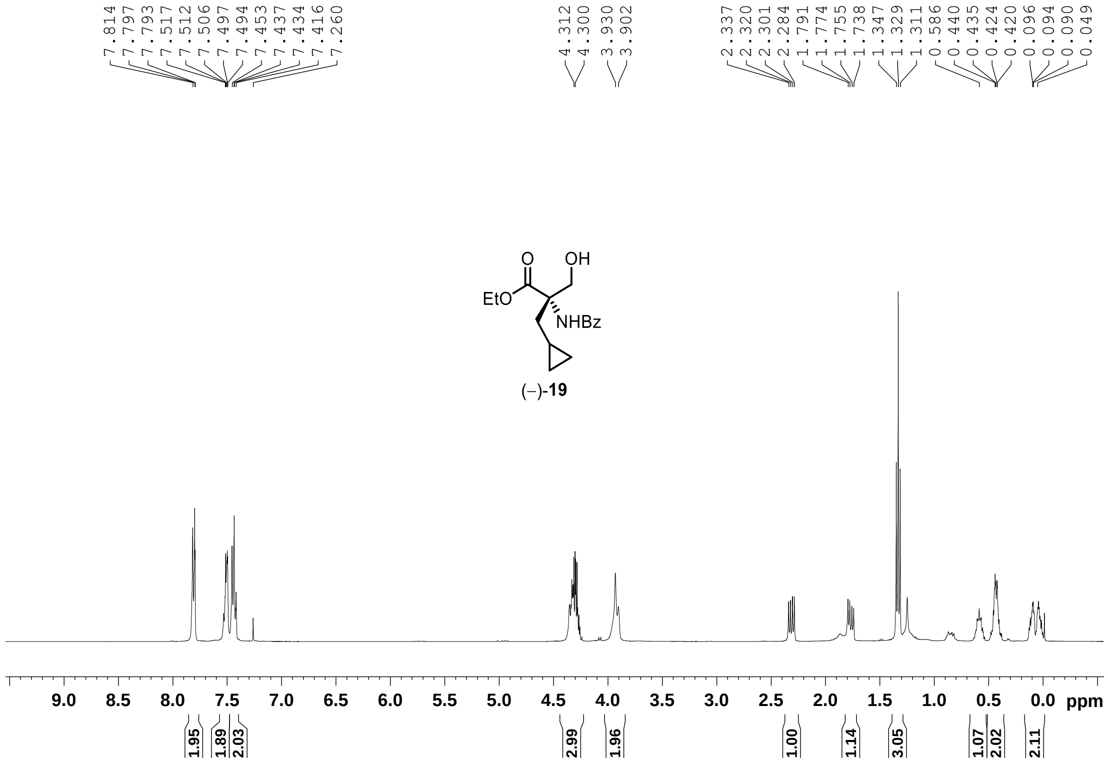

Supplementary Fig. 142. ^{13}C NMR of compound (–)-19 (CDCl$_3$, 100 MHz, 25 °C)

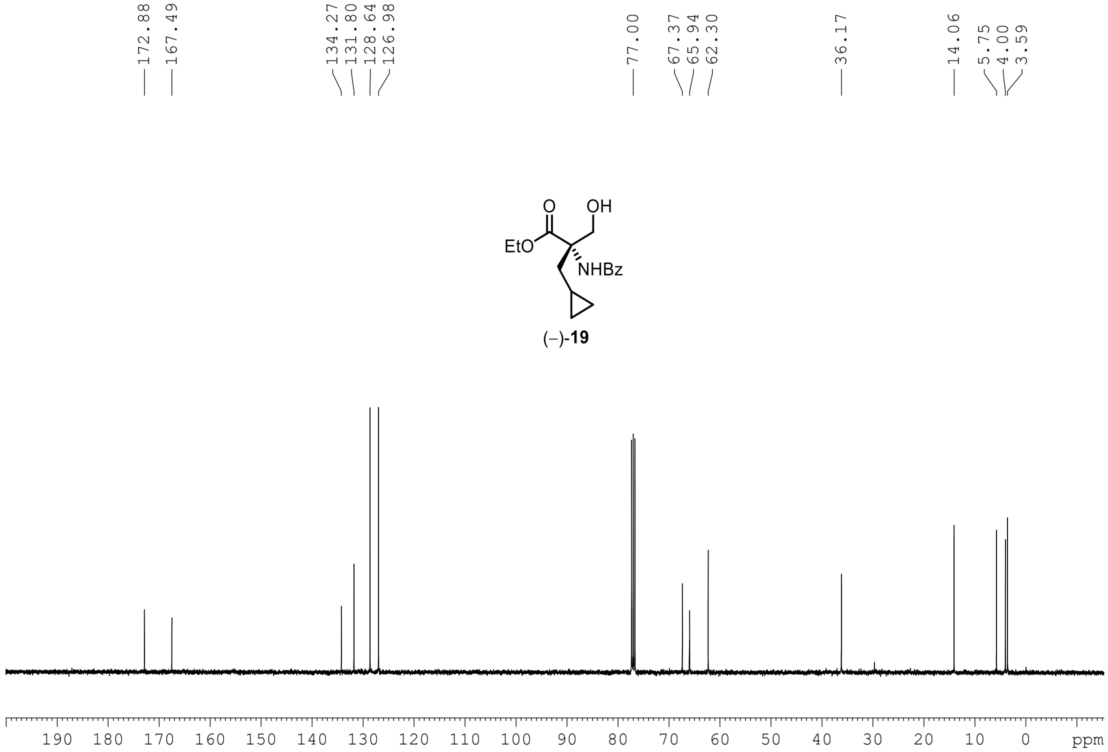

Supplementary Fig. 143. ^{1}H NMR of compound (–)-20 ($CDCl_3$, 400 MHz, 25 °C)

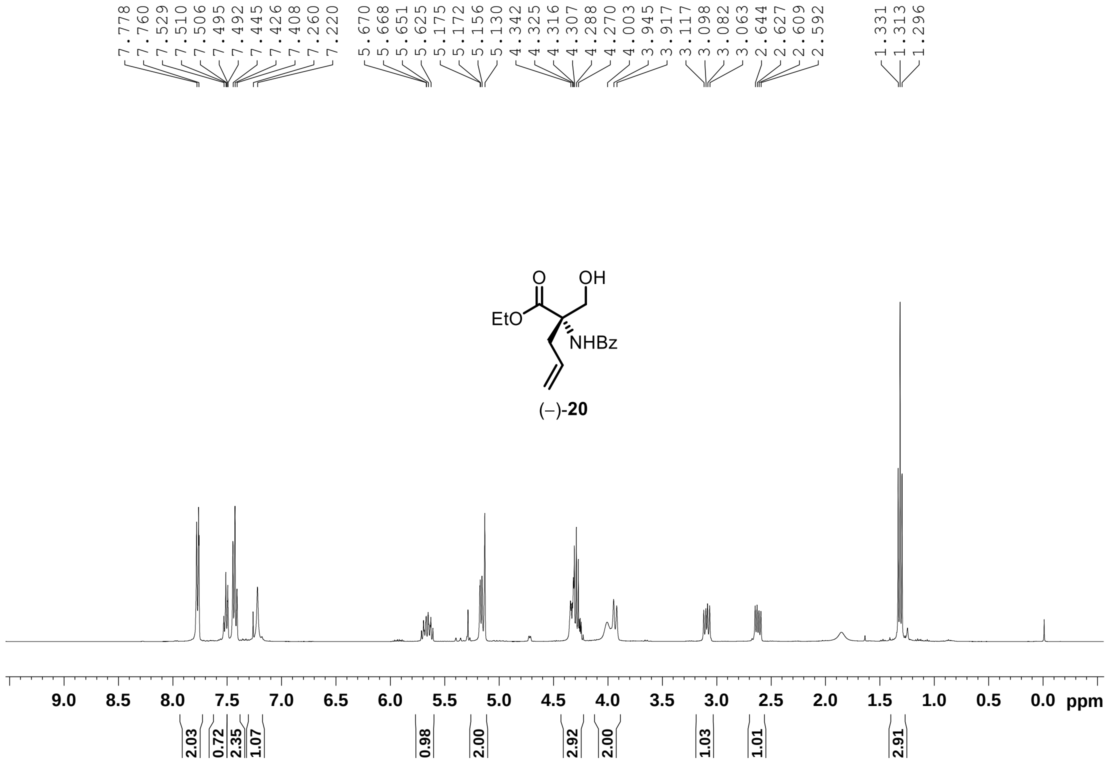

Supplementary Fig. 144. ^{13}C NMR of compound (–)-20 ($CDCl_3$, 100 MHz, 25 °C)

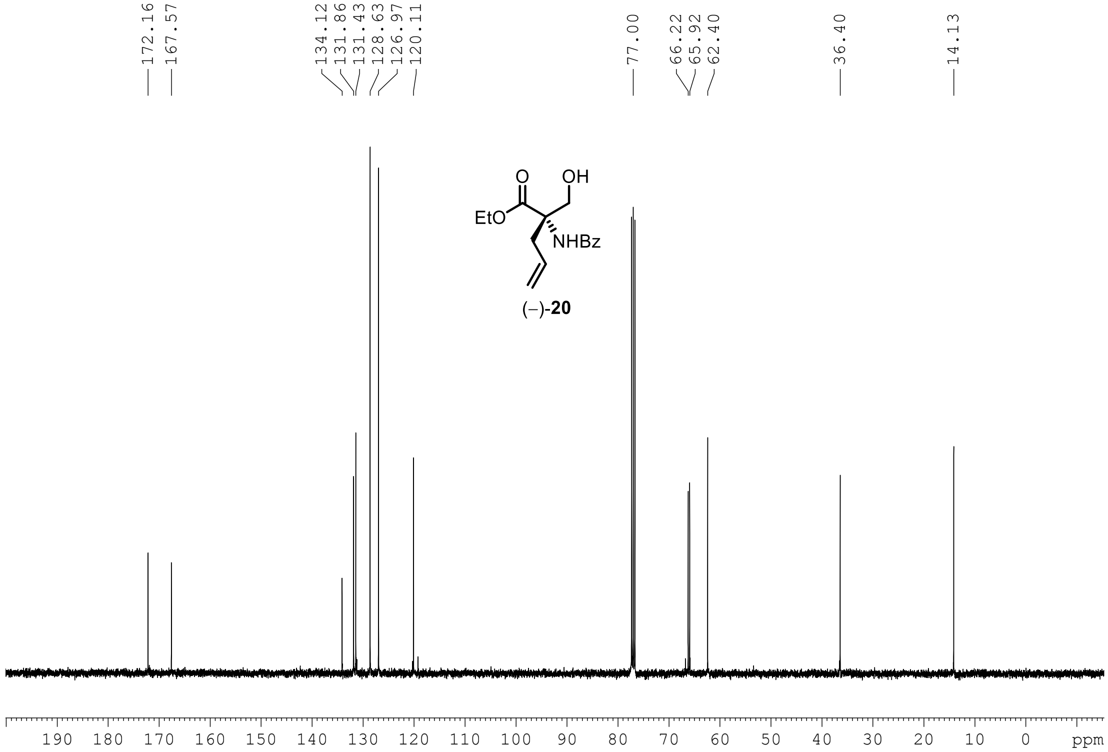

Supplementary Fig. 145. ¹H NMR of compound (–)-21 (CDCl₃, 400 MHz, 25 °C)

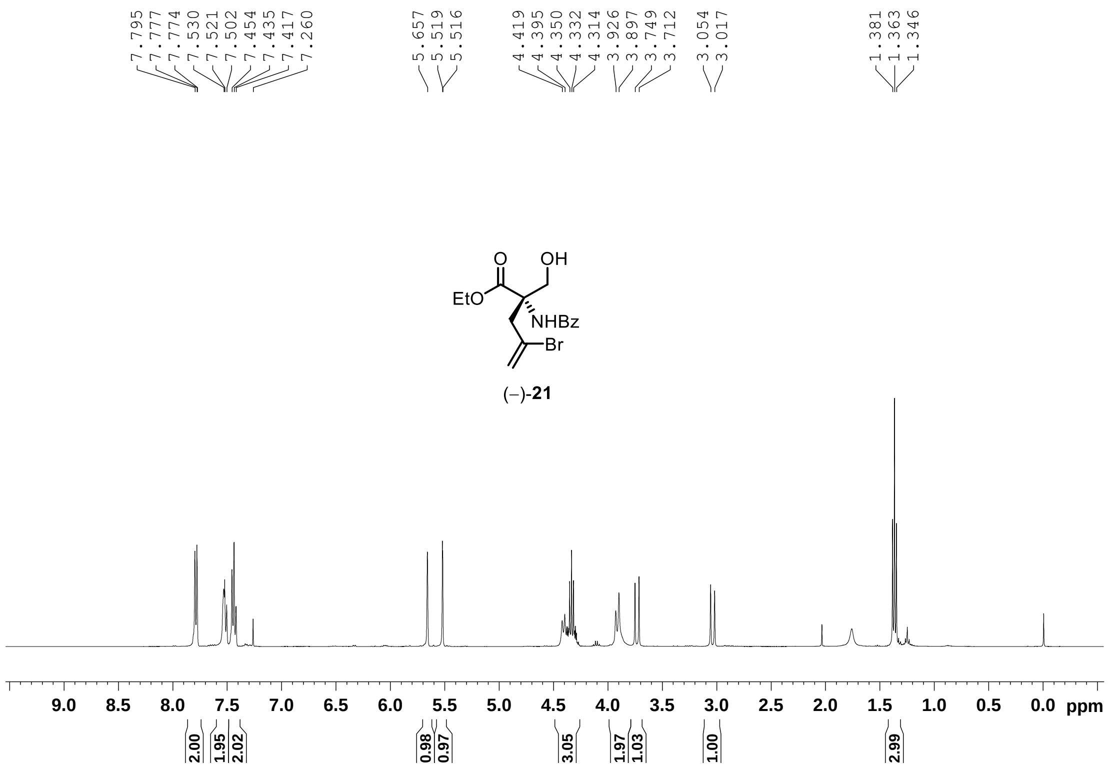

Supplementary Fig. 146. ¹³C NMR of compound (–)-21 (CDCl₃, 100 MHz, 25 °C)

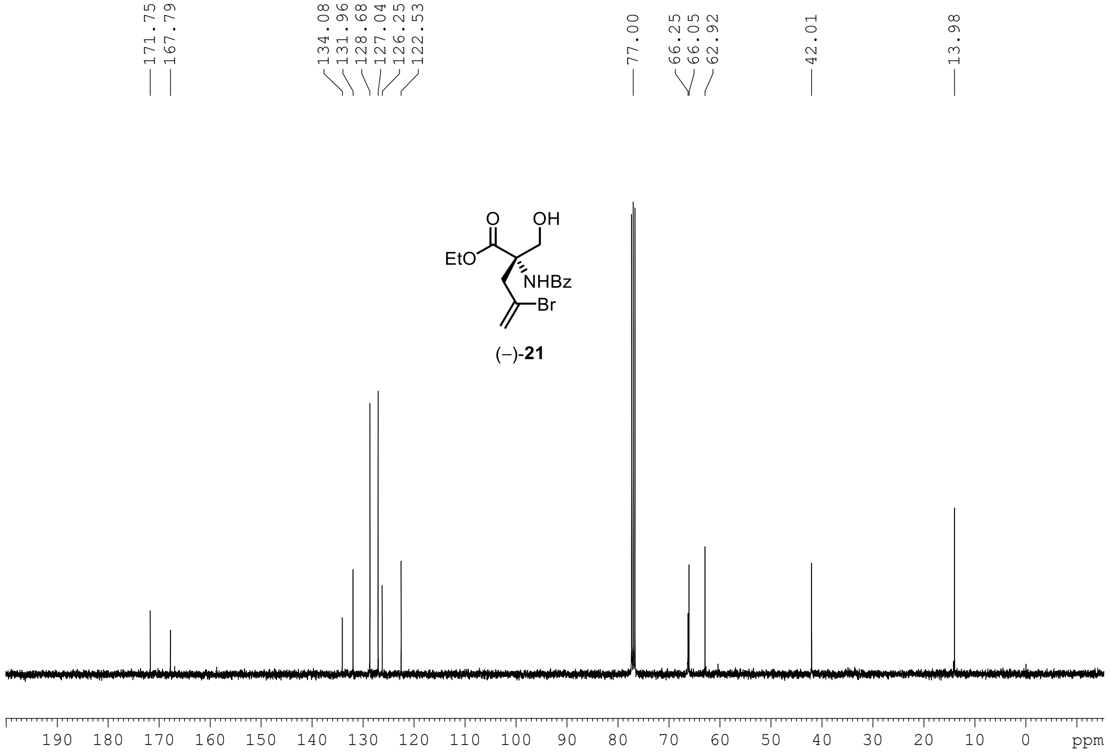

Supplementary Fig. 147. ^{1}H NMR of compound (–)-22 ($CDCl_3$, 400 MHz, 25 °C)

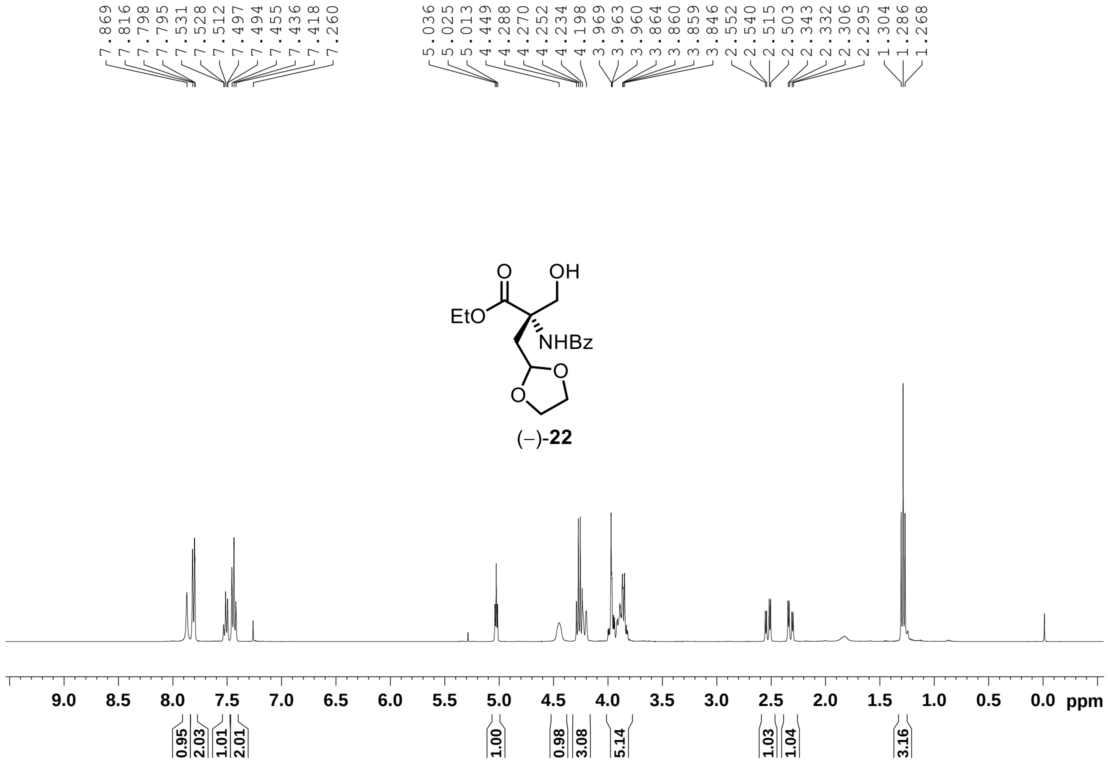

Supplementary Fig. 148. ^{13}C NMR of compound (–)-22 ($CDCl_3$, 100 MHz, 25 °C)

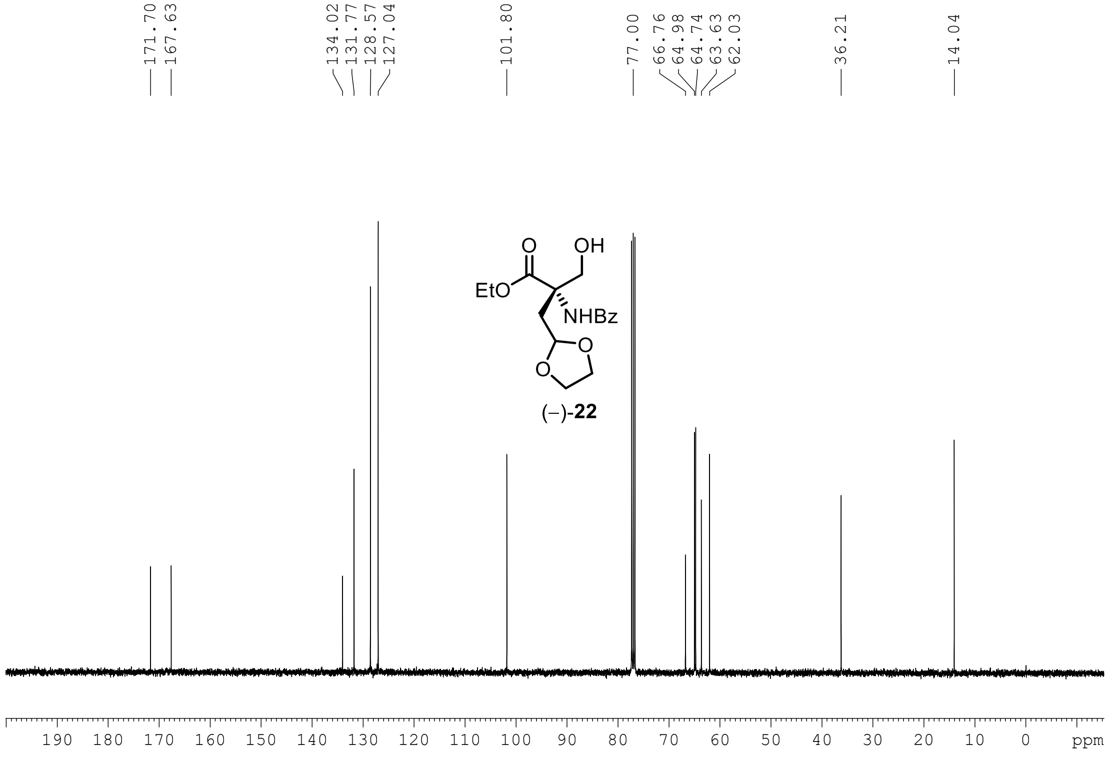

Supplementary Fig. 149. ^{1}H NMR of compound (–)-23 ($CDCl_3$, 400 MHz, 25 °C)

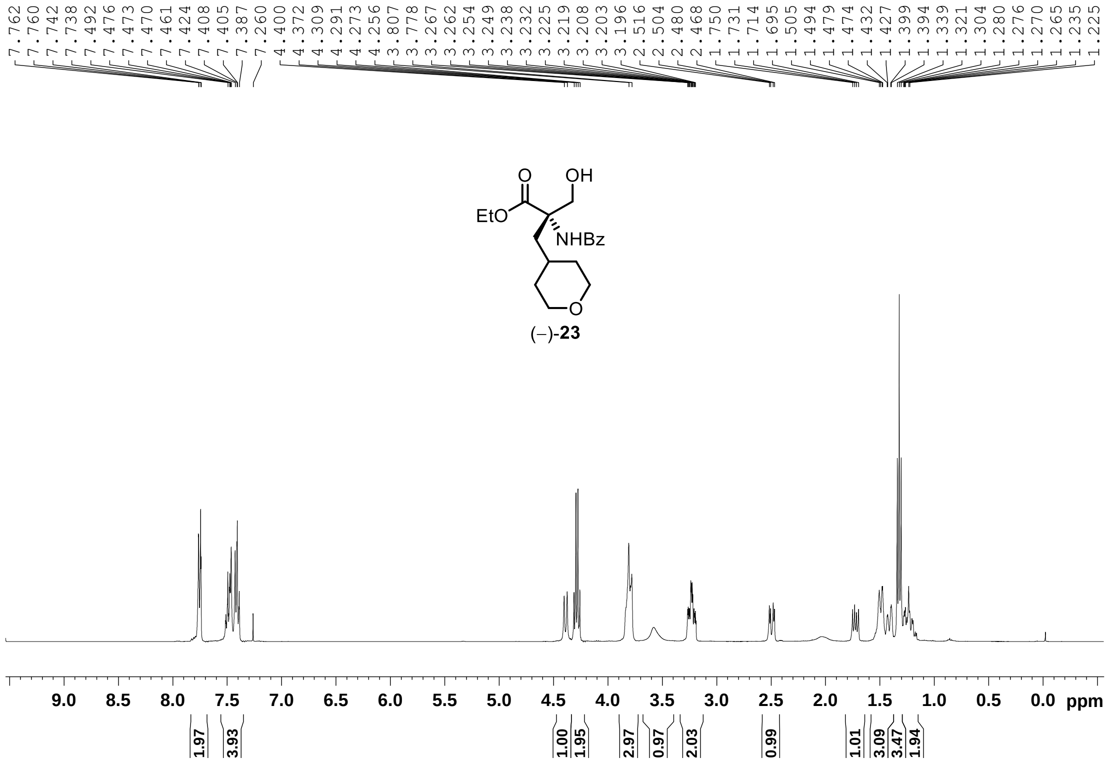

Supplementary Fig. 150. ^{13}C NMR of compound (–)-23 ($CDCl_3$, 100 MHz, 25 °C)

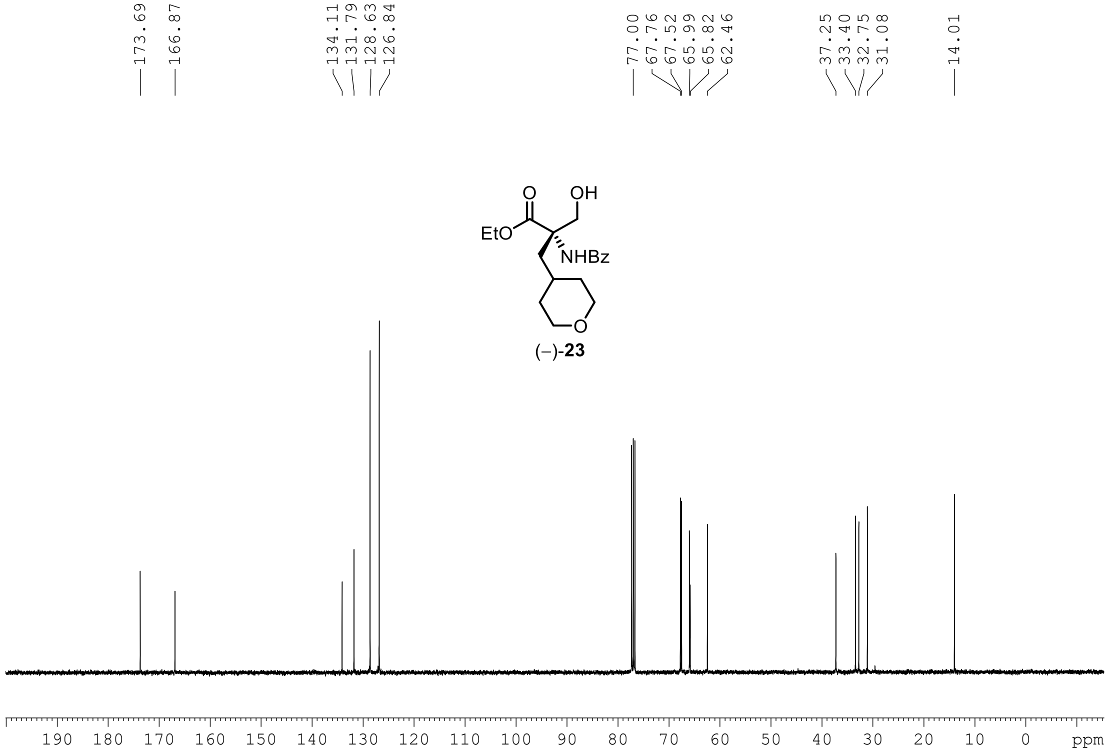

Supplementary Fig. 151. ^{1}H NMR of compound (−)-24 ($CDCl_3$, 400 MHz, 25 °C)

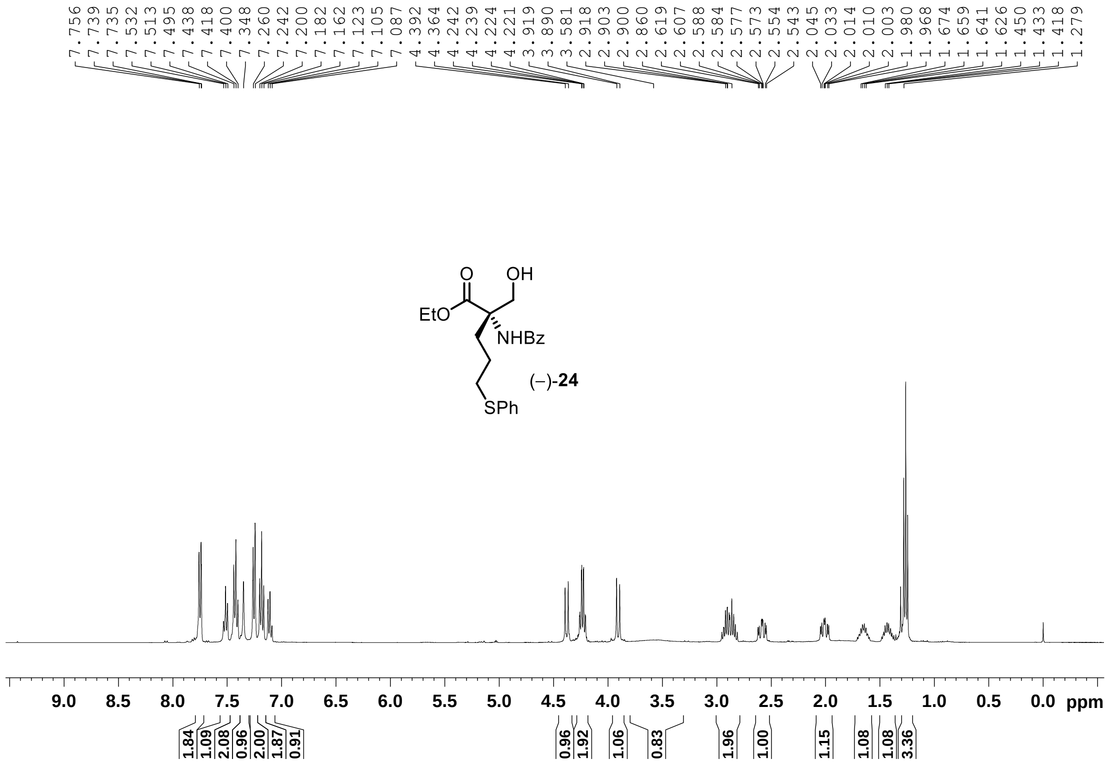

Supplementary Fig. 152. ^{13}C NMR of compound (−)-24 ($CDCl_3$, 100 MHz, 25 °C)

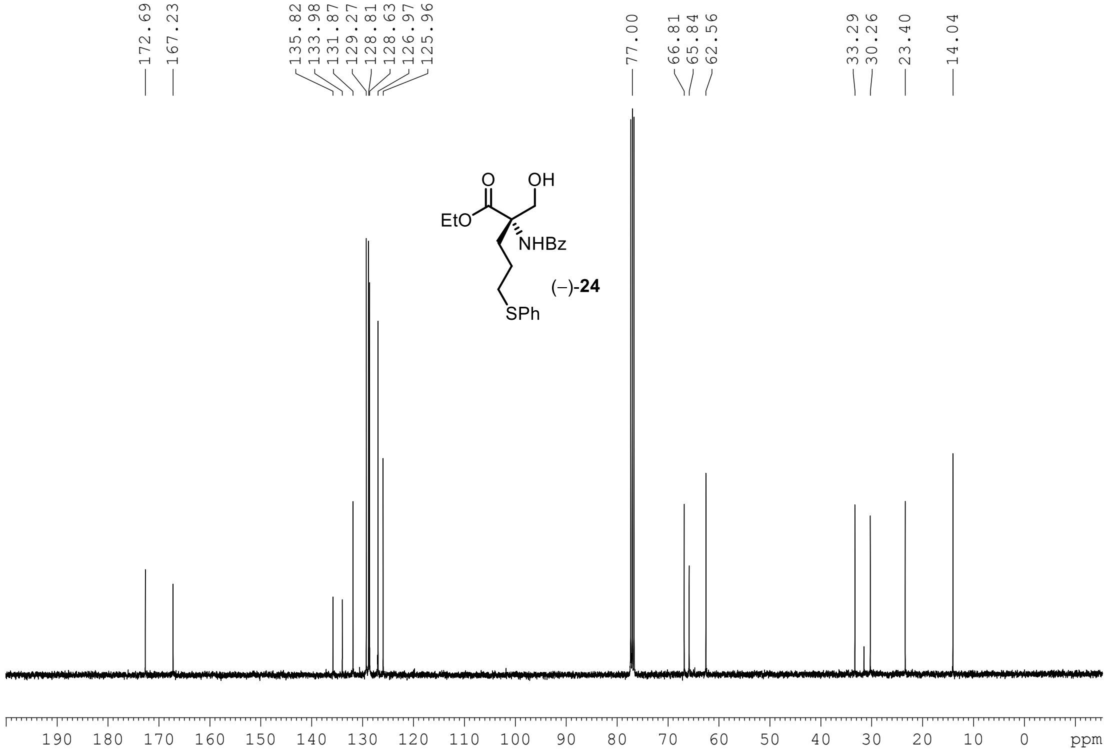

Supplementary Fig. 153. ^{1}H NMR of compound (−)-25 ($CDCl_3$, 400 MHz, 25 °C)

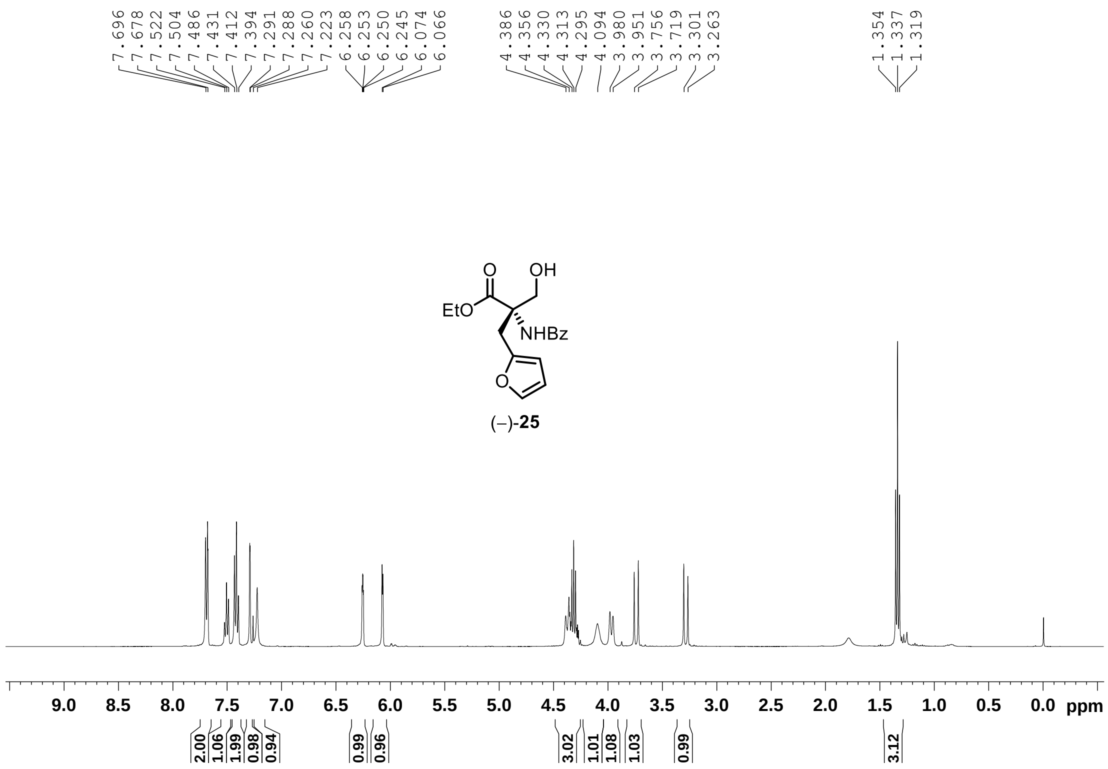

Supplementary Fig. 154. ^{13}C NMR of compound (−)-25 ($CDCl_3$, 100 MHz, 25 °C)

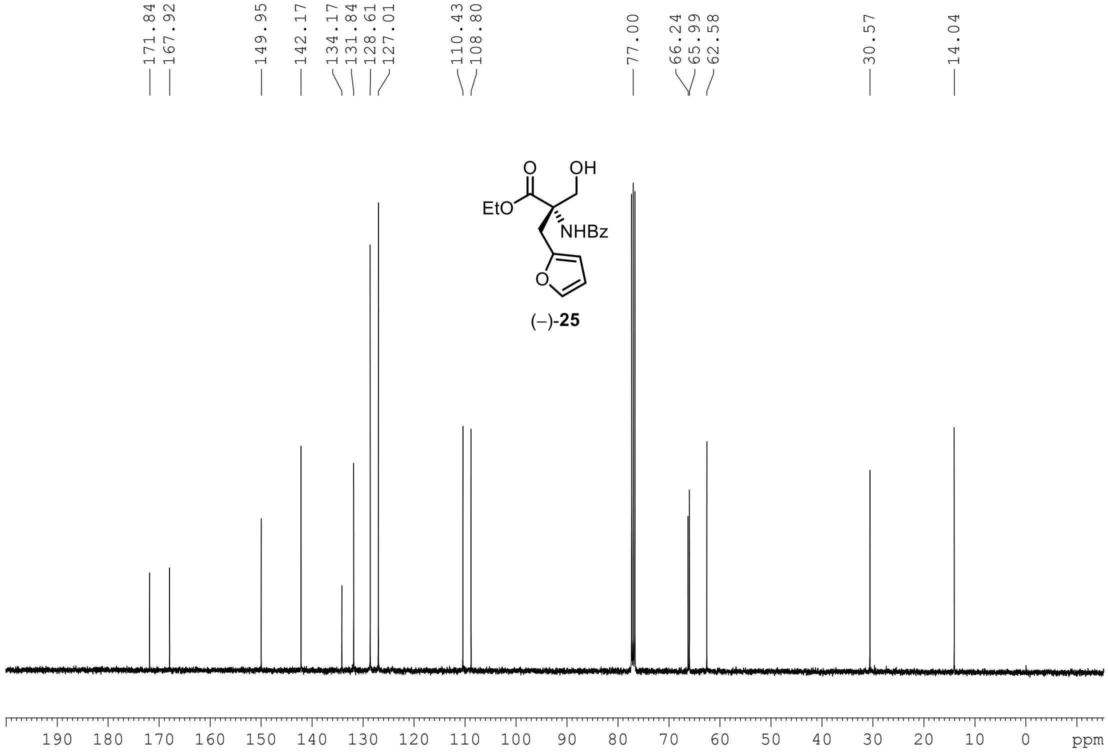

Supplementary Fig. 155. ^{1}H NMR of compound (–)-26 ($CDCl_3$, 400 MHz, 25 °C)

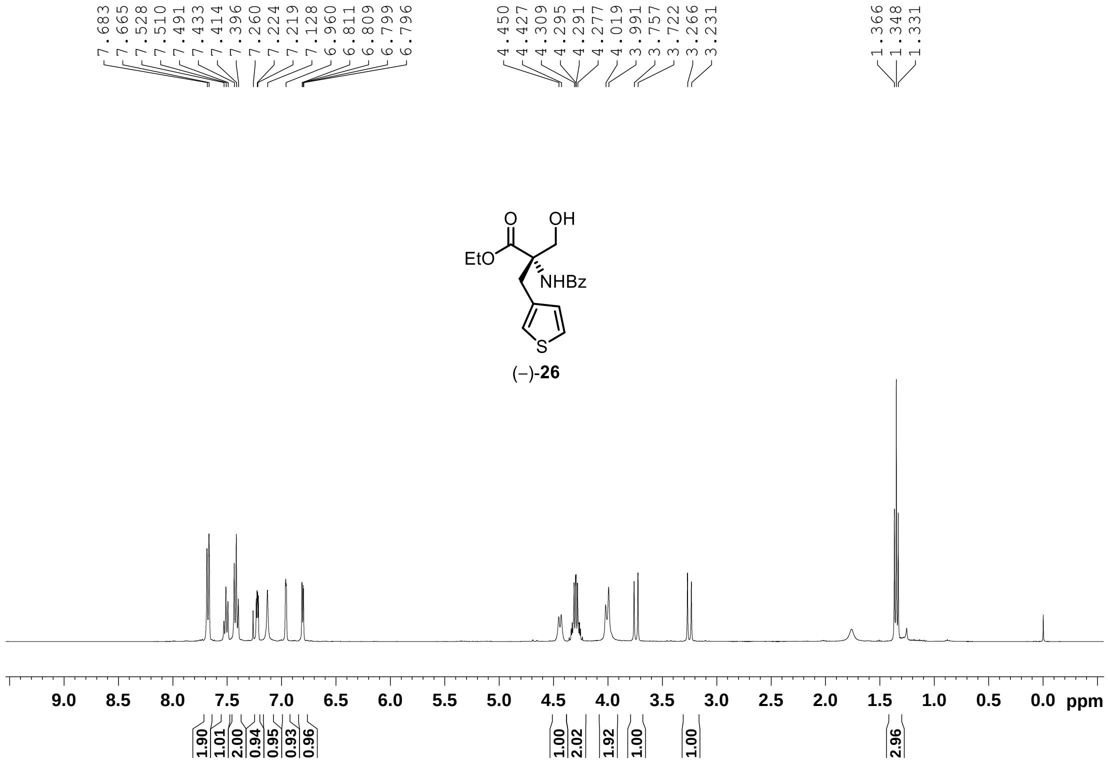

Supplementary Fig. 156. ^{13}C NMR of compound (–)-26 ($CDCl_3$, 100 MHz, 25 °C)

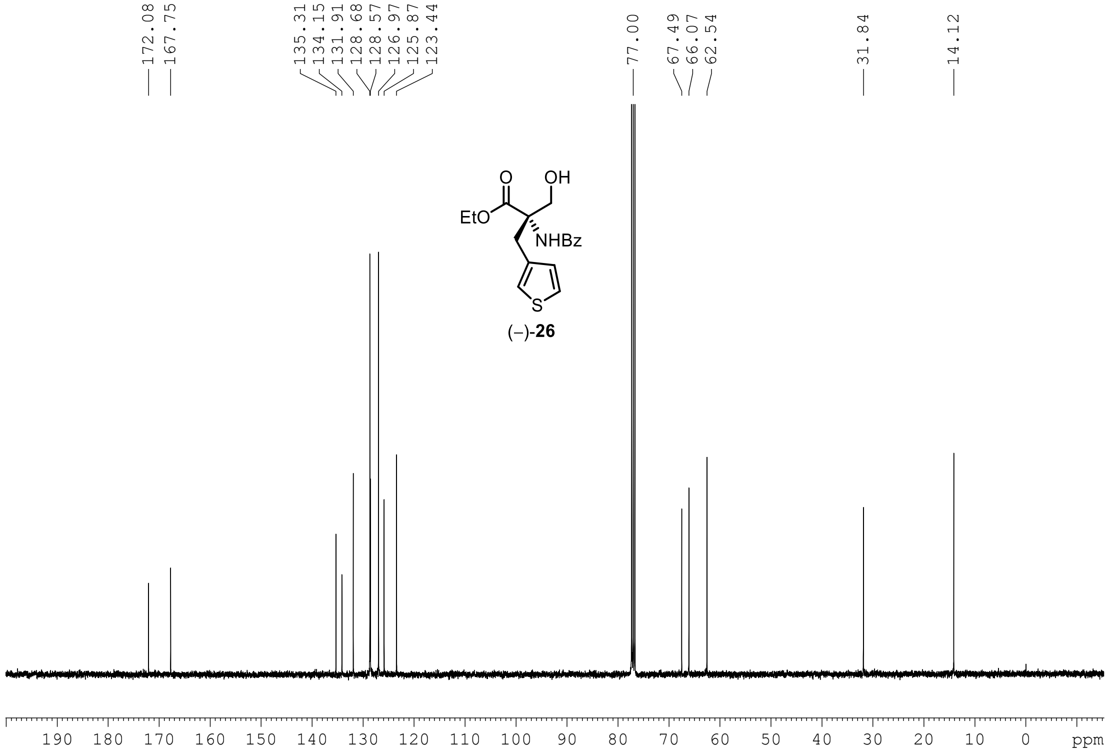

Supplementary Fig. 157. ¹H NMR of compound (−)-27 (CDCl₃, 400 MHz, 25 °C)

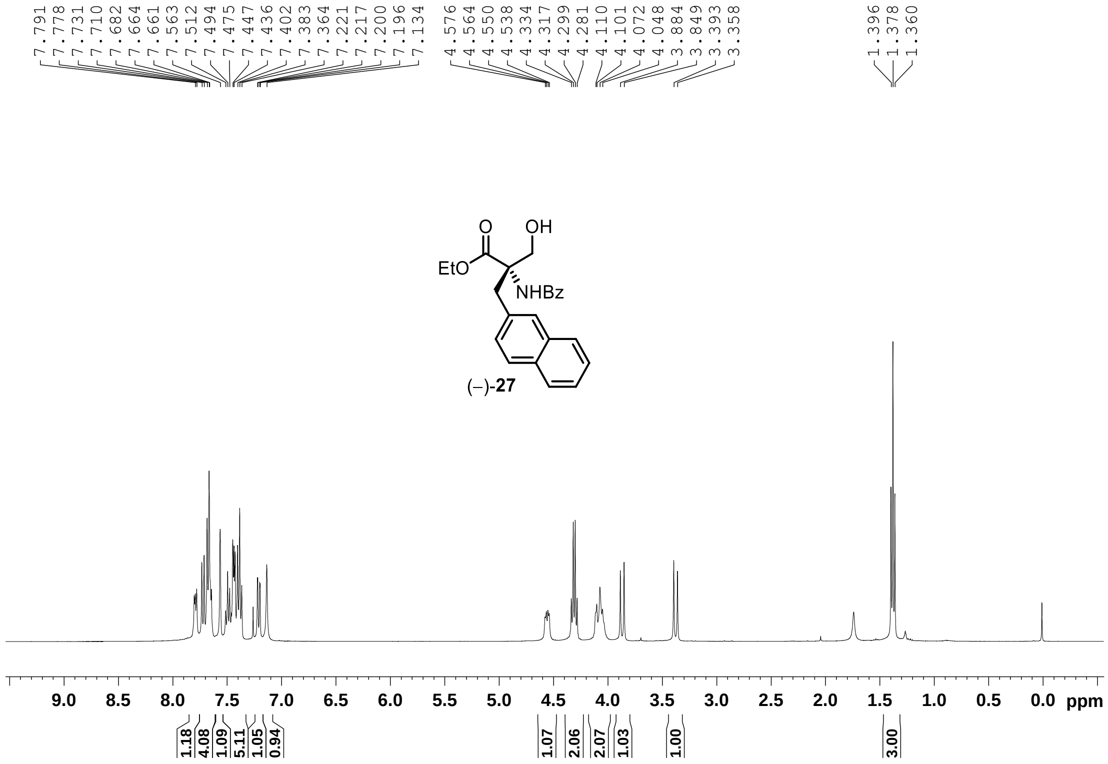

Supplementary Fig. 158. ¹³C NMR of compound (−)-27 (CDCl₃, 100 MHz, 25 °C)

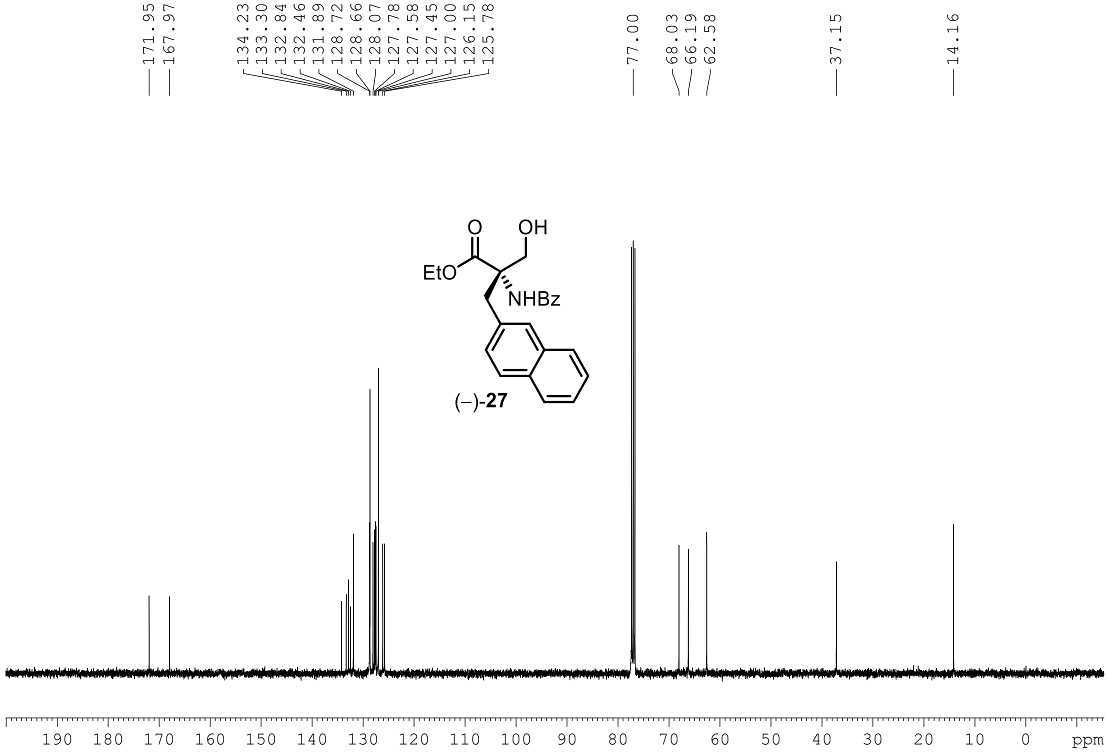

Supplementary Fig. 159. ^{1}H NMR of compound (–)-28 ($CDCl_3$, 400 MHz, 25 °C)

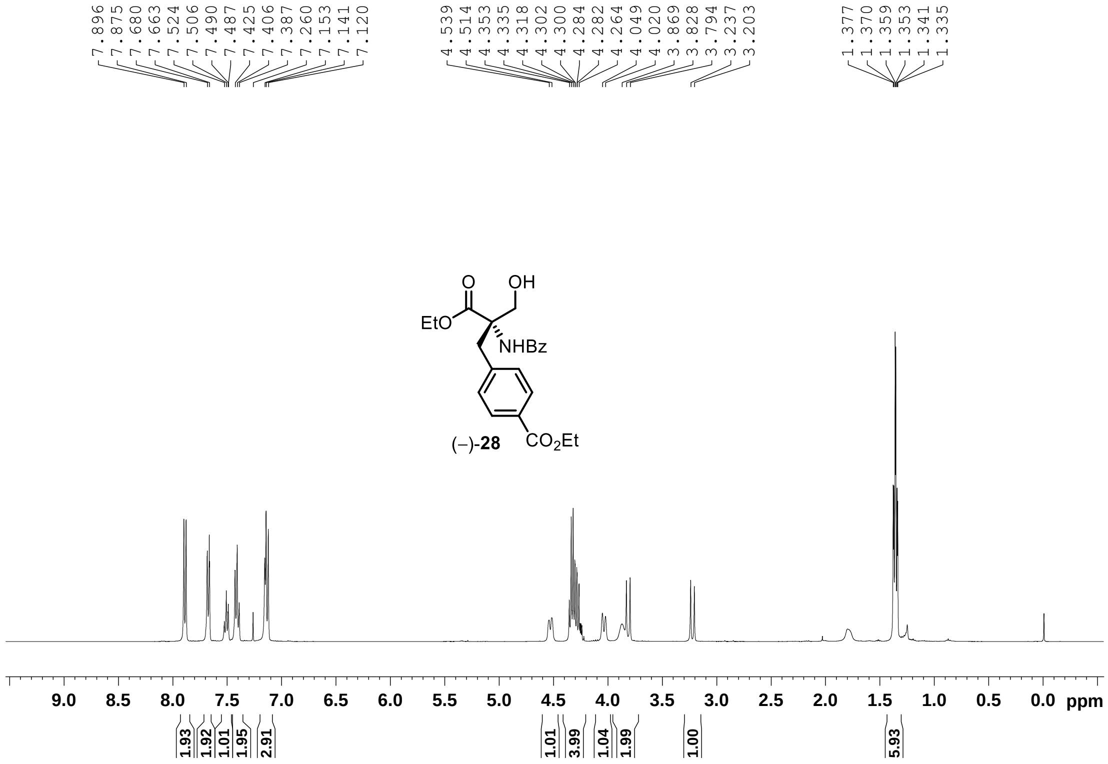

Supplementary Fig. 160. ^{13}C NMR of compound (–)-28 ($CDCl_3$, 100 MHz, 25 °C)

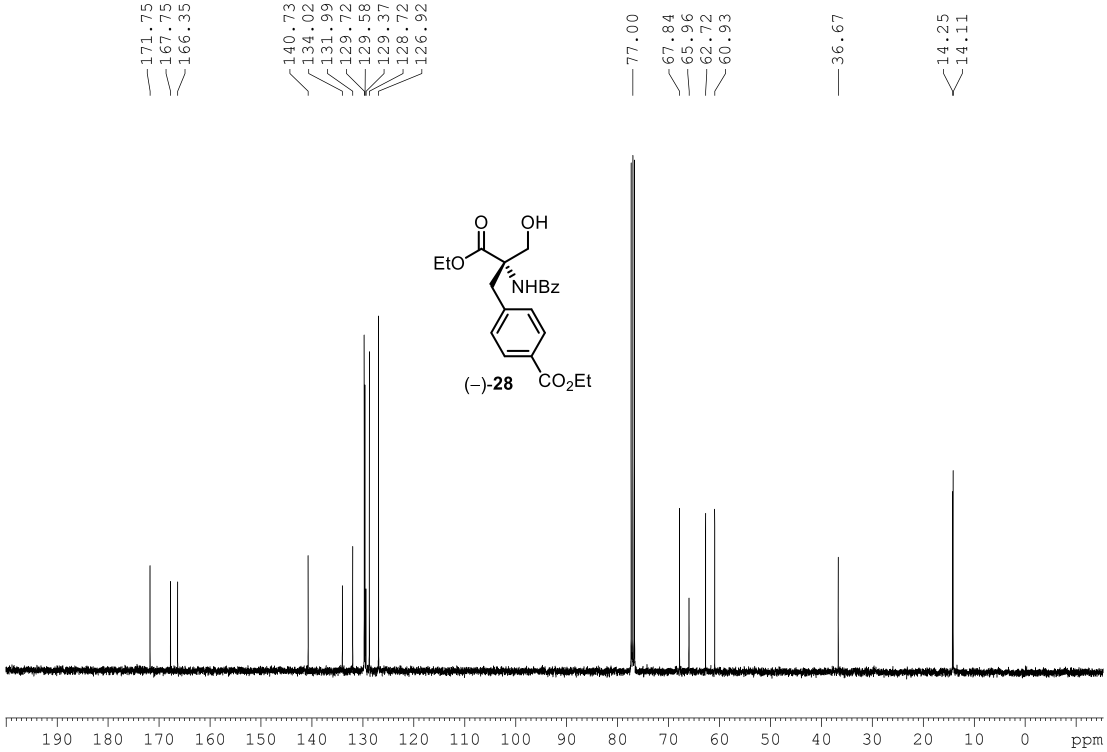

Supplementary Fig. 161. ^{1}H NMR of compound (−)-29 ($CDCl_3$, 400 MHz, 25 °C)

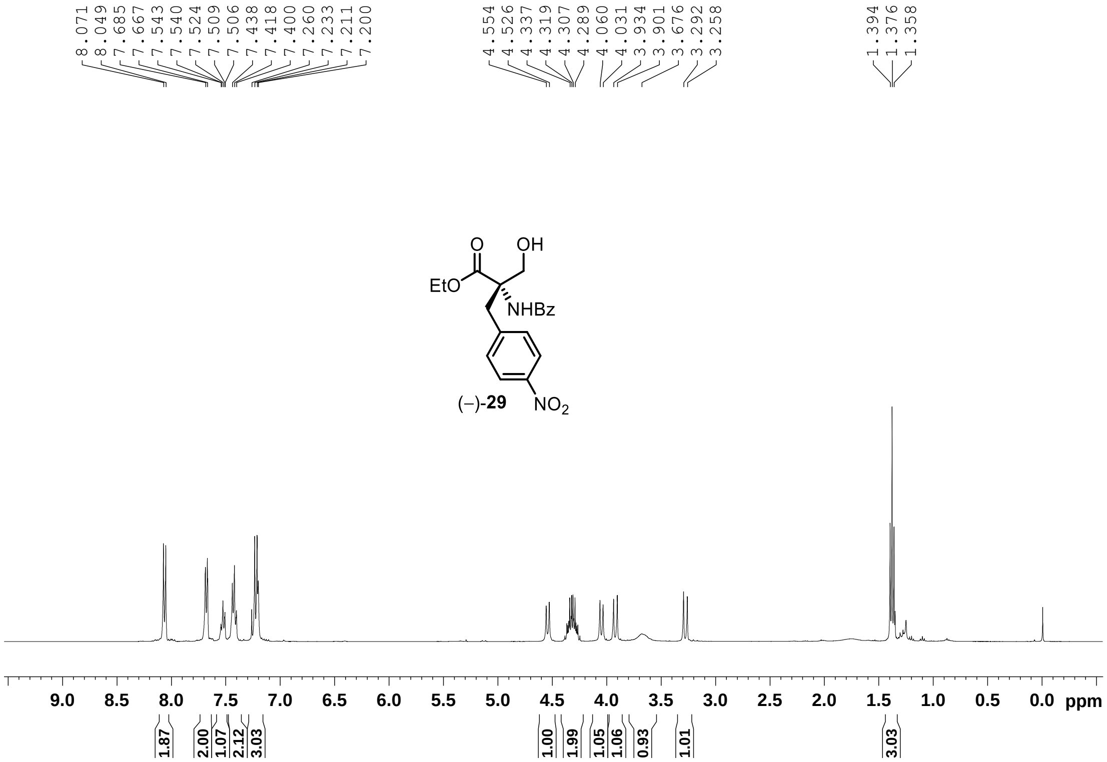

Supplementary Fig. 162. ^{13}C NMR of compound (−)-29 ($CDCl_3$, 100 MHz, 25 °C)

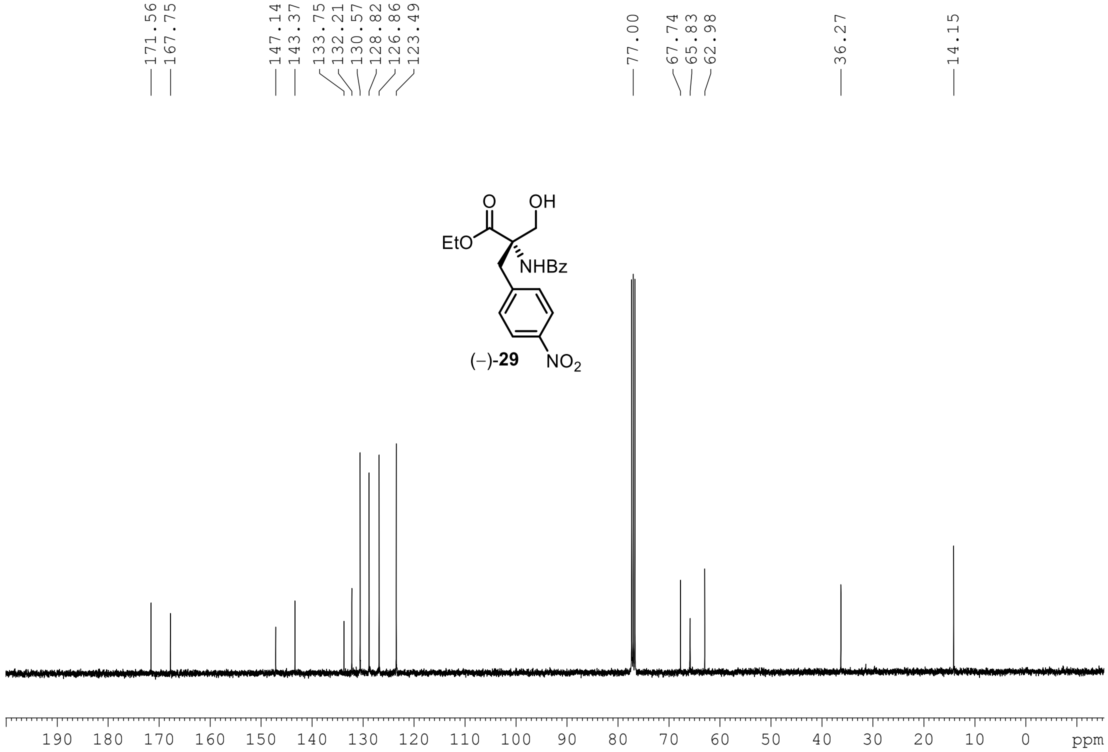

Supplementary Fig. 163. ^{1}H NMR of compound (–)-30 ($CDCl_3$, 400 MHz, 25 °C)

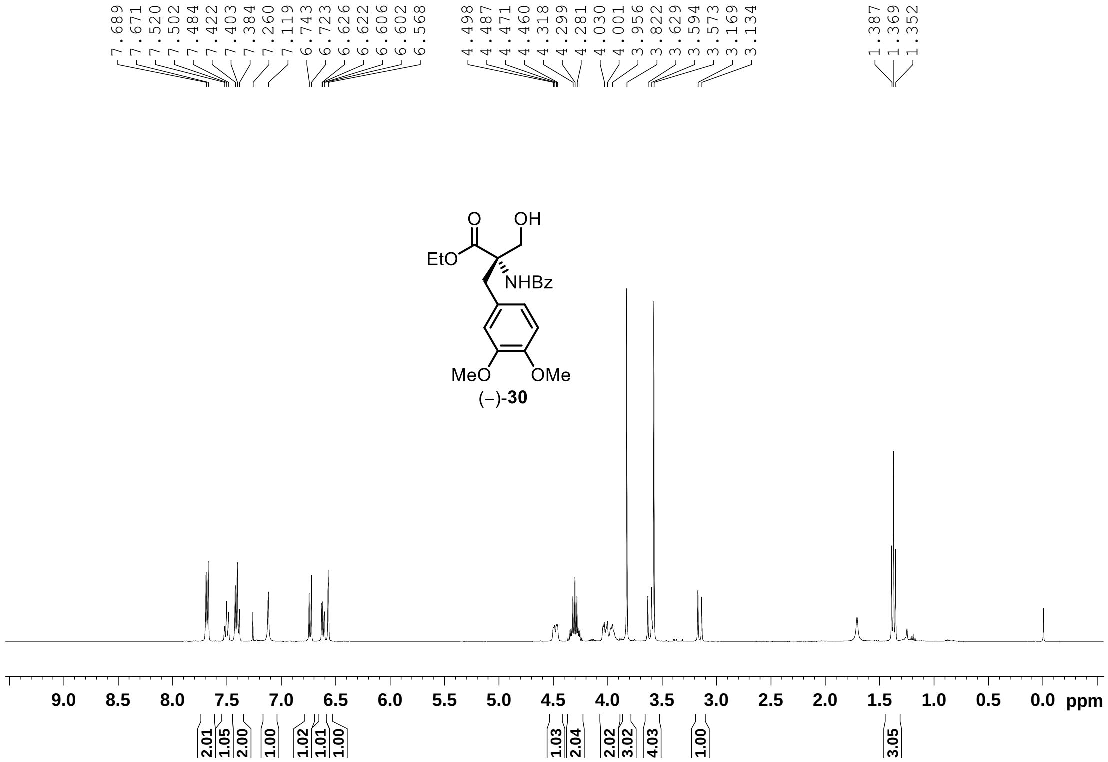

Supplementary Fig. 164. ^{13}C NMR of compound (–)-30 ($CDCl_3$, 100 MHz, 25 °C)

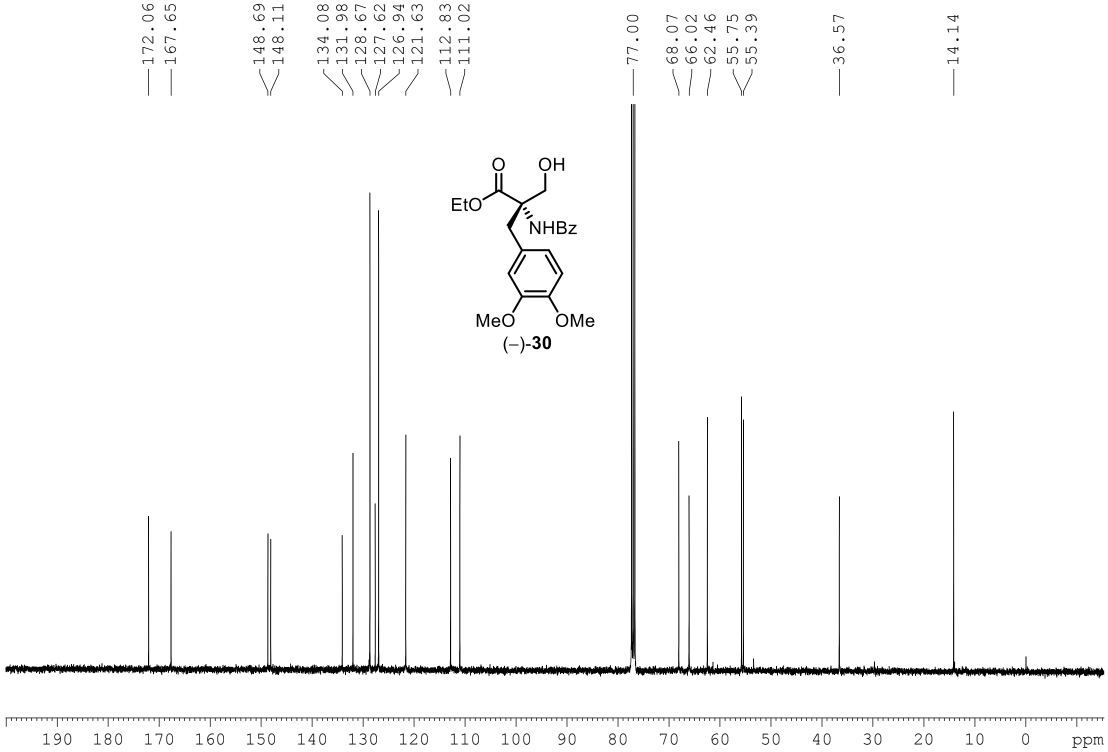

Supplementary Fig. 165. ¹H NMR of compound (−)-31 (CDCl₃, 400 MHz, 25 °C)

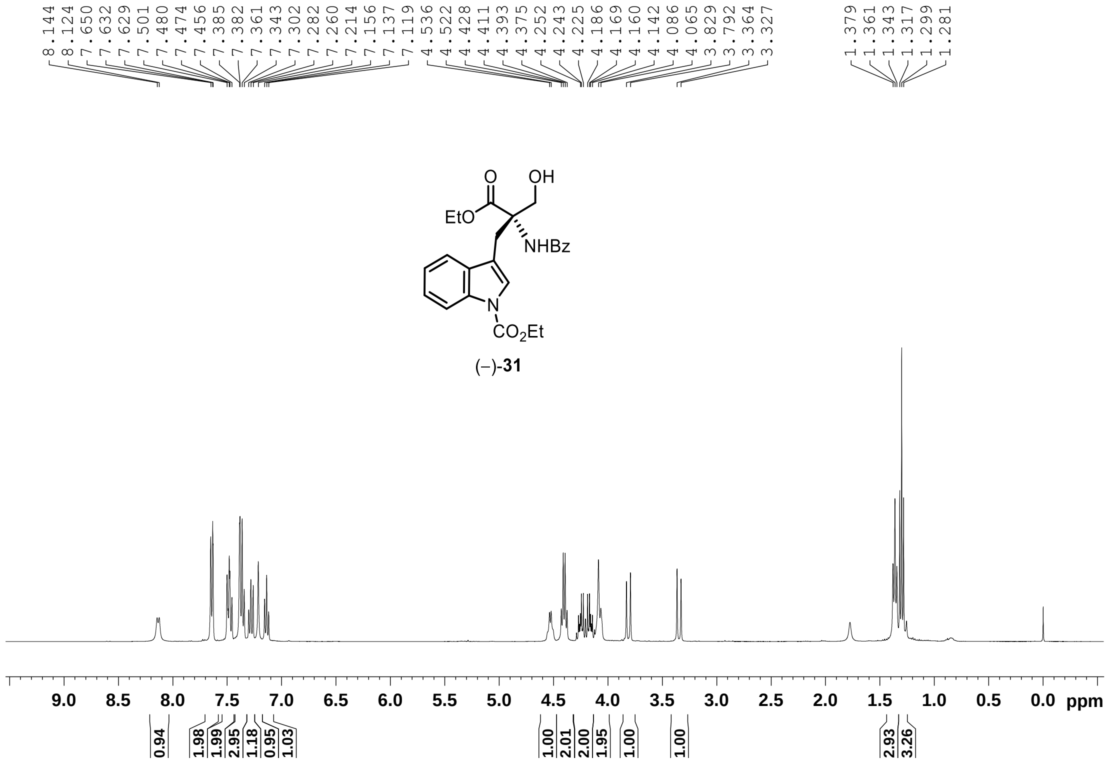

Supplementary Fig. 166. ¹³C NMR of compound (−)-31 (CDCl₃, 100 MHz, 25 °C)

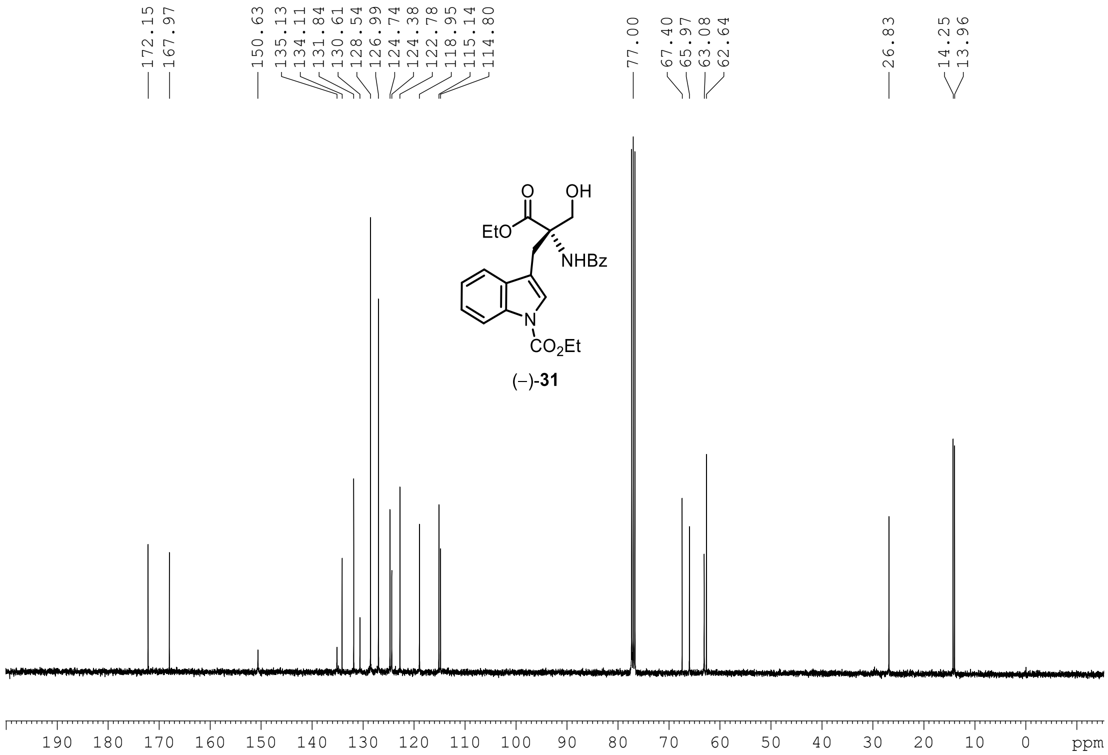

Supplementary Fig. 167. ¹H NMR of compound (–)-32 ($CDCl_3$, 400 MHz, 25 °C)

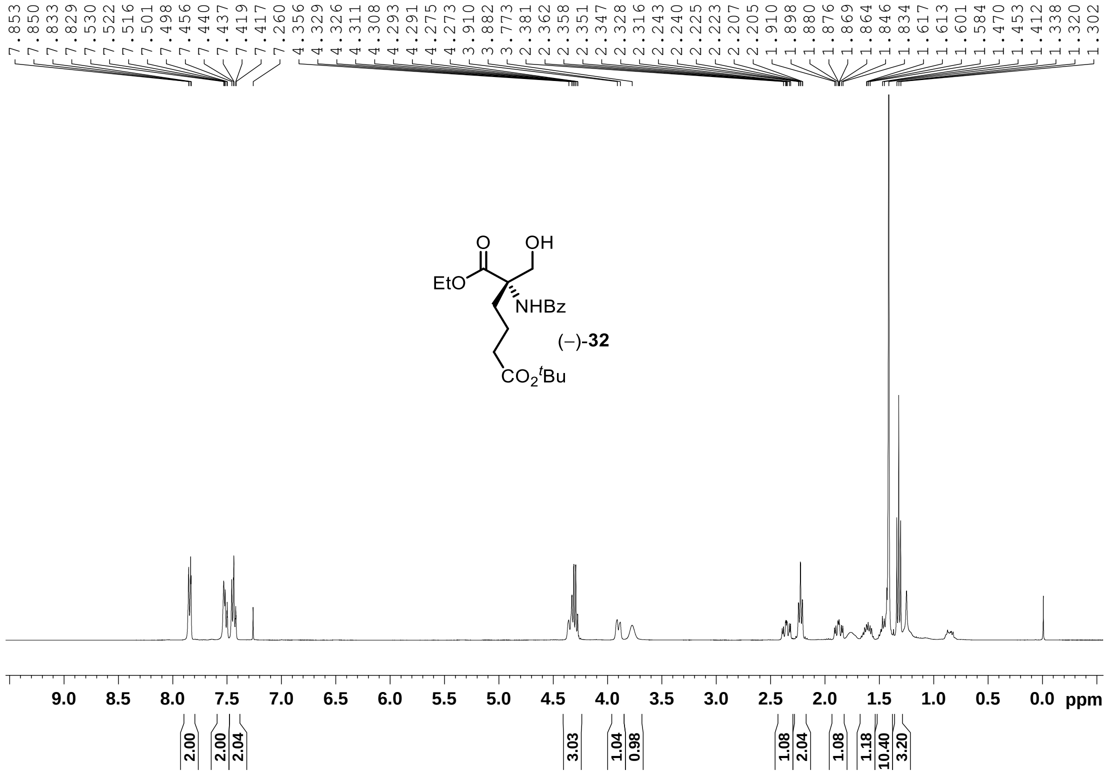

Supplementary Fig. 168. ¹³C NMR of compound (–)-32 ($CDCl_3$, 100 MHz, 25 °C)

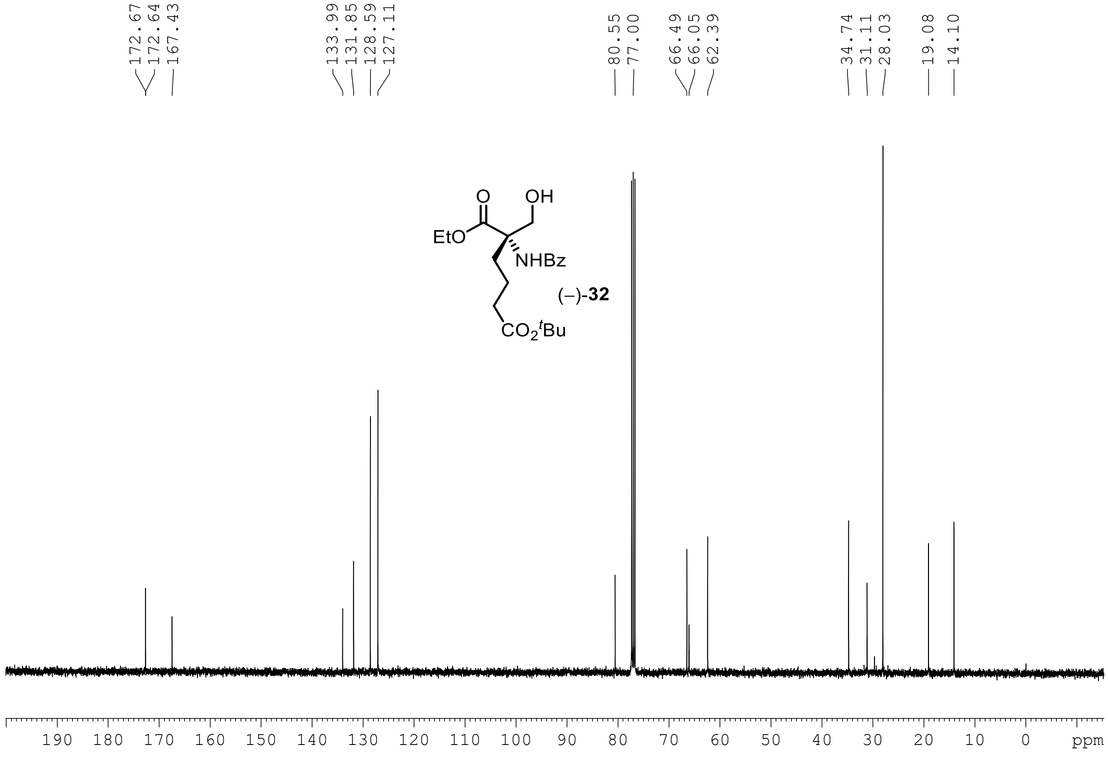

Supplementary Fig. 169. ^{1}H NMR of compound (–)-33 ($CDCl_3$, 400 MHz, 25 °C)

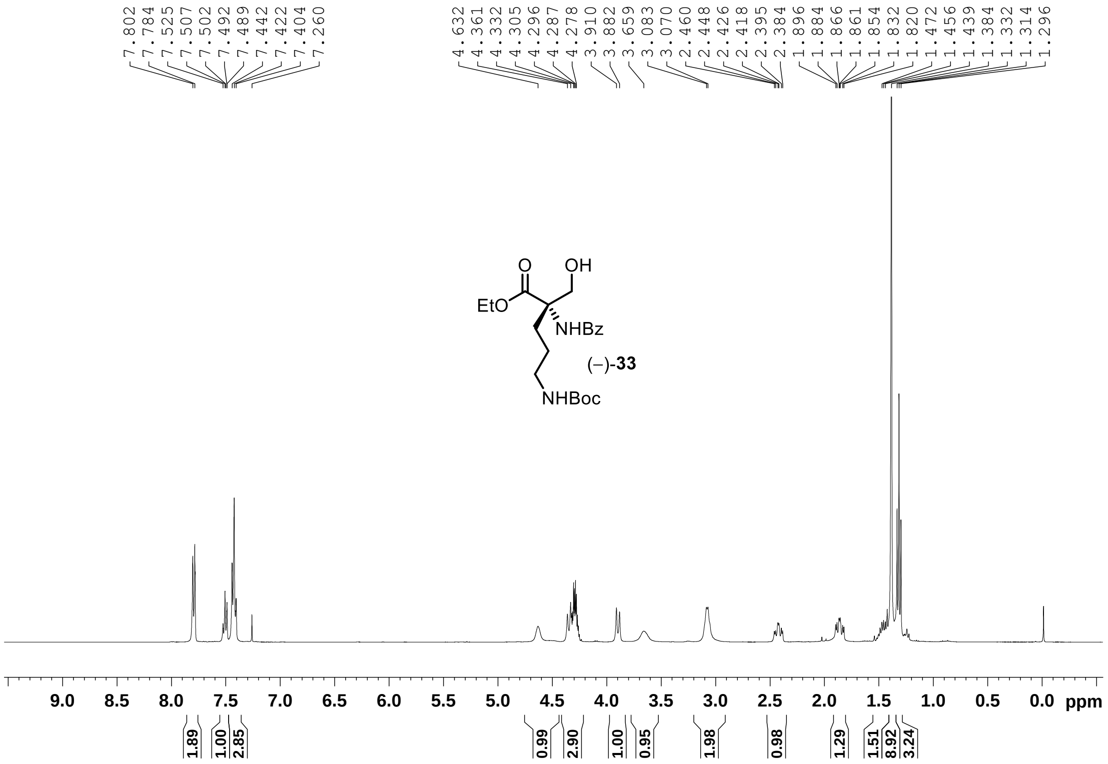

Supplementary Fig. 170. ^{13}C NMR of compound (–)-33 ($CDCl_3$, 100 MHz, 25 °C)

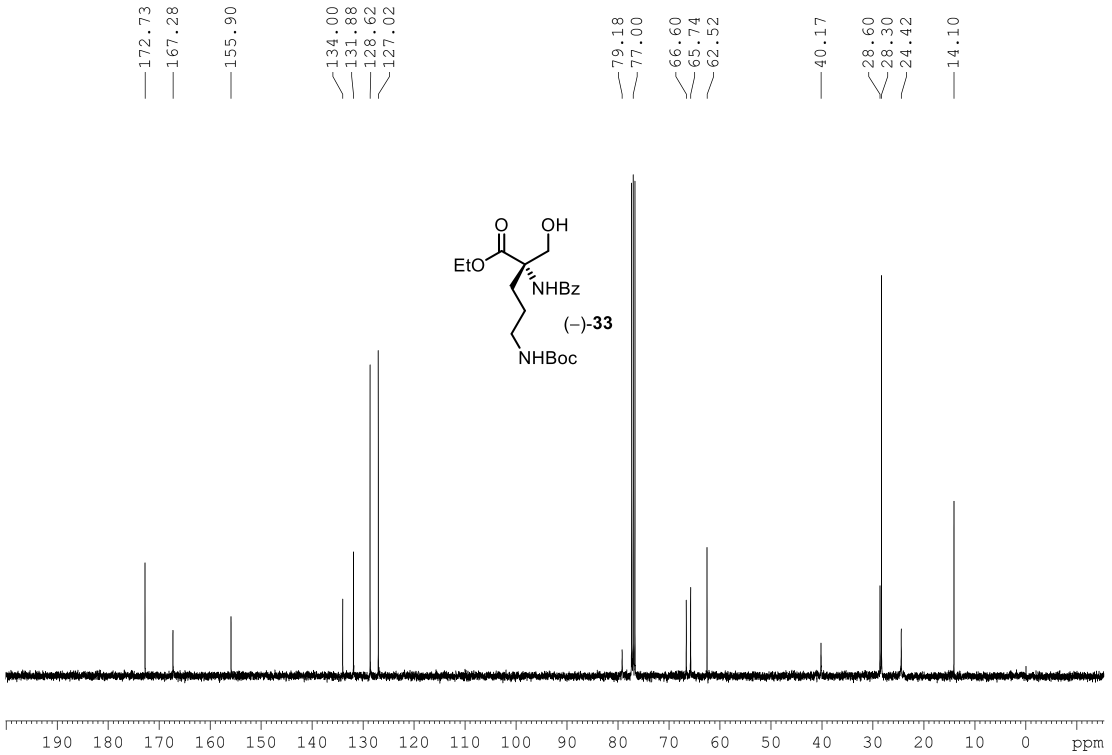

Supplementary Fig. 171. ^{1}H NMR of compound (–)-34 ($CDCl_3$, 400 MHz, 25 °C)

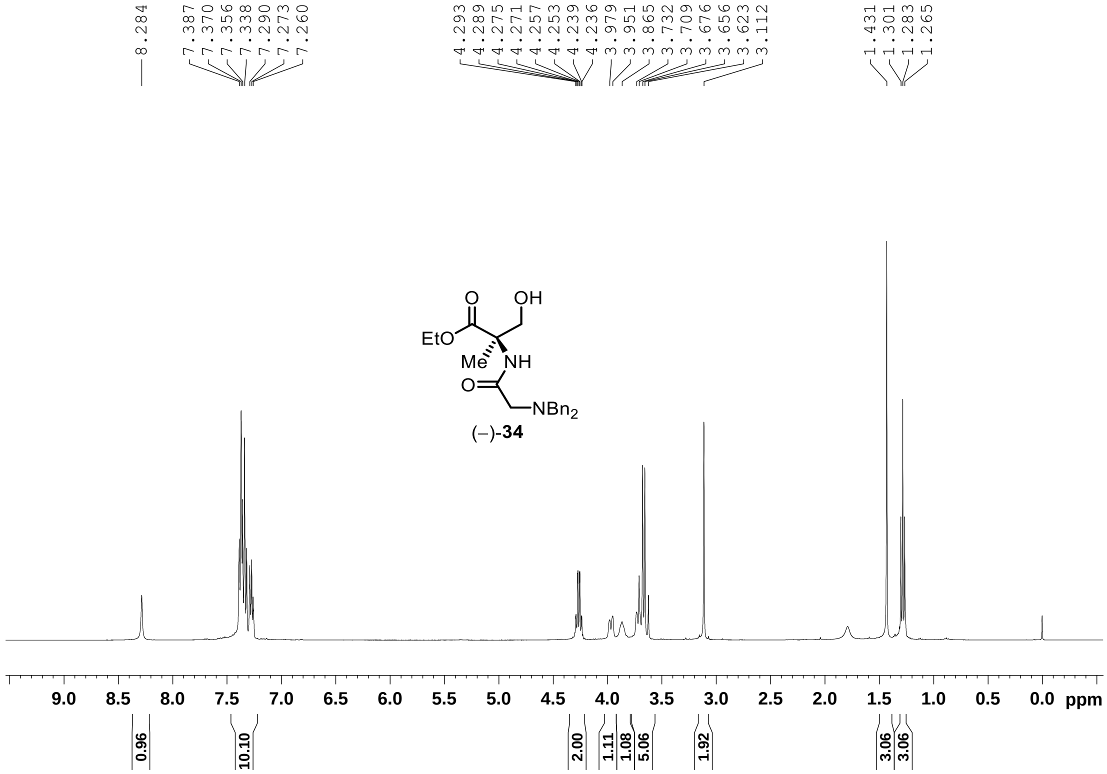

Supplementary Fig. 172. ^{13}C NMR of compound (–)-34 ($CDCl_3$, 100 MHz, 25 °C)

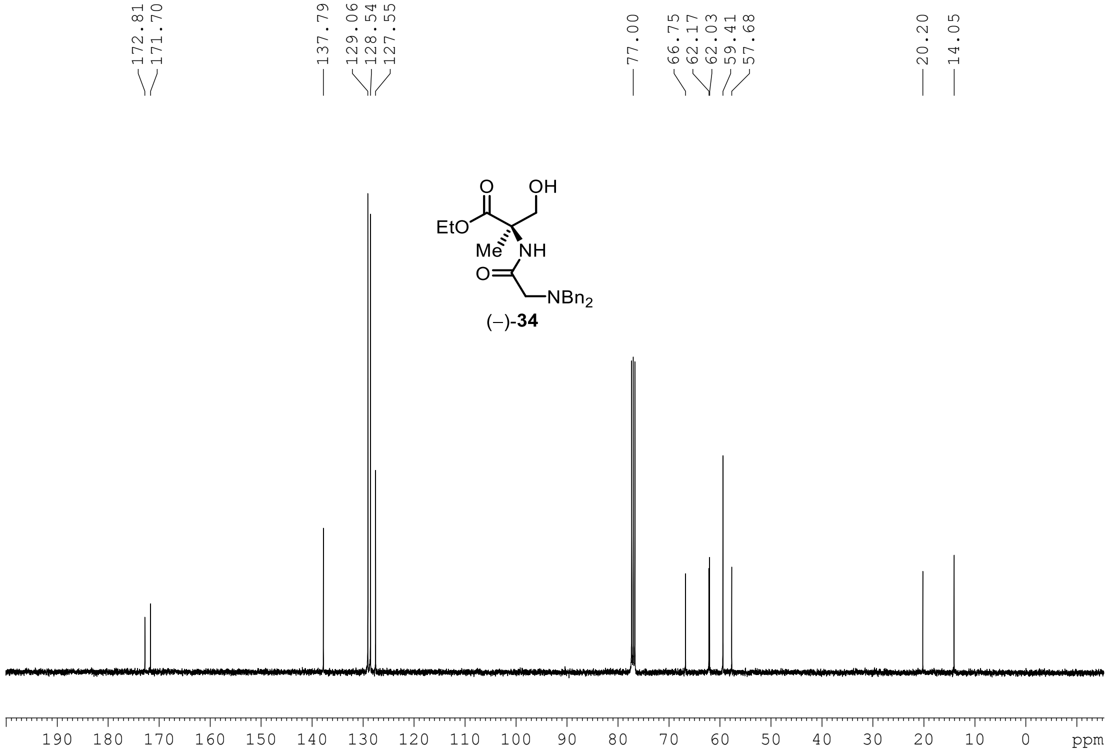

Supplementary Fig. 173. ¹H NMR of compound (–)-35 (CDCl₃, 400 MHz, 25 °C)

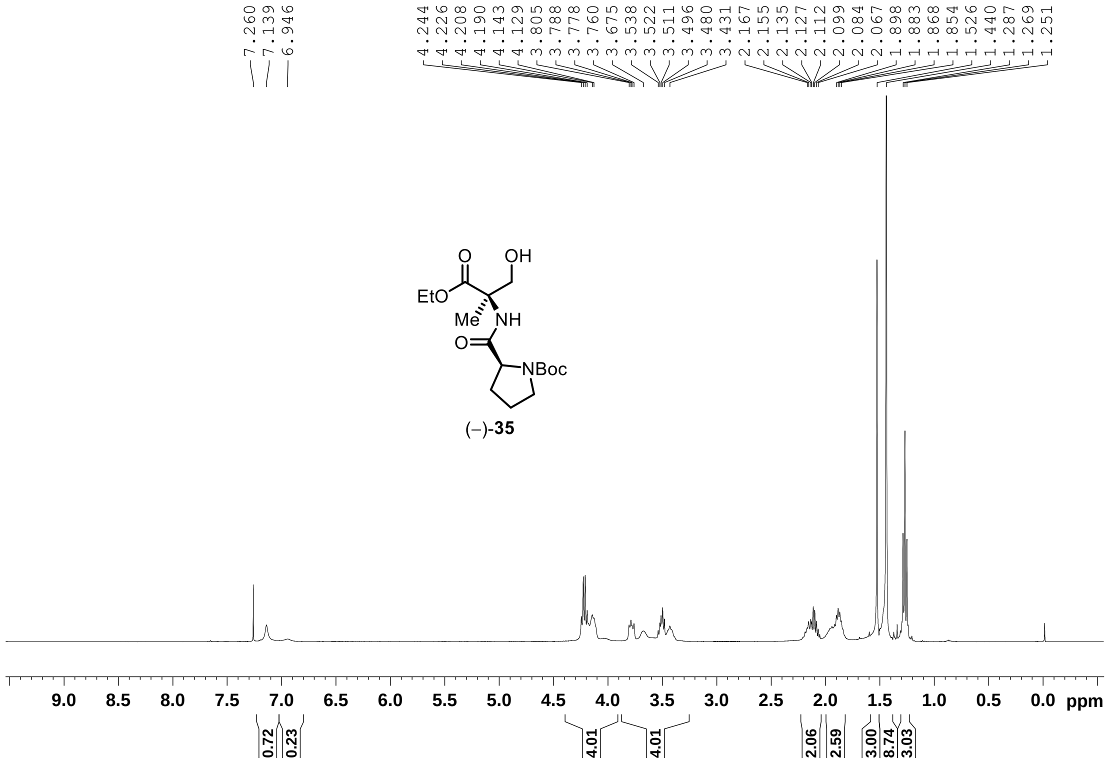

Supplementary Fig. 174. ¹³C NMR of compound (–)-35 (CDCl₃, 100 MHz, 25 °C)

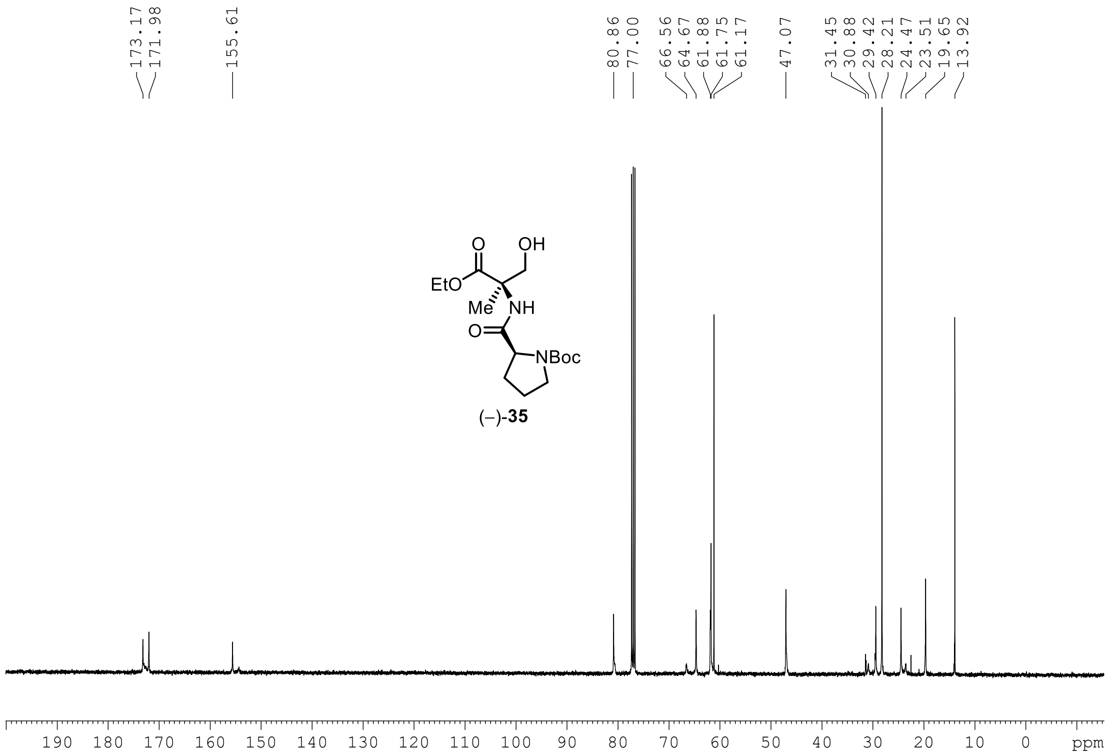

Supplementary Fig. 175. ¹H NMR of compound (–)-36 ($CDCl_3$, 400 MHz, 25 °C)

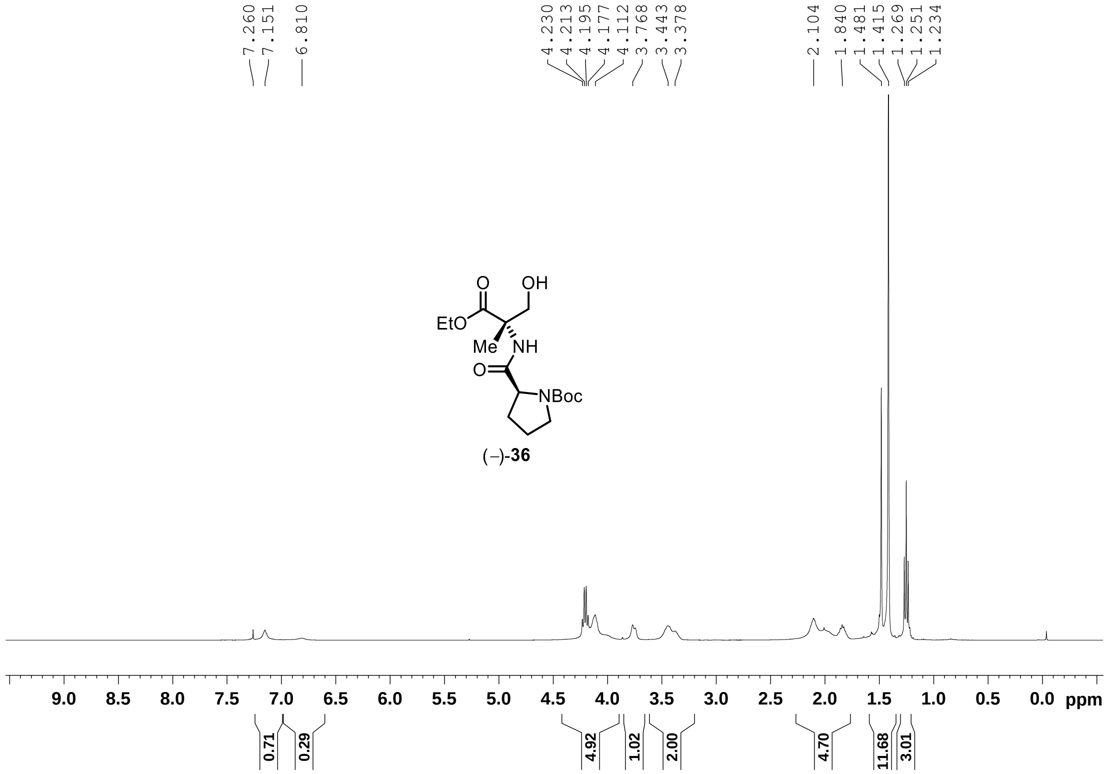

Supplementary Fig. 176. ¹³C NMR of compound (–)-36 ($CDCl_3$, 100 MHz, 25 °C)

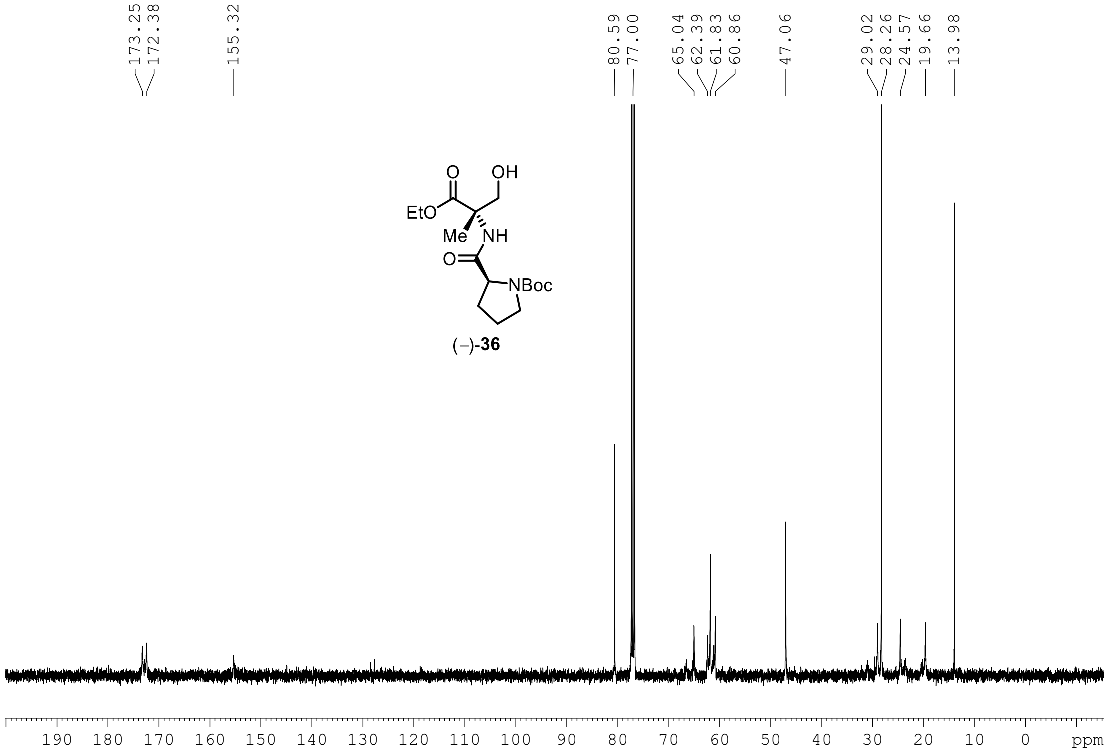

Supplementary Fig. 177. ^{1}H NMR of compound (+)-37 ($CDCl_3$, 400 MHz, 25 °C)

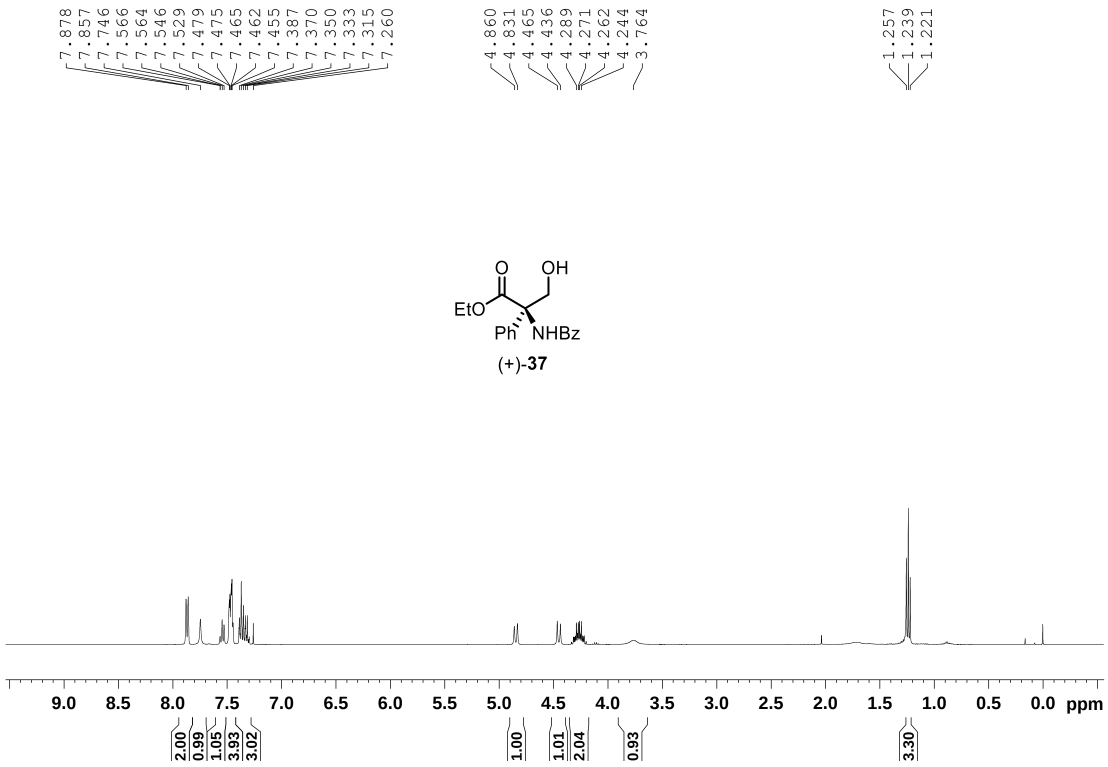

Supplementary Fig. 178. ^{13}C NMR of compound (+)-37 ($CDCl_3$, 100 MHz, 25 °C)

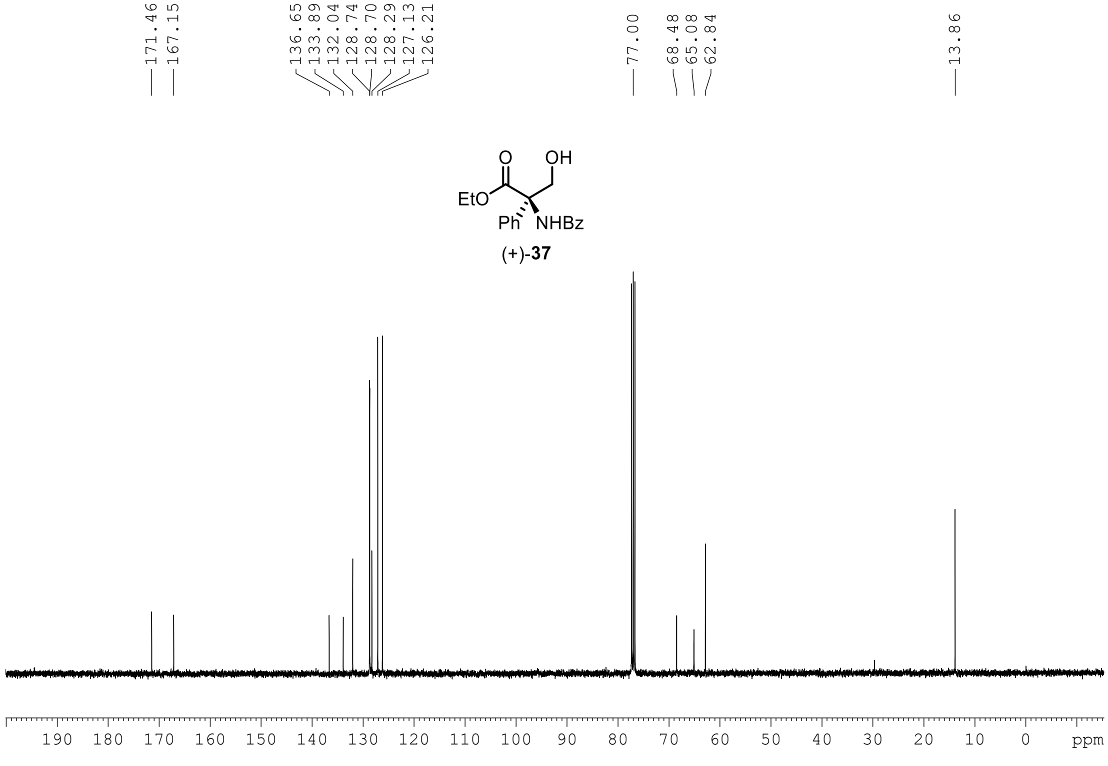

Supplementary Fig. 179. ¹H NMR of compound (+)-38 ($CDCl_3$, 400 MHz, 25 °C)

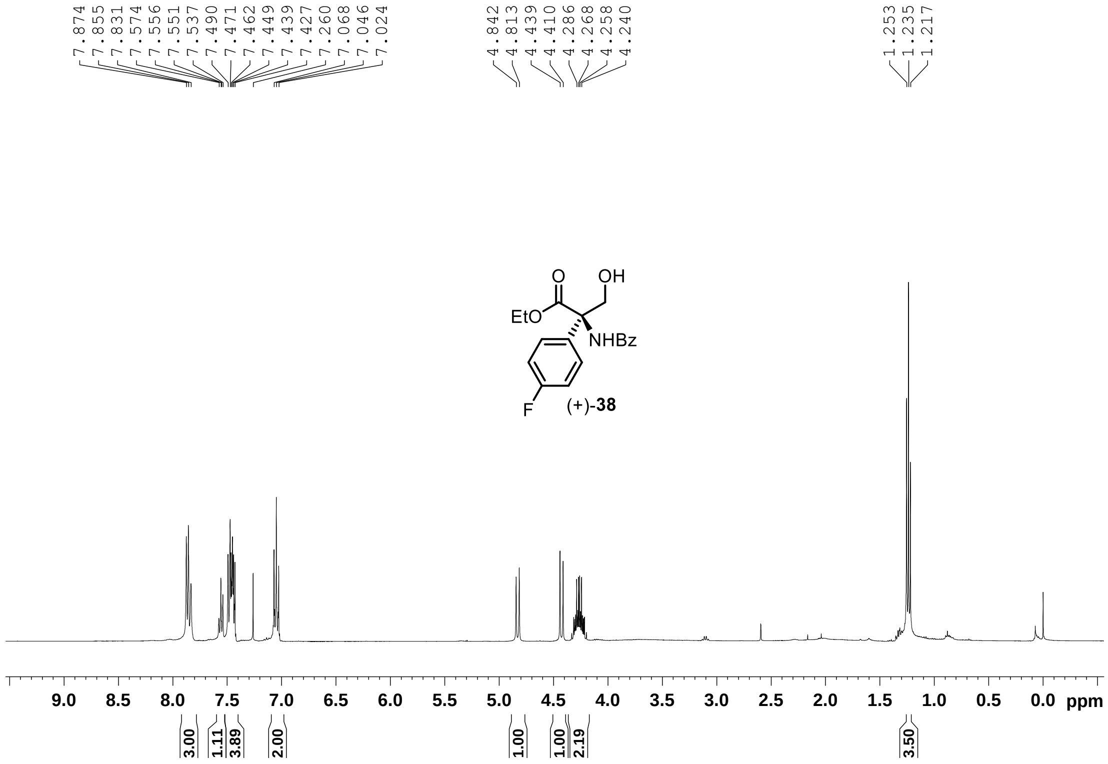

Supplementary Fig. 180. ¹³C NMR of compound (+)-38 ($CDCl_3$, 100 MHz, 25 °C)

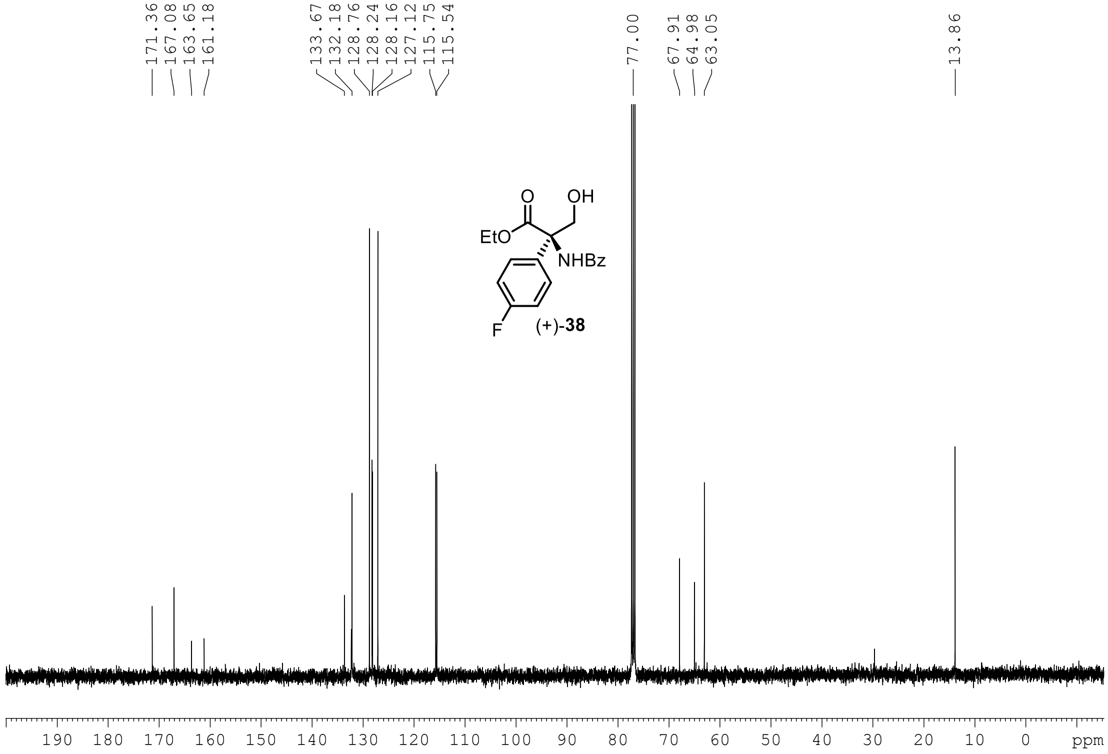

Supplementary Fig. 181. ¹H NMR of compound (+)-39 ($CDCl_3$, 400 MHz, 25 °C)

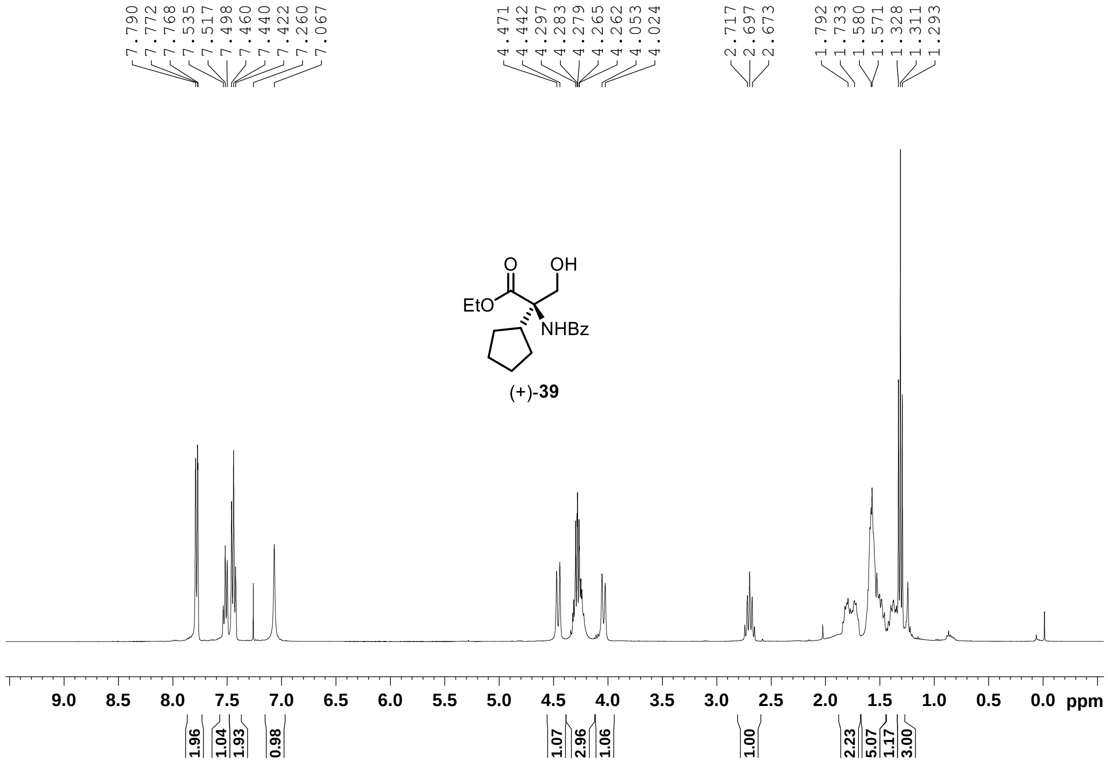

Supplementary Fig. 182. ¹³C NMR of compound (+)-39 ($CDCl_3$, 100 MHz, 25 °C)

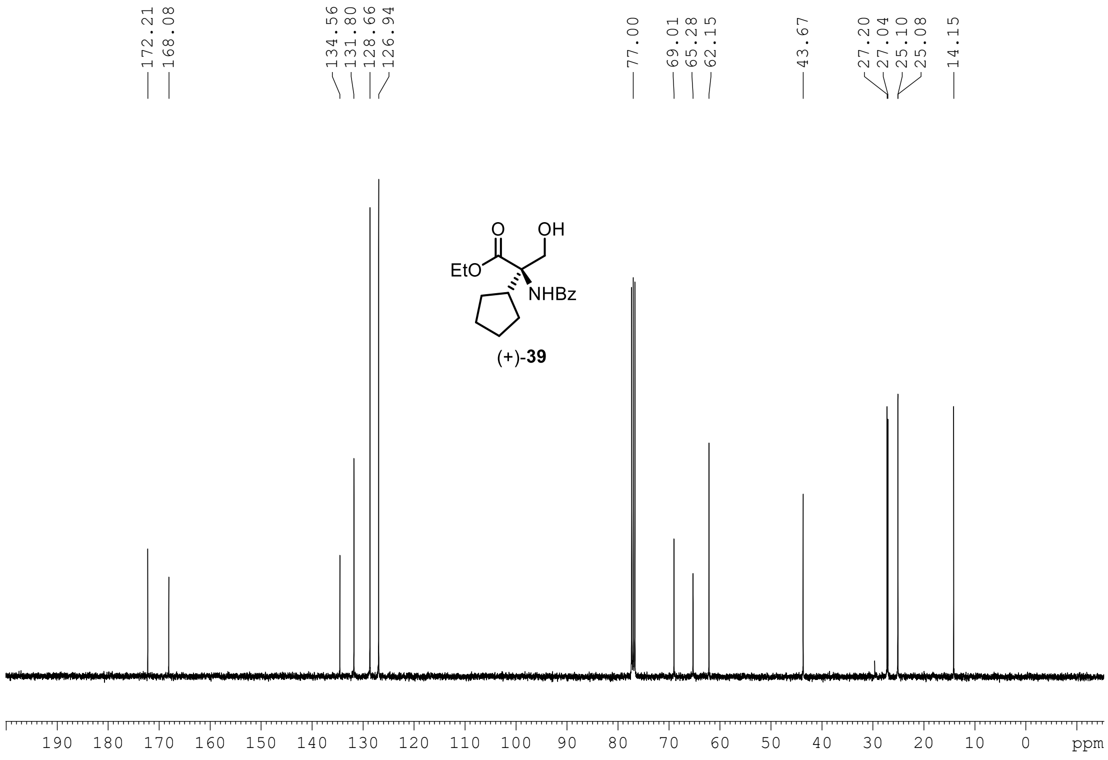

Supplementary Fig. 183. ¹H NMR of compound (+)-10 ($CDCl_3$, 400 MHz, 25 °C)

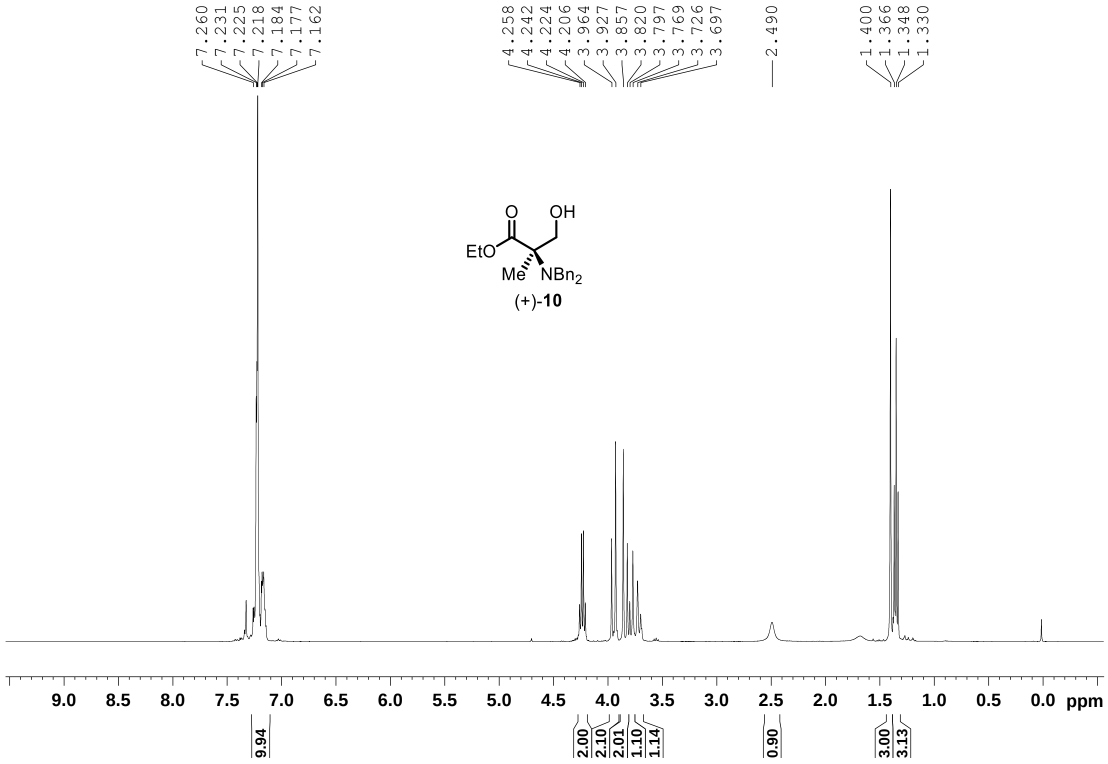

Supplementary Fig. 184. ¹³C NMR of compound (+)-10 ($CDCl_3$, 100 MHz, 25 °C)

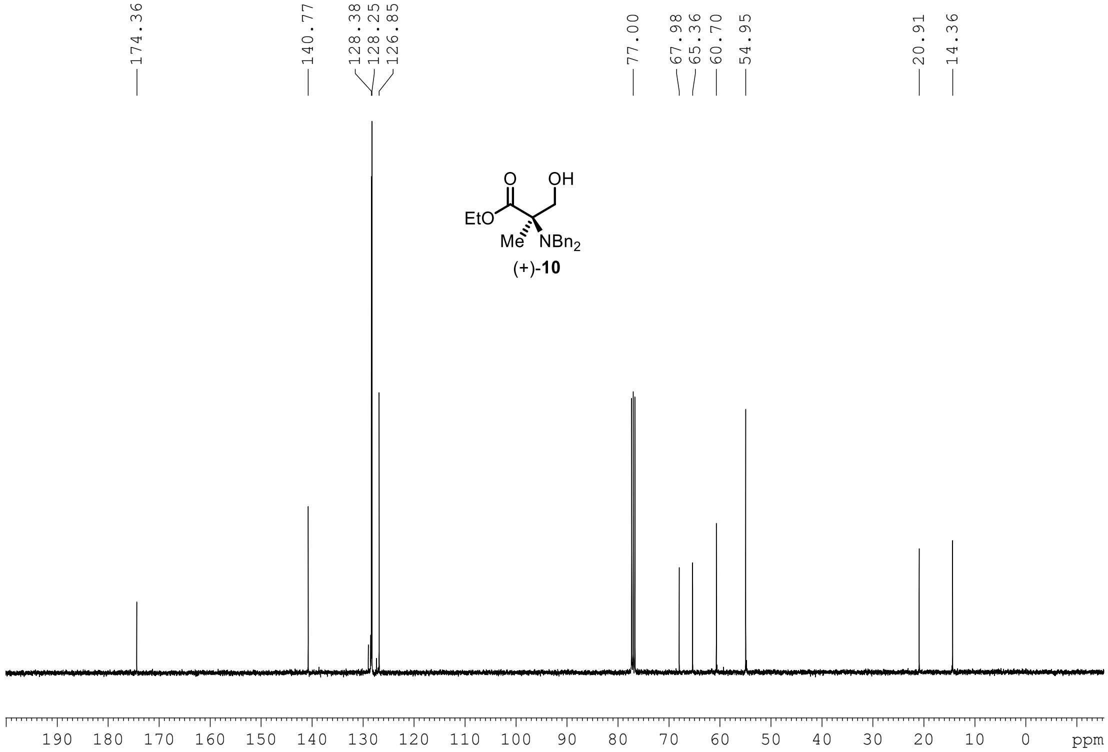

Supplementary Fig. 185. ¹H NMR of compound (+)-40 ($CDCl_3$, 400 MHz, 25 °C)

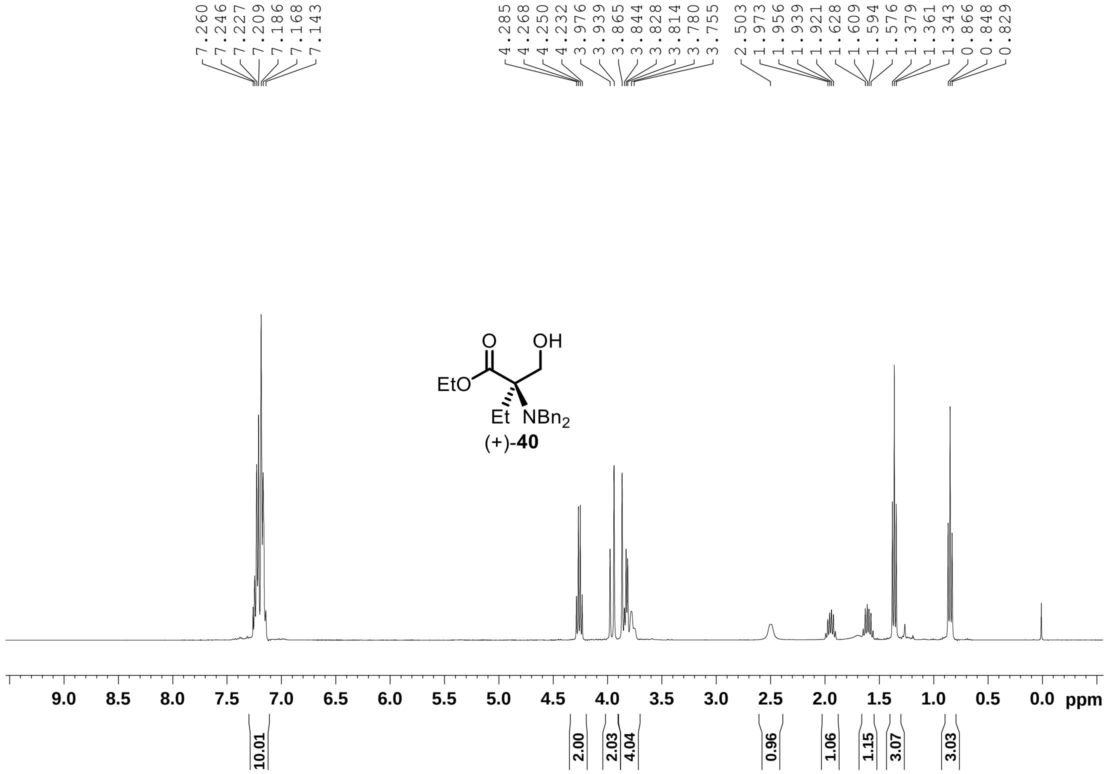

Supplementary Fig. 186. ¹³C NMR of compound (+)-40 ($CDCl_3$, 100 MHz, 25 °C)

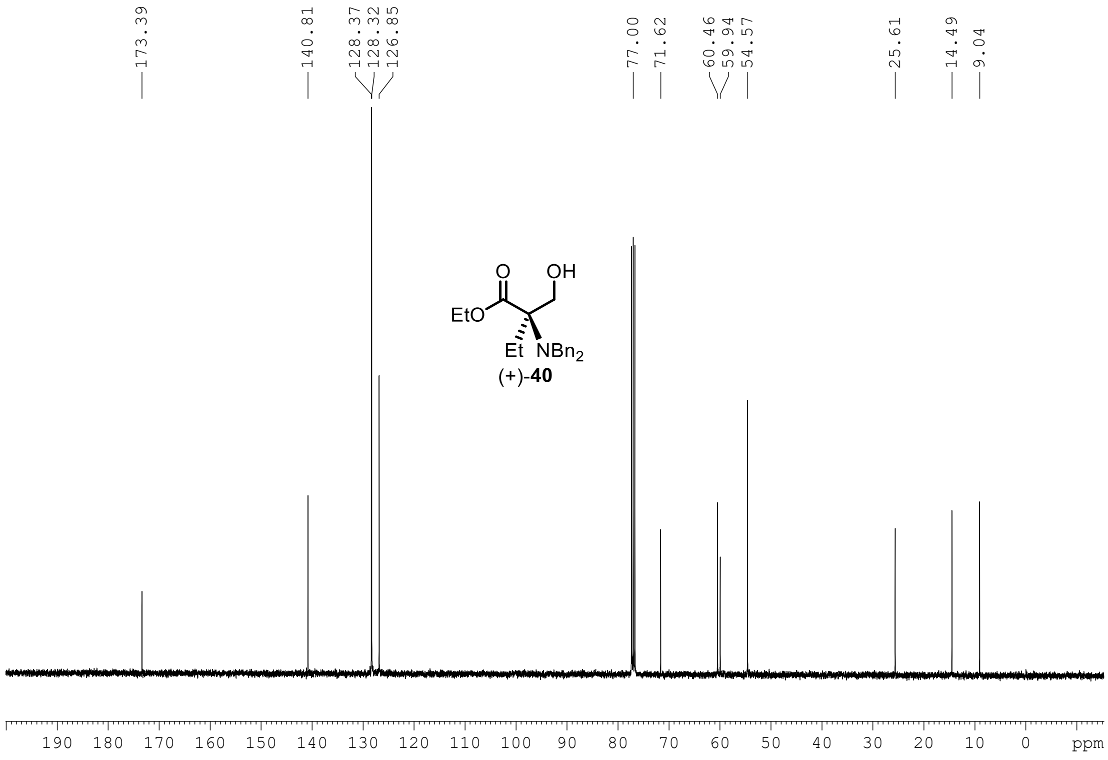

Supplementary Fig. 187. ^{1}H NMR of compound (+)-41 ($CDCl_3$, 400 MHz, 25 °C)

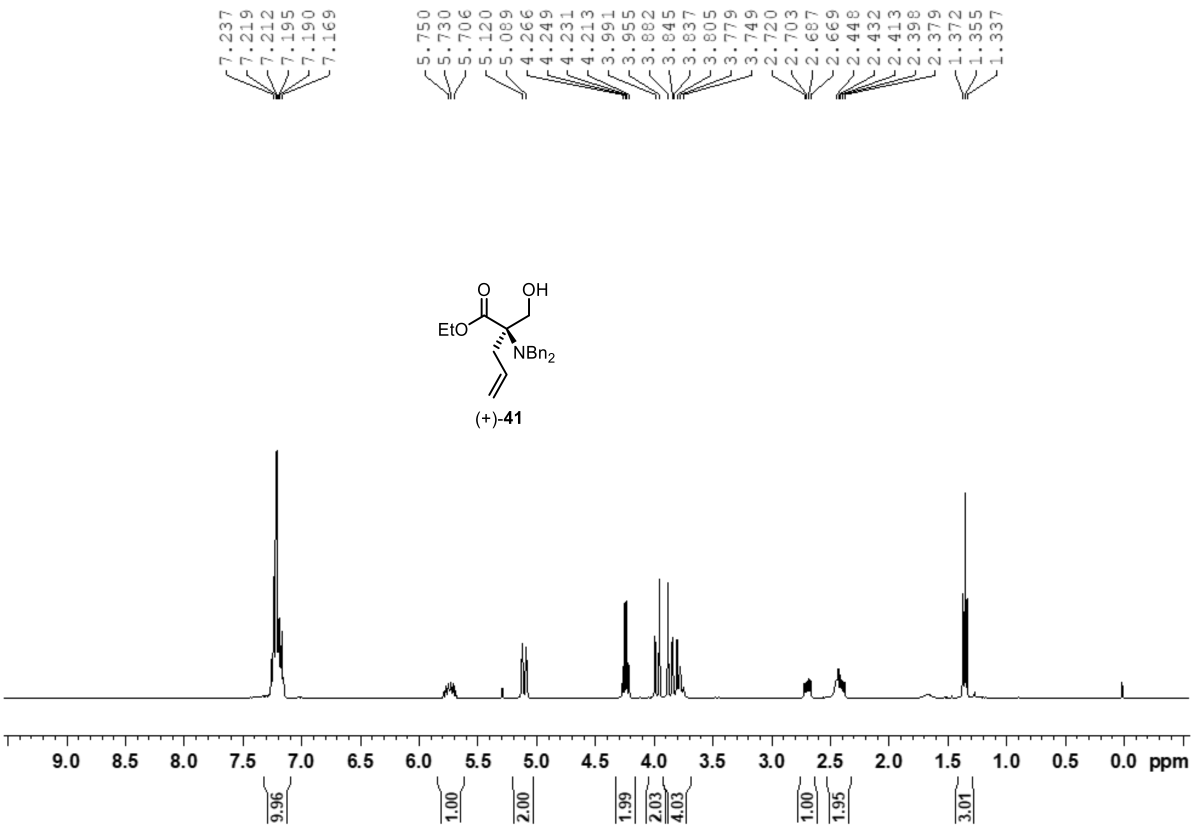

Supplementary Fig. 188. ^{13}C NMR of compound (+)-41 ($CDCl_3$, 100 MHz, 25 °C)

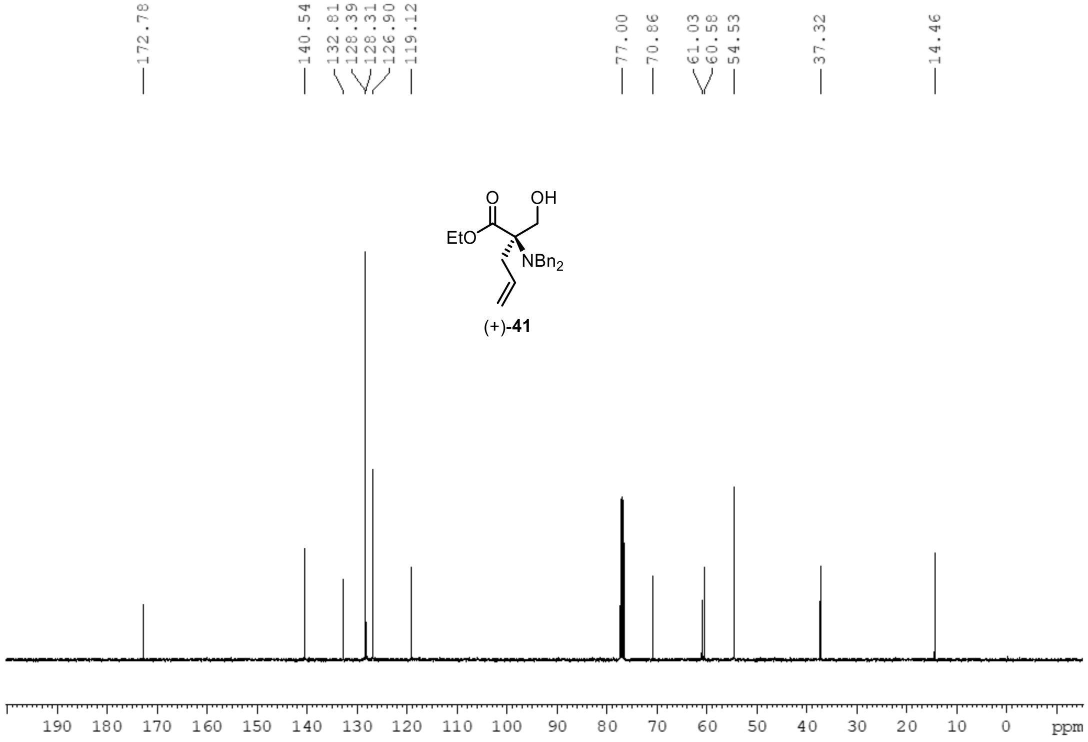

Supplementary Fig. 189. ^{1}H NMR of compound (+)-42 ($CDCl_3$, 400 MHz, 25 °C)

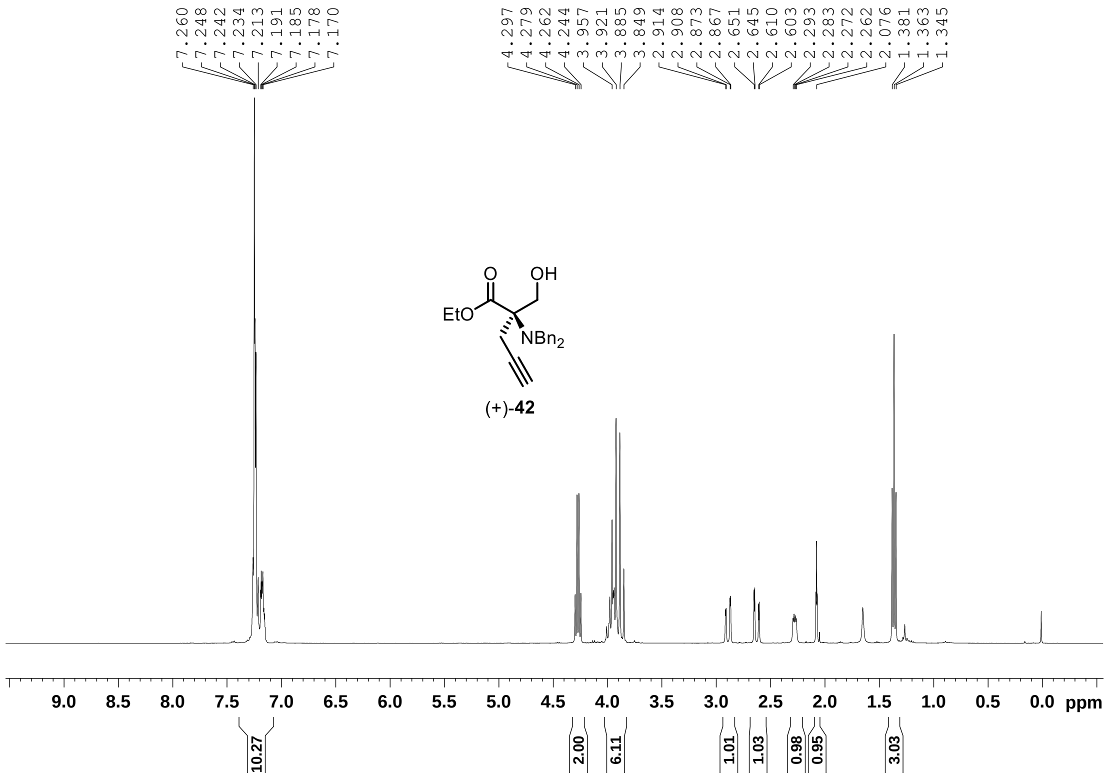

Supplementary Fig. 190. ^{13}C NMR of compound (+)-42 ($CDCl_3$, 100 MHz, 25 °C)

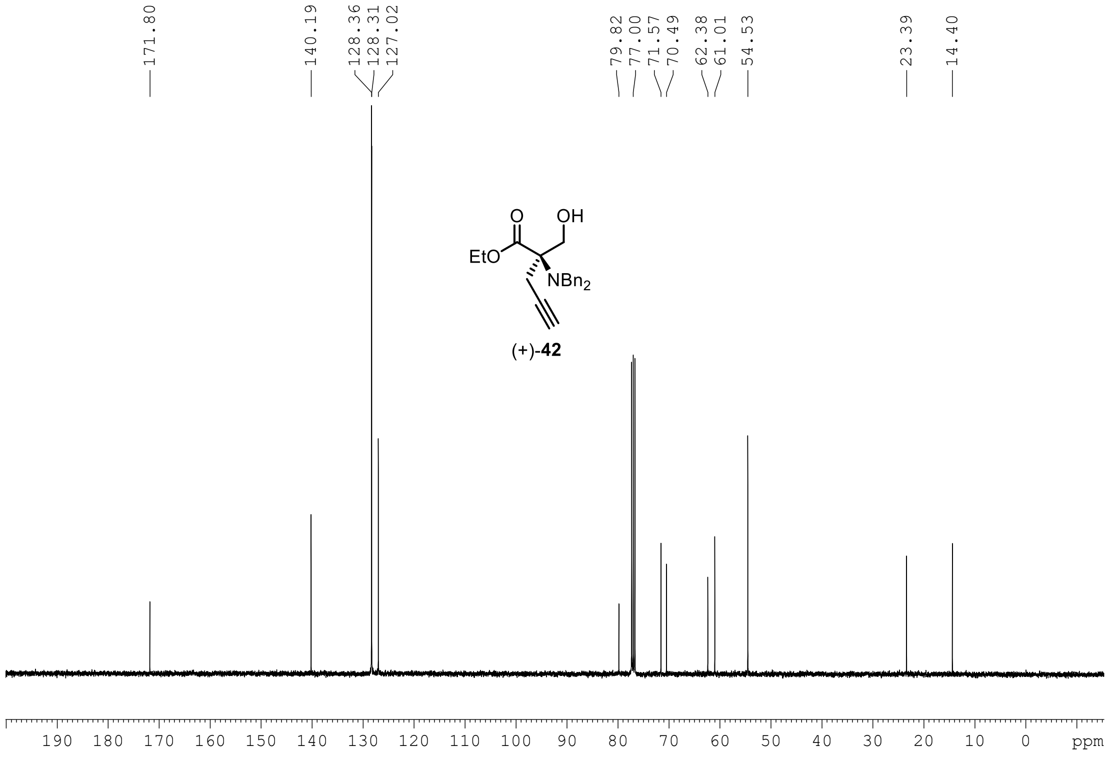

Supplementary Fig. 191. ^{1}H NMR of compound (+)-44 (CDCl$_3$, 400 MHz, 25 °C)

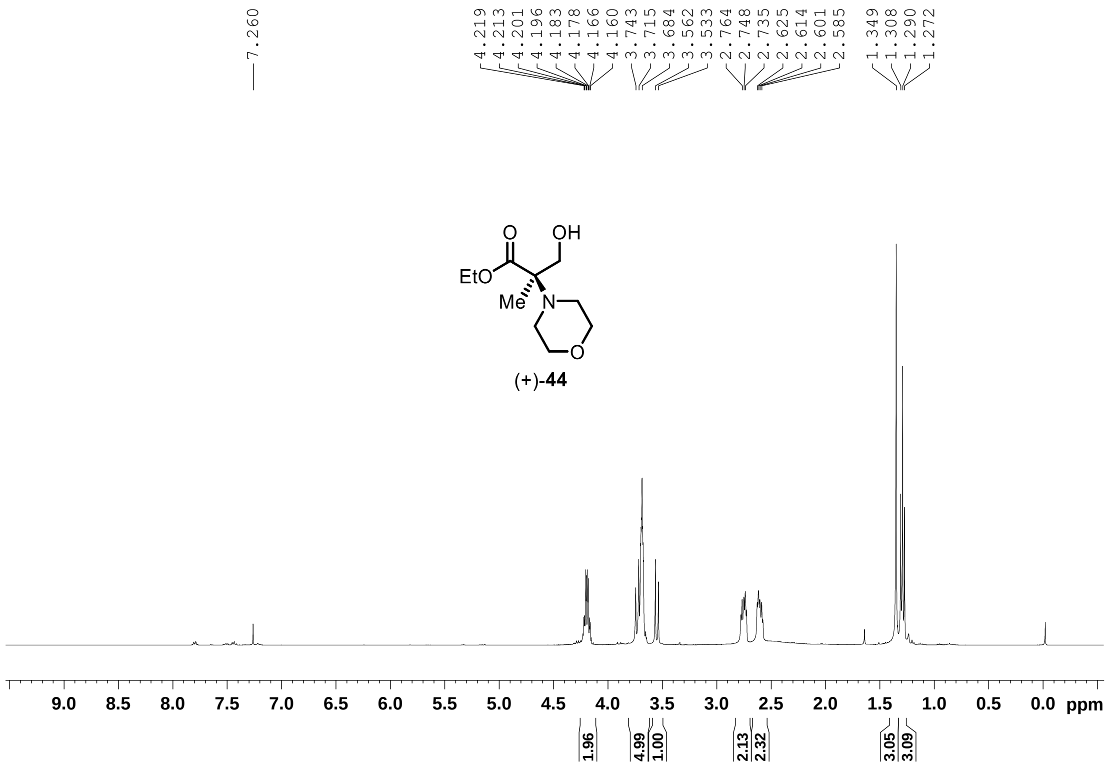

Supplementary Fig. 192. ^{13}C NMR of compound (+)-44 (CDCl$_3$, 100 MHz, 25 °C)

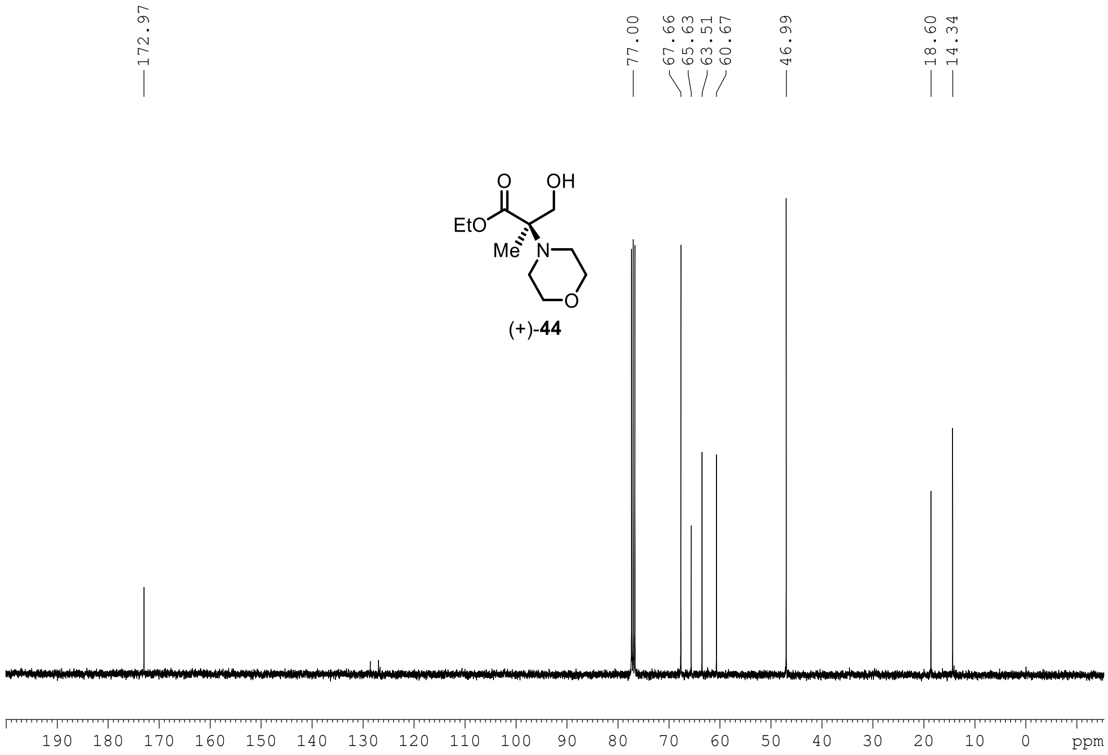

Supplementary Fig. 193. ^{1}H NMR of compound (+)-45 ($CDCl_3$, 400 MHz, 25 °C)

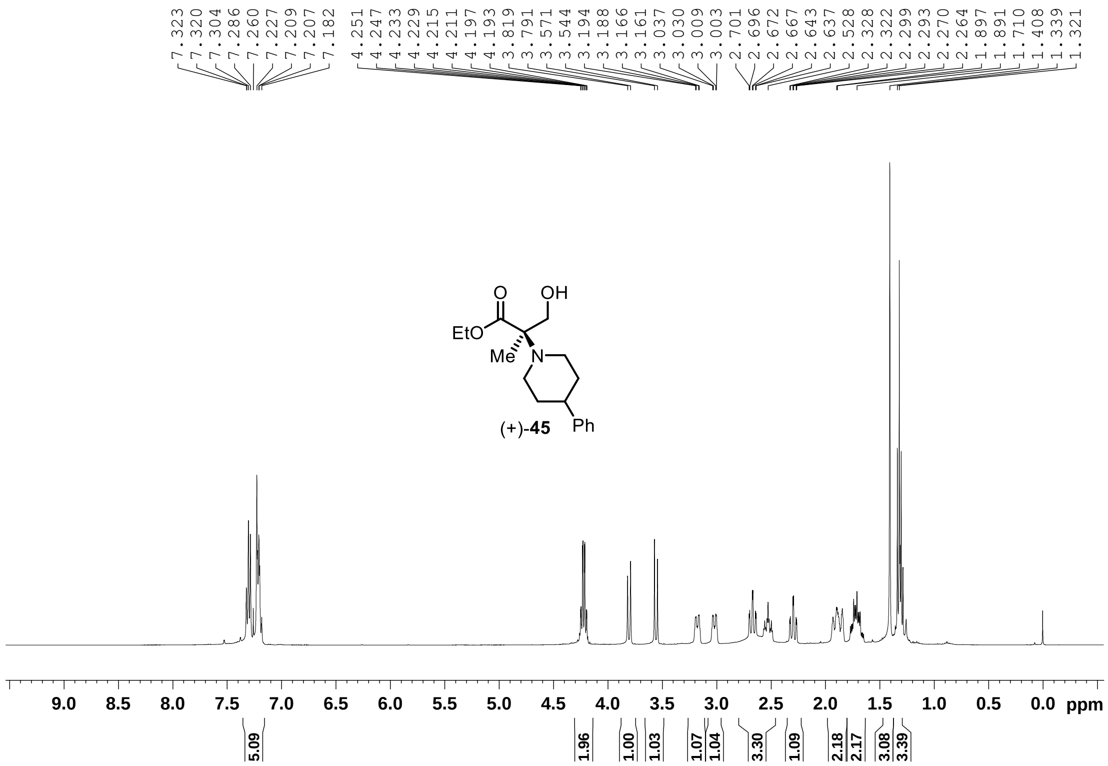

Supplementary Fig. 194. ^{13}C NMR of compound (+)-45 ($CDCl_3$, 100 MHz, 25 °C)

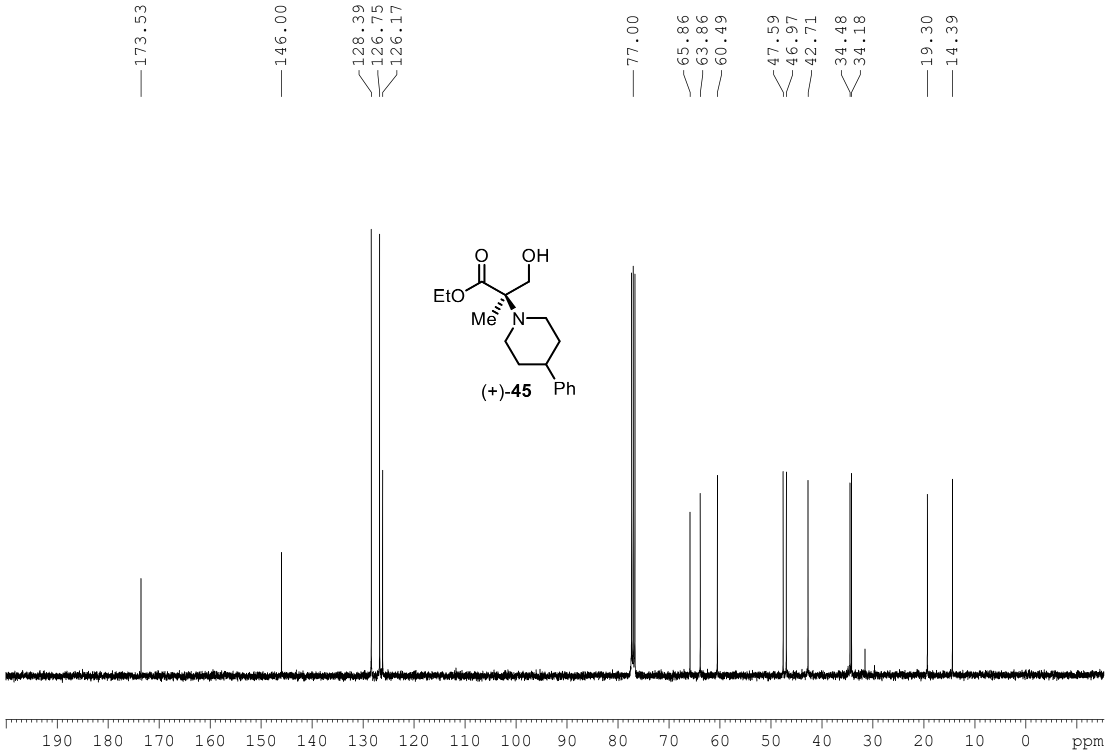

Supplementary Fig. 195. ^{1}H NMR of compound (−)-46 ($CDCl_3$, 400 MHz, 25 °C)

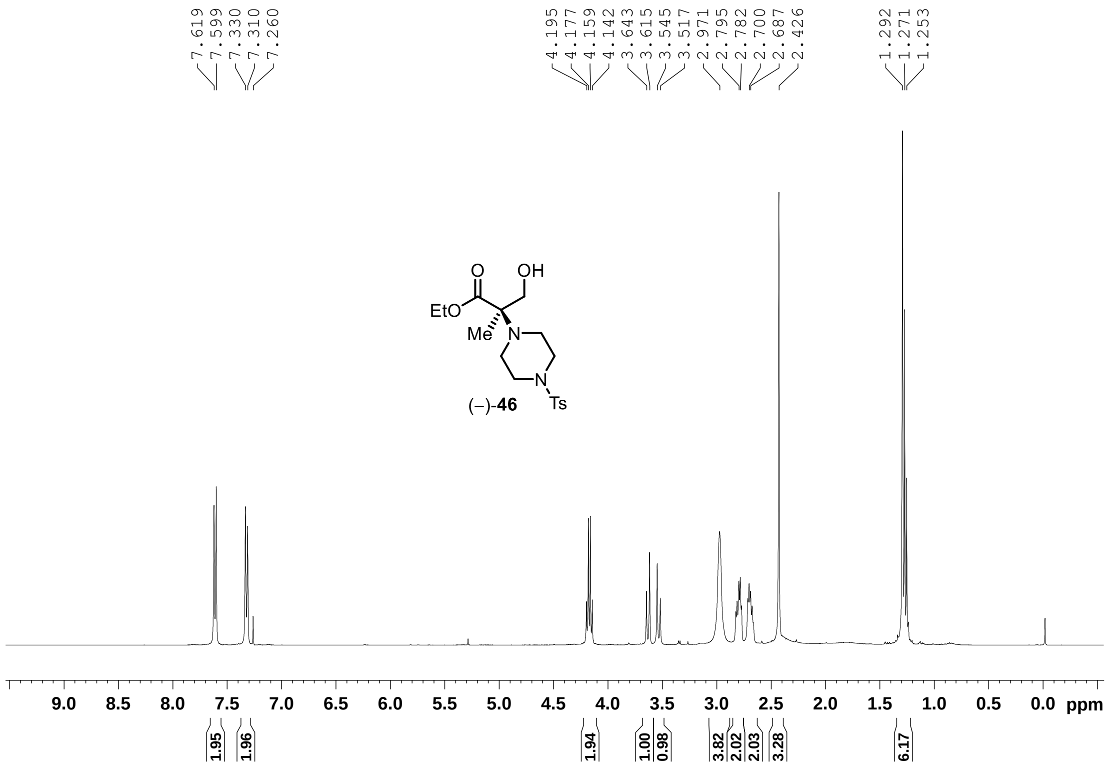

Supplementary Fig. 196. ^{13}C NMR of compound (−)-46 ($CDCl_3$, 100 MHz, 25 °C)

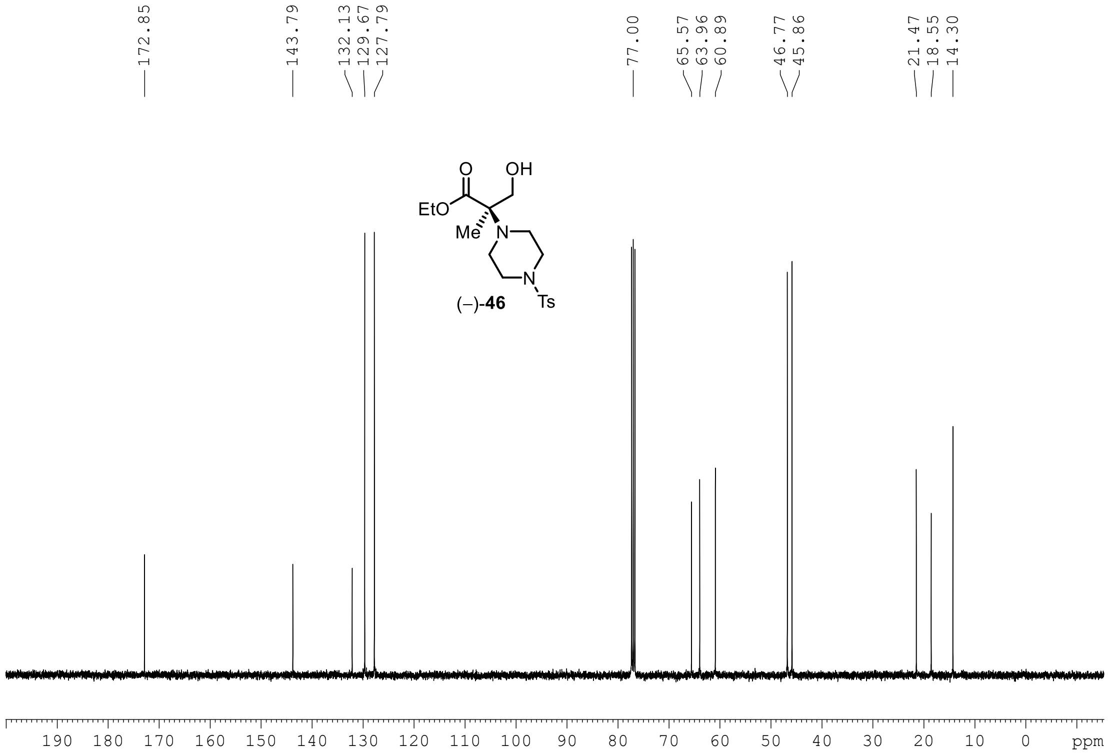

Supplementary Fig. 197. ^{1}H NMR of compound (+)-11 ($CDCl_3$, 400 MHz, 25 °C)

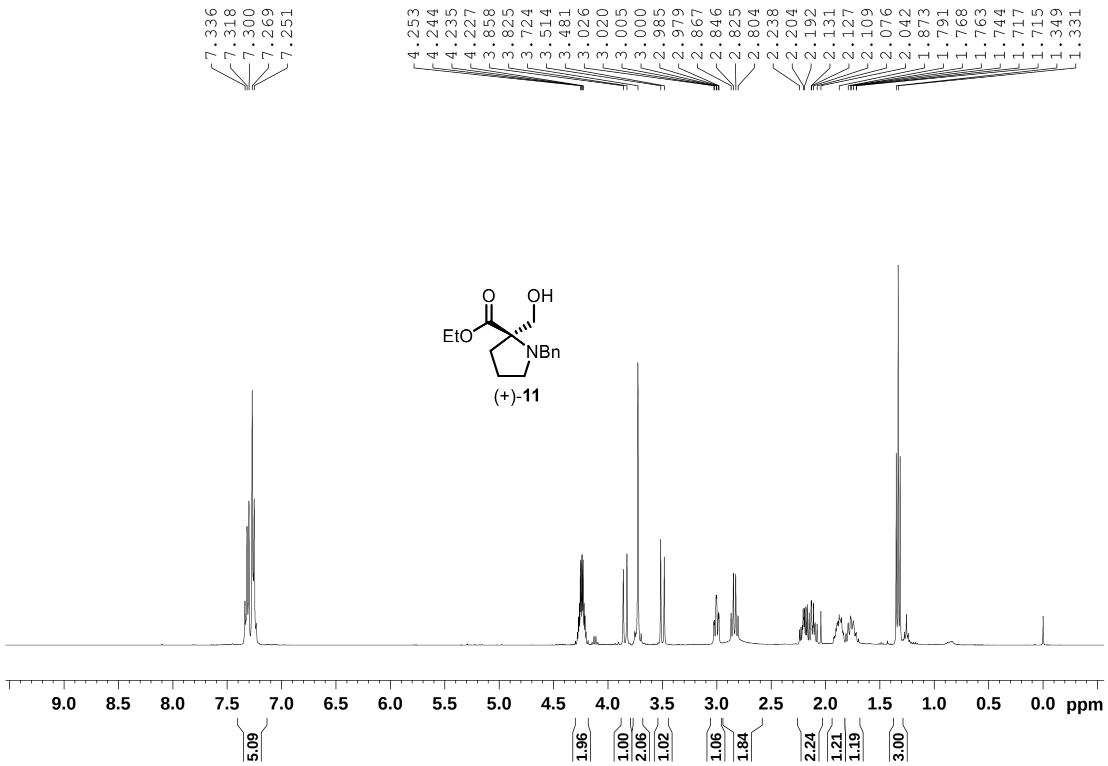

Supplementary Fig. 198. ^{13}C NMR of compound (+)-11 ($CDCl_3$, 100 MHz, 25 °C)

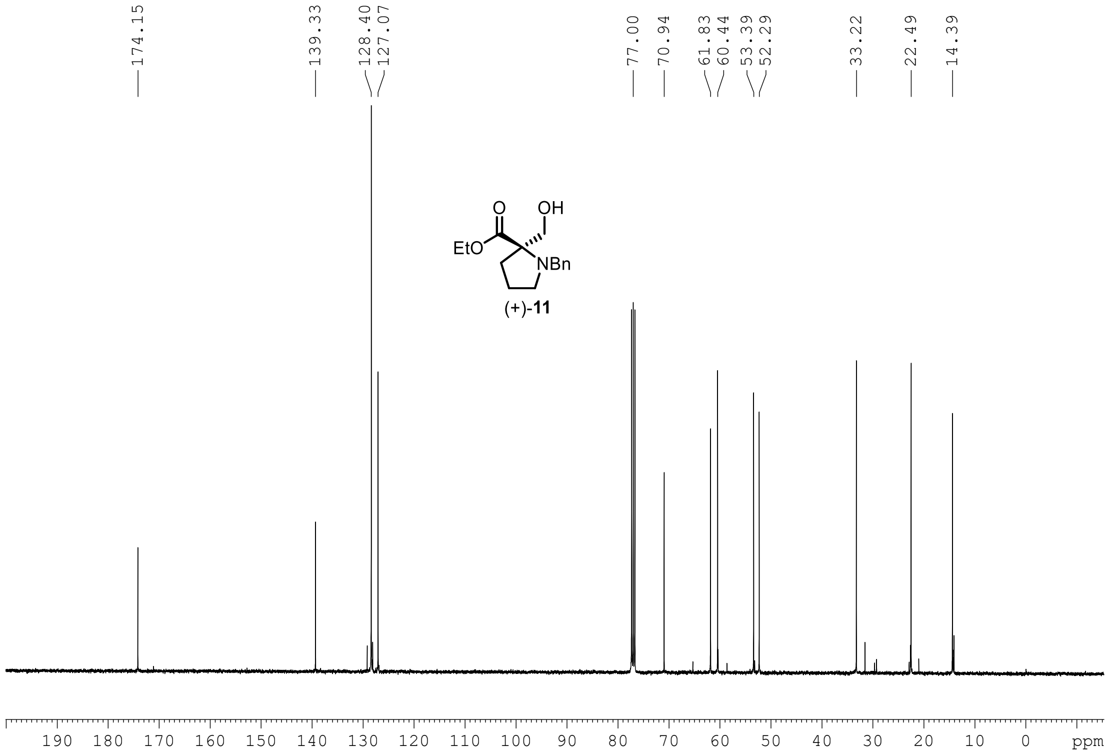

Supplementary Fig. 199. ^{1}H NMR of compound (+)-47 ($CDCl_3$, 400 MHz, 25 °C)

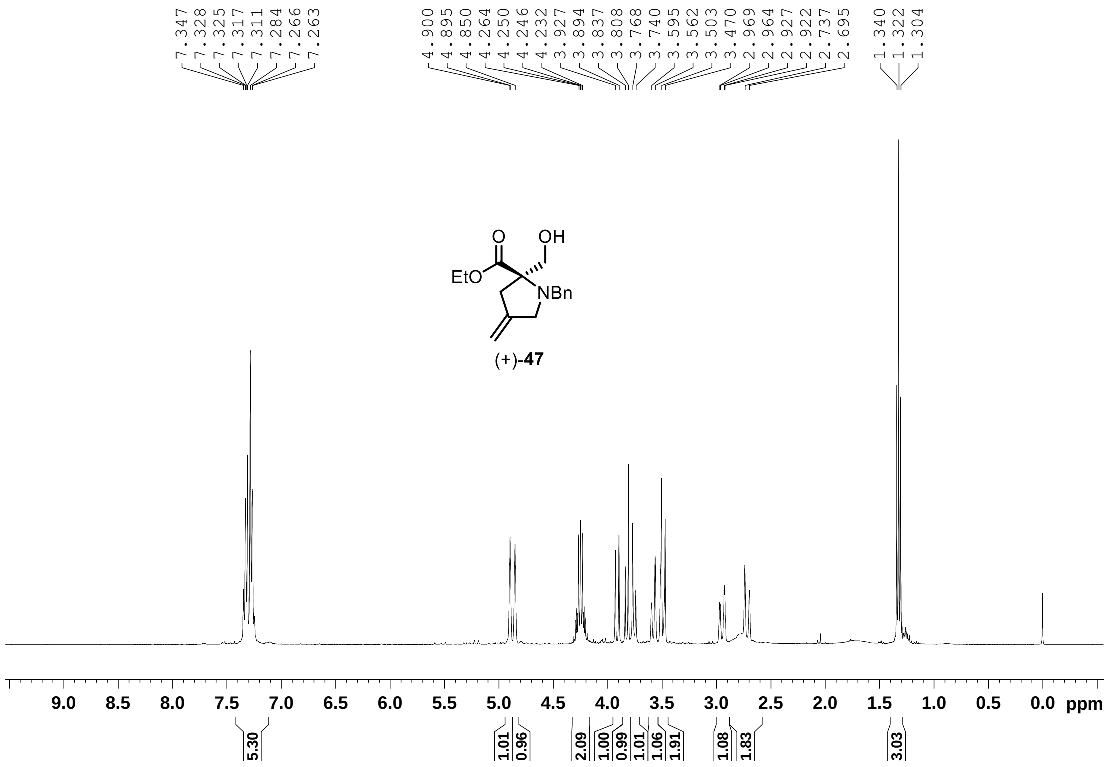

Supplementary Fig. 200. ^{13}C NMR of compound (+)-47 ($CDCl_3$, 100 MHz, 25 °C)

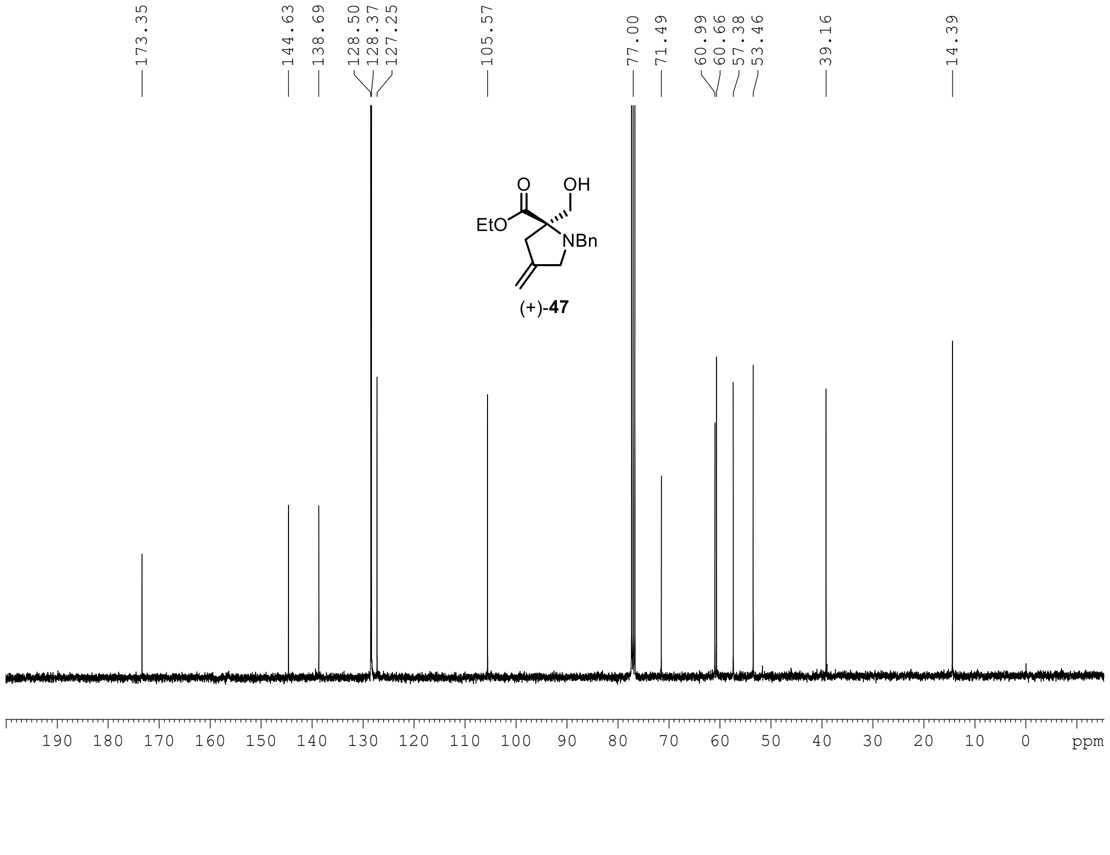

Supplementary Fig. 201. ^{1}H NMR of compound (+)-48 ($CDCl_3$, 400 MHz, 25 °C)

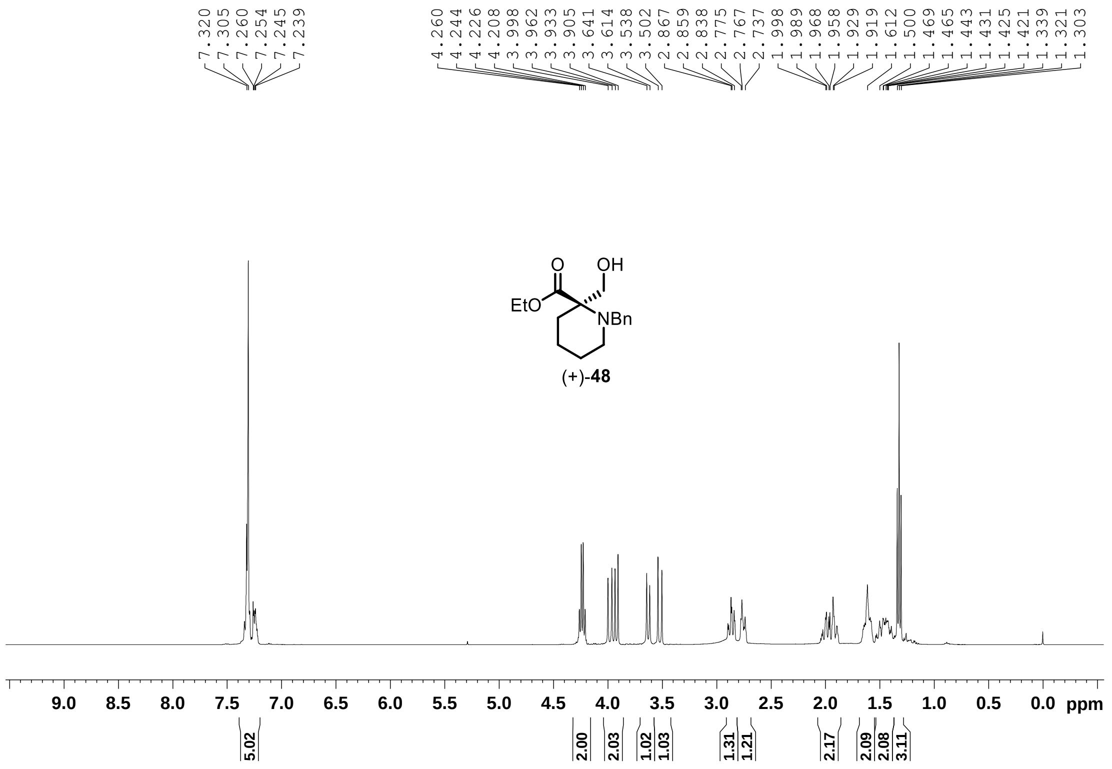

Supplementary Fig. 202. ^{13}C NMR of compound (+)-48 ($CDCl_3$, 100 MHz, 25 °C)

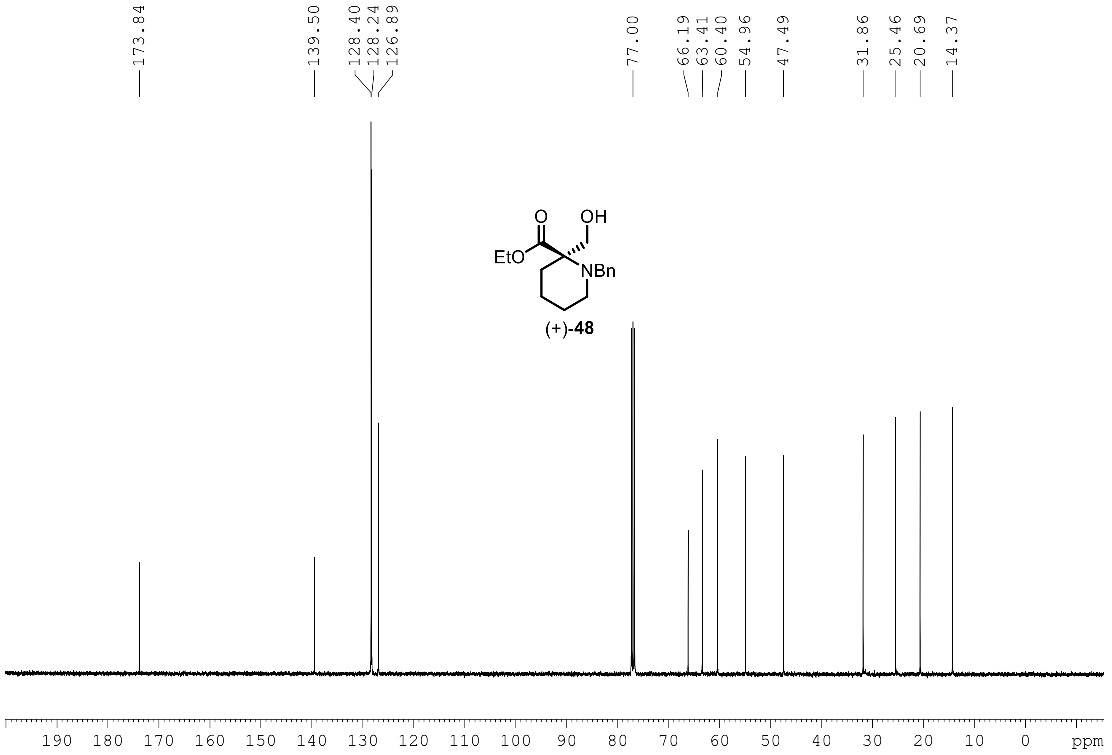

Supplementary Fig. 203. ^{1}H NMR of compound (-)-49 ($CDCl_3$, 400 MHz, 25 °C)

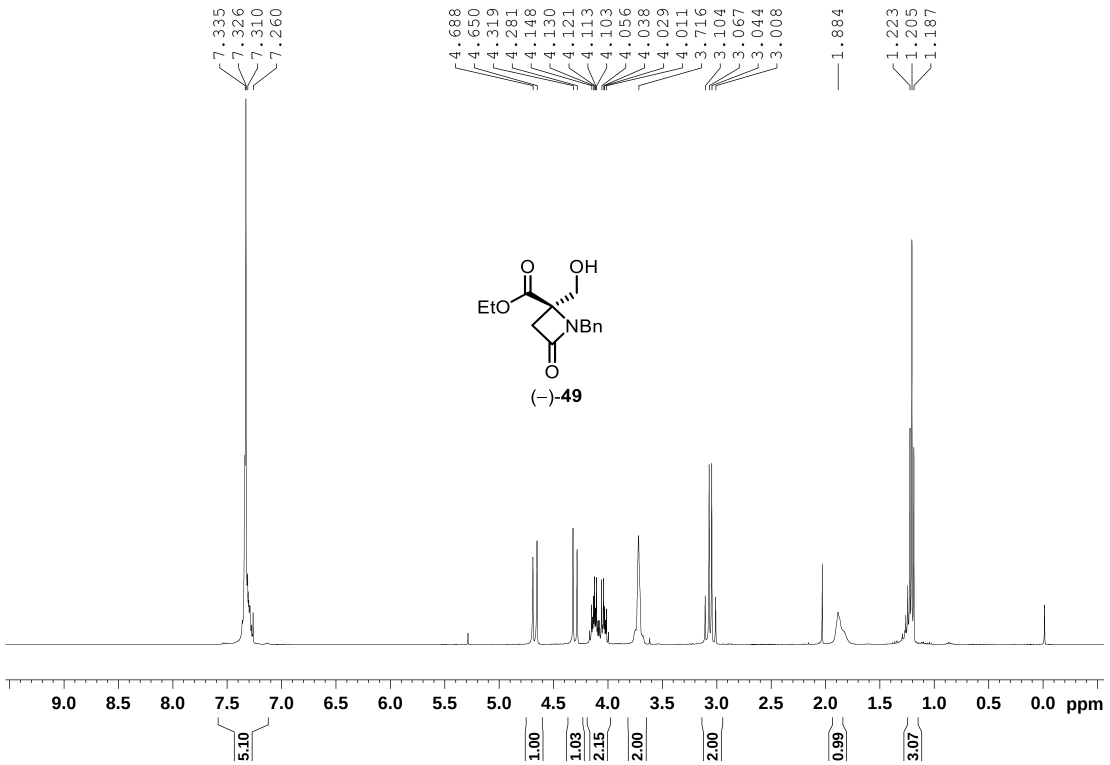

Supplementary Fig. 204. ^{13}C NMR of compound (-)-49 ($CDCl_3$, 100 MHz, 25 °C)

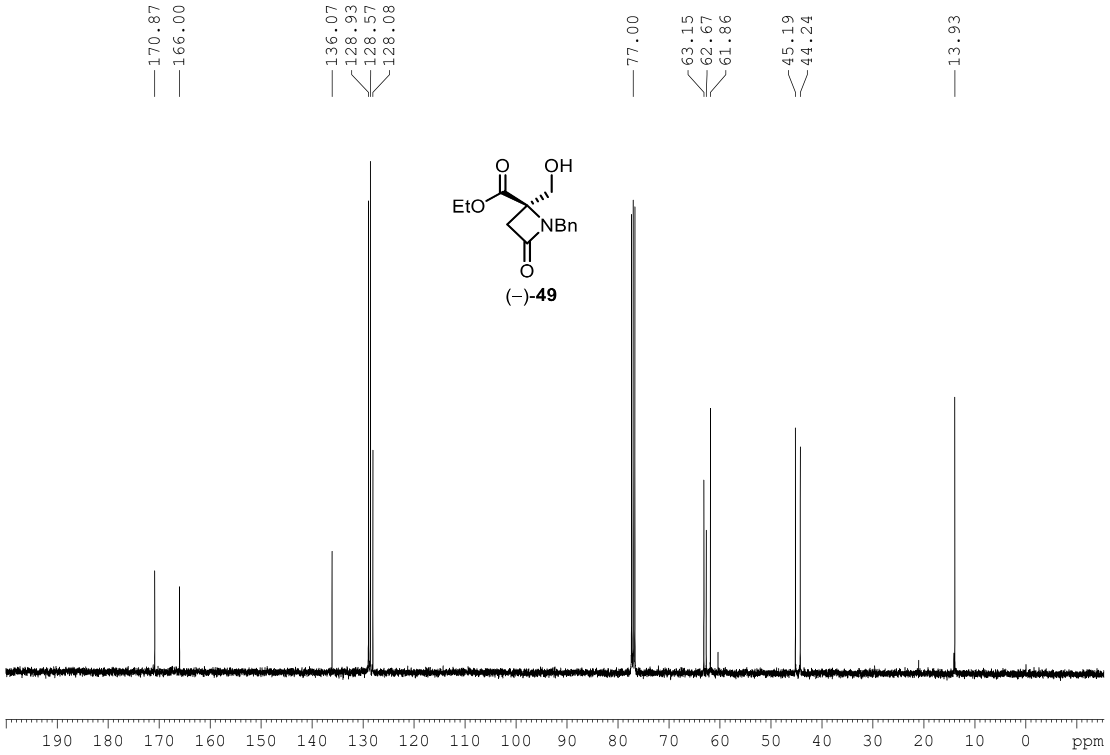

Supplementary Fig. 205. ^{1}H NMR of compound (+)-50 ($CDCl_3$, 400 MHz, 25 °C)

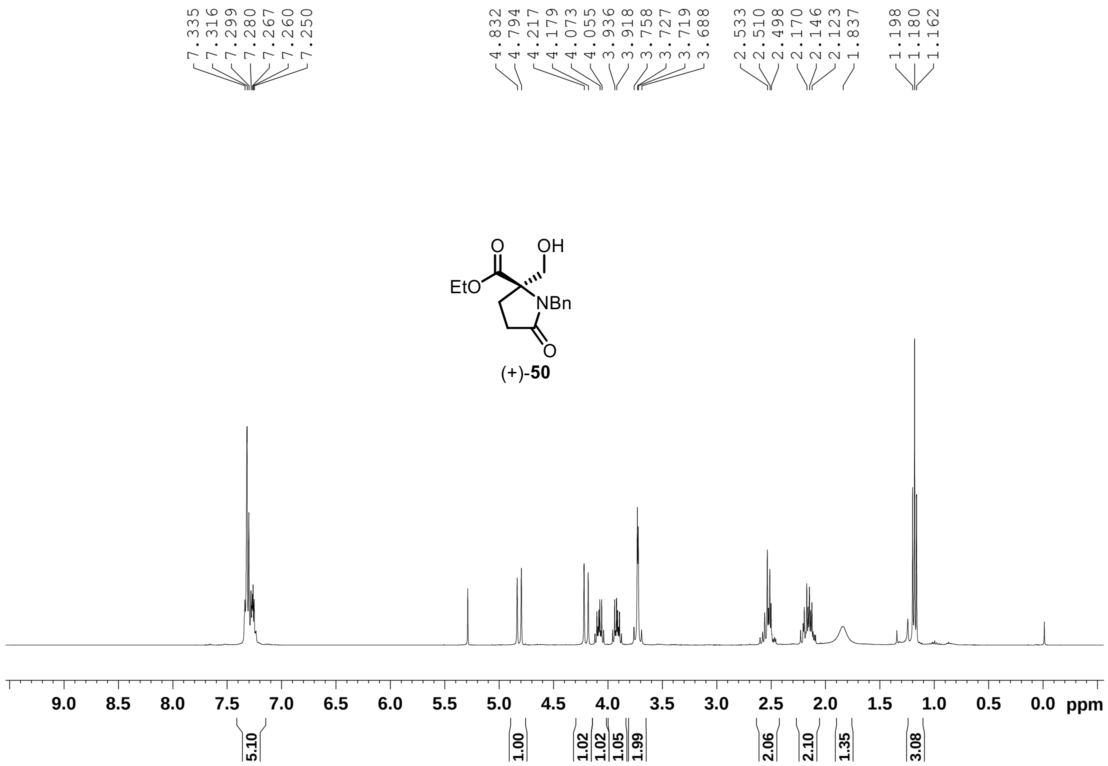

Supplementary Fig. 206. ^{13}C NMR of compound (+)-50 ($CDCl_3$, 100 MHz, 25 °C)

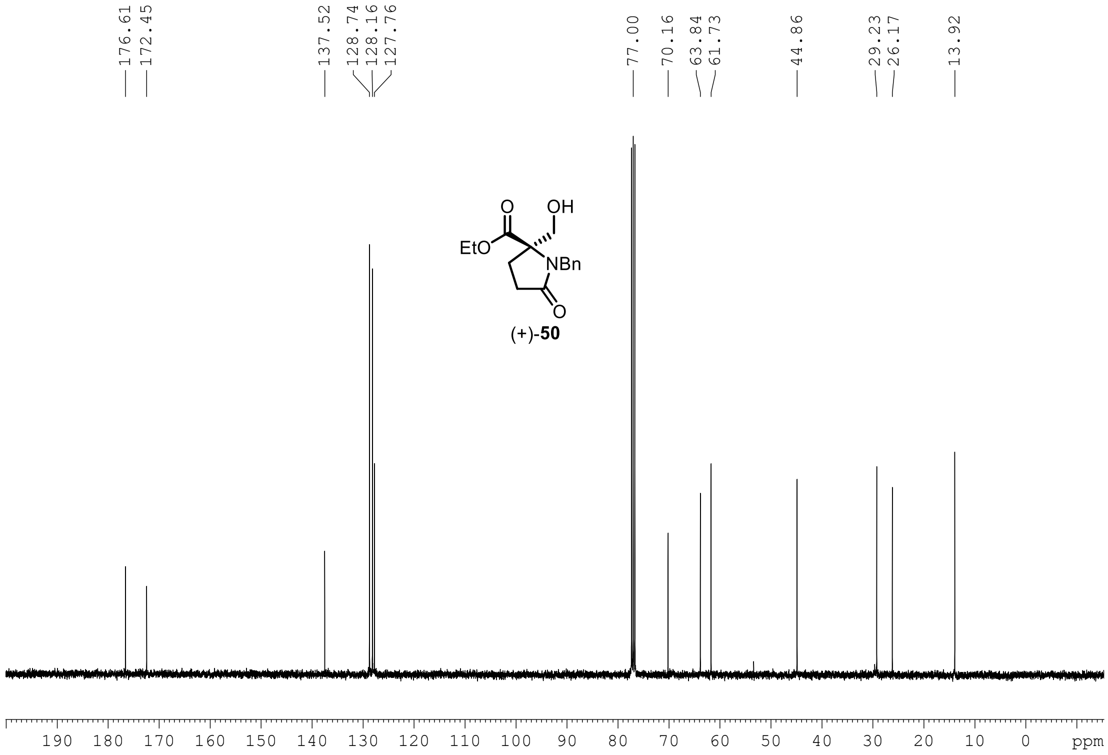

Supplementary Fig. 207. ^{1}H NMR of compound (+)-51 ($CDCl_3$, 400 MHz, 25 °C)

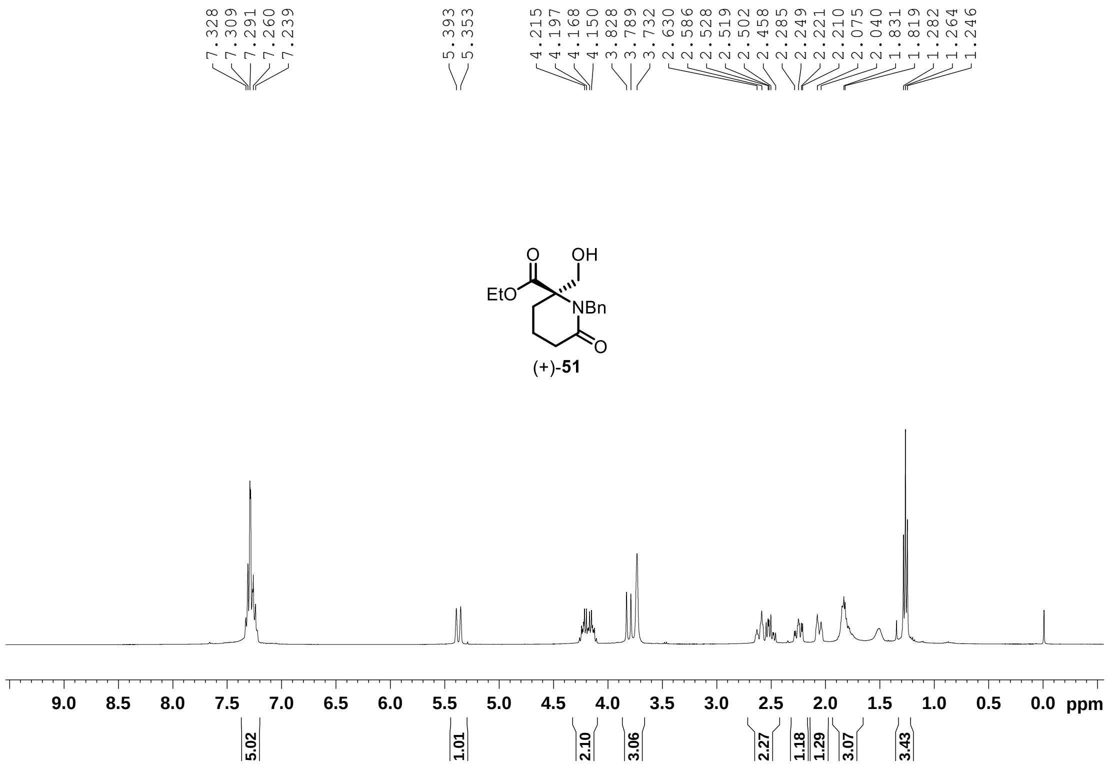

Supplementary Fig. 208. ^{13}C NMR of compound (+)-51 ($CDCl_3$, 100 MHz, 25 °C)

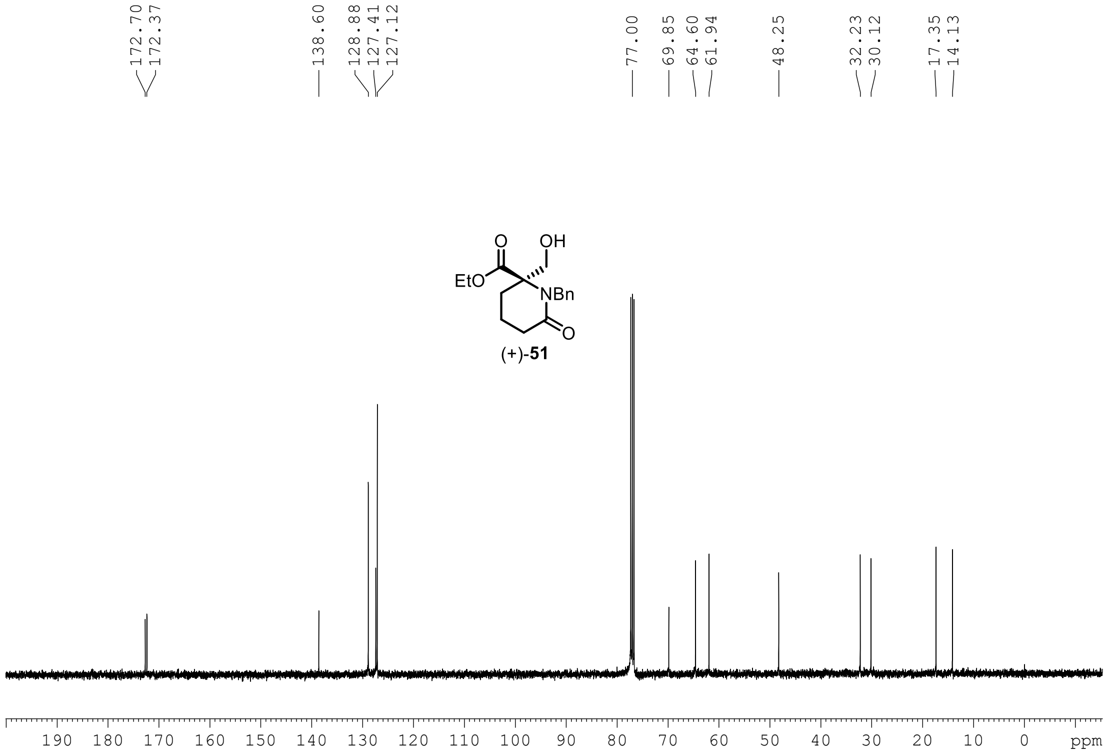

Supplementary Fig. 209. ^{1}H NMR of compound (+)-52 ($CDCl_3$, 400 MHz, 25 °C)

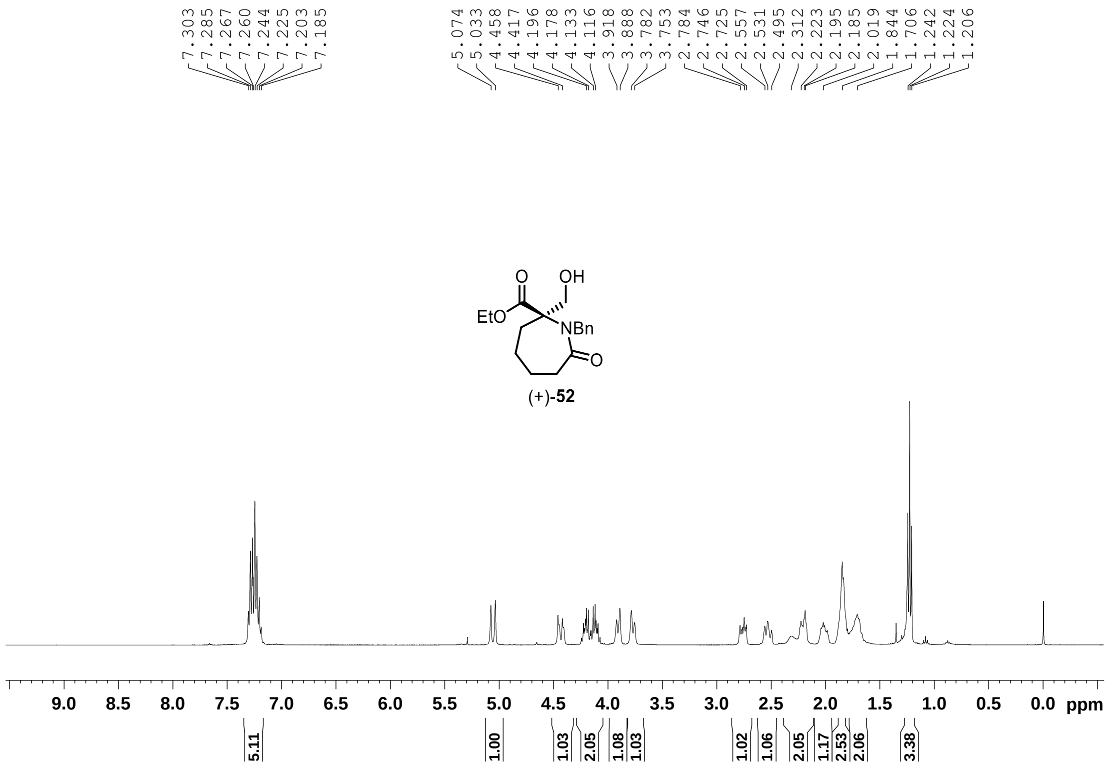

Supplementary Fig. 210. ^{13}C NMR of compound (+)-52 ($CDCl_3$, 100 MHz, 25 °C)

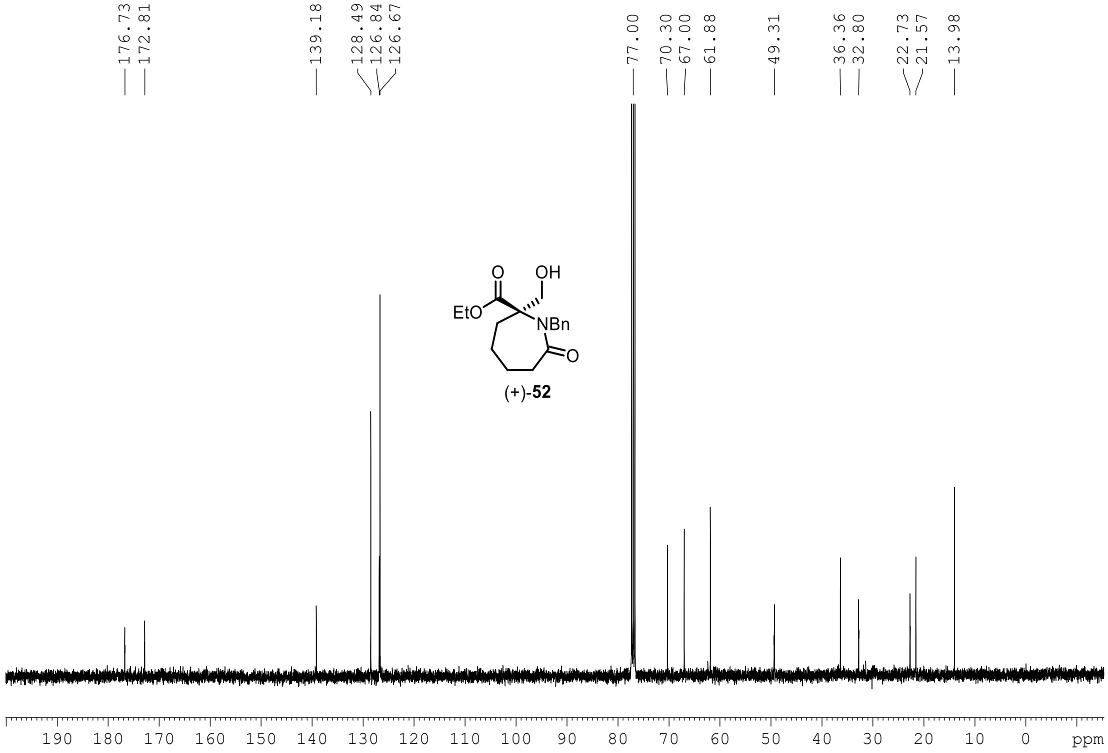

Supplementary Fig. 211. ^{1}H NMR of compound (−)-12 ($CDCl_3$, 400 MHz, 25 °C)

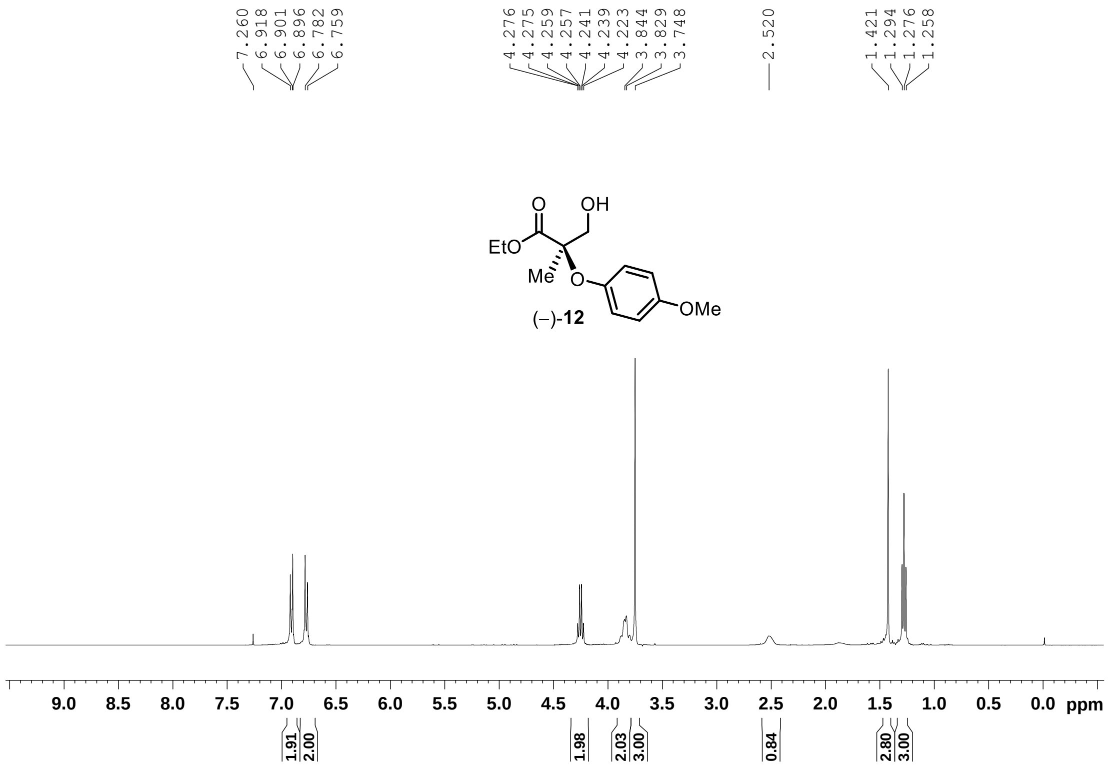

Supplementary Fig. 212. ^{13}C NMR of compound (−)-12 ($CDCl_3$, 100 MHz, 25 °C)

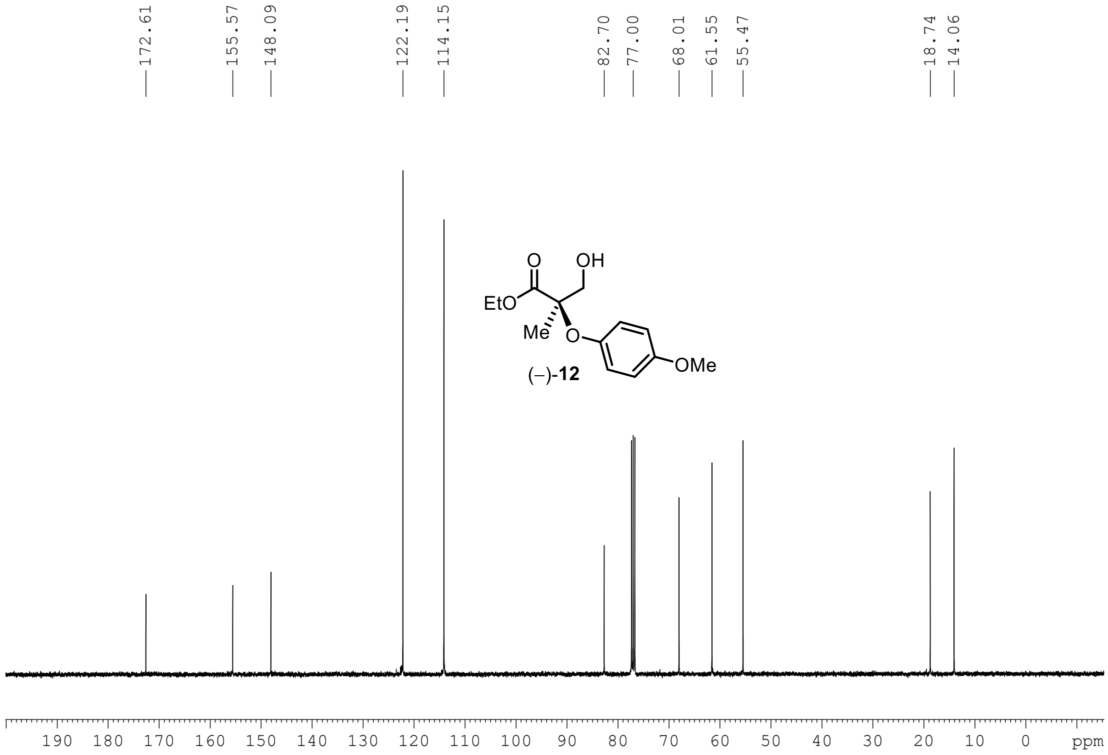

Supplementary Fig. 213. ^{1}H NMR of compound (+)-53 ($CDCl_3$, 400 MHz, 25 °C)

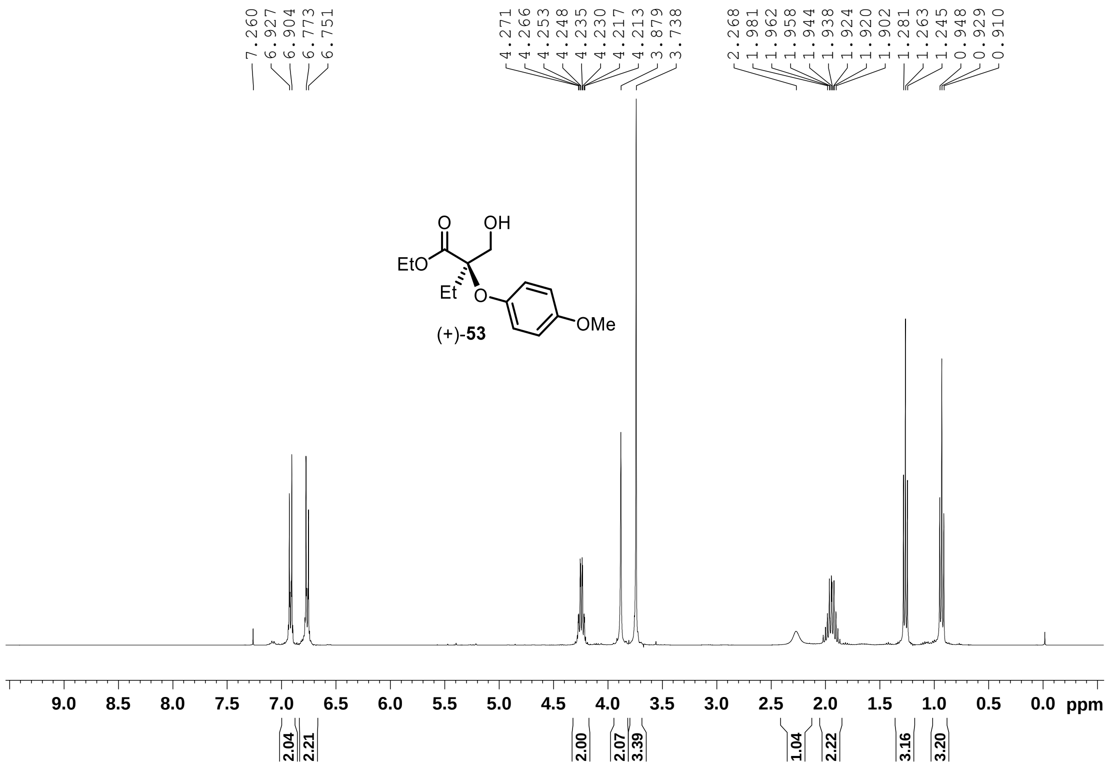

Supplementary Fig. 214. ^{13}C NMR of compound (+)-53 ($CDCl_3$, 100 MHz, 25 °C)

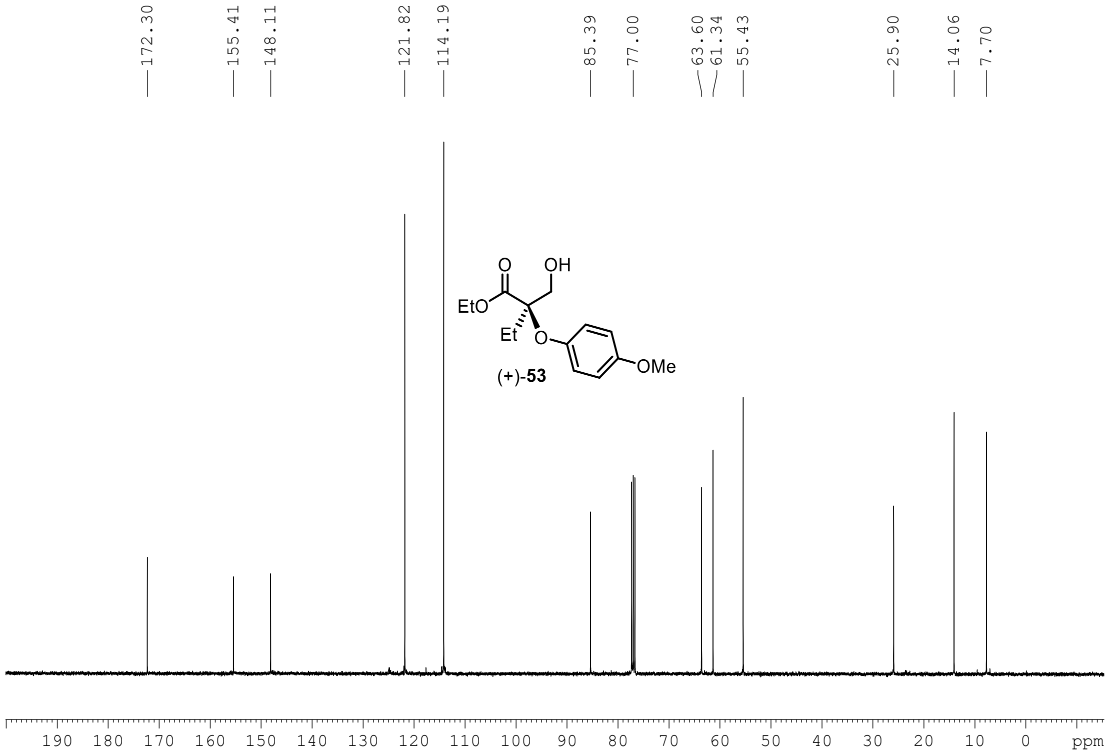

Supplementary Fig. 215. ^{1}H NMR of compound (+)-54 ($CDCl_3$, 400 MHz, 25 °C)

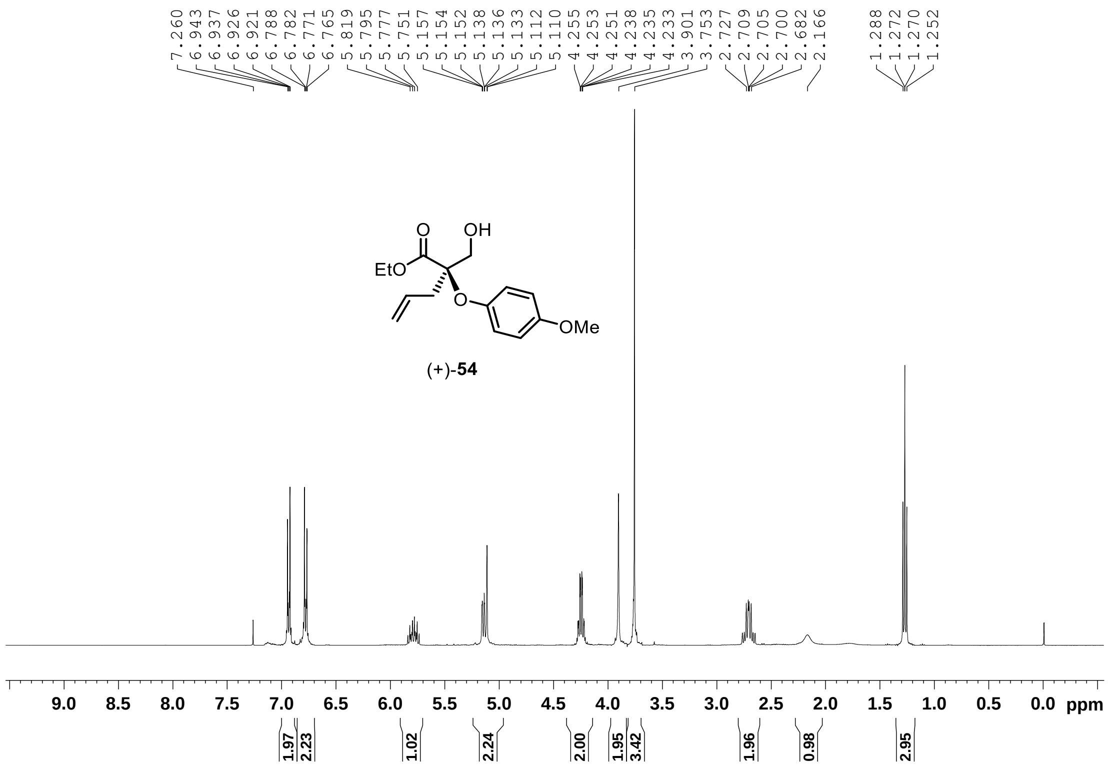

Supplementary Fig. 216. ^{13}C NMR of compound (+)-54 ($CDCl_3$, 100 MHz, 25 °C)

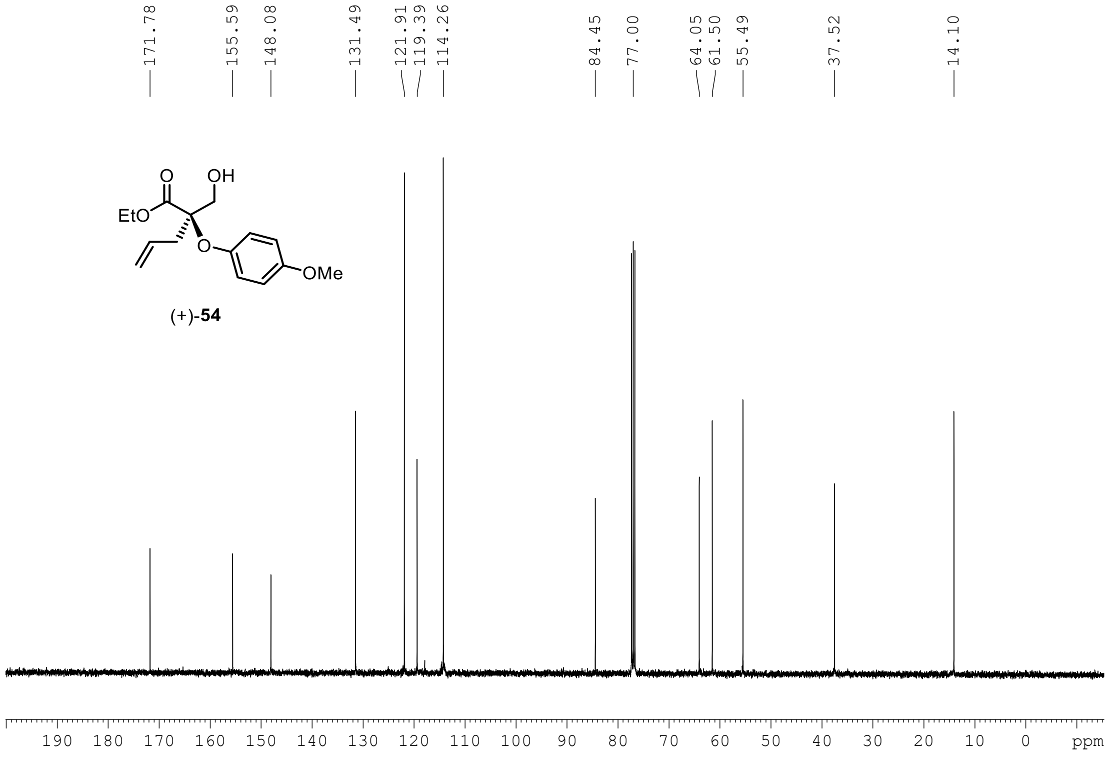

Supplementary Fig. 217. ^{1}H NMR of compound (+)-55 ($CDCl_3$, 400 MHz, 25 °C)

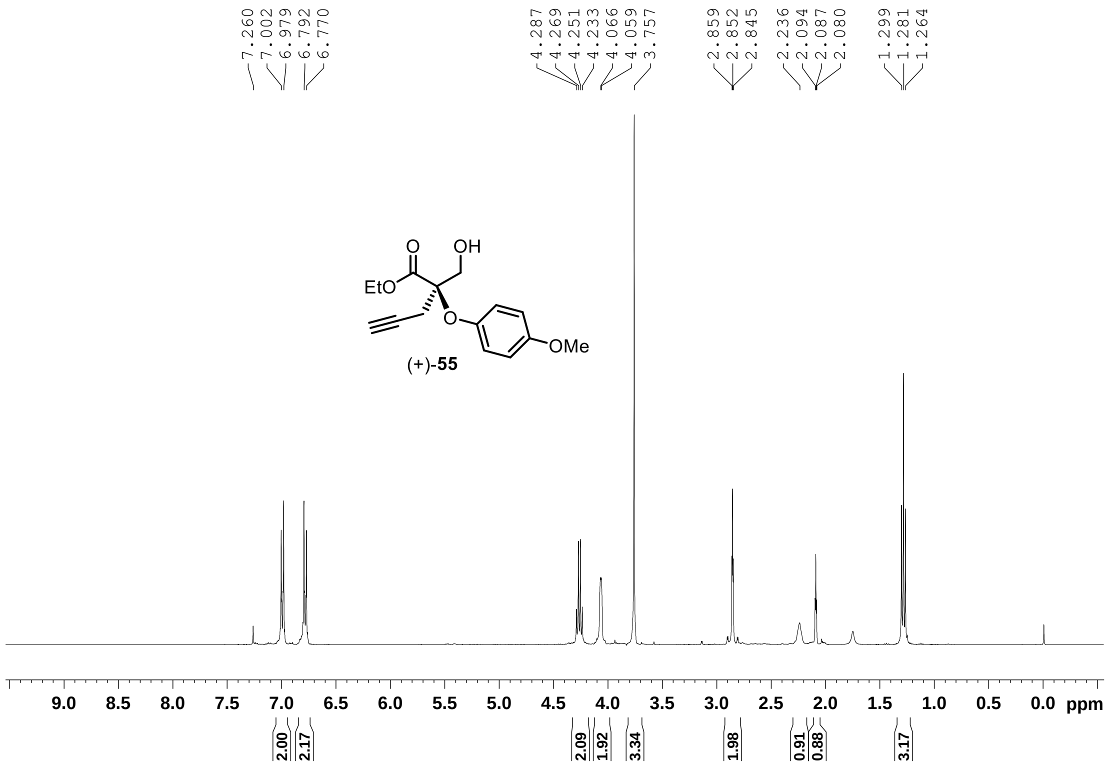

Supplementary Fig. 218. ^{13}C NMR of compound (+)-55 ($CDCl_3$, 100 MHz, 25 °C)

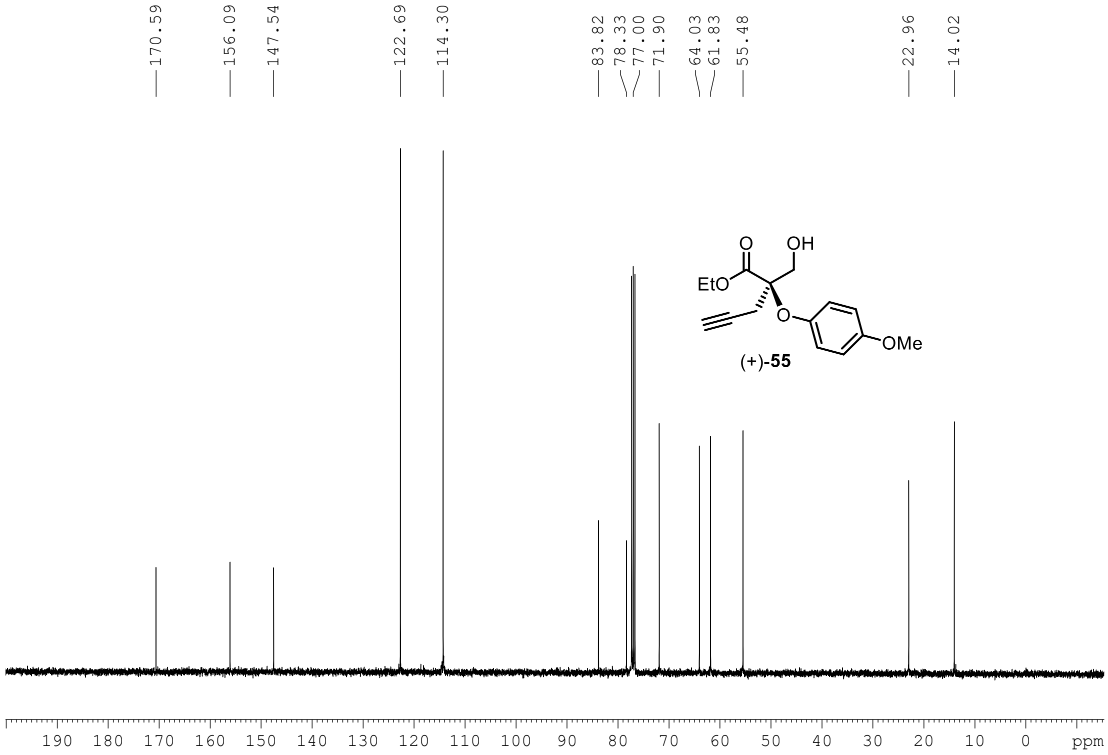

Supplementary Fig. 219. ^{1}H NMR of compound (+)-13 ($CDCl_3$, 400 MHz, 25 °C)

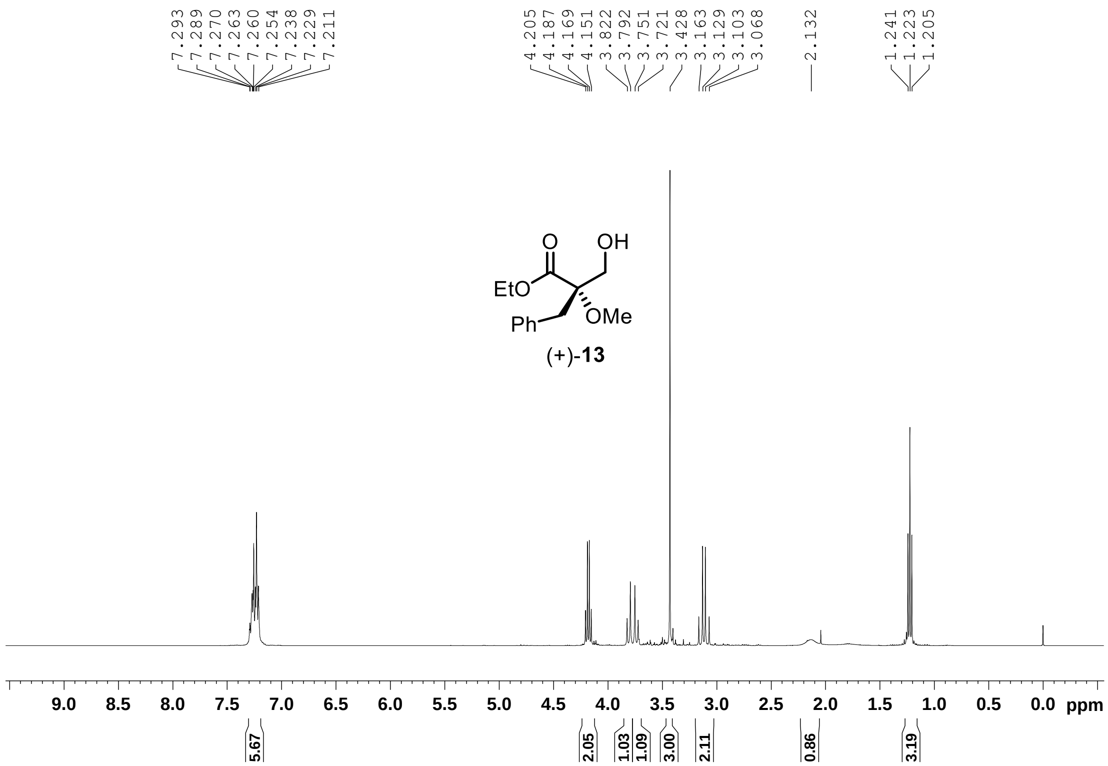

Supplementary Fig. 220. ^{13}C NMR of compound (+)-13 ($CDCl_3$, 100 MHz, 25 °C)

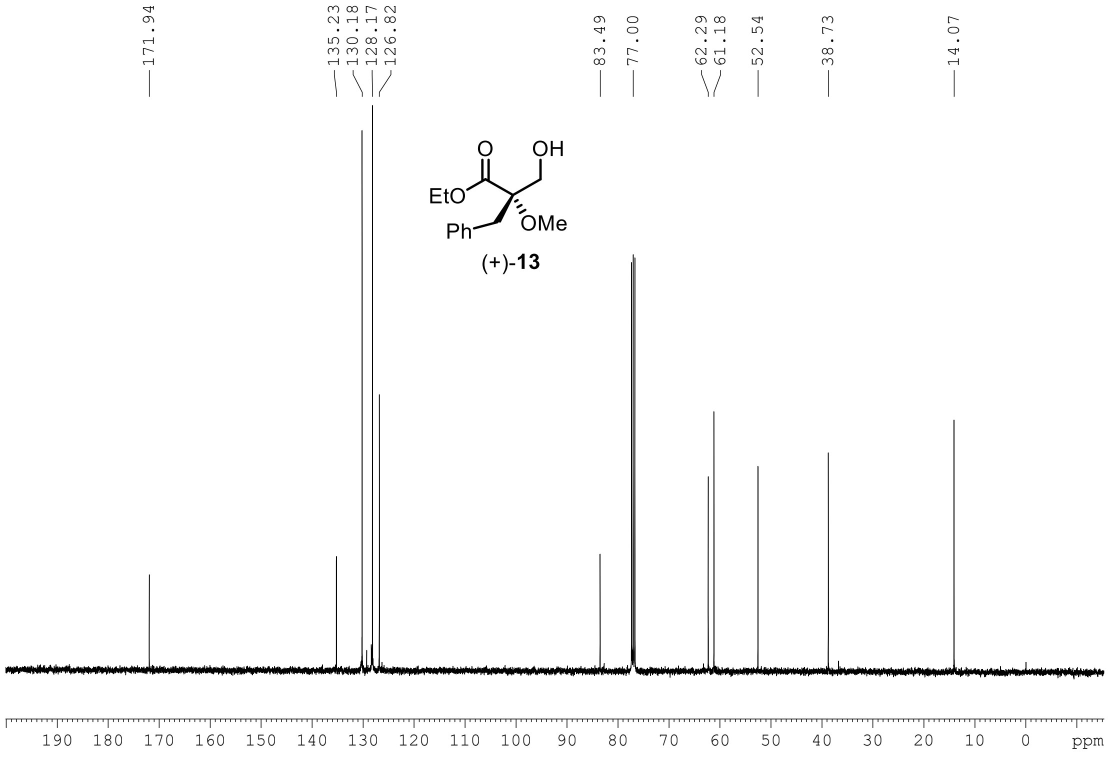

Supplementary Fig. 221. ^{1}**H NMR of compound (+)-57 (CDCl$_3$, 400 MHz, 25 °C)**

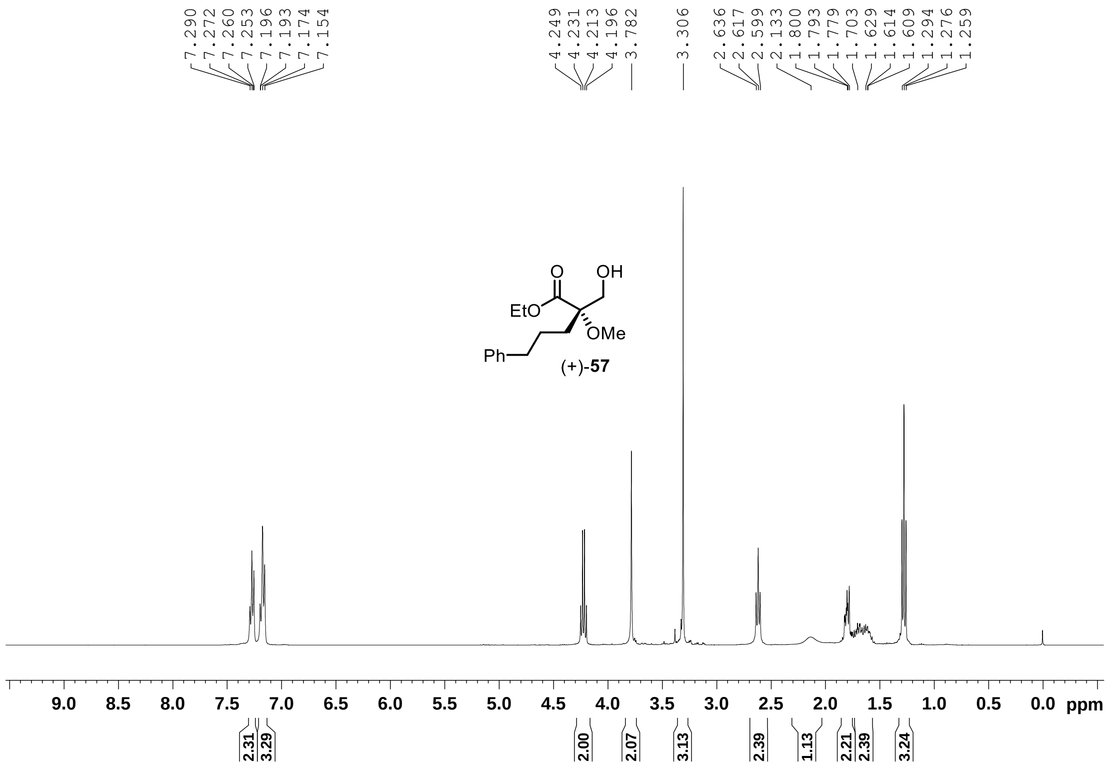

Supplementary Fig. 222. ^{13}**C NMR of compound (+)-57 (CDCl$_3$, 100 MHz, 25 °C)**

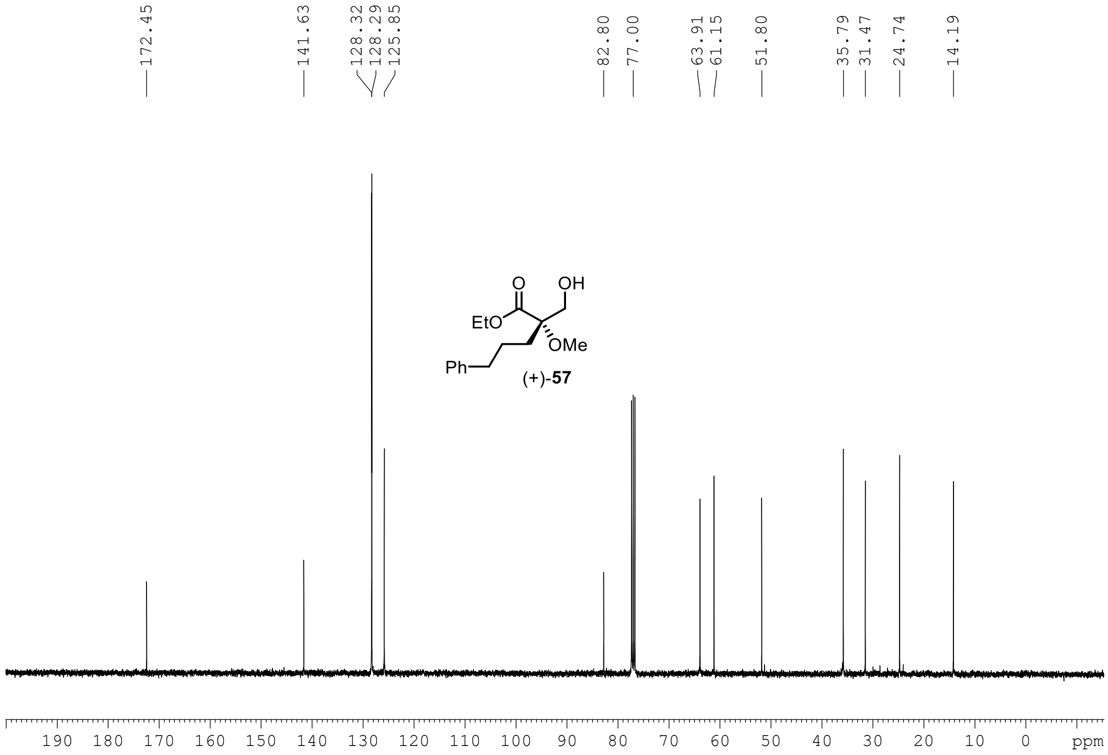

Supplementary Fig. 223. ^{1}H NMR of compound (–)-58 ($CDCl_3$, 400 MHz, 25 °C)

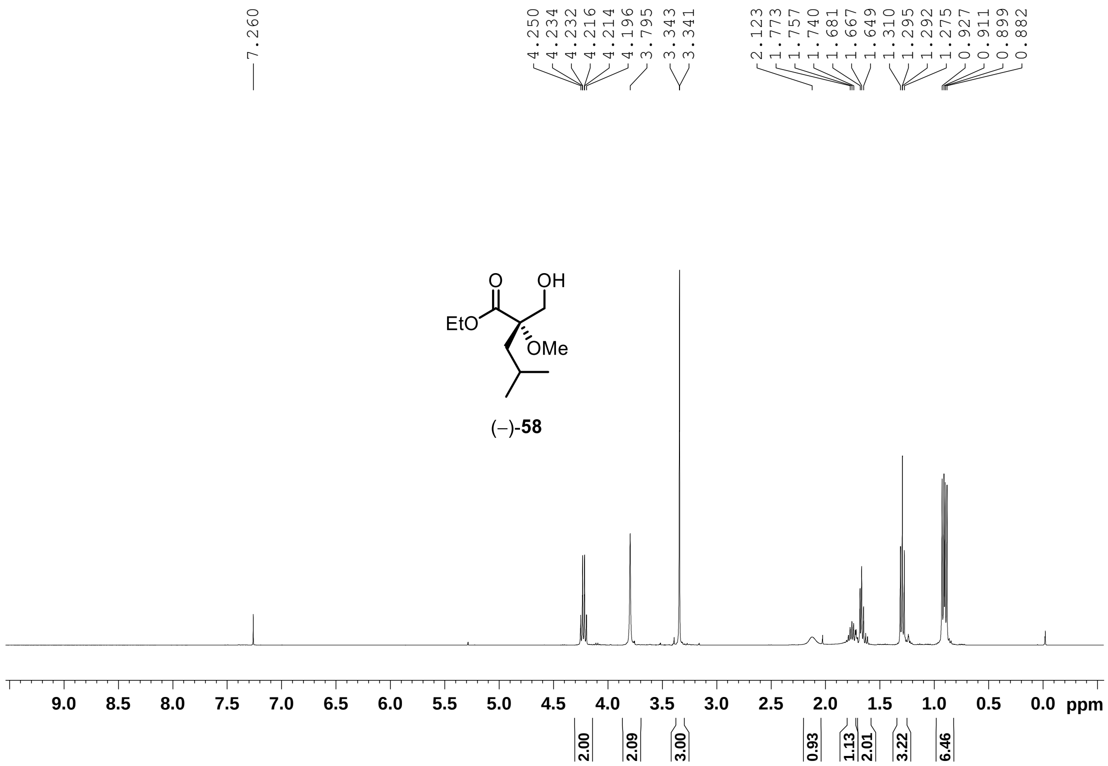

Supplementary Fig. 224. ^{13}C NMR of compound (–)-58 ($CDCl_3$, 100 MHz, 25 °C)

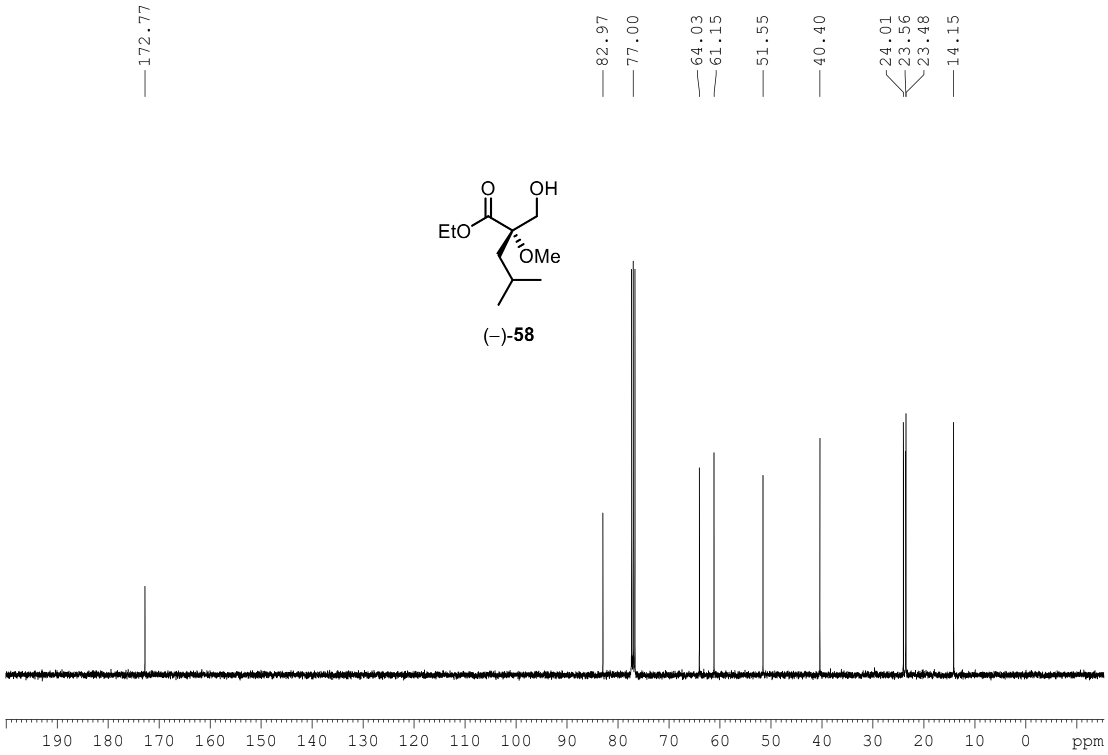

Supplementary Fig. 225. ^{1}H NMR of compound (+)-59 ($CDCl_3$, 400 MHz, 25 °C)

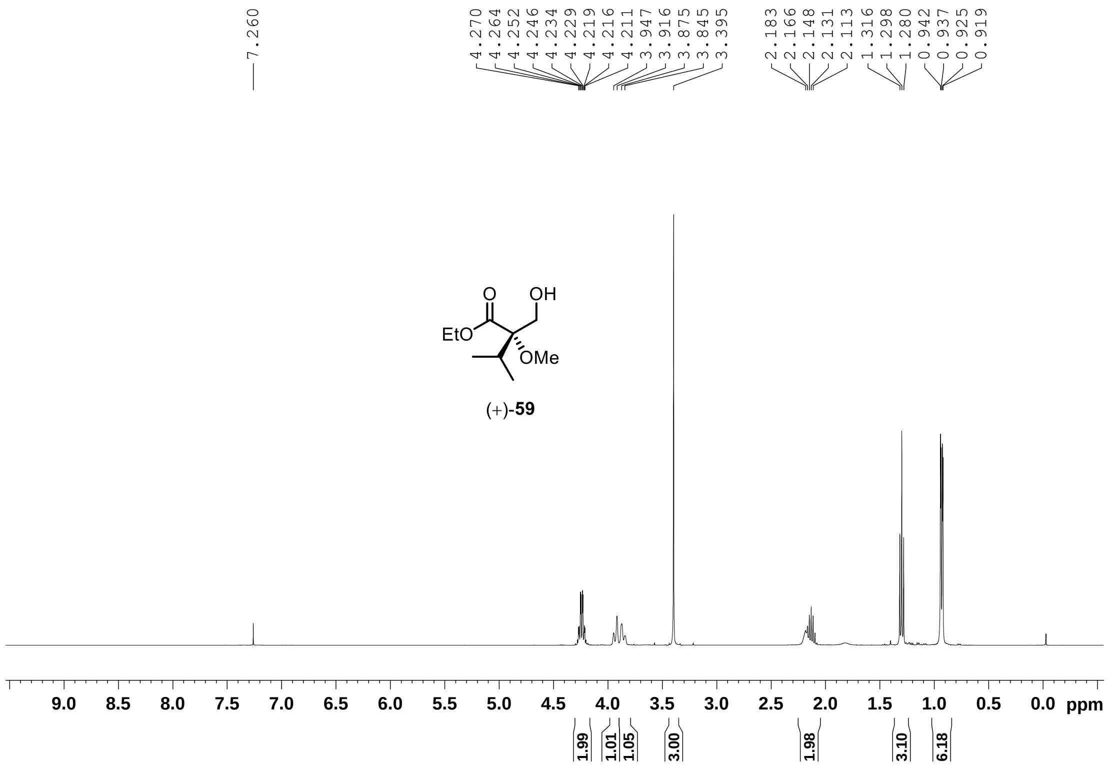

Supplementary Fig. 226. ^{13}C NMR of compound (+)-59 ($CDCl_3$, 100 MHz, 25 °C)

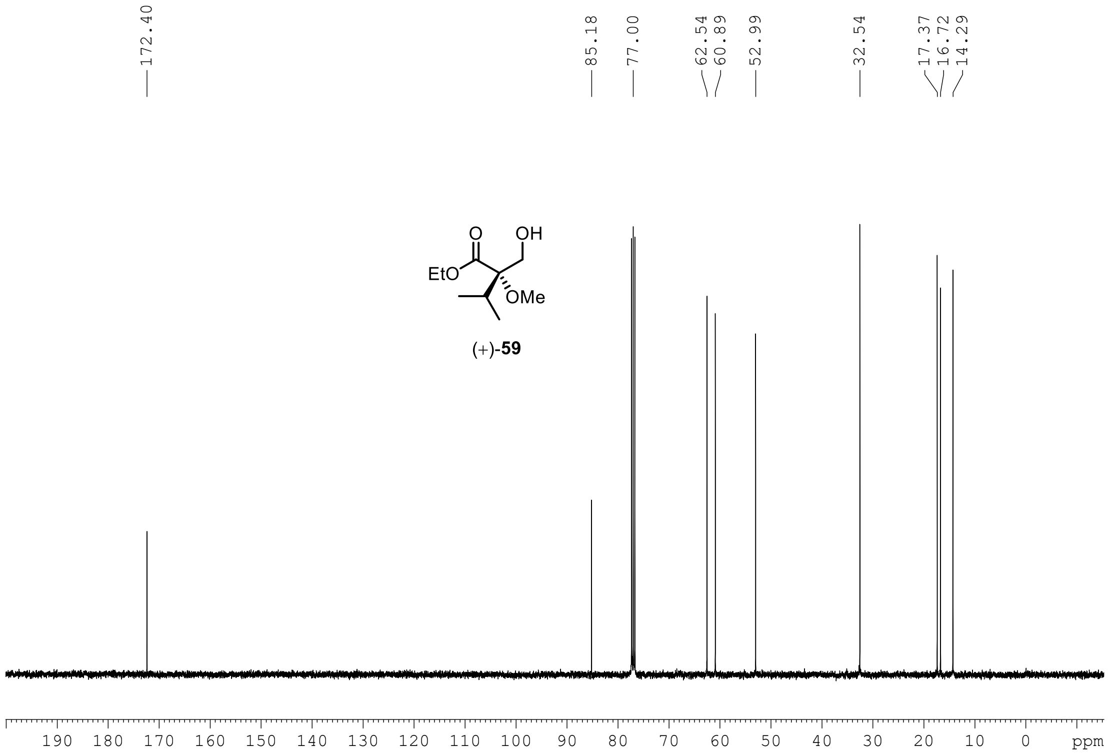

Supplementary Fig. 227. ^{1}H NMR of compound (+)-60 ($CDCl_3$, 400 MHz, 25 °C)

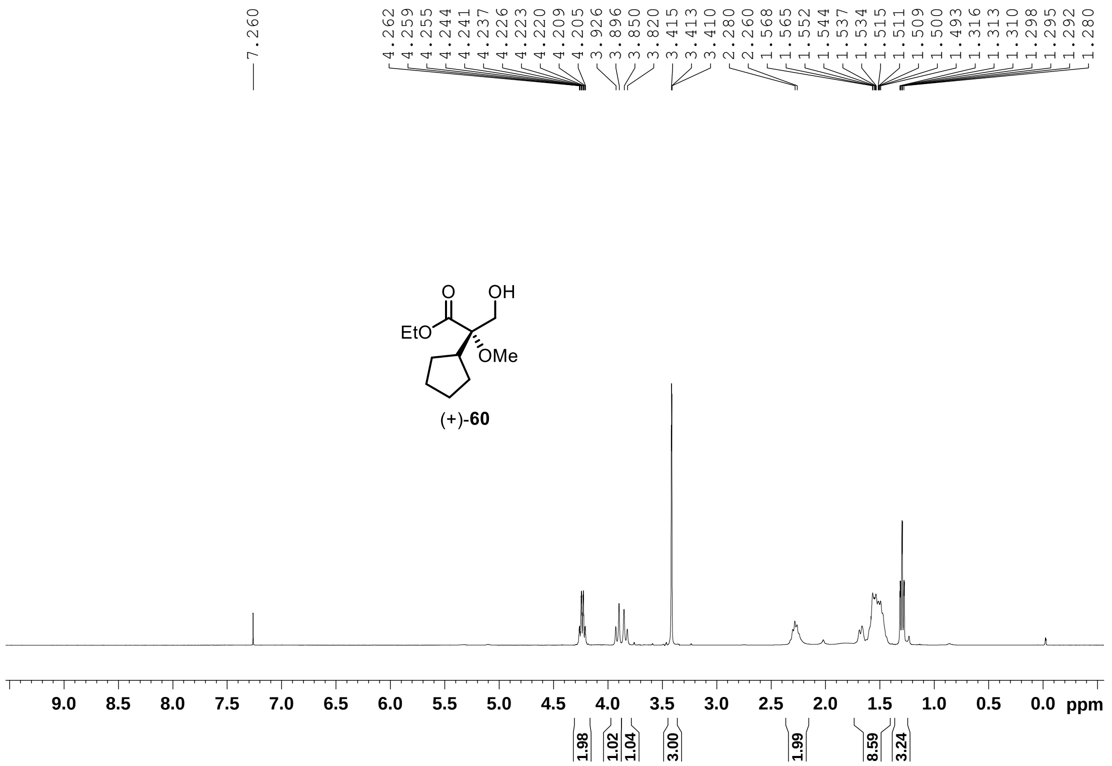

Supplementary Fig. 228. ^{13}C NMR of compound (+)-60 ($CDCl_3$, 100 MHz, 25 °C)

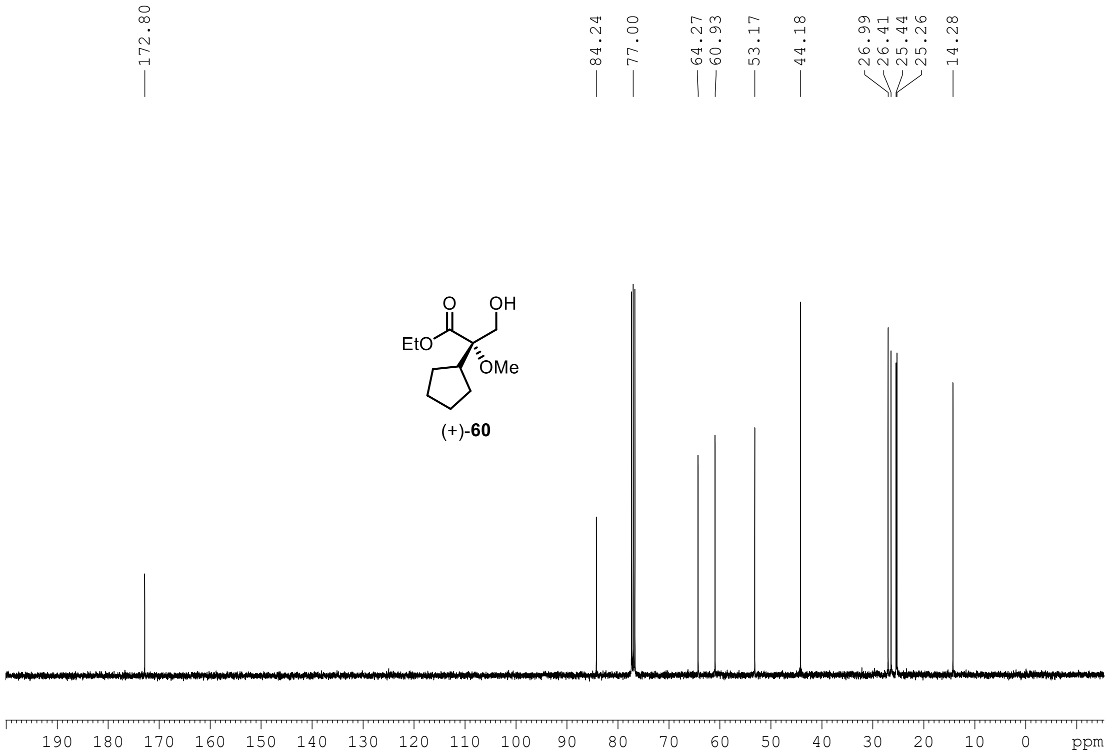

Supplementary Fig. 229. ^{1}H NMR of compound (+)-61 ($CDCl_3$, 400 MHz, 25 °C)

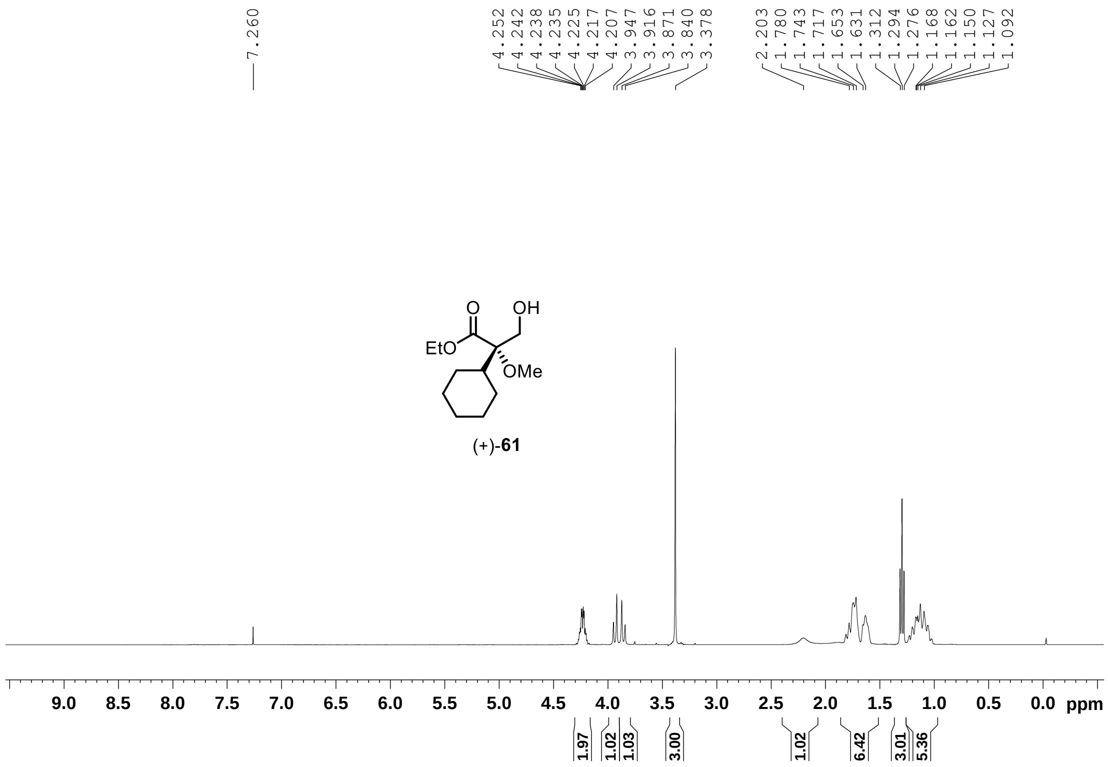

Supplementary Fig. 230. ^{13}C NMR of compound (+)-61 ($CDCl_3$, 100 MHz, 25 °C)

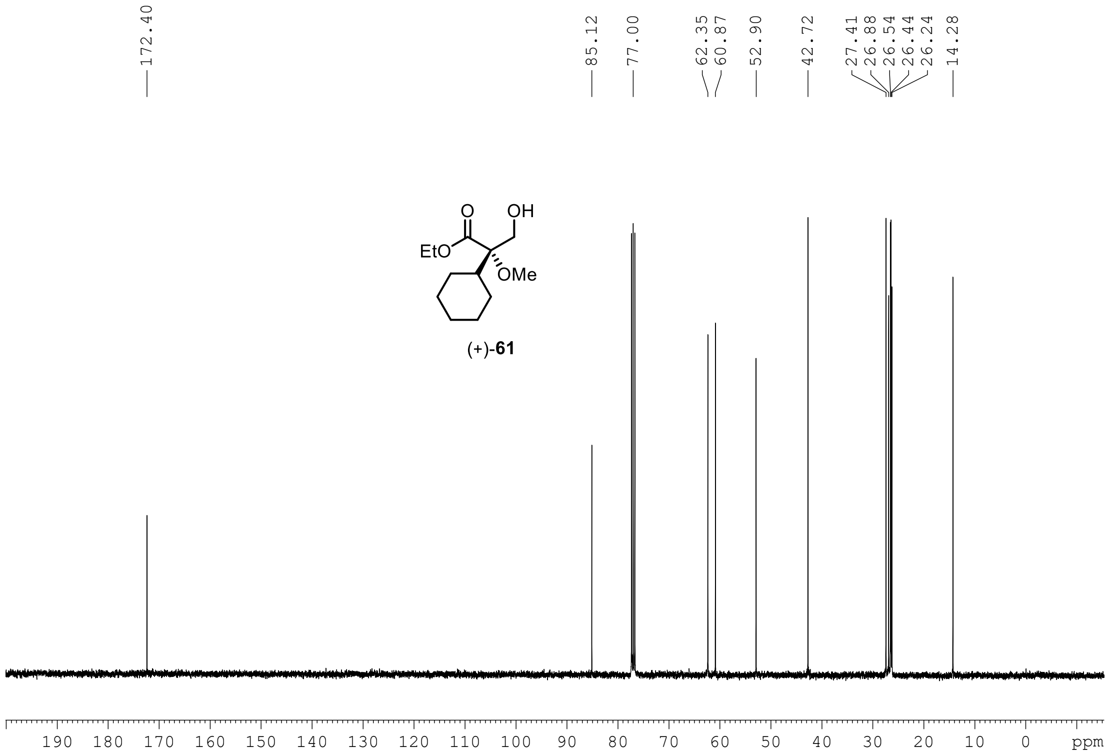

Supplementary Fig. 231. ^{1}H NMR of compound (+)-62 ($CDCl_3$, 400 MHz, 25 °C)

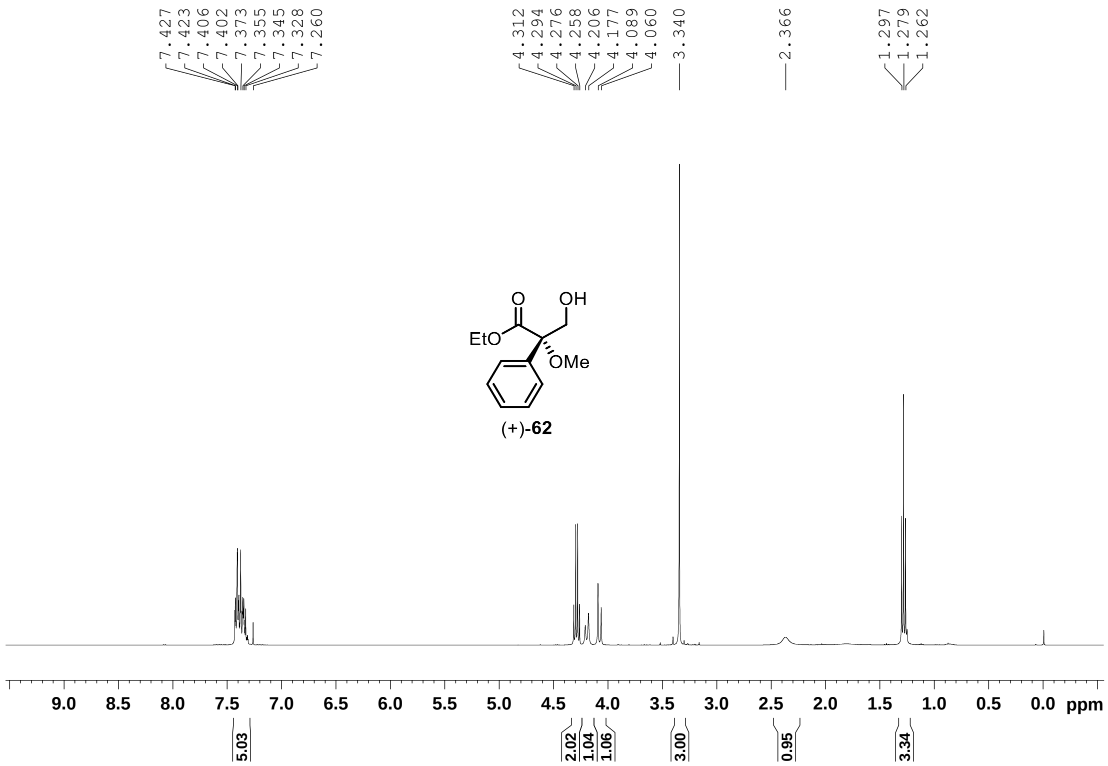

Supplementary Fig. 232. ^{13}C NMR of compound (+)-62 ($CDCl_3$, 100 MHz, 25 °C)

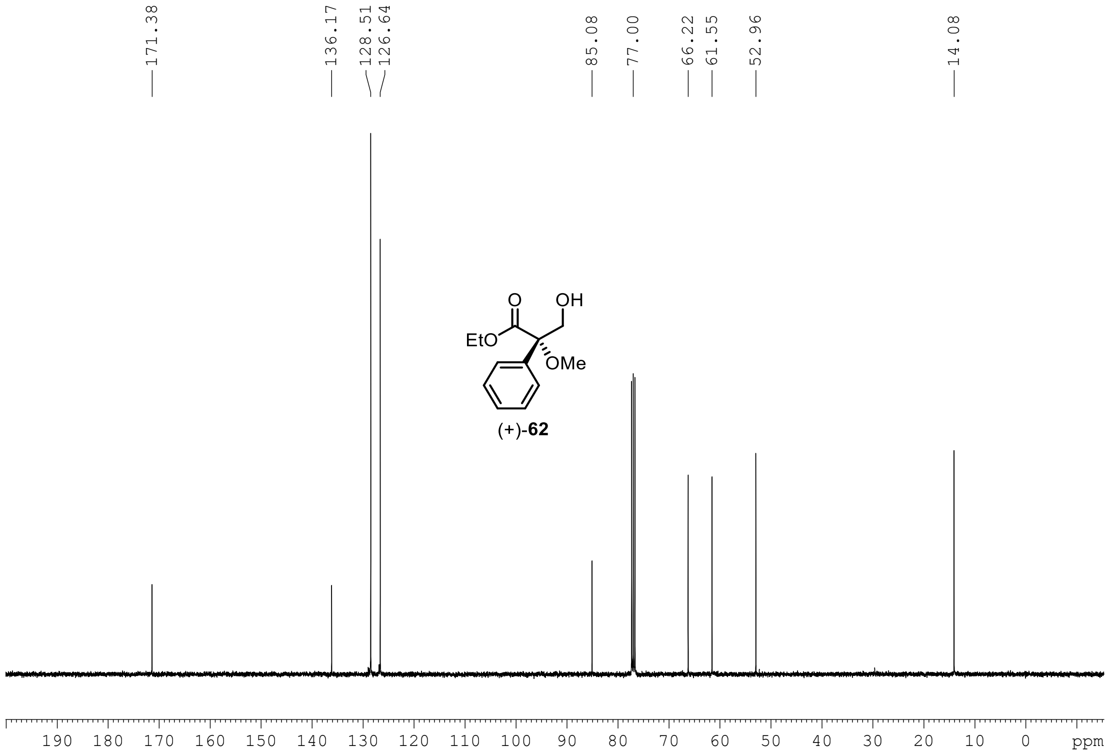

Supplementary Fig. 233. ¹H NMR of compound (−)-63 (CDCl₃, 400 MHz, 25 °C)

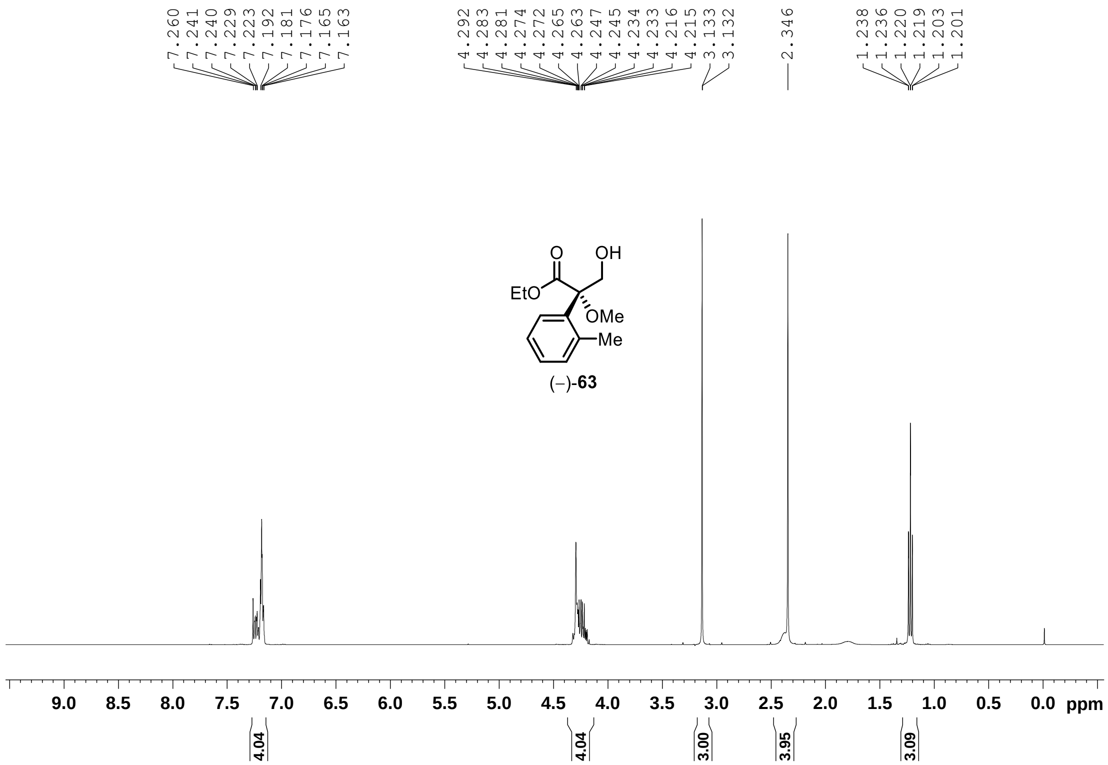

Supplementary Fig. 234. ¹³C NMR of compound (−)-63 (CDCl₃, 100 MHz, 25 °C)

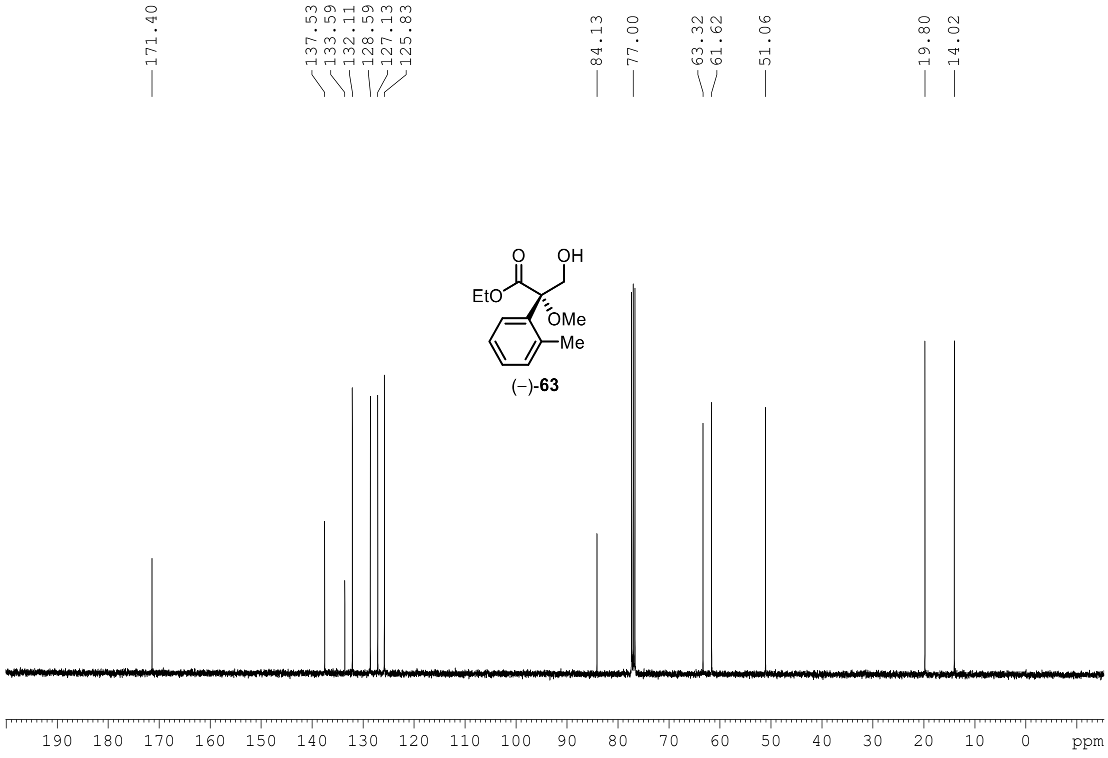

Supplementary Fig. 235. ^{1}H NMR of compound (+)-64 (CDCl$_3$, 400 MHz, 25 °C)

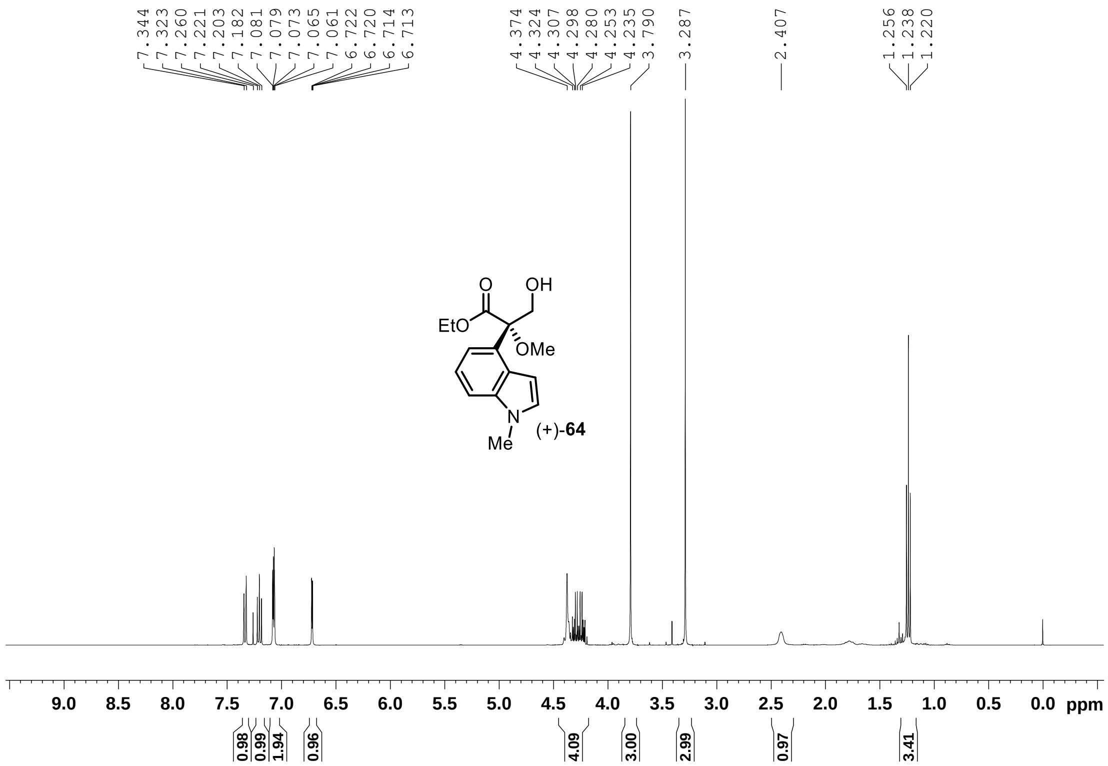

Supplementary Fig. 236. ^{13}C NMR of compound (+)-64 (CDCl$_3$, 100 MHz, 25 °C)

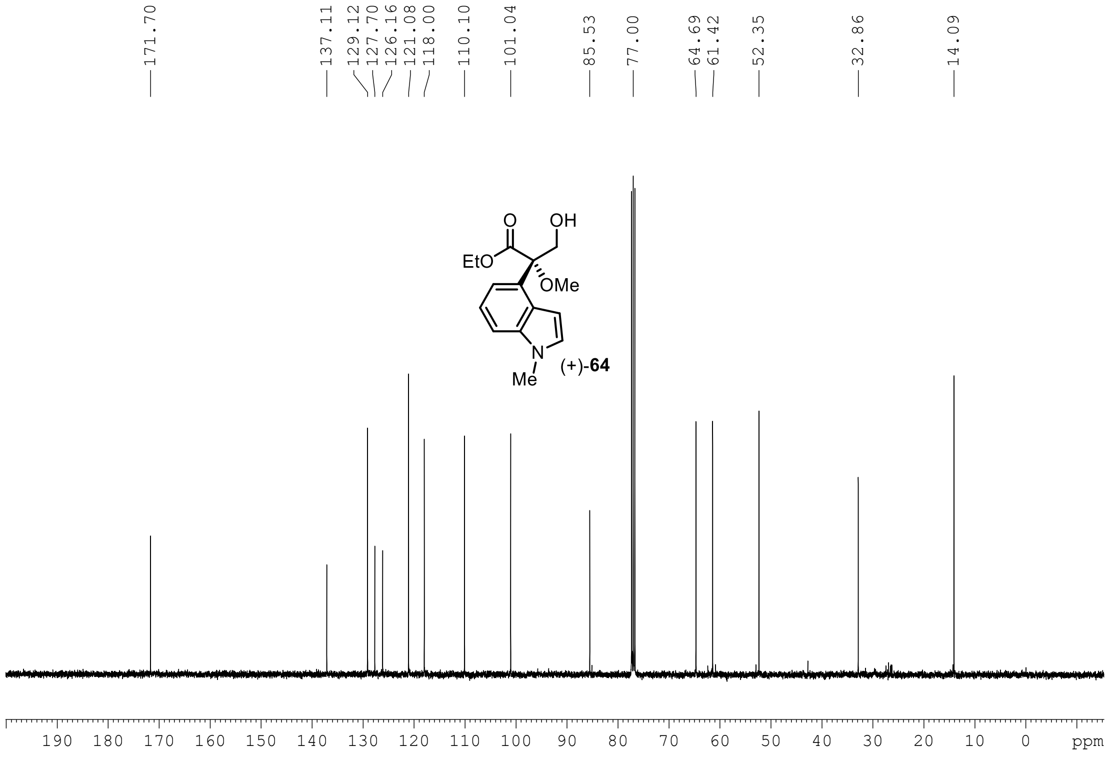

Supplementary Fig. 237. ^{1}H NMR of compound (+)-66 (CDCl3, 400 MHz, 25 °C)

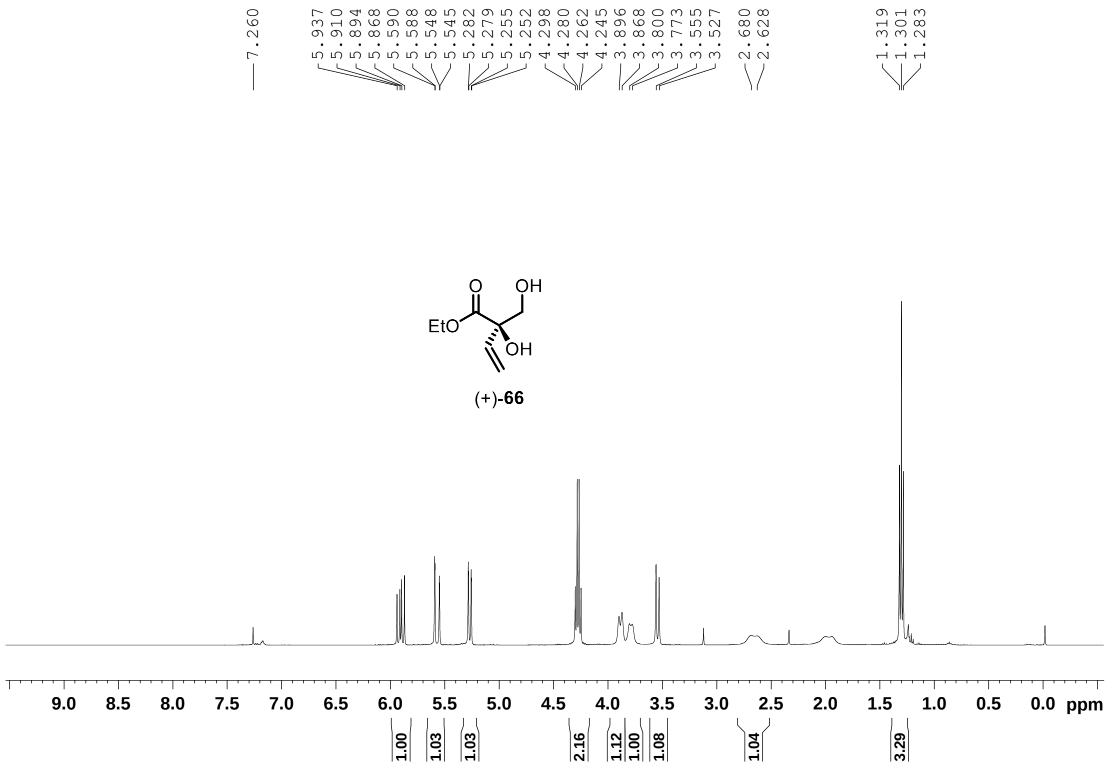

Supplementary Fig. 238. ^{13}C NMR of compound (+)-66 (CDCl3, 100 MHz, 25 °C)

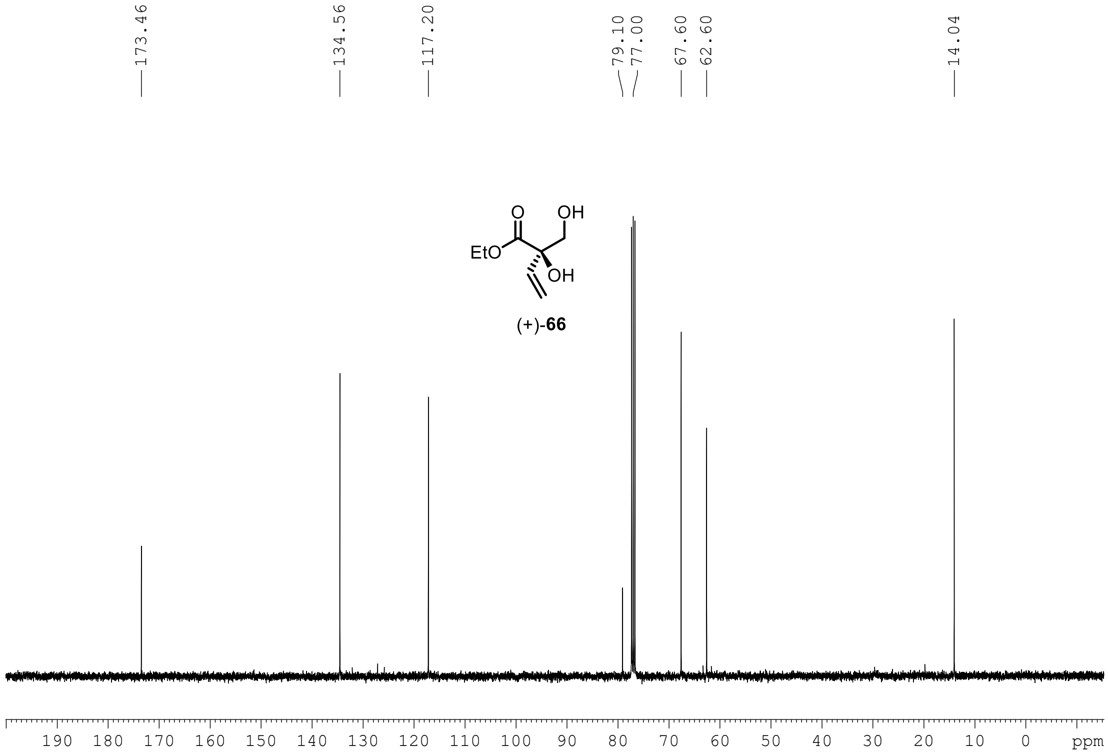

Supplementary Fig. 239. ¹H NMR of compound (+)-14 (CDCl₃, 400 MHz, 25 °C)

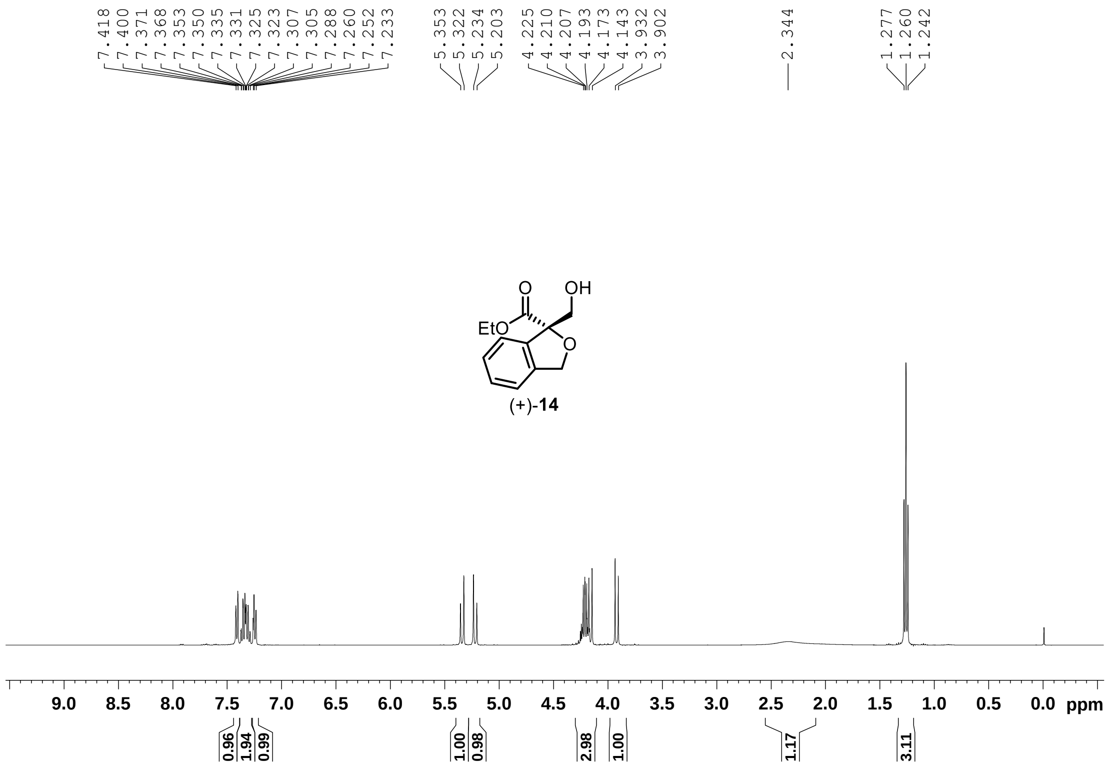

Supplementary Fig. 240. ¹³C NMR of compound (+)-14 (CDCl₃, 100 MHz, 25 °C)

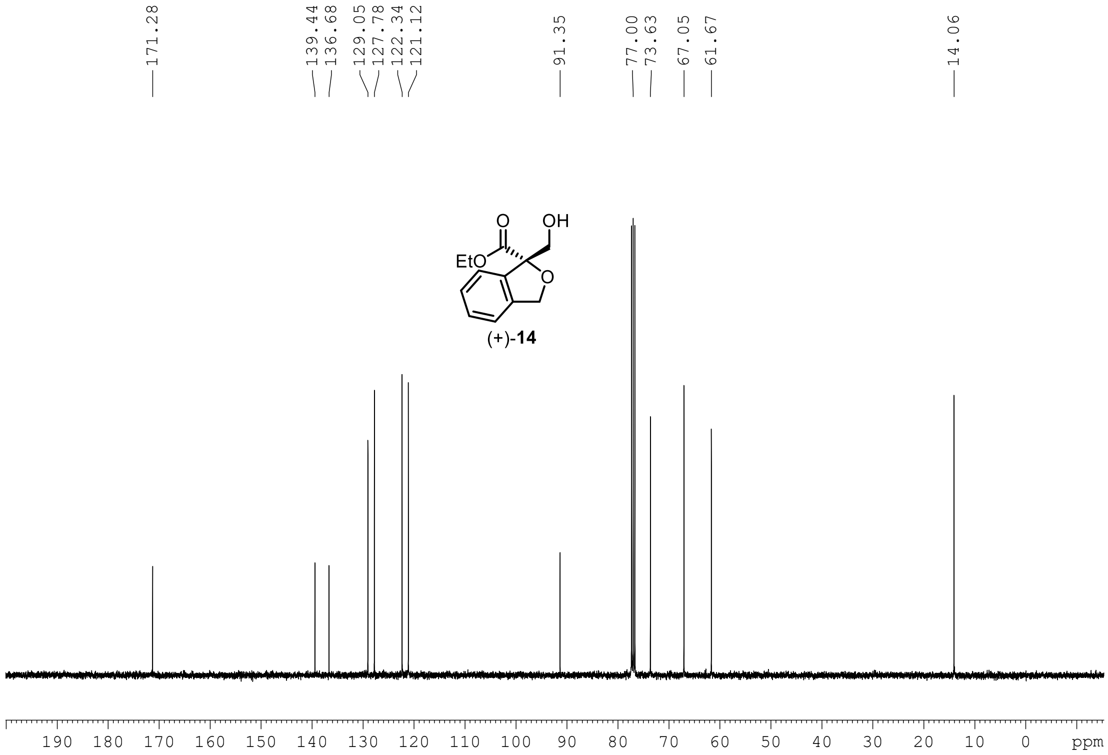

Supplementary Fig. 241. ^{1}H NMR of compound (+)-67 ($CDCl_3$, 400 MHz, 25 °C)

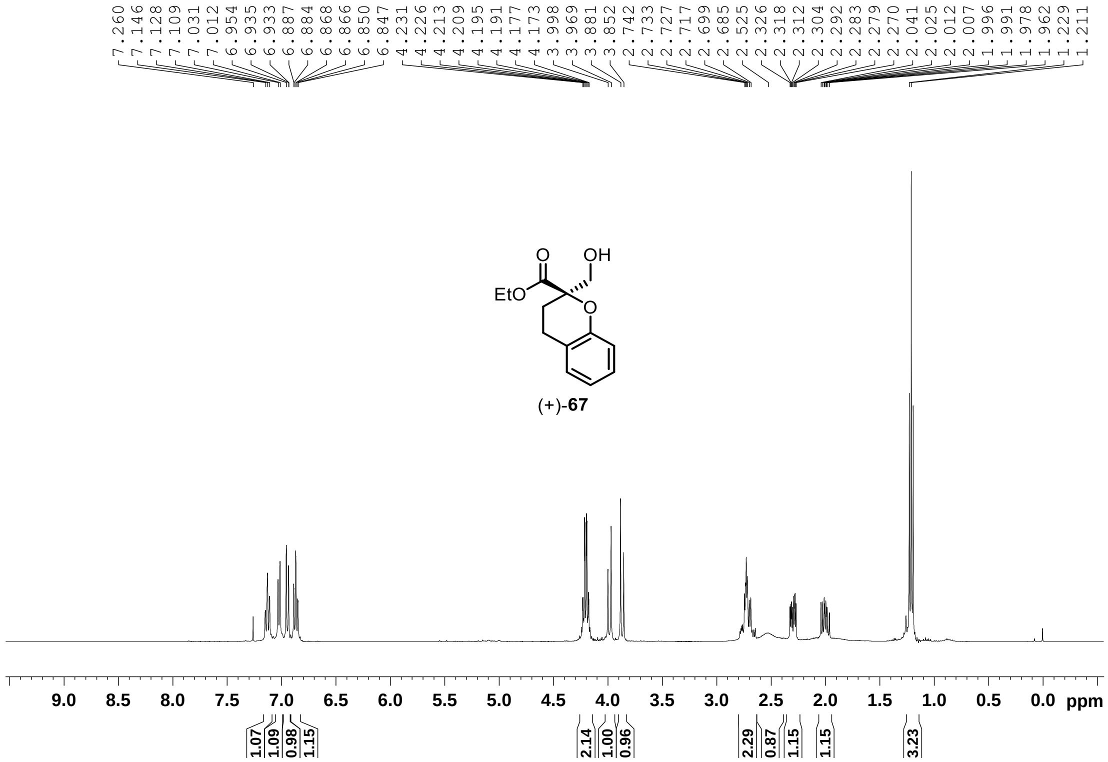

Supplementary Fig. 242. ^{13}C NMR of compound (+)-67 ($CDCl_3$, 100 MHz, 25 °C)

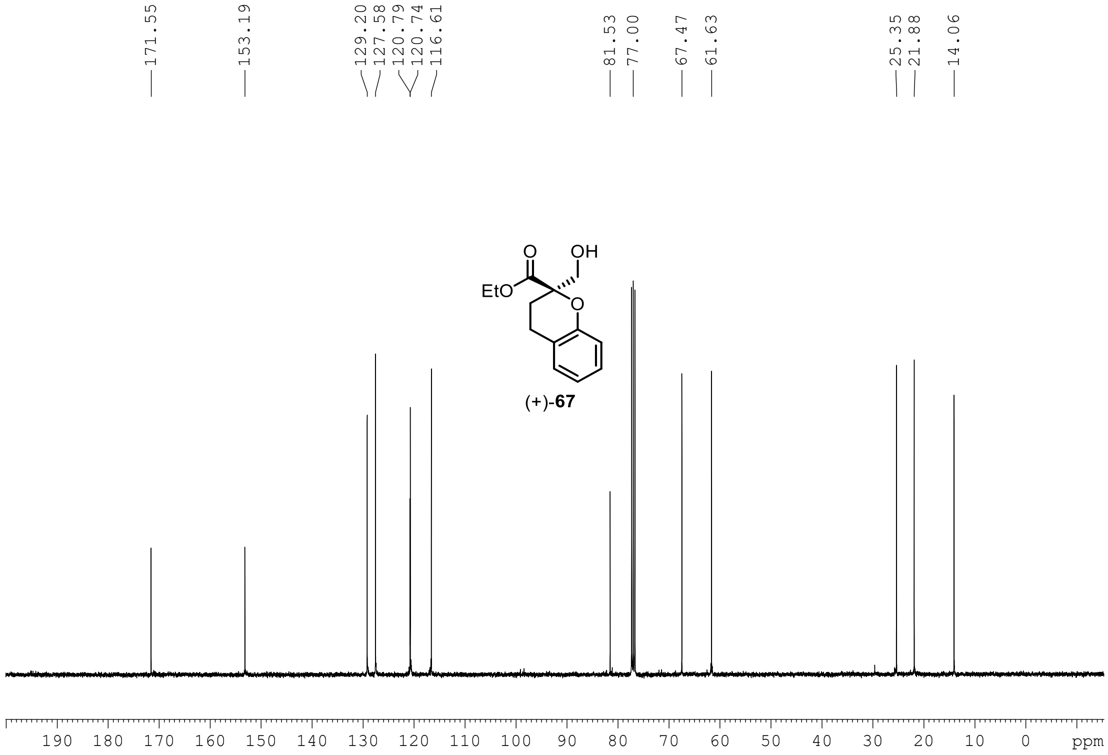

Supplementary Fig. 243. ^{1}H NMR of compound (–)-68 ($CDCl_3$, 400 MHz, 25 °C)

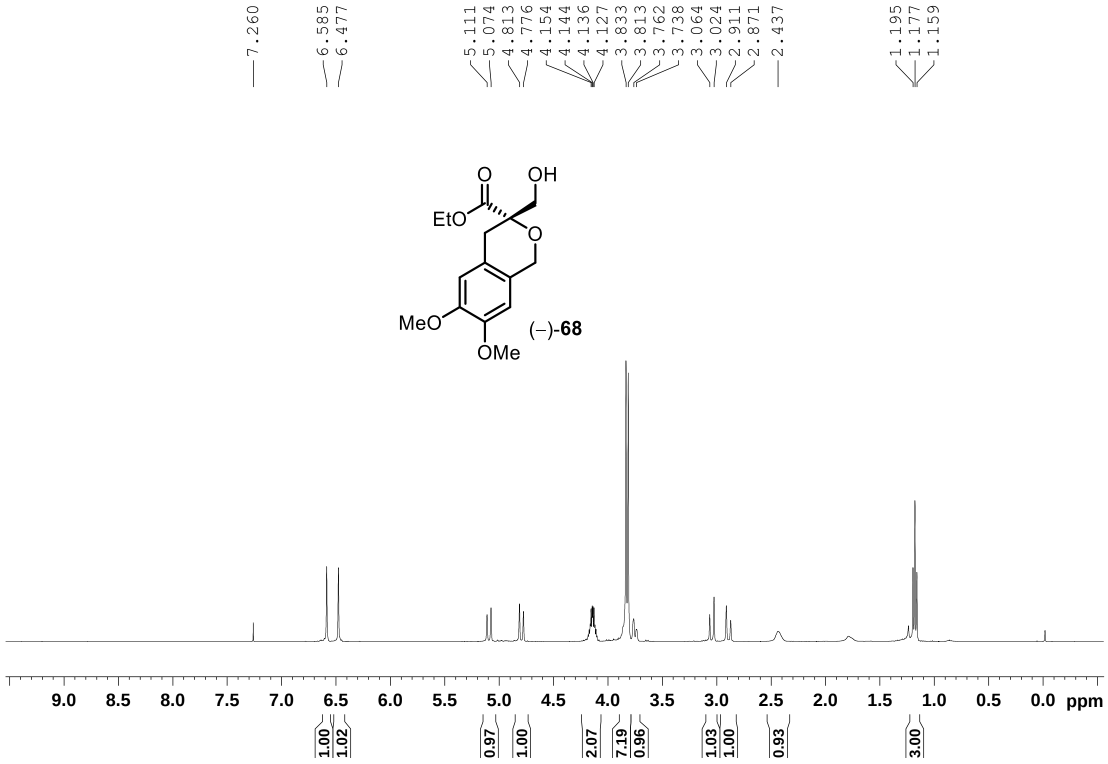

Supplementary Fig. 244. ^{13}C NMR of compound (–)-68 ($CDCl_3$, 100 MHz, 25 °C)

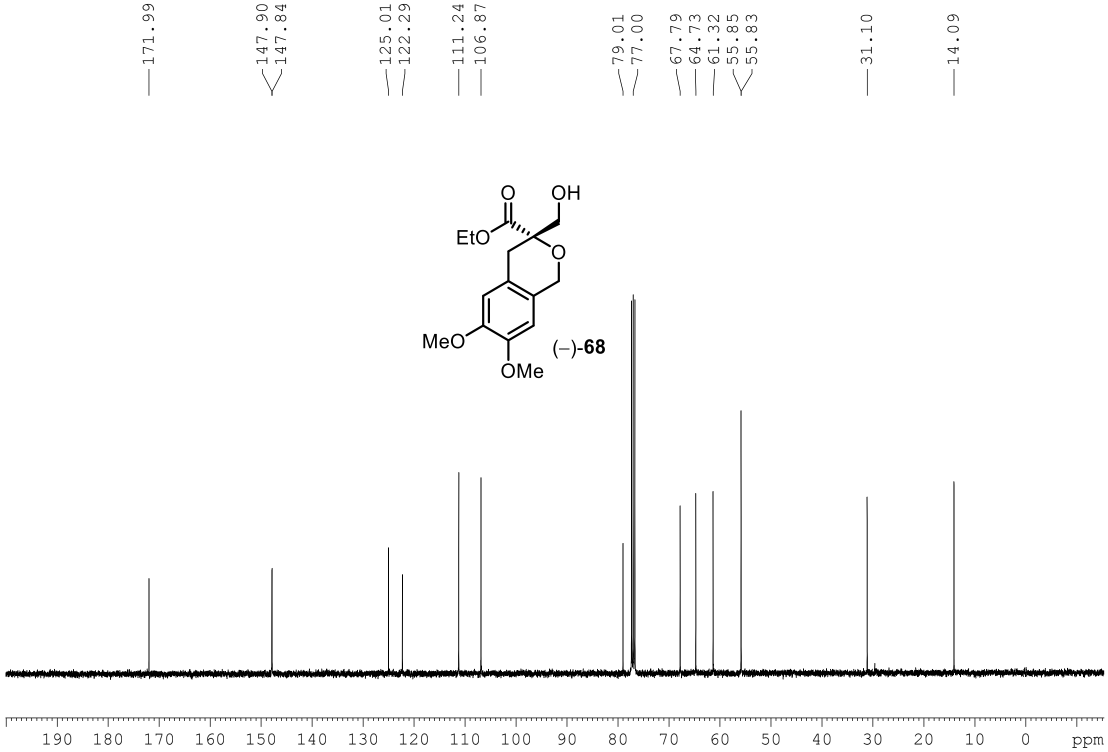

Supplementary Fig. 245. ^{1}H NMR of compound (–)-69 (CDCl3, 400 MHz, 25 °C)

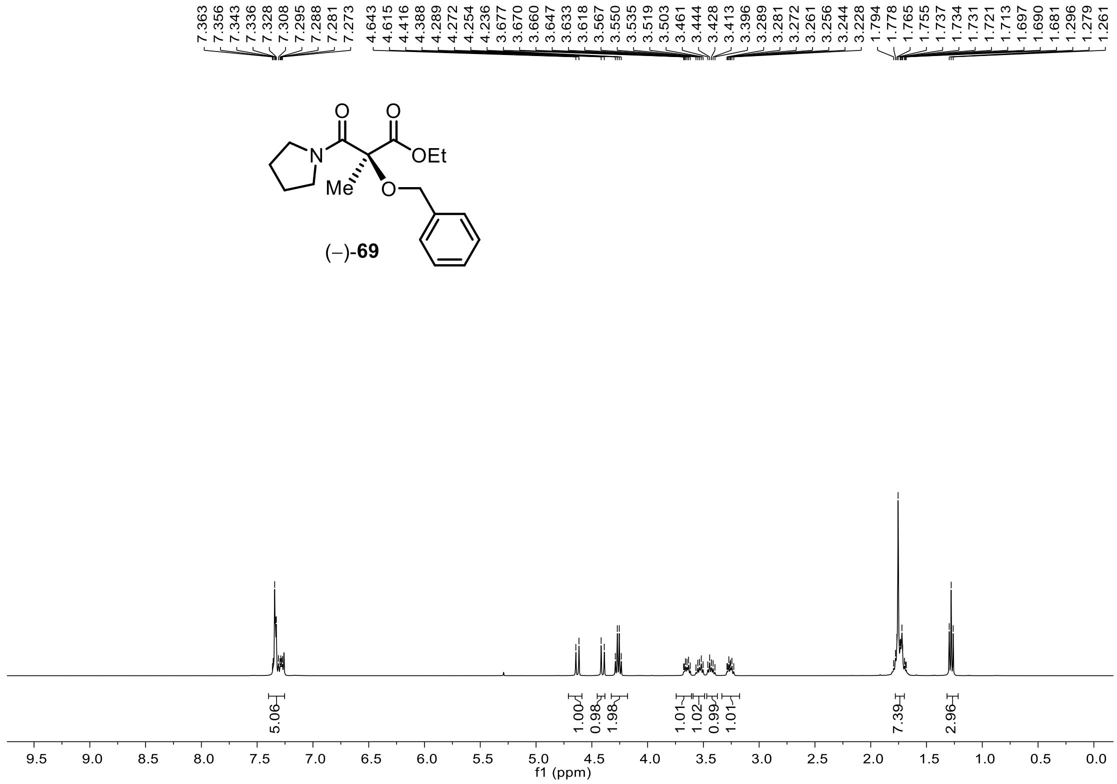

Supplementary Fig. 246. ^{13}C NMR of compound (–)-69 (CDCl3, 100 MHz, 25 °C)

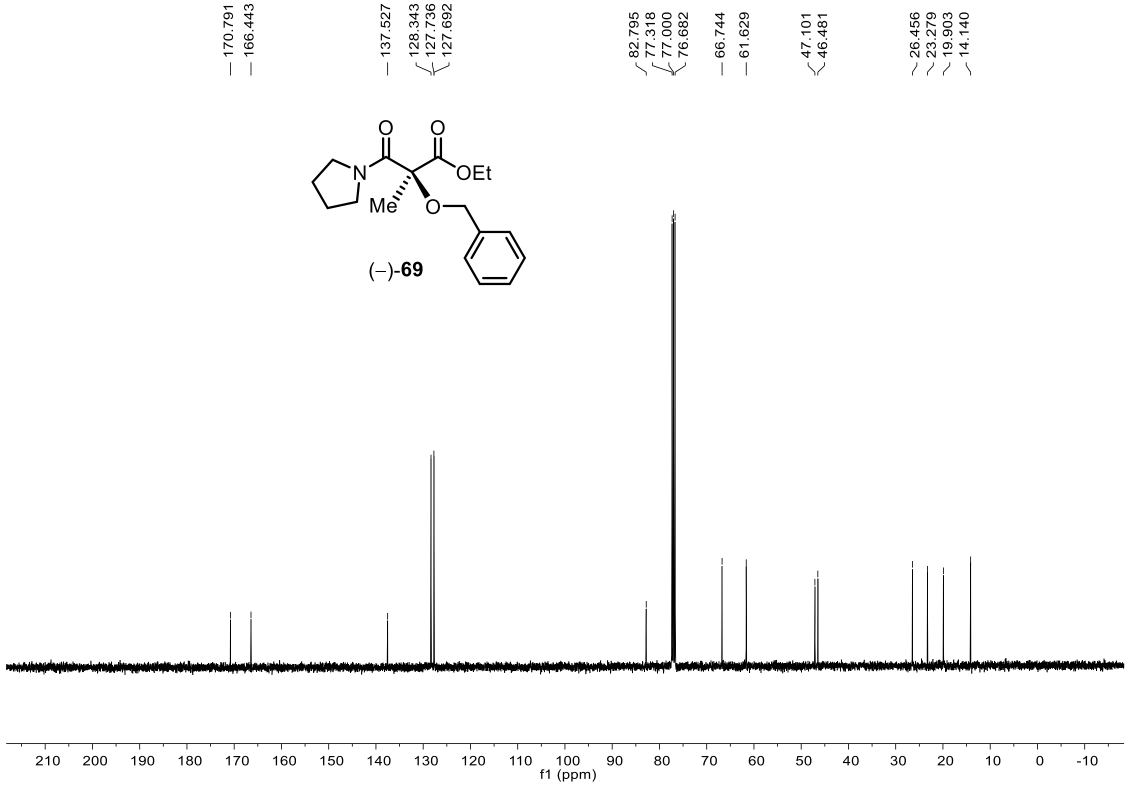

Supplementary Fig. 247. ^{1}H NMR of compound (+)-70 ($CDCl_3$, 400 MHz, 25 °C)

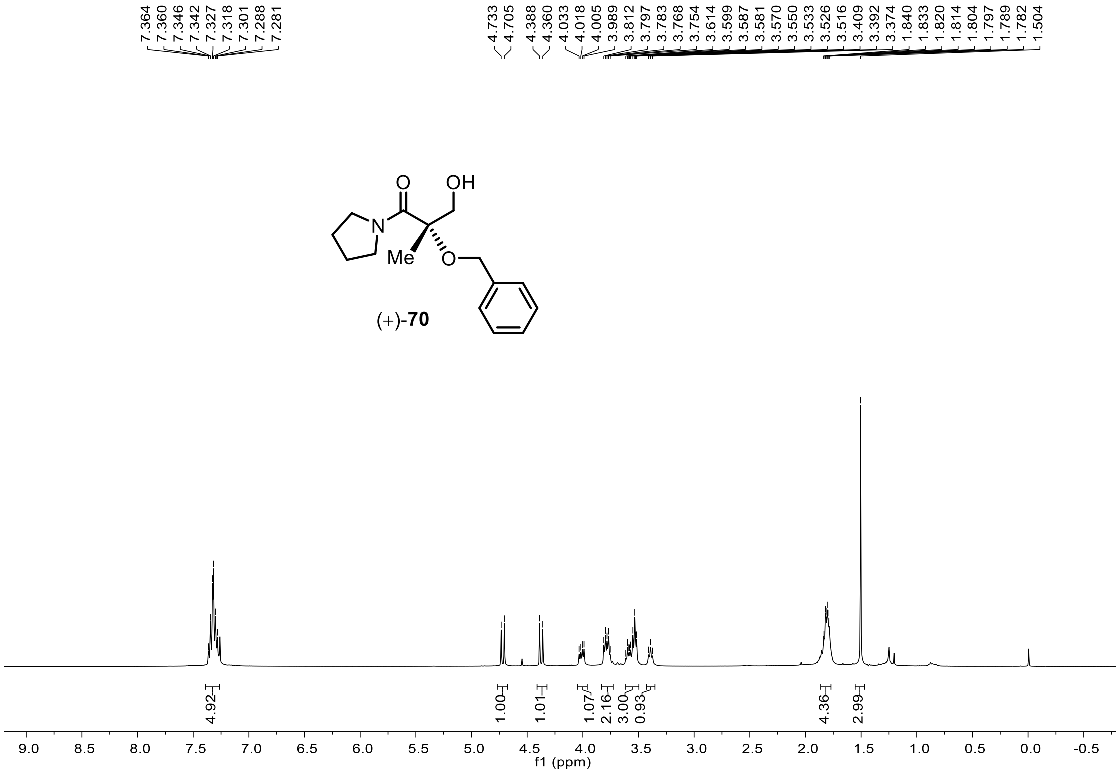

Supplementary Fig. 248. ^{13}C NMR of compound (+)-70 ($CDCl_3$, 100 MHz, 25 °C)

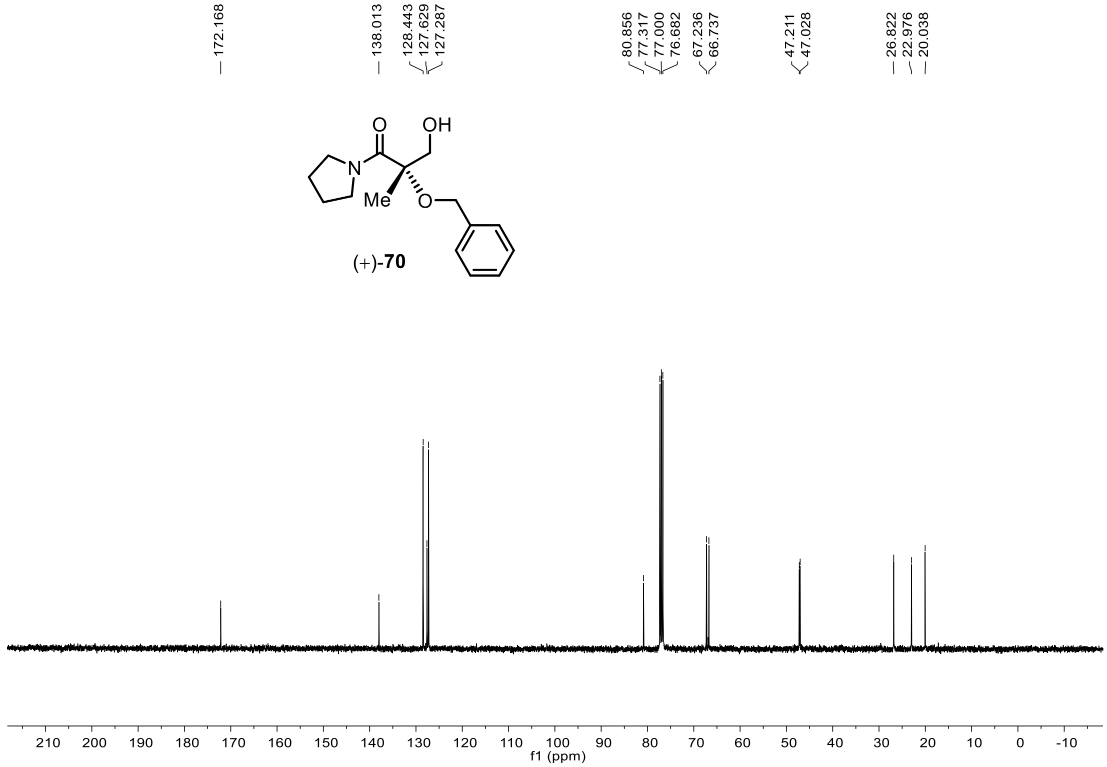

Supplementary Fig. 249. ¹H NMR of compound (+)-72 ($CDCl_3$, 400 MHz, 25 °C)

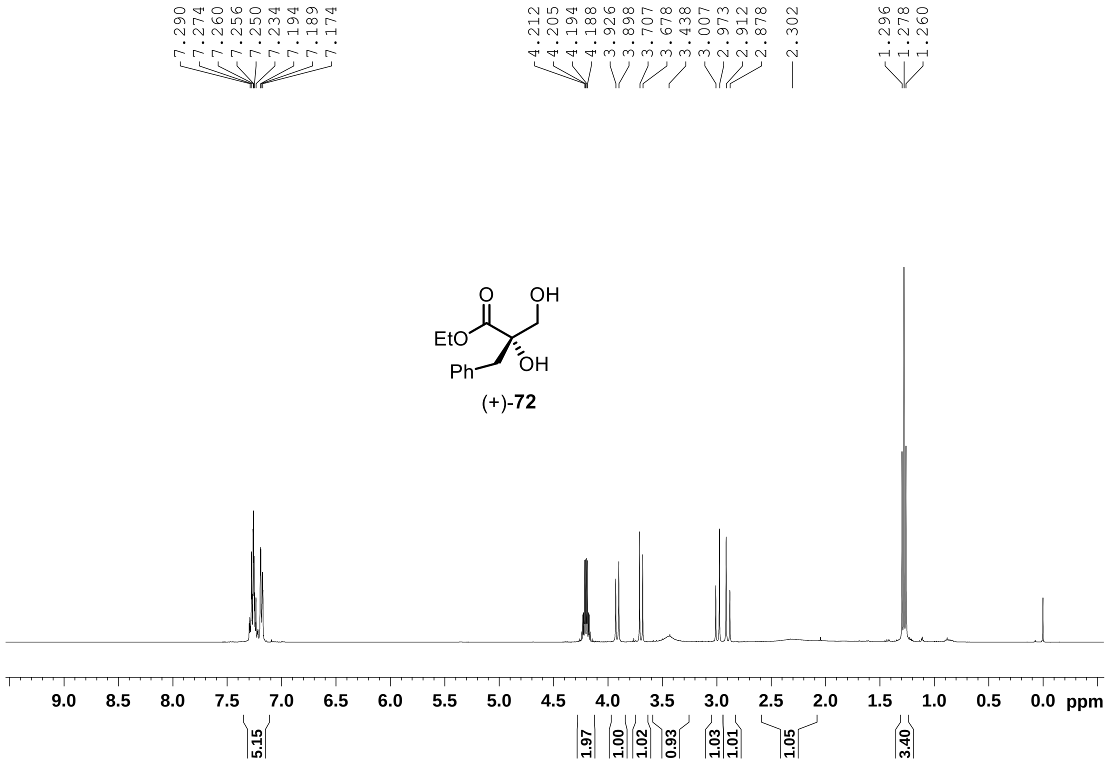

Supplementary Fig. 250. ¹³C NMR of compound (+)-72 ($CDCl_3$, 100 MHz, 25 °C)

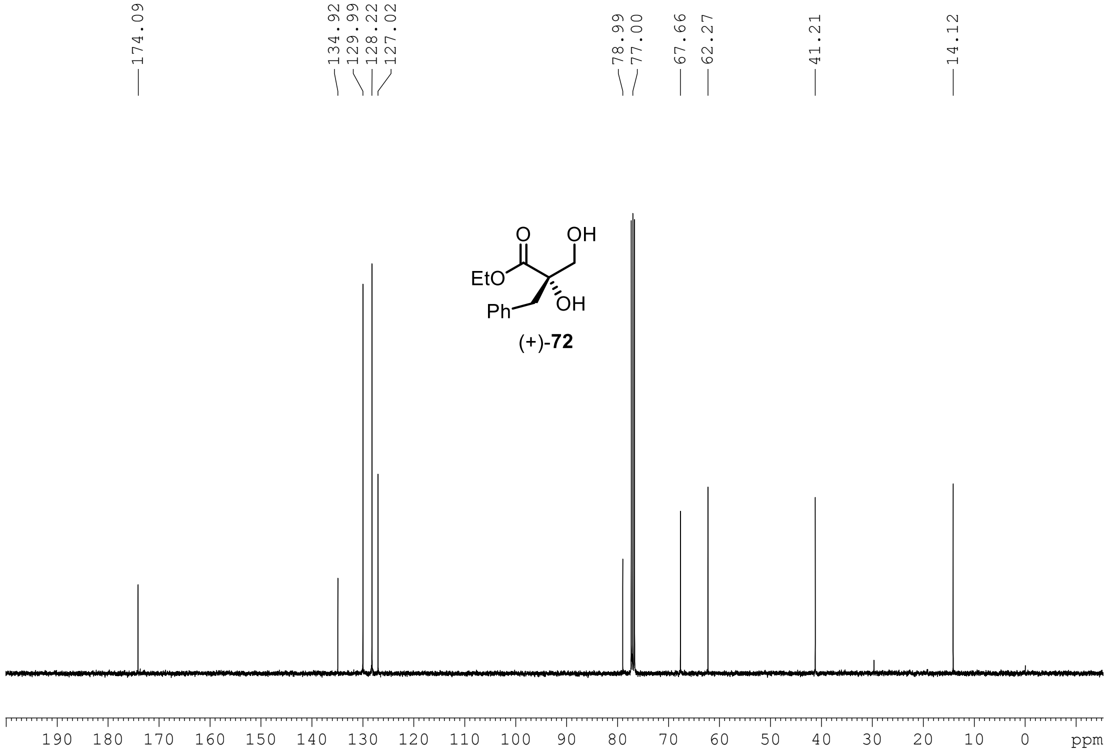

Supplementary Fig. 251. ^{1}H NMR of compound (+)-73 ($CDCl_3$, 400 MHz, 25 °C)

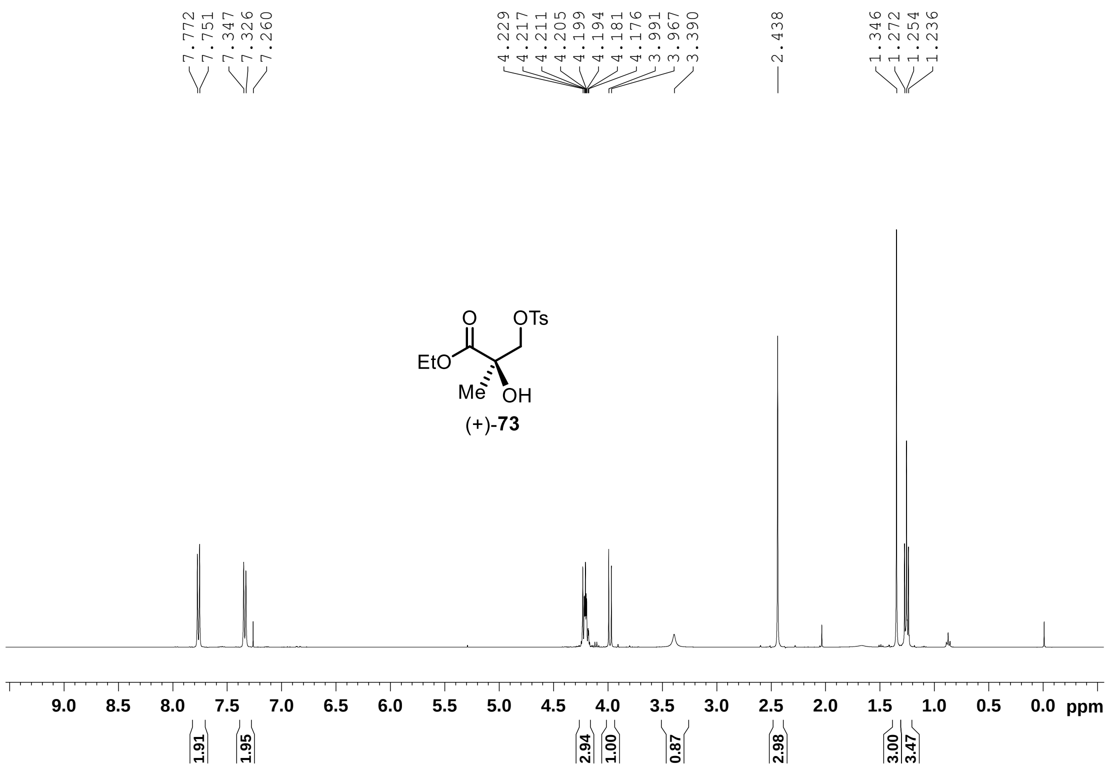

Supplementary Fig. 252. ^{13}C NMR of compound (+)-73 ($CDCl_3$, 100 MHz, 25 °C)

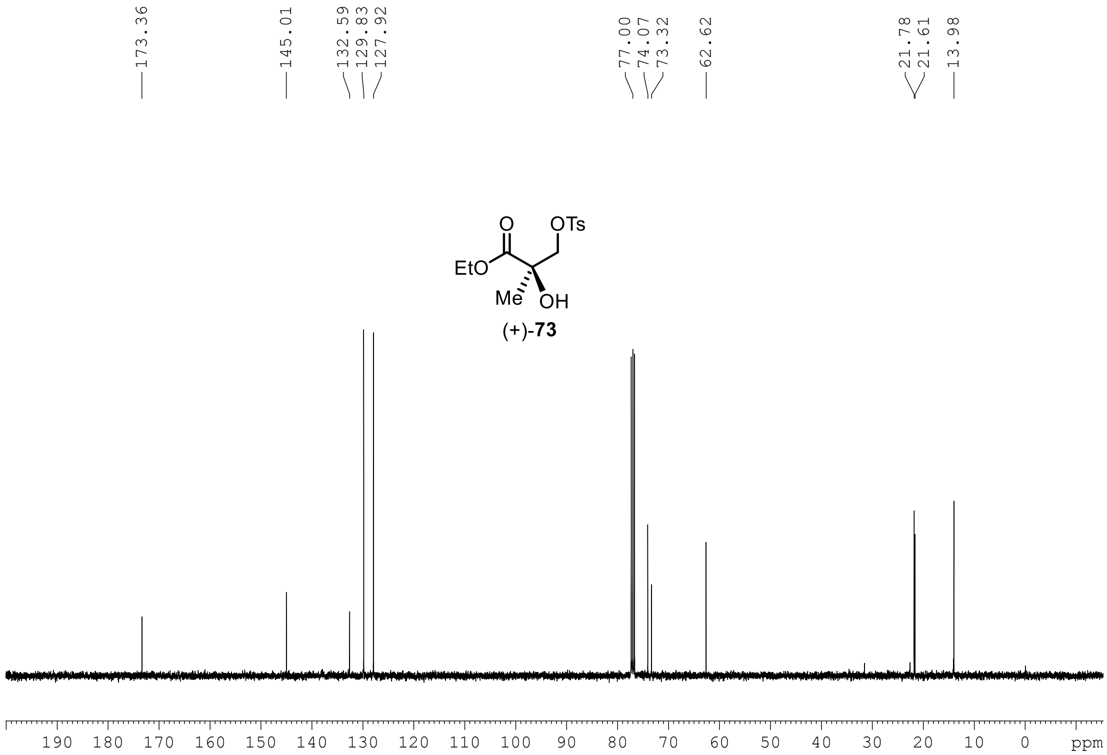

Supplementary Fig. 253. ^{1}H NMR of compound (–)-S74 ($CDCl_3$, 400 MHz, 25 °C)

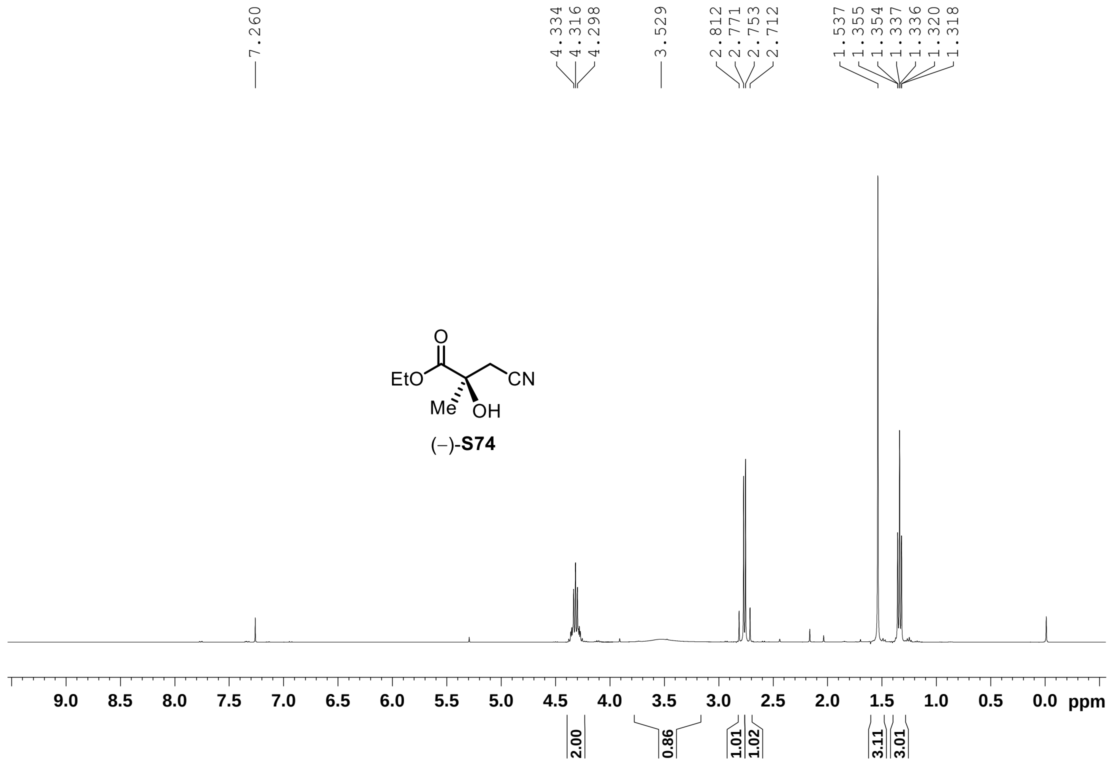

Supplementary Fig. 254. ^{13}C NMR of compound (–)-S74 ($CDCl_3$, 100 MHz, 25 °C)

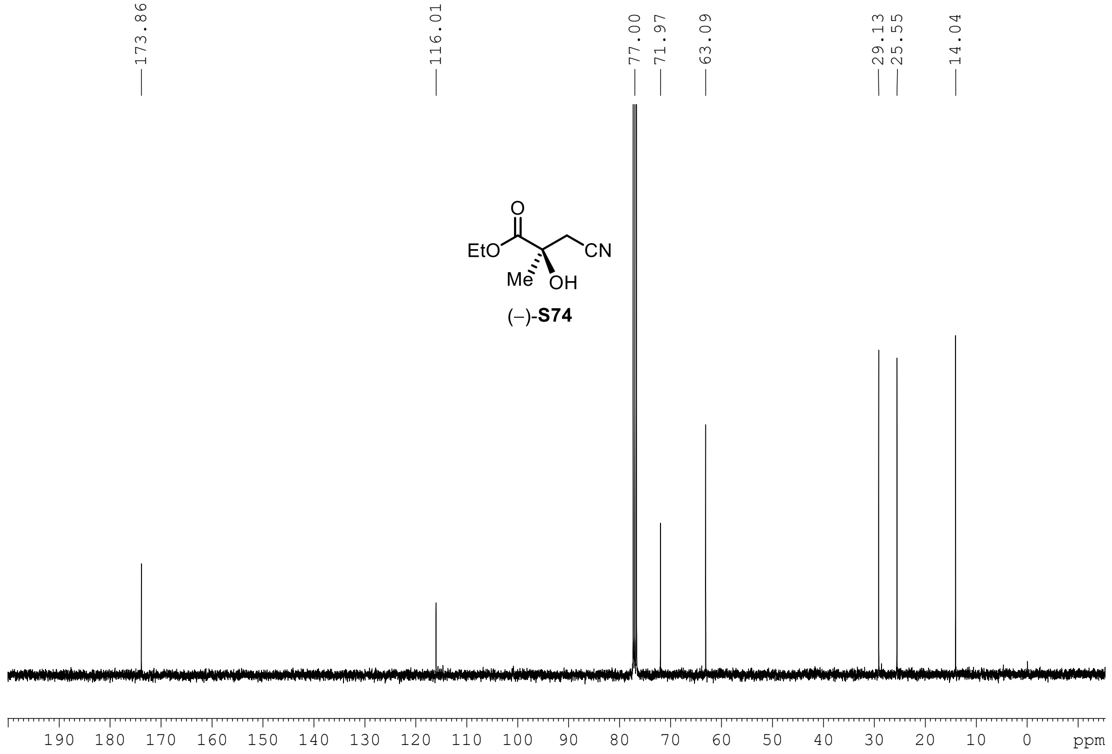

Supplementary Fig. 255. ^{1}H NMR of compound (–)-citramalic acid (500 MHz, CD_3OD, 25 °C)

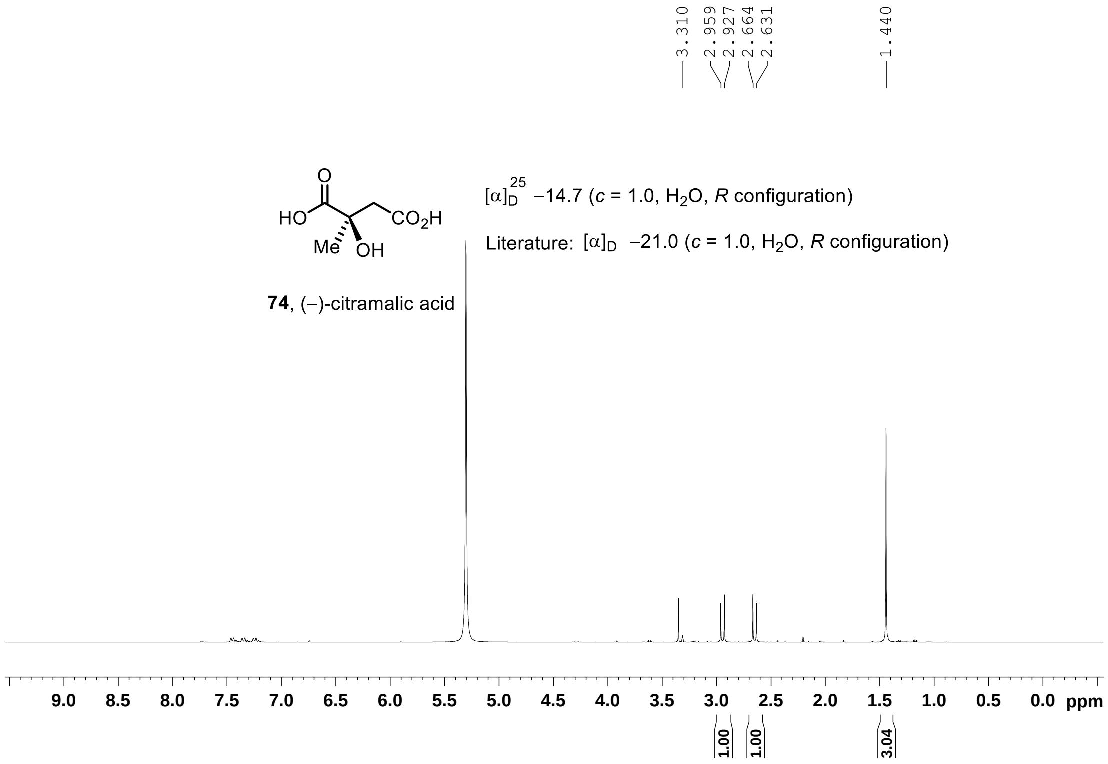

Supplementary Fig. 256. ^{13}C NMR of compound (-)-citramalic acid (125 MHz, CD_3OD, 25 °C)

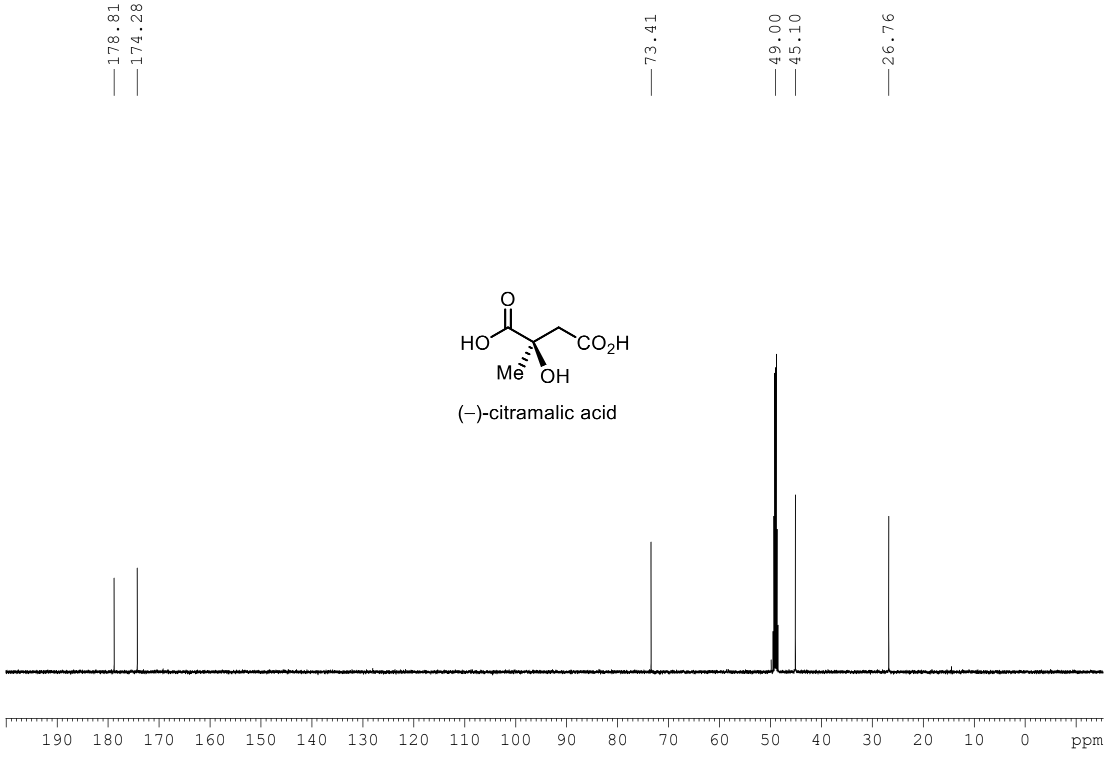

Supplementary Fig. 257. ^{1}H NMR of (–)-macahydantoin B ($CDCl_3$, 400 MHz, 25 °C)

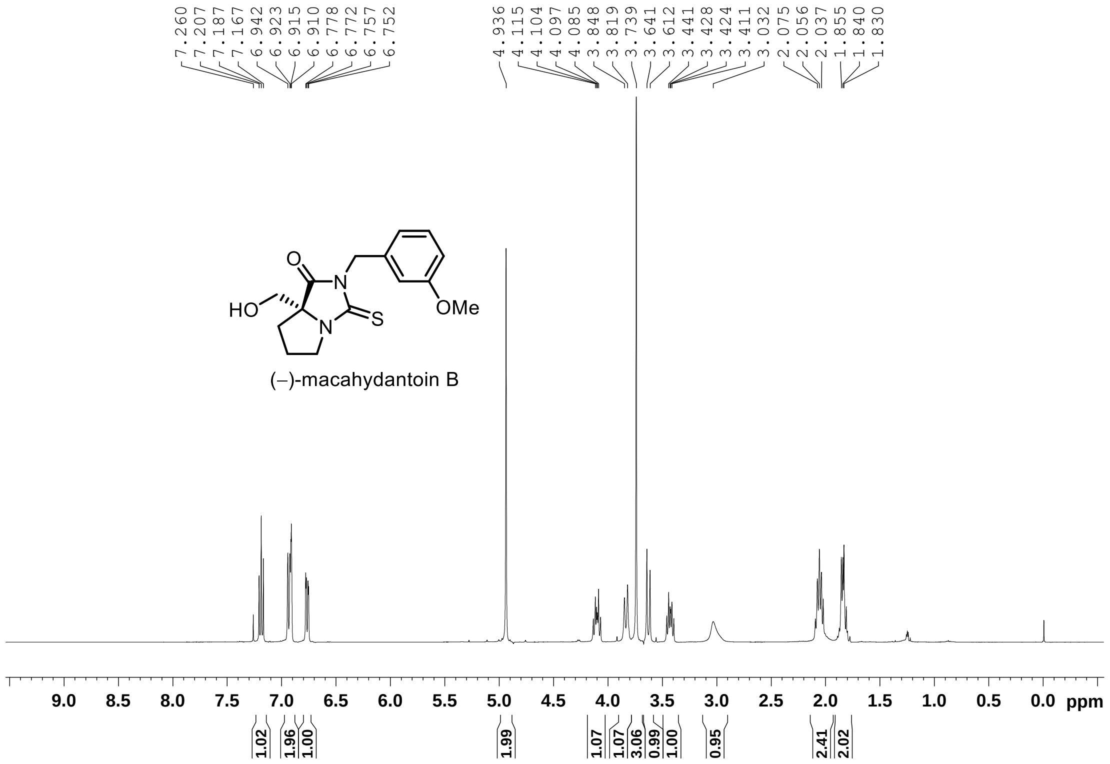

Supplementary Fig. 258. ^{13}C NMR of (–)-macahydantoin B ($CDCl_3$, 100 MHz, 25 °C)

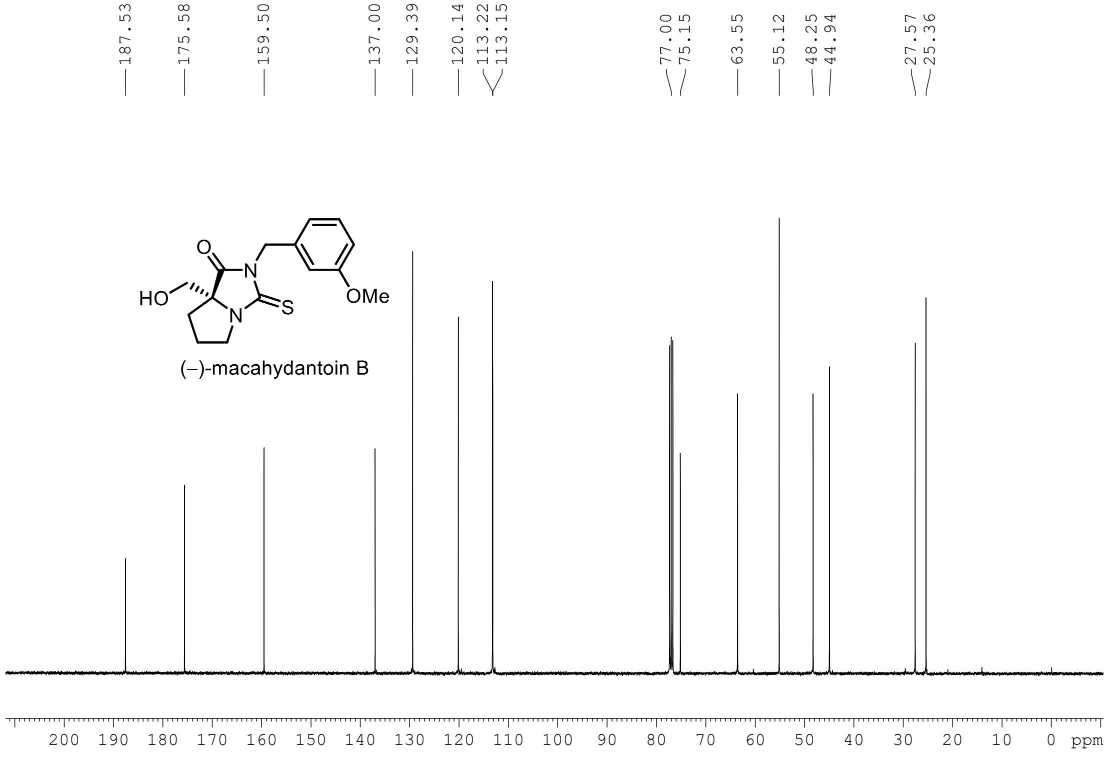

Supplementary Fig. 259. ^{1}H NMR of compound (+)-79 ($CDCl_3$, 400 MHz, 25 °C)

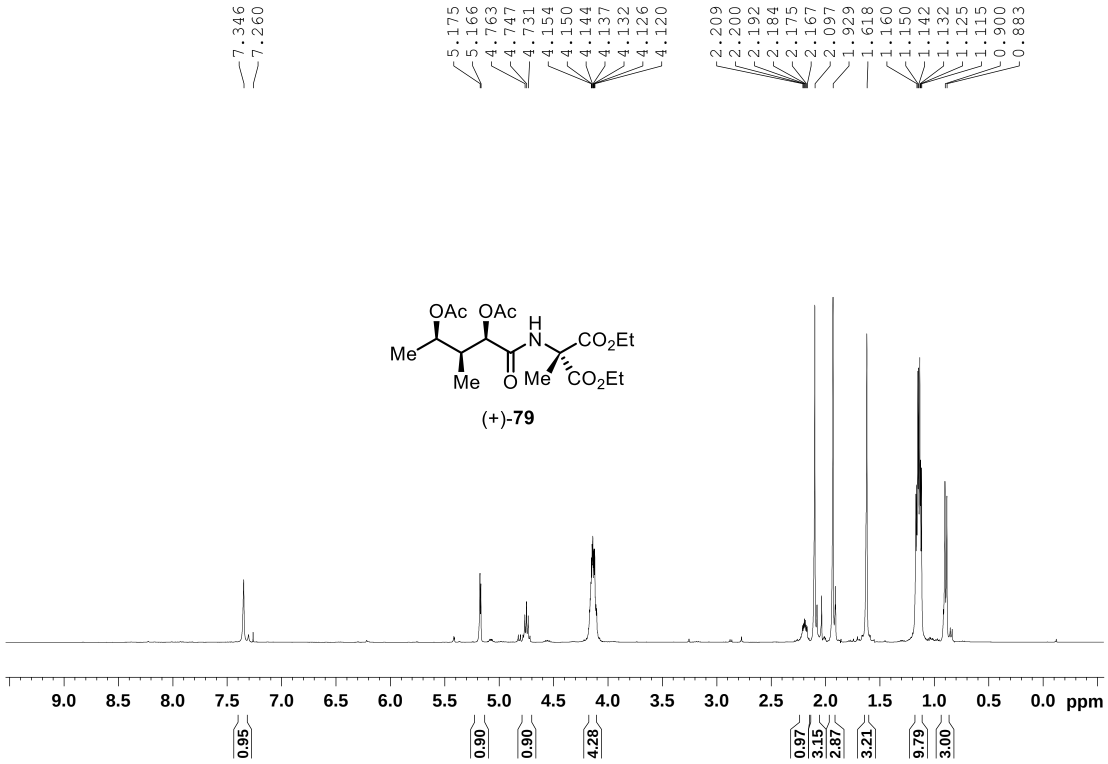

Supplementary Fig. 260. ^{13}C NMR of compound (+)-79 ($CDCl_3$, 100 MHz, 25 °C)

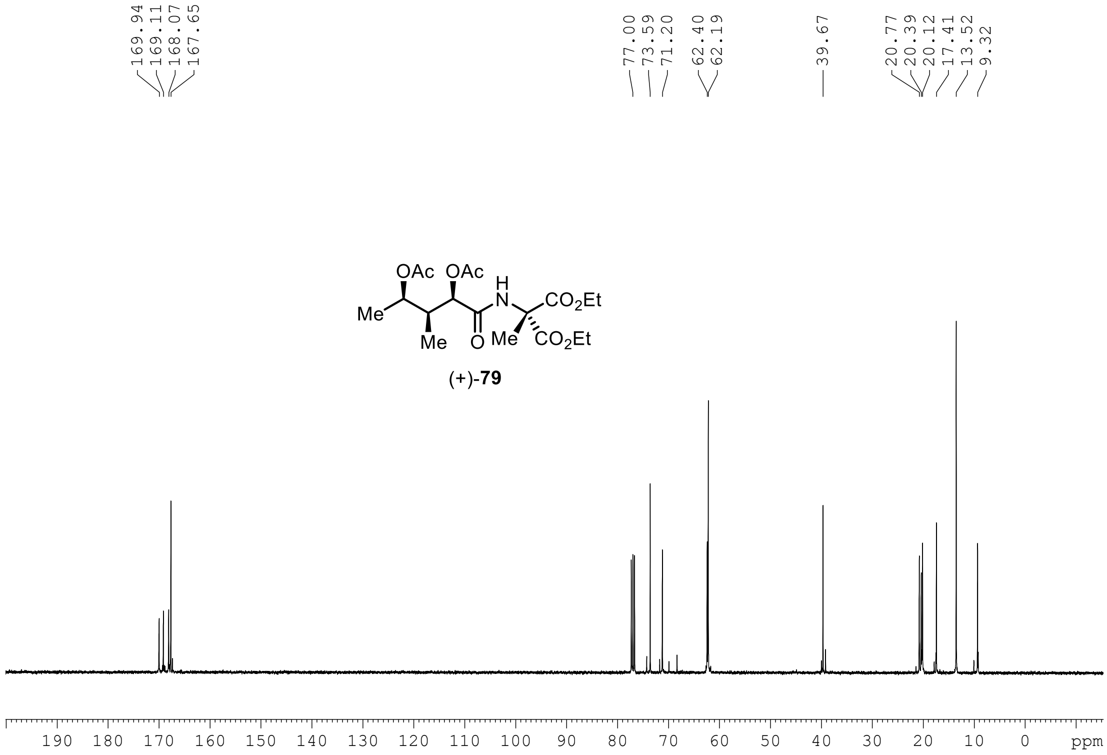

Supplementary Fig. 261. ¹H NMR of compound (+)-S80 ($CDCl_3$, 400 MHz, 25 °C)

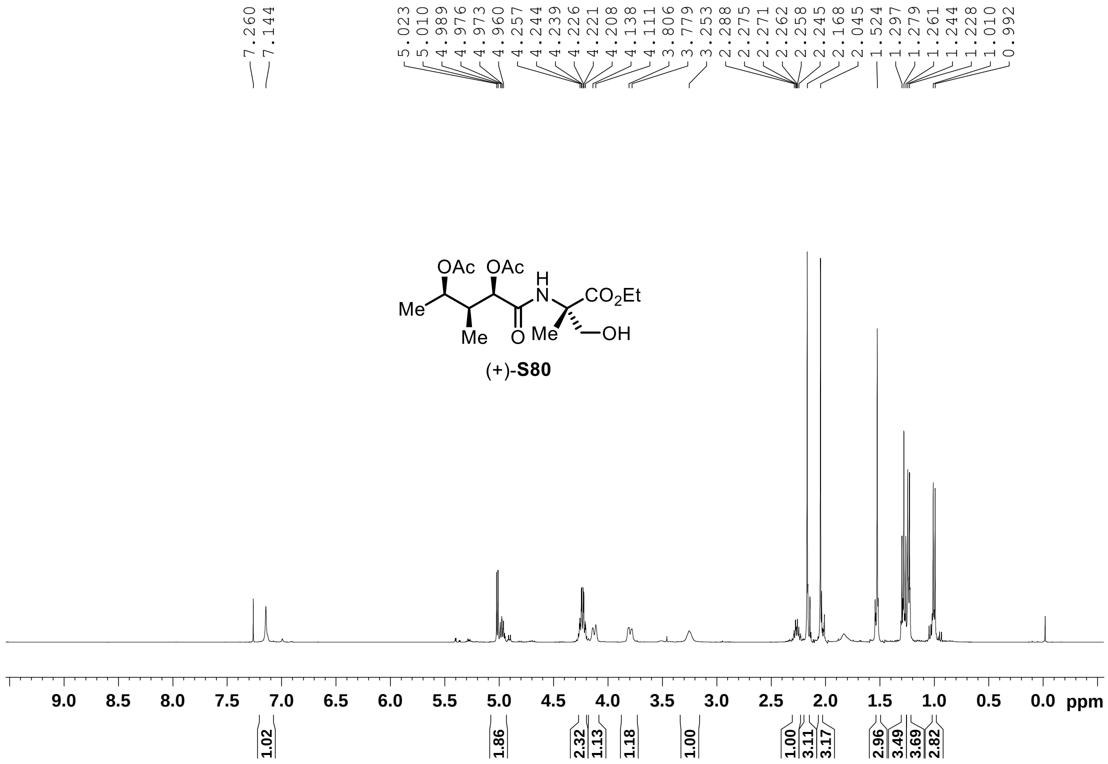

Supplementary Fig. 262. ¹³C NMR of compound (+)-S80 ($CDCl_3$, 100 MHz, 25 °C)

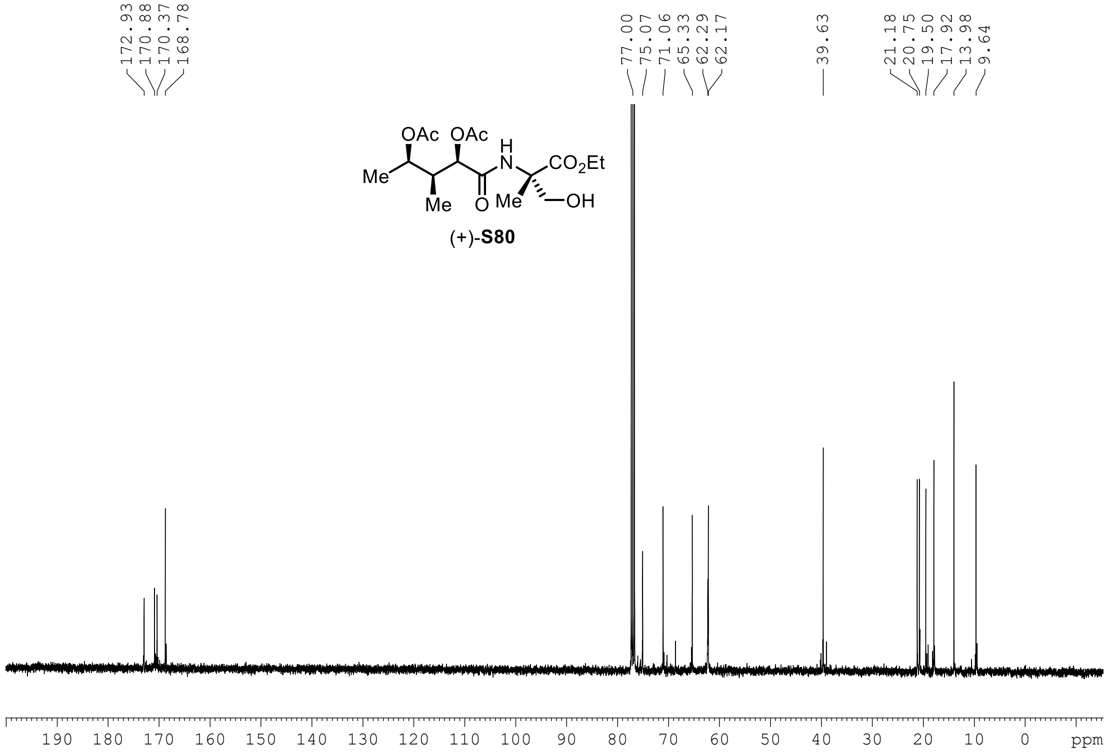

Supplementary Fig. 263. ^{1}H NMR of (+)-conagenin (CD_3OD, 400 MHz, 25 °C)

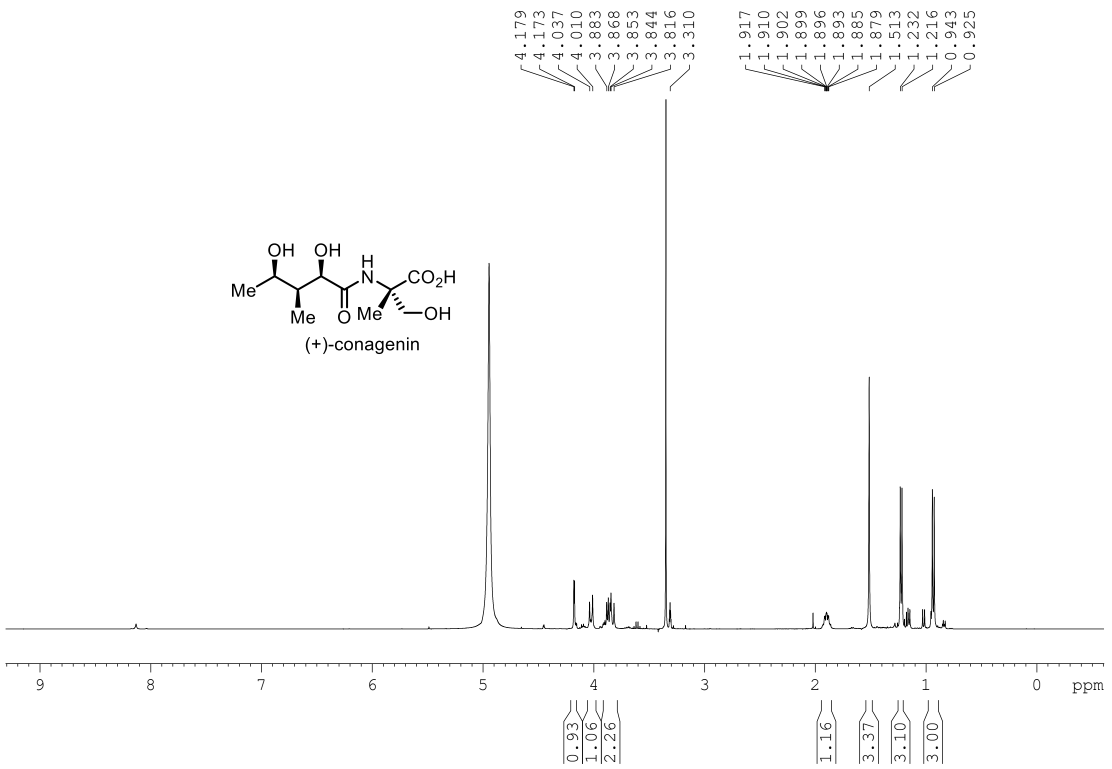

Supplementary Fig. 264. ^{13}C NMR of (+)-conagenin (CD_3OD, 100 MHz, 25 °C)

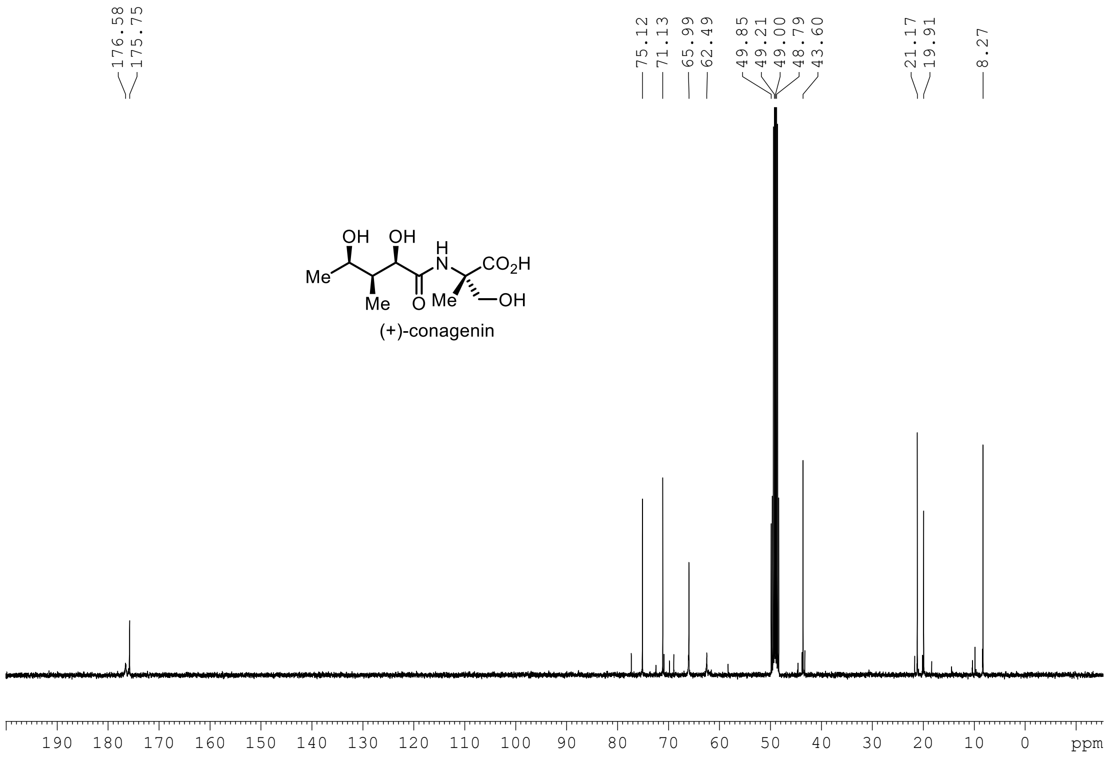

Supplementary Fig. 265. ^{1}H NMR of compound 82 ($CDCl_3$, 400 MHz, 25 °C)

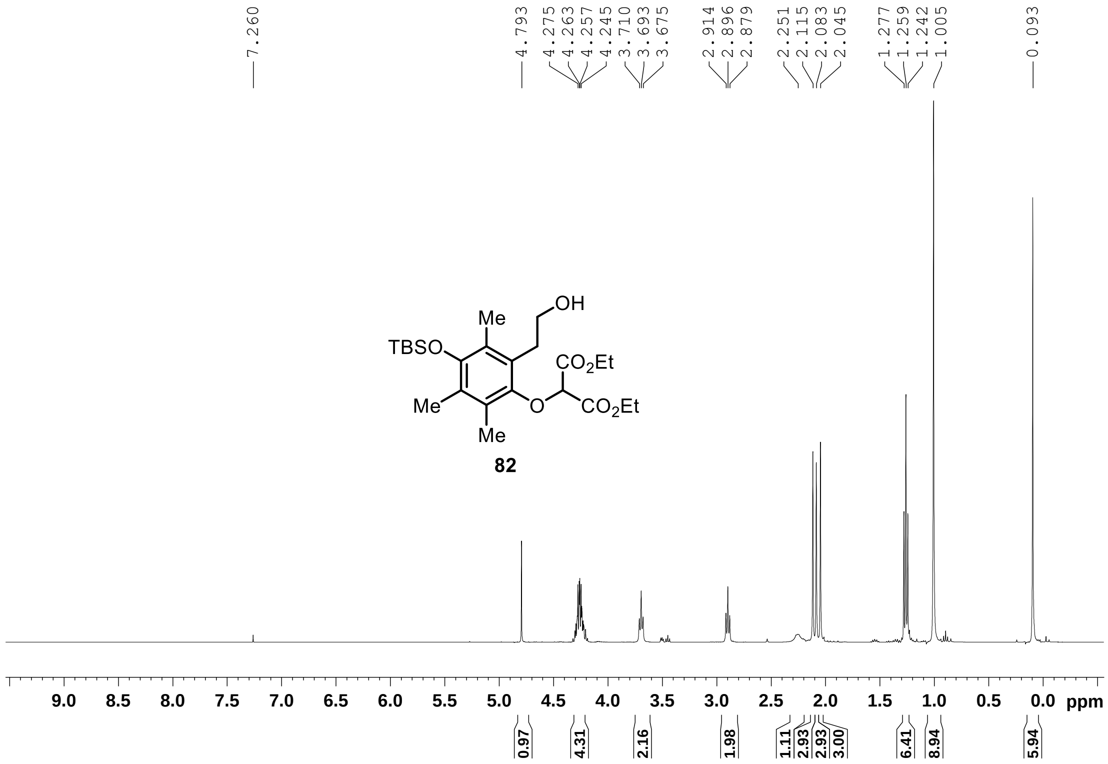

Supplementary Fig. 266. ^{13}C NMR of compound 82 ($CDCl_3$, 100 MHz, 25 °C)

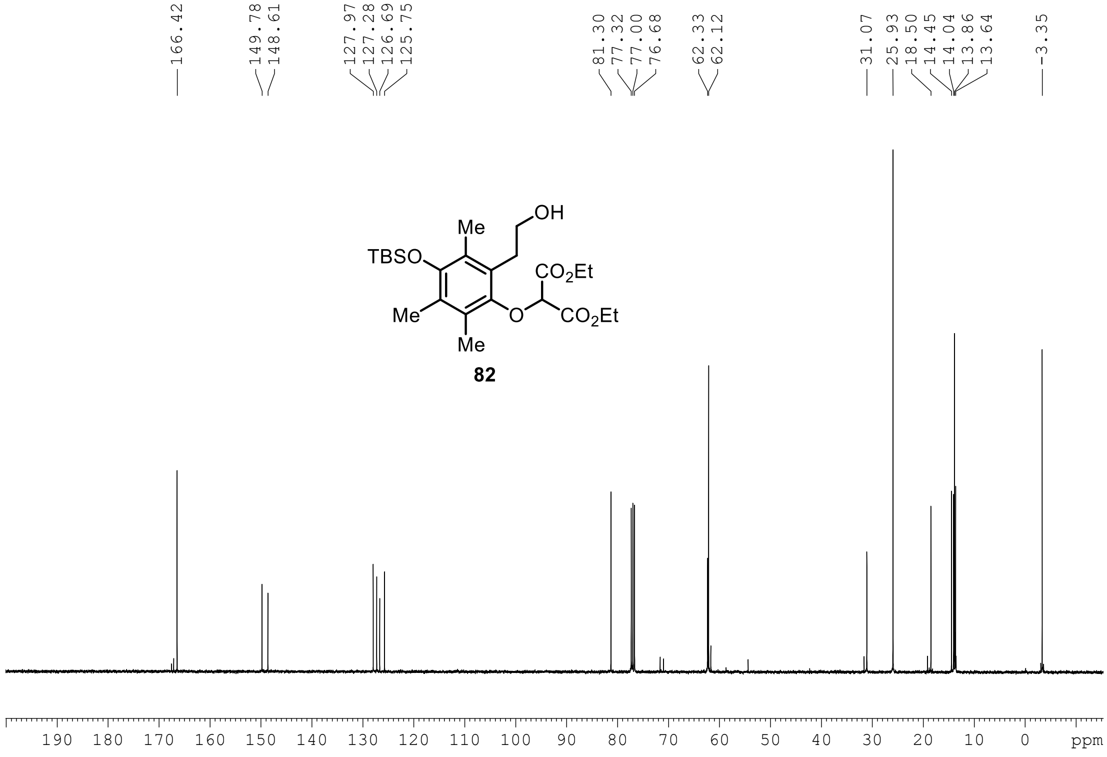

Supplementary Fig. 267. ¹H NMR of compound 83 ($CDCl_3$, 400 MHz, 25 °C)

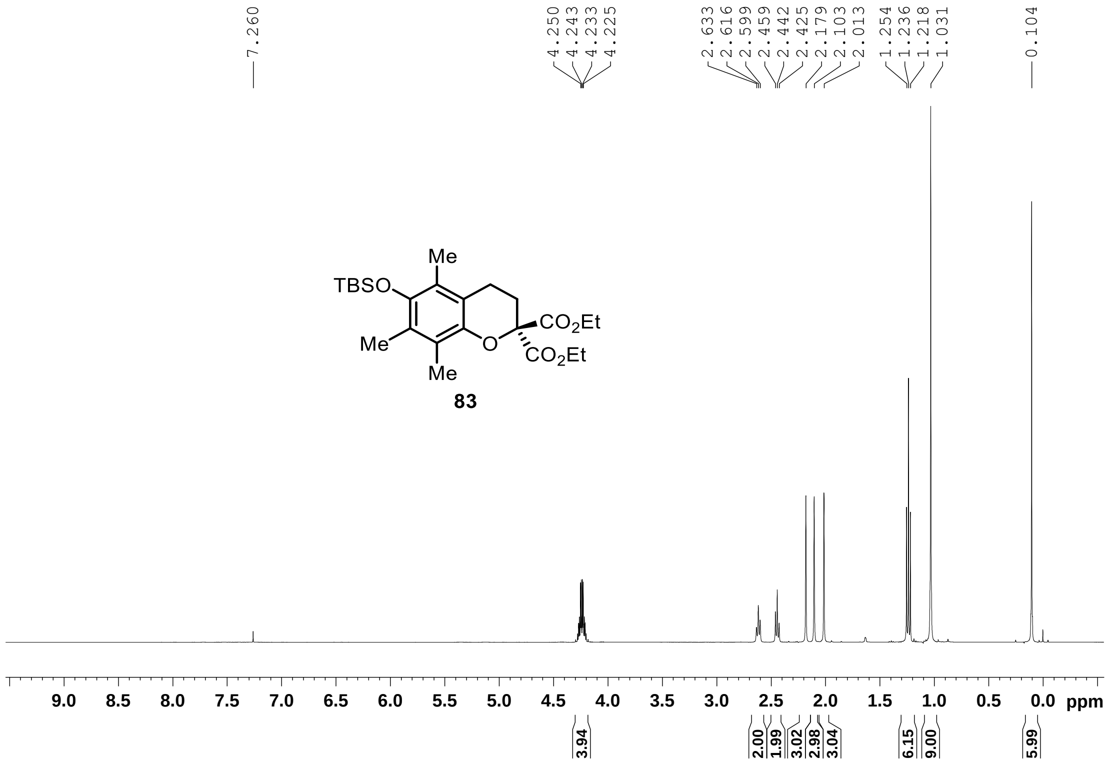

Supplementary Fig. 268. ¹³C NMR of compound 83 ($CDCl_3$, 100 MHz, 25 °C)

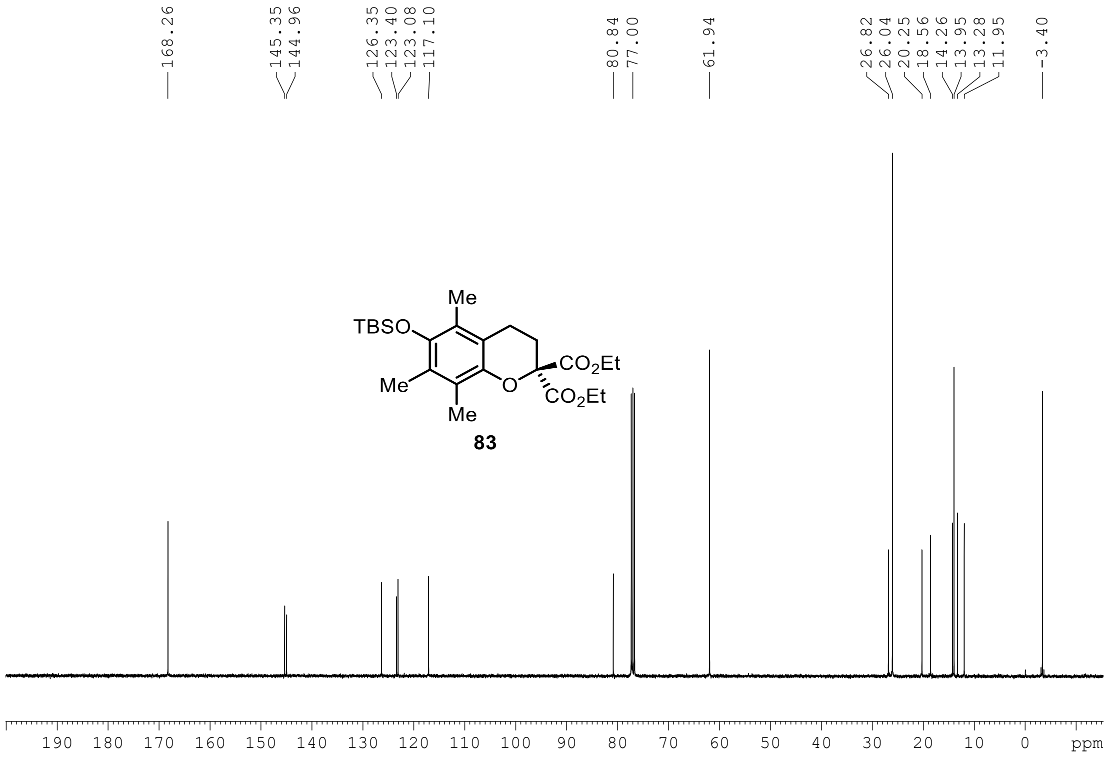

Supplementary Fig. 269. ¹H NMR of compound (–)-84 (CDCl₃, 400 MHz, 25 °C)

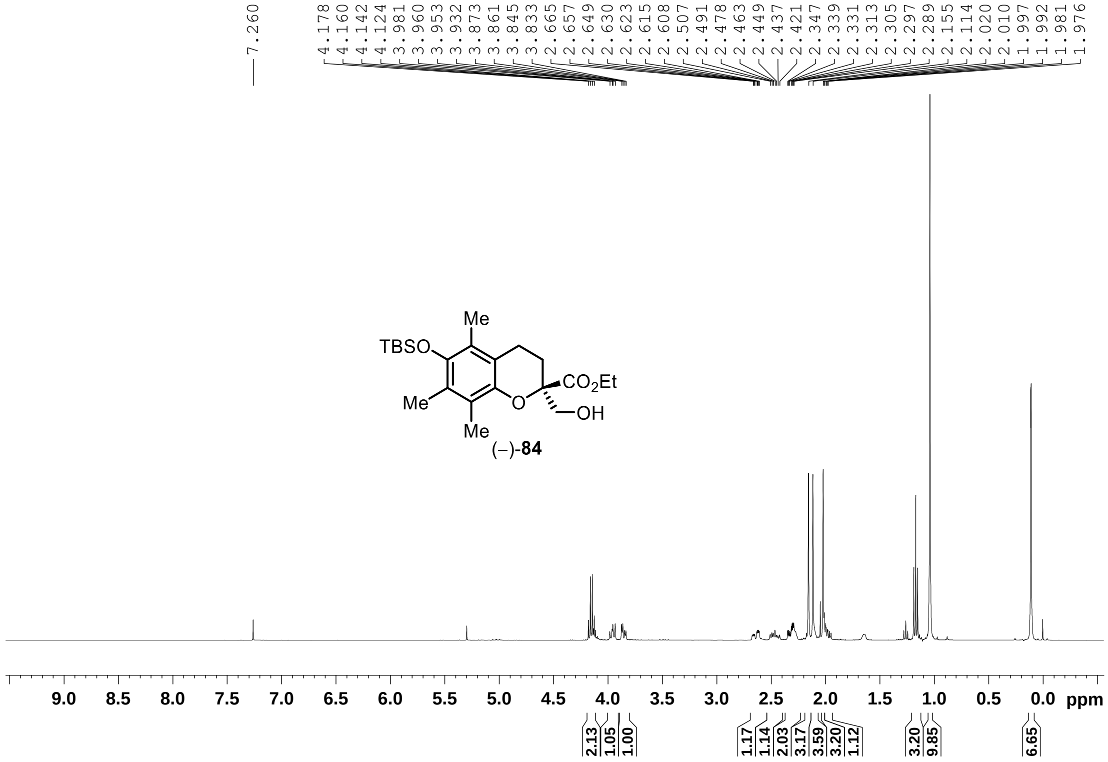

Supplementary Fig. 270. ¹³C NMR of compound (–)-84 (CDCl₃, 100 MHz, 25 °C)

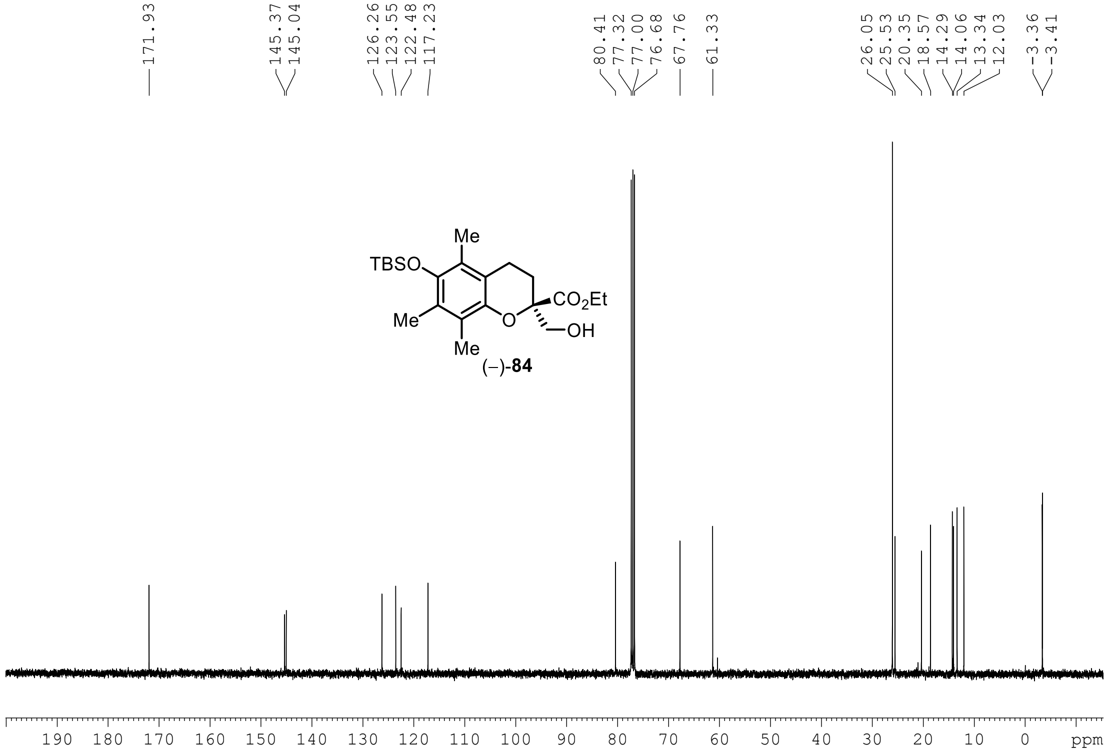

Supplementary Fig. 271. ^{1}H NMR of compound (−)-85 ($CDCl_3$, 400 MHz, 25 °C)

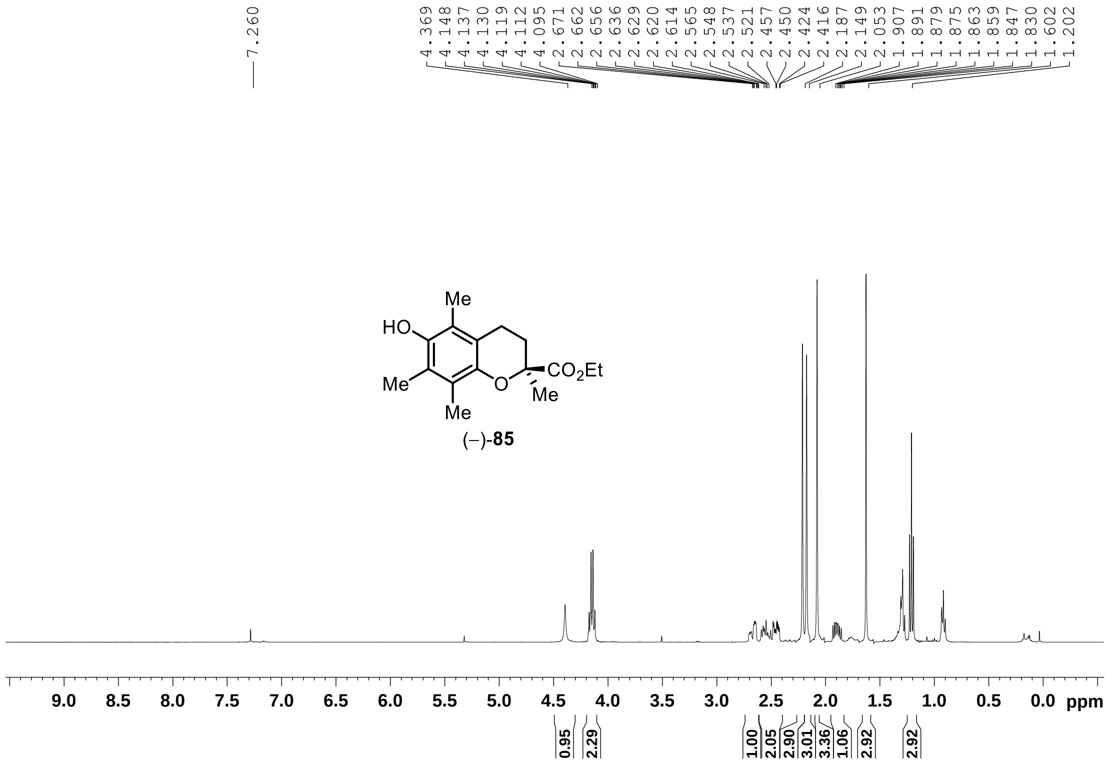

Supplementary Fig. 272. ^{13}C NMR of compound (−)-85 ($CDCl_3$, 100 MHz, 25 °C)

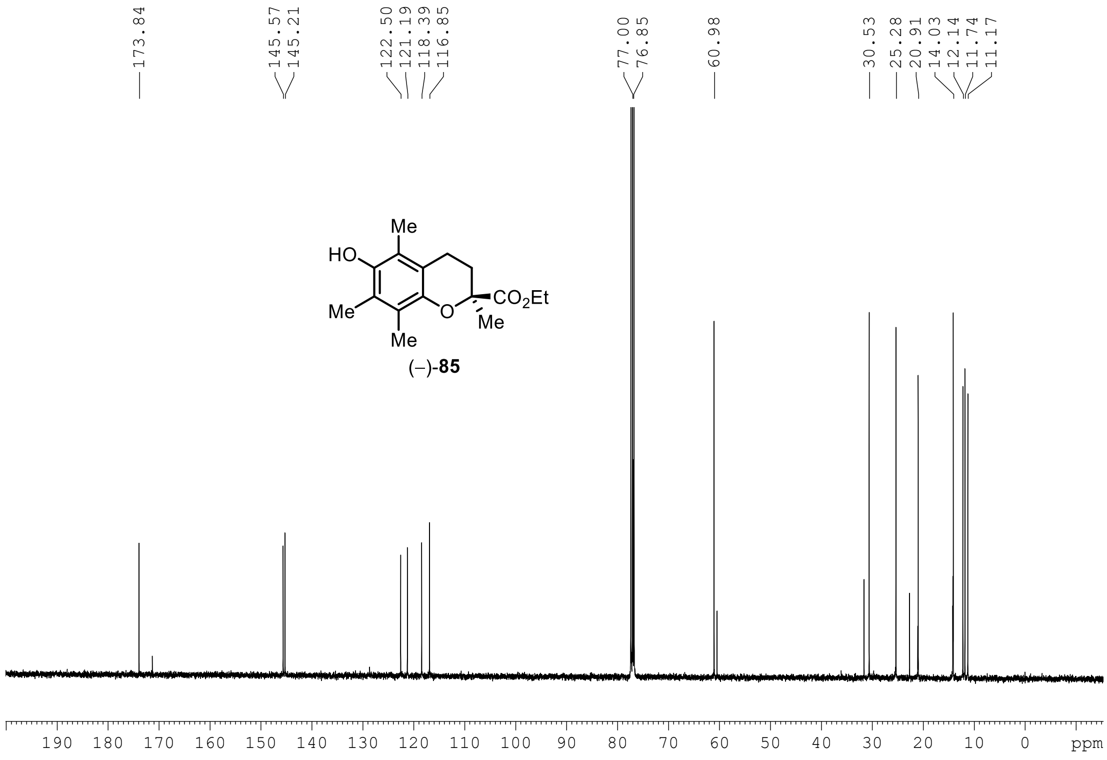

Supplementary Fig. 273. ^{1}H NMR of (–)-Trolox ($CDCl_3$, 500 MHz, 25 °C)

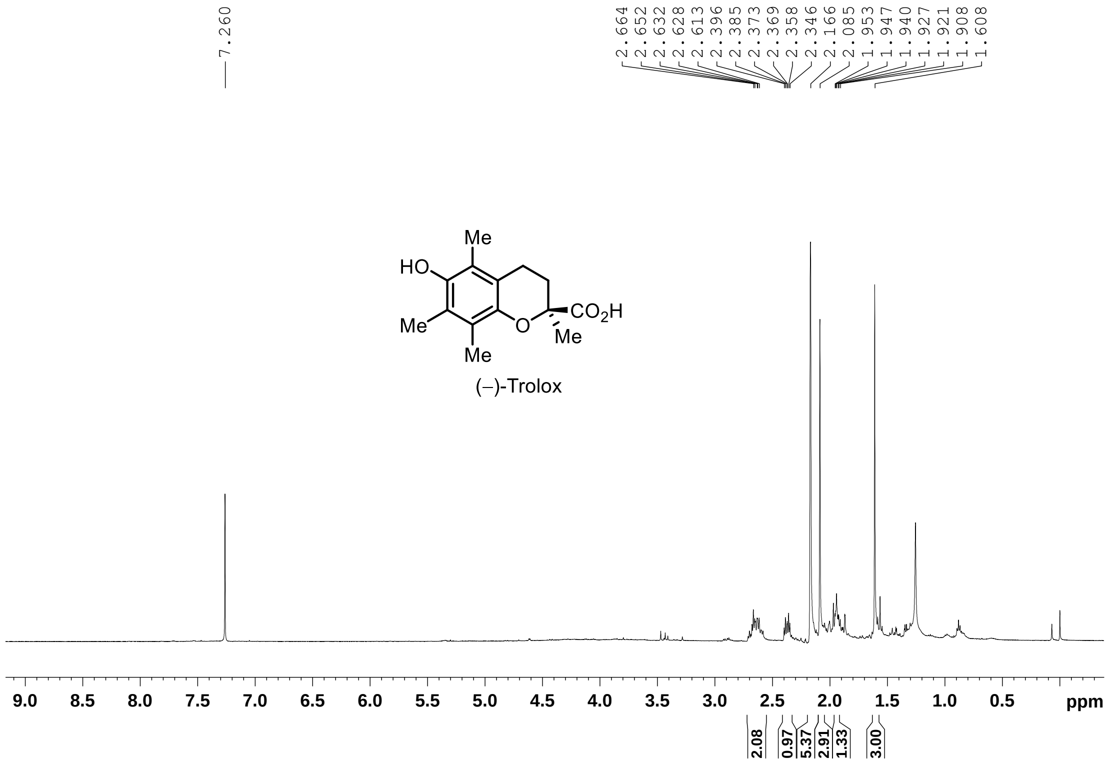

Supplementary Fig. 274. ^{13}C NMR of (–)-Trolox ($CDCl_3$, 125 MHz, 25 °C)

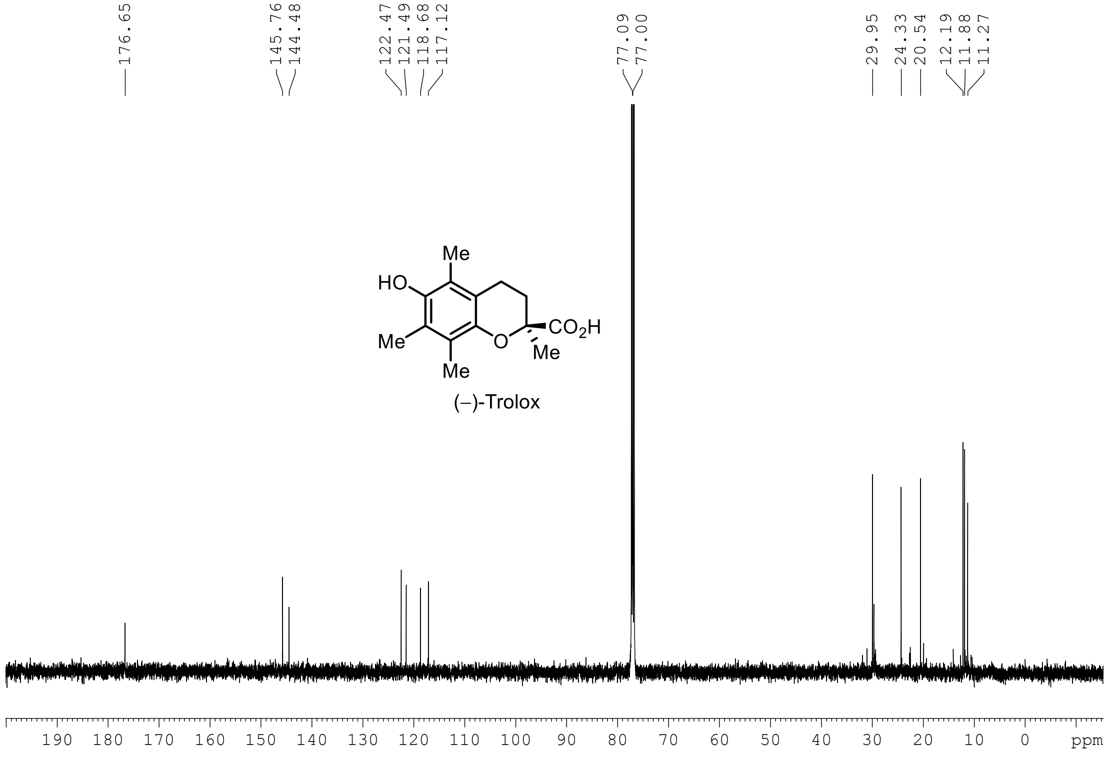

2. Supplementary references

1. Xu, P.; Huang, Z. Catalytic reductive desymmetrization of malonic esters. *Nat. Chem.* **2021**, *13,* 634−642.
2. Eschmann, C.; Song, L.; Schreiner, P. R. London Dispersion Interactions Rather than Steric Hindrance Determine the Enantioselectivity of the Corey–Bakshi–Shibata Reduction. *Angew. Chem. Int. Ed*. **2021**, *60*, 4823-4832.
3. Rocke, B. N.; Conn, E. L.; Eisenbeis, S. A.; Ruggeri, R. B. 1,4-Addition of an aryllithium reagent to diethyl ketomalonate. Scalable synthesis of ethyl 1-(hydroxymethyl)-1,3-dihydroisobenzofuran-1-carboxylate. *Tetrahedron Letters* **2012**. *53*, 5467-5470.
4. Meng, S. S.; Tang, W. B.; Zheng, W. H. Catalytically Enantioselective Synthesis of Acyclic α-Tertiary Amines through Desymmetrization of 2-Substituted 2-Nitro-1,3-diols. *Org. Lett*. **2018**, *20*, 518-521.
5. Smith, N. D.; Wohlrab, A.M.; Goodman, M. Enantiocontrolled synthesis of α-methyl amino acids via Bn_2N-α-Methylserine-β-lactone, *Org. Lett.* **2005**, *7,* 255-258.
6. Jew, S.; Kim, H.; Kim, J.; Park, H. Enantioselective synthesis of eucomols using Sharpless catalytic asymmetric dihydroxylation. *Heterocycles* **1997**, *46*, 65-70.
7. Ruano, J. L. G.; Fernández-Ibáñez, M. A.; Castro, A. M. M.; Ramos, J. H. R.; Flamarique, A. C. R. Regio- and stereocontrolled hydrocyanation of chiral 2-alkylglycidamides with Et_2AlCN: synthesis of enantiomerically pure mono- and disubstituted malic acid derivatives. *Tetrahedron: Asymmetry* **2002**, *13*, 1321-1325.
8. Zhou, M.; Ma, H.-Y.; Xing, H.-H.; Li, P.; Li, G.-P.; Geng, H.-C.; Hu, Q.-F.; Yang, G.-Y. Biomimetic Synthesis of Macahydantoins A and B from Lepidium meyenii, and Structure Revision of Macahydantoin B as a Class of Thiohydantoin with a 4-Methyl-hexahydropyrrolo[[1],2-c]imidazole Skeleton. *Org. Lett.* **2017**, *19*, 4952-4955.
9. Matsukawa, Y.; Isobe, M.; Kotsuki, H.; Ichikawa, Y. Synthesis of (+)-Conagenin. *J. Org. Chem.* **2005**. *70*, 5339-5341.
10. Sano, S.; Miwa, T.; Hayashi, K.; Nozaki, K.; Ozaki, Y.; Nagao, Y. Chemoenzymatic total synthesis of (+)-conagenin, a low-molecular-weight immunomodulator. *Tetrahedron Lett*. **2001**, *42*, 4029.
11. Uria, U.; Vila, C.; Lin, M.-Y.; Rueping, M. Gold Catalyzed Asymmetric Allylic Substitution of Free Alcohols - An Enantioselective Approach to Chiral Chromans with Quaternary Stereocenters for the Synthesis of Vitamin E and Analogues. *Chem. - Eur. J.* **2014**. *20*, 13913-13917.
12. Gilbert, J. C.; Pinto, M. Development of novel phenolic antioxidants. Synthesis, structure determination, and analysis of spiro[2,3-dihydro-5-hydroxy-4,6,7-trimethylbenzofuran-2,1'-cyclopropane]. *J. Org. Chem.* **1992**. *57*, 5271-5276.
